# Transcriptome analysis of *Azospirillum brasilense* vegetative and cyst states reveals large-scale alterations in metabolic and replicative gene expression

**DOI:** 10.1099/mgen.0.000200

**Published:** 2018-07-30

**Authors:** Elizabeth A. Malinich, Carl E. Bauer

**Affiliations:** ^1^​ Indiana University, USA; ^2^​ Department of Biochemistry, Indiana University Bloomington, Simon Hall MSB, Bloomington, IN 47405-7003, USA

**Keywords:** Gram-negative, differentiation, dormancy, cyst, *Azospirillum brasilense*, *Rhodospirillum centenum*

## Abstract

Several Gram-negative soil bacteria have the ability to differentiate into dormant cysts when faced with harsh environmental conditions. For example, when challenged with nutrient deprivation or desiccation, the plant-growth-promoting bacterium *Azospirillum brasilense* differentiates from a replicative and motile rod-shaped vegetative cell into a non-motile dormant spherical cyst. Currently, little is known about either the metabolic differences that exist between vegetative and cyst cell types, or about aspects of cyst physiology that allow dormant cells to survive harsh conditions. Here we compared transcriptomic profiles of vegetative and encysted *A. brasilense*. We observed that approximately one fifth of the *A. brasilense* transcriptome undergoes changes in expression between replicative vegetative cells and non-replicative cysts. A dramatic alteration in expression of genes involved in cell wall or cell membrane biogenesis was observed, which is congruent with changes in exopolysaccharide and lipid composition that occur between these cell types. Encysted cells also exhibited repressed mRNA abundance of genes involved in amino acid biosynthesis, ribosomal biogenesis and translation. We further observed that cysts create an anaerobic/micro-aerobic environment, as evidenced by repressed expression of oxidative phosphorylation genes coupled with increased expression of nitrate/nitrite reduction and nitrogen fixation genes.

## Data Summary

Sequence data presented in this publication have been deposited in NCBI’s Gene Expression Omnibus and are accessible through GEO Series number GSE104188.

**Impact Statement**

*Azospirillum brasilense* is a soil bacterium that can form a productive endosymbiotic relationship with a wide variety of crop plants. This bacterium can provide plants with fixed nitrogen as well as bacterially synthesized plant growth hormones that stimulate root development, thereby enhancing plant fitness and crop yields. *A. brasilense* vegetative cells, however, do not survive well within a biofertilizer matrix. It has been suggested that the application of encysted *A. brasilense* in biofertilizer will increase shelf life and effectiveness. Biofertilizer formulated with encysted *A. brasilense* will need to account for cyst physiology and environmental interaction in order to support a productive *A. brasilense* colonization of plants. This study provides a new understanding of cysts as a non-replicative, but still active, morphotype of *A. brasilense* and reveals an initial understanding of internal cyst physiology.

## Introduction

Many bacteria can differentiate into specialized morphotypes in response to changing environmental conditions. Such life-style switches allow survival in a diverse array of environments. One well-characterized example are Gram-positive endospores that can form under stressful conditions. Gram-positive spores are known to persist for decades and be resistant to such adverse environmental conditions as UV radiation, heat, starvation and dehydration. The genetic and physical processes of Gram-positive endospore development have been extensively studied [1–3].

A lesser-studied dormant cell state involves the development of cysts by various Gram-negative bacteria such as members of the genera *Azotobacter*, *Azospirillum*, *Ramlibacter* and *Rhodospirillum* [4–7]. As with endospores, cysts are metabolically dormant cells that exhibit resistance to desiccation, starvation and oxidative stress. Despite functional similarities, Gram-negative cysts are quite distinct from Gram-positive endospores. Developmentally, endospores form within a terminally differentiated mother cell that lyses to release the spore. In contrast, Gram-negative cysts do not form internally and instead develop by formation of a thick extracellular polysaccharide exine layer that protects cysts from desiccation [8, 9]. Cysts also store large quantities of poly-β-hydroxybutyrate granules for use as an energy source [10].

While cysts have been physiologically characterized in several species, little is known about the genetics of cyst development beyond a few studies on *Rhodospirillum centenum* encystment, which have revealed that the control of cyst development in *R. centenum* involves several input signals [11–19]. One signal for controlling *R. centenum* encystment involves the production of cGMP, which was not previously thought to occur in bacteria [15, 16]. During cyst development, *R. centenum* synthesizes and excretes large quantities of cGMP, which is sensed by the encystment master regulator CgrA that is an ortholog of the cAMP catabolite repressor protein CRP [17]. Although direct involvement of cGMP in *Azospirillum brasilense* cyst development remains to be established, the *A. brasilense* genome does code for genes involved in cGMP production as well as a homolog of CgrA, indicating that cGMP may also be a signal controlling encystment in this species.

This study undertakes to differentiate the vegetative transcriptome from the cyst transcriptome in *A. brasilense*. Members of the genus *Azospirillum* are plant-growth-promoting bacteria that are known to provide direct agricultural benefits by increasing crop yields (see reference [20] for a review). *A. brasilense* is noted for its secretion of several plant phytohormones that alter and enhance root development [21] across a broad range of plant hosts. Although *A. brasilense* is widely utilized as a bio-fertilizer, its usefulness is limited due to the short shelf life of replicative *A. brasilense* inside the bio-fertilizer mix. As cysts can survive for long periods in unfavourable conditions [4, 22, 23], the use of cysts as a seed inoculum may overcome this shelf-life problem. The effective use of *A. brasilense* cysts in bio-fertilizers, however, requires the ability to control the induction, yield and persistence of cysts [22, 23]. In this study, we compared the transcriptome of vegetative and encysted cells of *A. brasilense* with the intent of understanding cyst development and physiology under non-nutritive desiccated conditions. Such information is needed to understand the physiology of cysts at a level that would allow their use as a seed inoculum to enhance crop production.

## Methods

### Bacterial strain and growth conditions

*A. brasilense* Sp7 (ATCC 29145) was the wild-type parent strain used in this study. Vegetative *A. brasilense* cells were grown in nutrient broth (NB) with 50 µg ml^−1^ rifampicin for 48 h at 30 °C with shaking. Bacteria were streaked onto NB plates with no drug selection and incubated for 48 h at 30 °C, from these cultures single colonies were selected for RNA extraction. *A. brasilense* cysts were grown by culturing *A. brasilense* in 5 ml NB with 50 µg ml^−1^ rifampicin selection for 48 h at 30 °C. Aliquots of 1 ml were centrifuged at 6000 ***g*** for three minutes and the pellets re-suspended in the same volume of encystment media (salts amended with 8 mM fructose and 0.5 mM nitrate). The aliquots were incubated without shaking for 24 h at 30 °C to induce cysting. Finally, the encysted culture was placed onto an empty sterile Petri dish (no medium or agar), allowed to evaporate to dryness and then incubated under desiccation conditions at 42 °C for 1 week to kill all remaining vegetative cells [4]. A subset of desiccated cysts were checked for viability after heating for 7 day by cultivating on NB at 30 °C.

### RNA isolation and sequencing

Three biological replicates of vegetative and cyst cells were aseptically collected from plates and suspended directly into 750 µl lysis buffer composed of 35 mM sodium dodecyl sulfate, 100 mM β-mercaptoethanol, 300 mM guanidinium thiocyanate and 500 µg ml^−1^ lysozyme in TE buffer at pH 8.0. Samples were incubated at room temperature for 15 min before being transferred to an MP bead bashing tube with lysis matrix B. Samples were subjected to bead bashing using an MP Fast Prep for three repetitions of 20 s at speed 4.0 followed by a 30 s rest at 4 °C. Samples were centrifuged at 12 000 ***g*** for 5 min and the supernatant removed to a 1.5 ml RNase free microcentrifuge tube with 500 µl acid phenol : chloroform at 5 : 1 ratio. A volume of 500 µl of 100 mM sodium citrate with 2 % PEG 2000 was added. Samples were mixed by inversion, incubated in a 64 °C water bath for six minutes, inverting every minute and finally cooled down on ice bath for 2 min. Samples were then centrifuged at 12 000 ***g*** for 5 min. Supernatant was mixed with 500 µl chloroform: isoamyl alcohol (98 : 2, v:v) in a heavy phase lock gel tube (VWR, catalogue number 10847-802). Samples were inverted to mix and centrifuged at 12 000 ***g*** for 5 min. The upper phase was recovered and RNA precipitated by adding one quarter volume of 8 M LiCl and an equal volume of 100 % ethanol, followed by incubation at −20 °C overnight. Precipitated RNA was pelleted at 12 000 ***g*** for 30 min at 4 °C and re-suspended in 100 µl RNase free H_2_O. RNA was finally cleaned with a Qiagen RNA Extraction Kit with in-column DNase treatment according to the manufacturer’s protocol.

RNA was sequenced by the Indiana University Bloomington Center for Genomics and Bioinformatics. In brief, RNA was quality checked using an Agilent Tape Station and depleted of ribosomal RNA. cDNA libraries were prepared using a Bacterial Scriptseq Complete Kit (Illumina). Sequencing was performed with Illumina NextSeq on high output 75 cycle. Read depth was 10 million 50 bp reads per sample, equal to 75× genome coverage. For vegetative and cyst samples, 96.9±0.3 and 90.27±0.3 % respectively aligned back to the *A. brasilense* Sp7 genome (NCBI Assembly GCF_001315025.1).

### RNA-seq validation

RNA sequencing (RNA-seq) was validated by quantitative real-time polymerase chain reaction (RT-qPCR) of three biological replicates. A set of 11 genes were randomly selected as validation points, normalized to three housekeeping genes (Table S1 and Fig. S1, available in the online version of this article). Quantitative reverse transcriptase polymerases chain reaction (RT-qPCR) was performed with a StepOne quantitative thermocycler with SYBR Green high ROX master mix (Bioline). Thermocycling conditions were as follows: 96 °C for three minutes followed by 40 repetitions of 96 °C for 30 s and 50 °C for 10 s, which was immediately followed by a melting curve. Samples were run in triplicate.

### RNA seq data analysis

Reads were trimmed with Trimmomatic on a sliding window of 5:25, minimum read length of 40 bp. Processed reads were aligned to the *A. brasilense* Sp7 genome (NCBI Assembly GCF_001315025.1) with Bowtie2 [24]. HTSeq-count was used to quantify the gene counts [25]. DESeq2 package in R was used to normalize sample libraries and analyze significantly differentially expressed genes between the vegetative and cyst cell states (for a thorough description of DESeq2 methodology see references [26, 27]). Genes were considered to be significantly different with a adjusted *P*-value of <0.01 and at least a log_2_fold change of ≥3.

## Results

### Technical overview

RNA-seq transcriptome data from *A. brasilense* vegetative and cyst cells was generated in order to understand transcriptional differences between the two cell states (see Methods). Changes in individual gene expression were considered significant if there was a ≥3 log_2_fold mRNA abundance change in the cyst state compared with the vegetative state, with an adjusted *P*-value of <0.01. A significant gene is described as ‘repressed’ if there was a negative log_2_ fold change, indicating that the mRNA abundance of that gene was less in cysts as compared with vegetative cells. Likewise, genes are described as ‘elevated’ if there was a positive log_2_ fold change, indicating that mRNA abundance of a specific gene was greater in cysts as compared with vegetative cells.

**Fig. 1.**
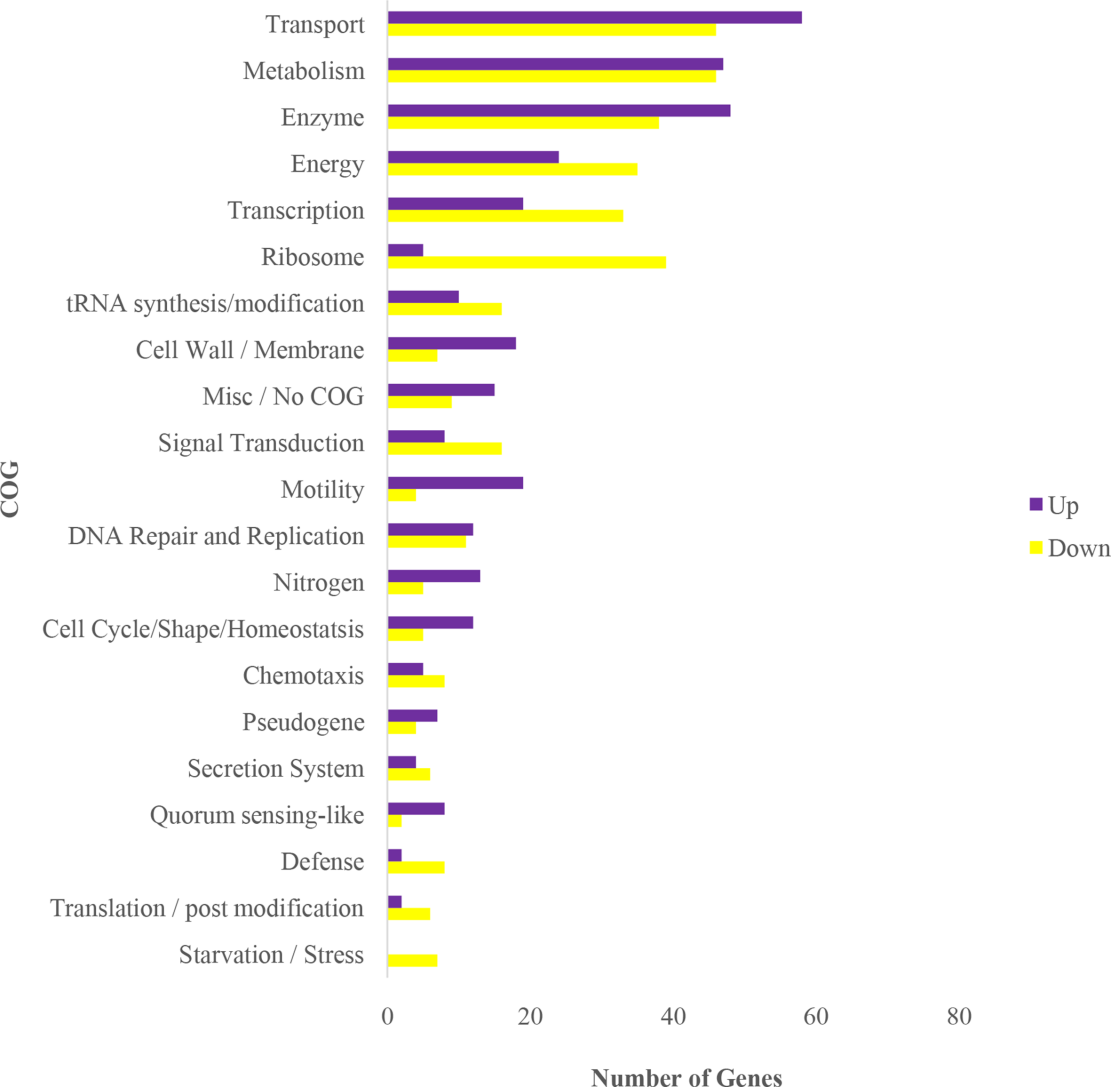
Differentially expressed genes (DEGs) were sorted into COGs (clusters of orthologous groups) based on the KEGG database. A total of 1011 DEGs were identified as significant between vegetative and cyst *A. brasilense*. Significant genes were considered to have a ≥ 3 log_2_fold change with a false-discovery-rate adjusted P-value of <0.01. DEGs were sorted into 21 COGs and the numbers of genes in each COG which were upregulated (top, purple bars) or downregulated (bottom, yellow bars) in cysts compared with vegetative cells are shown.

This screen resulted in 1011 genes, out of a total of 5859 annotated in the genome, that were scored as exhibiting significant changes in expression. Of those genes, 326 encoded hypothetical proteins with 137 repressed and 189 elevated in cyst cells relative to vegetative cells. The remaining 685 functionally annotated genes were manually sorted into 21 clusters of orthologous groups (COGs) based on the *A. brasilense* Sp7 Kyoto Encylopedia of Genes and Genomes orthologue and UniProt GO Biological process (Fig. 1). Multiple COGs were observed to be highly differentially regulated between cysts and vegetative cells, the details of which are highlighted below.

### Transcription and signal transduction

Given the large number of changes in gene expression that exist between vegetative and cyst cells, it was not surprising to find a large number of expression changes in transcription regulatory genes. Specifically, 52 transcription factors exhibited significant differences in expression between cyst and vegetative cells (Fig. S2). Of these genes, 33 were repressed and 19 were elevated in cysts when compared with vegetative cells. Of particular interest, were four elevated transcription factors that belong to the GntR/LacI family of regulators (AMK58_05505, AMK58_26435, AMK58_22705 and AMK58_14110). The GntR/LacI family of transcription factors are involved in gluconate metabolism in the Entner–Doudoroff pathway. Repressed transcription factors included a repressor of polyhydroxyalkanoate (PHA) biosynthesis (AMK58_26785). PHA is interesting because it is the parent form of poly-β-hydroxybutyrate (PHB) and is known to accumulate to high levels in cyst cells, where it may serve as an energy source [28, 29].

A total of 24 genes involved in signal transduction were also identified as being differentially expressed between cyst and vegetative cells (Fig. S3). Many of these signal transduction genes control processes related to the various COGs, as discussed below. For example, cell cycle signal transduction genes (AMK58_19580, AMK58_16040 and AMK58_08720) were repressed in cysts, as were a number of genes involved in the cell cycle (Data S1). Likewise, a response regulator transcript (AMK58_21415, NasT) required for nitrogen fixation and growth in the presence of nitrate was elevated in cysts as was also the case for several *nif* genes.

There were eight histidine kinases/response regulators without known functions that were highly differentially regulated in the encysted versus vegetative cell states. Five of these genes were repressed (AMK58_05345, AMK58_08865, AMK58_17100, AMK58_23595 and AMK58_21530) and three were elevated (AMK58_02315, AMK58_18075 and AMK58_13080). AMK58_13080 was one of the most elevated (6.42 log_2_ fold change) during encystment and also contains a GGDEF domain, indicating probable involvement in the synthesis of c-di-GMP. In this regard we also observed that two additional di-guanylyl cyclases (AMK58_02945 and AMK58_12665) and one di-cyclic-GMP binding protein (AMK58_18090) were also differentially regulated, as was also the case for an adenyl cyclase (AMK58_04750). *A. brasilense’s* close neighbor, *R. centenum*, also differentially regulates several di-guanylyl cyclases during induction of its cysting state [18]. Thus, the role of c-di-GMP in regulating encystment warrants further scrutiny and experimentation.

### Ribosome biogenesis and translation

**Fig. 2.**
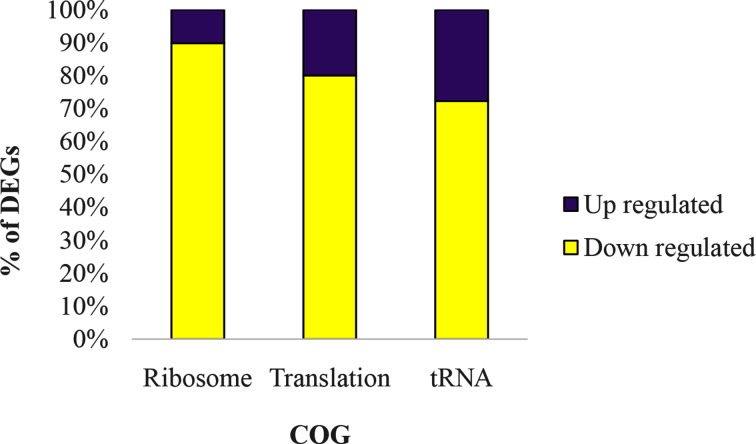
Ribosome biogenesis, translation and tRNA modification are transcriptionally downregulated in cysts. Data is shown as the percentage of DEGs in each COG which were upregulated or downregulated in cysts compared with vegetative cells.

Previously it has been shown that *Azospirillum* cysts have reduced protein content [10]. This observation matches our transcriptome results, which showed repression of genes involved in ribosome biogenesis, translation and tRNA expression/modification (Fig. 2; Data S1). Not all tRNA genes were repressed, however, as those tRNAs that attach to methionine, lysine and glutamine were elevated. The specific role of these three elevated tRNAs in cyst development is unclear. Genes involved in translation, such as elongation factors Tu and G, were also transcriptionally repressed (Fig. 2). In congruence with an overall reduction in protein synthesis, we also observed six genes involved in amino acid metabolism that were repressed (Data S1). Reduced protein synthesis in *Azospirillum* cysts is also consistent with our previous transcriptome study on *R. centenum* cysts [19].

### Cell cycle, DNA replication, maintenance and repair

**Fig. 3.**
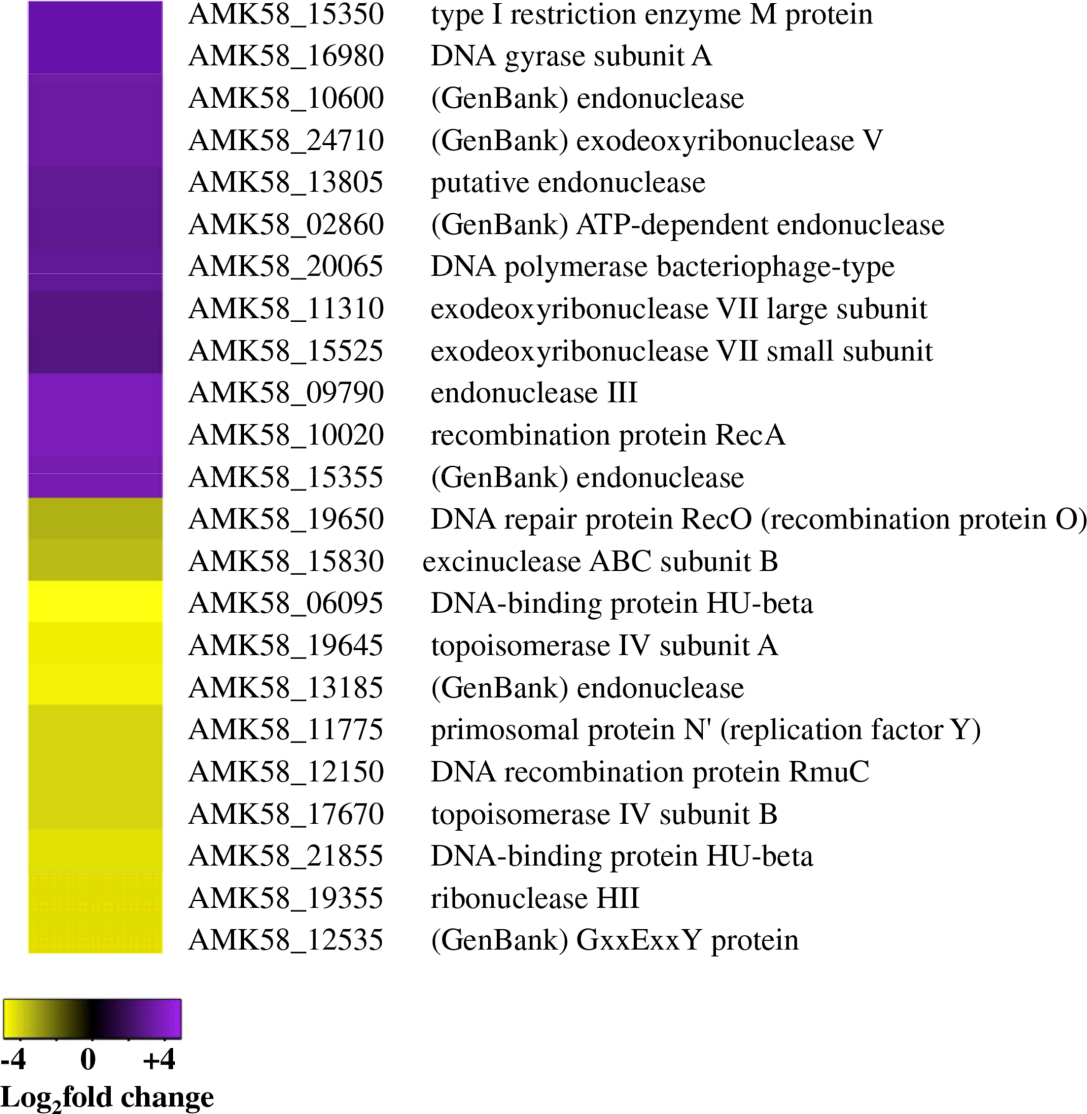
Cysts suppress DNA replication while elevating DNA repair expression. Heat maps display the DEGs belonging to the DNA repair and replication and cell cycle COGs. Purple indicates upregulated and yellow indicates downregulated DEGs in cysts as compared with vegetative cells.

Given that cysts are a dormant non-replicative form of *Azospirillum,* it was not surprising to see that genes involved in cell cycle, DNA replication and DNA maintenance and repair were altered in cyst cells (Data S1). Six genes participating in cell division were identified as significantly regulated in cysts. Three transcriptionally repressed genes included those involved in septation ring formation (AMK58_17660), chromosome partitioning (AMK58_ 20365) and cell division topological specificity (AMK58_ 04425). However, genes for a hypothetical chromosome partitioning protein (AMK58_ 21450), GTP binding protein (AMK58_12430) and circadian clock protein (AMK58_25610) were elevated. A total of 23 genes involved in DNA replication and repair were differentially expressed between vegetative and cyst cells (Fig. 3). Specifically, genes involved in DNA replication initiation and elongation (AMK58_19645, AMK58_19355, AMK58_17670, AMK58_12150, AMK58_06095 and AMK58_21855) were generally repressed, while many genes involved in DNA repair were elevated. This indicates that cysts prioritize the maintenance of DNA integrity over DNA replication, which is suppressed.

### Energy generating processes

**Fig. 4.**
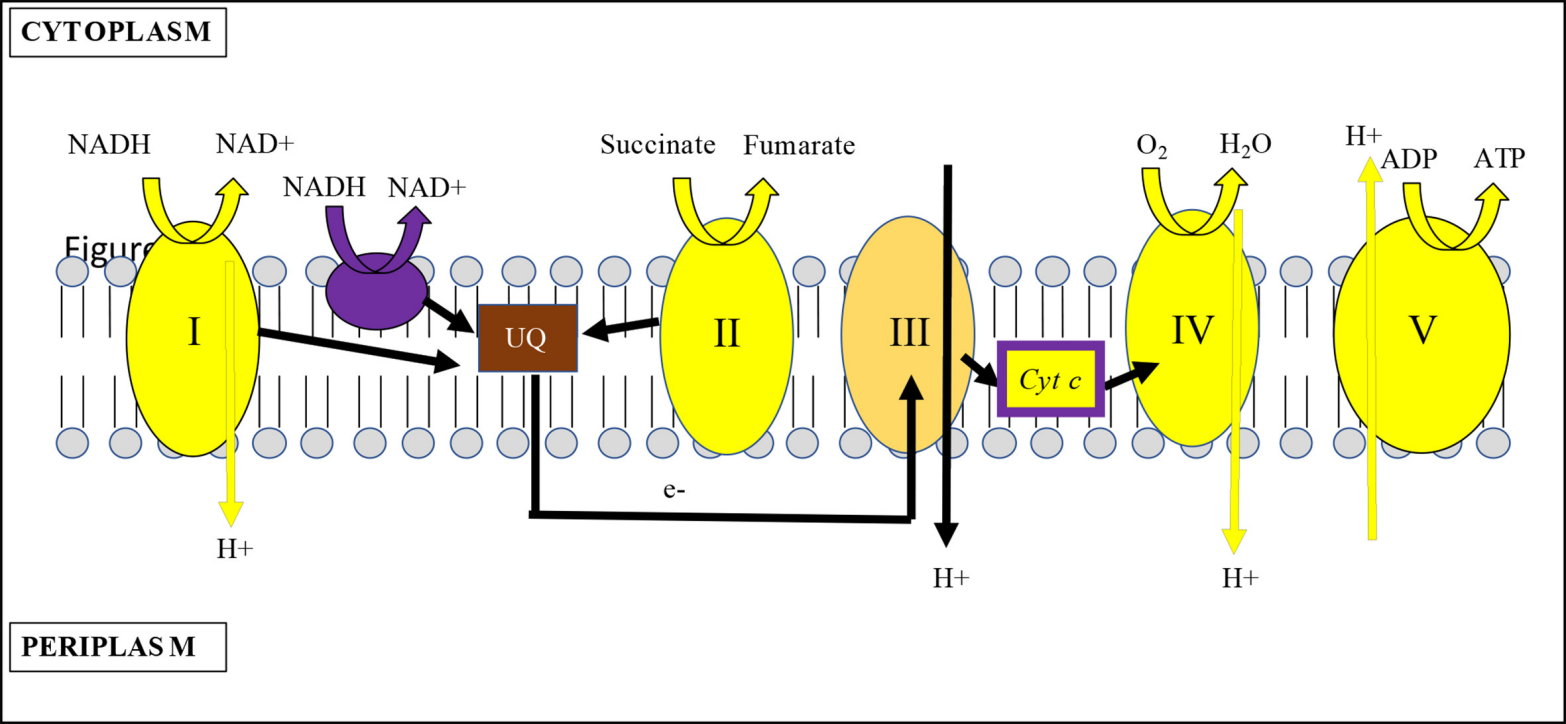
Membrane polarization is transcriptionally repressed in cysts. Schematic displays of the electron transport chain in an encysted *A. brasilense* cell. Typical bacterial electron transport chain coded in the A. *brasilense* genome. Processes in yellow are downregulated while those in purple are upregulated in cyst cells as compared with vegetative cells. Cyt c is colored both yellow and purple to denote that there is mixed regulation of cytochrome *c* genes. I, NAD(P)H-quinone oxidoreductase complex; II, succinate dehydrogenase complex; III, cytochrome *bc_1_* complex; IV, cytochrome c oxidase complex; V, ATPase complex; UQ, ubiquinone; H +, proton; *Cyt c,* cytochrome C; arrows denote direction of electron flow.

Analysis of genes encompassing the energy generation COG shows that cysts display a drastically different energy transcriptome from vegetative cells. Notably, the expression of many high-energy-generating processes, such as oxidative phosphorylation, are attenuated in cyst cells (Fig. 4). For example, there was a three to eight log_2_ fold repression of an operon that codes for 11 type 1 NAD(P)H-quinone oxidoreductase subunits, a gene coding for succinate dehydrogenase, 11 respiratory cytochrome genes and four genes coding for ATPase subunits (Fig. S4). As shown in Fig. 4, type I NAD(P)H-quinone oxidoreductase (complex I) and succinate dehydrogenase (complex II) both shuttle electrons to ubiquinone to generate reduced ubiquinol. These ubiquinol electrons can be directly involved in reduction of oxygen to water via ubiquinol (*cbb_3_* type) cytochrome oxidase or shuttled to cytochrome c oxidase (complex IV) via additional electron transfer components involving cytochrome *bc*_1_ (complex III) and cytochrome *c*. Note that electron transfer among complex I, III and IV all result in transduction of protons across the membrane, generating a membrane potential that is used to drive ATP production by ATPase (complex V) (Fig. 4). In cyst cells, there was significant reduction of transcripts coding for subunits of complex I and IV. This would lead to membrane depolarization coupled with reduced need for ATPase, which is manifested by concurrent reduction of ATPase gene expression. Interestingly, cyst cells appeared to retain a reduced ubiquinone pool via increased expression of three type II NADH dehydrogenase genes (AMK 58_27485, AMK 58_19750 and AMK 58_17980). Unlike type I NAD(P)H-quinone oxidoreductases, which translocate protons [30], type II NADH dehydrogenases reduce ubiquinone to ubiquinol without proton translocation across the membrane [31]. Presumably the increased expression of these type II NADH dehydrogenases reflects a need to keep the ubiquinone pool reduced without concomitant membrane polarization. This nascent ubiquinol-reducing power may be used to scrub the cells of residual oxygen via ubiquinol (*cbb_3_* type) cytochrome oxidase, which was upregulated. This oxidase is thought to have a high affinity for oxygen allowing respiration under low-oxygen conditions [32].

The hypothesis of low oxygen levels in cyst cells is also supported by elevated expression of numerous genes involved in anaerobic respiration (Fig. S4). This included genes involved in transport of nitrate/nitrite (AMK58_21400, AMK58_21405 and AMK58_21410) as well as nitrite reductase (AMK58_21395). Indeed, *Azospirillum* is known to anaerobically respire by reduction of nitrate to nitrite to nitrous oxide [33, 34]. Nitrate reduction provides roughly half the energy of aerobic phosphorylation while reduction of nitrite to nitrous oxide is two-thirds as effective [35]. In addition, there was also elevated expression of several genes involved in the transport (sulfate transport permease, AMK58_20635 and AMK58_20640) and reduction (sulfite reductase, AMK_08205) of oxidized sulfur compounds (Fig. S4). Cyst cells additionally have elevated expression of an alternative terminal tetrathionate/DMSO reductase (AMK58_25705, AMK58_24705, AMK58_25700, AMK58_24700 and AMK58_24695). Thus, while cysts exhibit repression of several oxidative phosphorylation processes, at the same time they elevate several anoxygenic nitrogen and sulfur energy-generating processes.

### Enzymes

Enzymes made up a large portion of the differentially expressed transcripts between cysts and vegetative cells. Enzymes with an annotated specific function were assigned to their cognate COGs, whereas enzymes lacking a specific functional purpose, and that could not be reliably identified, were grouped into a generic ‘Enzymes’ COG (Fig. S5). Dehydrogenases, dehydratases and peptidases were overall repressed, while hydrolases were generally elevated in cysts. Glycosyl transferases, often involved in lipid metabolism, were also primarily elevated in cysts (discussed later).

**Fig. 5.**
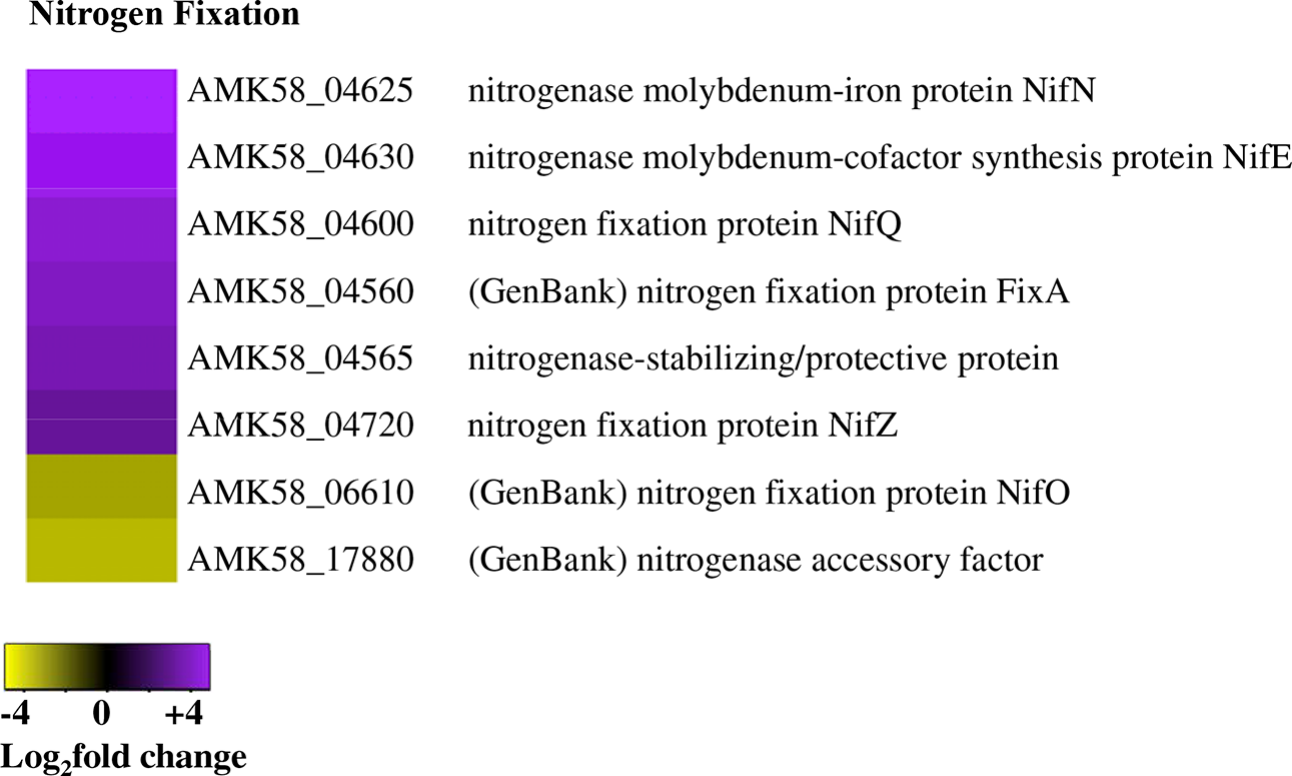
Nitrogenase genes associated with co-factor biosynthesis are upregulated in cysts. The heat-map displays the DEGs involved in nitrogen fixation from the nitrogen COG. Purple indicates upregulated and yellow indicates downregulated DEGs in cysts as compared with vegetative cells.

Expression of the nitrogenase and related enzymes were particularly interesting given their role in nitrogen fixation. Expression of most *nif* genes, including an operon that codes for the nitrogenase structural genes NifHDK, occurred at similar levels in both cell types (Data S1). However, NifQ (AMK58_04600), NifE (AMK58_04630), NifN (AMK58_04625), which are involved in insertion of the molybdenum MoFe_3_S_4_ cluster into the nitrogenase enzyme [36], were highly expressed in cyst cells. Furthermore, several nitrogenase stability and accessory factors were elevated in cysts (Fig. 5).

### Metabolism

**Fig. 6.**
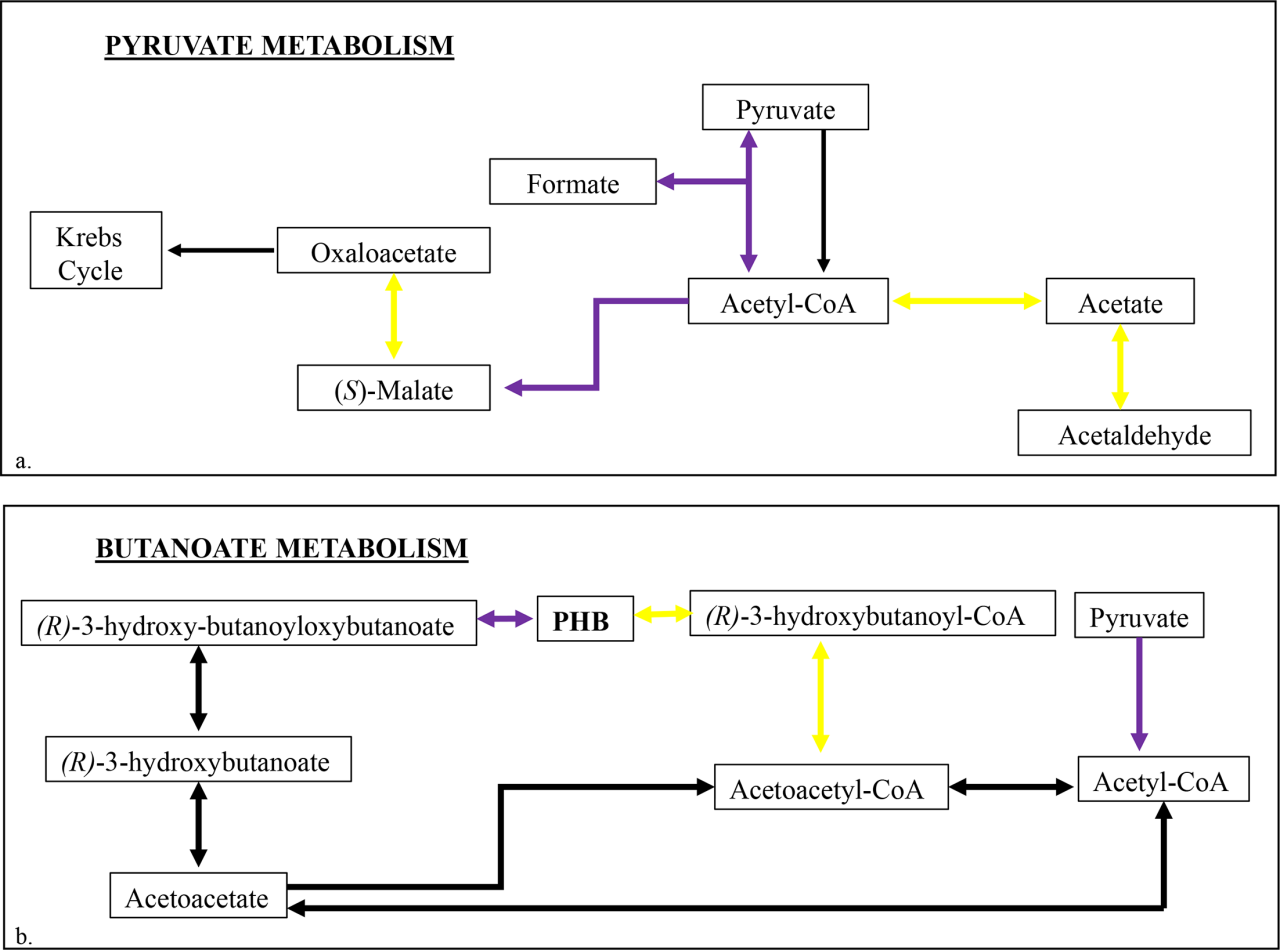
Maps of transcriptionally altered metabolic pathways in encysted compared with vegetative *A. brasilense*. Maps were redrawn based on the KEGG metabolic pathway to highlight metabolic pathways significantly regulated in cysts. Thick purple arrows indicate processes transcriptionally upregulated in a cysts vs. vegetative cells and thick yellow arrows indicate processes transcriptionally downregulated. Thin black arrows denote processes not significantly different between cysts and vegetative cells. (a) Pyruvate metabolism. (b) Butanoate metabolism, focusing on poly-hydroxybutanoate (PHB) synthesis.

Given that cysts are generally acknowledged to be dormant, we were surprised that genes involved in carbon metabolism did not exhibit an extreme repression of expression (Data S1). A lack of general metabolic repression has also been noted by Dong *et al*. [19] regarding the *R. centenum* cyst transcriptome. *Azospirillum* cysts did, however, display different global patterns for several metabolic processes as compared with vegetative cells. Pyruvate metabolism showed distinct changes as genes involved in the enzymatic conversion of acetate and acetaldehyde to acetyl-CoA (AMK58_06725, AMK58_03455 and AMK58_02065) were differentially expressed (Fig. 6). Expression of enzymes that synthesize acetyl-CoA via catalysis of pyruvate was also elevated (AMK58_26495) as were the expression of pathways between Acetyl-CoA, malate and fumarate and acetyl-CoA and homocitrate (AMK58_04510, AMK58_05095, AMK58_04575 and AMK58_26495) (Fig. 6a). Collectively these cysts appear to be utilizing the glyoxylate shunt for central metabolism, which bypasses loss of carbon via CO_2_ generation that occurs in the larger citric acid cycle. Since these cells are not actively transporting or fixing carbon the preferential use of the glyoxylate cycle over that of the citric acid cycle makes sense.

Acetyl-CoA is also used in the initial steps of PHB synthesis in butanoate metabolism, which is known to be ramped up during cyst development. PHB is a well-known component of Gram-negative cysts, composing up to 40 % of cyst dry weight [37, 38]. As we stated previously, PHB stores are thought to sustain cysts through starvation periods [28, 29]. Thus, an accumulation of PHB within the cell is consistent with known cyst physiology. In Fig. 6(b) we show that while acetyl-CoA synthesis genes are elevated, genes involved in the conversion of PHB to (S)-3-Hydroxybutanoyl-CoA were transcriptionally repressed (AMK58_06740, AMK58_26800 and AMK58_19430).

We also note that a metabolic gene annotated as an indole pyruvate decarboxylase (AMK58_025580) was repressed in cyst cells. Indole pyruvate decarboxylase functions in the biosynthesis of the plant hormone, indole-3-acetic acid (IAA), as well as in *A. brasilense* tryptophan metabolism. We did not see any consistent repression of additional tryptophan metabolism genes or other genes involved in IAA biosynthesis, but repression of indole pyruvate decarboxylase is notable as IAA is well known to be involved in regulating plant development.

### Cell membrane and wall

**Fig. 7.**
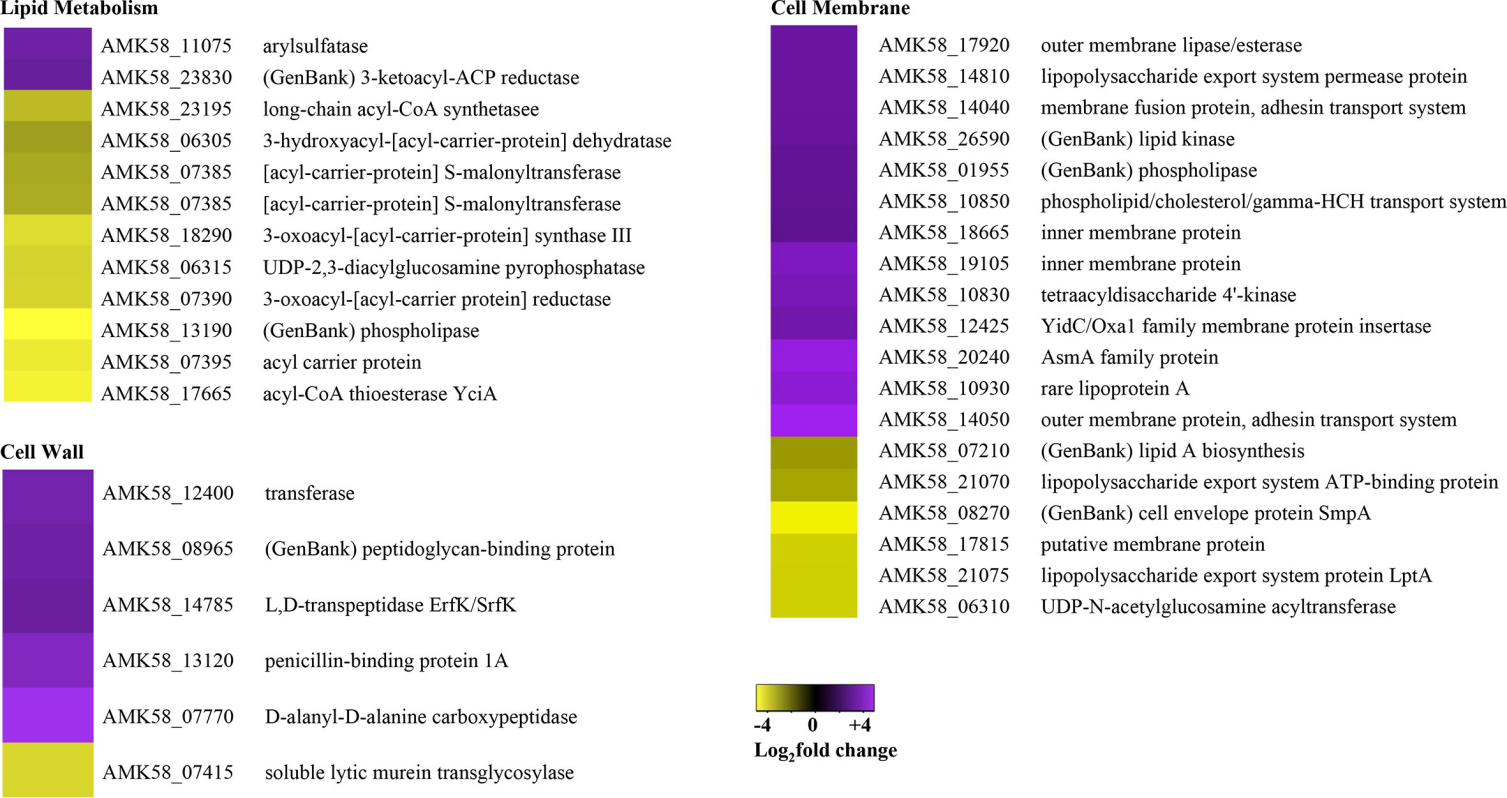
Cell wall and membrane structures are altered in cysts. Heat maps display the DEGs belonging the cell wall, membrane, and lipid metabolism COGs. Purple indicates upregulated and yellow indicates downregulated DEGs in cysts as compared with vegetative cells.

*A. brasilense* vegetative cells are vibriod or S-shaped and relatively small (approximately 1 µm in diameter) whereas *A. brasilense* cysts are circular and much larger, indicating that extensive cell wall changes occur during cyst development. In *Azotobacter,* the exine layer of cysts is composed of alkyl lipids that are distinct from the phospholipids present in vegetative cells [5, 8, 39, 40]. Likewise, *Azospirillum* cysts also have a distinct exine lipopolysaccharide (LPS) layer [41–43]. Alteration in lipid and LPS compositions is thought to be a critical element that provides cysts with their extensive desiccation resistance [41–43]. Given these known alterations in cell wall biochemistry, it is not surprising that multiple genes involved in cell wall and membrane structure were differentially expressed between these cell types (Fig. 7).

Peptidoglycan biosynthesis genes were generally elevated in cysts, indicating modification of the cell wall. Genes specific to LPS biosynthesis (AMK58_06310, AMK58_07210, AMK58_10830 and AMK58_10930) and LPS export (AMK58_21075, AMK58_21070, AMK58_10850 and AMK58_14810) were both repressed and elevated, confirming that cyst cells display significantly different outer membrane LPS composition than their vegetative counterparts. Three genes involved in lipid modification of the outer membrane were elevated (AMK58_01955, AMK58_26590 and AMK58_17920), supporting the idea that LPS is different between cysts and vegetative cells. Furthermore, of the 12 differentially expressed lipid metabolism genes, 10 were repressed (Fig. 7). Notably, genes involved in the transformation of acetyl-CoA to acetyl-[acyl-carrier protein] and malonyl-[acyl-carrier protein] (AMK58_18290, AMK58_07390 and AMK58_07385), were transcriptionally repressed. These genes code for products feeding into fatty acid biosynthesis. Interestingly, a gene encoding a rare lipoprotein A (AMK58_10930) was transcriptionally elevated in cysts. As little is known about this family of proteins, its function in cysts will need to be further researched. Finally, we note that the transport of adhesins is elevated in cysts (AMK58_14050 and AMK58_14040). This coincides with numerous observations that the production of cysts involves the formation of ‘flocculant’ cell aggregates during development [4, 9], in which adhesins play a role.

### Transport and homeostasis

The transport COG makes up a large percentage of differentially regulated genes (Figs 1 and S6). By the nature of this COG, most of these genes are intertwined with the functions of other COGs. For example, we note an elevation of transcripts encoding NiT/TauT transporters that import nitrogen and sulfur compounds into the cell. This coincides with the aforementioned evidence that cysts are using nitrogen and sulfur for anaerobic energy production. C4-dicarboxylate acid and sugar transporters are involved in carbohydrate metabolism, which itself has a number of genes that are significantly altered in cysts (Data S1).

An unclear aspect of transport involves iron acquisition, as this group of genes are equally repressed and elevated, indicating that cysts either have differential iron needs or utilize different mechanisms of iron uptake than do vegetative cells. For example, several ferric iron-siderophore transporters (AMK58_14115 and AMK58_27900) exhibit increased cyst expression while others show decreased expression (AMK58_27615 and AMK58_12560) [18].

There were also a number of differentially expressed transporters involved in branched-chain amino acid (leucine, isoleucine and valine) transport. These transporters were primarily repressed, which fits with reduced protein synthesis exhibited by cyst cells. A defined quorum-sensing pathway for *A. brasilense* has not yet been identified, but branched-chain amino acids do play a role in quorum sensing in other *Proteobacteria* [44].

A group of 11 transport genes predicted to function in the maintenance of cellular homeostasis were found to have significant differential expression in cysts. Of the nine elevated genes, five are involved in protection from osmotic stress (AMK58_24770, AMK58_16550, AMK58_24775, AMK58_16560 and AMK58_16555). These genes function primarily in glycine betaine/proline transport and thus provide a clue as to how cysts survive osmotic stress in a desiccated environment. Indeed, it has been shown that in salt-stressed environments the uptake of glycine betaine stimulates both growth and nitrogen fixation in *A. brasilense* [45].

### Cell motility

**Fig. 8.**
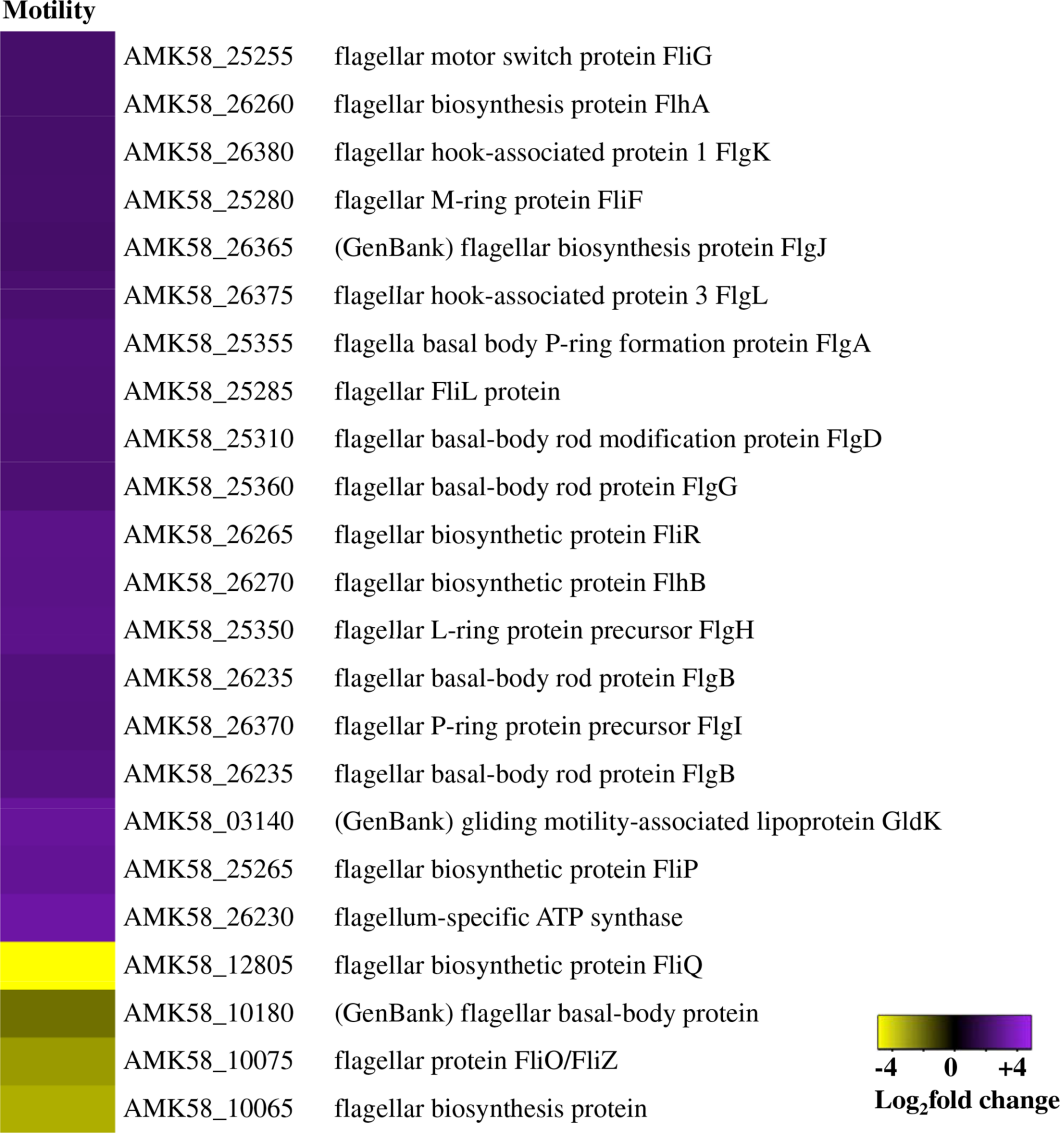
Flagellar biosynthesis genes are upregulated in cysts. Heat maps display the DEGs belonging to the motility COG. Purple indicates upregulated and yellow indicates downregulated DEGs in cysts as compared with vegetative cells.

As cysts are non-motile, repression of cellular motility genes in cysts was expected. Instead, we observed 23 differentially expressed motility genes, 19 of which were elevated (Fig. 8). This curious increase in motility genes has also been seen in *R. centenum* cyst transcriptome analysis [19]. We propose that, since the results of microscopic analysis indicated that cysts are non-motile, post-translational repression of flagella assembly most probably occurs in cysts.

### Congruence with the *Rhodospirillum centenum* transcriptome

*R. centenum* is a photosynthetic member of the *Azospirillum* clade that has been extensively used as a model organism to study Gram-negative cyst development [11, 15, 17–19, 46–48]. *R. centenum* cysts are functionally similar to *A. brasilense* cysts but also exhibit several differences. For example, *R. centenum* develops cysts that are larger and also grouped into multiple germinating bodies relative to *A. brasilense* cysts that are smaller and typically singular [49]. Furthermore, *R. centenum* cysts have a lower heat tolerance than do *A. brasilense* cysts [4]. To address whether there are any similarities in cyst development between Gram-negative species, we compared a previously generated transcriptome profile of *R. centenum* cysts [19] with the results of this *A. brasilense* study. This comparative analysis revealed that of the 1011 genes found by our study to be significantly differentially expressed in the *A. brasilense* cyst state, only 67 of these were homologous to genes significantly differentially expressed during *R. centenum* cyst development as found by Dong *et al.* [19] (Table S2). Of those 68 genes, 29 were either consistently repressed or consistently elevated. The lack of extensive coherent genetic pathways expressed in both *R. centenum* and *A. brasilense* cyst development is in itself informative and indicates several possibilities. One explanation is that the *R. centenum* and *A. brasilense* cysts were generated under significantly different conditions. Specifically, *R. centenum* cysts were made by shifting vegetative cells grown in a rich liquid culture to minimal liquid culture containing butyrate as the sole carbon source. In contrast, *A. brasilense* cysts in this study were obtained by shifting *A. brasilense* from a rich medium to a cyst-inducing minimal media, which utilized 8 mM fructose as a carbon source to induce encystment, and then desiccated to eliminate vegetative cells. This difference in the method of cyst induction may itself be partially responsible for the low number of congruent differentially expressed genes between these studies. Another possibility is that *R. centenum* and *A. brasilense* may use completely different genetic pathways to construct and maintain cysts.

Of 29 DEGs conserved in both *R. centenum* and *A. brasilense* cysts, a few genes do bear further investigation. For example, there were a number of transcription factors and hypothetical proteins (AMK58_03365, AMK58_12240, AMK58_20425, AMK58_03340 and AMK58_04205) that are regulated in similar manner in both species. Further, we observed the elevation of a phospholipase (AMK58_01955) and a rare lipoprotein A gene (AMK58_10930) in both species, which may help elucidate some critical membrane features that are required by cysts to promote long-term survival under harsh conditions.

## Discussion

Our transcriptome analysis of the vegetative and cyst states of *A. brasilense* revealed new processes involved in cyst maintenance while still reflecting on long-known aspects of cyst physiology. We showed that *A. brasilense* cysts are transcriptionally repressed for cell division, ribosome biogenesis, translation and energy production depending on the oxidative respiratory electron transport chain. The repression of these essential cellular functions is expected of a dormant bacterial morphotype. We further noted numerous alterations of lipid metabolism, lipid transport and outer membrane LPS export and modification. Cysts are characterized by the development of an extensive exine layer with altered LPS. Investigation of the LPS-related genes identified in this study may help characterize the composition and function of this exine layer. Finally, we presented transcriptomic evidence for the accumulation of PHB within the cell, which highlights that both PHB synthesis pathways and the accumulation of internal reducing power required for PHB synthesis are elevated. Multiple studies have demonstrated the importance of PHB anabolism and catabolism for cellular survival during starvation periods [28, 29, 37, 50, 51]. We further speculate that the building up of internal reducing power may be required for cyst germination as the energy requirements to initiate vegetative growth are likely to be vast.

Even though our cysts were grown aerobically, this work does show that cysts probably sustain an anaerobic or perhaps micro-aerobic internal environment. This was revealed by the reduced expression of oxidative phosphorylation genes, the presence of alternative nitrogen and sulfur-based electron acceptors and the elevation of nitrogen fixation genes. At first glance, nitrogen fixation in cysts seems counter-intuitive as nitrogen fixation is an energy-intensive process. However, this is not the first evidence of nitrogen fixation occurring in *Azospirillum* cysts. Ueckert *et al*. [52] reported that nitrogenase activity is increased in *Azospirillum* cysts, called ‘c-forms’ in older literature. It has also been reported that cysts produce pigments that protect nitrogenase from oxidative damage [53]. A hyper-nitrogen-fixing *Azospirillum* isolated from sugarcane has also been found to have an enhanced capacity to form cysts at aerobic interfaces [54].

Similar differential gene expression patterns between vegetative and cyst cells have also been observed in *R. centenum* regarding many global cellular processes [18]. These include, but are not limited to, depletion of ribosomes, repression of translation, repression of cell division, global alterations to lipid metabolism and cell membrane biogenesis and the continued transcription of flagella genes. A recent proteomic analysis between vegetative and cyst forms of *Azotobacter vinelandii* also revealed similar changes in nitrogen fixation, flagella synthesis cell division, cyst use of the glyoxylate shunt and cyst use of phenolic lipids [55]. Yet despite the striking similarity in changes in global metabolism upon cyst induction we found little homology between genes that are significantly differentially regulated between vegetative and cyst forms in *R. centenum* and *A. brasilense*. This may be due to the different methods used to induce the *R. centenum* and *A. brasilense* cysts in each study. Alternatively, these two species may simply have diverged to a degree where they now use very different pathways to develop desiccation-resistant cysts.

We recently demonstrated that *A. brasilense* is surprisingly broadly disseminated throughout plant tissue - well beyond plant roots [56]. Even more surprising is that *A. brasilense* is a common inhabitant of plant seeds and that *A. brasilense* appears to selectively migrate from roots to developing seeds [56]. Indeed, viable species of the genus *Azospirillum* can be cultivated from dry seeds that have undergone extensive storage under desiccating conditions, indicating that *A. brasilense* has a mechanism for long-term survival outside of the plant–soil rhizosphere. Presumably *Azospirillum* that inhabits seeds is in cyst state, although this possibility needs to be established. It has, however, been shown that cyst-like *Azospirillum* is often found on plant roots – particularly stressed plants, where secretion of ammonia may be conductive to establishing a productive microbe–plant interaction [57, 58]. Thus, the cyst stage may be an important component in the ability of this bacterium to form a productive interaction with plants.

Finally, while the use of *Azospirillum* as a bio-fertilizer has good greenhouse success, field responses have been quite variable, which has impeded large-scale adoption of *Azospirillum* as an effective biofertilizer [59, 60]. One explanation is that vegetative *Azospirillum* does not have a long enough shelf-life needed for effective use as a commercial bio-fertilizer, which often involves extended shipping and storage of inoculated seeds. Consequently, the use of cysts as a seed inoculant may provide an attractive solution, as this form has much more robust survival in response to environmental changes [22, 23]. The one caveat is that it has been difficult to entice vegetative *A. brasilense* cultures to reproducibly produce cysts at yields high enough for their use as a seed inoculum. To solve these issues will require much more detailed understanding of genetic mechanisms used by *Azospirillum* to regulate cyst induction as well as a better understanding of overall cyst physiology. This study provides a foundation from which to better understand the numerous physiological differences that exist between vegetative and cyst states.

## Data bibliography

1. Sequence data has been deposited in NCBI’s Gene Expression Omnibus through GEO Series number GSE104188.

## Supplementary Data

Supplementary File 1
