# Supplementary material for: Transcriptome analysis of Azospirillum brasilense vegetative and cyst states reveals large-scale alterations in metabolic and replicative gene expression
Source: Microb Genom. 2018 Jul 30;4(8):e000200. doi: 10.1099/mgen.0.000200 (PMC6159551; doi:10.1099/mgen.0.000200)
Supplement: Supplementary File 1 [file mgen-5-200-s001.pdf]

Supplemental Table S1. PCR primers used for RT-qPCR validation of RNASeq.

Gene	Forward Primer 5' – 3'	Reverse Primer 5' – 3'
CstA (AMK58_ 22895)	CGC TCA CCG ACT TCA TCC AT	GTG ATC GAG ATG AAC GGC CA
OxyR (AMK58_ 19140)	GGC GG CCT CGT CAG G	GGG CCA TGT CCA CCA GTT C
ExbD (AMK58_22845)	CAT CCA GGC GGA CAA GAC	GCG TTC ATC ACC TCG GTC
ExoP (AMK58_ 20520)	GGA TCA ACG ACA CCA TCG	GCC TTG AAA GAG ACG AGG
PhaZ (AMK58_13180)	TGC TTC TGG AAC ACC GTC TC	TCT CCG GCT TCA TGT CGA TG
RpoH (AMK58_ 18070)	GGA CCT TCT GGC CGA TGA G	CGA GAC GTG GAA GTG CTG G
TonB (AMK58_ 22840)	CGG TAG TCG CCG TTG ATC TC	GTC ATC GCT CTA CGG CTC C
Hsp15 (AMK58_ 11850)	CC TAC GAG GAT CTT GCC CC	TCC TCT CCG TAG AGC TGG TC
Bfr1 (AMK58_ 88550)	GAT CGT TGC CGA GCA TTT CC	CCT GGA AGG TCT TCC CAA
Amidase (AMK58_ 31750)	CTG CGT GAG ATC GTC ATG GG	GAT CTG GTT GAC GAG TGG GG
Dps (AMK58_ 33250)	CAT GAC CCA GTA CAC GGA GC	CTT GGT GAA CTG CGA GAA GC
*recA** (AMK58_ 10020)	ACGC TAG AGA TCG CCG ATA CG	CCC ATC TCG CCT TCC AGT TC
*16S** (Lin et al 2012)	ATT GAG TTG GGC ACT CTG GT	TCC GCG ATT ACT AGC GAT TC
*gryA** (AMK58_ 16980)	ACG GCA GCG ATC CAA AGC	GAT CGT ATC ACC GGG CGT GT

** denotes housekeeping genes used for normalization.*

Supplemental Table S2. Homologous DEG’s in both *R. centenum* and *A. brasilense.*

Gene ID		COG	log2fold change (cyst / vegetative)				
			R. centenum				*A. brasilense*
A. brasilense sp7	*R. centenum*		24 hrs	48 hrs	72 hrs	96 hrs	
AMK58_12430	2284	Cell Cycle/Shape/Homeostasis	-	1.534	1.944	2.634	3.135
AMK58_13120	2700	Cell Wall	-	-	-	-2.563	3.849
AMK58_17940	dnaJ	Chaperone	-	-	-2.029	-1.824	3.742
AMK58_04635	327	Chemotaxis	-	-	-1.522	-	3.423
AMK58_05485	2156	Defense	-	-	-	1.944	-3.446
AMK58_16895	3229	Defense	-	-	-	1.890	-4.750
AMK58_19425	1946	dehydrogenase	1.585	1.991	2.391	3.410	-5.369
AMK58_14155	2649	dehydrogenase	-	-	-	1.842	-3.597
AMK58_04120	2649	Dehydrogenase	-	-	-	1.842	4.135
AMK58_06095	2649	DNA Repair and Replication	-	-	-	1.842	-5.740
AMK58_21855	2393	DNA Repair and Replication	-	-	-	-2.306	-5.074
AMK58_08205	2178	Energy	-1.735	-1.570	-1.624	-3.443	3.038
AMK58_19150	3864	Energy	-	-1.548	-1.540	-1.562	-5.596
AMK58_17980	1936	Energy	-	2.118	2.237	-	4.109
AMK58_17220	497	Hypothetical	1.973	2.115	2.612	5.529	-4.941
AMK58_08345	965	Hypothetical	2.524	2.619	2.843	4.634	-3.877
AMK58_11590	1988	Hypothetical	-	-	-	-1.637	3.097
AMK58_12240	2202	Hypothetical	-	-	-	-1.633	-4.112
AMK58_03345	2828	Hypothetical	-	-	-	-2.048	4.346
AMK58_04205	1598	Hypothetical	-	-	1.626	2.455	3.199
AMK58_03340	2019	Hypothetical	-	-	-	1.698	3.006

AMK58_13280	2719	Hypothetical	-	-	-	1.597	-4.189
AMK58_07275	0845/2585	Hypothetical	-	-	-	2.332	-6.124
AMK58_10930	4087	Membrane	-	-	1.506	1.803	4.357
AMK58_01955	796	Membrane	-	-	-	2.517	3.089
AMK58_04130	119	Metabolism: Carbohydrate	-	-	-	-	4.844
AMK58_19430	1947	Metabolism: Carbon	-	1.904	2.316	3.023	-3.260
AMK58_02065	336	Metabolism: Carbon	-	-	1.883	2.054	-5.788
AMK58_02575	4080/3026	Metabolism: Carbon	-	-1.684	-2.816	-4.463	-5.622
AMK58_26800	3949	Metabolism: Carbon	-	1.821	2.286	2.934	-5.935
AMK58_27345	1906	Metabolism: Carbon	-	-	-	-1.771	-3.163
AMK58_21280	2991	Metabolism: Co-Factors and Vitamins	-	-2.119	-2.658	-1.661	4.090
AMK58_06940	3970	Metabolism: Co-Factors and Vitamins	-	1.924	2.157	3.978	3.136
AMK58_17665	1370	Metabolism: Lipid	-	-	-	-1.882	-5.129
AMK58_13190	796	Metabolism: Lipid	-	-	-	2.517	-5.356
AMK58_05965	903	Methyltransferase	-	-	-	1.532	3.164
AMK58_25280	0184/0186	Motility	-	-	1.644	-	3.261
AMK58_14130	330	peptidase	-	-	2.506	2.845	4.541
AMK58_10430	700	Ribosome	-	1.614	1.667	-	-6.959
AMK58_12215	2210	Ribosome	-	1.518	-	-	4.101
AMK58_07380	1573	Ribosome	-	1.692	-	-	-3.464
AMK58_10405	707	Ribosome	-	2.111	-	-	-4.970
AMK58_10410	706	Ribosome	-	1.645	-	-	-6.258
AMK58_13080	3432	signal transduction	-	-	-	-2.831	6.417
AMK58_01025	3261	Signal Transduction	-	-	-	-1.783	3.295
AMK58_14140	574	Starvation	-	-1.541	-1.950	-2.833	-5.181
AMK58_03325	1876	Starvation	-	-	-	3.829	-3.644

AMK58_20425	1648	Transcription	-	-	-	-1.611	-3.214
AMK58_01155	2558	Transcription	-	-	-	1.659	-3.441
AMK58_03365	2015	Transcription	-	-2.502	-3.305	-	-4.633
AMK58_20510	2123	Translation	-	-	-	-1.589	-3.262
AMK58_10395	693	translation	-	1.673	-	-	-6.407
AMK58_10465	693	translation	-	1.673	-	-	-5.373
AMK58_25005	24	Transport	2.255	3.533	2.236	2.357	4.863
AMK58_20460	3351	Transport	-	-	-	-1.862	5.313
AMK58_20470	3352	Transport: Amino Acid	-	-	-	-1.862	3.072
AMK58_09260	1802	Transport: Biopolymer	-	-1.771	-2.657	-3.795	-6.468
AMK58_27900	313	Transport: Metal	1.730	1.664	3.370	4.124	4.897
AMK58_14060	3732	Transport: Sugar	2.122	1.965	1.944	3.372	3.332
AMK58_10470	692	tRNA synthesis/modification	1.803	2.702	2.071	-	-3.892
AMK58_16325	406	tRNA synthesis/modification	-	2.455	-	-	3.086
AMK58_10475	691	tRNA synthesis/modification	-	2.189	-	-	-5.623
AMK58_14400	1241	tRNA synthesis/modification	-	2.810	-	-	3.465
AMK58_06050	1256	tRNA synthesis/modification	-	2.739	-	-	-4.504
AMK58_06055	1256	tRNA synthesis/modification	-	2.739	-	-	-3.660
AMK58_02175	2497	tRNA synthesis/modification	-	1.812	-	-	-5.059
AMK58_12220	2209	tRNA synthesis/modification	-	2.059	-	-	3.945

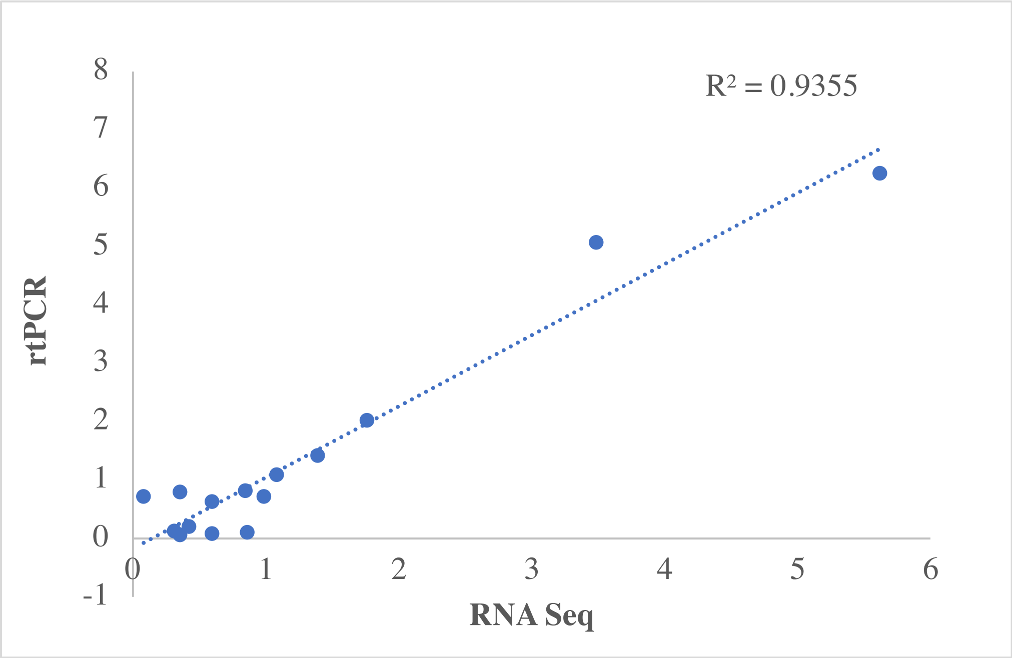

Figure S1. Validation of RNA-seq with rt-PCR. 12 genes were chosen to validate the RNA-seq data. Quantitative reverse transcription PCR (RT-qPCR) on sub-samples of the sequenced RNA was done with the primers in Supplemental Table S1. Log-fold changes of cyst over vegetative RNA obtained from RT-qPCR and RNA-seq analysis were plotted against each for correlation analysis.

Transcription

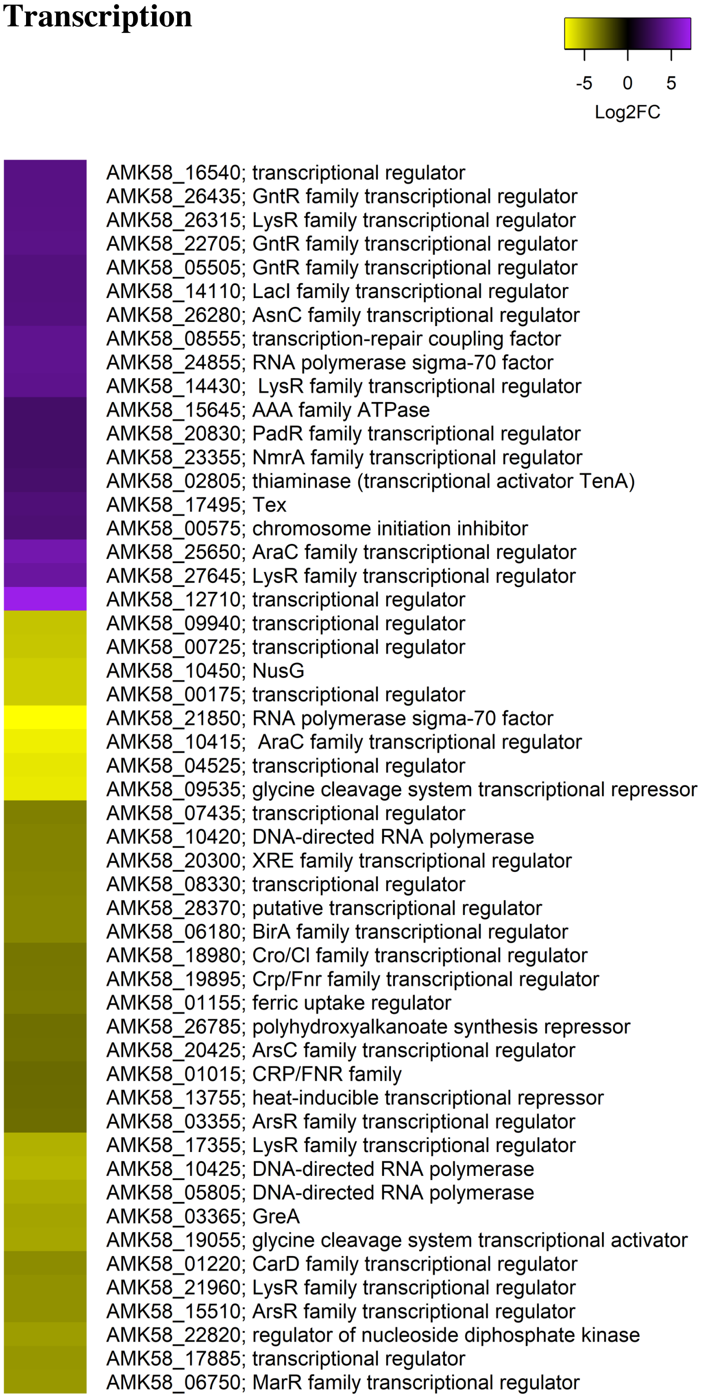

Figure S2. Differentially regulated transcription factors. Heat maps display the DEGs belonging the Transcription COG. Purple indicates genes that are up-regulated and yellow indicates genes that are down-regulated in cyst cells relative to vegetative expression levels.

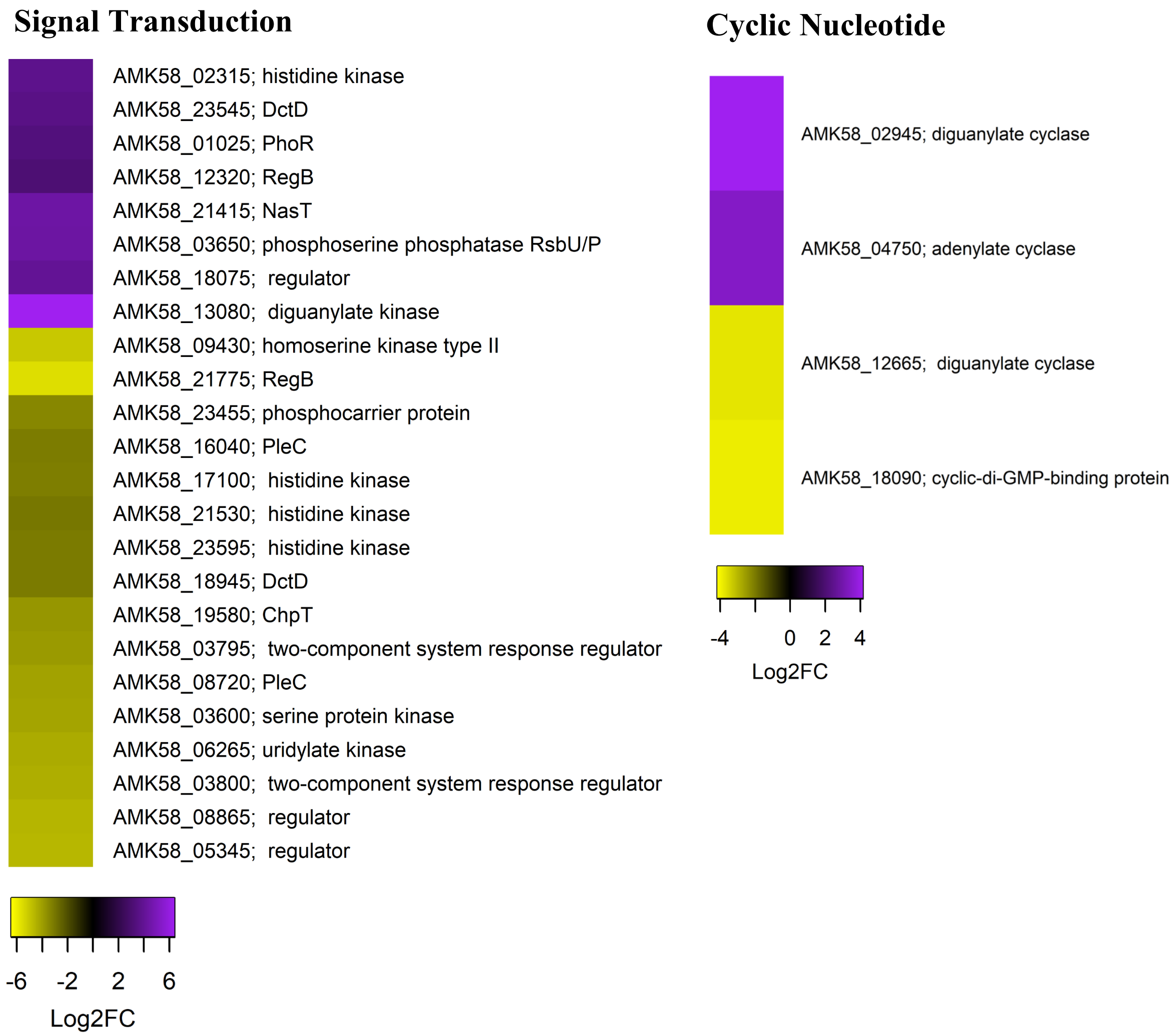

Figure S3. Differentially regulated signal transduction and cyclic nucleotide activity in cysts. Heat maps display the DEGs belonging to the signal transduction COG. Purple indicates up-regulated genes and yellow indicates down-regulated genes in cyst cells relative to vegetative expression levels..

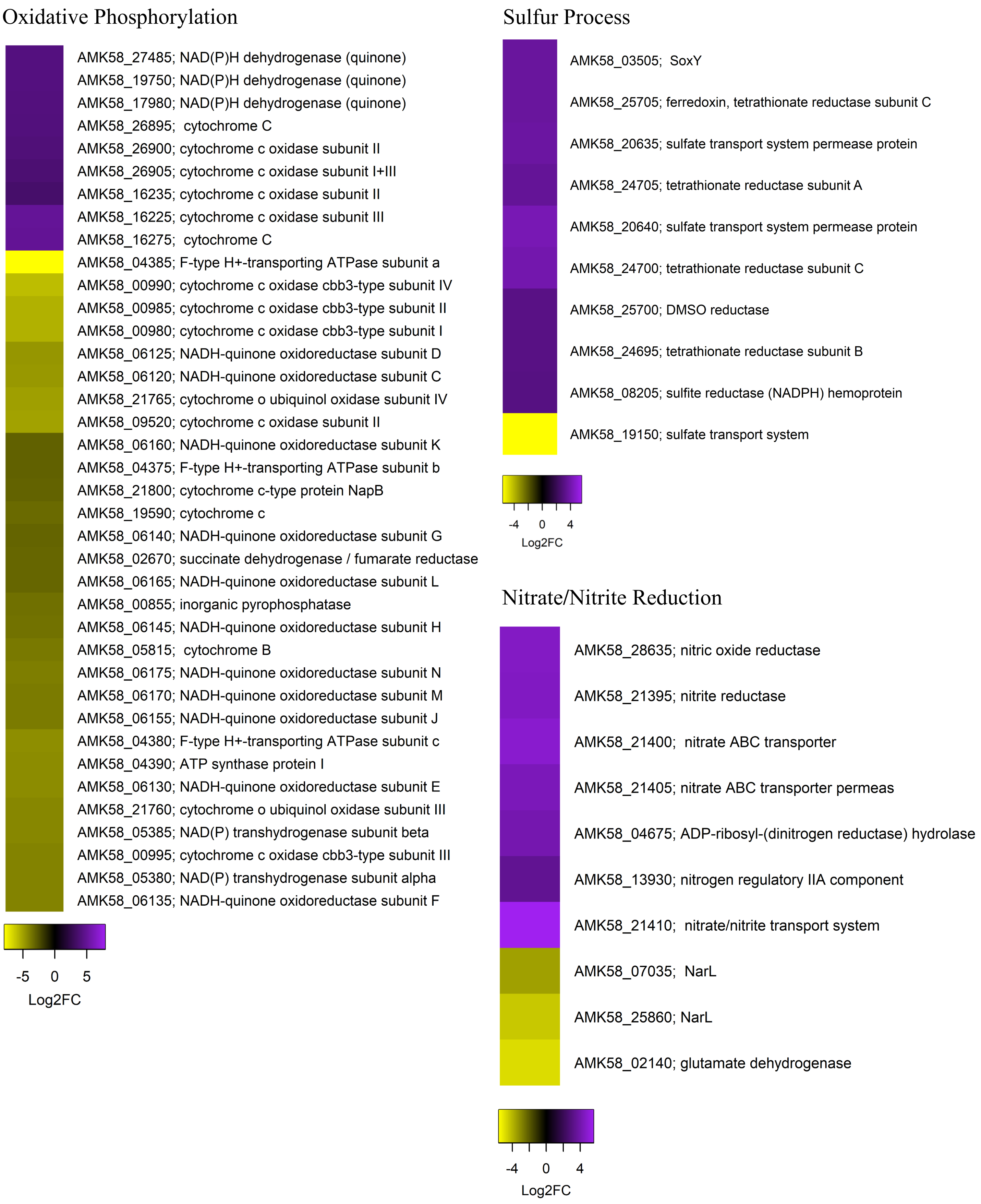

Figure S4. Differentially expressed energy genes in cyst vs vegetative cell show down-regulation of oxidative phosphorylation and up-regulation of alternate electron acceptors. Heat maps display significantly differentially expressed genes from the Energy COG. Purple indicates gene expression up-regulation in cysts compared to vegetative cells and yellow indicates down-regulated gene expression.

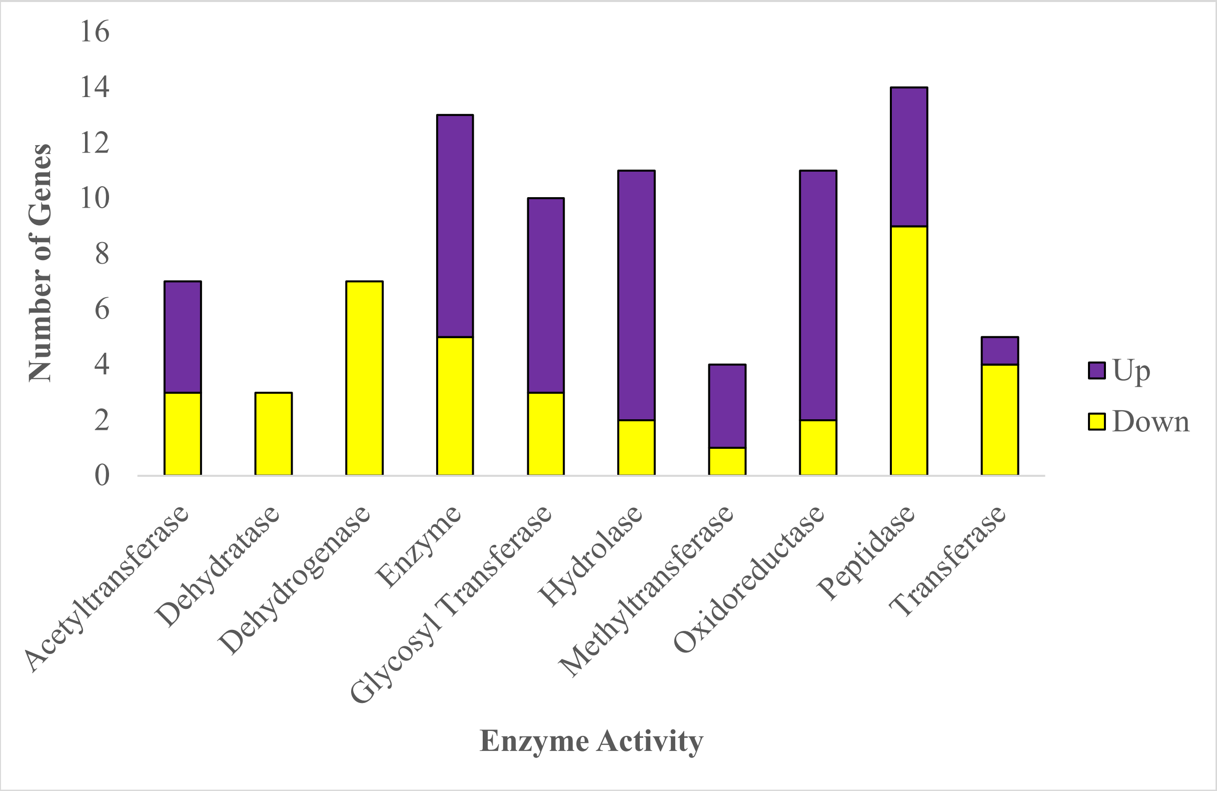

Figure S5. Global enzymatic patterns of cysts. DEGs belonging to the Enzyme COG were broken down in categories based on general enzymatic function. Purple indicates the number up-regulated DEGs and yellow indicates down-regulated DEGs.

A.

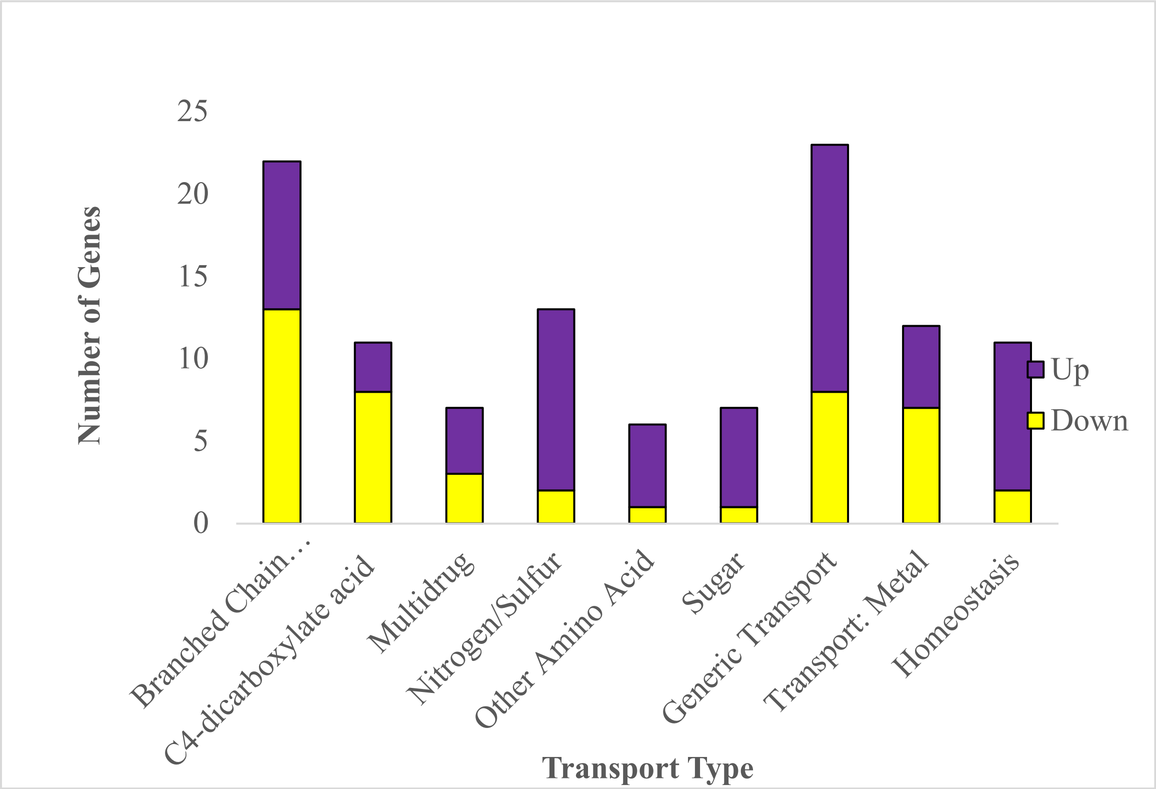

Number of Genes
25
20
15
10
5
0
Up
Down
Branched Chain…
C4-dicarboxylate acid
Multidrug
Nitrogen/Sulfur
Other Amino Acid
Sugar
Generic Transport
Transport: Metal
Homeostasis
Transport Type

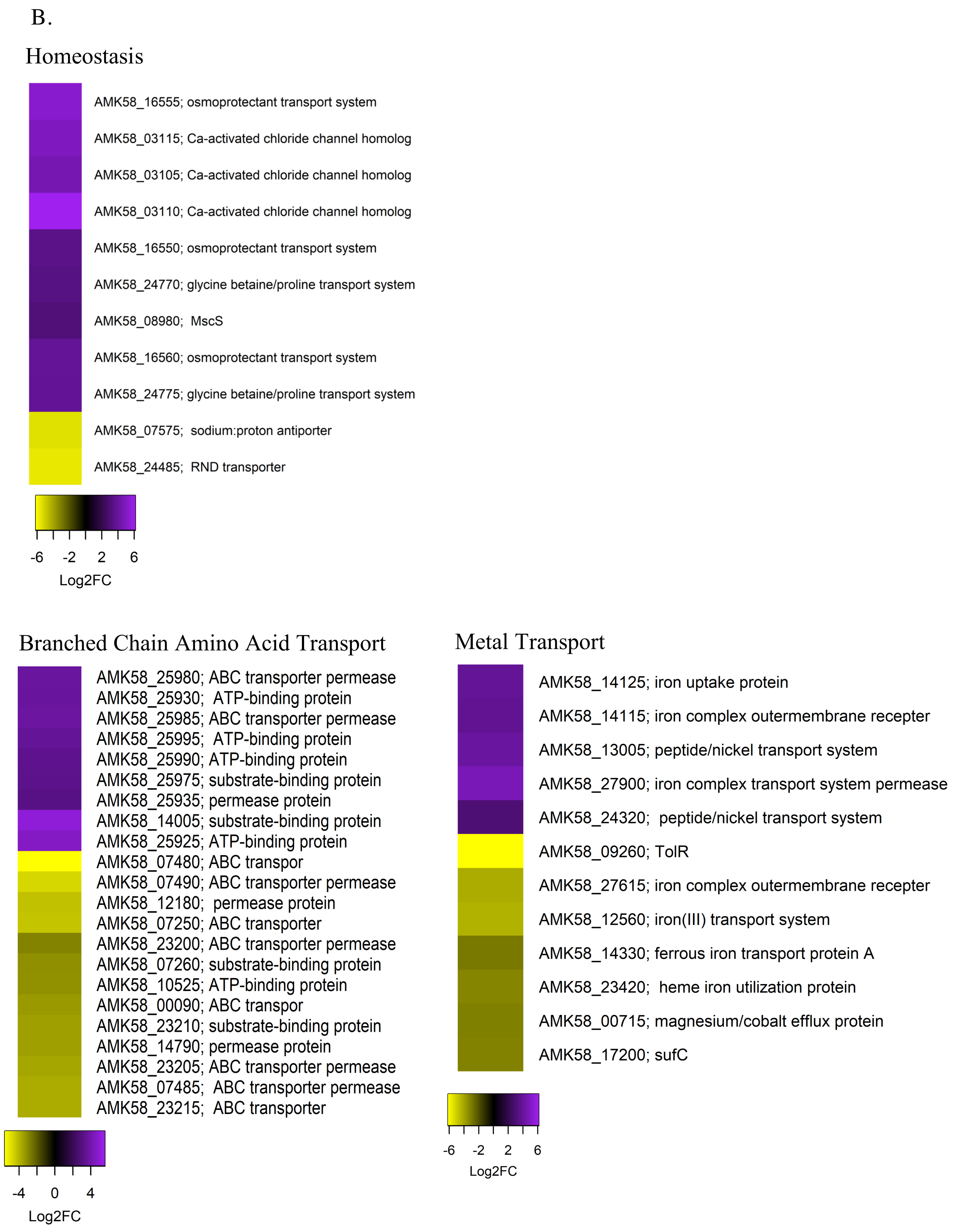

Figure S6. Global transport activity of cysts. (A) DEGs from the Transport COG were grouped based on the predicted substance transported. The number of genes up (purple) or down (yellow) regulated in cysts compared to vegetative cells in each group was calculated to assess overall enzyme patterns. (B) Heat-maps of branched-chain amino acid, metal transport, and homeostasis transport. Purple indicates genes up-regulated in cysts, yellow indicates genes down-regulated in cysts.

Supplemental Data File 1. This is a list of reads per kilobase transcript, per million reads (RPKM) for each annotated *A. brasilense* gene in each replicate vegetative and cyst sample.

Gene	KEGG ID	COG	Vegetative_R1	Vegetative_R2	Vegetative_R3	Cyst_R1	Cyst_R2	Cyst_R3
AMK58_00005	no KO assigned | (GenBank) hypothetical protein	Hypothetical	0.000	0.000	0.000	0.000	0.000	0.000
AMK58_00010	no KO assigned | (GenBank) pseudogene	Pseudogene	0.000	0.000	0.121	0.000	0.000	0.000
AMK58_00015	no KO assigned | (GenBank) pseudogene	Pseudogene	0.000	0.000	0.000	0.000	0.000	0.000
AMK58_00020	no KO assigned | (GenBank) pseudogene	Pseudogene	0.000	0.000	0.000	0.000	0.000	0.000
AMK58_00025	no KO assigned | (GenBank) pseudogene	Pseudogene	0.000	0.000	0.000	0.000	0.000	0.000
AMK58_00030	no KO assigned | (GenBank) ABC transporter substrate-binding protein	Transport	0.000	0.000	0.000	0.000	0.000	0.000
AMK58_00035	no KO assigned | (GenBank) pseudogene	Pseudogene	0.000	0.000	0.000	0.000	0.000	0.000
AMK58_00040	K10764 O-succinylhomoserine sulfhydrylase [EC:2.5.1.-] | (GenBank) O-succinylhomoserine sulfhydrylas	Metabolism	0.000	0.000	0.000	0.000	0.000	0.000
AMK58_00045	no KO assigned | (GenBank) hypothetical protein	Hypothetical	0.000	0.000	0.000	0.000	0.000	5.866
AMK58_00050	no KO assigned | (GenBank) hypothetical protein	Hypothetical	91.302	67.197	99.392	278.286	161.378	280.981
AMK58_00055	K03555 DNA mismatch repair protein MutS | (GenBank) DNA mismatch repair protein MutS	DNA Repair and Replication	16.009	20.361	16.287	16.639	15.121	8.483
AMK58_00060	no KO assigned | (GenBank) hypothetical protein	Hypothetical	15.557	22.900	15.221	67.955	38.522	39.305
AMK58_00065	no KO assigned | (GenBank) hypothetical protein	Hypothetical	1029.324	1149.256	1084.844	1244.941	1016.671	1595.558
AMK58_00070	K00990 [protein-PII] uridylyltransferase [EC:2.7.7.59] | (GenBank) protein-PII uridylyltransferase	Signal Transduction	3.111	4.131	6.081	52.036	22.488	16.628
AMK58_00075	K19302 undecaprenyl-diphosphatase [EC:3.6.1.27] | (GenBank) hypothetical protein	Hypothetical	156.414	152.417	162.041	276.752	230.818	291.824
AMK58_00080	no KO assigned | (GenBank) pirin	No COG	19.465	17.057	15.670	108.472	71.912	85.075
AMK58_00085	no KO assigned | (GenBank) hypothetical protein	Hypothetical	2225.861	2031.117	1878.650	475.104	577.051	604.480
AMK58_00090	K01999 branched-chain amino acid transport system substrate-binding protein | (GenBank) ABC transpor	Membrane Transport	119.878	137.782	129.910	492.241	104.913	323.595
AMK58_00095	no KO assigned | (GenBank) histidine kinase	Signal Transduction	4.142	4.604	6.090	136.768	18.351	49.213
AMK58_00100	K03980 putative peptidoglycan lipid II flippase | (GenBank) multidrug transporter MurJ	Cell Wall	169.043	146.049	131.638	74.039	71.805	136.720
AMK58_00105	K01867 tryptophanyl-tRNA synthetase [EC:6.1.1.2] | (GenBank) tryptophan--tRNA ligase	tRNA synthesis/modification	24.603	29.687	24.314	142.866	20.979	69.003
AMK58_00110	K01304 pyroglutamyl-peptidase [EC:3.4.19.3] | (GenBank) pyrrolidone-carboxylate peptidase	Peptidase	15.477	26.921	13.784	216.248	70.909	193.474
AMK58_00115	no KO assigned | (GenBank) hypothetical protein	Hypothetical	11.440	9.493	13.899	163.463	46.257	98.125
AMK58_00120	K09858 SEC-C motif domain protein | (GenBank) hypothetical protein	Hypothetical	169.505	147.685	193.784	150.698	89.028	219.861
AMK58_00125	no KO assigned | (GenBank) MarR family transcriptional regulator	Transcription	133.122	133.155	188.429	133.541	56.536	203.873
AMK58_00130	no KO assigned | (GenBank) hypothetical protein	Hypothetical	67.465	82.772	90.492	480.909	423.175	578.105
AMK58_00135	K00638 chloramphenicol O-acetyltransferase type B [EC:2.3.1.28] | (GenBank) chloramphenicol acetyltr	Defense	8.567	13.648	14.661	126.485	111.699	61.431
AMK58_00140	no KO assigned | (GenBank) TetR family transcriptional regulator	Transcription	2.143	2.134	4.075	190.413	65.036	161.119
AMK58_00145	no KO assigned | (GenBank) oxidoreductase	Oxidoreductase	16.830	20.500	27.653	530.332	1322.179	213.176
AMK58_00150	no KO assigned | (GenBank) hypothetical protein	Hypothetical	16.766	19.793	19.855	151.427	113.750	53.808
AMK58_00155	no KO assigned | (GenBank) transporter	Transport	24.118	20.861	24.012	128.030	120.120	92.918
AMK58_00160	K00797 spermidine synthase [EC:2.5.1.16] | (GenBank) spermidine synthase	Metabolism: Amino Acid	101.950	125.180	95.331	52.932	288.941	104.576
AMK58_00165	K01611 S-adenosylmethionine decarboxylase [EC:4.1.1.50] | (GenBank) S-adenosylmethionine decarboxy	Metabolism: Amino Acid	217.206	262.886	282.468	465.158	246.039	186.596
AMK58_00170	K07010 putative glutamine amidotransferase | (GenBank) peptidase C26	Peptidase	1678.840	1877.687	1977.115	60.873	353.985	134.665
AMK58_00175	no KO assigned | (GenBank) transcriptional regulator	Transcription	238.481	289.342	310.705	314.215	55.812	239.735
AMK58_00180	K16165 fumarylpyruvate hydrolase [EC:3.7.1.20] | (GenBank) fumarylacetoacetate hydrolase	Metabolism: Amino Acid	204.446	182.954	169.817	123.536	63.072	168.405
AMK58_00185	K01142 exodeoxyribonuclease III [EC:3.1.11.2] | (GenBank) exodeoxyribonuclease III	DNA Repair and Replication	24.976	17.288	34.029	191.971	25.164	68.915
AMK58_00190	K15634 probable phosphoglycerate mutase [EC:5.4.2.12] | (GenBank) phosphoglycerate mutase	Metabolism: Carbon	18.532	12.302	20.557	295.134	63.880	122.771
AMK58_00195	no KO assigned | (GenBank) hypothetical protein	Hypothetical	97.725	117.252	106.028	145.334	106.680	173.024
AMK58_00200	no KO assigned | (GenBank) hypothetical protein	Hypothetical	18.883	25.070	24.056	191.614	81.711	86.634
AMK58_00205	no KO assigned | (GenBank) glycosyl transferase	Transferase	89.073	104.957	96.248	178.595	199.497	158.851
AMK58_00210	no KO assigned | (GenBank) hypothetical protein	Hypothetical	249.121	280.425	233.599	391.851	227.374	431.050
AMK58_00215	no KO assigned | (GenBank) multidrug transporter	Membrane Transport	21.647	25.756	25.727	29.058	15.053	19.477
AMK58_00220	no KO assigned | (GenBank) cyclic nucleotide-binding protein	cyclic nucleotide	37.703	49.175	52.510	405.465	123.260	157.079
AMK58_00225	no KO assigned | (GenBank) hypothetical protein	Hypothetical	43.809	59.343	54.279	105.463	55.589	64.376
AMK58_00230	no KO assigned | (GenBank) hypothetical protein	Hypothetical	15.227	19.431	17.851	124.560	38.884	56.203
AMK58_00235	no KO assigned | (GenBank) hypothetical protein	Hypothetical	113.679	135.077	150.679	715.826	223.022	369.138
AMK58_00240	K02031 peptide/nickel transport system ATP-binding protein K02032 peptide/nickel transport system AT	Quorum Sensing	63.821	81.896	61.045	197.971	84.996	178.366
AMK58_00245	K02035 peptide/nickel transport system substrate-binding protein | (GenBank) peptide ABC transporter	Quorum Sensing	34.593	33.671	37.264	73.240	48.787	76.168
AMK58_00250	K02033 peptide/nickel transport system permease protein | (GenBank) ABC transporter permease	Quorum Sensing	125.438	136.127	128.947	190.759	141.815	248.140
AMK58_00255	K02034 peptide/nickel transport system permease protein | (GenBank) peptide ABC transporter permease	Quorum Sensing	46.964	56.734	46.421	91.809	26.685	76.474
AMK58_00260	no KO assigned | (GenBank) hypothetical protein	Hypothetical	55.661	67.226	72.181	290.206	78.249	212.436
AMK58_00265	no KO assigned | (GenBank) MBL fold metallo-hydrolase	Hydrolase	32.204	38.810	39.424	128.860	81.563	74.462
AMK58_00270	no KO assigned | (GenBank) hypothetical protein	Hypothetical	Data File 1	17.592	32.938	453.146	662.164	351.104
AMK58_00275	no KO assigned | (GenBank) sorbosone dehydrogenase	Metabolism: Carbohydrate	3.459	2.650	5.819	97.430	198.507	73.859
AMK58_00280	no KO assigned | (GenBank) hypothetical protein	Hypothetical	112.949	127.841	127.481	265.230	99.558	342.335
AMK58_00285	no KO assigned | (GenBank) two-component system response regulator	Signal Transduction	87.911	81.771	90.594	203.320	104.141	172.635
AMK58_00290	K01497 GTP cyclohydrolase II [EC:3.5.4.25] | (GenBank) GTP cyclohydrolase	Metabolism: Co-Factors and Vitamins	52.608	72.532	63.435	226.943	44.504	94.623
AMK58_00295	no KO assigned | (GenBank) hypothetical protein	Hypothetical	14.744	19.409	19.246	79.838	15.028	21.782
AMK58_00300	no KO assigned | (GenBank) hypothetical protein	Hypothetical	55.667	84.530	61.970	465.430	79.779	198.300
AMK58_00305	K06997 PLP dependent protein | (GenBank) alanine racemase	No COG	129.112	124.646	98.697	66.203	104.761	160.793
AMK58_00310	no KO assigned | (GenBank) hypothetical protein	Hypothetical	1294.845	864.744	776.099	257.290	114.242	512.169
AMK58_00315	no KO assigned | (GenBank) hypothetical protein	Hypothetical	27910.875	25033.849	24577.595	149.721	942.075	310.341
AMK58_00320	no KO assigned | (GenBank) hypothetical protein	Hypothetical	188.373	175.135	180.382	53.371	54.878	78.771
AMK58_00325	no KO assigned | (GenBank) hypothetical protein	Hypothetical	1139.448	1039.489	1065.703	852.677	432.222	858.447
AMK58_00330	K01869 leucyl-tRNA synthetase [EC:6.1.1.4] | (GenBank) leucine--tRNA ligase	tRNA synthesis/modification	31.505	28.135	26.821	43.716	15.616	38.471

AMK58_00335	K03643 LPS-assembly lipoprotein | (GenBank) hypothetical protein	Hypothetical	69.530	61.590	70.698	471.867	126.231	251.590
AMK58_00340	K02340 DNA polymerase III subunit delta [EC:2.7.7.7] | (GenBank) DNA polymerase III subunit delta	DNA Repair and Replication	15.741	16.970	21.493	200.711	114.276	287.204
AMK58_00345	no KO assigned | (GenBank) glycerophosphodiester phosphodiesterase	enzyme	105.818	144.141	112.420	105.391	82.952	161.537
AMK58_00350	K03497 chromosome partitioning protein, ParB family | (GenBank) chromosome partitioning protein ParB	Cell Cycle/Shape/Homeostatsis	172.283	203.963	192.368	64.520	133.267	156.196
AMK58_00355	K03496 chromosome partitioning protein | (GenBank) chromosome partitioning protein ParA	Cell Cycle/Shape/Homeostatsis	64.363	71.708	65.477	137.466	60.711	195.324
AMK58_00360	K03501 16S rRNA (guanine527-N7)-methyltransferase [EC:2.1.1.170] | (GenBank) 16S rRNA (guanine(	Ribosome	370.312	450.480	435.580	516.621	353.178	591.287
AMK58_00365	K03495 tRNA uridine 5-carboxymethylaminomethyl modification enzyme | (GenBank) tRNA uridine 5-car	tRNA synthesis/modification	12.697	13.546	16.322	148.302	29.782	88.122
AMK58_00370	K03650 tRNA modification GTPase [EC:3.6.-.-] | (GenBank) tRNA modification GTPase MnmE	tRNA synthesis/modification	35.541	30.411	32.323	63.925	67.025	47.538
AMK58_00375	no KO assigned | (GenBank) ferredoxin	Ferredoxin	150.335	154.718	128.549	91.004	260.089	195.224
AMK58_00380	no KO assigned | (GenBank) ribosomal protein S1	Ribosome	937.369	970.215	839.637	1445.514	1886.943	1385.550
AMK58_00385	K06888 uncharacterized protein | (GenBank) thymidylate kinase	Signal Transduction	15.052	16.122	17.619	67.879	108.187	21.409
AMK58_00390	K00567 methylated-DNA-[protein]-cysteine S-methyltransferase [EC:2.1.1.63] | (GenBank) cysteine meth	DNA Repair and Replication	882.779	914.587	966.823	384.026	844.195	309.494
AMK58_00395	no KO assigned | (GenBank) quinone oxidoreductase	Energy	75.377	77.158	86.782	64.043	127.579	83.022
AMK58_00400	K02342 DNA polymerase III subunit epsilon [EC:2.7.7.7] | (GenBank) DNA polymerase III subunit epsilo	DNA Repair and Replication	11.072	7.468	13.074	105.014	35.328	31.131
AMK58_00405	K00859 dephospho-CoA kinase [EC:2.7.1.24] | (GenBank) dephospho-CoA kinase	Metabolism: Co-Factors and Vitamins	71.894	68.410	68.179	304.004	95.160	182.591
AMK58_00410	K00014 shikimate dehydrogenase [EC:1.1.1.25] | (GenBank) shikimate dehydrogenase	Biosynthesis: Amino Acid	69.388	54.146	68.533	124.817	64.784	93.886
AMK58_00415	K06287 septum formation protein | (GenBank) septum formation protein Maf	No COG	278.532	217.363	231.750	109.722	289.617	257.538
AMK58_00420	K09773 [pyruvate, water dikinase]-phosphate phosphotransferase / [pyruvate, water dikinase] kinase [	Signal Transduction	277.374	272.643	242.441	99.527	117.979	239.218
AMK58_00425	K01599 uroporphyrinogen decarboxylase [EC:4.1.1.37] | (GenBank) uroporphyrinogen decarboxylase	Metabolism: Co-Factors and Vitamins	75.975	64.943	55.525	35.473	35.226	89.215
AMK58_00430	K08973 putative membrane protein | (GenBank) hypothetical protein	Membrane	60.114	50.584	47.497	48.769	63.129	96.829
AMK58_00435	no KO assigned | (GenBank) AbrB family transcriptional regulator	Transcription	112.465	115.737	114.607	302.747	362.310	346.290
AMK58_00440	K07341 death on curing protein | (GenBank) death-on-curing protein	Defense	253.603	239.489	261.870	988.822	1436.641	777.038
AMK58_00445	no KO assigned | (GenBank) carbonate dehydratase	Membrane	164.235	142.835	137.206	51.035	287.071	119.562
AMK58_00450	K03628 transcription termination factor Rho | (GenBank) transcription termination factor Rho	Transcription	221.152	210.044	198.276	61.287	365.730	108.608
AMK58_00455	K03071 preprotein translocase subunit SecB | (GenBank) preprotein translocase subunit SecB	Chaperone	91.900	92.961	85.299	302.877	782.126	234.233
AMK58_00460	K07113 UPF0716 protein FxsA | (GenBank) hypothetical protein	Membrane	563.047	746.879	651.779	261.343	4063.765	243.890
AMK58_00465	no KO assigned | (GenBank) translocase	Membrane	37.130	35.364	43.830	455.562	287.509	225.205
AMK58_00470	K08304 membrane-bound lytic murein transglycosylase A | (GenBank) murein transglycosylase	Cell Wall	8.634	14.395	10.118	118.345	110.703	135.636
AMK58_00475	no KO assigned | (GenBank) hypothetical protein	Hypothetical	168.471	137.292	127.000	65.413	93.150	61.928
AMK58_00480	no KO assigned | (GenBank) hypothetical protein	Hypothetical	188.339	211.714	186.873	139.330	196.579	153.040
AMK58_00485	K01523 phosphoribosyl-ATP pyrophosphohydrolase [EC:3.6.1.31] | (GenBank) phosphoribosyl-ATP pyr	Biosynthesis: Amino Acid	463.039	475.779	444.373	296.375	541.538	425.021
AMK58_00490	K02500 cyclase [EC:4.1.3.-] | (GenBank) imidazole glycerol phosphate synthase subunit HisF	Biosynthesis: Amino Acid	393.416	331.308	297.464	119.473	471.478	209.541
AMK58_00495	K01814 phosphoribosylformimino-5-aminoimidazole carboxamide ribotide isomerase [EC:5.3.1.16] | (Gen	Biosynthesis: Amino Acid	91.637	76.591	65.460	196.743	77.742	112.563
AMK58_00500	no KO assigned | (GenBank) acetyltransferase	Acetyltransferase	216.062	233.126	226.634	281.361	192.235	207.999
AMK58_00505	K02501 glutamine amidotransferase [EC:2.4.2.-] | (GenBank) imidazole glycerol phosphate synthase sub	Biosynthesis: Amino Acid	303.532	296.071	305.199	96.306	271.975	183.635
AMK58_00510	K01693 imidazoleglycerol-phosphate dehydratase [EC:4.2.1.19] | (GenBank) imidazoleglycerol-phosphate	Biosynthesis: Amino Acid	22.896	28.981	37.266	612.096	70.228	440.982
AMK58_00515	no KO assigned | (GenBank) hypothetical protein	Hypothetical	32.504	33.450	32.088	115.416	203.746	58.262
AMK58_00520	K01419 ATP-dependent HslUV protease, peptidase subunit HslV [EC:3.4.25.2] | (GenBank) ATP-depend	enzyme	260.675	238.483	230.238	358.641	1050.570	486.502
AMK58_00525	K03667 ATP-dependent HslUV protease ATP-binding subunit HslU | (GenBank) ATP-dependent proteas	enzyme	32.395	35.681	34.673	189.217	156.945	112.270
AMK58_00530	no KO assigned | (GenBank) ABC transporter	Transport	35.156	36.412	37.949	162.285	72.934	172.850
AMK58_00535	no KO assigned | (GenBank) hypothetical protein	Hypothetical	3.298	1.407	2.538	28.503	7.678	25.046
AMK58_00540	no KO assigned | (GenBank) XRE family transcriptional regulator	Transcription	12.855	21.433	23.024	221.454	18.482	73.460
AMK58_00545	no KO assigned | (GenBank) DNA mismatch repair protein MutS	DNA Repair and Replication	38.496	48.224	57.264	341.838	40.944	169.460
AMK58_00550	K00782 L-lactate dehydrogenase complex protein LldG | (GenBank) L-lactate dehydrogenase	Metabolism: Carbohydrate	133.764	121.086	141.930	413.845	139.659	337.136
AMK58_00555	K18929 L-lactate dehydrogenase complex protein LldF | (GenBank) (Fe-S)-binding protein	Metabolism: Carbohydrate	37.523	31.883	36.153	111.140	34.398	103.687
AMK58_00560	K18928 L-lactate dehydrogenase complex protein LldE | (GenBank) Fe-S oxidoreductase	Metabolism: Carbohydrate	61.154	49.696	64.443	392.052	33.590	355.092
AMK58_00565	no KO assigned | (GenBank) hypothetical protein	Hypothetical	4.824	4.434	5.910	117.694	9.386	68.050
AMK58_00570	K06895 L-lysine exporter family protein LysE/ArgO | (GenBank) amino acid transporter	Transport	5.247	10.850	9.593	342.753	30.848	157.739
AMK58_00575	K05596 LysR family transcriptional regulator, chromosome initiation inhibitor | (GenBank) LysR famil	Transcription	9.440	8.174	12.618	196.822	17.069	99.330
AMK58_00580	K09816 zinc transport system permease protein | (GenBank) hypothetical protein	Transport: Metal	17.494	24.754	24.512	168.147	18.629	132.048
AMK58_00585	K09817 zinc transport system ATP-binding protein [EC:3.6.3.-] | (GenBank) zinc ABC transporter ATP-b	Transport: Metal	8.421	8.544	13.446	330.331	51.258	248.850
AMK58_00590	K09815 zinc transport system substrate-binding protein | (GenBank) zinc transporter	Transport: Metal	33.066	47.322	47.339	144.165	46.778	62.139
AMK58_00595	K00215 4-hydroxy-tetrahydrodipicolinate reductase [EC:1.17.1.8] | (GenBank) 4-hydroxy-tetrahydrodipi	Biosynthesis: Amino Acid	35.807	26.287	44.865	251.002	121.427	140.407
AMK58_00600	K10778 AraC family transcriptional regulator, regulatory protein of adaptative response / methylated	Transcription	55.241	44.859	65.400	137.400	196.981	115.254
AMK58_00605	no KO assigned | (GenBank) hypothetical protein	Hypothetical	138.913	109.236	130.171	152.927	142.108	262.583
AMK58_00610	K10773 endonuclease III [EC:4.2.99.18] | (GenBank) endonuclease III	DNA Repair and Replication	43.908	44.652	43.525	142.914	150.585	84.549
AMK58_00615	no KO assigned | (GenBank) hypothetical protein	Hypothetical	28.280	22.000	25.629	180.733	81.788	230.003
AMK58_00620	no KO assigned | (GenBank) hypothetical protein	Hypothetical	121.075	115.862	103.159	412.135	91.318	237.014
AMK58_00625	no KO assigned | (GenBank) carbohydrate kinase	Signal Transduction	106.774	107.888	106.937	202.773	71.657	128.790
AMK58_00630	K07090 uncharacterized protein | (GenBank) permease	Membrane	29.280	41.762	34.736	167.309	70.567	132.204
AMK58_00635	no KO assigned | (GenBank) hypothetical protein	Hypothetical	96.394	125.196	118.071	96.064	163.486	138.610
AMK58_00640	no KO assigned | (GenBank) universal stress protein	Stress Response	86.419	83.867	109.905	466.578	241.861	236.743
AMK58_00645	no KO assigned | (GenBank) hypothetical protein	Hypothetical	35.159	46.843	41.355	202.097	50.931	122.907
AMK58_00650	K01768 adenylate cyclase [EC:4.6.1.1] | (GenBank) adenylate cyclase	cyclic nucleotide	153.848	140.604	152.105	94.836	449.162	112.270
AMK58_00655	no KO assigned | (GenBank) iron transporter	Transport: Metal	80.935	76.897	93.962	464.933	429.613	184.689
AMK58_00660	no KO assigned | (GenBank) chitin deacetylase	Metabolism: carbohydrate	29.695	37.675	25.618	116.571	88.796	104.402
AMK58_00665	K08641 D-alanyl-D-alanine dipeptidase [EC:3.4.13.22] | (GenBank) D-alanyl-D-alanine dipeptidase	Defense	28.295	31.450	28.780	184.222	70.266	48.813

AMK58_00670	K14742 tRNA threonylcarbamoyladenosine biosynthesis protein TsaB | (GenBank) tRNA threonylcarbamo	tRNA synthesis/modification	33.930	46.943	38.882	98.795	54.799	60.771
AMK58_00675	K03789 ribosomal-protein-alanine N-acetyltransferase [EC:2.3.1.128] | (GenBank) alanine acetyltransf	Ribosome	39.178	49.352	48.247	29.402	41.120	94.981
AMK58_00680	no KO assigned | (GenBank) SirA protein	No COG	726.449	743.212	769.717	323.100	587.602	416.939
AMK58_00685	no KO assigned | (GenBank) MucR family transcriptional regulator	Transcription	329.544	376.422	284.623	367.932	517.937	424.848
AMK58_00690	no KO assigned | (GenBank) hemolysin-like protein	No COG	50.631	42.963	50.373	344.817	181.293	182.217
AMK58_00695	K00655 1-acyl-sn-glycerol-3-phosphate acyltransferase [EC:2.3.1.51] | (GenBank) acyl-phosphate glyce	Metabolism: Lipid	381.371	412.850	467.101	363.071	1352.610	293.732
AMK58_00700	K06168 tRNA-2-methylthio-N6-dimethylallyladenosine synthase [EC:2.8.4.3] | (GenBank) (dimethylallyl)	tRNA synthesis/modification	92.465	108.235	116.910	142.003	175.107	104.619
AMK58_00705	K06217 phosphate starvation-inducible protein PhoH and related proteins | (GenBank) phosphate starva	Starvation	116.453	132.009	133.234	43.491	128.870	85.927
AMK58_00710	K07042 probable rRNA maturation factor | (GenBank) rRNA maturation factor	Ribosome	714.364	843.234	856.129	199.143	536.940	345.266
AMK58_00715	no KO assigned | (GenBank) magnesium/cobalt efflux protein	No COG	46.425	39.398	49.785	299.638	81.389	241.539
AMK58_00720	K03820 apolipoprotein N-acyltransferase [EC:2.3.1.-] | (GenBank) acyltransferase	Biosynthesis: Lipoprotein	750.588	968.114	992.340	32.335	322.087	83.531
AMK58_00725	no KO assigned | (GenBank) transcriptional regulator	Transcription	1435.318	1056.188	1106.696	454.717	596.695	543.670
AMK58_00730	K00789 S-adenosylmethionine synthetase [EC:2.5.1.6] | (GenBank) S-adenosylmethionine synthetase	Biosynthesis: Amino Acid	43.773	27.821	31.682	103.827	52.576	145.375
AMK58_00735	K03439 tRNA (guanine-N7-)-methyltransferase [EC:2.1.1.33] | (GenBank) tRNA (guanine-N7)-methyltra	tRNA synthesis/modification	57.728	49.852	59.583	55.431	35.794	89.058
AMK58_00740	no KO assigned | (GenBank) antibiotic biosynthesis monooxygenase	Biosynthesis	601.915	652.168	611.044	120.996	3809.745	330.394
AMK58_00745	K09748 ribosome maturation factor RimP | (GenBank) ribosome maturation factor RimP	Ribosome	871.938	1018.056	943.217	654.083	3804.449	901.160
AMK58_00750	K02600 N utilization substance protein A | (GenBank) transcription elongation factor NusA	Transcription	45.245	51.803	47.367	116.782	348.473	104.230
AMK58_00755	K07742 uncharacterized protein | (GenBank) hypothetical protein	Hypothetical	1004.416	916.250	1089.313	407.943	2106.231	626.839
AMK58_00760	K02519 translation initiation factor IF-2 | (GenBank) translation initiation factor IF-2	Translation	13.246	9.793	12.763	33.766	32.368	31.508
AMK58_00765	K02834 ribosome-binding factor A | (GenBank) ribosome-binding factor A	Ribosome	578.255	821.589	648.483	239.131	385.957	417.200
AMK58_00770	K03177 tRNA pseudouridine55 synthase [EC:5.4.99.25] | (GenBank) pseudouridine synthase	tRNA synthesis/modification	737.151	795.768	721.240	22.108	265.809	73.412
AMK58_00775	K02956 small subunit ribosomal protein S15 | (GenBank) 30S ribosomal protein S15	Ribosome	8866.710	8203.388	8649.270	1284.272	894.687	1462.001
AMK58_00780	K00962 polyribonucleotide nucleotidyltransferase [EC:2.7.7.8] | (GenBank) polyribonucleotide nucleot	Metabolism: Nucleotide	15.241	16.711	18.399	54.597	19.634	51.133
AMK58_00785	no KO assigned | (GenBank) hypothetical protein	Hypothetical	13.217	22.561	26.928	309.196	115.666	310.109
AMK58_00790	K07054 uncharacterized protein | (GenBank) flagellar biosynthesis protein FlgM	Motility	233.331	200.932	218.015	107.123	171.377	127.793
AMK58_00795	K05770 benzodiazapine receptor | (GenBank) hypothetical protein	Transport	58.683	57.347	49.781	366.452	199.849	273.291
AMK58_00800	K01843 lysine 2,3-aminomutase [EC:5.4.3.2] | (GenBank) lysine 2,3-aminomutase	Metabolism: Amino Acid	29.245	32.149	41.376	238.343	47.994	97.626
AMK58_00805	K01126 glycerophosphoryl diester phosphodiesterase [EC:3.1.4.46] | (GenBank) glycerophosphodiester p	Metabolism: Lipid	781.659	732.036	658.787	344.699	412.635	539.793
AMK58_00810	no KO assigned | (GenBank) 2-nitropropane dioxygenase	No COG	66.095	84.800	73.982	81.437	116.871	73.736
AMK58_00815	K09826 Fur family transcriptional regulator, iron response regulator | (GenBank) Fur family transcri	Transcription	49.607	55.256	40.238	136.657	174.842	161.388
AMK58_00820	no KO assigned | (GenBank) rubrerythrin	No COG	17.585	17.223	21.105	447.556	113.087	141.674
AMK58_00825	no KO assigned | (GenBank) hypothetical protein	Hypothetical	38.320	46.275	42.443	329.286	69.192	164.246
AMK58_00830	no KO assigned | (GenBank) hypothetical protein	Hypothetical	297.997	308.892	300.554	291.642	299.895	352.385
AMK58_00835	no KO assigned | (GenBank) D-glycerate dehydrogenase	Energy	194.087	241.849	198.028	49.803	287.445	92.764
AMK58_00840	no KO assigned | (GenBank) hypothetical protein	Hypothetical	202.003	252.480	229.836	828.761	548.885	800.583
AMK58_00845	K09181 acetyltransferase | (GenBank) GCN5 family acetyltransferase	Acetyltransferase	28.769	34.215	42.875	56.539	35.886	42.608
AMK58_00850	K00016 L-lactate dehydrogenase [EC:1.1.1.27] | (GenBank) L-lactate dehydrogenase	Metabolism: Carbohydrate	577.740	513.721	440.678	103.820	146.784	158.036
AMK58_00855	K01507 inorganic pyrophosphatase [EC:3.6.1.1] | (GenBank) inorganic pyrophosphatase	Energy	50.990	71.298	69.732	713.560	382.915	375.238
AMK58_00860	K13893 microcin C transport system substrate-binding protein | (GenBank) peptide ABC transporter sub	Membrane Transport	10.355	11.104	13.885	76.380	67.450	50.349
AMK58_00865	no KO assigned | (GenBank) hypothetical protein	Hypothetical	122.067	116.606	119.837	452.658	117.501	385.582
AMK58_00870	K03688 ubiquinone biosynthesis protein | (GenBank) 2-octaprenylphenol hydroxylase	Biosynthesis: Co-Factors and Vitamins	60.105	66.272	62.459	103.592	41.208	81.485
AMK58_00875	K03183 demethylmenaquinone methyltransferase / 2-methoxy-6-polyprenyl-1,4-benzoquinol methylase [EC	Biosynthesis: Co-Factors and Vitamins	21.557	38.734	40.530	518.691	43.360	133.611
AMK58_00880	no KO assigned | (GenBank) hypothetical protein	Hypothetical	17.732	19.365	23.084	272.268	28.219	103.724
AMK58_00885	no KO assigned | (GenBank) hypothetical protein	Hypothetical	27.913	30.804	33.471	108.441	46.430	49.898
AMK58_00890	K10563 formamidopyrimidine-DNA glycosylase [EC:3.2.2.23 4.2.99.18] | (GenBank) 5-hydroxymethylura	DNA Repair and Replication	46.103	82.375	57.355	154.490	61.831	104.412
AMK58_00895	no KO assigned | (GenBank) hypothetical protein	Hypothetical	732.476	743.710	755.001	478.483	210.326	350.660
AMK58_00900	K01692 enoyl-CoA hydratase [EC:4.2.1.17] | (GenBank) enoyl-CoA hydratase	Metabolism	971.559	1224.212	904.654	22.543	788.009	117.905
AMK58_00905	K02968 small subunit ribosomal protein S20 | (GenBank) 30S ribosomal protein S20	Ribosome	510.277	579.239	583.534	1094.910	854.624	1089.145
AMK58_00910	K02313 chromosomal replication initiator protein | (GenBank) chromosomal replication initiation prot	DNA Repair and Replication	210.333	265.933	239.850	107.074	72.319	137.776
AMK58_00915	K11069 spermidine/putrescine transport system substrate-binding protein | (GenBank) spermidine/putre	Membrane Transport	65.033	81.830	67.699	164.147	58.527	108.586
AMK58_00920	K11072 spermidine/putrescine transport system ATP-binding protein [EC:3.6.3.31] | (GenBank) ABC tran	Membrane Transport	19.837	23.839	19.620	216.248	46.005	58.566
AMK58_00925	K14166 copper transport protein | (GenBank) hypothetical protein	Transport: Metal	14.226	10.736	6.763	55.366	16.917	59.641
AMK58_00930	K09796 periplasmic copper chaperone A | (GenBank) hypothetical protein	Chaperone	46.412	61.619	49.459	107.824	32.511	92.519
AMK58_00935	no KO assigned | (GenBank) hypothetical protein	Hypothetical	122.895	130.530	98.096	402.010	75.039	528.427
AMK58_00940	K11071 spermidine/putrescine transport system permease protein | (GenBank) ABC transporter permease	Membrane Transport	115.876	125.055	107.704	153.756	92.420	180.519
AMK58_00945	K11070 spermidine/putrescine transport system permease protein | (GenBank) spermidine/putrescine ABC	Membrane Transport	207.920	272.375	181.348	63.273	134.749	65.560
AMK58_00950	no KO assigned | (GenBank) hypothetical protein	Hypothetical	91.678	98.233	80.536	168.860	232.983	271.665
AMK58_00955	K07107 acyl-CoA thioester hydrolase [EC:3.1.2.-] | (GenBank) thioesterase	enzyme	78.103	93.672	98.279	572.901	339.743	509.925
AMK58_00960	no KO assigned | (GenBank) copper oxidase	enzyme	41.042	41.378	45.283	149.930	71.314	76.511
AMK58_00965	K03215 23S rRNA (uracil1939-C5)-methyltransferase [EC:2.1.1.190] | (GenBank) RNA methyltransferase	Ribosome	168.681	164.132	172.296	167.101	220.787	150.191
AMK58_00970	K06969 23S rRNA (cytosine1962-C5)-methyltransferase [EC:2.1.1.191] | (GenBank) SAM-dependent me	Ribosome	136.998	129.833	139.495	60.099	532.553	152.293
AMK58_00975	no KO assigned | (GenBank) hypothetical protein	Hypothetical	6455.726	5252.842	5187.507	238.239	252.964	587.989
AMK58_00980	K00404 cytochrome c oxidase cbb3-type subunit I [EC:1.9.3.1] | (GenBank) cytochrome oxidase	Energy	981.458	1001.681	804.141	37.378	54.092	98.695
AMK58_00985	K00405 cytochrome c oxidase cbb3-type subunit II | (GenBank) peptidase S41	Energy	270.253	268.452	251.677	9.630	22.407	21.969
AMK58_00990	K00407 cytochrome c oxidase cbb3-type subunit IV | (GenBank) cytochrome oxidase	Energy	6197.235	7197.834	6072.169	1045.198	956.013	1474.059
AMK58_00995	K00406 cytochrome c oxidase cbb3-type subunit III | (GenBank) cytochrome C oxidase Cbb3	Energy	149.298	158.845	149.732	291.679	235.251	400.150
AMK58_01000	no KO assigned | (GenBank) cytochrome c oxidase accessory protein CcoG	Energy	62.180	52.803	50.867	79.417	80.794	65.055

AMK58_01005	no KO assigned | (GenBank) hypothetical protein	Hypothetical	224.853	252.141	248.218	693.538	390.652	497.836
AMK58_01010	K01533 Cu2+-exporting ATPase [EC:3.6.3.4] | (GenBank) ATPase P	Energy	131.029	131.546	121.040	35.275	80.531	62.933
AMK58_01015	K01420 CRP/FNR family transcriptional regulator, anaerobic regulatory protein | (GenBank) Crp/Fnr fa	Transcription	241.165	184.269	171.119	495.348	313.678	533.661
AMK58_01020	K00029 malate dehydrogenase (oxaloacetate-decarboxylating)(NADP+) [EC:1.1.1.40] | (GenBank) malic e	Metabolism: Carbon	3.945	3.822	6.336	176.826	17.798	68.766
AMK58_01025	K07636 two-component system, OmpR family, phosphate regulon sensor histidine kinase PhoR [EC:2.7.1	Signal Transduction	41.484	43.709	37.914	125.570	111.965	174.368
AMK58_01030	K02040 phosphate transport system substrate-binding protein | (GenBank) phosphate ABC transporter su	Signal Transduction	36.722	32.584	36.644	220.631	140.861	248.477
AMK58_01035	K02037 phosphate transport system permease protein | (GenBank) phosphate ABC transporter permease	Signal Transduction	18.209	12.030	14.650	141.781	53.434	152.047
AMK58_01040	K02038 phosphate transport system permease protein | (GenBank) phosphate ABC transporter permease	Signal Transduction	13.580	10.285	11.639	104.281	48.697	139.761
AMK58_01045	K02036 phosphate transport system ATP-binding protein [EC:3.6.3.27] | (GenBank) phosphate ABC trans	Signal Transduction	16.853	19.431	14.197	166.543	62.646	138.589
AMK58_01050	K02039 phosphate transport system protein | (GenBank) PhoU family transcriptional regulator	Signal Transduction	37.210	44.462	45.585	211.455	99.094	192.557
AMK58_01055	K07657 two-component system, OmpR family, phosphate regulon response regulator PhoB | (GenBank) tw	Starvation	27.478	31.807	31.025	47.390	50.576	94.434
AMK58_01060	no KO assigned | (GenBank) hypothetical protein	Hypothetical	57.655	45.044	55.662	130.105	96.212	135.299
AMK58_01065	no KO assigned | (GenBank) hypothetical protein	Hypothetical	351.614	376.541	372.954	524.697	336.815	251.197
AMK58_01070	K21029 molybdopterin-synthase adenylyltransferase [EC:2.7.7.80] | (GenBank) adenylyltransferase	Genetic Information Processing	1172.078	1244.098	1344.951	190.553	467.098	287.860
AMK58_01075	no KO assigned | (GenBank) hypothetical protein	Hypothetical	447.068	268.700	313.639	264.525	234.522	268.177
AMK58_01080	K01738 cysteine synthase A [EC:2.5.1.47] | (GenBank) cysteine synthase	Metabolism	46.034	27.879	27.101	31.930	45.364	43.621
AMK58_01085	no KO assigned | (GenBank) Rrf2 family transcriptional regulator	Transcription	26.817	19.036	20.196	152.938	42.678	56.627
AMK58_01090	K01520 dUTP pyrophosphatase [EC:3.6.1.23] | (GenBank) deoxyuridine 5'-triphosphate nucleotidohydrola	Metabolism: Nucleotide	97.594	78.912	94.653	672.007	358.791	390.789
AMK58_01095	K13038 phosphopantothenoylcysteine decarboxylase / phosphopantothenate--cysteine ligase [EC:4.1.1.36	Biosynthesis: Co-Factors and Vitamins	130.322	138.111	138.839	165.412	62.363	239.723
AMK58_01100	no KO assigned | (GenBank) AAA family ATPase	Energy	66.562	93.628	99.017	33.408	114.311	32.501
AMK58_01105	K03704 cold shock protein (beta-ribbon, CspA family) | (GenBank) cold-shock protein	Stress Response	906.331	1136.702	1190.111	3174.982	1430.923	2406.848
AMK58_01110	no KO assigned | (GenBank) hypothetical protein	Hypothetical	16.453	16.132	18.074	50.975	20.355	15.657
AMK58_01115	K11709 manganese/zinc/iron transport system permease protein | (GenBank) zinc ABC transporter permea	Transport: Metal	64.401	70.357	69.515	216.412	104.242	57.670
AMK58_01120	K11708 manganese/zinc/iron transport system permease protein | (GenBank) manganese transporter	Transport: Metal	48.691	50.383	62.141	169.448	192.291	105.474
AMK58_01125	K11710 manganese/zinc/iron transport system ATP- binding protein | (GenBank) manganese ABC transpo	Transport: Metal	142.806	172.228	157.990	227.535	938.860	187.939
AMK58_01130	K11707 manganese/zinc/iron transport system substrate-binding protein | (GenBank) manganese transpor	Transport: Metal	29.478	40.816	43.305	128.969	47.498	78.366
AMK58_01135	K09134 adenosyl-fluoride synthase [EC:2.5.1.63] | (GenBank) hypothetical protein	Transferase	16.273	15.556	21.198	224.968	53.047	66.245
AMK58_01140	no KO assigned | (GenBank) hypothetical protein	Hypothetical	91.337	76.143	105.309	354.858	351.659	212.721
AMK58_01145	no KO assigned | (GenBank) haloacid dehalogenase	enzyme	106.352	97.275	107.049	33.644	87.419	83.038
AMK58_01150	no KO assigned | (GenBank) pseudogene	Pseudogene	1197.833	1546.373	1129.189	268.868	593.366	561.847
AMK58_01155	K03711 Fur family transcriptional regulator, ferric uptake regulator | (GenBank) Fur family transcri	Transcription	40.915	39.909	50.017	341.150	307.539	437.723
AMK58_01160	no KO assigned | (GenBank) transglutaminase	enzyme	69.920	64.503	78.579	251.371	162.532	117.514
AMK58_01165	no KO assigned | (GenBank) histidine kinase	Signal Transduction	206.760	169.631	182.802	176.448	1080.653	295.437
AMK58_01170	K04079 molecular chaperone HtpG | (GenBank) heat-shock protein Hsp90	Chaperone	74.567	94.151	119.504	207.924	95.553	124.388
AMK58_01175	no KO assigned | (GenBank) hypothetical protein	Hypothetical	46.315	64.506	70.516	359.881	259.715	195.264
AMK58_01180	K17675 ATP-dependent RNA helicase SUPV3L1/SUV3 [EC:3.6.4.13] | (GenBank) disulfide oxidoreduct	Hydrolase	11.944	18.415	14.149	35.869	16.294	25.991
AMK58_01185	K04762 ribosome-associated heat shock protein Hsp15 | (GenBank) RNA-binding protein S4	Stress Response	186.864	165.150	164.005	173.294	294.247	316.047
AMK58_01190	no KO assigned | (GenBank) hypothetical protein	Hypothetical	166.561	187.756	175.981	40.768	84.408	142.024
AMK58_01195	no KO assigned | (GenBank) hypothetical protein	Hypothetical	18.627	23.606	19.857	183.357	41.558	61.238
AMK58_01200	no KO assigned | (GenBank) SAM-dependent methyltransferase	Methyltransferase	34.846	48.224	48.294	586.243	87.852	247.358
AMK58_01205	no KO assigned | (GenBank) MFS transporter	Transport: MFS	20.893	20.702	24.613	153.585	62.319	92.991
AMK58_01210	no KO assigned | (GenBank) transcriptional regulator	Transcription	66.958	82.199	74.130	46.865	82.628	86.146
AMK58_01215	K05524 ferredoxin | (GenBank) ferredoxin	Ferredoxin	2140.442	2120.105	2122.613	218.683	5126.177	688.452
AMK58_01220	K07736 CarD family transcriptional regulator | (GenBank) CarD family transcriptional regulator	Transcription	341.716	365.287	373.390	606.012	1204.466	522.581
AMK58_01225	K01259 proline iminopeptidase [EC:3.4.11.5] | (GenBank) proline iminopeptidase	Metabolism: Amino Acid	4.201	6.846	8.958	148.031	88.296	79.451
AMK58_01230	no KO assigned | (GenBank) nuclease	DNA Repair and Replication	35.151	39.754	41.536	514.461	347.631	287.448
AMK58_01235	no KO assigned | (GenBank) peptidase	Peptidase	66.769	75.442	80.277	249.194	288.143	272.906
AMK58_01240	K01768 adenylate cyclase [EC:4.6.1.1] | (GenBank) guanylate cyclase	cyclin nucleotide	223.197	208.505	247.617	97.525	841.585	149.579
AMK58_01245	K03089 RNA polymerase sigma-32 factor | (GenBank) RNA polymerase subunit sigma-70	Transcription	31.107	24.032	31.234	213.015	167.140	207.009
AMK58_01250	no KO assigned | (GenBank) hypothetical protein	Hypothetical	445.573	401.569	390.501	13.515	340.233	59.162
AMK58_01255	K02916 large subunit ribosomal protein L35 | (GenBank) 50S ribosomal protein L35	Ribosome	2547.389	2400.389	2012.577	151.696	2368.428	404.953
AMK58_01260	K02887 large subunit ribosomal protein L20 | (GenBank) rplT; 50S ribosomal protein L20	Ribosome	486.252	400.568	391.157	382.794	369.866	548.975
AMK58_01265	K01889 phenylalanyl-tRNA synthetase alpha chain [EC:6.1.1.20] | (GenBank) phenylalanine--tRNA ligase	tRNA synthesis/modification	202.812	188.289	208.033	345.936	205.287	301.383
AMK58_01270	K01890 phenylalanyl-tRNA synthetase beta chain [EC:6.1.1.20] | (GenBank) phenylalanine--tRNA ligase	tRNA synthesis/modification	27.629	38.325	38.853	55.551	21.575	32.739
AMK58_01275	no KO assigned | (GenBank) regulator	Signal Transduction	501.115	592.750	562.804	817.687	492.960	783.705
AMK58_01280	no KO assigned | (GenBank) phosphodiesterase	enzyme	18.005	28.260	21.617	118.752	20.152	55.675
AMK58_01285	no KO assigned | (GenBank) hypothetical protein	Hypothetical	23.710	15.070	26.741	260.173	36.717	85.956
AMK58_01290	no KO assigned | (GenBank) LysR family transcriptional regulator	Transcription	129.010	112.794	104.973	127.183	47.210	123.433
AMK58_01295	K06077 outer membrane lipoprotein SlyB | (GenBank) hypothetical protein	Membrane	593.636	518.741	542.725	254.156	611.064	491.869
AMK58_01300	K03596 GTP-binding protein LepA | (GenBank) GTP-binding protein LepA	cyclic nucleotide	28.875	29.287	27.710	194.749	31.702	132.320
AMK58_01305	no KO assigned | (GenBank) hypothetical protein	Hypothetical	102.872	113.694	121.850	236.937	34.307	170.664
AMK58_01310	K00703 starch synthase [EC:2.4.1.21] | (GenBank) glycogen synthase	Metabolism: Carbohydrate	323.767	305.678	294.063	113.497	66.344	169.657
AMK58_01315	K00975 glucose-1-phosphate adenylyltransferase [EC:2.7.7.27] | (GenBank) glgC; glucose-1-phosphate a	Metabolism: Carbohydrate	19.037	14.217	18.824	276.303	41.834	224.451
AMK58_01320	K03320 ammonium transporter, Amt family | (GenBank) ammonia channel protein	Transport: Inorganic	27.488	30.758	31.555	259.842	61.278	108.434
AMK58_01325	no KO assigned | (GenBank) methyltransferase	Methyltransferase	63.909	66.556	73.401	137.969	110.061	142.534
AMK58_01330	K13652 AraC family transcriptional regulator | (GenBank) transcriptional regulator	Transcription	248.118	266.589	286.716	239.662	250.942	237.717
AMK58_01335	no KO assigned | (GenBank) L-asparaginase II	Biosynthesis: Amino Acid	39.039	29.565	34.295	116.668	119.159	97.484

AMK58_01340	no KO assigned | (GenBank) rRNA methyltransferase	Methyltransferase	107.208	131.070	123.581	205.435	326.158	223.580
AMK58_01345	K07167 putative transcriptional regulator | (GenBank) hypothetical protein	Transcription	81.245	95.816	81.941	253.548	315.966	86.449
AMK58_01350	K03088 RNA polymerase sigma-70 factor, ECF subfamily | (GenBank) RNA polymerase subunit sigma	Transcription	214.824	204.700	214.449	216.248	153.814	176.396
AMK58_01355	no KO assigned | (GenBank) hypothetical protein	Hypothetical	357.428	436.065	394.956	274.911	1046.035	482.521
AMK58_01360	K06941 23S rRNA (adenine2503-C2)-methyltransferase [EC:2.1.1.192] | (GenBank) 23S rRNA (adenine(	Ribosome	21.110	19.784	19.974	16.357	52.692	16.984
AMK58_01365	no KO assigned | (GenBank) hypothetical protein	Hypothetical	84.803	88.512	132.605	253.886	269.315	257.225
AMK58_01370	no KO assigned | (GenBank) hypothetical protein	Hypothetical	3198.004	2652.304	2456.240	547.377	1032.476	854.617
AMK58_01375	K01940 argininosuccinate synthase [EC:6.3.4.5] | (GenBank) argininosuccinate synthase	Biosynthesis: Amino Acid	38.399	45.649	49.177	248.396	67.496	249.791
AMK58_01380	K03406 methyl-accepting chemotaxis protein | (GenBank) hypothetical protein	Chemotaxis	33.619	22.214	32.186	54.685	17.240	61.630
AMK58_01385	K07032 uncharacterized protein | (GenBank) glyoxalase	enzyme	127.476	144.671	109.826	170.388	77.902	219.636
AMK58_01390	no KO assigned | (GenBank) rhodanese	enzyme	7.890	7.252	15.003	166.657	165.768	197.527
AMK58_01395	no KO assigned | (GenBank) hypothetical protein	Hypothetical	62.992	71.684	67.671	641.803	234.485	277.893
AMK58_01400	no KO assigned | (GenBank) hypothetical protein	Hypothetical	40.637	42.404	50.679	308.153	134.529	122.464
AMK58_01405	K02200 cytochrome c-type biogenesis protein CcmH | (GenBank) cytochrome C biogenesis protein	Energy	15.542	15.975	16.684	59.323	45.834	43.079
AMK58_01410	K02200 cytochrome c-type biogenesis protein CcmH | (GenBank) cytochrome C biogenesis protein CcmH	Energy	90.373	87.276	84.279	156.216	304.749	175.177
AMK58_01415	K02199 cytochrome c biogenesis protein CcmG, thiol:disulfide interchange protein DsbE | (GenBank) di	Energy	275.067	255.936	282.331	781.029	1356.877	561.976
AMK58_01420	K02198 cytochrome c-type biogenesis protein CcmF | (GenBank) cytochrome C biogenesis protein CcmF	Energy	23.644	23.603	26.410	25.077	93.803	29.138
AMK58_01425	K02197 cytochrome c-type biogenesis protein CcmE | (GenBank) cytochrome C biogenesis protein CcmE	Energy	5.576	1.586	5.049	24.897	50.736	38.900
AMK58_01430	K02196 heme exporter protein D | (GenBank) cytochrome C biogenesis protein CcmD	Energy	514.706	442.601	558.248	596.903	3102.179	736.674
AMK58_01435	K02195 heme exporter protein C | (GenBank) heme transporter HemC	Membrane Transport	32.623	42.196	46.048	347.573	148.946	178.696
AMK58_01440	no KO assigned | (GenBank) LysR family transcriptional regulator	Transcription	5.399	4.032	7.700	88.777	35.351	47.686
AMK58_01445	no KO assigned | (GenBank) hypothetical protein	Hypothetical	44.705	44.514	51.951	728.589	264.509	434.109
AMK58_01450	K01104 protein-tyrosine phosphatase [EC:3.1.3.48] | (GenBank) phosphotyrosine protein phosphatase	Signal Transduction	57.983	41.204	56.831	432.496	414.022	291.333
AMK58_01455	K12410 NAD-dependent deacetylase [EC:3.5.1.-] | (GenBank) NAD-dependent deacylase	Energy	21.833	20.752	23.590	196.750	160.449	45.859
AMK58_01460	no KO assigned | (GenBank) uracil-DNA glycosylase	DNA Repair and Replication	44.999	60.280	49.885	83.255	246.329	97.862
AMK58_01465	no KO assigned | (GenBank) acetylglutamate kinase	Signal Transduction	174.886	193.943	176.849	108.907	245.559	155.117
AMK58_01470	K01724 4a-hydroxytetrahydrobiopterin dehydratase [EC:4.2.1.96] | (GenBank) pterin-4-alpha-carbinolam	Biosynthesis	131.064	159.950	139.055	551.325	262.754	203.419
AMK58_01475	K03813 molybdenum transport protein [EC:2.4.2.-] | (GenBank) pyrophosphorylase	Transport	53.957	65.521	59.089	233.093	132.806	187.252
AMK58_01480	K03284 magnesium transporter | (GenBank) magnesium transporter	Transport: Metal	95.759	93.525	95.467	176.275	104.476	74.620
AMK58_01485	K01491 methylenetetrahydrofolate dehydrogenase (NADP+) / methenyltetrahydrofolate cyclohydrolase [E(	Energy	34.098	54.112	27.735	84.751	28.895	71.018
AMK58_01490	K09131 uncharacterized protein | (GenBank) hypothetical protein	Hypothetical	251.566	239.109	220.645	112.629	108.875	198.655
AMK58_01495	K02221 YggT family protein | (GenBank) hypothetical protein	Secretion System	21.666	22.843	31.102	109.262	104.099	157.571
AMK58_01500	K14218 tRNA Ala | (GenBank) tRNA-Ala	tRNA synthesis/modification	393.493	485.410	524.094	2341.025	2807.817	1948.470
AMK58_01505	no KO assigned | (GenBank) hypothetical protein	Hypothetical	69.869	82.928	84.398	119.843	46.676	130.440
AMK58_01510	K10019 octopine/nopaline transport system permease protein | (GenBank) ABC transporter permease	Membrane Transport	407.443	363.774	389.903	208.491	114.792	166.615
AMK58_01515	K10020 octopine/nopaline transport system permease protein | (GenBank) ABC transporter permease	Membrane Transport	2106.530	2133.129	2110.677	260.188	289.174	281.072
AMK58_01520	no KO assigned | (GenBank) amino acid ABC transporter substrate-binding protein	Membrane Transport	198.017	228.109	203.460	146.434	75.553	157.366
AMK58_01525	no KO assigned | (GenBank) amino acid transporter	Transport: Amino Acid	70.064	75.294	85.590	429.883	97.306	274.622
AMK58_01530	K07085 putative transport protein | (GenBank) transporter	Transport	28.324	39.017	30.698	257.040	23.507	193.512
AMK58_01535	K07085 putative transport protein | (GenBank) transporter	Transport	14.918	13.348	16.034	85.534	26.490	47.581
AMK58_01540	K02193 heme exporter protein A [EC:3.6.3.41] | (GenBank) cytochrome C biogenesis protein CcmA	Energy	26.321	34.595	33.368	191.332	46.942	146.439
AMK58_01545	K02194 heme exporter protein B | (GenBank) heme transporter	Membrane Transport	801.348	800.470	659.082	50.166	2626.435	228.754
AMK58_01550	K03671 thioredoxin 1 | (GenBank) thioredoxin	Chaperone	258.966	301.398	310.182	1763.521	395.678	1194.425
AMK58_01555	K16898 ATP-dependent helicase/nuclease subunit A [EC:3.1.-.- 3.6.4.12] | (GenBank) DNA helicase UvrI	DNA Repair and Replication	29.057	30.218	22.453	30.138	35.915	54.178
AMK58_01560	no KO assigned | (GenBank) hypothetical protein	Hypothetical	104.160	111.695	132.189	724.868	409.614	273.107
AMK58_01565	K16899 ATP-dependent helicase/nuclease subunit B [EC:3.1.-.- 3.6.4.12] | (GenBank) double-strand bre	DNA Repair and Replication	13.819	12.627	13.564	54.225	29.558	50.237
AMK58_01570	K00992 MurNAc alpha-1-phosphate uridylyltransferase [EC:2.7.7.-] | (GenBank) mannose-1-phosphate gu	Metabolism: Carbohydrate	28.872	31.531	30.551	311.480	88.286	168.405
AMK58_01575	K07102 anomeric MurNAc/GlcNAc kinase [EC:2.7.1.-] | (GenBank) aminoglycoside phosphotransferase	Signal Transduction	5.612	4.280	7.947	58.860	24.024	25.244
AMK58_01580	K06925 tRNA threonylcarbamoyladenosine biosynthesis protein TsaE | (GenBank) tRNA threonylcarbamo	tRNA synthesis/modification	33.794	31.955	41.378	273.241	96.194	236.939
AMK58_01585	no KO assigned | (GenBank) hypothetical protein	Hypothetical	348.161	285.267	315.091	496.837	394.223	519.708
AMK58_01590	K00384 thioredoxin reductase (NADPH) [EC:1.8.1.9] | (GenBank) ferredoxin-NADP reductase	Energy	39.530	43.133	58.066	201.028	131.038	73.091
AMK58_01595	no KO assigned | (GenBank) hypothetical protein	Hypothetical	58.953	82.056	74.536	247.949	97.142	191.623
AMK58_01600	no KO assigned | (GenBank) two-component system response regulator	Signal Transduction	33.187	34.873	49.631	390.721	76.482	169.332
AMK58_01605	no KO assigned | (GenBank) histidine kinase	Signal Transduction	5.703	9.959	12.263	226.355	158.184	134.154
AMK58_01610	K03885 NADH dehydrogenase [EC:1.6.99.3] | (GenBank) pyridine nucleotide-disulfide oxidoreductase	Energy	106.407	129.057	151.632	266.976	181.857	162.634
AMK58_01615	no KO assigned | (GenBank) histidine kinase	Signal Transduction	32.337	43.068	46.289	324.236	61.741	182.644
AMK58_01620	no KO assigned | (GenBank) hypothetical protein	Hypothetical	8.653	10.180	13.723	82.078	15.284	53.378
AMK58_01625	K09969 general L-amino acid transport system substrate-binding protein | (GenBank) hypothetical prot	Transport: Amino Acid	297.063	274.058	276.894	164.845	378.285	171.063
AMK58_01630	K01696 tryptophan synthase beta chain [EC:4.2.1.20] | (GenBank) tryptophan synthase subunit beta	Biosynthesis: Amino Acid	123.560	122.341	117.765	111.852	108.048	46.364
AMK58_01635	K01695 tryptophan synthase alpha chain [EC:4.2.1.20] | (GenBank) trpA; tryptophan synthase subunit a	Biosynthesis: Amino Acid	400.609	357.871	364.745	161.237	250.539	124.744
AMK58_01640	K01963 acetyl-CoA carboxylase carboxyl transferase subunit beta [EC:6.4.1.2] | (GenBank) acetyl-CoA	Metabolism	99.847	95.858	121.221	230.962	147.330	113.071
AMK58_01645	K11754 dihydrofolate synthase / folylpolyglutamate synthase [EC:6.3.2.12 6.3.2.17] | (GenBank) bifun	Biosynthesis: Co-Factors and Vitamins	104.671	134.202	121.322	305.519	187.534	180.029
AMK58_01650	no KO assigned | (GenBank) hypothetical protein	Hypothetical	2.553	2.119	2.023	7.126	5.342	15.324
AMK58_01655	K14219 tRNA Arg | (GenBank) tRNA-Arg	tRNA synthesis/modification	617.953	648.202	895.536	1636.603	1385.676	1418.680
AMK58_01660	K13938 dihydromonapterin reductase / dihydrofolate reductase [EC:1.5.1.- 1.5.1.3] | (GenBank) hypoth	Biosynthesis: Co-Factors and Vitamins	180.865	178.606	175.126	114.131	65.340	222.229
AMK58_01665	no KO assigned | (GenBank) oxidoreductase	Oxidoreductase	422.575	490.747	462.959	709.484	312.481	469.111
AMK58_01670	no KO assigned | (GenBank) hypothetical protein	Hypothetical	75.701	65.547	66.165	77.366	39.198	71.722

AMK58_01675	K01495 GTP cyclohydrolase I [EC:3.5.4.16] | (GenBank) hypothetical protein	Biosynthesis: Co-Factors and Vitamins	12.985	18.870	17.018	214.838	75.147	43.769
AMK58_01680	no KO assigned | (GenBank) hypothetical protein	Hypothetical	48.341	49.460	54.537	125.947	118.812	138.041
AMK58_01685	K03976 Cys-tRNA(Pro)/Cys-tRNA(Cys) deacylase [EC:3.1.1.-] | (GenBank) cys-tRNA(Pro)/cys-tRNA(C	tRNA synthesis/modification	9.024	17.972	14.777	221.620	143.701	80.193
AMK58_01690	no KO assigned | (GenBank) hypothetical protein	Hypothetical	120.869	108.257	113.357	119.709	1107.956	194.754
AMK58_01695	K01495 GTP cyclohydrolase I [EC:3.5.4.16] | (GenBank) GTP cyclohydrolase	Biosynthesis: Co-Factors and Vitamins	145.435	171.607	155.566	99.357	141.815	165.707
AMK58_01700	K06195 ApaG protein | (GenBank) Co2+/Mg2+ efflux protein ApaG	Inorganic Efflux	83.181	119.639	97.544	322.308	253.487	365.948
AMK58_01705	no KO assigned | (GenBank) GCN5 family acetyltransferase	Acetyltransferase	31.186	29.592	19.884	20.642	83.306	43.547
AMK58_01710	K14225 tRNA Gly | (GenBank) tRNA-Gly	tRNA synthesis/modification	178.340	107.526	199.125	425.190	99.311	2674.749
AMK58_01715	no KO assigned | (GenBank) hypothetical protein	Hypothetical	38.524	31.053	41.571	233.859	134.031	706.505
AMK58_01720	K07260 D-alanyl-D-alanine carboxypeptidase [EC:3.4.16.4] | (GenBank) hypothetical protein	Cell Wall	13.766	16.822	28.110	128.030	115.697	243.686
AMK58_01725	no KO assigned | (GenBank) hypothetical protein	Hypothetical	28.412	46.295	41.136	1059.831	257.307	494.136
AMK58_01730	K03654 ATP-dependent DNA helicase RecQ [EC:3.6.4.12] | (GenBank) hypothetical protein	DNA Repair and Replication	4.158	6.763	6.040	22.900	13.763	41.043
AMK58_01735	no KO assigned | (GenBank) hypothetical protein	Hypothetical	81.080	58.650	94.492	215.923	14.713	394.236
AMK58_01740	no KO assigned | (GenBank) hypothetical protein	Hypothetical	18.444	13.092	19.621	53.328	31.201	170.691
AMK58_01745	no KO assigned | (GenBank) hypothetical protein	Hypothetical	56.031	72.570	90.158	674.462	511.340	1452.239
AMK58_01750	no KO assigned | (GenBank) hypothetical protein	Hypothetical	8.760	11.215	13.802	26.738	19.482	78.212
AMK58_01755	no KO assigned | (GenBank) hypothetical protein	Hypothetical	2.703	7.535	4.797	26.790	37.264	85.205
AMK58_01760	no KO assigned | (GenBank) hypothetical protein	Hypothetical	3.174	8.128	8.192	211.388	81.044	245.143
AMK58_01765	no KO assigned | (GenBank) hypothetical protein	Hypothetical	5.765	4.306	5.824	116.812	67.237	201.484
AMK58_01770	no KO assigned | (GenBank) hypothetical protein	Hypothetical	0.992	3.953	4.403	206.056	42.018	57.362
AMK58_01775	no KO assigned | (GenBank) hypothetical protein	Hypothetical	2.328	4.637	2.583	145.032	30.098	143.216
AMK58_01780	no KO assigned | (GenBank) hypothetical protein	Hypothetical	496.407	568.351	884.223	844.139	196.363	1050.523
AMK58_01785	no KO assigned | (GenBank) hypothetical protein	Hypothetical	109.168	122.133	145.777	109.959	30.796	145.003
AMK58_01790	no KO assigned | (GenBank) hypothetical protein	Hypothetical	479.497	546.444	670.603	302.318	70.553	378.473
AMK58_01795	K04043 molecular chaperone DnaK | (GenBank) hypothetical protein	Chaperone	216.926	236.095	337.116	269.443	54.088	364.397
AMK58_01800	no KO assigned | (GenBank) hypothetical protein	Hypothetical	123.514	141.456	139.575	108.893	28.590	169.452
AMK58_01805	no KO assigned | (GenBank) hypothetical protein	Hypothetical	91.932	99.838	93.534	30.953	19.594	163.058
AMK58_01810	no KO assigned | (GenBank) hypothetical protein	Hypothetical	87.955	82.557	83.420	116.498	104.905	301.216
AMK58_01815	K12132 eukaryotic-like serine/threonine-protein kinase [EC:2.7.11.1] | (GenBank) serine/threonine pr	Signal Transduction	26.270	23.765	27.499	74.488	24.871	95.057
AMK58_01820	no KO assigned | (GenBank) hypothetical protein	Hypothetical	370.543	359.804	337.404	169.189	197.441	545.836
AMK58_01825	K06915 uncharacterized protein | (GenBank) nucleoside triphosphate hydrolase	Hydrolase	41.938	45.907	40.012	55.505	36.970	135.310
AMK58_01830	no KO assigned | (GenBank) hypothetical protein	Hypothetical	29.715	25.182	34.165	111.370	25.347	117.057
AMK58_01835	no KO assigned | (GenBank) hypothetical protein	Hypothetical	332.623	366.535	342.124	655.476	195.582	1063.752
AMK58_01840	no KO assigned | (GenBank) hypothetical protein	Hypothetical	2.856	4.437	4.807	45.989	7.063	17.560
AMK58_01845	no KO assigned | (GenBank) hypothetical protein	Hypothetical	14.711	9.334	17.577	174.742	29.706	64.827
AMK58_01850	no KO assigned | (GenBank) hypothetical protein	Hypothetical	43.205	37.656	39.340	58.692	44.450	117.266
AMK58_01855	no KO assigned | (GenBank) hypothetical protein	Hypothetical	523.850	429.191	457.790	795.792	1194.898	1533.978
AMK58_01860	K03427 type I restriction enzyme M protein [EC:2.1.1.72] | (GenBank) restriction endonuclease subuni	DNA Repair and Replication	9.196	8.749	9.858	31.229	89.807	36.120
AMK58_01865	no KO assigned | (GenBank) hypothetical protein	Hypothetical	279.966	301.873	315.444	565.735	1755.995	873.700
AMK58_01870	no KO assigned | (GenBank) hypothetical protein	Hypothetical	27.342	25.051	24.336	45.871	62.941	193.596
AMK58_01875	no KO assigned | (GenBank) hypothetical protein	Hypothetical	5.942	10.355	11.535	204.474	159.610	491.897
AMK58_01880	no KO assigned | (GenBank) hypothetical protein	Hypothetical	11.707	11.248	11.803	93.860	82.198	58.590
AMK58_01885	no KO assigned | (GenBank) hypothetical protein	Hypothetical	21.405	22.534	22.055	31.578	8.373	22.996
AMK58_01890	no KO assigned | (GenBank) hypothetical protein	Hypothetical	48.855	25.381	39.383	302.320	104.457	363.892
AMK58_01895	no KO assigned | (GenBank) hypothetical protein	Hypothetical	254.787	199.714	207.757	543.350	131.511	398.047
AMK58_01900	K02049 NitT/TauT family transport system ATP-binding protein | (GenBank) sulfonate ABC transporter A	Transport: NitT/TauT	187.980	182.443	176.107	180.069	41.570	210.595
AMK58_01905	K02050 NitT/TauT family transport system permease protein | (GenBank) ABC transporter permease	Transport: NitT/TauT	894.480	890.213	773.296	182.125	147.305	325.283
AMK58_01910	K02051 NitT/TauT family transport system substrate-binding protein | (GenBank) hypothetical protein	Transport: NitT/TauT	19.336	20.933	20.852	34.419	100.356	52.585
AMK58_01915	no KO assigned | (GenBank) hypothetical protein	Hypothetical	518.113	602.796	727.012	2453.244	1360.932	1348.247
AMK58_01920	K20975 two-component system, sensor histidine kinase [EC:2.7.13.3] | (GenBank) histidine kinase	Signal Transduction	1.349	1.466	1.788	7.121	4.183	17.100
AMK58_01925	no KO assigned | (GenBank) hypothetical protein	Hypothetical	923.609	1039.577	966.873	2431.902	667.823	1554.598
AMK58_01930	no KO assigned | (GenBank) hypothetical protein	Hypothetical	3.553	3.821	5.810	116.405	139.208	98.961
AMK58_01935	no KO assigned | (GenBank) hypothetical protein	Hypothetical	1.333	2.896	4.955	199.201	101.218	109.235
AMK58_01940	K07010 putative glutamine amidotransferase | (GenBank) glutamine amidotransferase	Peptidase	488.369	556.194	588.332	767.544	557.525	561.348
AMK58_01945	no KO assigned | (GenBank) hypothetical protein	Hypothetical	8.345	10.661	13.473	82.183	798.921	43.896
AMK58_01950	no KO assigned | (GenBank) hypothetical protein	Hypothetical	18.695	21.275	40.348	942.904	84.058	327.178
AMK58_01955	no KO assigned | (GenBank) phospholipase	Membrane	47.033	66.468	68.065	111.310	17.508	114.902
AMK58_01960	K03406 methyl-accepting chemotaxis protein | (GenBank) hypothetical protein	Chemotaxis	6.919	10.334	17.085	109.025	131.881	64.956
AMK58_01965	no KO assigned | (GenBank) hypothetical protein	Hypothetical	0.946	0.942	2.248	51.105	22.859	27.629
AMK58_01970	no KO assigned | (GenBank) hypothetical protein	Hypothetical	2.306	1.378	3.289	40.469	22.034	70.570
AMK58_01975	no KO assigned | (GenBank) hypothetical protein	Hypothetical	8.892	5.449	13.441	275.502	131.736	193.459
AMK58_01980	no KO assigned | (GenBank) hypothetical protein	Hypothetical	1.636	5.431	3.457	73.300	27.586	20.566
AMK58_01985	no KO assigned | (GenBank) hypothetical protein	Hypothetical	64.125	50.903	83.141	458.325	420.881	258.220
AMK58_01990	no KO assigned | (GenBank) hypothetical protein	Hypothetical	98.615	119.511	102.773	378.669	680.466	246.668
AMK58_01995	K04772 serine protease DegQ [EC:3.4.21.-] | (GenBank) peptidase	Peptidase	43.327	54.531	56.148	220.520	41.780	113.799
AMK58_02000	no KO assigned | (GenBank) hypothetical protein	Hypothetical	35.902	47.336	56.265	261.686	34.649	128.111
AMK58_02005	no KO assigned | (GenBank) hypothetical protein	Hypothetical	311.566	307.497	341.474	185.337	184.873	91.665

AMK58_02010	K02498 HemY protein | (GenBank) heme biosynthesis protein HemY	Membrane Transport	198.099	192.382	192.028	178.174	165.049	85.516
AMK58_02015	no KO assigned | (GenBank) hypothetical protein	Hypothetical	69.213	90.611	77.785	84.536	66.457	76.634
AMK58_02020	K01719 uroporphyrinogen-III synthase [EC:4.2.1.75] | (GenBank) uroporphyrinogen III synthase	Metabolism: Co-Factors and Vitamins	93.208	126.300	139.483	239.846	82.808	100.686
AMK58_02025	K01749 hydroxymethylbilane synthase [EC:2.5.1.61] | (GenBank) porphobilinogen deaminase	Metabolism: Co-Factors and Vitamins	19.200	17.947	27.321	156.237	27.013	120.142
AMK58_02030	no KO assigned | (GenBank) hypothetical protein	Hypothetical	28.137	39.054	38.238	318.661	50.955	90.791
AMK58_02035	K01409 N6-L-threonylcarbamoyladenine synthase [EC:2.3.1.234] | (GenBank) tRNA threonylcarbamoyl a	tRNA synthesis/modification	14.993	22.900	17.900	107.218	21.795	55.194
AMK58_02040	no KO assigned | (GenBank) GNAT family acetyltransferase	Acetyltransferase	31.821	27.821	40.587	448.090	96.545	150.173
AMK58_02045	K00057 glycerol-3-phosphate dehydrogenase (NAD(P)+) [EC:1.1.1.94] | (GenBank) glycerol-3-phosphate	Energy	21.676	25.757	34.160	70.906	20.192	44.530
AMK58_02050	K09780 uncharacterized protein | (GenBank) hypothetical protein	Hypothetical	299.501	413.709	306.984	136.578	159.492	286.780
AMK58_02055	no KO assigned | (GenBank) ubiquinol-cytochrome C reductase	Energy	179.273	338.964	354.533	191.567	91.386	201.760
AMK58_02060	no KO assigned | (GenBank) hypothetical protein	Hypothetical	18766.231	16561.828	17241.459	652.605	6297.956	1361.604
AMK58_02065	K01895 acetyl-CoA synthetase [EC:6.2.1.1] | (GenBank) acetyl-coenzyme A synthetase	Metabolism: Carbon	2.433	1.863	3.796	47.294	31.902	23.888
AMK58_02070	no KO assigned | (GenBank) hypothetical protein	Hypothetical	20.688	24.314	27.087	409.781	213.861	154.725
AMK58_02075	no KO assigned | (GenBank) hypothetical protein	Hypothetical	47.892	59.476	49.885	157.140	117.040	184.122
AMK58_02080	no KO assigned | (GenBank) hypothetical protein	Hypothetical	532.607	507.810	493.035	1098.441	3331.534	1313.782
AMK58_02085	K03799 heat shock protein HtpX [EC:3.4.24.-] | (GenBank) heat-shock protein HtpX	Stress Response	133.848	100.669	110.338	64.802	321.675	208.537
AMK58_02090	no KO assigned | (GenBank) exopolyphosphatase	Membrane	8.565	8.528	12.092	180.438	38.509	45.846
AMK58_02095	no KO assigned | (GenBank) hypothetical protein	Hypothetical	76.127	88.883	86.226	372.285	218.639	147.198
AMK58_02100	K03500 16S rRNA (cytosine967-C5)-methyltransferase [EC:2.1.1.176] | (GenBank) MFS transporter	Ribosome	74.189	109.300	93.179	203.440	149.837	139.293
AMK58_02105	no KO assigned | (GenBank) hypothetical protein	Hypothetical	178.060	187.731	181.488	239.109	124.979	174.008
AMK58_02110	K00602 phosphoribosylaminoimidazolecarboxamide formyltransferase / IMP cyclohydrolase [EC:2.1.2.3 3	Metabolism: Nucleotide	64.950	69.857	60.966	47.089	34.002	42.739
AMK58_02115	no KO assigned | (GenBank) hypothetical protein	Hypothetical	293.606	301.398	297.870	1223.828	362.348	848.104
AMK58_02120	K06902 MFS transporter, UMF1 family | (GenBank) MFS transporter	Transport: MFS	76.376	113.111	85.527	194.969	48.063	124.687
AMK58_02125	no KO assigned | (GenBank) hypothetical protein	Hypothetical	35.978	39.252	33.019	44.423	36.749	96.459
AMK58_02130	no KO assigned | (GenBank) hypothetical protein	Hypothetical	620.774	688.457	770.994	1168.734	366.497	1202.467
AMK58_02135	no KO assigned | (GenBank) hypothetical protein	Hypothetical	3398.166	3256.206	3768.297	558.971	281.592	472.288
AMK58_02140	K15371 glutamate dehydrogenase [EC:1.4.1.2] | (GenBank) NAD-glutamate dehydrogenase	Energy	3.216	4.617	4.432	69.656	12.385	50.067
AMK58_02145	no KO assigned | (GenBank) transcriptional regulator	Transcription	81.152	74.591	78.126	133.599	57.929	145.251
AMK58_02150	no KO assigned | (GenBank) 3-phosphoglycerate dehydrogenase	Membrane	20.677	17.861	17.410	319.199	93.109	357.715
AMK58_02155	K03406 methyl-accepting chemotaxis protein | (GenBank) chemotaxis protein	Chemotaxis	13.405	14.058	11.729	119.490	45.044	111.278
AMK58_02160	no KO assigned | (GenBank) 3-phosphoglycerate dehydrogenase	Membrane	9.228	13.232	14.507	200.260	69.568	239.828
AMK58_02165	K07088 uncharacterized protein | (GenBank) transporter	Transport	15.362	17.112	20.796	153.989	48.876	163.334
AMK58_02170	no KO assigned | (GenBank) dimethylmenaquinone methyltransferase	Methyltransferase	1002.171	3324.448	1567.519	150.704	608.003	155.820
AMK58_02175	K14225 tRNA Gly | (GenBank) tRNA-Gly	tRNA synthesis/modification	511.743	453.303	658.480	1288.837	391.948	2071.750
AMK58_02180	K19055 Ala-tRNA(Pro) deacylase [EC:3.1.1.-] | (GenBank) DNA-binding protein	tRNA synthesis/modification	542.808	540.922	577.219	399.315	863.364	419.378
AMK58_02185	K05838 putative thioredoxin | (GenBank) thioredoxin	Chaperone	127.801	136.482	118.860	193.297	523.117	134.394
AMK58_02190	K07157 uncharacterized protein | (GenBank) peptidase S16	Peptidase	119.671	115.318	124.796	53.827	235.895	58.087
AMK58_02195	K09791 uncharacterized protein | (GenBank) hypothetical protein	Hypothetical	566.037	604.322	562.966	143.709	1725.570	380.864
AMK58_02200	no KO assigned | (GenBank) hypothetical protein	Hypothetical	68.759	60.726	77.883	93.707	211.364	115.042
AMK58_02205	no KO assigned | (GenBank) hypothetical protein	Hypothetical	86.392	99.838	87.838	199.776	323.576	235.357
AMK58_02210	K06995 uncharacterized protein | (GenBank) cupin	No COG	134.980	127.651	138.336	298.022	528.743	472.036
AMK58_02215	K07025 putative hydrolase of the HAD superfamily | (GenBank) haloacid dehalogenase	Hydrolase	282.332	490.660	316.535	104.583	1701.066	170.284
AMK58_02220	no KO assigned | (GenBank) hypothetical protein	Hypothetical	755.124	552.035	496.324	845.785	509.753	1283.715
AMK58_02225	K01581 ornithine decarboxylase [EC:4.1.1.17] | (GenBank) ornithine decarboxylase	Metabolism: Amino Acid	7.561	8.907	10.024	103.987	10.773	39.398
AMK58_02230	K07507 putative Mg2+ transporter-C (MgtC) family protein | (GenBank) hypothetical protein	Transport: Ion	115.966	109.877	127.991	411.624	73.215	335.151
AMK58_02235	K07552 MFS transporter, DHA1 family, bicyclomycin/chloramphenicol resistance protein | (GenBank) MI	Transport: MFS	11.546	17.702	10.492	115.925	12.748	106.869
AMK58_02240	K07043 uncharacterized protein | (GenBank) hypothetical protein	Hypothetical	231.947	299.381	302.134	248.605	204.899	202.837
AMK58_02245	no KO assigned | (GenBank) hypothetical protein	Hypothetical	9.528	9.594	11.400	77.436	26.834	57.971
AMK58_02250	no KO assigned | (GenBank) hypothetical protein	Hypothetical	3082.536	3745.651	3890.888	833.766	503.701	951.389
AMK58_02255	K05813 sn-glycerol 3-phosphate transport system substrate-binding protein | (GenBank) glycerol-3-pho	Membrane Transport	236.811	240.037	268.869	56.001	38.346	146.672
AMK58_02260	K05814 sn-glycerol 3-phosphate transport system permease protein | (GenBank) glycerol-3-phosphate tr	Membrane Transport	187.563	198.616	208.385	57.177	39.675	136.880
AMK58_02265	K05815 sn-glycerol 3-phosphate transport system permease protein | (GenBank) glycerol-3-phosphate tr	Membrane Transport	111.604	134.051	151.552	290.217	24.893	172.390
AMK58_02270	K05816 sn-glycerol 3-phosphate transport system ATP-binding protein [EC:3.6.3.20] | (GenBank) glycer	Membrane Transport	80.942	90.251	91.645	108.275	23.316	112.583
AMK58_02275	K00799 glutathione S-transferase [EC:2.5.1.18] | (GenBank) glutathione S-transferase	Metabolism: Amino Acid	149.246	153.982	146.083	59.682	89.157	214.350
AMK58_02280	K06991 uncharacterized protein | (GenBank) glyoxalase	enzyme	50.935	46.561	48.165	111.479	128.585	588.706
AMK58_02285	no KO assigned | (GenBank) hypothetical protein	Hypothetical	10.762	17.223	16.811	182.009	47.093	124.744
AMK58_02290	no KO assigned | (GenBank) hypothetical protein	Hypothetical	231.941	199.987	267.708	454.438	586.268	372.911
AMK58_02295	K11927 ATP-dependent RNA helicase RhlE [EC:3.6.4.13] | (GenBank) DEAD/DEAH box helicase	Genetic Information Processing	86.362	155.041	90.685	43.114	23.771	84.691
AMK58_02300	K03711 Fur family transcriptional regulator, ferric uptake regulator | (GenBank) Fur family transcri	Transcription	68.378	113.648	80.901	109.157	19.070	68.411
AMK58_02305	no KO assigned | (GenBank) hypothetical protein	Hypothetical	209.325	318.276	271.169	1181.434	322.995	1093.257
AMK58_02310	no KO assigned | (GenBank) hypothetical protein	Hypothetical	5.778	8.631	11.246	416.047	53.280	232.493
AMK58_02315	no KO assigned | (GenBank) histidine kinase	Signal Transduction	9.773	10.993	9.797	86.981	23.578	165.769
AMK58_02320	no KO assigned | (GenBank) PAS domain-containing sensor histidine kinase	Signal Transduction	20.930	17.024	15.911	23.179	4.193	46.215
AMK58_02325	no KO assigned | (GenBank) anti-anti-sigma factor	Transcription	38.380	59.098	55.302	420.835	60.041	250.956
AMK58_02330	no KO assigned | (GenBank) hypothetical protein	Hypothetical	19.174	12.010	22.200	234.890	55.967	256.227
AMK58_02335	K07315 phosphoserine phosphatase RsbU/P [EC:3.1.3.3] | (GenBank) transcriptional regulator	Transcription	306.974	288.593	319.211	90.757	673.062	171.604
AMK58_02340	no KO assigned | (GenBank) hypothetical protein	Hypothetical	28.801	43.840	53.722	197.818	371.576	163.301

AMK58_02345	no KO assigned | (GenBank) hypothetical protein	Hypothetical	50.590	50.941	74.584	223.862	364.513	188.961
AMK58_02350	no KO assigned | (GenBank) twitching motility protein PilT	Motility	82.259	130.542	102.165	377.745	277.676	379.000
AMK58_02355	K07315 phosphoserine phosphatase RsbU/P [EC:3.1.3.3] | (GenBank) transcriptional regulator	Transcription	22.376	27.469	25.550	172.451	43.412	104.880
AMK58_02360	K07315 phosphoserine phosphatase RsbU/P [EC:3.1.3.3] | (GenBank) regulator	Transcription	28.363	29.760	36.247	141.487	40.565	87.289
AMK58_02365	no KO assigned | (GenBank) hypothetical protein	Hypothetical	112.380	99.976	82.362	75.159	42.045	83.670
AMK58_02370	no KO assigned | (GenBank) anti-anti-sigma factor	Transcription	216.431	217.479	258.830	409.175	234.827	555.111
AMK58_02375	K03406 methyl-accepting chemotaxis protein | (GenBank) chemotaxis protein	Chemotaxis	30.502	39.670	34.526	178.307	34.402	129.490
AMK58_02380	K03412 two-component system, chemotaxis family, response regulator CheB [EC:3.1.1.61] | (GenBank) cl	Chemotaxis	20.463	14.943	19.565	111.627	19.296	90.659
AMK58_02385	K03411 chemotaxis protein CheD [EC:3.5.1.44] | (GenBank) chemotaxis protein	Chemotaxis	66.813	49.608	57.651	295.659	46.884	289.179
AMK58_02390	K00575 chemotaxis protein methyltransferase CheR [EC:2.1.1.80] | (GenBank) chemotaxis protein	Chemotaxis	132.380	149.226	149.367	595.210	82.602	408.852
AMK58_02395	K03406 methyl-accepting chemotaxis protein | (GenBank) chemotaxis protein	Chemotaxis	30.186	32.355	27.448	35.831	5.929	46.462
AMK58_02400	K03408 purine-binding chemotaxis protein CheW | (GenBank) chemotaxis protein CheW	Chemotaxis	387.439	379.944	404.428	802.738	185.857	730.547
AMK58_02405	K03407 two-component system, chemotaxis family, sensor kinase CheA [EC:2.7.13.3] | (GenBank) chemo	Chemotaxis	20.558	19.393	18.621	16.813	7.054	25.785
AMK58_02410	K03413 two-component system, chemotaxis family, response regulator CheY | (GenBank) Fis family trans	Chemotaxis	65.169	79.055	77.060	220.236	49.826	92.331
AMK58_02415	no KO assigned | (GenBank) hypothetical protein	Hypothetical	288.329	329.220	379.302	1612.502	663.018	1234.966
AMK58_02420	no KO assigned | (GenBank) histidine kinase	Signal Transduction	2.040	1.815	2.888	17.207	5.268	6.237
AMK58_02425	no KO assigned | (GenBank) hypothetical protein	Hypothetical	1337.514	1465.259	1608.221	1534.528	1138.763	1113.101
AMK58_02430	K02342 DNA polymerase III subunit epsilon [EC:2.7.7.7] | (GenBank) exonuclease	DNA Repair and Replication	69.914	67.487	67.101	37.168	43.947	39.915
AMK58_02435	no KO assigned | (GenBank) two-component system response regulator	Signal Transduction	98.137	119.692	109.874	422.772	197.776	252.809
AMK58_02440	no KO assigned | (GenBank) hypothetical protein	Hypothetical	74.407	72.141	77.677	162.040	223.398	194.076
AMK58_02445	no KO assigned | (GenBank) allantoinase	Metabolism: Carbohydrate	74.122	63.038	62.576	74.733	240.609	130.737
AMK58_02450	K06190 intracellular septation protein | (GenBank) septation protein A	Cell Cycle/Shape/Homeostatsis	80.505	83.318	92.015	312.201	595.675	438.106
AMK58_02455	no KO assigned | (GenBank) hypothetical protein	Hypothetical	222.525	202.036	224.124	251.893	394.197	266.795
AMK58_02460	K01241 AMP nucleosidase [EC:3.2.2.4] | (GenBank) AMP nucleosidase	Metabolism: Nucleotide	38.696	41.132	42.963	212.114	74.006	119.461
AMK58_02465	K03110 fused signal recognition particle receptor | (GenBank) signal recognition particle-docking pr	Secretion System	101.917	115.981	102.733	337.374	338.654	205.354
AMK58_02470	K18707 threonylcarbamoyladenosine tRNA methylthiotransferase MtaB [EC:2.8.4.5] | (GenBank) 2-methy	tRNA synthesis/modification	63.708	86.114	75.193	159.097	239.135	82.242
AMK58_02475	K01778 diaminopimelate epimerase [EC:5.1.1.7] | (GenBank) diaminopimelate epimerase	Biosynthesis: Amino Acid	0.579	1.728	4.401	88.941	76.746	77.395
AMK58_02480	no KO assigned | (GenBank) hypothetical protein	Hypothetical	32.671	45.927	43.489	269.795	89.865	224.896
AMK58_02485	no KO assigned | (GenBank) GtrA-like protein	Biosynthesis: Polysaccharide	249.665	164.626	229.444	255.023	339.138	443.401
AMK58_02490	no KO assigned | (GenBank) glycosyl transferase	Transferase	25.972	19.016	23.726	271.163	56.097	281.087
AMK58_02495	no KO assigned | (GenBank) hypothetical protein	Hypothetical	42.439	34.636	36.112	30.665	24.061	75.329
AMK58_02500	K01704 3-isopropylmalate/(R)-2-methylmalate dehydratase small subunit [EC:4.2.1.33 4.2.1.35] | (GenB	Metabolism	416.046	347.494	412.133	352.463	418.509	353.319
AMK58_02505	K00052 3-isopropylmalate dehydrogenase [EC:1.1.1.85] | (GenBank) 3-isopropylmalate dehydrogenase	Metabolism	3.255	2.593	8.562	130.359	486.570	96.576
AMK58_02510	no KO assigned | (GenBank) hypothetical protein	Hypothetical	16.596	29.295	32.994	322.856	481.626	251.820
AMK58_02515	no KO assigned | (GenBank) hypothetical protein	Hypothetical	94.714	85.019	98.040	411.558	196.142	324.911
AMK58_02520	K01644 citrate lyase subunit beta / citryl-CoA lyase [EC:4.1.3.34] | (GenBank) malyl-CoA thiolestera	Signal Transduction	168.571	186.990	147.880	171.196	302.584	111.251
AMK58_02525	K00868 pyridoxine kinase [EC:2.7.1.35] | (GenBank) pyridoxamine kinase	Signal Transduction	97.591	82.333	96.133	148.058	140.361	107.184
AMK58_02530	K06966 uncharacterized protein | (GenBank) LOG family protein	No COG	25.302	22.488	26.046	136.135	91.199	109.361
AMK58_02535	no KO assigned | (GenBank) hypothetical protein	Hypothetical	199.159	172.976	194.924	436.413	277.446	471.587
AMK58_02540	K06916 cell division protein ZapE | (GenBank) ATPase	Cell Cycle/Shape/Homeostatsis	38.576	31.940	32.791	138.736	67.870	260.407
AMK58_02545	K13069 diguanylate cyclase [EC:2.7.7.65] | (GenBank) diguanylate cyclase	cyclin nucleotide	173.090	174.639	211.261	263.212	72.960	292.098
AMK58_02550	no KO assigned | (GenBank) PAS domain-containing sensor histidine kinase	Signal Transduction	678.062	538.573	538.195	42.297	124.404	68.851
AMK58_02555	K00658 2-oxoglutarate dehydrogenase E2 component (dihydrolipoamide succinyltransferase) [EC:2.3.1.61	Metabolism	2002.562	1525.738	1594.957	286.089	517.784	424.637
AMK58_02560	K00164 2-oxoglutarate dehydrogenase E1 component [EC:1.2.4.2] | (GenBank) sucA; 2-oxoglutarate dehy	Metabolism	155.307	136.757	144.479	25.783	19.774	12.091
AMK58_02565	K01902 succinyl-CoA synthetase alpha subunit [EC:6.2.1.5] | (GenBank) succinate--CoA ligase	Metabolism: Carbohydrate	2969.926	2128.779	2218.799	146.819	334.640	199.420
AMK58_02570	K01903 succinyl-CoA synthetase beta subunit [EC:6.2.1.5] | (GenBank) sucC; succinyl-CoA synthetase s	Metabolism: Carbohydrate	2150.923	1723.484	1703.703	79.264	360.073	174.454
AMK58_02575	K00024 malate dehydrogenase [EC:1.1.1.37] | (GenBank) malate dehydrogenase	Metabolism: Carbon	273.886	294.866	250.078	187.125	106.459	140.656
AMK58_02580	no KO assigned | (GenBank) hypothetical protein	Hypothetical	159.290	128.028	179.640	644.916	401.856	127.173
AMK58_02585	no KO assigned | (GenBank) hypothetical protein	Hypothetical	193.720	178.192	202.722	188.953	291.328	342.468
AMK58_02590	K04566 lysyl-tRNA synthetase, class I [EC:6.1.1.6] | (GenBank) lysK; lysine--tRNA ligase	tRNA synthesis/modification	3.473	3.688	4.549	28.116	15.613	55.312
AMK58_02595	K03297 small multidrug resistance pump | (GenBank) ligand-binding protein SH3	Transport	33.258	49.117	32.333	375.430	152.475	607.434
AMK58_02600	no KO assigned | (GenBank) hypothetical protein	Hypothetical	5.701	11.653	13.980	243.781	43.341	145.102
AMK58_02605	K03814 monofunctional biosynthetic peptidoglycan transglycosylase [EC:2.4.1.-] | (GenBank) monofunct	Cell Wall	97.812	118.980	109.757	716.026	203.446	251.013
AMK58_02610	K03722 ATP-dependent DNA helicase DinG [EC:3.6.4.12] | (GenBank) helicase	DNA Repair and Replication	37.123	19.331	26.233	91.548	20.298	82.753
AMK58_02615	K03406 methyl-accepting chemotaxis protein | (GenBank) chemotaxis protein	Chemotaxis	7.063	6.459	6.236	62.461	13.204	26.664
AMK58_02620	no KO assigned | (GenBank) hypothetical protein	Hypothetical	60.285	55.759	57.559	276.392	253.474	456.096
AMK58_02625	K00651 homoserine O-succinyltransferase [EC:2.3.1.46] | (GenBank) homoserine O-succinyltransferase	Metabolism: Amino Acid	3.321	3.815	3.157	33.703	15.361	107.888
AMK58_02630	no KO assigned | (GenBank) hypothetical protein	Hypothetical	223.336	212.112	241.202	756.867	257.520	847.509
AMK58_02635	K03406 methyl-accepting chemotaxis protein | (GenBank) chemotaxis protein	Chemotaxis	78.355	92.343	75.589	96.526	67.403	143.854
AMK58_02640	K07088 uncharacterized protein | (GenBank) transporter	Transport	269.098	293.005	247.603	34.559	281.374	55.780
AMK58_02645	no KO assigned | (GenBank) hypothetical protein	Hypothetical	323.165	400.078	393.678	485.356	576.632	181.018
AMK58_02650	no KO assigned | (GenBank) 2-hydroxy-3-oxopropionate reductase	Enzyme	58.265	71.595	67.447	96.131	142.441	104.606
AMK58_02655	K03827 putative acetyltransferase [EC:2.3.1.-] | (GenBank) GNAT family acetyltransferase	Acetyltransferase	108.139	91.192	117.333	244.665	409.734	365.836
AMK58_02660	K00133 aspartate-semialdehyde dehydrogenase [EC:1.2.1.11] | (GenBank) aspartate-semialdehyde dehydro	Biosynthesis: Amino Acid	35.135	44.678	40.404	31.801	58.840	85.413
AMK58_02665	no KO assigned | (GenBank) hypothetical protein	Hypothetical	907.602	756.235	671.963	192.903	401.063	368.279
AMK58_02670	K00241 succinate dehydrogenase / fumarate reductase, cytochrome b subunit | (GenBank) succinate dehy	Energy	412.586	315.309	311.411	147.631	165.406	222.236
AMK58_02675	K00242 succinate dehydrogenase / fumarate reductase, membrane anchor subunit | (GenBank) succinate d	Energy	1802.058	1512.471	1446.254	555.773	3993.785	843.157

AMK58_02680	K00239 succinate dehydrogenase / fumarate reductase, flavoprotein subunit [EC:1.3.5.1 1.3.5.4] | (Ge	Energy	167.684	133.549	128.491	53.334	687.034	131.170
AMK58_02685	K00240 succinate dehydrogenase / fumarate reductase, iron-sulfur subunit [EC:1.3.5.1 1.3.5.4] | (Gen	Energy	1.091	0.000	2.519	114.803	62.179	54.184
AMK58_02690	no KO assigned | (GenBank) hypothetical protein	Hypothetical	1283.749	1301.331	1306.939	933.364	473.658	669.215
AMK58_02695	K00826 branched-chain amino acid aminotransferase [EC:2.6.1.42] | (GenBank) aminotransferase	Biosynthesis: Amino Acid	182.682	236.006	186.441	152.252	131.284	178.521
AMK58_02700	no KO assigned | (GenBank) hypothetical protein	Hypothetical	33.020	36.533	38.761	54.062	46.281	75.224
AMK58_02705	no KO assigned | (GenBank) hypothetical protein	Hypothetical	384.466	403.979	409.648	454.310	193.396	438.902
AMK58_02710	no KO assigned | (GenBank) DNA-binding protein	No COG	1.339	2.285	2.364	117.860	21.715	105.885
AMK58_02715	no KO assigned | (GenBank) hypothetical protein	Hypothetical	2.316	3.953	11.323	345.642	66.187	179.141
AMK58_02720	no KO assigned | (GenBank) hypothetical protein	Hypothetical	5.226	3.469	11.043	234.528	57.440	45.231
AMK58_02725	no KO assigned | (GenBank) hypothetical protein	Hypothetical	20.179	31.846	33.305	632.933	129.246	353.755
AMK58_02730	no KO assigned | (GenBank) pseudogene	Pseudogene	12.315	10.638	13.967	23.453	31.355	97.273
AMK58_02735	no KO assigned | (GenBank) hypothetical protein	Hypothetical	23.147	10.638	12.415	209.888	106.740	378.635
AMK58_02740	no KO assigned | (GenBank) pseudogene	Pseudogene	26.906	23.442	21.851	126.145	101.440	300.425
AMK58_02745	no KO assigned | (GenBank) hypothetical protein	Hypothetical	40.833	53.897	44.242	137.381	145.166	362.785
AMK58_02750	K01173 endonuclease G, mitochondrial | (GenBank) endonuclease	DNA Repair and Replication	40.205	48.590	49.462	217.892	105.218	369.110
AMK58_02755	no KO assigned | (GenBank) hypothetical protein	Hypothetical	215.043	291.501	257.156	152.503	132.031	549.619
AMK58_02760	no KO assigned | (GenBank) hypothetical protein	Hypothetical	87.490	104.541	97.677	124.380	91.363	306.190
AMK58_02765	no KO assigned | (GenBank) hypothetical protein	Hypothetical	716.701	702.411	853.392	87.965	40.367	273.856
AMK58_02770	no KO assigned | (GenBank) hypothetical protein	Hypothetical	420.879	344.455	505.016	181.976	32.403	180.433
AMK58_02775	K16263 amino acid efflux transporter | (GenBank) hypothetical protein	Transport	250.758	256.050	358.559	258.530	29.133	135.015
AMK58_02780	no KO assigned | (GenBank) hypothetical protein	Hypothetical	179.460	159.138	226.912	164.348	62.603	252.233
AMK58_02785	no KO assigned | (GenBank) hypothetical protein	Hypothetical	132.961	135.423	189.499	203.288	94.457	289.133
AMK58_02790	no KO assigned | (GenBank) 3'-5' exonuclease	DNA Repair and Replication	32.287	50.854	48.978	196.196	121.577	237.603
AMK58_02795	K00449 protocatechuate 3,4-dioxygenase, beta subunit [EC:1.13.11.3] | (GenBank) hypothetical protein	Metabolism	28.269	30.001	42.971	225.715	277.831	263.170
AMK58_02800	no KO assigned | (GenBank) hypothetical protein	Hypothetical	5.444	6.729	14.635	303.250	40.089	163.270
AMK58_02805	K03707 thiaminase (transcriptional activator TenA) [EC:3.5.99.2] | (GenBank) thiaminase II	Transcription	53.084	55.392	58.424	101.305	76.833	222.854
AMK58_02810	no KO assigned | (GenBank) hypothetical protein	Hypothetical	41.367	28.130	37.733	84.697	76.741	311.548
AMK58_02815	no KO assigned | (GenBank) hypothetical protein	Hypothetical	111.297	150.388	112.350	157.727	89.560	297.071
AMK58_02820	no KO assigned | (GenBank) hypothetical protein	Hypothetical	1677.382	1741.754	1857.473	107.951	133.339	315.407
AMK58_02825	no KO assigned | (GenBank) hypothetical protein	Hypothetical	289.124	315.430	293.899	208.980	63.808	449.447
AMK58_02830	no KO assigned | (GenBank) transposase	Genetic Information Processing	12.273	16.921	17.728	147.959	82.505	266.777
AMK58_02835	no KO assigned | (GenBank) transposase	Genetic Information Processing	86.882	107.164	100.196	54.062	99.235	265.341
AMK58_02840	no KO assigned | (GenBank) hypothetical protein	Hypothetical	333.917	334.408	304.928	126.788	146.624	323.444
AMK58_02845	no KO assigned | (GenBank) transposase	Genetic Information Processing	131.355	154.680	129.961	203.497	155.352	378.116
AMK58_02850	no KO assigned | (GenBank) transposase	Genetic Information Processing	0.992	1.185	1.320	4.383	10.366	22.987
AMK58_02855	no KO assigned | (GenBank) hypothetical protein	Hypothetical	3.586	6.476	10.228	194.578	47.426	225.003
AMK58_02860	no KO assigned | (GenBank) ATP-dependent endonuclease	DNA Repair and Replication	41.134	46.060	34.142	44.844	15.994	303.176
AMK58_02865	K03657 DNA helicase II / ATP-dependent DNA helicase PcrA [EC:3.6.4.12] | (GenBank) Fis family trans	DNA Repair and Replication	25.154	27.324	26.268	66.563	29.317	371.138
AMK58_02870	no KO assigned | (GenBank) hypothetical protein	Hypothetical	72.842	56.849	65.827	94.938	79.295	323.118
AMK58_02875	K02342 DNA polymerase III subunit epsilon [EC:2.7.7.7] | (GenBank) DNA polymerase III subunit epsilo	DNA Repair and Replication	30.795	17.141	33.826	52.012	32.250	195.629
AMK58_02880	no KO assigned | (GenBank) AraC family transcriptional regulator	Transcription	3.143	2.253	5.140	164.881	22.188	135.237
AMK58_02885	no KO assigned | (GenBank) MFS transporter	Transport: MFS	4.259	10.905	8.870	234.178	22.340	133.332
AMK58_02890	no KO assigned | (GenBank) hypothetical protein	Hypothetical	8.072	4.822	14.198	94.608	51.715	405.294
AMK58_02895	no KO assigned | (GenBank) hypothetical protein	Hypothetical	63.385	56.212	58.148	77.279	23.656	291.121
AMK58_02900	no KO assigned | (GenBank) hypothetical protein	Hypothetical	103.673	105.443	105.614	84.647	32.185	326.967
AMK58_02905	no KO assigned | (GenBank) hypothetical protein	Hypothetical	99.889	99.899	106.545	99.446	77.247	349.229
AMK58_02910	no KO assigned | (GenBank) hypothetical protein	Hypothetical	128.797	141.015	128.418	108.514	58.466	301.412
AMK58_02915	no KO assigned | (GenBank) transposase	Genetic Information Processing	0.542	1.618	1.803	62.286	5.886	27.377
AMK58_02920	no KO assigned | (GenBank) transposase	Genetic Information Processing	6.331	12.833	7.954	108.124	34.563	201.058
AMK58_02925	no KO assigned | (GenBank) transposase	Genetic Information Processing	11.182	14.590	13.565	176.132	19.070	224.063
AMK58_02930	no KO assigned | (GenBank) pseudogene	Pseudogene	0.000	3.039	2.902	144.468	8.959	81.451
AMK58_02935	no KO assigned | (GenBank) hypothetical protein	Hypothetical	0.000	0.000	1.265	128.323	5.484	34.956
AMK58_02940	no KO assigned | (GenBank) hypothetical protein	Hypothetical	0.684	6.130	4.390	247.402	14.417	116.868
AMK58_02945	no KO assigned | (GenBank) diguanylate cyclase	cyclic nucleotide	142.914	202.378	179.189	199.362	35.452	174.177
AMK58_02950	no KO assigned | (GenBank) hypothetical protein	Hypothetical	25.431	11.560	47.046	232.170	215.326	394.225
AMK58_02955	no KO assigned | (GenBank) hypothetical protein	Hypothetical	1.183	1.766	4.357	87.925	17.448	221.661
AMK58_02960	no KO assigned | (GenBank) hypothetical protein	Hypothetical	2.242	2.679	5.116	388.645	26.211	271.942
AMK58_02965	no KO assigned | (GenBank) hypothetical protein	Hypothetical	0.637	0.000	2.626	149.666	7.879	86.073
AMK58_02970	no KO assigned | (GenBank) hypothetical protein	Hypothetical	0.255	0.382	3.339	197.685	7.034	112.684
AMK58_02975	no KO assigned | (GenBank) hypothetical protein	Hypothetical	0.364	1.086	2.593	154.880	4.700	70.464
AMK58_02980	no KO assigned | (GenBank) hypothetical protein	Hypothetical	0.537	0.712	3.117	205.467	13.134	167.879
AMK58_02985	no KO assigned | (GenBank) hypothetical protein	Hypothetical	0.942	2.815	2.314	206.887	9.532	100.184
AMK58_02990	no KO assigned | (GenBank) hypothetical protein	Hypothetical	0.351	0.000	1.335	357.279	9.073	69.314
AMK58_02995	no KO assigned | (GenBank) hydrolase	Hydrolase	3.259	4.493	6.912	342.840	20.287	118.313
AMK58_03000	no KO assigned | (GenBank) hypothetical protein	Hypothetical	4.376	2.905	7.397	337.399	44.271	122.189
AMK58_03005	no KO assigned | (GenBank) glutathione S-transferase	Transferase	38.116	30.618	35.632	137.300	42.484	65.342
AMK58_03010	K00254 dihydroorotate dehydrogenase [EC:1.3.5.2] | (GenBank) dihydroorotate dehydrogenase (quinone)	Metabolism: Nucleotide	74.854	74.087	82.427	263.816	65.766	107.984

AMK58_03015	K13593 cyclic-di-GMP phosphodiesterase, flagellum assembly factor TipF | (GenBank) diguanylate phosp	Motility	19.380	17.308	24.031	175.701	31.553	147.655
AMK58_03020	K01868 threonyl-tRNA synthetase [EC:6.1.1.3] | (GenBank) threonine--tRNA ligase	tRNA synthesis/modification	202.469	169.703	150.504	114.975	178.202	232.138
AMK58_03025	K02520 translation initiation factor IF-3 | (GenBank) translation initiation factor IF-3	Translation	855.779	963.104	891.300	128.090	492.307	278.760
AMK58_03030	K05366 penicillin-binding protein 1A [EC:2.4.1.- 3.4.-.-] | (GenBank) hypothetical protein	Defense	3.418	5.891	7.000	193.003	63.096	196.990
AMK58_03035	no KO assigned | (GenBank) hypothetical protein	Hypothetical	3.243	1.076	2.570	187.769	17.619	35.920
AMK58_03040	no KO assigned | (GenBank) hypothetical protein	Hypothetical	14.056	20.358	18.225	160.759	30.642	119.577
AMK58_03045	no KO assigned | (GenBank) hypothetical protein	Hypothetical	100.129	152.607	104.919	182.031	35.028	391.760
AMK58_03050	no KO assigned | (GenBank) HAD family hydrolase	Hydrolase	35.106	43.765	45.995	189.772	34.179	107.400
AMK58_03055	no KO assigned | (GenBank) glycosyl transferase	Transferase	42.893	44.846	52.925	180.076	42.406	139.930
AMK58_03060	no KO assigned | (GenBank) alpha/beta hydrolase	Hydrolase	134.958	173.605	141.673	245.009	88.733	196.996
AMK58_03065	K14233 tRNA Ser | (GenBank) tRNA-Ser	tRNA synthesis/modification	142.363	182.826	156.865	185.355	116.651	666.216
AMK58_03070	no KO assigned | (GenBank) hypothetical protein	Hypothetical	60.537	42.779	55.703	207.528	57.071	137.621
AMK58_03075	no KO assigned | (GenBank) peptidase	Peptidase	85.888	87.845	76.650	169.113	29.293	217.863
AMK58_03080	K02014 iron complex outermembrane recepter protein | (GenBank) TonB-dependent receptor	Transport: Metal	75.888	68.327	60.604	159.394	25.139	147.666
AMK58_03085	K07165 transmembrane sensor | (GenBank) hypothetical protein	Membrane	22.779	16.272	22.482	160.030	15.724	119.043
AMK58_03090	K03088 RNA polymerase sigma-70 factor, ECF subfamily | (GenBank) hypothetical protein	Transcription	99.627	139.160	122.794	231.385	39.921	182.625
AMK58_03095	K01130 arylsulfatase [EC:3.1.6.1] | (GenBank) arylsulfatase	Metabolism: Lipid	14.827	11.667	13.685	90.200	36.422	241.373
AMK58_03100	no KO assigned | (GenBank) hypothetical protein	Hypothetical	1.156	2.302	4.030	201.668	22.628	124.685
AMK58_03105	K07114 Ca-activated chloride channel homolog | (GenBank) hypothetical protein	Transport: Ion	0.188	1.121	1.249	240.639	10.339	57.962
AMK58_03110	K07114 Ca-activated chloride channel homolog | (GenBank) hypothetical protein	Transport: Ion	1.059	2.372	2.642	192.851	17.105	111.288
AMK58_03115	K07114 Ca-activated chloride channel homolog | (GenBank) hypothetical protein	Transport: Ion	0.752	0.000	4.171	245.965	29.304	158.409
AMK58_03120	no KO assigned | (GenBank) hypothetical protein	Hypothetical	0.771	0.000	3.911	247.927	41.030	99.398
AMK58_03125	no KO assigned | (GenBank) MoxR protein	No COG	1.886	5.258	3.945	163.702	55.257	123.345
AMK58_03130	K03924 MoxR-like ATPase [EC:3.6.3.-] | (GenBank) AAA family ATPase	No COG	0.623	0.744	1.184	74.919	16.382	100.719
AMK58_03135	no KO assigned | (GenBank) haloacid dehalogenase	enzyme	0.958	2.862	3.644	158.657	25.173	216.988
AMK58_03140	no KO assigned | (GenBank) gliding motility-associated lipoprotein GldK	Motility	0.789	4.712	3.750	119.394	32.656	258.915
AMK58_03145	no KO assigned | (GenBank) hypothetical protein	Hypothetical	0.984	0.000	1.872	65.929	38.726	202.633
AMK58_03150	no KO assigned | (GenBank) hypothetical protein	Hypothetical	13.499	19.223	17.890	76.455	56.922	237.122
AMK58_03155	no KO assigned | (GenBank) hypothetical protein	Hypothetical	37.017	45.509	50.063	168.093	73.154	177.327
AMK58_03160	no KO assigned | (GenBank) CoA-transferase	Metabolism	45.525	54.164	49.681	210.502	34.626	98.711
AMK58_03165	no KO assigned | (GenBank) enoyl-CoA hydratase	Metabolism	11.816	13.752	13.569	114.291	9.314	24.048
AMK58_03170	no KO assigned | (GenBank) hypothetical protein	Hypothetical	41.779	54.337	50.263	161.424	13.737	72.211
AMK58_03175	K01426 amidase [EC:3.5.1.4] | (GenBank) hypothetical protein	Metabolism: Carbohydrate	22.261	26.791	27.638	136.549	11.341	45.220
AMK58_03180	no KO assigned | (GenBank) short-chain dehydrogenase	enzyme	33.091	50.810	41.755	161.564	10.429	62.372
AMK58_03185	no KO assigned | (GenBank) hypothetical protein	Hypothetical	136.597	140.343	132.928	169.394	40.712	229.241
AMK58_03190	K01997 branched-chain amino acid transport system permease protein | (GenBank) hypothetical protein	Transport: Amino Acid	77.411	82.293	89.712	164.585	34.836	152.631
AMK58_03195	K01998 branched-chain amino acid transport system permease protein | (GenBank) hypothetical protein	Transport: Amino Acid	34.710	27.416	36.247	172.431	12.697	99.613
AMK58_03200	K01995 branched-chain amino acid transport system ATP-binding protein | (GenBank) livG; ABC transpor	Transport: Amino Acid	25.612	25.967	34.978	171.959	16.052	97.876
AMK58_03205	K01996 branched-chain amino acid transport system ATP-binding protein | (GenBank) ABC transporter A	Membrane Transport	34.084	30.393	34.665	171.953	24.588	109.282
AMK58_03210	no KO assigned | (GenBank) hypothetical protein	Hypothetical	285.181	258.683	209.654	111.704	43.862	169.520
AMK58_03215	no KO assigned | (GenBank) hypothetical protein	Hypothetical	254.148	242.049	185.050	169.909	21.018	94.642
AMK58_03220	no KO assigned | (GenBank) hypothetical protein	Hypothetical	40.511	40.490	40.907	272.554	13.096	135.288
AMK58_03225	K02004 putative ABC transport system permease protein | (GenBank) hypothetical protein	Membrane Transport	15.679	16.768	21.993	107.994	9.573	40.385
AMK58_03230	K02003 putative ABC transport system ATP-binding protein | (GenBank) hypothetical protein	Membrane Transport	55.210	73.300	66.309	199.164	15.242	80.235
AMK58_03235	no KO assigned | (GenBank) hypothetical protein	Hypothetical	133.913	109.733	114.769	214.191	21.129	178.287
AMK58_03240	no KO assigned | (GenBank) hypothetical protein	Hypothetical	94.095	91.252	89.996	143.966	18.672	84.978
AMK58_03245	no KO assigned | (GenBank) virulence factor	Defense	76.843	64.585	63.427	150.782	14.176	99.573
AMK58_03250	no KO assigned | (GenBank) hypothetical protein	Hypothetical	85.781	84.536	78.198	168.271	15.950	171.871
AMK58_03255	no KO assigned | (GenBank) hypothetical protein	Hypothetical	57.238	57.336	57.111	203.622	18.878	90.022
AMK58_03260	no KO assigned | (GenBank) hypothetical protein	Hypothetical	70.403	75.015	75.840	195.900	24.005	85.034
AMK58_03265	no KO assigned | (GenBank) hypothetical protein	Hypothetical	89.022	104.135	80.639	202.145	64.825	139.822
AMK58_03270	no KO assigned | (GenBank) metallophosphoesterase	Hydrolase	30.503	32.243	42.389	165.958	183.304	242.381
AMK58_03275	no KO assigned | (GenBank) inositol monophosphatase	Phosphatase	271.914	244.133	236.473	117.585	669.692	259.156
AMK58_03280	no KO assigned | (GenBank) cytochrome B	Energy	110.643	101.147	108.452	283.596	75.351	164.282
AMK58_03285	no KO assigned | (GenBank) cytochrome C	Energy	972.991	1115.378	1180.687	206.318	126.527	278.510
AMK58_03290	K00812 aspartate aminotransferase [EC:2.6.1.1] | (GenBank) aspartate aminotransferase	Metabolism: Amino Acid	616.216	521.824	493.217	165.951	254.357	238.689
AMK58_03295	K03313 Na+:H+ antiporter, NhaA family | (GenBank) nhaA; sodium:proton antiporter	Transport: Ion	32.859	30.066	43.064	198.668	141.972	201.390
AMK58_03300	no KO assigned | (GenBank) hypothetical protein	Hypothetical	10.222	14.741	13.070	87.585	82.706	240.881
AMK58_03305	no KO assigned | (GenBank) hypothetical protein	Hypothetical	1989.326	2186.811	1920.248	1841.110	1056.612	1769.807
AMK58_03310	K02355 elongation factor G | (GenBank) elongation factor G	Translation	70.686	86.519	99.657	107.090	30.221	44.633
AMK58_03315	K00077 2-dehydropantoate 2-reductase [EC:1.1.1.169] | (GenBank) hypothetical protein	Biosynthesis: Co-Factors and Vitamins	16.117	27.400	17.691	115.847	46.101	111.176
AMK58_03320	no KO assigned | (GenBank) chemotaxis protein	Chemotaxis	1154.394	1367.318	1112.114	165.153	2202.663	496.999
AMK58_03325	K04047 starvation-inducible DNA-binding protein | (GenBank) DNA starvation/stationary phase protecti	Starvation	24.318	21.639	18.695	160.453	367.277	142.016
AMK58_03330	K15977 putative oxidoreductase | (GenBank) hypothetical protein	Oxidoreductase	295.784	264.913	256.998	346.779	1920.824	555.193
AMK58_03335	K00384 thioredoxin reductase (NADPH) [EC:1.8.1.9] | (GenBank) thioredoxin reductase	Energy	3.954	4.169	4.976	83.040	202.375	140.742
AMK58_03340	no KO assigned | (GenBank) hypothetical protein	Hypothetical	2.440	1.121	5.533	242.650	122.800	217.055
AMK58_03345	no KO assigned | (GenBank) hypothetical protein	Hypothetical	39.068	28.626	36.796	295.489	43.707	221.805

AMK58_03350	K07276 uncharacterized protein | (GenBank) hypothetical protein	Hypothetical	280.918	248.425	286.169	47.018	81.106	176.683
AMK58_03355	no KO assigned | (GenBank) ArsR family transcriptional regulator	Transcription	103.623	130.334	140.632	327.002	139.771	250.634
AMK58_03360	no KO assigned | (GenBank) dienelactone hydrolase	Hydrolase	1150.741	1135.002	1041.751	86.784	906.840	226.386
AMK58_03365	K03624 transcription elongation factor GreA | (GenBank) transcription elongation factor GreA	Transcription	1239.764	1170.922	1287.125	1329.642	1556.029	1321.064
AMK58_03370	K01955 carbamoyl-phosphate synthase large subunit [EC:6.3.5.5] | (GenBank) carbamoyl phosphate synth	Metabolism	11.467	12.011	14.655	43.988	9.793	19.333
AMK58_03375	no KO assigned | (GenBank) hypothetical protein	Hypothetical	10.052	7.171	11.127	91.443	28.669	86.254
AMK58_03380	no KO assigned | (GenBank) hypothetical protein	Hypothetical	65.884	55.643	88.553	254.137	137.256	399.821
AMK58_03385	K07507 putative Mg2+ transporter-C (MgtC) family protein | (GenBank) magnesium transporter MgtC	Transport: Ion	32.885	31.752	42.795	100.560	35.097	146.613
AMK58_03390	no KO assigned | (GenBank) diguanylate cyclase	cyclic nucleotide	405.797	408.725	400.810	576.221	215.481	819.660
AMK58_03395	K03466 DNA segregation ATPase FtsK/SpoIIIE, S-DNA-T family | (GenBank) cell division protein FtsK	Cell Cycle/Shape/Homeostatsis	23.771	20.588	25.878	32.625	9.848	52.858
AMK58_03400	no KO assigned | (GenBank) pseudogene	Pseudogene	166.614	123.818	147.010	233.781	481.230	615.207
AMK58_03405	no KO assigned | (GenBank) hypothetical protein	Hypothetical	59.423	63.277	68.160	238.948	391.111	295.147
AMK58_03410	no KO assigned | (GenBank) hypothetical protein	Hypothetical	88.832	102.213	85.401	157.926	360.820	114.202
AMK58_03415	K07304 peptide-methionine (S)-S-oxide reductase [EC:1.8.4.11] | (GenBank) methionine sulfoxide reduc	Oxidoreductase	15.302	14.856	20.550	120.166	114.593	84.956
AMK58_03420	no KO assigned | (GenBank) hypothetical protein	Hypothetical	25.022	20.897	51.420	413.754	231.358	123.996
AMK58_03425	K03214 RNA methyltransferase, TrmH family [EC:2.1.1.-] | (GenBank) RNA methyltransferase	tRNA synthesis/modification	8.798	10.012	15.137	308.658	201.393	148.786
AMK58_03430	no KO assigned | (GenBank) short-chain dehydrogenase	enzyme	66.567	62.485	60.484	67.083	133.327	133.605
AMK58_03435	no KO assigned | (GenBank) cytochrome B	Energy	19.447	14.992	20.081	205.043	71.220	77.367
AMK58_03440	no KO assigned | (GenBank) hypothetical protein	Hypothetical	83.177	91.155	116.523	448.319	184.777	355.064
AMK58_03445	K08738 cytochrome c | (GenBank) cytochrome C	Energy	40.043	47.093	47.966	255.105	72.300	217.230
AMK58_03450	K02019 molybdate transport system regulatory protein | (GenBank) LysR family transcriptional regulat	transcription	3173.762	2602.662	2870.637	839.948	507.681	1008.226
AMK58_03455	K00138 aldehyde dehydrogenase [EC:1.2.1.-] | (GenBank) aldehyde dehydrogenase	Metabolism: Carbohydrate	33.433	36.144	30.275	27.298	50.017	68.056
AMK58_03460	K09959 uncharacterized protein | (GenBank) acetaldehyde dehydrogenase	Metabolism: Carbohydrate	227.961	278.793	297.990	943.550	129.005	671.571
AMK58_03465	no KO assigned | (GenBank) FIST domain containing protein	No COG	105.916	151.132	143.945	464.053	61.752	390.795
AMK58_03470	K20975 two-component system, sensor histidine kinase [EC:2.7.13.3] | (GenBank) hypothetical protein	Signal Transduction	9.043	10.645	9.487	36.280	11.717	58.365
AMK58_03475	K01992 ABC-2 type transport system permease protein | (GenBank) multidrug ABC transporter permease	Membrane Transport	8.520	8.037	15.349	184.812	12.433	79.282
AMK58_03480	K01990 ABC-2 type transport system ATP-binding protein | (GenBank) ABC transporter ATP-binding pro	Membrane Transport	77.122	57.595	70.687	138.547	46.834	274.841
AMK58_03485	no KO assigned | (GenBank) hypothetical protein	Hypothetical	24.990	32.099	28.988	227.629	15.871	166.725
AMK58_03490	no KO assigned | (GenBank) branched-chain amino acid ABC transporter substrate-binding protein	Membrane Transport	7.649	9.019	8.708	74.015	5.430	68.500
AMK58_03495	no KO assigned | (GenBank) cytochrome c-550 PedF	Energy	17.725	13.033	19.964	373.685	34.943	252.607
AMK58_03500	no KO assigned | (GenBank) ABC transporter substrate-binding protein	Membrane Transport	3.094	2.520	7.354	190.441	10.590	114.356
AMK58_03505	K17226 sulfur-oxidizing protein SoxY | (GenBank) quinoprotein dehydrogenase-associated SoxYZ-like ca	Energy	8.313	9.031	12.360	202.277	20.728	244.162
AMK58_03510	no KO assigned | (GenBank) MBL fold metallo-hydrolase	Hydrolase	106.754	112.781	152.130	173.173	168.873	377.975
AMK58_03515	K00114 alcohol dehydrogenase (cytochrome c) [EC:1.1.2.8] | (GenBank) dehydrogenase	Metabolism: Carbohydrate	48.789	47.481	59.991	56.932	44.044	62.415
AMK58_03520	no KO assigned | (GenBank) two-component system response regulator	Signal Transduction	2.909	2.172	3.111	183.616	64.981	74.172
AMK58_03525	no KO assigned | (GenBank) hypothetical protein	Hypothetical	5.146	9.709	8.499	333.805	108.996	131.609
AMK58_03530	K02049 NitT/TauT family transport system ATP-binding protein | (GenBank) ABC transporter	Transport: NitT/TauT	14.304	15.261	16.191	154.202	117.526	153.009
AMK58_03535	K02050 NitT/TauT family transport system permease protein | (GenBank) ABC transporter permease	Transport: NitT/TauT	65.562	90.539	92.065	309.628	401.825	213.446
AMK58_03540	K02051 NitT/TauT family transport system substrate-binding protein | (GenBank) ABC transporter subst	Transport: NitT/TauT	13.246	17.751	21.188	212.931	26.718	106.530
AMK58_03545	no KO assigned | (GenBank) sulfurtransferase	Transferase	38.917	28.295	51.686	46.339	74.064	124.299
AMK58_03550	K08738 cytochrome c | (GenBank) cytochrome C	Energy	26.803	42.334	40.131	278.564	280.151	331.096
AMK58_03555	no KO assigned | (GenBank) cupin	No COG	10.701	12.787	24.128	59.796	58.080	170.673
AMK58_03560	K14228 tRNA Leu | (GenBank) tRNA-Leu	tRNA synthesis/modification	918.498	1164.020	1030.778	1472.722	1201.374	1045.269
AMK58_03565	no KO assigned | (GenBank) disulfide bond formation protein	Oxidoreductase	147.068	156.659	175.388	190.286	277.056	167.242
AMK58_03570	K06134 ubiquinone biosynthesis monooxygenase Coq7 [EC:1.14.13.-] | (GenBank) ubiquinone biosynthes	Biosynthesis: Co-Factors and Vitamins	832.389	834.325	852.273	192.289	4891.622	478.209
AMK58_03575	no KO assigned | (GenBank) MFS transporter	Transport: MFS	30.913	33.346	32.250	368.081	51.879	401.902
AMK58_03580	K01894 glutamyl-Q tRNA(Asp) synthetase [EC:6.1.1.-] | (GenBank) glutamyl-Q tRNA(Asp) synthetase	tRNA synthesis/modification	282.934	343.849	333.061	423.930	195.993	322.973
AMK58_03585	no KO assigned | (GenBank) hypothetical protein	Hypothetical	22.917	23.364	25.879	93.875	42.727	100.303
AMK58_03590	no KO assigned | (GenBank) DNA mismatch repair protein MutT	DNA Repair and Replication	25.527	46.008	28.339	84.436	36.227	137.659
AMK58_03595	K03413 two-component system, chemotaxis family, response regulator CheY | (GenBank) regulator	Chemotaxis	4305.606	5247.110	4185.016	426.674	4292.519	1346.372
AMK58_03600	K07180 serine protein kinase | (GenBank) serine/threonine protein kinase	Signal Transduction	435.111	553.198	481.998	85.550	550.035	188.961
AMK58_03605	K09786 uncharacterized protein | (GenBank) hypothetical protein	Hypothetical	623.633	671.887	588.950	122.800	598.157	346.968
AMK58_03610	no KO assigned | (GenBank) SpoVR family protein	No COG	65.009	71.546	81.330	141.144	154.274	103.761
AMK58_03615	K01768 adenylate cyclase [EC:4.6.1.1] | (GenBank) hypothetical protein	Metabolism: Nucleotide	9.034	4.693	7.376	60.507	27.042	37.970
AMK58_03620	no KO assigned | (GenBank) DNA-binding protein	No COG	25.224	27.400	36.629	788.199	210.326	322.137
AMK58_03625	K03579 ATP-dependent helicase HrpB [EC:3.6.4.13] | (GenBank) ATP-dependent helicase	Hydrolase	0.476	1.423	1.993	104.294	25.119	97.468
AMK58_03630	K02049 NitT/TauT family transport system ATP-binding protein | (GenBank) nitrate ABC transporter ATP	Transport: NitT/TauT	1.926	2.192	4.361	217.108	58.871	151.649
AMK58_03635	K02050 NitT/TauT family transport system permease protein | (GenBank) sulfonate ABC transporter perm	Transport: NitT/TauT	3.152	4.094	4.430	22.579	6.162	72.178
AMK58_03640	no KO assigned | (GenBank) rubrerythrin	Iron Homeostatsis	6.684	4.607	13.687	40.977	25.716	123.473
AMK58_03645	no KO assigned | (GenBank) hypothetical protein	Hypothetical	9.095	10.188	25.402	1126.164	170.596	654.643
AMK58_03650	K07315 phosphoserine phosphatase RsbU/P [EC:3.1.3.3] | (GenBank) regulator	Signal Transduction	3.748	6.315	6.213	17.506	24.302	57.471
AMK58_03655	K20978 HptB-dependent secretion and biofilm anti anti-sigma factor | (GenBank) anti-anti-sigma facto	Signal Transduction	7.122	26.003	21.820	470.657	310.880	364.693
AMK58_03660	no KO assigned | (GenBank) hypothetical protein	Hypothetical	115.272	187.537	153.116	60.069	79.758	117.039
AMK58_03665	no KO assigned | (GenBank) aldolase	Metabolism	423.617	575.909	592.711	243.473	112.921	204.347
AMK58_03670	K07340 inner membrane protein | (GenBank) hypothetical protein	Membrane	310.166	298.447	324.694	313.030	273.773	425.787
AMK58_03675	no KO assigned | (GenBank) hypothetical protein	Hypothetical	2.185	3.435	6.778	265.843	87.498	200.230
AMK58_03680	no KO assigned | (GenBank) hypothetical protein	Hypothetical	93.335	87.739	89.158	43.570	221.075	121.146

AMK58_03685	K05805 CreA protein | (GenBank) CREA protein	No COG	83.891	117.850	102.184	419.132	630.259	283.618
AMK58_03690	K07120 uncharacterized protein | (GenBank) ammonia monooxygenase	Nitrogen	88.925	133.501	123.682	132.101	167.874	153.922
AMK58_03695	no KO assigned | (GenBank) succinylglutamate desuccinylase	Metabolism	75.751	84.430	72.751	204.310	164.571	225.602
AMK58_03700	no KO assigned | (GenBank) N-formylglutamate amidohydrolase	Cell Wall	56.557	91.775	85.377	185.382	167.487	149.693
AMK58_03705	K00858 NAD+ kinase [EC:2.7.1.23] | (GenBank) ppnK; NAD kinase	Signal Transduction	353.796	398.241	362.813	227.769	1119.608	316.564
AMK58_03710	K03639 GTP 3',8-cyclase [EC:4.1.99.22] | (GenBank) cyclic pyranopterin phosphate synthase MoaA	Biosynthesis: Co-Factors and Vitamins	46.967	33.841	49.032	407.678	179.203	253.865
AMK58_03715	no KO assigned | (GenBank) 4Fe-4S ferredoxin	Ferredoxin	16.202	20.967	17.574	54.689	32.544	58.575
AMK58_03720	K03823 phosphinothricin acetyltransferase [EC:2.3.1.183] | (GenBank) GCN5 family acetyltransferase	Metabolism: Amino Acid	37.997	44.889	52.457	542.057	307.899	326.458
AMK58_03725	no KO assigned | (GenBank) hypothetical protein	Hypothetical	3.363	1.546	4.551	82.566	34.896	30.466
AMK58_03730	no KO assigned | (GenBank) hypothetical protein	Hypothetical	222.597	275.564	250.497	492.602	365.451	413.283
AMK58_03735	K07716 two-component system, cell cycle sensor histidine kinase PleC [EC:2.7.13.3] | (GenBank) hypot	Signal Transduction	168.915	254.969	282.838	300.799	42.759	118.719
AMK58_03740	no KO assigned | (GenBank) hypothetical protein	Hypothetical	105.243	158.295	150.216	421.617	150.293	236.633
AMK58_03745	no KO assigned | (GenBank) hypothetical protein	Hypothetical	42.844	35.183	40.890	91.334	41.781	133.411
AMK58_03750	no KO assigned | (GenBank) enoyl-CoA hydratase	Metabolism	18.973	23.366	23.340	175.673	32.082	82.485
AMK58_03755	K00925 acetate kinase [EC:2.7.2.1] | (GenBank) acetate kinase	Signal Transduction	146.211	141.201	129.592	19.988	14.746	86.454
AMK58_03760	no KO assigned | (GenBank) hypothetical protein	Hypothetical	1039.480	1541.943	1102.175	278.487	231.397	327.060
AMK58_03765	no KO assigned | (GenBank) hypothetical protein	Hypothetical	757.685	875.293	975.094	441.012	560.530	499.831
AMK58_03770	no KO assigned | (GenBank) hypothetical protein	Hypothetical	28.678	35.742	38.373	161.168	320.433	80.834
AMK58_03775	no KO assigned | (GenBank) hypothetical protein	Hypothetical	79.148	78.082	83.108	41.737	1041.383	78.628
AMK58_03780	no KO assigned | (GenBank) hypothetical protein	Hypothetical	183.227	220.222	201.330	350.321	1384.219	255.725
AMK58_03785	no KO assigned | (GenBank) hypothetical protein	Hypothetical	29.351	19.341	36.731	128.072	309.788	126.478
AMK58_03790	no KO assigned | (GenBank) hypothetical protein	Hypothetical	587.740	729.872	871.343	151.714	464.679	150.199
AMK58_03795	no KO assigned | (GenBank) two-component system response regulator	Signal Transduction	1124.918	1139.040	1461.125	167.323	401.300	187.793
AMK58_03800	no KO assigned | (GenBank) two-component system response regulator	Signal Transduction	145.114	152.010	165.838	683.061	475.340	526.203
AMK58_03805	K03657 DNA helicase II / ATP-dependent DNA helicase PcrA [EC:3.6.4.12] | (GenBank) DNA helicase I	DNA Repair and Replication	11.745	9.583	14.464	83.727	46.586	70.912
AMK58_03810	no KO assigned | (GenBank) hypothetical protein	Hypothetical	43.797	44.114	42.028	134.952	23.308	90.791
AMK58_03815	K04771 serine protease Do [EC:3.4.21.107] | (GenBank) hypothetical protein	Defense	74.291	74.760	78.461	219.570	54.995	154.292
AMK58_03820	K02687 ribosomal protein L11 methyltransferase [EC:2.1.1.-] | (GenBank) 50S ribosomal protein L11 me	Ribosome	59.810	52.967	54.603	126.204	265.196	100.736
AMK58_03825	no KO assigned | (GenBank) hypothetical protein	Hypothetical	299.804	330.364	380.491	958.020	714.047	538.749
AMK58_03830	K01262 Xaa-Pro aminopeptidase [EC:3.4.11.9] | (GenBank) X-Pro aminopeptidase	Peptidase	163.758	153.148	133.427	17.329	37.122	43.670
AMK58_03835	K03413 two-component system, chemotaxis family, response regulator CheY | (GenBank) two-component	Chemotaxis	1048.322	996.096	1074.129	711.668	394.150	503.066
AMK58_03840	no KO assigned | (GenBank) hypothetical protein	Hypothetical	85.608	87.071	87.308	80.137	74.805	139.676
AMK58_03845	K03414 chemotaxis protein CheZ | (GenBank) hypothetical protein	Chemotaxis	299.898	302.868	249.580	96.257	175.372	243.707
AMK58_03850	K05807 outer membrane protein assembly factor BamD | (GenBank) transporter	Membrane	69.186	72.424	73.935	445.962	233.303	192.325
AMK58_03855	K03631 DNA repair protein RecN (Recombination protein N) | (GenBank) DNA repair protein RecN	DNA Repair and Replication	2.681	3.896	5.856	148.889	83.317	228.839
AMK58_03860	no KO assigned | (GenBank) hypothetical protein	Hypothetical	6.031	4.751	7.246	16.955	57.357	74.293
AMK58_03865	no KO assigned | (GenBank) hypothetical protein	Hypothetical	63.972	89.778	68.173	203.883	184.112	250.019
AMK58_03870	no KO assigned | (GenBank) hypothetical protein	Hypothetical	201.345	194.493	218.578	998.503	163.161	706.493
AMK58_03875	K01972 DNA ligase (NAD+) [EC:6.5.1.2] | (GenBank) aromatic ring-opening dioxygenase LigA	Energy	25.959	40.893	24.682	16.096	25.076	43.864
AMK58_03880	K07216 hemerythrin | (GenBank) hypothetical protein	No COG	1217.424	1325.245	1317.665	446.316	703.319	656.454
AMK58_03885	K02535 UDP-3-O-[3-hydroxymyristoyl] N-acetylglucosamine deacetylase [EC:3.5.1.108] | (GenBank) UI	Biosynthesis: Lipopolysaccharide	558.866	504.435	495.681	317.850	68.407	205.293
AMK58_03890	K03531 cell division protein FtsZ | (GenBank) cell division protein FtsZ	Cell Cycle/Shape/Homeostatsis	73.933	91.699	83.847	126.114	38.356	147.342
AMK58_03895	K03590 cell division protein FtsA | (GenBank) cell division protein FtsA	Cell Cycle/Shape/Homeostatsis	70.843	69.300	77.475	122.763	30.387	103.914
AMK58_03900	K03589 cell division protein FtsQ | (GenBank) cell division protein	Cell Cycle/Shape/Homeostatsis	51.548	61.594	59.293	164.544	32.363	128.358
AMK58_03905	K01921 D-alanine-D-alanine ligase [EC:6.3.2.4] | (GenBank) D-alanine--D-alanine ligase	Cell Wall	17.315	22.819	27.478	216.418	26.045	96.332
AMK58_03910	K00075 UDP-N-acetylmuramate dehydrogenase [EC:1.3.1.98] | (GenBank) UDP-N-acetylenolpyruvoylglu	Cell Wall	114.544	90.517	102.452	279.758	58.871	235.803
AMK58_03915	K01924 UDP-N-acetylmuramate--alanine ligase [EC:6.3.2.8] | (GenBank) murC; UDP-N-acetylmuramate--	Cell Wall	3.506	4.087	5.447	55.322	4.709	14.272
AMK58_03920	no KO assigned | (GenBank) hypothetical protein	Hypothetical	44.893	43.347	53.032	596.504	55.049	155.580
AMK58_03925	K02563 UDP-N-acetylglucosamine--N-acetylmuramyl-(pentapeptide) pyrophosphoryl-undecaprenol N-ace	Cell Wall	69.990	60.280	65.032	170.864	34.859	161.609
AMK58_03930	K03588 cell division protein FtsW | (GenBank) cell division protein FtsW	Cell Cycle/Shape/Homeostatsis	54.457	57.868	63.546	205.435	52.865	164.662
AMK58_03935	K01925 UDP-N-acetylmuramoylalanine--D-glutamate ligase [EC:6.3.2.9] | (GenBank) UDP-N-acetylmura	Cell Wall	84.856	82.949	85.683	41.817	43.232	164.886
AMK58_03940	K01000 phospho-N-acetylmuramoyl-pentapeptide-transferase [EC:2.7.8.13] | (GenBank) mraY; phospho-N	Cell Wall	40.024	47.957	47.807	198.028	50.878	110.099
AMK58_03945	K01929 UDP-N-acetylmuramoyl-tripeptide--D-alanyl-D-alanine ligase [EC:6.3.2.10] | (GenBank) UDP-N-	Cell Wall	43.836	46.388	60.751	158.177	40.819	90.521
AMK58_03950	K01928 UDP-N-acetylmuramoyl-L-alanyl-D-glutamate--2,6-diaminopimelate ligase [EC:6.3.2.13] | (GenBa	Cell Wall	193.536	170.875	191.151	182.962	198.610	248.277
AMK58_03955	K03587 cell division protein FtsI (penicillin-binding protein 3) | (GenBank) penicillin-binding prot	Cell Cycle/Shape/Homeostatsis	54.504	54.071	69.989	68.610	48.153	43.578
AMK58_03960	no KO assigned | (GenBank) hypothetical protein	Hypothetical	61.038	73.730	85.784	276.565	128.220	153.714
AMK58_03965	K03438 16S rRNA (cytosine1402-N4)-methyltransferase [EC:2.1.1.199] | (GenBank) ribosomal RNA sma	Ribosome	85.201	88.338	84.928	73.381	117.838	103.729
AMK58_03970	K03925 MraZ protein | (GenBank) MraZ family transcriptional regulator	Transcription	53707.365	79550.229	66340.871	33743.455	74991.723	61993.825
AMK58_03975	no KO assigned | (GenBank) N-acetylmuramoyl-L-alanine amidase	Cell Wall	21.116	31.539	24.287	142.112	64.123	234.645
AMK58_03980	K15268 O-acetylserine/cysteine efflux transporter | (GenBank) multidrug DMT transporter permease	Transport	23.554	25.511	24.098	80.078	51.867	114.058
AMK58_03985	K15738 ATP-binding cassette, subfamily F, uup | (GenBank) elongation factor 3	Transport	61.962	61.368	68.051	172.661	210.136	135.314
AMK58_03990	K05801 DnaJ like chaperone protein | (GenBank) molecular chaperone DjlA	Chaperone	141.090	152.904	194.361	314.043	1645.289	252.379
AMK58_03995	no KO assigned | (GenBank) GCN5 family acetyltransferase	Acetyltransferase	0.436	2.607	3.111	256.867	19.862	215.234
AMK58_04000	K01469 5-oxoprolinase (ATP-hydrolysing) [EC:3.5.2.9] | (GenBank) 5-oxoprolinase	Metabolism: Amino Acide	108.226	98.812	109.920	178.499	117.276	146.084
AMK58_04005	K00102 D-lactate dehydrogenase (cytochrome) [EC:1.1.2.4] | (GenBank) lactate dehydrogenase	Metabolism: Carbohydrate	371.417	348.282	380.204	175.239	145.011	228.538
AMK58_04010	K00020 3-hydroxyisobutyrate dehydrogenase [EC:1.1.1.31] | (GenBank) oxidoreductase	Metabolism: Amino Acid	67.126	63.970	65.652	131.845	52.154	157.967
AMK58_04015	no KO assigned | (GenBank) hypothetical protein	Hypothetical	886.309	1111.650	883.077	165.696	213.272	355.278

AMK58_04020	no KO assigned | (GenBank) hypothetical protein	Hypothetical	40.358	30.618	64.564	155.321	479.566	258.398
AMK58_04025	no KO assigned | (GenBank) histidine kinase	Signal Transduction	79.583	99.563	98.735	217.463	131.569	170.086
AMK58_04030	no KO assigned | (GenBank) alkylhydroperoxidase	Defense	94.066	90.883	81.764	177.157	140.630	217.918
AMK58_04035	no KO assigned | (GenBank) hypothetical protein	Hypothetical	12.868	13.978	16.128	228.392	65.746	89.490
AMK58_04040	K14232 tRNA Pro | (GenBank) tRNA-Pro	tRNA synthesis/modification	37.738	28.183	25.416	58.977	28.279	533.645
AMK58_04045	K14232 tRNA Pro | (GenBank) tRNA-Pro	tRNA synthesis/modification	75.475	206.673	100.168	227.481	121.954	704.237
AMK58_04050	K02523 octaprenyl-diphosphate synthase [EC:2.5.1.90] | (GenBank) farnesyltranstransferase	Biosynthesis: Terpenoids	230.721	259.611	193.110	209.869	409.216	242.313
AMK58_04055	no KO assigned | (GenBank) hypothetical protein	Hypothetical	114.348	100.466	120.981	102.117	158.775	316.538
AMK58_04060	no KO assigned | (GenBank) methyltransferase	Methyltransferase	19.854	23.334	22.745	183.767	43.535	70.018
AMK58_04065	no KO assigned | (GenBank) peptidase S49	Peptidase	97.246	110.273	109.225	174.548	176.348	192.608
AMK58_04070	no KO assigned | (GenBank) hypothetical protein	Hypothetical	89.213	88.833	77.251	93.186	158.178	82.233
AMK58_04075	no KO assigned | (GenBank) hypothetical protein	Hypothetical	36.771	32.149	26.008	505.780	123.492	104.369
AMK58_04080	K01878 glycyl-tRNA synthetase alpha chain [EC:6.1.1.14] | (GenBank) glycine--tRNA ligase subunit alp	tRNA synthesis/modification	189.793	171.064	153.103	185.199	139.312	164.612
AMK58_04085	K01879 glycyl-tRNA synthetase beta chain [EC:6.1.1.14] | (GenBank) glycine--tRNA ligase subunit beta	tRNA synthesis/modification	127.453	123.331	122.121	231.783	84.472	203.677
AMK58_04090	K01649 2-isopropylmalate synthase [EC:2.3.3.13] | (GenBank) 2-isopropylmalate synthase	Metabolism	141.645	110.566	141.242	161.614	524.451	245.330
AMK58_04095	no KO assigned | (GenBank) hypothetical protein	Hypothetical	26.124	19.009	21.177	253.710	88.846	166.463
AMK58_04100	no KO assigned | (GenBank) murein hydrolase transporter LrgA	Membrane	18.403	11.574	27.629	235.710	154.239	133.526
AMK58_04105	no KO assigned | (GenBank) dienelactone hydrolase	Hydrolase	2.385	4.384	6.802	344.276	214.037	182.353
AMK58_04110	no KO assigned | (GenBank) hypothetical protein	Hypothetical	2.505	0.000	5.293	73.450	600.687	185.826
AMK58_04115	no KO assigned | (GenBank) hypothetical protein	Hypothetical	1.805	0.000	1.872	114.277	142.179	58.265
AMK58_04120	no KO assigned | (GenBank) alcohol dehydrogenase	Dehydrogenase	1.853	1.845	4.699	138.465	205.413	183.966
AMK58_04125	no KO assigned | (GenBank) hypothetical protein	Hypothetical	2.374	0.000	4.514	252.501	140.363	243.031
AMK58_04130	K01236 maltooligosyltrehalose trehalohydrolase [EC:3.2.1.141] | (GenBank) malto-oligosyltrehalose tr	Metabolism: Carbohydrate	1.267	1.514	3.735	244.255	73.067	164.791
AMK58_04135	no KO assigned | (GenBank) glycogen debranching protein	Metabolism: Carbohydrate	1.234	2.808	4.413	234.426	97.464	158.735
AMK58_04140	no KO assigned | (GenBank) hypothetical protein	Hypothetical	197.673	224.511	223.190	180.942	162.479	232.940
AMK58_04145	no KO assigned | (GenBank) hypothetical protein	Hypothetical	794.076	1257.726	1026.391	182.642	255.430	333.438
AMK58_04150	no KO assigned | (GenBank) hypothetical protein	Hypothetical	433.686	442.709	460.790	143.131	283.342	272.392
AMK58_04155	no KO assigned | (GenBank) hypothetical protein	Hypothetical	72.062	61.504	61.154	209.780	188.421	203.251
AMK58_04160	no KO assigned | (GenBank) hypothetical protein	Hypothetical	5.765	2.460	10.181	169.357	1551.648	360.103
AMK58_04165	no KO assigned | (GenBank) hypothetical protein	Hypothetical	28.975	29.676	32.666	123.649	1943.457	265.609
AMK58_04170	no KO assigned | (GenBank) peptidoglycan-binding protein	Cell Wall	123.431	130.239	120.814	213.486	133.003	158.161
AMK58_04175	no KO assigned | (GenBank) hypothetical protein	Hypothetical	104.039	75.122	90.822	196.959	62.462	195.767
AMK58_04180	no KO assigned | (GenBank) hypothetical protein	Hypothetical	30.681	30.755	36.415	181.218	999.405	274.564
AMK58_04185	no KO assigned | (GenBank) pseudogene	Pseudogene	435.514	537.880	556.619	83.172	106.681	252.504
AMK58_04190	K03406 methyl-accepting chemotaxis protein | (GenBank) chemotaxis protein	Chemotaxis	70.826	63.546	73.958	140.082	24.470	124.575
AMK58_04195	no KO assigned | (GenBank) hypothetical protein	Hypothetical	22.460	29.354	26.027	127.868	75.344	127.239
AMK58_04200	no KO assigned | (GenBank) hypothetical protein	Hypothetical	4.387	6.552	10.219	172.763	128.697	189.150
AMK58_04205	no KO assigned | (GenBank) hypothetical protein	Hypothetical	65.499	64.726	63.536	166.728	51.035	193.558
AMK58_04210	K02030 polar amino acid transport system substrate-binding protein | (GenBank) amino acid ABC transp	Transport: Amino Acid	19.625	17.966	20.616	170.878	24.016	156.297
AMK58_04215	K03786 3-dehydroquinate dehydratase II [EC:4.2.1.10] | (GenBank) 3-dehydroquinate dehydratase	Biosynthesis: Amino Acid	537.006	424.656	403.489	69.393	201.770	211.693
AMK58_04220	K02160 acetyl-CoA carboxylase biotin carboxyl carrier protein | (GenBank) acetyl-CoA carboxylase bio	Metabolism: Lipid	141.792	139.849	129.445	58.027	67.744	49.648
AMK58_04225	K01961 acetyl-CoA carboxylase, biotin carboxylase subunit [EC:6.4.1.2 6.3.4.14] | (GenBank) acetyl-C	Metabolism: Lipid	234.853	226.049	206.769	169.547	97.108	209.754
AMK58_04230	K01166 ribonuclease T2 [EC:3.1.27.1] | (GenBank) ribonuclease T	Ribosome	83.679	60.833	63.192	116.556	26.636	262.134
AMK58_04235	K00684 leucyl/phenylalanyl-tRNA--protein transferase [EC:2.3.2.6] | (GenBank) leucyl/phenylalanyl-tR	tRNA synthesis/modification	31.495	40.562	40.345	212.964	39.005	270.706
AMK58_04240	no KO assigned | (GenBank) glycosyl hydrolase family 5	Hydrolase	132.458	152.502	118.727	236.117	100.034	282.114
AMK58_04245	no KO assigned | (GenBank) hypothetical protein	Hypothetical	15.246	24.112	31.977	87.100	114.923	150.109
AMK58_04250	K03694 ATP-dependent Clp protease ATP-binding subunit ClpA | (GenBank) clpA; ATP-dependent Clp p	Chaperone	906.649	1028.814	928.159	66.947	3409.851	244.366
AMK58_04255	K06891 ATP-dependent Clp protease adaptor protein ClpS | (GenBank) ATP-dependent Clp protease adap	Chaperone	1396.248	1586.073	1119.153	50.590	3900.506	293.892
AMK58_04260	no KO assigned | (GenBank) hypothetical protein	Hypothetical	47.583	44.652	56.138	88.768	182.858	240.248
AMK58_04265	no KO assigned | (GenBank) hypothetical protein	Hypothetical	518.119	645.228	618.684	155.843	361.901	244.401
AMK58_04270	K07395 putative proteasome-type protease | (GenBank) peptidase	No COG	101.531	94.929	92.156	80.667	161.990	231.169
AMK58_04275	no KO assigned | (GenBank) hypothetical protein	Hypothetical	51.171	50.043	59.080	182.383	637.498	128.086
AMK58_04280	K01286 D-alanyl-D-alanine carboxypeptidase [EC:3.4.16.4] | (GenBank) D-alanyl-D-alanine carboxypepti	Cell Wall	88.086	110.076	91.428	253.386	45.266	161.733
AMK58_04285	K05772 tungstate transport system substrate-binding protein | (GenBank) sulfate transporter	Transport: Organic	524.512	436.268	461.580	135.155	121.081	208.251
AMK58_04290	K05773 tungstate transport system permease protein | (GenBank) ABC transporter permease	Transport: Organic	16.700	23.904	17.863	307.128	26.397	300.838
AMK58_04295	K02379 FdhD protein | (GenBank) formate dehydrogenase family accessory protein FdhD	Biosynthesis: Co-Factors and Vitamins	155.393	141.063	137.046	106.285	192.876	127.799
AMK58_04300	K03752 molybdopterin-guanine dinucleotide biosynthesis protein A [EC:2.7.7.77] | (GenBank) molybdenu	Biosynthesis: Co-Factors and Vitamins	67.972	74.989	71.789	145.631	63.163	73.593
AMK58_04305	no KO assigned | (GenBank) hypothetical protein	Hypothetical	93.680	103.754	128.375	258.187	105.025	203.900
AMK58_04310	no KO assigned | (GenBank) hypothetical protein	Hypothetical	68.821	78.681	80.785	212.549	111.740	286.189
AMK58_04315	no KO assigned | (GenBank) molecular chaperone TorD	Chaperone	111.974	118.195	193.370	164.041	396.938	186.749
AMK58_04320	no KO assigned | (GenBank) formate dehydrogenase	Metabolism: Carbon	194.563	136.754	176.974	117.000	504.425	77.639
AMK58_04325	K00123 formate dehydrogenase major subunit [EC:1.2.1.2] | (GenBank) formate dehydrogenase	Metabolism: Carbon	143.789	125.692	179.472	148.881	163.415	215.990
AMK58_04330	K00124 formate dehydrogenase iron-sulfur subunit | (GenBank) formate dehydrogenase	Metabolism: Carbon	107.487	71.487	138.066	92.639	114.665	187.304
AMK58_04335	no KO assigned | (GenBank) hypothetical protein	Hypothetical	57.743	59.352	90.914	143.056	53.739	147.966
AMK58_04340	K00127 formate dehydrogenase subunit gamma | (GenBank) formate dehydrogenase	Metabolism: Carbon	65.799	55.962	92.689	172.565	103.467	190.226
AMK58_04345	no KO assigned | (GenBank) hypothetical protein	Hypothetical	26.265	24.877	34.718	283.182	108.371	196.323
AMK58_04350	no KO assigned | (GenBank) hypothetical protein	Hypothetical	6.534	6.889	12.547	269.452	50.117	188.898

AMK58_04355	K01628 L-fuculose-phosphate aldolase [EC:4.1.2.17] | (GenBank) fuculose phosphate aldolase	Metabolism: Carbohydrate	21.651	22.767	23.057	127.382	42.833	175.873
AMK58_04360	no KO assigned | (GenBank) histidine kinase	Signal Transduction	69.752	73.172	79.629	166.483	32.122	93.288
AMK58_04365	K15777 4,5-DOPA dioxygenase extradiol [EC:1.13.11.-] | (GenBank) extradiol ring-cleavage dioxygenase	Oxidoreductase	19.801	20.771	24.001	287.318	14.952	135.957
AMK58_04370	K02109 F-type H+-transporting ATPase subunit b | (GenBank) ATP synthase subunit B	Energy	633.120	610.331	654.739	307.477	300.539	140.805
AMK58_04375	K02109 F-type H+-transporting ATPase subunit b | (GenBank) ATPase	Energy	999.843	950.029	934.214	153.466	473.086	124.687
AMK58_04380	K02110 F-type H+-transporting ATPase subunit c | (GenBank) ATP synthase subunit C	Energy	7229.510	7657.924	6950.114	59.829	2837.997	150.192
AMK58_04385	K02108 F-type H+-transporting ATPase subunit a | (GenBank) ATP synthase F0F1 subunit A	Energy	1696.023	1219.204	1300.194	120.331	1411.967	325.039
AMK58_04390	K02116 ATP synthase protein I | (GenBank) phosphoribosylaminoimidazolecarboxamide formyltransferase	Energy	865.201	838.933	807.101	145.655	883.668	231.344
AMK58_04395	K07152 protein SCO1/2 | (GenBank) electron transporter SenC	Energy	177.094	257.913	218.974	84.993	217.117	165.891
AMK58_04400	no KO assigned | (GenBank) hypothetical protein	Hypothetical	57.810	68.426	76.227	369.667	28.199	190.150
AMK58_04405	K03406 methyl-accepting chemotaxis protein | (GenBank) chemotaxis protein	Chemotaxis	109.955	125.112	137.069	151.071	13.451	188.634
AMK58_04410	K06217 phosphate starvation-inducible protein PhoH and related proteins | (GenBank) phosphate starva	Starvation	139.613	154.865	163.007	145.704	41.492	269.721
AMK58_04415	K03610 septum site-determining protein MinC | (GenBank) septum site-determining protein MinC	Cell Cycle/Shape/Homeostatsis	209.992	244.000	227.333	127.078	270.198	190.396
AMK58_04420	K03609 septum site-determining protein MinD | (GenBank) septum site-determining protein MinD	Cell Cycle/Shape/Homeostatsis	820.964	729.561	802.870	155.627	260.845	257.147
AMK58_04425	K03608 cell division topological specificity factor | (GenBank) cell division topological specificit	Cell Cycle/Shape/Homeostatsis	221.053	231.985	299.426	189.217	118.910	186.549
AMK58_04430	no KO assigned | (GenBank) hypothetical protein	Hypothetical	228.611	311.702	267.463	254.137	367.568	143.216
AMK58_04435	no KO assigned | (GenBank) chemotaxis protein	Chemotaxis	224.727	220.037	166.283	72.969	172.038	180.163
AMK58_04440	no KO assigned | (GenBank) chemotaxis protein	Chemotaxis	66.402	60.693	52.510	188.536	73.704	171.273
AMK58_04445	no KO assigned | (GenBank) chemotaxis protein	Chemotaxis	24.718	26.138	24.126	141.528	65.158	176.771
AMK58_04450	K19802 L-Ala-D/L-Glu epimerase [EC:5.1.1.20] | (GenBank) mandelate racemase	Metabolism: Amino Acid	188.702	258.496	206.730	223.229	646.409	164.640
AMK58_04455	K11312 cupin 2 domain-containing protein | (GenBank) cupin	No COG	29.236	43.667	37.467	168.145	132.878	49.799
AMK58_04460	no KO assigned | (GenBank) aminotransferase class V	tRNA synthesis/modification	73.168	78.769	94.354	242.807	191.187	175.357
AMK58_04465	no KO assigned | (GenBank) hypothetical protein	Hypothetical	58.443	66.537	77.329	269.762	199.833	200.949
AMK58_04470	K01724 4a-hydroxytetrahydrobiopterin dehydratase [EC:4.2.1.96] | (GenBank) pterin-4-alpha-carbinolam	Biosynthesis	397.258	399.465	372.848	165.413	545.388	275.926
AMK58_04475	K02334 DNA polymerase bacteriophage-type [EC:2.7.7.7] | (GenBank) DNA polymerase	DNA Repair and Replication	11.388	35.810	23.366	238.997	103.754	193.415
AMK58_04480	K00845 glucokinase [EC:2.7.1.2] | (GenBank) glucokinase	Metabolism: Carbohydrate	57.247	64.546	73.440	178.488	95.056	132.881
AMK58_04485	K00697 trehalose 6-phosphate synthase [EC:2.4.1.15] | (GenBank) alpha,alpha-trehalose-phosphate synt	Metabolism: Carbohydrate	17.494	19.145	26.268	103.019	303.143	230.562
AMK58_04490	no KO assigned | (GenBank) N-formylglutamate amidohydrolase	Cell Wall	32.094	33.740	39.421	212.189	160.401	286.102
AMK58_04495	K14237 tRNA Val | (GenBank) tRNA-Val	tRNA synthesis/modification	69.416	125.382	101.305	285.447	132.464	1739.431
AMK58_04500	K03406 methyl-accepting chemotaxis protein | (GenBank) chemotaxis protein	Chemotaxis	5.274	4.312	8.129	137.126	44.179	185.520
AMK58_04505	no KO assigned | (GenBank) chemotaxis protein	Chemotaxis	4.126	4.622	8.214	178.940	23.552	174.741
AMK58_04510	K01638 malate synthase [EC:2.3.3.9] | (GenBank) malate synthase G	Metabolism: Carbon	202.516	171.847	188.261	161.514	80.453	171.454
AMK58_04515	K03581 exodeoxyribonuclease V alpha subunit [EC:3.1.11.5] | (GenBank) recombinase RecD	DNA Repair and Replication	12.881	19.157	19.344	206.250	60.092	212.390
AMK58_04520	K03704 cold shock protein (beta-ribbon, CspA family) | (GenBank) cold-shock protein	Stress Response	3863.856	4044.377	3878.128	95.607	464.817	155.776
AMK58_04525	no KO assigned | (GenBank) transcriptional regulator	Transcription	113.706	107.053	145.849	262.683	56.470	283.392
AMK58_04530	no KO assigned | (GenBank) histidine kinase	Signal Transduction	133.052	161.554	156.657	164.905	84.248	136.892
AMK58_04535	K03406 methyl-accepting chemotaxis protein | (GenBank) chemotaxis protein	Chemotaxis	20.605	24.693	23.001	130.188	119.013	185.594
AMK58_04540	no KO assigned | (GenBank) hypothetical protein	Hypothetical	305.512	166.372	227.552	166.511	665.496	328.576
AMK58_04545	K03855 ferredoxin like protein | (GenBank) ferredoxin	Ferredoxin	5.948	12.690	6.463	63.736	27.696	197.358
AMK58_04550	K00313 electron transfer flavoprotein-quinone oxidoreductase [EC:1.5.5.-] | (GenBank) nitrogen fixat	Nitrogen	7.811	7.778	9.372	103.141	45.573	218.072
AMK58_04555	K03522 electron transfer flavoprotein alpha subunit | (GenBank) electron transfer flavoprotein subun	Energy	3.251	4.019	5.223	188.616	27.597	199.071
AMK58_04560	K03521 electron transfer flavoprotein beta subunit | (GenBank) nitrogen fixation protein FixA	Nitrogen	3.979	2.547	5.945	147.719	17.092	202.665
AMK58_04565	K02595 nitrogenase-stabilizing/protective protein | (GenBank) nitrogen fixation protein NifW	Nitrogen	19.805	15.628	19.186	152.675	31.083	237.360
AMK58_04570	K00640 serine O-acetyltransferase [EC:2.3.1.30] | (GenBank) serine acetyltransferase	Metabolism	3.147	9.808	7.024	275.808	18.915	160.476
AMK58_04575	K02594 homocitrate synthase NifV [EC:2.3.3.14] | (GenBank) homocitrate synthase	Metabolism: Carbohydrate	3.064	2.525	5.123	214.691	16.507	143.423
AMK58_04580	K04487 cysteine desulfurase [EC:2.8.1.7] | (GenBank) cysteine desulfurase NifS	Metabolism: Co-Factors and Vitamins	6.425	7.797	11.168	179.669	20.538	144.666
AMK58_04585	K13819 NifU-like protein | (GenBank) nitrogen fixation protein NifU	Nitrogen	1.846	3.152	3.260	159.889	24.313	162.045
AMK58_04590	no KO assigned | (GenBank) hypothetical protein	Hypothetical	0.953	5.696	2.115	248.600	9.287	60.408
AMK58_04595	no KO assigned | (GenBank) hypothetical protein	Hypothetical	3.161	2.905	9.015	336.747	12.024	201.093
AMK58_04600	K15790 nitrogen fixation protein NifQ | (GenBank) hypothetical protein	Nitrogen	2.868	7.344	7.597	270.035	18.652	274.690
AMK58_04605	no KO assigned | (GenBank) ferredoxin	Ferredoxin	3.295	7.381	7.831	99.849	29.163	277.237
AMK58_04610	no KO assigned | (GenBank) hypothetical protein	Hypothetical	4.679	10.483	17.240	345.526	51.281	379.114
AMK58_04615	no KO assigned | (GenBank) hypothetical protein	Hypothetical	7.291	12.445	5.694	95.219	34.243	160.075
AMK58_04620	K02596 nitrogen fixation protein NifX | (GenBank) nitrogen fixation protein NifX	Nitrogen	0.904	0.000	3.723	138.382	23.698	249.674
AMK58_04625	K02592 nitrogenase molybdenum-iron protein NifN | (GenBank) nitrogen fixation protein NifN	Nitrogen	0.778	2.582	4.355	182.329	20.594	213.961
AMK58_04630	K02587 nitrogenase molybdenum-cofactor synthesis protein NifE | (GenBank) nitrogen fixation protein	Nitrogen	4.788	3.576	6.829	143.287	25.566	204.004
AMK58_04635	K03406 methyl-accepting chemotaxis protein | (GenBank) chemotaxis protein	Chemotaxis	131.897	118.129	135.621	121.857	40.517	217.296
AMK58_04640	K03885 NADH dehydrogenase [EC:1.6.99.3] | (GenBank) pyridine nucleotide-disulfide oxidoreductase	Energy	7.911	8.376	8.379	170.235	19.812	134.185
AMK58_04645	no KO assigned | (GenBank) transcriptional regulator	Transcription	44.919	39.505	51.164	161.575	56.641	254.562
AMK58_04650	K02591 nitrogenase molybdenum-iron protein beta chain [EC:1.18.6.1] | (GenBank) nitrogenase molybden	Nitrogen	19.325	19.011	23.467	81.613	63.772	235.119
AMK58_04655	K02586 nitrogenase molybdenum-iron protein alpha chain [EC:1.18.6.1] | (GenBank) nitrogenase molybde	Nitrogen	15.639	16.577	17.907	49.895	59.824	215.417
AMK58_04660	K02588 nitrogenase iron protein NifH [EC:1.18.6.1] | (GenBank) nifH; nitrogenase reductase	Nitrogen	7.962	10.662	10.964	73.554	34.563	240.705
AMK58_04665	no KO assigned | (GenBank) hypothetical protein	Hypothetical	70.468	79.109	93.561	148.215	34.762	72.155
AMK58_04670	K05951 NAD+---dinitrogen-reductase ADP-D-ribosyltransferase [EC:2.4.2.37] | (GenBank) NAD(+)--dir	Nitrogen	3.000	4.073	8.686	146.479	17.931	272.330
AMK58_04675	no KO assigned | (GenBank) ADP-ribosyl-(dinitrogen reductase) hydrolase	Nitrogen	1.625	2.427	6.052	205.907	15.223	192.767
AMK58_04680	no KO assigned | (GenBank) hypothetical protein	Hypothetical	3.643	10.047	12.258	293.211	17.642	157.749
AMK58_04685	no KO assigned | (GenBank) glutamine amidotransferase	Metabolism: Carbon	86.707	67.815	74.348	147.403	121.917	257.869

AMK58_04690	K01118 FMN-dependent NADH-azoreductase [EC:1.7.-.-] | (GenBank) FMN-dependent NADH-azoredu	Energy	42.869	48.454	47.737	250.651	66.419	287.389
AMK58_04695	no KO assigned | (GenBank) transcriptional regulator	Transcription	18.226	21.606	20.218	193.383	29.267	242.245
AMK58_04700	no KO assigned | (GenBank) UDP-4-amino-4-deoxy-L-arabinose-oxoglutarate aminotransferase	tRNA synthesis/modification	13.419	15.911	16.946	163.284	17.962	216.751
AMK58_04705	no KO assigned | (GenBank) hypothetical protein	Hypothetical	7.222	12.690	8.482	117.229	7.640	52.393
AMK58_04710	K02593 nitrogen fixation protein NifT | (GenBank) nitrogen fixation protein FixU	Nitrogen	21.332	20.667	22.476	97.312	26.571	169.474
AMK58_04715	K04487 cysteine desulfurase [EC:2.8.1.7] | (GenBank) cysteine desulfurase NifS	Metabolism: Co-Factors and Vitamins	4.099	2.512	5.496	137.548	10.750	52.919
AMK58_04720	K02597 nitrogen fixation protein NifZ | (GenBank) nitrogen fixation protein NifZ	Nitrogen	10.866	9.274	14.390	280.706	19.629	160.128
AMK58_04725	K02597 nitrogen fixation protein NifZ | (GenBank) nitrogen fixation protein NifZ	Nitrogen	8.153	7.307	9.303	126.145	15.580	145.157
AMK58_04730	no KO assigned | (GenBank) LRV FeS4 cluster domain-containing protein	No COG	1.251	2.804	2.826	196.970	7.561	76.152
AMK58_04735	no KO assigned | (GenBank) hypothetical protein	Hypothetical	9.523	13.546	11.857	206.225	16.311	114.793
AMK58_04740	no KO assigned | (GenBank) ferredoxin	Ferredoxin	56.123	41.442	38.373	119.105	35.441	271.318
AMK58_04745	K02585 nitrogen fixation protein NifB | (GenBank) nitrogen fixation protein NifB	Nitrogen	3.493	8.346	4.280	139.106	23.206	178.120
AMK58_04750	no KO assigned | (GenBank) adenylate cyclase	cyclic nucleotide	7.066	6.495	9.949	198.409	41.088	142.007
AMK58_04755	no KO assigned | (GenBank) hypothetical protein	Hypothetical	74.036	87.976	79.209	153.784	109.580	191.035
AMK58_04760	K02584 Nif-specific regulatory protein | (GenBank) nif-specific transcriptional activator NifA	Transcription	88.131	106.693	119.103	101.215	188.632	174.562
AMK58_04765	no KO assigned | (GenBank) TetR family transcriptional regulator	Transcription	12.833	11.111	13.439	142.753	24.459	120.375
AMK58_04770	K01993 HlyD family secretion protein | (GenBank) secretion protein HlyD	Secretion System	2.682	3.815	3.279	139.945	12.202	27.712
AMK58_04775	K01990 ABC-2 type transport system ATP-binding protein | (GenBank) multidrug ABC transporter ATP-l	Membrane Transport	19.794	26.108	34.340	212.460	37.563	146.833
AMK58_04780	no KO assigned | (GenBank) hypothetical protein	Hypothetical	208.026	266.603	340.677	90.103	58.273	217.542
AMK58_04785	K01992 ABC-2 type transport system permease protein | (GenBank) mannose-1-phosphate guanyltransfera	Membrane Transport	30.749	29.980	30.150	154.892	70.087	193.353
AMK58_04790	K07261 penicillin-insensitive murein endopeptidase [EC:3.4.24.-] | (GenBank) peptidase	Cell Wall	60.033	73.089	67.752	238.379	43.947	172.849
AMK58_04795	no KO assigned | (GenBank) phosphoethanolamine methyltransferase	Methyltransferase	110.137	123.939	113.087	199.238	124.081	207.985
AMK58_04800	K02919 large subunit ribosomal protein L36 | (GenBank) rpmJ; 50S ribosomal protein L36	Ribosome	803.322	717.615	581.990	360.413	247.344	448.188
AMK58_04805	K00104 glycolate oxidase [EC:1.1.3.15] | (GenBank) glycolate oxidase subunit GlcD	Metabolism: Carbohydrate	211.230	212.923	209.968	175.592	104.631	194.133
AMK58_04810	K11472 glycolate oxidase FAD binding subunit | (GenBank) 2-hydroxy-acid oxidase	Metabolism: Carbohydrate	77.321	95.636	85.786	169.853	85.276	106.062
AMK58_04815	K11473 glycolate oxidase iron-sulfur subunit | (GenBank) 2-hydroxy-acid oxidase	Metabolism: Carbohydrate	104.896	105.791	89.907	124.619	134.376	108.186
AMK58_04820	K01004 phosphatidylcholine synthase [EC:2.7.8.24] | (GenBank) phosphatidylcholine synthase	Metabolism: Lipid	60.537	63.655	68.304	116.990	433.502	183.973
AMK58_04825	no KO assigned | (GenBank) hypothetical protein	Hypothetical	30.707	26.209	61.313	241.516	648.415	131.795
AMK58_04830	no KO assigned | (GenBank) hypothetical protein	Hypothetical	54.287	45.877	73.180	285.380	284.631	199.039
AMK58_04835	K20035 3-(methylthio)propanoyl-CoA dehydrogenase [EC:1.3.8.-] | (GenBank) acyl-CoA dehydrogenase	Energy	450.062	433.611	426.771	228.141	266.062	172.771
AMK58_04840	no KO assigned | (GenBank) hypothetical protein	Hypothetical	58.660	77.102	64.364	191.103	75.563	204.849
AMK58_04845	no KO assigned | (GenBank) hypothetical protein	Hypothetical	13.998	14.120	16.248	202.611	74.381	70.464
AMK58_04850	no KO assigned | (GenBank) hydroxyacid dehydrogenase	Dehydrogenase	90.556	78.214	79.759	163.415	114.629	177.296
AMK58_04855	K07566 L-threonylcarbamoyladenylate synthase [EC:2.7.7.87] | (GenBank) translation factor Sua5	Translation	24.913	32.744	31.622	221.087	94.929	100.950
AMK58_04860	K03496 chromosome partitioning protein | (GenBank) cobyrinic acid a,c-diamide synthase	Cell Cycle/Shape/Homeostatsis	38.303	30.140	35.531	271.561	50.195	177.934
AMK58_04865	no KO assigned | (GenBank) hypothetical protein	Hypothetical	56.283	53.743	58.484	82.396	87.859	187.792
AMK58_04870	K03564 peroxiredoxin Q/BCP [EC:1.11.1.15] | (GenBank) alkyl hydroperoxide reductase	Cell Cycle/Shape/Homeostatsis	366.232	318.995	334.393	90.663	189.347	162.477
AMK58_04875	K00982 [glutamine synthetase] adenylyltransferase / [glutamine synthetase]-adenylyl-L-tyrosine phosp	Metabolism	65.472	63.276	69.155	237.905	103.671	163.234
AMK58_04880	no KO assigned | (GenBank) hypothetical protein	Hypothetical	11.931	13.738	14.639	195.623	83.948	151.732
AMK58_04885	K01866 tyrosyl-tRNA synthetase [EC:6.1.1.1] | (GenBank) tyrosine--tRNA ligase	tRNA synthesis/modification	112.192	87.679	91.710	121.963	93.876	176.731
AMK58_04890	K09001 anhydro-N-acetylmuramic acid kinase [EC:2.7.1.170] | (GenBank) anhydro-N-acetylmuramic acid	Cell Wall	37.524	51.475	52.386	194.988	74.928	91.140
AMK58_04895	K07018 uncharacterized protein | (GenBank) alpha/beta hydrolase	Hydrolase	261.324	226.935	277.466	171.726	570.237	218.274
AMK58_04900	K00640 serine O-acetyltransferase [EC:2.3.1.30] | (GenBank) serine acetyltransferase	Metabolism	329.592	428.032	388.489	134.736	1845.171	177.253
AMK58_04905	K13643 Rrf2 family transcriptional regulator, iron-sulfur cluster assembly transcription factor | (G	Transcription	833.486	903.071	846.589	241.096	1471.103	182.467
AMK58_04910	K04487 cysteine desulfurase [EC:2.8.1.7] | (GenBank) cysteine desulfurase	Metabolism: Co-Factors and Vitamins	94.858	108.536	113.154	205.027	290.772	85.337
AMK58_04915	K04082 molecular chaperone HscB | (GenBank) molecular chaperone DnaJ	Chaperone	37.450	42.359	41.484	181.911	157.754	145.141
AMK58_04920	K04755 ferredoxin, 2Fe-2S | (GenBank) 2Fe-2S ferredoxin	Ferredoxin	194.087	155.631	240.354	252.125	272.599	187.116
AMK58_04925	K00566 tRNA-uridine 2-sulfurtransferase [EC:2.8.1.13] | (GenBank) tRNA-specific 2-thiouridylase	tRNA synthesis/modification	48.793	59.323	62.635	188.216	553.014	131.476
AMK58_04930	K14230 tRNA Met | (GenBank) tRNA-Met	tRNA synthesis/modification	137.218	163.961	138.143	188.136	248.597	13186.447
AMK58_04935	no KO assigned | (GenBank) hypothetical protein	Hypothetical	41.016	45.428	54.223	151.706	76.183	178.167
AMK58_04940	no KO assigned | (GenBank) hypothetical protein	Hypothetical	5.067	7.028	8.518	214.551	30.514	76.860
AMK58_04945	no KO assigned | (GenBank) hypothetical protein	Hypothetical	0.758	2.469	2.881	148.071	18.115	57.220
AMK58_04950	no KO assigned | (GenBank) hypothetical protein	Hypothetical	4.805	6.889	7.492	70.538	44.284	230.955
AMK58_04955	no KO assigned | (GenBank) hypothetical protein	Hypothetical	7.800	10.131	13.866	173.543	47.652	151.265
AMK58_04960	K01607 4-carboxymuconolactone decarboxylase [EC:4.1.1.44] | (GenBank) 4-carboxymuconolactone deca	Metabolism	11.653	20.376	17.565	404.703	43.767	94.349
AMK58_04965	no KO assigned | (GenBank) oxidoreductase	Oxidoreductase	14.390	15.811	19.816	188.109	63.956	152.914
AMK58_04970	no KO assigned | (GenBank) LysR family transcriptional regulator	Transcription	14.855	16.493	21.749	198.638	33.395	182.606
AMK58_04975	no KO assigned | (GenBank) hypothetical protein	Hypothetical	87.524	98.771	79.520	102.479	59.939	199.741
AMK58_04980	K20276 large repetitive protein | (GenBank) hypothetical protein	Quorum Sensing	10.904	10.290	11.231	133.191	37.228	146.671
AMK58_04985	no KO assigned | (GenBank) hypothetical protein	Hypothetical	11.483	12.106	14.691	154.269	134.859	155.565
AMK58_04990	no KO assigned | (GenBank) hypothetical protein	Hypothetical	104.868	136.697	129.320	110.678	592.952	314.119
AMK58_04995	K03413 two-component system, chemotaxis family, response regulator CheY | (GenBank) regulator	Chemotaxis	73.243	94.661	69.071	145.367	116.603	261.131
AMK58_05000	no KO assigned | (GenBank) hypothetical protein	Hypothetical	34.305	38.177	42.210	171.196	88.461	256.973
AMK58_05005	K14236 tRNA Tyr | (GenBank) tRNA-Tyr	tRNA synthesis/modification	28.157	8.411	25.433	158.414	56.969	507.824
AMK58_05010	K15790 nitrogen fixation protein NifQ | (GenBank) hydrogenase	Nitrogen	15.834	16.151	17.626	265.395	94.202	212.721
AMK58_05015	K09386 uncharacterized protein | (GenBank) carbon monoxide dehydrogenase	Dehydrogenase	8.999	4.888	10.890	282.729	107.587	188.128
AMK58_05020	no KO assigned | (GenBank) hypothetical protein	Hypothetical	36.611	32.723	39.195	172.998	57.678	179.097

AMK58_05025	no KO assigned | (GenBank) hypothetical protein	Hypothetical	11.487	3.710	7.970	76.934	18.495	23.030
AMK58_05030	no KO assigned | (GenBank) murein transglycosylase	Cell Wall	33.894	29.243	40.568	261.031	28.236	127.513
AMK58_05035	no KO assigned | (GenBank) hypothetical protein	Hypothetical	7.244	4.122	13.447	302.192	876.657	270.919
AMK58_05040	no KO assigned | (GenBank) hypothetical protein	Hypothetical	4.554	3.753	4.031	139.888	724.771	148.237
AMK58_05045	no KO assigned | (GenBank) hypothetical protein	Hypothetical	21.910	26.398	28.628	189.400	128.515	98.880
AMK58_05050	no KO assigned | (GenBank) alcohol dehydrogenase	Dehydrogenase	274.640	305.822	338.668	206.824	134.845	136.646
AMK58_05055	no KO assigned | (GenBank) hypothetical protein	Hypothetical	118.868	133.745	132.057	112.770	91.083	74.554
AMK58_05060	no KO assigned | (GenBank) hypothetical protein	Hypothetical	21.440	22.605	27.431	125.441	55.997	114.170
AMK58_05065	no KO assigned | (GenBank) glycosyltransferase	Glycosyltransferase	7.869	7.388	18.492	267.147	122.825	212.030
AMK58_05070	no KO assigned | (GenBank) hypothetical protein	Hypothetical	4.248	7.347	12.968	236.465	111.086	118.904
AMK58_05075	no KO assigned | (GenBank) hypothetical protein	Hypothetical	17.949	26.647	26.892	223.971	87.183	151.307
AMK58_05080	no KO assigned | (GenBank) GNAT family acetyltransferase	Acetyltransferase	348.951	396.963	424.677	141.418	94.325	262.191
AMK58_05085	no KO assigned | (GenBank) hypothetical protein	Hypothetical	1855.359	2123.183	2379.125	129.899	170.116	484.943
AMK58_05090	no KO assigned | (GenBank) hypothetical protein	Hypothetical	8.546	14.183	14.898	299.567	85.392	292.341
AMK58_05095	K01679 fumarate hydratase, class II [EC:4.2.1.2] | (GenBank) fumarate hydratase	Metabolism: Carbon	71.470	65.572	56.204	174.573	80.918	200.579
AMK58_05100	no KO assigned | (GenBank) arylsulfate sulfotransferase	Transferase	62.911	63.825	45.145	211.478	88.060	216.284
AMK58_05105	no KO assigned | (GenBank) hypothetical protein	Hypothetical	34.254	29.380	34.826	145.150	51.845	236.332
AMK58_05110	no KO assigned | (GenBank) hypothetical protein	Hypothetical	123.756	156.811	132.762	138.085	51.471	189.013
AMK58_05115	K01589 5-(carboxyamino)imidazole ribonucleotide synthase [EC:6.3.4.18] | (GenBank) phosphoribosylam	Metabolism: Nucleotides	111.237	100.856	96.304	284.387	139.360	119.112
AMK58_05120	K01588 5-(carboxyamino)imidazole ribonucleotide mutase [EC:5.4.99.18] | (GenBank) purE; N5-carboxya	Metabolism: Nucleotides	122.110	117.468	107.494	249.517	166.627	102.674
AMK58_05125	no KO assigned | (GenBank) hypothetical protein	Hypothetical	146.976	140.353	174.683	95.214	165.885	311.674
AMK58_05130	K21071 ATP-dependent phosphofructokinase / diphosphate-dependent phosphofructokinase [EC:2.7.1.11	Metabolism: Carbohydrate	391.988	411.633	365.285	109.169	235.218	136.150
AMK58_05135	no KO assigned | (GenBank) hypothetical protein	Hypothetical	159.861	151.873	193.111	234.151	164.372	88.698
AMK58_05140	no KO assigned | (GenBank) GCN5 family acetyltransferase	Acetyltransferase	71.451	71.351	72.243	195.009	139.565	106.923
AMK58_05145	no KO assigned | (GenBank) hypothetical protein	Hypothetical	272.724	396.384	313.670	76.997	177.334	212.065
AMK58_05150	no KO assigned | (GenBank) hypothetical protein	Hypothetical	15.297	17.543	23.699	167.400	59.949	88.524
AMK58_05155	K02390 flagellar hook protein FlgE | (GenBank) hypothetical protein	Motility	178.301	207.351	185.399	83.518	81.696	197.598
AMK58_05160	K01673 carbonic anhydrase [EC:4.2.1.1] | (GenBank) carbonic anhydrase	Nitrogen	164.054	185.797	154.987	140.187	225.251	237.176
AMK58_05165	K03186 flavin prenyltransferase [EC:2.5.1.129] | (GenBank) 3-octaprenyl-4-hydroxybenzoate carboxy-ly	Biosynthesis: Terpenoid	88.054	85.244	101.940	204.234	258.210	209.797
AMK58_05170	K09987 uncharacterized protein | (GenBank) cytoplasmic protein	No COG	539.743	589.921	579.507	237.768	1652.497	300.657
AMK58_05175	no KO assigned | (GenBank) NAD(P)H-quinone oxidoreductase	Energy	152.545	150.699	162.172	173.288	206.165	179.208
AMK58_05180	no KO assigned | (GenBank) hypothetical protein	Hypothetical	210.760	241.119	286.884	319.223	110.891	290.475
AMK58_05185	no KO assigned | (GenBank) hypothetical protein	Hypothetical	1276.330	1523.352	1599.194	145.002	334.400	256.082
AMK58_05190	no KO assigned | (GenBank) hypothetical protein	Hypothetical	276.202	275.026	266.812	91.230	119.790	339.148
AMK58_05195	no KO assigned | (GenBank) pseudogene	Pseudogene	68.718	74.725	79.857	197.497	81.656	116.889
AMK58_05200	no KO assigned | (GenBank) hypothetical protein	Hypothetical	448.625	518.835	469.726	166.047	510.755	190.123
AMK58_05205	no KO assigned | (GenBank) hypothetical protein	Hypothetical	370.191	363.113	319.090	68.865	952.922	167.736
AMK58_05210	K00286 pyrroline-5-carboxylate reductase [EC:1.5.1.2] | (GenBank) pyrroline-5-carboxylate reductase	Metabolism: Amino Acid	145.787	145.021	200.485	195.877	411.731	147.795
AMK58_05215	no KO assigned | (GenBank) prolyl-tRNA editing protein	tRNA synthesis/modification	46.625	64.398	75.316	208.525	244.010	182.700
AMK58_05220	K00666 fatty-acyl-CoA synthase [EC:6.2.1.-] | (GenBank) acyl-CoA synthetase	Biosynthesis: Lipid	491.903	424.141	426.760	115.959	226.575	185.082
AMK58_05225	no KO assigned | (GenBank) hypothetical protein	Hypothetical	87.344	95.147	88.032	207.105	65.045	164.185
AMK58_05230	no KO assigned | (GenBank) chemotaxis protein	Chemotaxis	169.797	167.669	169.253	170.757	64.337	227.483
AMK58_05235	no KO assigned | (GenBank) ligase	DNA Repair and Replication	31.542	39.211	37.535	271.491	57.146	140.428
AMK58_05240	no KO assigned | (GenBank) heptosyltransferase	Transferase	13.068	22.198	15.227	207.838	57.894	128.071
AMK58_05245	K07027 glycosyltransferase 2 family protein | (GenBank) hypothetical protein	Glycosyltransferase	26.654	27.041	41.840	154.439	56.246	71.815
AMK58_05250	K18979 epoxyqueuosine reductase [EC:1.17.99.6] | (GenBank) epoxyqueuosine reductase	tRNA synthesis/modification	28.070	24.920	31.513	259.437	121.394	151.993
AMK58_05255	K09457 7-cyano-7-deazaguanine reductase [EC:1.7.1.13] | (GenBank) 7-cyano-7-deazaguanine reductase	Biosynthesis: Co-Factors and Vitamins	124.720	104.225	148.541	83.360	196.969	186.875
AMK58_05260	K00773 queuine tRNA-ribosyltransferase [EC:2.4.2.29] | (GenBank) queuine tRNA-ribosyltransferase	tRNA synthesis/modification	79.308	84.181	81.913	141.289	143.899	159.492
AMK58_05265	K07568 S-adenosylmethionine:tRNA ribosyltransferase-isomerase [EC:2.4.99.17] | (GenBank) S-adenosyl	tRNA synthesis/modification	51.397	59.570	64.783	232.307	146.233	124.817
AMK58_05270	no KO assigned | (GenBank) ABC transporter ATP-binding protein	Membrane Transport	184.803	195.472	220.153	254.055	504.767	210.954
AMK58_05275	K03406 methyl-accepting chemotaxis protein | (GenBank) hypothetical protein	Chemotaxis	14.963	20.201	18.879	114.195	38.087	241.883
AMK58_05280	no KO assigned | (GenBank) FAD-linked oxidase	Oxidoreductase	2.549	3.046	5.332	199.403	51.954	131.809
AMK58_05285	no KO assigned | (GenBank) hypothetical protein	Hypothetical	103.305	79.173	81.041	101.265	144.218	325.359
AMK58_05290	K08602 oligoendopeptidase F [EC:3.4.24.-] | (GenBank) oligoendopeptidase F	Peptidase	167.534	179.037	165.539	168.742	251.991	243.127
AMK58_05295	no KO assigned | (GenBank) hypothetical protein	Hypothetical	11.870	12.157	23.862	295.751	34.309	321.399
AMK58_05300	no KO assigned | (GenBank) hypothetical protein	Hypothetical	17.518	25.834	34.051	229.763	30.035	181.050
AMK58_05305	K01908 propionyl-CoA synthetase [EC:6.2.1.17] | (GenBank) prpE; propionate--CoA ligase	Metabolism: Carbohydrate	137.760	116.986	155.408	136.293	81.812	235.632
AMK58_05310	no KO assigned | (GenBank) hypothetical protein	Hypothetical	8.561	14.613	16.279	331.471	21.078	203.749
AMK58_05315	K03088 RNA polymerase sigma-70 factor, ECF subfamily | (GenBank) RNA polymerase subunit sigma-2	Transcription	272.300	312.036	362.060	144.377	740.240	1182.794
AMK58_05320	no KO assigned | (GenBank) hypothetical protein	Hypothetical	88.181	85.412	102.729	212.689	399.320	168.932
AMK58_05325	no KO assigned | (GenBank) hypothetical protein	Hypothetical	52.228	65.462	58.688	132.342	176.121	217.089
AMK58_05330	no KO assigned | (GenBank) hypothetical protein	Hypothetical	21.954	26.839	25.766	168.106	125.098	177.038
AMK58_05335	no KO assigned | (GenBank) hypothetical protein	Hypothetical	114.283	114.707	130.145	117.922	356.015	227.325
AMK58_05340	K04090 indolepyruvate ferredoxin oxidoreductase [EC:1.2.7.8] | (GenBank) indolepyruvate ferredoxin o	Oxidoreductase	1639.554	1692.371	1524.767	168.573	3598.119	245.718
AMK58_05345	no KO assigned | (GenBank) regulator	Signal Transduction	10.810	17.223	15.760	263.391	163.771	125.635
AMK58_05350	no KO assigned | (GenBank) inosine-5-monophosphate dehydrogenase	Dehydrogenase	220.312	307.178	273.342	227.727	711.537	201.727
AMK58_05355	no KO assigned | (GenBank) cation diffusion facilitator family transporter	Transport: Ion	18.181	16.177	15.692	209.339	41.886	233.387

AMK58_05360	no KO assigned | (GenBank) hypothetical protein	Hypothetical	58.041	98.187	103.844	220.428	22.682	84.492
AMK58_05365	K13503 anthranilate synthase [EC:4.1.3.27] | (GenBank) anthranilate synthase	Biosynthesis: Amino Acid	17.870	21.352	21.434	143.920	50.247	82.596
AMK58_05370	no KO assigned | (GenBank) hypothetical protein	Hypothetical	224.645	264.359	226.077	135.481	201.134	205.602
AMK58_05375	K00324 NAD(P) transhydrogenase subunit alpha [EC:1.6.1.2] | (GenBank) NAD(P) transhydrogenase sul	Energy	525.180	412.275	477.475	80.253	85.440	89.389
AMK58_05380	K00324 NAD(P) transhydrogenase subunit alpha [EC:1.6.1.2] | (GenBank) NAD(P) transhydrogenase sul	Energy	158.571	107.734	130.904	29.907	20.913	14.863
AMK58_05385	K00325 NAD(P) transhydrogenase subunit beta [EC:1.6.1.2] | (GenBank) NAD synthetase	Energy	404.537	308.528	375.312	47.784	97.168	52.795
AMK58_05390	no KO assigned | (GenBank) hypothetical protein	Hypothetical	99.634	113.024	93.934	87.288	49.617	196.862
AMK58_05395	K00606 3-methyl-2-oxobutanoate hydroxymethyltransferase [EC:2.1.2.11] | (GenBank) 3-methyl-2-oxobut	Biosynthesis: Co-Factors and Vitamins	138.099	119.244	137.864	217.427	525.237	230.799
AMK58_05400	K02970 small subunit ribosomal protein S21 | (GenBank) 30S ribosomal protein S21	Ribosome	1826.805	2740.950	1910.749	165.366	3697.866	457.334
AMK58_05405	K01462 peptide deformylase [EC:3.5.1.88] | (GenBank) peptide deformylase	Peptidase	380.170	361.012	412.563	94.683	1768.961	217.510
AMK58_05410	K18587 ubiquinone biosynthesis protein COQ9 | (GenBank) ubiquinone biosynthesis protein	Biosynthesis	269.340	290.160	278.042	202.045	1264.629	227.869
AMK58_05415	K07343 DNA transformation protein and related proteins | (GenBank) transcriptional regulator	Transcription	211.400	252.600	224.451	122.712	1610.076	240.281
AMK58_05420	K09985 uncharacterized protein | (GenBank) hypothetical protein	Hypothetical	1971.789	1919.674	2034.822	75.746	8128.453	267.926
AMK58_05425	K03465 thymidylate synthase (FAD) [EC:2.1.1.148] | (GenBank) FAD-dependent thymidylate synthase	Metabolism: Co-Factors and Vitamins	3542.630	4689.100	3223.575	633.199	12364.045	6543.803
AMK58_05430	no KO assigned | (GenBank) hypothetical protein	Hypothetical	67.222	47.694	57.469	48.970	104.972	106.454
AMK58_05435	no KO assigned | (GenBank) hypothetical protein	Hypothetical	179.370	158.960	157.187	775.689	636.110	321.840
AMK58_05440	no KO assigned | (GenBank) hypothetical protein	Hypothetical	314.557	329.765	361.722	350.608	1010.025	216.284
AMK58_05445	K01989 putative ABC transport system substrate-binding protein | (GenBank) ABC transporter permease	Membrane Transport	5.498	6.718	3.802	80.675	25.140	41.594
AMK58_05450	K05832 putative ABC transport system permease protein | (GenBank) ABC transporter permease	Membrane Transport	70.896	56.261	67.920	257.155	61.091	230.652
AMK58_05455	K05833 putative ABC transport system ATP-binding protein | (GenBank) ABC transporter ATP-binding p	Membrane Transport	50.372	32.633	52.655	238.401	59.861	192.040
AMK58_05460	K11747 glutathione-regulated potassium-efflux system protein KefB | (GenBank) potassium transporter	Transport: Ion	9.839	11.196	8.400	40.434	20.011	48.532
AMK58_05465	K03593 ATP-binding protein involved in chromosome partitioning | (GenBank) hypothetical protein	Cell Cycle/Shape/Homeostatsis	67.641	63.355	69.894	378.158	140.259	239.814
AMK58_05470	K04088 membrane protease subunit HflK [EC:3.4.-.-] | (GenBank) hypothetical protein	Hydrolase	422.930	348.628	391.048	159.817	387.234	162.425
AMK58_05475	K04087 membrane protease subunit HflC [EC:3.4.-.-] | (GenBank) hypothetical protein	Hydrolase	980.604	817.209	910.751	201.022	1742.940	186.524
AMK58_05480	K09937 uncharacterized protein | (GenBank) hypothetical protein	Hypothetical	3951.266	3452.205	4069.972	1006.582	5471.811	1116.163
AMK58_05485	K04771 serine protease Do [EC:3.4.21.107] | (GenBank) serine protease	Defense	13.136	11.820	14.522	40.387	34.513	30.819
AMK58_05490	K01079 phosphoserine phosphatase [EC:3.1.3.3] | (GenBank) phosphoserine phosphatase	Metabolism	1402.011	1253.978	1261.817	310.449	1647.987	342.692
AMK58_05495	no KO assigned | (GenBank) hypothetical protein	Hypothetical	63.632	57.691	68.506	289.878	74.865	255.764
AMK58_05500	K00791 tRNA dimethylallyltransferase [EC:2.5.1.75] | (GenBank) tRNA dimethylallyltransferase	tRNA synthesis/modification	1.463	5.099	4.289	123.640	14.802	119.958
AMK58_05505	no KO assigned | (GenBank) GntR family transcriptional regulator	Transcription	17.696	16.075	16.628	146.660	15.703	122.057
AMK58_05510	K01652 acetolactate synthase I/II/III large subunit [EC:2.2.1.6] | (GenBank) acetolactate synthase	Metabolism	196.712	177.968	140.309	81.920	36.569	139.510
AMK58_05515	K01653 acetolactate synthase I/III small subunit [EC:2.2.1.6] | (GenBank) acetolactate synthase smal	Metabolism	448.239	313.882	373.739	456.488	421.701	707.055
AMK58_05520	K00053 ketol-acid reductoisomerase [EC:1.1.1.86] | (GenBank) ketol-acid reductoisomerase	Metabolism	70.390	56.734	63.541	97.789	54.304	152.445
AMK58_05525	K02039 phosphate transport system protein | (GenBank) PhoU family transcriptional regulator	Transport: ion	504.221	317.045	354.011	200.144	116.596	304.004
AMK58_05530	no KO assigned | (GenBank) hypothetical protein	Hypothetical	78.933	83.925	103.035	215.352	105.265	271.473
AMK58_05535	K03569 rod shape-determining protein MreB and related proteins | (GenBank) rod shape-determining pro	Cell Cycle/Shape/Homeostatsis	9.188	11.118	9.621	71.979	29.938	117.015
AMK58_05540	K03570 rod shape-determining protein MreC | (GenBank) rod shape-determining protein MreC	Cell Cycle/Shape/Homeostatsis	378.950	424.867	405.033	229.670	181.301	205.312
AMK58_05545	K03571 rod shape-determining protein MreD | (GenBank) rod shape-determining protein MreD	Cell Cycle/Shape/Homeostatsis	263.391	272.140	282.973	407.204	132.114	291.070
AMK58_05550	K05515 penicillin-binding protein 2 | (GenBank) penicillin-binding protein 2	Cell Wall	9.671	12.326	12.015	23.749	30.291	40.592
AMK58_05555	K03406 methyl-accepting chemotaxis protein | (GenBank) hypothetical protein	Chemotaxis	21.890	25.986	28.542	134.773	80.550	192.736
AMK58_05560	no KO assigned | (GenBank) alkaline phosphatase	Phosphatase	205.184	153.633	168.094	349.011	286.438	468.453
AMK58_05565	no KO assigned | (GenBank) lysine transporter LysE	Transport: Amino Acid	36.395	57.050	42.656	90.988	113.411	217.857
AMK58_05570	K07335 basic membrane protein A and related proteins | (GenBank) hypothetical protein	Membrane	20.728	24.039	23.650	90.811	74.008	154.215
AMK58_05575	K02056 simple sugar transport system ATP-binding protein [EC:3.6.3.17] | (GenBank) heme ABC transpo	Transport: Sugar	97.349	95.129	86.114	100.627	69.907	150.351
AMK58_05580	K02057 simple sugar transport system permease protein | (GenBank) sugar ABC transporter permease	Transport: Sugar	21.378	29.283	26.474	341.834	70.684	133.350
AMK58_05585	K02057 simple sugar transport system permease protein | (GenBank) sugar ABC transporter permease	Transport: Sugar	49.423	40.805	43.804	214.252	85.425	168.692
AMK58_05590	K01489 cytidine deaminase [EC:3.5.4.5] | (GenBank) cytidine deaminase	Metabolism: Nucleotides	77.568	63.272	100.967	419.843	221.996	346.895
AMK58_05595	K03815 xanthosine phosphorylase [EC:2.4.2.-] | (GenBank) purine nucleoside phosphorylase	Metabolism: Nucleotides	7.549	5.204	8.834	118.820	18.766	44.019
AMK58_05600	K01619 deoxyribose-phosphate aldolase [EC:4.1.2.4] | (GenBank) 2-deoxyribose-5-phosphate aldolase	Metabolism: Carbohydrate	19.704	31.662	32.688	231.356	68.428	101.937
AMK58_05605	K00758 thymidine phosphorylase [EC:2.4.2.4] | (GenBank) thymidine phosphorylase	Metabolism: Nucleotides	19.767	21.323	24.712	111.924	50.302	98.607
AMK58_05610	no KO assigned | (GenBank) pseudogene	Pseudogene	14.532	14.395	18.614	154.621	42.825	78.198
AMK58_05615	no KO assigned | (GenBank) S-adenosylmethionine uptake transporter	Membrane Transport	9.988	9.677	14.502	321.840	53.709	145.187
AMK58_05620	no KO assigned | (GenBank) hypothetical protein	Hypothetical	367.388	434.396	442.507	635.443	420.161	239.984
AMK58_05625	K01999 branched-chain amino acid transport system substrate-binding protein | (GenBank) ABC transpor	Transport: Amino Acid	2.201	3.131	3.588	38.054	17.792	28.092
AMK58_05630	K01997 branched-chain amino acid transport system permease protein | (GenBank) ABC transporter perme	Transport: Amino Acid	158.560	162.653	131.249	243.554	180.227	154.640
AMK58_05635	K01995 branched-chain amino acid transport system ATP-binding protein K01998 branched-chain amino a	Transport: Amino Acid	23.213	24.028	20.864	108.975	34.816	62.219
AMK58_05640	K01996 branched-chain amino acid transport system ATP-binding protein | (GenBank) ABC transporter A'	Transport: Amino Acid	97.817	97.060	104.388	374.081	120.715	325.392
AMK58_05645	no KO assigned | (GenBank) hypothetical protein	Hypothetical	15.437	21.098	18.131	138.534	29.600	30.968
AMK58_05650	K03281 chloride channel protein, CIC family | (GenBank) chloride channel protein	Transport: Ion	57.251	60.869	59.934	118.514	54.304	138.966
AMK58_05655	no KO assigned | (GenBank) polyphosphate kinase	Signal Transduction	78.203	94.504	134.938	448.932	623.802	340.373
AMK58_05660	no KO assigned | (GenBank) polyphosphate kinase	Signal Transduction	370.536	387.013	355.784	123.794	243.916	197.150
AMK58_05665	K16147 starch synthase (maltosyl-transferring) [EC:2.4.99.16] | (GenBank) alpha-amylase	Metabolism: Carbohydrate	6.021	6.424	6.168	52.141	24.092	64.211
AMK58_05670	no KO assigned | (GenBank) hypothetical protein	Hypothetical	151.242	211.892	205.431	1350.457	3121.437	2611.309
AMK58_05675	no KO assigned | (GenBank) diguanylate phosphodiesterase	cyclic nucleotide	21.493	24.943	30.875	51.369	65.005	54.486
AMK58_05680	K03520 carbon-monoxide dehydrogenase large subunit [EC:1.2.7.4] | (GenBank) carbon monoxide dehydr	Dehydrogenase	135.276	152.532	141.975	58.398	76.585	56.643
AMK58_05685	no KO assigned | (GenBank) hypothetical protein	Hypothetical	176.418	183.128	211.780	626.389	340.137	502.924
AMK58_05690	no KO assigned | (GenBank) nuclease	DNA Repair and Replication	121.376	165.544	144.615	453.878	313.150	382.052

AMK58_05695	no KO assigned | (GenBank) regulator	Signal Transduction	144.824	229.990	196.293	108.956	114.981	127.815
AMK58_05700	no KO assigned | (GenBank) transglycosylase	Transglycosylase	648.079	765.411	840.190	814.701	7045.197	777.185
AMK58_05705	K01783 ribulose-phosphate 3-epimerase [EC:5.1.3.1] | (GenBank) ribulose phosphate epimerase	Metabolism	50.494	27.525	98.999	103.434	384.458	152.085
AMK58_05710	K02019 molybdate transport system regulatory protein | (GenBank) LysR family transcriptional regulat	Transcription	271.265	225.618	218.452	238.645	222.934	400.963
AMK58_05715	no KO assigned | (GenBank) Zn-dependent oxidoreductase	Oxidoreductase	9.194	15.694	10.218	137.074	38.922	90.126
AMK58_05720	no KO assigned | (GenBank) HxlR family transcriptional regulator	Transcription	74.379	73.730	95.140	647.403	200.953	491.141
AMK58_05725	K07234 uncharacterized protein involved in response to NO | (GenBank) hypothetical protein	Nitrogen	27.374	29.736	35.184	73.049	20.432	76.552
AMK58_05730	K00020 3-hydroxyisobutyrate dehydrogenase [EC:1.1.1.31] | (GenBank) hypothetical protein	Metabolism: Amino Acid	29.466	38.379	37.178	171.165	42.696	73.940
AMK58_05735	no KO assigned | (GenBank) sugar dehydrogenase	Metabolism: Carbohydrate	43.241	49.755	62.432	246.926	82.628	74.401
AMK58_05740	K01754 threonine dehydratase [EC:4.3.1.19] | (GenBank) pyridoxal-5'-phosphate-dependent protein	Metabolism: Amino Acid	36.335	35.694	42.083	137.360	55.095	106.277
AMK58_05745	no KO assigned | (GenBank) hypothetical protein	Hypothetical	71.075	61.720	89.450	162.186	76.020	82.254
AMK58_05750	no KO assigned | (GenBank) HAD family hydrolase	Hydrolase	35.254	40.028	42.620	264.968	101.487	126.322
AMK58_05755	K07234 uncharacterized protein involved in response to NO | (GenBank) short-chain dehydrogenase	Nitrogen	5.408	5.385	6.570	92.831	220.518	126.942
AMK58_05760	no KO assigned | (GenBank) hypothetical protein	Hypothetical	26.051	22.964	35.531	449.086	522.291	218.797
AMK58_05765	K03831 molybdopterin adenylyltransferase [EC:2.7.7.75] | (GenBank) molybdopterin adenylyltransferase	Biosynthesis: Co-Factors and Vitamins	29.148	42.866	44.768	156.479	521.691	209.154
AMK58_05770	no KO assigned | (GenBank) cytochrome c oxidase accessory protein CcoG	Energy	11.578	5.929	11.874	51.514	27.609	75.997
AMK58_05775	K09790 uncharacterized protein | (GenBank) hypothetical protein	Hypothetical	189.241	151.434	183.910	416.343	217.970	690.048
AMK58_05780	no KO assigned | (GenBank) hypothetical protein	Hypothetical	36.663	28.866	36.481	269.548	79.547	197.131
AMK58_05785	no KO assigned | (GenBank) hypothetical protein	Hypothetical	5.435	12.990	7.752	48.783	20.009	82.281
AMK58_05790	K00873 pyruvate kinase [EC:2.7.1.40] | (GenBank) pyruvate kinase	Metabolism: Carbohydrate	45.812	53.136	54.169	93.288	54.607	114.526
AMK58_05795	K02952 small subunit ribosomal protein S13 | (GenBank) 30S ribosomal protein S13	Ribosome	282.180	268.563	345.981	817.522	335.623	730.818
AMK58_05800	K02948 small subunit ribosomal protein S11 | (GenBank) 30S ribosomal protein S11	Ribosome	2227.897	1945.264	1889.509	117.879	2380.096	274.343
AMK58_05805	K03040 DNA-directed RNA polymerase subunit alpha [EC:2.7.7.6] | (GenBank) DNA-directed RNA poly	Transcription	754.106	621.235	645.230	85.863	705.283	102.694
AMK58_05810	K02879 large subunit ribosomal protein L17 | (GenBank) 50S ribosomal protein L17	Ribosome	2564.038	2115.345	2180.184	422.910	2484.103	608.857
AMK58_05815	no KO assigned | (GenBank) cytochrome B	Energy	859.861	991.959	724.116	82.634	1088.040	423.096
AMK58_05820	no KO assigned | (GenBank) hypothetical protein	Hypothetical	266.456	221.939	254.221	209.490	779.443	537.959
AMK58_05825	no KO assigned | (GenBank) hypothetical protein	Hypothetical	43.069	55.781	64.432	137.979	133.385	95.513
AMK58_05830	K07478 putative ATPase | (GenBank) AAA family ATPase	DNA Repair and Replication	47.507	33.519	44.912	30.546	12.613	23.075
AMK58_05835	no KO assigned | (GenBank) hypothetical protein	Hypothetical	73.946	66.625	67.910	284.181	53.572	180.961
AMK58_05840	K06199 CrcB protein | (GenBank) protein CrcB	Transport: ion	26.805	34.188	32.932	611.626	58.229	188.792
AMK58_05845	K06179 23S rRNA pseudouridine955/2504/2580 synthase [EC:5.4.99.24] | (GenBank) RNA pseudouridir	Ribosome	88.669	112.758	113.200	54.539	36.825	82.771
AMK58_05850	no KO assigned | (GenBank) GntR family transcriptional regulator	Transcription	143.711	143.623	152.825	231.996	74.555	229.746
AMK58_05855	K05782 benzoate membrane transport protein | (GenBank) benzoate transporter	Membrane Transport	14.585	14.324	14.627	155.763	20.436	47.891
AMK58_05860	K01091 phosphoglycolate phosphatase [EC:3.1.3.18] | (GenBank) haloacid dehalogenase	Metabolism: Carbohydrate	16.004	12.472	23.487	434.593	68.047	174.373
AMK58_05865	no KO assigned | (GenBank) ATPase	Energy	27.194	27.941	27.339	215.552	50.232	96.048
AMK58_05870	no KO assigned | (GenBank) hypothetical protein	Hypothetical	82.764	101.340	78.136	334.557	275.474	199.319
AMK58_05875	no KO assigned | (GenBank) hypothetical protein	Hypothetical	117.955	217.822	155.160	129.279	256.407	67.976
AMK58_05880	K06980 tRNA-modifying protein YgfZ | (GenBank) glycine cleavage system protein T	tRNA synthesis/modification	49.446	36.727	86.403	230.390	3896.081	155.145
AMK58_05885	K06140 regulator of nucleoside diphosphate kinase | (GenBank) hypothetical protein	transcription	351.058	425.503	385.705	304.099	615.088	421.837
AMK58_05890	K02056 simple sugar transport system ATP-binding protein [EC:3.6.3.17] | (GenBank) ABC transporter	Transport: Sugar	343.316	403.561	375.996	55.930	52.330	90.577
AMK58_05895	K02057 simple sugar transport system permease protein | (GenBank) sugar ABC transporter permease	Transport: Sugar	81.825	68.891	76.641	273.577	72.907	224.025
AMK58_05900	K02057 simple sugar transport system permease protein | (GenBank) ABC transporter permease	Transport: Sugar	72.282	62.799	68.973	230.676	39.967	230.917
AMK58_05905	K02058 simple sugar transport system substrate-binding protein | (GenBank) ABC transporter substrate	Transport: Sugar	78.243	69.179	71.188	190.949	48.144	151.986
AMK58_05910	no KO assigned | (GenBank) hypothetical protein	Hypothetical	4767.283	4486.212	4395.807	312.139	782.784	576.372
AMK58_05915	no KO assigned | (GenBank) hypothetical protein	Hypothetical	26.469	21.920	29.901	419.858	100.155	189.789
AMK58_05920	no KO assigned | (GenBank) hypothetical protein	Hypothetical	6.490	6.058	8.677	107.852	48.328	236.763
AMK58_05925	no KO assigned | (GenBank) hypothetical protein	Hypothetical	9.212	11.794	15.850	238.578	108.234	180.404
AMK58_05930	no KO assigned | (GenBank) hypothetical protein	Hypothetical	3.283	4.904	6.114	106.658	71.660	130.537
AMK58_05935	K00948 ribose-phosphate pyrophosphokinase [EC:2.7.6.1] | (GenBank) ribose-phosphate pyrophosphokin	Metabolism: Carbohydrate	43.473	48.547	37.987	185.044	111.280	201.432
AMK58_05940	K07576 metallo-beta-lactamase family protein | (GenBank) MBL fold metallo-hydrolase	Hydrolase	110.308	128.207	162.441	92.087	33.600	101.099
AMK58_05945	no KO assigned | (GenBank) hypothetical protein	Hypothetical	309.015	312.455	325.149	529.743	173.136	402.355
AMK58_05950	no KO assigned | (GenBank) diguanylate cyclase	cyclic nucleotide	48.091	46.978	43.454	19.262	21.291	39.236
AMK58_05955	no KO assigned | (GenBank) short-chain dehydrogenase	Dehydrogenase	223.937	256.575	247.555	335.877	281.608	403.499
AMK58_05960	no KO assigned | (GenBank) hypothetical protein	Hypothetical	4.399	5.142	6.456	196.391	178.448	90.277
AMK58_05965	no KO assigned | (GenBank) dimethylhistidine N-methyltransferase	Methyltransferase	10.307	8.161	14.641	302.248	236.174	215.212
AMK58_05970	no KO assigned | (GenBank) hemerythrin	No COG	15.035	12.608	20.315	367.127	252.914	296.695
AMK58_05975	K00796 dihydropteroate synthase [EC:2.5.1.15] | (GenBank) dihydropteroate synthase	Biosynthesis: Co-Factors and Vitamins	24.507	23.053	26.474	30.314	296.803	119.716
AMK58_05980	K03431 phosphoglucosamine mutase [EC:5.4.2.10] | (GenBank) phosphoglucosamine mutase	Metabolism: Carbohydrate	31.685	30.669	33.071	138.474	126.742	122.610
AMK58_05985	K00941 hydroxymethylpyrimidine/phosphomethylpyrimidine kinase [EC:2.7.1.49 2.7.4.7] | (GenBank) pho	Metabolism: Co-Factors and Vitamins	252.428	331.425	301.091	295.822	464.688	345.079
AMK58_05990	no KO assigned | (GenBank) transcriptional regulator	Transcription	147.633	172.672	175.773	92.545	206.920	89.816
AMK58_05995	no KO assigned | (GenBank) hypothetical protein	Hypothetical	878.996	1156.926	1069.163	810.433	1180.723	586.755
AMK58_06000	no KO assigned | (GenBank) hypothetical protein	Hypothetical	21.072	40.542	36.335	90.422	532.731	110.283
AMK58_06005	K06890 uncharacterized protein | (GenBank) hypothetical protein	Hypothetical	58.036	66.756	67.073	44.009	566.118	104.383
AMK58_06010	no KO assigned | (GenBank) hypothetical protein	Hypothetical	733.284	713.845	705.048	44.529	4645.799	190.850
AMK58_06015	no KO assigned | (GenBank) hypothetical protein	Hypothetical	35.010	31.955	37.448	644.184	271.093	1296.737
AMK58_06020	no KO assigned | (GenBank) GCN5 family acetyltransferase	Acetyltransferase	1348.953	1283.653	1259.959	49.006	158.612	116.308
AMK58_06025	no KO assigned | (GenBank) isomerase	Isomerase	1157.901	1330.246	1379.690	101.800	122.227	105.350

AMK58_06030	no KO assigned | (GenBank) acyl dehydratase	dehydratase	3582.592	3591.397	3459.375	159.696	297.256	204.104
AMK58_06035	K04046 hypothetical chaperone protein | (GenBank) heat-shock protein	Hypothetical	481.653	468.685	460.566	57.227	74.474	41.718
AMK58_06040	K01915 glutamine synthetase [EC:6.3.1.2] | (GenBank) glnA; glutamine synthetase	Metabolism	83.550	89.778	101.403	208.771	77.699	149.693
AMK58_06045	K04751 nitrogen regulatory protein P-II 1 | (GenBank) transcriptional regulator	Nitrogen	6577.325	5251.263	5242.157	478.903	1251.335	1019.700
AMK58_06050	K14226 tRNA His | (GenBank) tRNA-His	tRNA synthesis/modification	3957.234	3445.457	2988.548	358.516	2052.142	1472.801
AMK58_06055	K14226 tRNA His | (GenBank) tRNA-His	tRNA synthesis/modification	138.599	228.428	245.385	168.588	207.721	781.456
AMK58_06060	no KO assigned | (GenBank) hypothetical protein	Hypothetical	23.687	36.981	28.093	27.528	27.738	60.459
AMK58_06065	K01578 malonyl-CoA decarboxylase [EC:4.1.1.9] | (GenBank) malonyl-CoA decarboxylase	Metabolism: Carbohydrate	16.237	15.391	18.704	139.913	85.727	89.685
AMK58_06070	K14228 tRNA Leu | (GenBank) tRNA-Leu	tRNA synthesis/modification	1888.767	2425.369	1901.494	3209.372	3291.854	2892.595
AMK58_06075	K03545 trigger factor | (GenBank) trigger factor	Chaperone	81.714	74.774	60.966	20.167	45.568	21.192
AMK58_06080	K01358 ATP-dependent Clp protease, protease subunit [EC:3.4.21.92] | (GenBank) clpP; ATP-dependent (	Peptidase	2535.111	1837.529	2068.140	276.459	1260.313	503.529
AMK58_06085	K03544 ATP-dependent Clp protease ATP-binding subunit ClpX | (GenBank) ATP-dependent Clp proteas	Peptidase	610.431	533.539	558.268	49.078	2225.858	163.760
AMK58_06090	K01338 ATP-dependent Lon protease [EC:3.4.21.53] | (GenBank) DNA-binding protein	Peptidase	1431.440	1648.891	1496.546	40.093	2854.858	144.393
AMK58_06095	K03530 DNA-binding protein HU-beta | (GenBank) DNA-binding protein HU	No COG	9424.763	9877.910	9281.623	667.754	21605.418	2144.227
AMK58_06100	K14237 tRNA Val | (GenBank) tRNA-Val	tRNA synthesis/modification	1754.777	2719.817	1708.365	1029.340	553.446	1937.026
AMK58_06105	K14221 tRNA Asp | (GenBank) tRNA-Asp	tRNA synthesis/modification	273.598	347.586	321.436	149.548	346.419	686.740
AMK58_06110	K00330 NADH-quinone oxidoreductase subunit A [EC:1.6.5.3] | (GenBank) NADH:ubiquinone oxidored	Energy	18.703	15.683	30.886	32.965	62.699	257.399
AMK58_06115	K00331 NADH-quinone oxidoreductase subunit B [EC:1.6.5.3] | (GenBank) NADH dehydrogenase	Energy	979.970	768.649	865.406	48.088	1610.561	191.665
AMK58_06120	K00332 NADH-quinone oxidoreductase subunit C [EC:1.6.5.3] | (GenBank) NADH dehydrogenase	Energy	879.041	652.168	736.396	35.526	1199.348	171.078
AMK58_06125	K00333 NADH-quinone oxidoreductase subunit D [EC:1.6.5.3] | (GenBank) NADH dehydrogenase	Energy	523.063	406.627	407.606	30.435	603.066	117.259
AMK58_06130	K00334 NADH-quinone oxidoreductase subunit E [EC:1.6.5.3] | (GenBank) NADH dehydrogenase	Energy	1441.339	1123.659	1060.659	99.726	1410.921	381.317
AMK58_06135	K00335 NADH-quinone oxidoreductase subunit F [EC:1.6.5.3] | (GenBank) NADH dehydrogenase	Energy	207.222	184.056	176.106	44.529	134.304	92.299
AMK58_06140	K00336 NADH-quinone oxidoreductase subunit G [EC:1.6.5.3] | (GenBank) NADH dehydrogenase	Energy	397.427	307.383	297.653	46.205	227.801	144.093
AMK58_06145	K00337 NADH-quinone oxidoreductase subunit H [EC:1.6.5.3] | (GenBank) NADH:ubiquinone oxidored	Energy	907.881	759.130	825.873	238.867	478.682	432.644
AMK58_06150	K00338 NADH-quinone oxidoreductase subunit I [EC:1.6.5.3] | (GenBank) NADH dehydrogenase	Energy	1191.931	897.908	1025.241	96.184	364.863	385.712
AMK58_06155	K00339 NADH-quinone oxidoreductase subunit J [EC:1.6.5.3] | (GenBank) NADH:ubiquinone oxidoredu	Energy	403.583	319.923	310.728	88.873	124.586	185.473
AMK58_06160	K00340 NADH-quinone oxidoreductase subunit K [EC:1.6.5.3] | (GenBank) NADH-quinone oxidoreduct	Energy	793.451	564.171	662.027	237.243	236.952	259.419
AMK58_06165	K00341 NADH-quinone oxidoreductase subunit L [EC:1.6.5.3] | (GenBank) NADH:ubiquinone oxidoredu	Energy	112.493	77.601	90.805	13.438	22.487	32.907
AMK58_06170	K00342 NADH-quinone oxidoreductase subunit M [EC:1.6.5.3] | (GenBank) NADH-quinone oxidoreduc	Energy	994.922	762.674	785.930	100.052	228.426	266.300
AMK58_06175	K00343 NADH-quinone oxidoreductase subunit N [EC:1.6.5.3] | (GenBank) NADH-quinone oxidoreduct	Energy	995.170	748.316	767.698	115.847	103.314	273.740
AMK58_06180	K03524 BirA family transcriptional regulator, biotin operon repressor / biotin-[acetyl-CoA-carboxyla	Transcription	1010.327	678.771	784.257	135.257	154.753	296.279
AMK58_06185	K03525 type III pantothenate kinase [EC:2.7.1.33] | (GenBank) type III pantothenate kinase	Metabolism: Co-Factors and Vitamins	53.342	60.747	55.923	293.569	20.221	137.799
AMK58_06190	K12574 ribonuclease J [EC:3.1.-.-] | (GenBank) MBL fold metallo-hydrolase	Genetic Information Processing	58.497	61.860	50.661	75.424	25.656	80.103
AMK58_06195	K05606 methylmalonyl-CoA/ethylmalonyl-CoA epimerase [EC:5.1.99.1] | (GenBank) methylmalonyl-CoA	Metabolism	203.286	223.258	214.036	855.781	194.563	563.566
AMK58_06200	no KO assigned | (GenBank) hypothetical protein	Hypothetical	195.470	229.498	168.286	134.829	100.020	256.101
AMK58_06205	K01246 DNA-3-methyladenine glycosylase I [EC:3.2.2.20] | (GenBank) DNA-3-methyladenine glycosylas	DNA Repair and Replication	19.170	27.729	15.925	222.195	18.599	51.457
AMK58_06210	no KO assigned | (GenBank) hypothetical protein	Hypothetical	31.192	35.146	30.178	73.854	36.138	160.730
AMK58_06215	no KO assigned | (GenBank) hypothetical protein	Hypothetical	306.783	320.887	289.384	232.507	204.993	648.294
AMK58_06220	K01881 prolyl-tRNA synthetase [EC:6.1.1.15] | (GenBank) proline--tRNA ligase	tRNA synthesis/modification	17.046	17.616	17.522	58.259	14.707	33.835
AMK58_06225	K09808 lipoprotein-releasing system permease protein | (GenBank) multidrug ABC transporter substrate	Membrane Transport	423.665	356.461	342.774	85.771	132.604	218.152
AMK58_06230	K09810 lipoprotein-releasing system ATP-binding protein [EC:3.6.3.-] | (GenBank) ABC transporter	Membrane Transport	122.479	99.592	117.788	201.910	126.231	353.243
AMK58_06235	K01061 carboxymethylenebutenolidase [EC:3.1.1.45] | (GenBank) carboxymethylenebutenolidase	Metabolism	23.707	16.861	28.310	123.057	45.523	73.145
AMK58_06240	K15268 O-acetylserine/cysteine efflux transporter | (GenBank) hypothetical protein	Transport	630.598	711.951	679.176	172.962	123.220	170.427
AMK58_06245	no KO assigned | (GenBank) hypothetical protein	Hypothetical	133.524	128.357	152.156	332.172	109.010	299.013
AMK58_06250	K02337 DNA polymerase III subunit alpha [EC:2.7.7.7] | (GenBank) DNA polymerase III subunit alpha	DNA Repair and Replication	4.206	6.230	5.751	45.634	7.111	35.135
AMK58_06255	K02967 small subunit ribosomal protein S2 | (GenBank) 30S ribosomal protein S2	Ribosome	181.384	189.255	165.076	675.060	139.517	886.154
AMK58_06260	K02357 elongation factor Ts | (GenBank) elongation factor Ts	Translation	1560.042	1215.704	1447.899	182.416	3276.627	250.253
AMK58_06265	K09903 uridylate kinase [EC:2.7.4.22] | (GenBank) pyrH; uridylate kinase	Signal Transduction	972.602	807.556	909.379	195.438	1924.387	237.014
AMK58_06270	K02838 ribosome recycling factor | (GenBank) ribosome-recycling factor	Translation	963.496	983.874	944.990	168.400	2029.926	460.908
AMK58_06275	K00806 undecaprenyl diphosphate synthase [EC:2.5.1.31] | (GenBank) UDP pyrophosphate synthase	Biosynthesis: Terpenoid	304.328	341.094	325.078	125.485	641.371	163.536
AMK58_06280	K00981 phosphatidate cytidylyltransferase [EC:2.7.7.41] | (GenBank) phosphatidate cytidylyltransfera	Metabolism: Lipid	250.809	252.204	263.171	113.509	674.730	183.101
AMK58_06285	K00099 1-deoxy-D-xylulose-5-phosphate reductoisomerase [EC:1.1.1.267] | (GenBank) 1-deoxy-D-xylulc	Biosynthesis: Terpenoid	39.443	53.447	44.946	162.594	169.802	59.670
AMK58_06290	K11749 regulator of sigma E protease [EC:3.4.24.-] | (GenBank) RIP metalloprotease RseP	Quorum Sensing	43.027	69.529	48.322	145.166	144.134	93.855
AMK58_06295	K07277 outer membrane protein insertion porin family | (GenBank) outer membrane protein assembly fac	Membrane	143.837	169.608	159.231	85.787	251.488	97.471
AMK58_06300	no KO assigned | (GenBank) hypothetical protein	Hypothetical	2272.191	2020.148	1914.871	424.372	730.296	873.338
AMK58_06305	K02372 3-hydroxyacyl-[acyl-carrier-protein] dehydratase [EC:4.2.1.59] | (GenBank) 3-hydroxyacyl-ACP	Metabolism: Lipid	691.232	672.675	631.565	74.034	184.925	211.906
AMK58_06310	K00677 UDP-N-acetylglucosamine acyltransferase [EC:2.3.1.129] | (GenBank) UDP-N-acetylglucosamine	Biosynthesis: Lipopolysaccharide	506.243	539.079	421.665	34.123	141.613	118.815
AMK58_06315	K09949 uncharacterized protein | (GenBank) UDP-2,3-diacylglucosamine pyrophosphatase	No COG	274.465	281.448	252.633	74.840	104.774	152.090
AMK58_06320	K00748 lipid-A-disaccharide synthase [EC:2.4.1.182] | (GenBank) lipid-A-disaccharide synthase	Biosynthesis: Lipopolysaccharide	12.891	15.403	13.765	145.981	26.416	47.813
AMK58_06325	K01759 lactoylglutathione lyase [EC:4.4.1.5] | (GenBank) glyoxalase I	Metabolism: Carbohydrate	57.480	63.933	59.594	676.184	168.054	313.845
AMK58_06330	no KO assigned | (GenBank) hypothetical protein	Hypothetical	120.387	154.809	118.389	37.782	181.973	92.282
AMK58_06335	K03566 LysR family transcriptional regulator, glycine cleavage system transcriptional activator | (G	Transcription	19.588	26.704	22.874	77.835	66.347	130.067
AMK58_06340	K00799 glutathione S-transferase [EC:2.5.1.18] | (GenBank) glutathione S-transferase	Metabolism: Amino Acid	9.543	22.568	17.958	406.929	66.147	219.378
AMK58_06345	no KO assigned | (GenBank) hypothetical protein	Hypothetical	280.168	241.119	276.397	196.660	110.452	424.130
AMK58_06350	K01647 citrate synthase [EC:2.3.3.1] | (GenBank) gltA; type II citrate synthase	Metabolism	7.003	5.505	8.849	22.834	2.382	8.288
AMK58_06355	K01885 glutamyl-tRNA synthetase [EC:6.1.1.17] | (GenBank) glutamate--tRNA ligase	tRNA synthesis/modification	1807.125	1384.768	1566.774	156.906	304.645	191.486
AMK58_06360	K02238 competence protein ComEC | (GenBank) competence protein ComEC	Secretion System	76.184	69.497	75.136	101.394	65.192	105.889

AMK58_06365	K01356 repressor LexA [EC:3.4.21.88] | (GenBank) LexA family transcriptional regulator	Transcription	34.469	31.045	52.207	693.292	124.801	432.054
AMK58_06370	K03750 molybdopterin molybdotransferase [EC:2.10.1.1] | (GenBank) molybdenum cofactor biosynthesis	Biosynthesis: Co-Factors and Vitamins	62.625	54.044	73.721	67.910	56.426	89.041
AMK58_06375	K03637 cyclic pyranopterin monophosphate synthase [EC:4.6.1.17] | (GenBank) moaC; molybdenum cofac	Biosynthesis: Co-Factors and Vitamins	57.480	78.912	87.211	380.727	61.861	143.342
AMK58_06380	K03829 putative acetyltransferase [EC:2.3.1.-] | (GenBank) GCN5 family acetyltransferase	Acetyltransferase	134.443	123.573	138.383	94.946	93.281	141.039
AMK58_06385	K01609 indole-3-glycerol phosphate synthase [EC:4.1.1.48] | (GenBank) indole-3-glycerol phosphate sy	Biosynthesis: Amino Acid (IAA)	57.085	56.842	44.063	139.780	37.602	149.266
AMK58_06390	K00766 anthranilate phosphoribosyltransferase [EC:2.4.2.18] | (GenBank) anthranilate phosphoribosylt	Biosynthesis: Amino Acid (IAA)	47.799	47.146	41.481	133.796	30.792	113.420
AMK58_06395	K01658 anthranilate synthase component II [EC:4.1.3.27] | (GenBank) anthranilate synthase	Biosynthesis: Amino Acid (IAA)	141.356	139.531	147.454	291.715	96.946	204.593
AMK58_06400	no KO assigned | (GenBank) hypothetical protein	Hypothetical	181.211	178.843	188.561	190.828	184.462	209.917
AMK58_06405	K09798 uncharacterized protein | (GenBank) hypothetical protein	Hypothetical	3.456	3.037	5.896	55.151	10.056	43.456
AMK58_06410	K07213 copper chaperone | (GenBank) heavy metal transporter	Chaperone	81.886	80.373	111.226	1186.229	186.060	484.875
AMK58_06415	no KO assigned | (GenBank) hypothetical protein	Hypothetical	6.221	8.158	9.954	31.909	12.450	27.012
AMK58_06420	K01657 anthranilate synthase component I [EC:4.1.3.27] | (GenBank) anthranilate synthase	Biosynthesis: Amino Acid (IAA)	13.613	17.701	14.999	86.671	22.772	62.521
AMK58_06425	K03770 peptidyl-prolyl cis-trans isomerase D [EC:5.2.1.8] | (GenBank) peptidylprolyl isomerase	Chaperone	70.771	71.301	75.221	158.146	34.763	113.519
AMK58_06430	K01803 triosephosphate isomerase (TIM) [EC:5.3.1.1] | (GenBank) triosephosphate isomerase	Metabolism	305.890	300.441	304.852	513.374	148.334	386.558
AMK58_06435	K03075 preprotein translocase subunit SecG | (GenBank) preprotein translocase subunit SecG	Secretion System	678.819	490.926	603.251	447.594	189.631	377.267
AMK58_06440	K01937 CTP synthase [EC:6.3.4.2] | (GenBank) CTP synthetase	Metabolism: Nucleotides	91.196	94.138	97.664	31.262	132.083	35.976
AMK58_06445	K01061 carboxymethylenebutenolidase [EC:3.1.1.45] | (GenBank) carboxymethylenebutenolidase	Metabolism	429.218	334.254	382.248	188.173	500.177	413.262
AMK58_06450	K01627 2-dehydro-3-deoxyphosphooctonate aldolase (KDO 8-P synthase) [EC:2.5.1.55] | (GenBank) 2-de	Biosynthesis: Lipopolysaccharide	495.120	469.133	498.432	113.608	291.746	244.065
AMK58_06455	no KO assigned | (GenBank) DNA-binding protein	No COG	280.849	279.103	269.399	299.562	272.186	350.907
AMK58_06460	K06139 pyrroloquinoline quinone biosynthesis protein E | (GenBank) pyrroloquinoline quinone biosynth	Biosynthesis: quinone	15.421	13.722	20.382	69.152	96.999	73.528
AMK58_06465	K06138 pyrroloquinoline quinone biosynthesis protein D | (GenBank) pyrroloquinoline quinone biosynth	Biosynthesis: quinone	266.112	318.980	288.597	685.911	798.130	815.125
AMK58_06470	K06137 pyrroloquinoline-quinone synthase [EC:1.3.3.11] | (GenBank) pyrroloquinoline quinone biosynth	Biosynthesis: quinone	24.932	28.860	32.002	61.368	24.521	38.290
AMK58_06475	K06136 pyrroloquinoline quinone biosynthesis protein B | (GenBank) pyrroloquinoline quinone biosynth	Biosynthesis: quinone	149.977	147.005	152.007	78.651	99.363	139.190
AMK58_06480	no KO assigned | (GenBank) pyrroloquinoline quinone biosynthesis protein PqqA	Biosynthesis: quinone	2709.983	3009.516	3514.681	2176.494	3472.897	1094.976
AMK58_06485	K01689 enolase [EC:4.2.1.11] | (GenBank) eno; enolase	Metabolism: Carbohydrate	327.129	323.755	287.617	7.107	293.058	28.990
AMK58_06490	no KO assigned | (GenBank) hypothetical protein	Hypothetical	929.042	906.190	926.161	141.062	345.941	194.923
AMK58_06495	K04075 tRNA(Ile)-lysidine synthase [EC:6.3.4.19] | (GenBank) tRNA(Ile)-lysidine synthetase	tRNA synthesis/modification	60.849	65.469	50.486	68.144	55.779	108.552
AMK58_06500	K03798 cell division protease FtsH [EC:3.4.24.-] | (GenBank) cell division protein FtsH	Cell Cycle/Shape/Homeostatsis	6.685	9.331	11.821	120.008	31.952	74.846
AMK58_06505	no KO assigned | (GenBank) hypothetical protein	Hypothetical	4771.994	4306.672	4558.240	427.200	9631.880	693.475
AMK58_06510	no KO assigned | (GenBank) hypothetical protein	Hypothetical	91.479	73.943	86.979	421.683	464.531	956.538
AMK58_06515	no KO assigned | (GenBank) peroxiredoxin	Cell Cycle/Shape/Homeostatsis	21.661	31.332	21.896	64.141	45.877	135.738
AMK58_06520	K01745 histidine ammonia-lyase [EC:4.3.1.3] | (GenBank) histidine ammonia-lyase	Biosynthesis: Amino Acid	55.661	64.425	49.606	30.695	126.200	83.228
AMK58_06525	no KO assigned | (GenBank) ABC transporter ATP-binding protein	Membrane Transport	17.174	16.673	22.724	131.225	122.580	86.392
AMK58_06530	no KO assigned | (GenBank) protein secretion protein	Secretion System	15.379	16.464	23.582	294.959	66.448	195.359
AMK58_06535	no KO assigned | (GenBank) regulator	Signal Transduction	32.748	27.556	41.004	482.851	218.008	509.170
AMK58_06540	no KO assigned | (GenBank) hypothetical protein	Hypothetical	316.721	418.733	433.473	123.252	32.857	121.972
AMK58_06545	no KO assigned | (GenBank) hypothetical protein	Hypothetical	11.225	14.846	11.325	82.872	18.396	68.490
AMK58_06550	no KO assigned | (GenBank) esterase	Esterase	747.870	669.568	626.660	277.795	1234.959	468.510
AMK58_06555	K03980 putative peptidoglycan lipid II flippase | (GenBank) multidrug transporter MurJ	Cell Wall	52.380	54.100	48.764	76.753	43.522	100.760
AMK58_06560	K01011 thiosulfate/3-mercaptopyruvate sulfurtransferase [EC:2.8.1.1 2.8.1.2] | (GenBank) sulfurtrans	Metabolism	51.417	61.781	60.905	460.392	68.612	233.995
AMK58_06565	K02417 flagellar motor switch protein FliN/FliY | (GenBank) flagellar motor switch protein FliN	Motility	171.288	199.063	267.272	832.303	150.863	614.872
AMK58_06570	no KO assigned | (GenBank) hypothetical protein	Hypothetical	125.798	194.947	185.742	100.647	216.205	277.887
AMK58_06575	K01751 diaminopropionate ammonia-lyase [EC:4.3.1.15] | (GenBank) PLP-dependent lyase/thiolase	Lyase	9.230	7.040	17.179	26.044	42.936	53.176
AMK58_06580	no KO assigned | (GenBank) aspartate aminotransferase	tRNA synthesis/modification	58.898	70.377	68.175	254.530	42.025	113.789
AMK58_06585	no KO assigned | (GenBank) hypothetical protein	Hypothetical	115.985	127.076	127.910	379.895	79.286	254.208
AMK58_06590	no KO assigned | (GenBank) hypothetical protein	Hypothetical	31.178	36.220	48.913	256.156	27.452	156.117
AMK58_06595	K02049 NitT/TauT family transport system ATP-binding protein | (GenBank) mannosyltransferase	Transport: NitT/TauT	1.699	2.538	3.635	168.256	73.697	125.532
AMK58_06600	K02050 NitT/TauT family transport system permease protein | (GenBank) ABC transporter permease	Transport: NitT/TauT	202.539	216.114	204.230	167.192	93.081	139.852
AMK58_06605	K02051 NitT/TauT family transport system substrate-binding protein | (GenBank) ABC transporter subst	Transport: NitT/TauT	360.732	303.526	285.653	51.995	97.400	177.540
AMK58_06610	no KO assigned | (GenBank) nitrogen fixation protein NifO	Nitrogen	3061.342	2411.185	2640.807	319.116	358.820	550.953
AMK58_06615	no KO assigned | (GenBank) hypothetical protein	Hypothetical	120.144	179.970	125.182	47.363	31.245	38.448
AMK58_06620	no KO assigned | (GenBank) hypothetical protein	Hypothetical	61.188	81.484	80.459	239.999	101.460	145.899
AMK58_06625	no KO assigned | (GenBank) histidine kinase	Signal Transduction	10.147	13.990	14.494	112.429	40.720	76.592
AMK58_06630	no KO assigned | (GenBank) regulator	Signal Transduction	23.062	22.964	28.140	269.881	50.981	193.235
AMK58_06635	K03406 methyl-accepting chemotaxis protein | (GenBank) chemotaxis protein	Chemotaxis	10.628	7.294	12.427	224.232	29.059	219.212
AMK58_06640	no KO assigned | (GenBank) alpha/beta hydrolase	Hydrolase	34.654	40.896	46.509	418.359	163.664	423.425
AMK58_06645	no KO assigned | (GenBank) hypothetical protein	Hypothetical	115.819	115.513	117.439	261.006	394.565	190.423
AMK58_06650	no KO assigned | (GenBank) cupin	No COG	68.319	96.132	83.267	325.785	279.302	224.784
AMK58_06655	no KO assigned | (GenBank) hypothetical protein	Hypothetical	23.431	26.902	29.890	90.699	36.460	41.831
AMK58_06660	no KO assigned | (GenBank) hypothetical protein	Hypothetical	165.836	173.167	219.424	1046.836	615.719	729.072
AMK58_06665	no KO assigned | (GenBank) histidine kinase	Signal Transduction	24.933	37.241	28.529	81.234	21.495	86.211
AMK58_06670	no KO assigned | (GenBank) AAA family ATPase	Biosynthesis	52.053	53.430	68.461	458.298	94.080	419.735
AMK58_06675	no KO assigned | (GenBank) hypothetical protein	Hypothetical	57.776	53.300	54.530	233.320	145.644	387.626
AMK58_06680	K03926 periplasmic divalent cation tolerance protein | (GenBank) dihydroorotate dehydrogenase	Membrane	51.889	64.298	76.015	311.500	91.593	178.919
AMK58_06685	no KO assigned | (GenBank) MFS transporter	Transport: MFS	21.557	28.229	24.896	47.733	33.525	68.457
AMK58_06690	no KO assigned | (GenBank) hypothetical protein	Hypothetical	1306.054	1516.896	1648.777	367.774	709.218	307.116
AMK58_06695	K01740 O-acetylhomoserine (thiol)-lyase [EC:2.5.1.49] | (GenBank) O-acetylhomoserine aminocarboxypro	Metabolism: Amino Acid	67.483	73.457	97.450	105.484	54.859	50.652

AMK58_06700	K06929 uncharacterized protein | (GenBank) hypothetical protein	Hypothetical	1508.186	1121.968	1199.766	511.245	297.097	563.295
AMK58_06705	no KO assigned | (GenBank) enoyl-CoA hydratase	Hydratase	91.069	69.019	82.309	85.414	33.560	91.123
AMK58_06710	no KO assigned | (GenBank) thioesterase	Esterase	273.768	256.476	277.410	249.305	102.287	250.521
AMK58_06715	K02871 large subunit ribosomal protein L13 | (GenBank) 50S ribosomal protein L13	Ribosome	195.022	241.119	210.680	156.954	80.778	365.299
AMK58_06720	K02996 small subunit ribosomal protein S9 | (GenBank) 30S ribosomal protein S9	Ribosome	1142.314	933.024	964.806	103.423	1308.523	235.464
AMK58_06725	K00145 N-acetyl-gamma-glutamyl-phosphate reductase [EC:1.2.1.38] | (GenBank) argC; N-acetyl-gamma-	Biosynthesis: Amino Acid	191.673	155.250	176.013	139.082	289.505	115.575
AMK58_06730	no KO assigned | (GenBank) carbonic anhydrase	Enzyme	187.328	154.706	168.797	300.622	194.831	439.777
AMK58_06735	K03286 OmpA-OmpF porin, OOP family | (GenBank) hypothetical protein	Transport: Ion	287.038	290.326	247.271	43.029	156.692	196.663
AMK58_06740	K03821 polyhydroxyalkanoate synthase [EC:2.3.1.-] | (GenBank) poly(3-hydroxyalkanoate) synthetase	Metabolism: Carbohydrate (cyst)	1080.929	1174.247	1380.404	63.883	125.886	68.859
AMK58_06745	no KO assigned | (GenBank) hypothetical protein	Hypothetical	4040.412	3408.539	3194.841	283.006	773.944	916.585
AMK58_06750	no KO assigned | (GenBank) MarR family transcriptional regulator	Transcription	180.060	166.928	190.255	269.554	117.694	167.750
AMK58_06755	K07552 MFS transporter, DHA1 family, bicyclomycin/chloramphenicol resistance protein | (GenBank) mu	Transport: MFS	4.513	6.222	5.859	91.963	9.463	28.651
AMK58_06760	no KO assigned | (GenBank) hypothetical protein	Hypothetical	54.667	56.992	63.664	450.189	67.016	221.137
AMK58_06765	K14261 alanine-synthesizing transaminase [EC:2.6.1.-] | (GenBank) aminotransferase	tRNA synthesis/modification	99.234	89.072	80.809	54.698	18.466	95.011
AMK58_06770	K00003 homoserine dehydrogenase [EC:1.1.1.3] | (GenBank) homoserine dehydrogenase	Biosynthesis: Amino Acid	513.920	397.071	397.196	101.924	90.000	225.569
AMK58_06775	K11532 fructose-1,6-bisphosphatase II / sedoheptulose-1,7-bisphosphatase [EC:3.1.3.11 3.1.3.37] | (G	Metabolism	356.331	324.237	316.637	189.049	84.107	147.020
AMK58_06780	K00005 glycerol dehydrogenase [EC:1.1.1.6] | (GenBank) gldA; glycerol dehydrogenase	Metabolism	441.391	415.333	451.726	203.326	80.634	143.833
AMK58_06785	K07462 single-stranded-DNA-specific exonuclease [EC:3.1.-.-] | (GenBank) single-stranded-DNA-specifi	DNA Repair and Replication	62.488	58.672	69.455	133.173	32.738	96.864
AMK58_06790	K14224 tRNA Glu | (GenBank) tRNA-Glu	tRNA synthesis/modification	454.030	599.624	633.154	4652.174	560.489	3174.573
AMK58_06795	K01153 type I restriction enzyme, R subunit [EC:3.1.21.3] | (GenBank) restriction endonuclease	DNA Repair and Replication	13.502	15.956	20.221	17.557	9.117	27.746
AMK58_06800	K03427 type I restriction enzyme M protein [EC:2.1.1.72] | (GenBank) DNA methyltransferase	DNA Repair and Replication	321.454	333.895	263.716	93.663	147.388	362.143
AMK58_06805	no KO assigned | (GenBank) hypothetical protein	Hypothetical	1071.915	1003.053	882.579	595.402	352.028	1780.348
AMK58_06810	no KO assigned | (GenBank) hypothetical protein	Hypothetical	144.911	131.862	108.643	34.296	14.176	151.798
AMK58_06815	no KO assigned | (GenBank) hypothetical protein	Hypothetical	312.611	222.343	237.000	118.316	28.260	507.974
AMK58_06820	no KO assigned | (GenBank) hypothetical protein	Hypothetical	128.413	131.519	125.584	205.014	363.504	1020.633
AMK58_06825	no KO assigned | (GenBank) hypothetical protein	Hypothetical	46.018	48.518	44.924	31.976	190.033	137.142
AMK58_06830	no KO assigned | (GenBank) hypothetical protein	Hypothetical	60.414	45.857	71.096	80.927	254.931	252.779
AMK58_06835	no KO assigned | (GenBank) hypothetical protein	Hypothetical	56.657	68.008	54.177	44.272	18.248	156.290
AMK58_06840	no KO assigned | (GenBank) hypothetical protein	Hypothetical	378.114	370.456	313.224	151.557	156.904	587.508
AMK58_06845	no KO assigned | (GenBank) hypothetical protein	Hypothetical	276.887	234.229	249.836	80.707	17.498	235.633
AMK58_06850	no KO assigned | (GenBank) hypothetical protein	Hypothetical	98.848	118.812	132.359	704.372	554.892	1913.466
AMK58_06855	K08177 MFS transporter, OFA family, oxalate/formate antiporter | (GenBank) oxalate/formate MFS antip	Transport: MFS	15.623	20.001	22.193	116.345	25.923	115.029
AMK58_06860	no KO assigned | (GenBank) acetoin utilization protein	No COG	14.190	19.445	18.568	33.309	34.389	71.708
AMK58_06865	no KO assigned | (GenBank) GCN5 family acetyltransferase	Acetyltransferase	123.582	101.839	95.575	354.929	137.220	518.114
AMK58_06870	K00077 2-dehydropantoate 2-reductase [EC:1.1.1.169] | (GenBank) 2-dehydropantoate 2-reductase	Metabolism: Co-Factors and Vitamins	9.087	8.460	11.220	134.997	35.151	160.198
AMK58_06875	no KO assigned | (GenBank) histidine kinase	Signal Transduction	48.324	47.589	46.199	82.516	80.880	292.246
AMK58_06880	K00324 NAD(P) transhydrogenase subunit alpha [EC:1.6.1.2] | (GenBank) NAD(P) transhydrogenase sul	Energy	18.095	12.754	18.365	282.702	50.964	276.951
AMK58_06885	K00324 NAD(P) transhydrogenase subunit alpha [EC:1.6.1.2] | (GenBank) NAD(P) transhydrogenase sul	Energy	14.473	6.652	15.614	177.472	63.823	196.407
AMK58_06890	K00325 NAD(P) transhydrogenase subunit beta [EC:1.6.1.2] | (GenBank) NAD synthetase	Energy	1.215	1.037	2.311	73.361	20.292	87.723
AMK58_06895	no KO assigned | (GenBank) hypothetical protein	Hypothetical	2.070	2.061	1.804	17.097	15.121	41.741
AMK58_06900	no KO assigned | (GenBank) GntR family transcriptional regulator	Transcription	8.247	7.338	7.007	146.672	61.341	95.836
AMK58_06905	K01577 oxalyl-CoA decarboxylase [EC:4.1.1.8] | (GenBank) oxalyl-CoA decarboxylase	Metabolism: Carbohydrate	19.973	23.865	30.122	75.558	15.328	71.270
AMK58_06910	no KO assigned | (GenBank) formyl-coenzyme A transferase	Stress Response	12.032	13.018	13.512	25.254	13.418	23.368
AMK58_06915	K01903 succinyl-CoA synthetase beta subunit [EC:6.2.1.5] | (GenBank) sucC; succinyl-CoA synthetase s	Metabolism	41.774	36.258	47.509	163.947	34.791	102.703
AMK58_06920	K01902 succinyl-CoA synthetase alpha subunit [EC:6.2.1.5] | (GenBank) succinate--CoA ligase	Metabolism	49.342	34.681	54.931	165.148	94.457	216.209
AMK58_06925	no KO assigned | (GenBank) MFS transporter	Transport: MFS	7.750	4.810	8.804	108.663	136.659	149.133
AMK58_06930	no KO assigned | (GenBank) oxalate/formate MFS antiporter	Transport: MFS	3.722	5.852	6.054	51.962	60.339	34.517
AMK58_06935	no KO assigned | (GenBank) hypothetical protein	Hypothetical	46.764	61.236	73.091	1935.933	95.049	1033.000
AMK58_06940	K03147 phosphomethylpyrimidine synthase [EC:4.1.99.17] | (GenBank) phosphomethylpyrimidine syntha	Metabolism: Co-Factors and Vitamins	47.523	37.699	37.561	52.917	20.466	104.956
AMK58_06945	K08177 MFS transporter, OFA family, oxalate/formate antiporter | (GenBank) oxalate/formate MFS antip	Transport: MFS	5.686	6.682	9.748	13.859	12.153	38.288
AMK58_06950	K11529 glycerate 2-kinase [EC:2.7.1.165] | (GenBank) hydroxypyruvate reductase	Metabolism	1409.472	1478.784	1346.609	197.371	250.851	261.963
AMK58_06955	no KO assigned | (GenBank) 2-hydroxy-3-oxopropionate reductase	Metabolism: Carbohydrate	524.791	450.406	492.137	294.040	88.333	260.357
AMK58_06960	K01816 hydroxypyruvate isomerase [EC:5.3.1.22] | (GenBank) hydroxypyruvate isomerase	Metabolism: Carbohydrate	89.061	76.143	106.608	364.613	128.589	292.351
AMK58_06965	K01608 tartronate-semialdehyde synthase [EC:4.1.1.47] | (GenBank) glyoxylate carboligase	Metabolism: Carbohydrate	14.091	13.895	15.609	103.634	36.368	139.861
AMK58_06970	K00788 thiamine-phosphate pyrophosphorylase [EC:2.5.1.3] | (GenBank) thiamine-phosphate pyrophosph	Metabolism: Co-Factors and Vitamins	38.418	35.936	43.539	366.218	87.457	326.554
AMK58_06975	no KO assigned | (GenBank) GntR family transcriptional regulator	Transcription	23.162	27.257	28.529	338.475	105.522	608.860
AMK58_06980	K00873 pyruvate kinase [EC:2.7.1.40] | (GenBank) pyruvate kinase	Metabolism	7.360	10.376	9.121	78.877	21.288	61.656
AMK58_06985	no KO assigned | (GenBank) hypothetical protein	Hypothetical	448.443	408.268	376.850	74.308	287.368	210.226
AMK58_06990	K01006 pyruvate, orthophosphate dikinase [EC:2.7.9.1] | (GenBank) pyruvate kinase	Metabolism	25.508	23.414	29.286	130.320	61.980	147.143
AMK58_06995	no KO assigned | (GenBank) histidine kinase	Signal Transduction	99.550	155.923	133.282	435.379	121.274	336.809
AMK58_07000	K07090 uncharacterized protein | (GenBank) hypothetical protein	Hypothetical	69.093	90.661	69.532	484.828	82.745	193.553
AMK58_07005	no KO assigned | (GenBank) acetyltransferase	Acetyltransferase	23.839	17.490	26.642	177.872	41.604	256.145
AMK58_07010	no KO assigned | (GenBank) FmdB family transcriptional regulator	Transcription	85.434	103.337	122.096	610.830	119.008	575.443
AMK58_07015	no KO assigned | (GenBank) Fis family transcriptional regulator	Transcription	49.937	60.045	60.546	47.120	31.331	80.089
AMK58_07020	K00285 D-amino-acid dehydrogenase [EC:1.4.5.1] | (GenBank) amino acid dehydrogenase	Metabolism: Amino Acid	2.396	3.303	3.855	43.447	10.046	23.838
AMK58_07025	no KO assigned | (GenBank) hypothetical protein	Hypothetical	8.401	10.826	12.217	311.353	126.095	197.503
AMK58_07030	K05520 protease I [EC:3.2.-.-] | (GenBank) glutamine amidotransferase	tRNA synthesis/modification	1892.612	1608.298	1391.673	560.999	323.014	522.358

AMK58_07035	K07684 two-component system, NarL family, nitrate/nitrite response regulator NarL | (GenBank) two-co	Nitrogen	112.674	72.008	94.283	185.670	316.524	183.221
AMK58_07040	no KO assigned | (GenBank) two-component system response regulator	Signal Transduction	16.040	22.669	13.611	142.779	1027.097	445.720
AMK58_07045	K13481 xanthine dehydrogenase small subunit [EC:1.17.1.4] | (GenBank) FAD-binding molybdopterin del	Metabolism: Nucleotide	6.272	7.396	7.533	58.153	162.533	61.530
AMK58_07050	K13482 xanthine dehydrogenase large subunit [EC:1.17.1.4] | (GenBank) hypothetical protein	Metabolism: Nucleotide	4.088	3.091	4.378	64.805	59.090	112.701
AMK58_07055	K03190 urease accessory protein | (GenBank) urease accessory protein	Nitrogen	240.687	272.569	313.240	310.856	234.711	294.369
AMK58_07060	K01430 urease subunit gamma [EC:3.5.1.5] | (GenBank) ureA; urease subunit gamma	Nitrogen	913.855	1024.157	1104.078	1179.728	462.627	941.509
AMK58_07065	K01429 urease subunit beta [EC:3.5.1.5] | (GenBank) ureB; urease subunit beta	Nitrogen	98.126	148.926	174.559	444.156	73.828	172.807
AMK58_07070	K01428 urease subunit alpha [EC:3.5.1.5] | (GenBank) ureC; urease subunit alpha	Nitrogen	15.931	13.113	13.935	26.557	11.938	34.731
AMK58_07075	K01776 glutamate racemase [EC:5.1.1.3] | (GenBank) glutamate racemase	Metabolism: Amino Acid	29.842	27.168	29.455	102.604	22.203	51.655
AMK58_07080	K03187 urease accessory protein | (GenBank) urease accessory protein UreE	Nitrogen	329.803	341.585	295.792	439.535	241.235	560.374
AMK58_07085	K03188 urease accessory protein | (GenBank) urease accessory protein UreF	Nitrogen	32.398	24.333	42.598	251.215	36.416	154.328
AMK58_07090	K03189 urease accessory protein | (GenBank) urease accessory protein UreG	Nitrogen	24.632	23.772	29.185	165.485	25.557	147.233
AMK58_07095	no KO assigned | (GenBank) hypothetical protein	Hypothetical	11.511	9.943	11.472	129.303	11.848	61.472
AMK58_07100	no KO assigned | (GenBank) hypothetical protein	Hypothetical	253.462	222.852	209.598	109.762	103.101	281.241
AMK58_07105	K00208 enoyl-[acyl-carrier protein] reductase I [EC:1.3.1.9 1.3.1.10] | (GenBank) short-chain dehydr	Metabolism: Lipid	28.189	28.529	32.661	191.280	309.067	125.411
AMK58_07110	K07216 hemerythrin | (GenBank) hypothetical protein	No COG	19.443	14.080	31.091	354.362	346.690	185.204
AMK58_07115	no KO assigned | (GenBank) N-formylglutamate deformylase	Cell Wall	79.689	64.006	73.118	59.566	55.097	227.397
AMK58_07120	no KO assigned | (GenBank) ArsR family transcriptional regulator	Transcription	360.465	350.343	289.929	106.738	100.422	392.784
AMK58_07125	K09950 uncharacterized protein | (GenBank) hypothetical protein	Hypothetical	148.837	112.112	128.073	253.781	130.603	545.616
AMK58_07130	no KO assigned | (GenBank) hypothetical protein	Hypothetical	71.860	73.959	67.368	34.379	51.586	86.419
AMK58_07135	no KO assigned | (GenBank) glycosyl transferase	Glycosyl Transferase	259.297	296.713	270.301	50.841	72.513	113.530
AMK58_07140	no KO assigned | (GenBank) hypothetical protein	Hypothetical	81.978	89.666	89.577	361.370	91.863	297.632
AMK58_07145	no KO assigned | (GenBank) glycosyl transferase	Glycosyl Transferase	22.879	28.866	30.131	265.170	59.102	129.268
AMK58_07150	no KO assigned | (GenBank) transcriptional regulator	Transcription	17.333	18.548	29.518	684.438	86.367	314.019
AMK58_07155	K13683 putative colanic acid biosynthesis glycosyltransferase [EC:2.4.-.-] | (GenBank) hypothetical	Biosynthesis: Polysaccharide	28.702	37.430	37.680	129.788	58.466	220.351
AMK58_07160	no KO assigned | (GenBank) sugar transporter	Transport: Sugar	26.746	22.590	26.339	14.235	14.137	43.808
AMK58_07165	no KO assigned | (GenBank) pseudogene	Pseudogene	15.495	23.681	21.585	441.184	61.566	519.581
AMK58_07170	K12251 N-carbamoylputrescine amidase [EC:3.5.1.53] | (GenBank) N-carbamoylputrescine amidase	Metabolism: Amino Acid	29.560	32.257	38.501	695.935	110.301	482.747
AMK58_07175	no KO assigned | (GenBank) transcriptional regulator	Transcription	60.883	84.047	67.098	151.065	57.030	180.487
AMK58_07180	no KO assigned | (GenBank) hypothetical protein	Hypothetical	44.791	46.899	40.656	97.980	197.756	179.666
AMK58_07185	K02039 phosphate transport system protein | (GenBank) PhoU family transcriptional regulator	Transport: Ion	43.423	51.886	49.221	68.889	50.724	101.058
AMK58_07190	K06197 cation transport regulator | (GenBank) hypothetical protein	Transport: Ion	266.365	225.044	274.751	814.534	454.249	1191.555
AMK58_07195	no KO assigned | (GenBank) hypothetical protein	Hypothetical	527.946	555.466	631.875	115.332	298.901	231.192
AMK58_07200	K04771 serine protease Do [EC:3.4.21.107] | (GenBank) serine protease	Defense	164.501	187.537	215.577	33.934	49.786	44.208
AMK58_07205	K00721 dolichol-phosphate mannosyltransferase [EC:2.4.1.83] | (GenBank) glycosyl transferase	Glycosyl Transferase	457.819	365.013	367.198	156.203	742.685	139.043
AMK58_07210	no KO assigned | (GenBank) lipid A biosynthesis	Biosynthesis: Lipid	50.074	49.117	87.760	983.127	956.853	920.681
AMK58_07215	no KO assigned | (GenBank) glycosyl transferase	Glycosyl Transferase	37.321	31.112	31.359	89.716	32.194	84.437
AMK58_07220	no KO assigned | (GenBank) hypothetical protein	Hypothetical	32.160	16.954	21.885	48.571	9.569	114.024
AMK58_07225	K00847 fructokinase [EC:2.7.1.4] | (GenBank) hypothetical protein	Metabolism: Carbohydrate	80.966	53.748	67.955	341.611	31.741	275.634
AMK58_07230	no KO assigned | (GenBank) hypothetical protein	Hypothetical	18.793	13.804	27.535	83.569	65.969	53.278
AMK58_07235	K01997 branched-chain amino acid transport system permease protein | (GenBank) branched-chain amino	Transport: Amino Acid	9.622	17.565	16.391	191.723	68.047	147.958
AMK58_07240	K01998 branched-chain amino acid transport system permease protein | (GenBank) ABC transporter perme	Transport: Amino Acid	164.623	205.856	163.047	110.688	186.657	130.390
AMK58_07245	K01995 branched-chain amino acid transport system ATP-binding protein | (GenBank) ABC transporter A'	Transport: Amino Acid	987.888	852.497	907.944	58.094	93.343	190.161
AMK58_07250	K01996 branched-chain amino acid transport system ATP-binding protein | (GenBank) ABC transporter A'	Transport: Amino Acid	1321.435	1013.308	1315.853	229.934	138.582	341.230
AMK58_07255	no KO assigned | (GenBank) hypothetical protein	Hypothetical	1287.429	1032.791	1150.870	246.432	163.312	391.696
AMK58_07260	K01999 branched-chain amino acid transport system substrate-binding protein | (GenBank) ABC transpor	Transport: Amino Acid	322.866	300.248	324.027	96.045	51.174	117.368
AMK58_07265	no KO assigned | (GenBank) hypothetical protein	Hypothetical	870.105	734.837	906.790	186.642	205.760	298.495
AMK58_07270	no KO assigned | (GenBank) hypothetical protein	Hypothetical	18430.273	21854.715	18453.672	608.197	1389.915	1084.234
AMK58_07275	no KO assigned | (GenBank) hypothetical protein	Hypothetical	13.492	14.592	16.256	37.392	203.944	70.964
AMK58_07280	no KO assigned | (GenBank) two-component system response regulator	Signal Transduction	55.000	82.990	50.331	50.542	20.467	111.399
AMK58_07285	no KO assigned | (GenBank) histidine kinase	Signal Transduction	286.229	291.516	359.275	145.236	317.551	134.586
AMK58_07290	K02572 ferredoxin-type protein NapF | (GenBank) hypothetical protein	Ferredoxin	390.971	346.608	363.744	140.786	229.657	500.536
AMK58_07295	no KO assigned | (GenBank) hypothetical protein	Hypothetical	182.024	167.307	184.556	359.310	200.282	688.893
AMK58_07300	K06177 tRNA pseudouridine32 synthase / 23S rRNA pseudouridine746 synthase [EC:5.4.99.28 5.4.99.29	tRNA synthesis/modification	13.862	14.677	16.017	71.691	35.108	146.439
AMK58_07305	no KO assigned | (GenBank) oxidoreductase	Oxidoreductase	13.453	17.295	15.058	95.350	458.046	171.010
AMK58_07310	K00764 amidophosphoribosyltransferase [EC:2.4.2.14] | (GenBank) amidophosphoribosyltransferase	Metabolism	16.962	13.978	17.002	82.940	108.856	86.236
AMK58_07315	K03558 membrane protein required for colicin V production | (GenBank) colicin V production protein	Membrane	58.687	52.646	55.968	176.115	174.920	171.918
AMK58_07320	K04485 DNA repair protein RadA/Sms | (GenBank) DNA repair protein RadA	DNA Repair and Replication	235.841	195.167	217.419	160.334	439.462	217.808
AMK58_07325	K01265 methionyl aminopeptidase [EC:3.4.11.18] | (GenBank) methionine aminopeptidase	Peptidase	45.476	41.934	55.196	152.784	303.909	211.251
AMK58_07330	K01515 ADP-ribose pyrophosphatase [EC:3.6.1.13] | (GenBank) NUDIX hydrolase	Metabolism: Nucleotide	60.025	59.974	56.683	595.230	163.036	356.945
AMK58_07335	no KO assigned | (GenBank) hypothetical protein	Hypothetical	782.840	776.203	671.790	408.057	557.423	844.329
AMK58_07340	K02065 phospholipid/cholesterol/gamma-HCH transport system ATP-binding protein | (GenBank) ABC tr	Transport: phospholipid	105.023	115.993	95.932	82.936	93.994	186.974
AMK58_07345	K02066 phospholipid/cholesterol/gamma-HCH transport system permease protein | (GenBank) ABC transp	Transport: phospholipid	91.624	87.510	80.450	24.839	51.320	68.489
AMK58_07350	K01775 alanine racemase [EC:5.1.1.1] | (GenBank) alanine racemase	Metabolism: Amino Acid	66.234	56.621	72.696	61.337	67.149	149.199
AMK58_07355	K02314 replicative DNA helicase [EC:3.6.4.12] | (GenBank) replicative DNA helicase	DNA Repair and Replication	39.332	34.446	40.551	82.310	55.574	75.506
AMK58_07360	K00574 cyclopropane-fatty-acyl-phospholipid synthase [EC:2.1.1.79] | (GenBank) cyclopropane-fatty-ac	Biosynthesis: Lipid	70.386	62.875	75.920	116.660	78.248	122.745
AMK58_07365	K02939 large subunit ribosomal protein L9 | (GenBank) 50S ribosomal protein L9	Ribosome	304.929	320.216	314.090	322.369	166.336	568.675

AMK58_07370	no KO assigned | (GenBank) hypothetical protein	Hypothetical	10.121	11.338	17.563	212.689	71.388	327.893
AMK58_07375	K02963 small subunit ribosomal protein S18 | (GenBank) 30S ribosomal protein S18	Ribosome	1910.143	1438.762	1632.328	370.711	1879.881	535.851
AMK58_07380	K02990 small subunit ribosomal protein S6 | (GenBank) 30S ribosomal protein S6	Ribosome	1837.959	1458.272	1748.336	375.101	2083.344	505.982
AMK58_07385	K00645 [acyl-carrier-protein] S-malonyltransferase [EC:2.3.1.39] | (GenBank) ACP S-malonyltransferas	Metabolism: Lipid	368.411	264.550	332.125	39.657	353.245	37.892
AMK58_07390	K00059 3-oxoacyl-[acyl-carrier protein] reductase [EC:1.1.1.100] | (GenBank) 3-oxoacyl-ACP synthase	Metabolism: Lipid	770.416	601.816	584.174	39.558	623.115	82.301
AMK58_07395	K02078 acyl carrier protein | (GenBank) acyl carrier protein	Metabolism: Lipid	586.708	593.754	619.244	551.432	326.624	494.922
AMK58_07400	K09458 3-oxoacyl-[acyl-carrier-protein] synthase II [EC:2.3.1.179] | (GenBank) 3-oxoacyl-ACP synthas	Metabolism: Lipid	134.496	112.827	120.679	59.584	103.659	72.802
AMK58_07405	K07082 UPF0755 protein | (GenBank) aminodeoxychorismate lyase	Lyase	688.265	750.066	618.219	35.286	303.290	93.670
AMK58_07410	no KO assigned | (GenBank) hypothetical protein	Hypothetical	4438.878	4019.700	3932.725	646.609	3617.811	995.655
AMK58_07415	K08309 soluble lytic murein transglycosylase [EC:3.2.1.-] | (GenBank) lytic murein transglycosylase	Cell Wall	57.487	71.047	69.013	36.977	99.455	92.787
AMK58_07420	K01714 4-hydroxy-tetrahydrodipicolinate synthase [EC:4.3.3.7] | (GenBank) 4-hydroxy-tetrahydrodipico	Biosynthesis: Co-Factors and Vitamins	79.473	108.173	111.308	23.513	25.945	51.905
AMK58_07425	K03664 SsrA-binding protein | (GenBank) SsrA-binding protein	Translation	332.346	304.230	325.011	822.177	314.811	687.432
AMK58_07430	K00564 16S rRNA (guanine1207-N2)-methyltransferase [EC:2.1.1.172] | (GenBank) rRNA (guanine-N2)	Ribosome	660.675	610.880	654.131	123.255	254.067	201.082
AMK58_07435	no KO assigned | (GenBank) transcriptional regulator	Transcription	250.668	244.456	223.599	39.471	138.919	67.206
AMK58_07440	K06910 uncharacterized protein | (GenBank) phosphatidylethanolamine-binding protein	No COG	130.506	90.419	124.504	211.547	150.393	163.930
AMK58_07445	K01698 porphobilinogen synthase [EC:4.2.1.24] | (GenBank) delta-aminolevulinic acid dehydratase	Metabolism: Co-Factors and Vitamens	36.179	37.095	40.530	134.195	98.513	134.635
AMK58_07450	no KO assigned | (GenBank) 3',5'-cyclic-nucleotide phosphodiesterase	phosphodiesterase	20.393	19.596	23.051	96.853	84.637	172.041
AMK58_07455	no KO assigned | (GenBank) diacylglycerol kinase	No COG	250.521	253.062	249.246	206.205	300.276	233.461
AMK58_07460	K01692 enoyl-CoA hydratase [EC:4.2.1.17] | (GenBank) enoyl-CoA hydratase	Metabolism	14.968	11.923	18.027	147.037	145.814	214.464
AMK58_07465	no KO assigned | (GenBank) MarR family transcriptional regulator	Transcription	43.168	51.886	58.531	380.145	156.191	402.181
AMK58_07470	K01995 branched-chain amino acid transport system ATP-binding protein | (GenBank) ABC transporter A	Transport: Amino Acid	334.717	294.700	308.507	171.625	97.929	284.238
AMK58_07475	K01996 branched-chain amino acid transport system ATP-binding protein | (GenBank) ABC transporter A	Transport: Amino Acid	2025.114	1920.944	2053.512	47.557	921.217	170.811
AMK58_07480	K01999 branched-chain amino acid transport system substrate-binding protein | (GenBank) ABC transpor	Transport: Amino Acid	693.365	828.733	635.665	99.581	181.794	204.211
AMK58_07485	K01997 branched-chain amino acid transport system permease protein | (GenBank) ABC transporter perme	Transport: Amino Acid	1010.729	1164.863	926.137	48.035	187.435	155.888
AMK58_07490	K01998 branched-chain amino acid transport system permease protein | (GenBank) ABC transporter perme	Transport: Amino Acid	2447.130	2642.093	2628.987	164.299	180.275	283.783
AMK58_07495	no KO assigned | (GenBank) hypothetical protein	Hypothetical	980.027	914.330	896.841	128.457	129.670	265.274
AMK58_07500	K00259 alanine dehydrogenase [EC:1.4.1.1] | (GenBank) alanine dehydrogenase	Metabolism: Amino Acid	320.269	349.072	313.877	84.354	70.418	158.422
AMK58_07505	no KO assigned | (GenBank) hypothetical protein	Hypothetical	458.034	565.161	463.724	131.293	160.336	322.949
AMK58_07510	no KO assigned | (GenBank) multifunctional 2',3'-cyclic-nucleotide 2'-phosphodiesterase/5'-nucleotid	Metabolism: Nucleotide	63.454	65.931	74.048	155.956	191.292	113.951
AMK58_07515	no KO assigned | (GenBank) glutamine amidotransferase	tRNA synthesis/modification	82.243	97.258	123.180	213.522	889.943	646.101
AMK58_07520	K07141 molybdenum cofactor cytidylyltransferase [EC:2.7.7.76] | (GenBank) 4-diphosphocytidyl-2C-meth	Biosynthesis: Co-Factors and Vitamins	80.491	80.822	83.463	186.348	80.592	238.408
AMK58_07525	K07402 xanthine dehydrogenase accessory factor | (GenBank) xanthine dehydrogenase	dehydrogenase	45.593	69.612	63.420	220.998	99.840	166.291
AMK58_07530	no KO assigned | (GenBank) xanthine dehydrogenase	dehydrogenase	101.857	126.300	124.986	768.195	416.920	371.025
AMK58_07535	K07161 uncharacterized protein | (GenBank) hypothetical protein	Hypothetical	66.389	59.074	77.226	121.234	191.211	129.952
AMK58_07540	no KO assigned | (GenBank) ATPase	No COG	18.083	23.486	25.291	64.242	86.163	30.862
AMK58_07545	K03519 carbon-monoxide dehydrogenase medium subunit [EC:1.2.7.4] | (GenBank) carbon monoxide deh	Metabolism: Carbon	22.844	32.756	32.002	280.306	52.383	108.033
AMK58_07550	K03520 carbon-monoxide dehydrogenase large subunit [EC:1.2.7.4] | (GenBank) carbon monoxide dehydr	Metabolism: Carbon	73.070	75.028	80.405	98.831	44.681	77.053
AMK58_07555	K03518 carbon-monoxide dehydrogenase small subunit [EC:1.2.7.4] | (GenBank) carbon monoxide dehyd	Metabolism: Carbon	574.011	689.948	790.576	323.395	335.587	305.698
AMK58_07560	K07080 uncharacterized protein | (GenBank) C4-dicarboxylate ABC transporter	Membrane Transport	1380.046	1296.494	1614.853	395.706	711.047	448.600
AMK58_07565	no KO assigned | (GenBank) C4-dicarboxylate ABC transporter	Membrane Transport	160.682	142.808	193.896	48.416	124.217	45.400
AMK58_07570	no KO assigned | (GenBank) hypothetical protein	Hypothetical	4599.253	5006.532	4612.613	194.112	608.312	513.612
AMK58_07575	no KO assigned | (GenBank) sodium:proton antiporter	Transport: Ion	996.414	936.947	981.391	178.073	106.841	265.127
AMK58_07580	no KO assigned | (GenBank) universal stress protein UspA	Stress Response	73.085	78.912	89.863	123.065	21.940	95.531
AMK58_07585	no KO assigned | (GenBank) hypothetical protein	Hypothetical	501.049	503.204	440.910	381.212	188.271	441.091
AMK58_07590	K04768 acetoin utilization protein AcuC | (GenBank) acetoin utilization protein	Metabolism	80.404	88.244	83.669	61.447	198.645	165.511
AMK58_07595	no KO assigned | (GenBank) hypothetical protein	Hypothetical	29.943	37.594	36.310	271.182	271.699	368.196
AMK58_07600	no KO assigned | (GenBank) hypothetical protein	Hypothetical	21.161	23.460	30.698	357.101	262.868	274.302
AMK58_07605	K09005 uncharacterized protein | (GenBank) hypothetical protein	Hypothetical	163.940	161.744	157.782	68.165	229.358	305.894
AMK58_07610	no KO assigned | (GenBank) hypothetical protein	Hypothetical	139.702	166.928	135.125	280.475	157.031	392.144
AMK58_07615	K14232 tRNA Pro | (GenBank) tRNA-Pro	tRNA synthesis/modification	423.762	556.427	439.813	638.347	347.212	1154.363
AMK58_07620	no KO assigned | (GenBank) oxidoreductase	Oxidoreductase	11.822	24.355	23.644	202.050	57.737	253.646
AMK58_07625	K14219 tRNA Arg | (GenBank) tRNA-Arg	tRNA synthesis/modification	136.799	197.279	104.654	393.880	98.977	309.106
AMK58_07630	no KO assigned | (GenBank) hypothetical protein	Hypothetical	455.753	649.844	506.336	75.819	220.183	224.083
AMK58_07635	K01496 phosphoribosyl-AMP cyclohydrolase [EC:3.5.4.19] | (GenBank) phosphoribosyl-AMP cyclohydr	Biosynthesis: Amino Acid	67.913	76.493	60.338	6.338	25.967	43.617
AMK58_07640	K01760 cystathionine beta-lyase [EC:4.4.1.8] | (GenBank) cystathionine beta-lyase	Biosynthesis: Amino Acid	11.296	15.338	15.232	29.576	226.476	92.368
AMK58_07645	K09969 general L-amino acid transport system substrate-binding protein | (GenBank) amino acid ABC tr	Transport: Amino Acid	39.530	28.284	39.048	31.391	59.732	111.721
AMK58_07650	K09970 general L-amino acid transport system permease protein | (GenBank) amino acid ABC transporter	Transport: Amino Acid	119.158	96.447	96.508	94.744	95.832	212.190
AMK58_07655	K09971 general L-amino acid transport system permease protein | (GenBank) amino acid ABC transporter	Transport: Amino Acid	4079.647	3537.267	3445.647	183.927	156.525	162.836
AMK58_07660	K09972 general L-amino acid transport system ATP-binding protein [EC:3.6.3.-] | (GenBank) ABC transp	Transport: Amino Acid	1181.453	1056.639	1196.822	272.397	66.383	325.683
AMK58_07665	K00817 histidinol-phosphate aminotransferase [EC:2.6.1.9] | (GenBank) histidinol phosphate aminotran	Metabolism: Amino Acid	747.074	632.770	706.614	73.775	45.239	148.659
AMK58_07670	no KO assigned | (GenBank) multidrug DMT transporter permease	Transport	440.641	426.128	418.241	83.813	109.274	200.414
AMK58_07675	no KO assigned | (GenBank) GNAT family acetyltransferase	Acetyltransferase	279.113	252.386	247.093	220.290	156.868	335.935
AMK58_07680	no KO assigned | (GenBank) acetyltransferase	Acetyltransferase	84.335	71.504	69.601	394.466	174.888	405.719
AMK58_07685	K01011 thiosulfate/3-mercaptopyruvate sulfurtransferase [EC:2.8.1.1 2.8.1.2] | (GenBank) sseA; 3-mer	Metabolism	36.068	32.042	30.086	86.391	84.399	105.556
AMK58_07690	K07050 misacylated tRNA(Ala) deacylase [EC:3.1.1.-] | (GenBank) Ala-tRNA(Pro) hydrolase	tRNA synthesis/modification	44.120	34.737	33.170	78.115	74.005	77.225
AMK58_07695	K01738 cysteine synthase A [EC:2.5.1.47] | (GenBank) cysteine synthase	Metabolism	82.765	60.460	65.635	239.971	120.114	120.760
AMK58_07700	K01515 ADP-ribose pyrophosphatase [EC:3.6.1.13] | (GenBank) nudF; ADP-ribose pyrophosphatase	Metabolism: Nucleotide	101.194	78.393	71.642	308.659	178.106	146.928

AMK58_07705	no KO assigned | (GenBank) serine protease	Protease	145.337	72.831	69.544	193.101	338.969	174.386
AMK58_07710	no KO assigned | (GenBank) hypothetical protein	Hypothetical	99.382	138.643	114.639	550.080	416.785	605.321
AMK58_07715	no KO assigned | (GenBank) hypothetical protein	Hypothetical	374.231	582.442	507.819	998.391	1593.056	1343.834
AMK58_07720	K14220 tRNA Asn | (GenBank) tRNA-Asn	tRNA synthesis/modification	1484.759	1361.051	1720.725	230.475	1022.490	821.341
AMK58_07725	K14222 tRNA Cys | (GenBank) tRNA-Cys	tRNA synthesis/modification	1979.736	2658.820	2230.818	140.269	956.331	884.503
AMK58_07730	K00573 protein-L-isoaspartate(D-aspartate) O-methyltransferase [EC:2.1.1.77] | (GenBank) protein-L-i	Methyltransferase	1.659	3.303	1.402	22.711	3.107	69.378
AMK58_07735	no KO assigned | (GenBank) sulfurtransferase	transferase	11.755	16.387	9.314	19.420	32.592	240.526
AMK58_07740	K12340 outer membrane protein | (GenBank) hypothetical protein	Membrane	410.541	395.975	374.964	43.459	106.372	103.317
AMK58_07745	K09991 uncharacterized protein | (GenBank) hypothetical protein	Hypothetical	376.498	368.643	332.736	45.652	142.948	95.637
AMK58_07750	K01873 valyl-tRNA synthetase [EC:6.1.1.9] | (GenBank) valine--tRNA ligase	tRNA synthesis/modification	213.361	206.012	190.436	99.032	88.527	91.615
AMK58_07755	K03406 methyl-accepting chemotaxis protein | (GenBank) hypothetical protein	Chemotaxis	55.624	59.818	49.421	76.912	15.561	48.828
AMK58_07760	no KO assigned | (GenBank) hypothetical protein	Hypothetical	750.838	764.988	685.744	361.904	355.082	632.426
AMK58_07765	K03642 rare lipoprotein A | (GenBank) hypothetical protein	Cell Wall	3.972	3.827	9.015	462.702	50.981	472.721
AMK58_07770	K07258 D-alanyl-D-alanine carboxypeptidase (penicillin-binding protein 5/6) [EC:3.4.16.4] | (GenBank	Cell Wall	27.701	29.752	35.118	173.860	52.012	154.118
AMK58_07775	K00943 dTMP kinase [EC:2.7.4.9] | (GenBank) thymidylate kinase	Signal Transduction	92.384	145.128	117.301	245.969	114.593	194.743
AMK58_07780	K02341 DNA polymerase III subunit delta' [EC:2.7.7.7] | (GenBank) DNA polymerase III subunit delta'	DNA Repair and Replication	193.785	215.261	223.037	120.009	113.715	203.972
AMK58_07785	K01874 methionyl-tRNA synthetase [EC:6.1.1.10] | (GenBank) methionine--tRNA ligase	tRNA synthesis/modification	49.257	44.772	60.640	120.358	37.555	48.715
AMK58_07790	K03424 TatD DNase family protein [EC:3.1.21.-] | (GenBank) LuxR family transcriptional regulator	Transcription	46.427	46.229	53.232	362.581	83.566	165.872
AMK58_07795	K06167 phosphoribosyl 1,2-cyclic phosphate phosphodiesterase [EC:3.1.4.55] | (GenBank) hydrolase	Metabolism: amino acids	295.713	288.101	288.362	249.639	428.127	260.174
AMK58_07800	no KO assigned | (GenBank) hypothetical protein	Hypothetical	59.350	63.825	59.252	76.005	115.012	128.340
AMK58_07805	no KO assigned | (GenBank) hypothetical protein	Hypothetical	61.525	63.230	59.572	79.203	114.839	213.417
AMK58_07810	K20074 PPM family protein phosphatase [EC:3.1.3.16] | (GenBank) protein phosphatase	phosphatases	115.135	120.104	104.259	76.503	209.019	276.649
AMK58_07815	no KO assigned | (GenBank) hypothetical protein	Hypothetical	201.280	189.232	204.494	289.471	1057.163	829.469
AMK58_07820	K10536 agmatine deiminase [EC:3.5.3.12] | (GenBank) agmatine deiminase	Metabolism: Amino Acid	72.621	107.401	88.292	133.640	682.473	272.891
AMK58_07825	K11074 putrescine transport system permease protein | (GenBank) spermidine/putrescine ABC transporte	Membrane Transport	204.259	221.170	218.919	72.731	908.430	148.347
AMK58_07830	K11075 putrescine transport system permease protein | (GenBank) spermidine/putrescine ABC transporte	Membrane Transport	486.134	531.557	608.735	92.678	2737.034	208.622
AMK58_07835	K11076 putrescine transport system ATP-binding protein | (GenBank) ABC transporter ATP-binding prote	Membrane Transport	52.235	42.070	49.065	87.147	45.483	113.634
AMK58_07840	K11073 putrescine transport system substrate-binding protein | (GenBank) spermidine/putrescine ABC t	Transport: Mineral and Ion	70.050	48.618	58.866	49.201	60.815	209.040
AMK58_07845	K01915 glutamine synthetase [EC:6.3.1.2] | (GenBank) glutamine synthetase	Metabolism	74.951	76.426	62.784	161.100	62.579	163.810
AMK58_07850	K09471 gamma-glutamylputrescine oxidase [EC:1.4.3.-] | (GenBank) FAD-dependent oxidoreductase	Metabolism: Amino Acid	1299.782	1175.100	1013.154	72.167	758.765	173.795
AMK58_07855	no KO assigned | (GenBank) hypothetical protein	Hypothetical	315.786	337.002	286.114	150.330	140.205	259.774
AMK58_07860	no KO assigned | (GenBank) ferric reductase	Oxidoreductase	14.175	14.564	17.169	141.142	91.541	177.488
AMK58_07865	no KO assigned | (GenBank) FAD-dependent oxidoreductase	Oxidoreductase	59.051	59.899	50.975	89.800	125.683	211.697
AMK58_07870	no KO assigned | (GenBank) hypothetical protein	Hypothetical	81.252	93.377	84.278	245.071	398.292	280.525
AMK58_07875	no KO assigned | (GenBank) hypothetical protein	Hypothetical	13.194	13.911	35.667	494.875	1092.526	518.648
AMK58_07880	K05794 tellurite resistance protein TerC | (GenBank) hypothetical protein	Defense	7.194	8.741	12.868	145.363	591.519	224.426
AMK58_07885	K03530 DNA-binding protein HU-beta | (GenBank) DNA-binding protein HU	DNA Repair and Replication	78.084	89.109	74.660	225.650	258.380	353.894
AMK58_07890	no KO assigned | (GenBank) MucR family transcriptional regulator	Transcription	50.669	49.280	59.100	252.158	1187.753	693.286
AMK58_07895	K03823 phosphinothricin acetyltransferase [EC:2.3.1.183] | (GenBank) GCN5 family acetyltransferase	Acetyltransferase	99.166	78.536	81.131	172.380	48.475	178.990
AMK58_07900	K03089 RNA polymerase sigma-32 factor | (GenBank) RNA polymerase subunit sigma-70	Transcription	309.821	253.296	232.564	40.592	60.486	180.312
AMK58_07905	no KO assigned | (GenBank) chemotaxis protein	Chemotaxis	33.797	35.980	31.450	47.078	38.999	147.443
AMK58_07910	no KO assigned | (GenBank) acetyltransferase	Acetyltransferase	2780.096	2973.795	2730.477	163.312	957.614	415.944
AMK58_07915	no KO assigned | (GenBank) hypothetical protein	Hypothetical	1514.077	1638.684	1472.349	189.389	292.413	792.562
AMK58_07920	no KO assigned | (GenBank) hypothetical protein	Hypothetical	339.743	458.438	362.799	53.500	51.845	96.134
AMK58_07925	no KO assigned | (GenBank) homoserine lactone transporter	Transport: Amino Acid	706.841	1149.862	1010.730	153.771	78.318	114.211
AMK58_07930	no KO assigned | (GenBank) hypothetical protein	Hypothetical	21.574	22.223	29.708	230.698	176.231	299.386
AMK58_07935	K03635 molybdopterin synthase catalytic subunit [EC:2.8.1.12] | (GenBank) molybdenum cofactor biosyn	Metabolism: Co-Factors and Vitamens	118.701	129.227	125.151	212.361	227.415	142.355
AMK58_07940	K03636 sulfur-carrier protein | (GenBank) molybdenum cofactor biosynthesis protein MoaD	Genetic Information Processing	90.326	86.114	94.562	664.190	559.494	784.106
AMK58_07945	K03750 molybdopterin molybdotransferase [EC:2.10.1.1] | (GenBank) molybdopterin biosynthesis protein	Biosynthesis: Co-Factors and Vitamins	44.993	39.802	42.320	77.989	88.233	92.931
AMK58_07950	K03753 molybdopterin-guanine dinucleotide biosynthesis protein B | (GenBank) molybdopterin-guanine d	Biosynthesis: Co-Factors and Vitamins	61.225	90.419	71.295	65.120	97.430	76.122
AMK58_07955	K08744 cardiolipin synthase (CMP-forming) [EC:2.7.8.41] | (GenBank) CDP-diacylglycerol--glycerol-3-p	Lipid metabolism	149.761	191.476	160.452	509.083	353.353	376.067
AMK58_07960	K03703 excinuclease ABC subunit C | (GenBank) excinuclease ABC subunit C	DNA Repair and Replication	50.898	47.225	45.766	45.539	94.919	74.429
AMK58_07965	no KO assigned | (GenBank) short-chain dehydrogenase	dehydrogenase	198.965	189.517	199.181	96.344	324.082	173.865
AMK58_07970	no KO assigned | (GenBank) hypothetical protein	Hypothetical	125.761	151.715	131.215	363.045	712.068	499.957
AMK58_07975	K06940 uncharacterized protein | (GenBank) hypothetical protein	Hypothetical	273.354	275.919	285.622	270.310	877.824	809.808
AMK58_07980	K01985 5S ribosomal RNA | (GenBank) 5S ribosomal RNA	Ribosome	136.731	137.188	124.051	1261.135	1055.895	1550.483
AMK58_07985	K01980 23S ribosomal RNA | (GenBank) 23S ribosomal RNA	Ribosome	3.035	3.416	3.262	24.449	14.287	30.912
AMK58_07990	K14218 tRNA Ala | (GenBank) tRNA-Ala	tRNA synthesis/modification	0.000	0.000	0.000	25.608	0.000	10.341
AMK58_07995	K14227 tRNA Ile | (GenBank) tRNA-Ile	tRNA synthesis/modification	0.000	0.000	0.000	0.000	0.000	0.000
AMK58_08000	K01977 16S ribosomal RNA | (GenBank) 16S ribosomal RNA	Ribosome	0.000	0.000	0.000	0.000	0.000	0.000
AMK58_08005	no KO assigned | (GenBank) peptide synthetase	Metabolism	0.000	0.000	0.000	0.000	0.000	0.000
AMK58_08010	K01676 fumarate hydratase, class I [EC:4.2.1.2] | (GenBank) fumarate hydratase	Metabolism: Carbohydrate	0.000	0.000	0.000	0.000	0.000	0.000
AMK58_08015	K03977 GTPase | (GenBank) ribosome-associated GTPase EngA	Ribosome	247.841	277.183	307.804	323.448	185.335	416.693
AMK58_08020	no KO assigned | (GenBank) pyrrolo-quinoline quinone	Energy	231.119	197.184	203.685	170.864	128.024	253.114
AMK58_08025	no KO assigned | (GenBank) hypothetical protein	Hypothetical	201.791	235.511	187.581	255.474	446.259	433.761
AMK58_08030	no KO assigned | (GenBank) alkyl hydroperoxide reductase	Defense	348.826	272.399	298.687	348.042	273.167	192.780
AMK58_08035	no KO assigned | (GenBank) two-component system response regulator	Signal Transduction	100.522	90.419	91.136	184.461	145.544	110.884

AMK58_08040	no KO assigned | (GenBank) transcriptional regulator	Transcription	105.717	104.556	132.098	112.669	201.731	252.690
AMK58_08045	K07684 two-component system, NarL family, nitrate/nitrite response regulator NarL | (GenBank) two-co	Nitrogen	3107.062	3461.251	3276.986	75.827	292.414	138.191
AMK58_08050	no KO assigned | (GenBank) hypothetical protein	Hypothetical	54.884	29.553	119.673	32.816	191.519	123.409
AMK58_08055	no KO assigned | (GenBank) hypothetical protein	Hypothetical	416.865	350.995	715.000	238.831	1529.756	1275.706
AMK58_08060	K03549 KUP system potassium uptake protein | (GenBank) trkD; potassium transport protein Kup	Transport: Ion	30.055	20.779	50.540	143.110	448.333	186.812
AMK58_08065	no KO assigned | (GenBank) amino acid ABC transporter substrate-binding protein	Membrane Transport	1.366	4.532	2.308	81.499	18.930	156.024
AMK58_08070	no KO assigned | (GenBank) pseudogene	Pseudogene	43.850	44.578	41.169	102.314	92.423	122.188
AMK58_08075	no KO assigned | (GenBank) hypothetical protein	Hypothetical	112.646	123.225	134.447	63.493	159.723	139.303
AMK58_08080	no KO assigned | (GenBank) chemotaxis protein	Chemotaxis	245.220	290.915	274.033	762.450	516.020	1138.764
AMK58_08085	no KO assigned | (GenBank) diguanylate cyclase	Cyclic nucleotide	57.516	71.756	70.433	104.127	66.998	108.949
AMK58_08090	K02225 cobalamin biosynthetic protein CobC | (GenBank) threonine-phosphate decarboxylase	Metabolism: Co-Factors and Vitamens	132.422	143.972	134.250	44.973	48.520	100.012
AMK58_08095	K02227 adenosylcobinamide-phosphate synthase [EC:6.3.1.10] | (GenBank) CobD/CbiB family cobalamin	Metabolism: Co-Factors and Vitamens	56.074	72.110	66.704	172.122	109.101	394.751
AMK58_08100	K00808 homospermidine synthase [EC:2.5.1.44] | (GenBank) homospermidine synthase	Transferase	14.761	13.764	17.199	124.925	26.758	80.701
AMK58_08105	K01581 ornithine decarboxylase [EC:4.1.1.17] | (GenBank) decarboxylase	Metabolism: Amino Acid	8.451	8.510	10.112	115.883	21.348	61.726
AMK58_08110	K07216 hemerythrin | (GenBank) hypothetical protein	hemerythrin	784.418	704.943	876.997	211.923	444.406	351.506
AMK58_08115	K08967 1,2-dihydroxy-3-keto-5-methylthiopentene dioxygenase [EC:1.13.11.53 1.13.11.54] | (GenBank) a	Metabolism: Amino Acid	35.421	44.889	43.068	442.848	1044.346	621.464
AMK58_08120	K08224 MFS transporter, YNFM family, putative membrane transport protein | (GenBank) hypothetical pr	Membrane Transport	30.991	41.079	30.130	96.511	52.533	143.225
AMK58_08125	no KO assigned | (GenBank) hypothetical protein	Hypothetical	59.081	55.515	62.768	127.073	49.262	90.150
AMK58_08130	no KO assigned | (GenBank) thioesterase	No COG	21.005	19.741	25.806	491.932	109.564	326.742
AMK58_08135	no KO assigned | (GenBank) MerR family transcriptional regulator	Transcription	64.080	46.015	55.363	366.053	86.920	275.104
AMK58_08140	K01897 long-chain acyl-CoA synthetase [EC:6.2.1.3] | (GenBank) dicarboxylate--CoA ligase PimA	Lipid metabolism	8.241	6.312	10.514	93.877	12.667	37.554
AMK58_08145	no KO assigned | (GenBank) sterol carrier	No COG	137.923	159.185	154.610	286.581	123.321	256.149
AMK58_08150	no KO assigned | (GenBank) hypothetical protein	Hypothetical	265.293	214.835	233.511	421.222	235.207	561.526
AMK58_08155	no KO assigned | (GenBank) hypothetical protein	Hypothetical	571.813	510.156	423.315	240.149	265.024	371.081
AMK58_08160	K02049 NitT/TauT family transport system ATP-binding protein | (GenBank) ABC transporter	Transport: NitT/TauT	179.924	145.055	147.071	77.539	108.983	141.642
AMK58_08165	K02050 NitT/TauT family transport system permease protein | (GenBank) nitrate ABC transporter permea	Transport: NitT/TauT	65.048	52.006	56.280	32.013	83.079	81.010
AMK58_08170	K02051 NitT/TauT family transport system substrate-binding protein | (GenBank) nitrate ABC transport	Transport: NitT/TauT	35.617	18.654	14.624	203.236	58.493	133.823
AMK58_08175	K00955 bifunctional enzyme CysN/CysC [EC:2.7.7.4 2.7.1.25] | (GenBank) NodQ bifunctional enzyme	Metabolism	21.826	3.472	5.035	65.047	29.178	55.626
AMK58_08180	K00957 sulfate adenylyltransferase subunit 2 [EC:2.7.7.4] | (GenBank) sulfate adenylyltransferase	Transferase	101.981	19.013	29.433	253.646	72.518	221.052
AMK58_08185	K00390 phosphoadenosine phosphosulfate reductase [EC:1.8.4.8 1.8.4.10] | (GenBank) phosphoadenosine	Energy	150.232	20.667	31.795	394.542	103.505	407.990
AMK58_08190	K02304 precorrin-2 dehydrogenase / sirohydrochlorin ferrochelatase [EC:1.3.1.76 4.99.1.4] | (GenBank	Metabolism: Co-Factors and Vitamens	137.999	29.816	33.422	245.023	123.898	303.611
AMK58_08195	K02303 uroporphyrin-III C-methyltransferase [EC:2.1.1.107] | (GenBank) uroporphyrin-III methyltransf	Metabolism: Co-Factors and Vitamens	14.235	6.138	6.000	157.075	26.559	188.341
AMK58_08200	no KO assigned | (GenBank) hypothetical protein	Hypothetical	10.785	10.393	9.262	247.473	35.588	246.477
AMK58_08205	K00381 sulfite reductase (NADPH) hemoprotein beta-component [EC:1.8.1.2] | (GenBank) sulfite reducta	Energy	9.821	5.996	6.475	89.399	22.642	72.254
AMK58_08210	no KO assigned | (GenBank) hypothetical protein	Hypothetical	48.868	17.430	22.423	139.714	83.350	92.882
AMK58_08215	no KO assigned | (GenBank) hypothetical protein	Hypothetical	1185.012	468.259	487.170	962.930	1460.865	1476.861
AMK58_08220	K03455 monovalent cation:H+ antiporter-2, CPA2 family | (GenBank) cation transporter	Transport: Ion	23.888	7.319	12.165	47.954	20.119	29.472
AMK58_08225	no KO assigned | (GenBank) hypothetical protein	Hypothetical	43.631	29.325	47.188	122.005	62.529	199.759
AMK58_08230	K14232 tRNA Pro | (GenBank) tRNA-Pro	tRNA synthesis/modification	1099.107	657.596	846.199	3176.316	843.071	5200.856
AMK58_08235	no KO assigned | (GenBank) MerR family transcriptional regulator	Transcription	73.289	62.129	62.621	36.152	17.255	215.815
AMK58_08240	K04764 integration host factor subunit alpha | (GenBank) integration host factor subunit alpha	DNA Repair and Replication	46.787	66.148	38.373	147.355	40.948	530.218
AMK58_08245	K00648 3-oxoacyl-[acyl-carrier-protein] synthase III [EC:2.3.1.180] | (GenBank) 3-oxoacyl-ACP syntha	Biosynthesis: Lipid	68.883	97.489	61.231	20.690	80.228	106.263
AMK58_08250	K03621 glycerol-3-phosphate acyltransferase PlsX [EC:2.3.1.15] | (GenBank) phosphate acyltransferase	Lipid metabolism	69.448	93.425	55.100	18.175	75.866	69.622
AMK58_08255	K02911 large subunit ribosomal protein L32 | (GenBank) 50S ribosomal protein L32	Ribosome	877.467	703.911	858.441	782.154	1088.746	1212.633
AMK58_08260	no KO assigned | (GenBank) hypothetical protein	Hypothetical	646.130	591.512	554.045	186.953	679.572	321.324
AMK58_08265	K17662 cytochrome b pre-mRNA-processing protein 3 | (GenBank) ubiquinol-cytochrome C chaperone	Chaperone	521.587	457.339	414.595	28.500	438.358	100.270
AMK58_08270	no KO assigned | (GenBank) cell envelope protein SmpA	Membrane	264.140	258.120	259.510	259.567	959.631	375.192
AMK58_08275	K15987 K(+)-stimulated pyrophosphate-energized sodium pump [EC:3.6.1.1] | (GenBank) hppA; pyropho	Energy	29.592	30.613	35.362	45.039	49.441	62.354
AMK58_08280	no KO assigned | (GenBank) hypothetical protein	Hypothetical	372.077	491.571	379.274	147.886	725.830	321.357
AMK58_08285	K00946 thiamine-monophosphate kinase [EC:2.7.4.16] | (GenBank) thiamine-monophosphate kinase	Signal Transduction	2576.638	2652.304	2464.787	232.625	196.134	467.350
AMK58_08290	K03625 N utilization substance protein B | (GenBank) antitermination protein NusB	Metabolism: Co-Factors and Vitamens	67.405	54.283	61.879	77.272	44.414	196.521
AMK58_08295	K00794 6,7-dimethyl-8-ribityllumazine synthase [EC:2.5.1.78] | (GenBank) 6,7-dimethyl-8-ribityllumaz	Metabolism: Co-Factors and Vitamens	156.746	183.561	182.952	422.381	288.869	371.818
AMK58_08300	K14652 3,4-dihydroxy 2-butanone 4-phosphate synthase / GTP cyclohydrolase II [EC:4.1.99.12 3.5.4.25]	Metabolism: Co-Factors and Vitamens	72.107	89.375	82.272	113.170	128.956	76.643
AMK58_08305	K00793 riboflavin synthase [EC:2.5.1.9] | (GenBank) riboflavin synthase subunit alpha	Metabolism: Co-Factors and Vitamens	284.361	306.319	244.334	58.199	132.159	137.282
AMK58_08310	K11752 diaminohydroxyphosphoribosylaminopyrimidine deaminase / 5-amino-6-(5-phosphoribosylamino)	Metabolism: Co-Factors and Vitamens	294.843	313.519	264.496	96.384	224.268	182.100
AMK58_08315	K07738 transcriptional repressor NrdR | (GenBank) NrdR family transcriptional regulator	Transcription	295.006	404.457	369.866	142.305	309.063	236.370
AMK58_08320	K00600 glycine hydroxymethyltransferase [EC:2.1.2.1] | (GenBank) glyA; serine hydroxymethyltransfera	Metabolism: Carbon	19.579	25.933	25.026	112.701	45.364	60.031
AMK58_08325	K01808 ribose 5-phosphate isomerase B [EC:5.3.1.6] | (GenBank) ribose 5-phosphate isomerase	Metabolism	577.908	455.446	622.761	85.598	362.285	192.314
AMK58_08330	no KO assigned | (GenBank) transcriptional regulator	Transcription	1510.708	1213.738	1497.843	448.933	1233.728	753.775
AMK58_08335	no KO assigned | (GenBank) pseudogene	Pseudogene	618.827	733.402	694.977	187.715	794.507	612.805
AMK58_08340	no KO assigned | (GenBank) hypothetical protein	Hypothetical	2272.021	1943.832	2047.506	335.051	677.106	562.272
AMK58_08345	no KO assigned | (GenBank) hypothetical protein	Hypothetical	233.889	320.081	230.238	57.856	149.358	150.568
AMK58_08350	no KO assigned | (GenBank) hypothetical protein	Hypothetical	184.904	196.657	179.890	84.352	104.885	242.057
AMK58_08355	no KO assigned | (GenBank) hypothetical protein	Hypothetical	16.720	15.500	26.039	132.066	2604.889	431.971
AMK58_08360	no KO assigned | (GenBank) hypothetical protein	Hypothetical	100.765	101.114	110.415	141.259	717.636	294.316
AMK58_08365	K02348 ElaA protein | (GenBank) hypothetical protein	No COG	145.559	178.428	168.074	194.623	180.550	211.067
AMK58_08370	no KO assigned | (GenBank) hypothetical protein	Hypothetical	35.665	42.990	34.208	64.958	34.287	133.592

AMK58_08375	K10747 DNA ligase 1 [EC:6.5.1.1 6.5.1.6 6.5.1.7] | (GenBank) ATP-dependent DNA ligase	DNA Repair and Replication	16.113	20.170	16.998	85.204	18.715	38.988
AMK58_08380	K07577 putative mRNA 3-end processing factor | (GenBank) DNA ligase-associated DEXH box helicase	DNA Repair and Replication	58.596	62.564	73.837	60.839	60.839	181.879
AMK58_08385	K16329 pseudouridylate synthase [EC:4.2.1.70] | (GenBank) pseudouridine-5-phosphate glycosidase	Metabolism: Nucleotide	27.344	34.558	34.373	466.130	139.492	388.860
AMK58_08390	no KO assigned | (GenBank) hypothetical protein	Hypothetical	49.424	69.889	52.276	417.217	276.789	296.403
AMK58_08395	K00772 5'-methylthioadenosine phosphorylase [EC:2.4.2.28] | (GenBank) 5'-methylthioadenosine phospho	Metabolism: Amino Acid	22.163	25.338	23.024	170.982	52.900	191.429
AMK58_08400	no KO assigned | (GenBank) hypothetical protein	Hypothetical	228.639	314.592	258.235	71.464	62.303	127.689
AMK58_08405	K09800 translocation and assembly module TamB | (GenBank) hypothetical protein	Hypothetical	28.759	28.693	29.427	24.115	44.796	36.069
AMK58_08410	K07278 translocation and assembly module TamA | (GenBank) hypothetical protein	Hypothetical	29.025	32.617	37.322	117.937	168.718	65.619
AMK58_08415	K06207 GTP-binding protein | (GenBank) GTP-binding protein TypA	Enzyme	42.280	52.262	54.188	374.261	194.195	171.200
AMK58_08420	no KO assigned | (GenBank) peroxiredoxin	No COG	132.825	141.924	159.321	715.123	261.850	486.028
AMK58_08425	no KO assigned | (GenBank) hypothetical protein	Hypothetical	489.592	422.946	538.480	1173.410	1878.532	2070.547
AMK58_08430	no KO assigned | (GenBank) diadenosine tetraphosphate hydrolase	Hydrolase	182.502	246.437	226.570	64.795	106.073	262.788
AMK58_08435	no KO assigned | (GenBank) cellulose synthase	Metabolism: Carbohydrates	14.798	15.243	18.216	17.524	210.032	97.042
AMK58_08440	no KO assigned | (GenBank) hypothetical protein	Hypothetical	51.134	32.773	32.226	44.352	176.613	201.649
AMK58_08445	no KO assigned | (GenBank) hypothetical protein	Hypothetical	1859.112	2053.002	2087.434	587.238	3479.581	1695.435
AMK58_08450	no KO assigned | (GenBank) Crp/Fnr family transcriptional regulator	Transcription	37.964	44.954	44.226	95.067	67.470	273.697
AMK58_08455	no KO assigned | (GenBank) hypothetical protein	Hypothetical	37.141	27.956	39.485	146.124	202.825	277.420
AMK58_08460	K16168 methyltransferase | (GenBank) hypothetical protein	methyltransferase	105.604	115.201	94.440	287.730	162.052	381.717
AMK58_08465	K16167 alkylresorcinol/alkylpyrone synthase | (GenBank) chalcone synthase	Biosynthesis	10.263	11.711	15.130	63.639	43.420	103.181
AMK58_08470	no KO assigned | (GenBank) hypothetical protein	Hypothetical	14.509	15.942	25.053	129.570	65.985	82.733
AMK58_08475	no KO assigned | (GenBank) hypothetical protein	Hypothetical	34.816	35.658	44.318	310.856	213.884	202.929
AMK58_08480	no KO assigned | (GenBank) hypothetical protein	Hypothetical	532.505	458.795	499.915	389.697	1217.278	1044.732
AMK58_08485	no KO assigned | (GenBank) transposase	Genetic Information Processing	58.604	86.495	59.398	64.157	91.286	224.815
AMK58_08490	K03655 ATP-dependent DNA helicase RecG [EC:3.6.4.12] | (GenBank) ATP-dependent DNA helicase Re	DNA Repair and Replication	1.338	0.000	0.885	1.636	0.588	10.893
AMK58_08495	K02232 adenosylcobyric acid synthase [EC:6.3.5.10] | (GenBank) cobalamin biosynthesis protein CobQ	Metabolism: Co-Factors and Vitamens	46.599	62.144	60.685	247.570	144.418	343.599
AMK58_08500	K19221 cob(I)alamin adenosyltransferase [EC:2.5.1.17] | (GenBank) cob(I)yrinic acid a,c-diamide aden	Metabolism: Co-Factors and Vitamens	27.911	30.421	37.656	457.000	40.701	223.490
AMK58_08505	K02231 adenosylcobinamide kinase / adenosylcobinamide-phosphate guanylyltransferase [EC:2.7.1.156 2.	Metabolism: Co-Factors and Vitamens	49.814	45.468	50.433	143.032	39.921	206.149
AMK58_08510	no KO assigned | (GenBank) esterase	esterase	12.776	12.841	13.624	205.531	37.983	82.154
AMK58_08515	no KO assigned | (GenBank) hypothetical protein	Hypothetical	54.396	41.934	58.394	1091.817	136.586	492.197
AMK58_08520	no KO assigned | (GenBank) hypothetical protein	Hypothetical	94.272	156.451	147.926	183.233	79.994	103.985
AMK58_08525	no KO assigned | (GenBank) hypothetical protein	Hypothetical	118.437	143.410	149.479	187.980	102.885	216.468
AMK58_08530	no KO assigned | (GenBank) disulfide bond formation protein DsbA	Oxidoreductase	26.844	34.287	27.460	130.691	45.781	61.113
AMK58_08535	K15034 ribosome-associated protein | (GenBank) peptide chain release factor I	Translation	208.710	270.397	299.857	361.829	347.038	276.932
AMK58_08540	no KO assigned | (GenBank) hypothetical protein	Hypothetical	84.352	145.540	138.281	137.834	830.455	236.339
AMK58_08545	no KO assigned | (GenBank) hypothetical protein	Hypothetical	3.040	4.250	5.781	27.724	180.803	51.877
AMK58_08550	K09707 uncharacterized protein | (GenBank) amino acid-binding protein	No COG	8.749	6.745	21.467	765.185	308.351	751.867
AMK58_08555	K03723 transcription-repair coupling factor (superfamily II helicase) [EC:3.6.4.-] | (GenBank) trans	Transcription	0.768	0.997	1.143	29.960	4.653	9.843
AMK58_08560	K09159 antitoxin CptB | (GenBank) hypothetical protein	Defense	407.978	470.300	509.107	1889.492	1076.169	1901.174
AMK58_08565	no KO assigned | (GenBank) enoyl-CoA hydratase	Hydratase	33.994	39.877	35.864	51.151	91.427	87.225
AMK58_08570	no KO assigned | (GenBank) hypothetical protein	Hypothetical	8.315	15.751	8.677	295.575	15.501	118.287
AMK58_08575	no KO assigned | (GenBank) hypothetical protein	Hypothetical	323.146	471.100	380.274	110.746	51.860	72.081
AMK58_08580	no KO assigned | (GenBank) ferredoxin	Ferredoxin	64.719	67.339	139.318	835.769	852.115	1040.770
AMK58_08585	K00763 nicotinate phosphoribosyltransferase [EC:6.3.4.21] | (GenBank) nicotinate phosphoribosyltrans	Metabolism: Co-Factors and Vitamens	3.433	4.350	3.066	27.449	39.635	67.034
AMK58_08590	no KO assigned | (GenBank) hypothetical protein	Hypothetical	270.432	227.829	263.172	505.288	288.260	689.530
AMK58_08595	K00383 glutathione reductase (NADPH) [EC:1.8.1.7] | (GenBank) glutathione reductase	Energy	11.902	9.089	11.571	73.121	13.177	90.695
AMK58_08600	K07217 Mn-containing catalase | (GenBank) Mn-containing catalase	Enzyme	325.701	293.536	304.674	245.539	216.657	233.426
AMK58_08605	K08218 MFS transporter, PAT family, beta-lactamase induction signal transducer AmpG | (GenBank) MFS	Transport: MFS	44.863	34.446	38.973	96.660	1079.430	159.550
AMK58_08610	K01626 3-deoxy-7-phosphoheptulonate synthase [EC:2.5.1.54] | (GenBank) phospho-2-dehydro-3-deoxyh	Biosynthesis: Amino Acid	23.062	25.051	25.831	153.527	91.613	183.552
AMK58_08615	no KO assigned | (GenBank) hypothetical protein	Hypothetical	902.047	780.841	871.397	631.558	676.861	680.310
AMK58_08620	K00135 succinate-semialdehyde dehydrogenase / glutarate-semialdehyde dehydrogenase [EC:1.2.1.16 1.2.	Metabolism	66.291	76.015	79.135	54.171	56.204	82.962
AMK58_08625	K01918 pantoate--beta-alanine ligase [EC:6.3.2.1] | (GenBank) pantoate--beta-alanine ligase	Metabolism: Co-Factors and Vitamens	416.220	417.289	441.871	249.377	279.976	249.236
AMK58_08630	no KO assigned | (GenBank) sugar tyrosine-protein kinase	Signal Transduction	234.078	249.886	219.075	867.940	412.404	877.745
AMK58_08635	K14237 tRNA Val | (GenBank) tRNA-Val	tRNA synthesis/modification	1067.072	1128.435	996.163	339.509	328.438	799.360
AMK58_08640	K14237 tRNA Val | (GenBank) tRNA-Val	tRNA synthesis/modification	1209.134	1475.645	1378.357	188.136	919.990	977.495
AMK58_08645	no KO assigned | (GenBank) hypothetical protein	Hypothetical	121.478	139.045	136.992	18.021	61.393	94.556
AMK58_08650	no KO assigned | (GenBank) hypothetical protein	Hypothetical	176.188	197.043	234.777	548.758	954.116	706.615
AMK58_08655	K01091 phosphoglycolate phosphatase [EC:3.1.3.18] | (GenBank) phosphoglycolate phosphatase	Metabolism: Carbohydrate	55.293	43.550	60.011	79.783	129.898	113.424
AMK58_08660	K04042 bifunctional UDP-N-acetylglucosamine pyrophosphorylase / Glucosamine-1-phosphate N-acetyltra	Metabolism: Carbohydrate	27.054	34.674	29.968	117.573	16.259	59.529
AMK58_08665	K00820 glucosamine--fructose-6-phosphate aminotransferase (isomerizing) [EC:2.6.1.16] | (GenBank) gl	Metabolism: Amino Acid	43.473	34.841	37.743	174.614	29.424	145.207
AMK58_08670	no KO assigned | (GenBank) hypothetical protein	Hypothetical	497.683	394.893	517.644	385.329	176.134	460.726
AMK58_08675	no KO assigned | (GenBank) hypothetical protein	Hypothetical	38.414	27.098	25.523	106.512	9.260	135.187
AMK58_08680	no KO assigned | (GenBank) hypothetical protein	Hypothetical	261.917	376.440	358.670	903.607	74.527	613.283
AMK58_08685	K03719 Lrp/AsnC family transcriptional regulator, leucine-responsive regulatory protein | (GenBank)	Transcription	1.062	4.759	8.583	219.449	12.236	95.774
AMK58_08690	no KO assigned | (GenBank) lysine transporter LysE	Transport: Amino Acid	10.090	5.796	12.361	121.639	27.917	139.078
AMK58_08695	K07001 NTE family protein | (GenBank) patatin	Metabolism: Lipid	8.723	20.846	14.929	126.352	33.206	67.836
AMK58_08700	K06968 23S rRNA (cytidine2498-2'-O)-methyltransferase [EC:2.1.1.186] | (GenBank) methyltransferase	Ribosome	29.260	26.372	34.895	292.104	124.185	139.869
AMK58_08705	no KO assigned | (GenBank) hypothetical protein	Hypothetical	43.453	39.200	45.318	183.578	102.139	163.354

AMK58_08710	K02192 bacterioferritin-associated ferredoxin | (GenBank) (2Fe-2S)-binding protein	Ferrodoxin	114.273	132.751	142.282	676.078	610.126	936.422
AMK58_08715	no KO assigned | (GenBank) hypothetical protein	Hypothetical	1725.976	1750.926	1414.779	177.849	211.559	446.630
AMK58_08720	K07716 two-component system, cell cycle sensor histidine kinase PleC [EC:2.7.13.3] | (GenBank) histi	Signal Transduction	92.179	174.278	140.897	53.510	165.950	54.416
AMK58_08725	K11712 two-component system, LuxR family, response regulator DctR | (GenBank) two-component syster	Signal Transduction	325.677	404.263	461.239	481.985	1252.825	561.721
AMK58_08730	K11711 two-component system, LuxR family, sensor histidine kinase DctS [EC:2.7.13.3] | (GenBank) PA	Signal Transduction	24.382	25.999	21.936	82.251	39.401	39.137
AMK58_08735	K11688 C4-dicarboxylate-binding protein DctP | (GenBank) C4-dicarboxylate ABC transporter	Transport	137.721	146.674	142.788	461.532	167.727	402.883
AMK58_08740	K11689 C4-dicarboxylate transporter, DctQ subunit | (GenBank) C4-dicarboxylate ABC transporter perme	Transport	2365.413	1513.515	1877.950	150.952	388.052	360.624
AMK58_08745	K11690 C4-dicarboxylate transporter, DctM subunit | (GenBank) C4-dicarboxylate ABC transporter perme	Transport	257.435	144.782	185.927	23.597	77.319	91.613
AMK58_08750	K06193 protein PhnA | (GenBank) PhnA protein	No COG	1136.814	630.150	899.991	414.324	295.059	615.125
AMK58_08755	no KO assigned | (GenBank) hypothetical protein	Hypothetical	29.076	23.976	20.374	79.419	9.277	82.289
AMK58_08760	K02030 polar amino acid transport system substrate-binding protein | (GenBank) hypothetical protein	Transport: Amino Acid	51.889	68.891	75.223	420.053	75.427	326.117
AMK58_08765	no KO assigned | (GenBank) dehydrogenase	Dehydrogenase	24.368	29.259	28.387	62.361	20.427	96.458
AMK58_08770	K00459 nitronate monooxygenase [EC:1.13.12.16] | (GenBank) nitronate monooxygenase	Nitrogen	150.155	173.077	184.295	444.049	143.177	367.568
AMK58_08775	K13590 diguanylate cyclase [EC:2.7.7.65] | (GenBank) diguanylate cyclase	cyclic nucleotide	13.997	15.331	20.185	254.216	74.602	80.363
AMK58_08780	no KO assigned | (GenBank) hypothetical protein	Hypothetical	346.231	273.550	378.793	314.887	1316.363	440.325
AMK58_08785	K07107 acyl-CoA thioester hydrolase [EC:3.1.2.-] | (GenBank) thioesterase-like protein	Hydrolase	554.009	495.389	525.128	100.588	1659.787	225.673
AMK58_08790	K00548 5-methyltetrahydrofolate--homocysteine methyltransferase [EC:2.1.1.13] | (GenBank) methionine	Metabolism: Amino Acid	18.515	21.750	21.263	31.490	24.787	38.677
AMK58_08795	no KO assigned | (GenBank) hypothetical protein	Hypothetical	866.425	911.062	929.452	1066.184	826.608	1498.113
AMK58_08800	K00297 methylenetetrahydrofolate reductase (NADPH) [EC:1.5.1.20] | (GenBank) 5,10-methylenetetrahyd	Energy	100.484	121.379	90.973	114.560	85.945	223.903
AMK58_08805	no KO assigned | (GenBank) ArsR family transcriptional regulator	Transcription	57.092	47.783	61.069	146.363	76.483	178.103
AMK58_08810	K04754 phospholipid-binding lipoprotein MlaA | (GenBank) hypothetical protein	Membrane	26.646	24.205	26.224	273.023	180.056	200.698
AMK58_08815	K07323 phospholipid transport system substrate-binding protein | (GenBank) toluene tolerance protein	Transport: Membrane	415.337	452.975	393.789	234.356	101.740	258.693
AMK58_08820	no KO assigned | (GenBank) hypothetical protein	Hypothetical	178.589	169.369	173.921	68.070	906.737	124.769
AMK58_08825	no KO assigned | (GenBank) ABC transporter ATP-binding protein	Membrane Transport	94.939	97.483	90.063	170.697	138.365	151.701
AMK58_08830	K09768 uncharacterized protein | (GenBank) hypothetical protein	Hypothetical	1240.360	1028.677	1026.988	405.305	1752.569	814.010
AMK58_08835	K02342 DNA polymerase III subunit epsilon [EC:2.7.7.7] | (GenBank) hypothetical protein	DNA Repair and Replication	6.174	9.909	8.936	75.292	44.225	88.944
AMK58_08840	no KO assigned | (GenBank) hypothetical protein	Hypothetical	37.172	41.244	56.213	398.826	113.610	148.216
AMK58_08845	no KO assigned | (GenBank) hypothetical protein	Hypothetical	43.067	59.875	57.946	581.620	181.762	463.144
AMK58_08850	no KO assigned | (GenBank) hypothetical protein	Hypothetical	86.570	75.465	113.654	278.564	89.690	310.241
AMK58_08855	K03594 bacterioferritin [EC:1.16.3.1] | (GenBank) bacterioferritin	Metabolism: Co-Factors and Vitamens	116.591	168.188	174.573	121.806	98.850	209.755
AMK58_08860	K00705 4-alpha-glucanotransferase [EC:2.4.1.25] | (GenBank) 4-alpha-glucanotransferase	Metabolism: Carbohydrates	246.517	328.318	236.125	19.107	74.199	55.827
AMK58_08865	no KO assigned | (GenBank) regulator	Signal Transduction	93.019	92.738	92.489	372.273	189.083	264.308
AMK58_08870	K01214 isoamylase [EC:3.2.1.68] | (GenBank) glycogen debranching protein	Metabolism: Carbohydrate	17.449	24.549	21.140	146.050	34.862	80.118
AMK58_08875	K00700 1,4-alpha-glucan branching enzyme [EC:2.4.1.18] | (GenBank) glycogen branching protein	Metabolism: Carbohydrate	96.753	88.144	101.345	148.939	149.973	219.583
AMK58_08880	K05343 maltose alpha-D-glucosyltransferase / alpha-amylase [EC:5.4.99.16 3.2.1.1] | (GenBank) alpha-	Metabolism: Carbohydrate	79.551	77.471	84.400	110.710	204.713	163.000
AMK58_08885	K07216 hemerythrin | (GenBank) hypothetical protein	hemerythrin	555.274	545.427	596.237	984.300	1788.609	1345.946
AMK58_08890	no KO assigned | (GenBank) GCN5 family acetyltransferase	Acetyltransferase	39.225	47.411	36.002	39.787	46.129	237.364
AMK58_08895	K01082 3'(2'), 5'-bisphosphate nucleotidase [EC:3.1.3.7] | (GenBank) 3'(2'),5'-bisphosphate nucleoti	Energy	6.257	14.953	8.775	191.103	12.836	153.319
AMK58_08900	no KO assigned | (GenBank) rhamnosyltransferase	Transferase	246.246	193.983	244.087	211.167	89.023	182.895
AMK58_08905	no KO assigned | (GenBank) acyl-CoA dehydrogenase	dehydrogenase	98.723	103.866	108.330	297.757	93.869	202.373
AMK58_08910	no KO assigned | (GenBank) aminoglycoside phosphotransferase	Transferase	29.940	37.347	39.207	54.503	19.477	40.169
AMK58_08915	no KO assigned | (GenBank) acyl-CoA dehydrogenase	dehydrogenase	111.466	145.638	132.911	167.335	31.721	73.815
AMK58_08920	no KO assigned | (GenBank) 2-deoxy-D-gluconate 3-dehydrogenase	Dehydrogenase	529.742	524.006	578.767	238.596	70.547	259.605
AMK58_08925	K00666 fatty-acyl-CoA synthase [EC:6.2.1.-] | (GenBank) acyl-CoA synthetase	Lipid biosynthesis	50.874	72.999	63.157	75.191	12.236	37.492
AMK58_08930	no KO assigned | (GenBank) hypothetical protein	Hypothetical	695.803	849.418	727.972	481.174	174.493	342.244
AMK58_08935	no KO assigned | (GenBank) hypothetical protein	Hypothetical	5.825	2.784	11.565	29.778	19.382	65.858
AMK58_08940	K01768 adenylate cyclase [EC:4.6.1.1] | (GenBank) hypothetical protein	Metabolism: Nucleotide	16.437	21.158	25.031	308.440	34.849	195.976
AMK58_08945	no KO assigned | (GenBank) hypothetical protein	Hypothetical	2.585	4.751	6.711	339.019	32.180	148.126
AMK58_08950	no KO assigned | (GenBank) hypothetical protein	Hypothetical	27.444	38.579	40.420	912.566	154.844	390.199
AMK58_08955	no KO assigned | (GenBank) hypothetical protein	Hypothetical	22.956	35.394	37.317	123.499	345.019	190.549
AMK58_08960	no KO assigned | (GenBank) hypothetical protein	Hypothetical	2.342	2.469	2.881	73.251	34.681	74.846
AMK58_08965	no KO assigned | (GenBank) peptidoglycan-binding protein	Cell Wall	1.160	1.848	3.529	117.238	17.381	59.433
AMK58_08970	no KO assigned | (GenBank) pseudogene	Pseudogene	17.255	8.736	37.817	399.197	162.063	697.211
AMK58_08975	no KO assigned | (GenBank) ATPase	enzyme	6.517	0.000	15.969	190.728	35.221	229.421
AMK58_08980	no KO assigned | (GenBank) mechanosensitive ion channel protein MscS	Transport: Ion	7.765	6.104	12.921	71.307	10.336	98.532
AMK58_08985	no KO assigned | (GenBank) hypothetical protein	Hypothetical	39.530	36.477	55.395	175.632	61.300	99.891
AMK58_08990	no KO assigned | (GenBank) transcriptional regulator	Transcription	129.501	164.279	179.847	484.775	145.565	319.331
AMK58_08995	no KO assigned | (GenBank) hypothetical protein	Hypothetical	38.228	35.943	59.109	159.500	16.483	69.733
AMK58_09000	no KO assigned | (GenBank) hypothetical protein	Hypothetical	4.684	8.611	16.103	605.784	255.580	564.356
AMK58_09005	no KO assigned | (GenBank) hypothetical protein	Hypothetical	502.442	491.085	477.374	85.309	160.232	331.316
AMK58_09010	no KO assigned | (GenBank) hypothetical protein	Hypothetical	72.068	98.170	110.322	92.678	36.130	81.529
AMK58_09015	no KO assigned | (GenBank) hypothetical protein	Hypothetical	77.957	72.129	121.022	420.020	278.390	214.944
AMK58_09020	K03724 ATP-dependent helicase Lhr and Lhr-like helicase [EC:3.6.4.-] | (GenBank) DNA ligase-associat	DNA Repair and Replication	31.808	45.830	39.906	11.847	87.598	76.523
AMK58_09025	K06953 uncharacterized protein | (GenBank) phosphoesterase	phosphoesterase	6.903	4.230	5.722	43.392	20.693	205.499
AMK58_09030	no KO assigned | (GenBank) Nex18 symbiotically induced protein	No COG	63.385	76.921	87.340	859.021	570.812	445.176
AMK58_09035	K01669 deoxyribodipyrimidine photo-lyase [EC:4.1.99.3] | (GenBank) deoxyribodipyrimidine photolyase	DNA Repair and Replication	3.571	5.954	7.738	117.690	57.032	41.504
AMK58_09040	K07093 uncharacterized protein | (GenBank) dTDP-glucose 4,6-dehydratase	Metabolism: Carbohydrate	226.494	233.295	176.414	643.959	3110.271	1992.869

AMK58_09045	K09823 Fur family transcriptional regulator, zinc uptake regulator | (GenBank) Fur family transcript	Transcription	75.702	87.031	104.220	662.499	429.217	400.584
AMK58_09050	no KO assigned | (GenBank) ATP-binding protein	No COG	16.389	22.643	23.082	284.031	184.106	354.381
AMK58_09055	no KO assigned | (GenBank) hypothetical protein	Hypothetical	8.102	8.677	8.285	37.680	14.262	32.178
AMK58_09060	K16649 rhamnopyranosyl-N-acetylglucosaminyl-diphospho-decaprenol beta-1,3/1,4-galactofuranosyltrans	Biosynthesis: polysaccharides	173.576	147.232	196.732	199.822	152.954	241.209
AMK58_09065	no KO assigned | (GenBank) transporter	Transport	26.223	31.450	35.592	217.031	158.775	253.194
AMK58_09070	no KO assigned | (GenBank) MBL fold metallo-hydrolase	Hydrolase	14.382	11.398	23.163	182.631	187.891	199.364
AMK58_09075	no KO assigned | (GenBank) hypothetical protein	Hypothetical	107.105	123.651	119.055	996.681	644.407	898.637
AMK58_09080	no KO assigned | (GenBank) chemotaxis protein	Chemotaxis	78.041	110.484	79.511	103.606	154.396	262.286
AMK58_09085	K03406 methyl-accepting chemotaxis protein | (GenBank) chemotaxis protein	Chemotaxis	8.176	11.952	15.465	8.971	21.705	45.005
AMK58_09090	K13283 ferrous-iron efflux pump FieF | (GenBank) fieF; ferrous iron transporter	Transport: Metal	61.702	93.511	106.141	122.333	144.090	227.058
AMK58_09095	K00275 pyridoxamine 5'-phosphate oxidase [EC:1.4.3.5] | (GenBank) pyridoxamine 5'-phosphate oxidase	Metabolism: Co-Factors and Vitamins	464.120	641.801	486.246	300.786	317.996	451.280
AMK58_09100	no KO assigned | (GenBank) hypothetical protein	Hypothetical	181.081	187.184	167.377	226.918	188.023	342.964
AMK58_09105	no KO assigned | (GenBank) molecular chaperone DnaJ	Chaperone	67.096	74.596	55.881	96.974	89.311	137.764
AMK58_09110	K03798 cell division protease FtsH [EC:3.4.24.-] | (GenBank) cell division protein FtsH	Cell Cycle/Shape/Homeostatsis	67.061	56.948	51.164	30.246	79.103	52.781
AMK58_09115	no KO assigned | (GenBank) ABC transporter	Membrane Transport	207.301	190.404	215.653	290.857	315.040	199.130
AMK58_09120	no KO assigned | (GenBank) hypothetical protein	Hypothetical	18.219	16.993	20.027	219.646	77.854	104.072
AMK58_09125	no KO assigned | (GenBank) two-component system response regulator	Signal Transduction	211.550	205.544	204.761	228.655	159.333	225.460
AMK58_09130	no KO assigned | (GenBank) histidine kinase	Signal Transduction	230.619	272.808	238.899	293.634	152.554	257.365
AMK58_09135	no KO assigned | (GenBank) Fe-S oxidoreductase	Oxidoreductase	426.644	386.152	387.769	189.014	433.179	424.036
AMK58_09140	no KO assigned | (GenBank) hypothetical protein	Hypothetical	162.968	186.081	170.176	262.671	241.368	374.842
AMK58_09145	no KO assigned | (GenBank) histidine kinase	Signal Transduction	54.129	54.985	48.355	11.415	31.265	35.274
AMK58_09150	no KO assigned | (GenBank) hypothetical protein	Hypothetical	188.909	177.774	151.649	40.776	74.582	144.724
AMK58_09155	no KO assigned | (GenBank) hypothetical protein	Hypothetical	92.338	114.878	105.877	462.216	178.132	757.477
AMK58_09160	no KO assigned | (GenBank) hypothetical protein	Hypothetical	192.426	262.433	256.739	583.332	262.412	313.445
AMK58_09165	no KO assigned | (GenBank) hypothetical protein	Hypothetical	147.539	193.685	208.221	787.000	231.284	460.122
AMK58_09170	K11741 quaternary ammonium compound-resistance protein SugE | (GenBank) molecular chaperone	Chaperone	92.139	156.955	131.047	354.463	245.225	376.704
AMK58_09175	no KO assigned | (GenBank) short-chain dehydrogenase	dehydrogenase	1.525	2.103	5.354	64.277	25.979	32.093
AMK58_09180	K07397 putative redox protein | (GenBank) peroxiredoxin	No COG	129.435	129.171	138.417	82.251	1333.065	109.329
AMK58_09185	no KO assigned | (GenBank) hypothetical protein	Hypothetical	41.139	57.557	45.057	409.825	446.327	312.424
AMK58_09190	K00790 UDP-N-acetylglucosamine 1-carboxyvinyltransferase [EC:2.5.1.7] | (GenBank) UDP-N-acetylglu	Cell Wall	43.448	53.147	35.613	39.386	113.148	60.520
AMK58_09195	no KO assigned | (GenBank) GCN5 family acetyltransferase	Acetyltransferase	122.722	124.660	122.167	146.004	441.609	271.382
AMK58_09200	no KO assigned | (GenBank) hypothetical protein	Hypothetical	112.352	102.670	100.389	198.460	227.261	260.755
AMK58_09205	K01953 asparagine synthase (glutamine-hydrolysing) [EC:6.3.5.4] | (GenBank) asparagine synthase	Metabolism: Amino Acid	8.490	6.616	7.429	18.048	20.746	26.128
AMK58_09210	no KO assigned | (GenBank) hypothetical protein	Hypothetical	90.360	93.887	115.798	519.569	200.326	469.943
AMK58_09215	K08679 UDP-glucuronate 4-epimerase [EC:5.1.3.6] | (GenBank) protein CapI	Metabolism: Carbohydrate	145.807	147.759	131.966	353.216	191.416	267.553
AMK58_09220	no KO assigned | (GenBank) glycosyl transferase family 1	Glycosyl Transferase	134.809	126.871	112.708	22.361	113.411	55.351
AMK58_09225	no KO assigned | (GenBank) hypothetical protein	Hypothetical	99.566	90.961	96.392	143.572	71.035	242.467
AMK58_09230	no KO assigned | (GenBank) NAD-dependent dehydratase	Energy	22.844	28.813	30.167	221.518	22.682	70.610
AMK58_09235	K13013 O-antigen biosynthesis protein WbqV | (GenBank) nucleotide sugar dehydratase	Biosynthesis: polysacchharide	29.083	25.400	25.309	95.661	11.151	70.447
AMK58_09240	K14234 tRNA Thr | (GenBank) tRNA-Thr	tRNA synthesis/modification	251.836	270.053	303.914	2543.075	270.372	1158.623
AMK58_09245	K03640 peptidoglycan-associated lipoprotein | (GenBank) cell envelope biogenesis protein OmpA	Cell Wall	462.216	401.864	436.583	776.588	301.859	775.061
AMK58_09250	K03641 TolB protein | (GenBank) tolB; translocation protein TolB	Transport	23.177	37.424	55.730	38.119	49.488	45.887
AMK58_09255	no KO assigned | (GenBank) hypothetical protein	Hypothetical	2807.304	2491.766	2148.390	42.857	526.627	130.303
AMK58_09260	K03560 biopolymer transport protein TolR | (GenBank) protein TolR	Transport: Biopolymer	440.682	329.479	382.300	352.242	171.596	469.301
AMK58_09265	K03562 biopolymer transport protein TolQ | (GenBank) protein TolQ	Transport: Biopolymer	69.329	49.024	63.053	216.248	57.411	113.823
AMK58_09270	K07107 acyl-CoA thioester hydrolase [EC:3.1.2.-] | (GenBank) acyl-CoA thioester hydrolase	Hydrolases	59.024	64.801	58.998	108.800	56.422	119.988
AMK58_09275	K03551 holliday junction DNA helicase RuvB [EC:3.6.4.12] | (GenBank) ATP-dependent DNA helicase R	DNA Repair and Replication	59.540	73.528	57.032	147.185	52.842	146.403
AMK58_09280	K03550 holliday junction DNA helicase RuvA [EC:3.6.4.12] | (GenBank) ATP-dependent DNA helicase R	DNA Repair and Replication	49.048	48.454	46.084	37.248	88.992	149.335
AMK58_09285	K01159 crossover junction endodeoxyribonuclease RuvC [EC:3.1.22.4] | (GenBank) crossover junction en	DNA Repair and Replication	33.874	46.754	42.731	188.543	101.787	142.955
AMK58_09290	no KO assigned | (GenBank) transcriptional regulator	Transcription	19.295	21.134	19.416	113.724	116.935	105.859
AMK58_09295	K09769 uncharacterized protein | (GenBank) metallophosphoesterase	No COG	108.408	102.959	119.040	193.360	399.008	273.572
AMK58_09300	K01934 5-formyltetrahydrofolate cyclo-ligase [EC:6.3.3.2] | (GenBank) 5-formyltetrahydrofolate cyclo	Metabolism: Co-Factors and Vitamens	205.767	195.983	183.359	194.144	453.421	215.691
AMK58_09305	K09888 cell division protein ZapA | (GenBank) hypothetical protein	Cell Cycle/Shape/Homeostatsis	268.286	254.897	275.920	522.599	968.206	469.751
AMK58_09310	no KO assigned | (GenBank) hypothetical protein	Hypothetical	156.590	160.746	162.701	571.615	970.193	383.713
AMK58_09315	K00615 transketolase [EC:2.2.1.1] | (GenBank) transketolase	Metabolism	37.832	44.918	44.606	35.531	281.043	354.600
AMK58_09320	K00134 glyceraldehyde 3-phosphate dehydrogenase [EC:1.2.1.12] | (GenBank) gapA; glyceraldehyde-3-ph	Metabolism	25.945	35.163	26.838	26.548	63.996	49.452
AMK58_09325	no KO assigned | (GenBank) antitoxin HicB	Defense	66.679	60.804	72.742	104.363	136.488	148.066
AMK58_09330	no KO assigned | (GenBank) hypothetical protein	Hypothetical	1518.889	1249.580	1463.359	831.751	650.121	830.897
AMK58_09335	no KO assigned | (GenBank) hypothetical protein	Hypothetical	2716.800	2408.245	2610.766	212.951	622.377	365.105
AMK58_09340	K11645 fructose-bisphosphate aldolase, class I [EC:4.1.2.13] | (GenBank) fructose-bisphosphate aldol	Metabolism	29.943	42.001	30.822	27.903	147.654	67.603
AMK58_09345	K00788 thiamine-phosphate pyrophosphorylase [EC:2.5.1.3] | (GenBank) thiamine-phosphate synthase	Metabolism: Co-Factors and Vitamens	12.316	5.660	10.269	107.109	32.799	70.103
AMK58_09350	K07090 uncharacterized protein | (GenBank) hypothetical protein	Hypothetical	108.022	128.178	150.496	95.463	30.355	58.987
AMK58_09355	K06076 long-chain fatty acid transport protein | (GenBank) long-chain fatty acid transporter	Transport: Lipid	275.587	288.376	276.046	137.142	166.403	157.694
AMK58_09360	K19745 acrylyl-CoA reductase (NADPH) [EC:1.3.1.-] | (GenBank) NADPH:quinone dehydrogenase	Energy	89.834	108.939	106.104	131.410	62.581	127.600
AMK58_09365	K04568 elongation factor P--(R)-beta-lysine ligase [EC:6.3.1.-] | (GenBank) elongation factor P--(R)	Translation	24.836	24.043	30.720	182.517	41.099	177.734
AMK58_09370	K02356 elongation factor P | (GenBank) elongation factor P	translation	2181.695	1719.724	1962.504	650.174	412.120	588.475
AMK58_09375	K01092 myo-inositol-1(or 4)-monophosphatase [EC:3.1.3.25] | (GenBank) inositol monophosphatase	Metabolism	572.521	581.223	577.884	191.588	269.640	177.005

AMK58_09380	no KO assigned | (GenBank) hypothetical protein	Hypothetical	58.660	69.158	98.163	315.361	225.943	244.121
AMK58_09385	no KO assigned | (GenBank) flagellar motor protein MotA	Motility	477.980	395.166	373.846	42.731	374.829	114.065
AMK58_09390	K02557 chemotaxis protein MotB | (GenBank) chemotaxis protein	Chemotaxis	74.021	61.924	68.374	121.025	98.564	124.857
AMK58_09395	K00283 glycine dehydrogenase subunit 2 [EC:1.4.4.2] | (GenBank) glycine dehydrogenase	Metabolism	18.942	18.015	20.069	72.290	28.592	26.813
AMK58_09400	K00282 glycine dehydrogenase subunit 1 [EC:1.4.4.2] | (GenBank) glycine dehydrogenase	Metabolism	117.824	106.804	116.750	108.487	92.353	148.270
AMK58_09405	K02437 glycine cleavage system H protein | (GenBank) glycine cleavage system protein H	Metabolism	399.385	380.003	416.577	652.636	303.397	708.646
AMK58_09410	K00605 aminomethyltransferase [EC:2.1.2.10] | (GenBank) gcvT; glycine cleavage system protein T	Metabolism	1944.267	1630.637	1681.274	222.951	160.182	168.657
AMK58_09415	no KO assigned | (GenBank) glutamate synthase	enzyme	12260.052	10670.965	11291.951	1127.390	985.293	1012.709
AMK58_09420	no KO assigned | (GenBank) hypothetical protein	Hypothetical	1740.553	1657.075	1667.658	80.541	134.010	101.959
AMK58_09425	K03527 4-hydroxy-3-methylbut-2-en-1-yl diphosphate reductase [EC:1.17.7.4] | (GenBank) ispH; 4-hydro	Biosynthesis: Terepenoids	2379.899	2007.349	1832.486	178.603	321.169	202.499
AMK58_09430	K02204 homoserine kinase type II [EC:2.7.1.39] | (GenBank) homoserine kinase	Signal Transduction	50.448	55.134	40.245	63.787	18.256	55.793
AMK58_09435	K03469 ribonuclease HI [EC:3.1.26.4] | (GenBank) ribonuclease H	Genetic Information Processing	232.123	257.592	224.994	143.382	71.005	260.568
AMK58_09440	K02182 crotonobetaine/carnitine-CoA ligase [EC:6.2.1.-] | (GenBank) hypothetical protein	Enzyme	45.431	47.048	57.379	129.120	102.985	158.821
AMK58_09445	no KO assigned | (GenBank) alkyl hydroperoxide reductase	defense	109.544	142.275	95.575	517.114	149.054	393.990
AMK58_09450	K08344 suppressor for copper-sensitivity B | (GenBank) copper resistance protein	Transport: Metal	6.446	6.245	5.577	33.215	9.203	31.500
AMK58_09455	K07735 putative transcriptional regulator | (GenBank) hypothetical protein	Transcription	319.293	317.724	357.548	379.560	194.689	307.767
AMK58_09460	no KO assigned | (GenBank) hypothetical protein	Hypothetical	1677.821	1623.139	1660.333	275.584	520.584	348.219
AMK58_09465	K03790 ribosomal-protein-alanine N-acetyltransferase [EC:2.3.1.128] | (GenBank) GCN5 family acetyltr	Ribosome	404.197	375.753	421.324	784.584	425.781	373.662
AMK58_09470	no KO assigned | (GenBank) peptidase M16	peptidase	167.309	176.091	162.467	45.288	269.480	110.483
AMK58_09475	K01733 threonine synthase [EC:4.2.3.1] | (GenBank) threonine synthase	Metabolism: Amino Acid	23.649	22.525	23.301	11.937	44.883	45.845
AMK58_09480	K01179 endoglucanase [EC:3.2.1.4] | (GenBank) 1,4-beta-glucanase	Metabolism: Carbohydrate	17.674	15.240	14.033	106.049	64.309	61.077
AMK58_09485	K01299 carboxypeptidase Taq [EC:3.4.17.19] | (GenBank) peptidase M32	Peptidase	103.929	91.809	84.205	147.668	157.275	111.745
AMK58_09490	K14998 surfeit locus 1 family protein | (GenBank) hypothetical protein	No COG	296.396	262.595	258.202	357.641	183.294	295.901
AMK58_09495	no KO assigned | (GenBank) hypothetical protein	Hypothetical	34.224	46.295	42.364	500.397	223.556	830.796
AMK58_09500	K02276 cytochrome c oxidase subunit III [EC:1.9.3.1] | (GenBank) cytochrome B562	Energy	146.139	180.204	198.597	398.162	108.079	205.631
AMK58_09505	K02258 cytochrome c oxidase assembly protein subunit 11 | (GenBank) cytochrome C oxidase assembly pı	Energy	58.791	95.056	87.631	234.962	42.965	106.152
AMK58_09510	K02301 protoheme IX farnesyltransferase [EC:2.5.1.-] | (GenBank) protoheme IX farnesyltransferase	Energy	92.694	84.003	98.532	80.221	31.023	90.661
AMK58_09515	K02274 cytochrome c oxidase subunit I [EC:1.9.3.1] | (GenBank) cytochrome C oxidase subunit I	Energy	1051.490	990.916	1112.460	63.608	307.179	151.988
AMK58_09520	K02275 cytochrome c oxidase subunit II [EC:1.9.3.1] | (GenBank) cytochrome C oxidase subunit II	Energy	1064.221	1094.695	993.152	188.558	353.747	194.288
AMK58_09525	no KO assigned | (GenBank) hypothetical protein	Hypothetical	1272.824	1325.071	1475.897	122.670	362.305	110.172
AMK58_09530	K03568 TldD protein | (GenBank) tldD; protease TldD	Peptidase	5281.698	4690.209	4995.632	85.165	1285.957	238.867
AMK58_09535	K03567 glycine cleavage system transcriptional repressor | (GenBank) amino acid-binding protein	Transcription	7109.281	7520.142	7643.459	209.760	2513.965	455.280
AMK58_09540	no KO assigned | (GenBank) hypothetical protein	Hypothetical	32.660	24.391	39.427	110.624	33.696	120.598
AMK58_09545	no KO assigned | (GenBank) hypothetical protein	Hypothetical	701.510	661.939	791.895	298.106	1299.733	343.870
AMK58_09550	no KO assigned | (GenBank) hypothetical protein	Hypothetical	887.882	857.310	1065.916	611.844	1886.583	798.956
AMK58_09555	K03088 RNA polymerase sigma-70 factor, ECF subfamily | (GenBank) RNA polymerase subunit sigma-7(	Transcription	48.152	51.549	77.805	1106.220	1016.788	613.999
AMK58_09560	no KO assigned | (GenBank) lytic transglycosylase	transglycosylase	8.752	9.441	16.066	139.063	874.631	128.727
AMK58_09565	no KO assigned | (GenBank) hypothetical protein	Hypothetical	15.952	10.483	32.761	114.748	1277.017	203.535
AMK58_09570	K14224 tRNA Glu | (GenBank) tRNA-Glu	tRNA synthesis/modification	135.413	152.285	331.724	1303.889	11718.340	1756.429
AMK58_09575	K14224 tRNA Glu | (GenBank) tRNA-Glu	tRNA synthesis/modification	218.253	218.910	657.390	2428.520	1332.281	2031.195
AMK58_09580	K01620 threonine aldolase [EC:4.1.2.48] | (GenBank) threonine aldolase	Metabolism: Amino Acid	68.325	69.958	71.990	77.362	278.192	182.583
AMK58_09585	no KO assigned | (GenBank) hypothetical protein	Hypothetical	822.716	732.204	985.728	22.815	1171.151	156.560
AMK58_09590	K06180 23S rRNA pseudouridine1911/1915/1917 synthase [EC:5.4.99.23] | (GenBank) RNA pseudourid	Ribosome	137.862	112.659	161.473	6.623	182.492	160.994
AMK58_09595	K03089 RNA polymerase sigma-32 factor | (GenBank) RNA polymerase subunit sigma-70	Transcription	35.980	37.598	46.048	282.038	62.126	131.425
AMK58_09600	no KO assigned | (GenBank) hypothetical protein	Hypothetical	30.269	27.400	23.329	44.540	25.260	20.413
AMK58_09605	no KO assigned | (GenBank) serine/threonine protein kinase	Signal Transduction	25.433	38.879	36.046	109.963	42.680	98.208
AMK58_09610	K01939 adenylosuccinate synthase [EC:6.3.4.4] | (GenBank) adenylosuccinate synthetase	Metabolism	138.866	153.846	164.799	74.633	572.791	208.042
AMK58_09615	K02502 ATP phosphoribosyltransferase regulatory subunit | (GenBank) ATP phosphoribosyltransferase re	Metabolism: Amino Acid	40.358	44.122	40.252	99.624	107.214	125.869
AMK58_09620	no KO assigned | (GenBank) hypothetical protein	Hypothetical	36.535	38.494	38.575	229.906	106.885	225.524
AMK58_09625	K03498 trk system potassium uptake protein | (GenBank) potassium transporter TrkH	Transport: Inorganic	265.460	252.551	267.040	189.100	204.726	165.985
AMK58_09630	K00831 phosphoserine aminotransferase [EC:2.6.1.52] | (GenBank) phosphoserine aminotransferase	Metabolism: Amino Acid	48.180	51.713	55.824	183.140	90.260	85.580
AMK58_09635	no KO assigned | (GenBank) hypothetical protein	Hypothetical	66.447	69.982	59.635	294.417	52.307	247.053
AMK58_09640	no KO assigned | (GenBank) hypothetical protein	Hypothetical	30.358	30.584	29.544	201.417	48.576	248.053
AMK58_09645	K07058 membrane protein | (GenBank) hypothetical protein	Membrane	191.005	209.435	206.308	206.134	113.158	164.696
AMK58_09650	K00208 enoyl-[acyl-carrier protein] reductase I [EC:1.3.1.9 1.3.1.10] | (GenBank) enoyl-ACP reductas	Metabolism: Lipid	16.257	11.928	20.610	307.561	75.500	247.549
AMK58_09655	no KO assigned | (GenBank) hypothetical protein	Hypothetical	56.273	61.523	49.944	88.617	103.005	215.087
AMK58_09660	K01736 chorismate synthase [EC:4.2.3.5] | (GenBank) chorismate synthase	Biosynthesis: Amino Acid	157.723	166.567	171.770	185.979	334.989	204.003
AMK58_09665	no KO assigned | (GenBank) hypothetical protein	Hypothetical	359.613	393.404	373.228	283.398	541.030	380.141
AMK58_09670	no KO assigned | (GenBank) regulator	signal transduction	89.783	93.805	73.487	88.869	66.244	276.932
AMK58_09675	no KO assigned | (GenBank) alkylhydroperoxidase	enzyme	75.223	85.843	82.170	325.504	106.404	319.567
AMK58_09680	K04773 protease IV [EC:3.4.21.-] | (GenBank) signal peptide peptidase SppA	Peptidases	9.744	7.019	8.082	44.620	27.110	50.111
AMK58_09685	no KO assigned | (GenBank) hypothetical protein	Hypothetical	115.661	111.738	143.665	1039.044	328.062	863.472
AMK58_09690	K13924 two-component system, chemotaxis family, CheB/CheR fusion protein [EC:2.1.1.80 3.1.1.61] | (G	Chemotaxis	68.124	73.807	68.777	20.273	31.032	41.806
AMK58_09695	K20975 two-component system, sensor histidine kinase [EC:2.7.13.3] | (GenBank) histidine kinase	Signal Transduction	89.511	95.709	95.729	126.145	60.860	86.968
AMK58_09700	no KO assigned | (GenBank) hypothetical protein	Hypothetical	193.265	254.703	232.940	215.486	258.769	485.448
AMK58_09705	K02069 putative ABC transport system permease protein | (GenBank) ABC transporter permease	Membrane Transport	248.625	221.325	225.083	371.777	403.371	806.275
AMK58_09710	no KO assigned | (GenBank) ATP-binding protein	No COG	214.698	242.342	269.000	808.734	660.662	624.607

AMK58_09715	no KO assigned | (GenBank) transporter	Transport	14.761	15.949	14.110	21.772	89.758	45.141
AMK58_09720	no KO assigned | (GenBank) hypothetical protein	Hypothetical	8.385	8.015	12.968	136.278	33.803	60.230
AMK58_09725	no KO assigned | (GenBank) histidine kinase	Signal Transduction	8.228	9.831	11.199	109.632	19.956	44.892
AMK58_09730	no KO assigned | (GenBank) XRE family transcriptional regulator	Transcription	145.004	134.429	154.680	462.876	145.126	388.473
AMK58_09735	K04771 serine protease Do [EC:3.4.21.107] | (GenBank) serine protease	Defense	8.087	8.362	11.904	130.103	68.353	150.126
AMK58_09740	no KO assigned | (GenBank) hypothetical protein	Hypothetical	100.367	108.566	131.794	571.189	255.086	449.471
AMK58_09745	K02355 elongation factor G | (GenBank) fusA; elongation factor G	Translation factors	53.228	56.992	56.728	58.901	68.713	54.695
AMK58_09750	no KO assigned | (GenBank) hypothetical protein	Hypothetical	20.179	33.258	38.042	1121.320	1721.110	1085.919
AMK58_09755	K04775 protease YdgD [EC:3.4.21.-] | (GenBank) hypothetical protein	Hydrolase	61.261	87.417	88.365	171.017	100.669	168.628
AMK58_09760	K03169 DNA topoisomerase III [EC:5.99.1.2] | (GenBank) DNA topoisomerase III	DNA Repair and Replication	1928.843	1497.592	1531.670	73.681	1294.715	240.334
AMK58_09765	no KO assigned | (GenBank) hypothetical protein	Hypothetical	1053.889	998.231	849.322	71.722	559.192	225.038
AMK58_09770	no KO assigned | (GenBank) hypothetical protein	Hypothetical	21.041	22.669	35.093	314.206	46.140	194.153
AMK58_09775	no KO assigned | (GenBank) hypothetical protein	Hypothetical	132.269	161.919	158.954	400.729	318.710	507.809
AMK58_09780	no KO assigned | (GenBank) glycosyl transferase	Glycosyl Transferase	0.320	0.546	1.172	34.675	5.748	47.668
AMK58_09785	no KO assigned | (GenBank) hypothetical protein	Hypothetical	3.669	8.220	3.052	289.969	72.686	198.598
AMK58_09790	K10773 endonuclease III [EC:4.2.99.18] | (GenBank) Fe-S cluster assembly protein HesB	DNA Repair and Replication	0.619	3.695	4.852	318.779	25.724	73.413
AMK58_09795	no KO assigned | (GenBank) hypothetical protein	Hypothetical	88.340	70.996	138.782	812.731	372.492	719.773
AMK58_09800	no KO assigned | (GenBank) RNA helicase	No COG	0.571	0.853	0.780	31.883	22.542	18.960
AMK58_09805	no KO assigned | (GenBank) hypothetical protein	Hypothetical	11.478	13.273	18.482	276.262	393.713	196.043
AMK58_09810	no KO assigned | (GenBank) hypothetical protein	Hypothetical	10.857	8.460	14.811	90.103	102.402	43.770
AMK58_09815	K14219 tRNA Arg | (GenBank) tRNA-Arg	tRNA synthesis/modification	479.582	507.288	653.337	6902.378	2310.049	7144.434
AMK58_09820	K00619 amino-acid N-acetyltransferase [EC:2.3.1.1] | (GenBank) hypothetical protein	Biosynthesis: Amino Acid	201.287	226.049	230.957	138.534	145.166	222.434
AMK58_09825	K14230 tRNA Met | (GenBank) tRNA-Met	tRNA synthesis/modification	1113.569	1189.730	1201.175	1553.570	891.769	1756.429
AMK58_09830	K03086 RNA polymerase primary sigma factor | (GenBank) RNA polymerase subunit sigma	Transcription	2.839	3.687	4.577	5.786	14.428	17.453
AMK58_09835	K02316 DNA primase [EC:2.7.7.-] | (GenBank) DNA primase	DNA Repair and Replication	8.510	8.598	10.352	43.333	30.243	30.751
AMK58_09840	K09117 uncharacterized protein | (GenBank) glutamyl-tRNA amidotransferase	tRNA synthesis/modification	2.097	4.697	1.246	8.074	20.031	31.591
AMK58_09845	K01956 carbamoyl-phosphate synthase small subunit [EC:6.3.5.5] | (GenBank) carbamoyl phosphate synth	Metabolism	1046.638	1185.909	1093.826	134.879	6059.966	424.544
AMK58_09850	no KO assigned | (GenBank) hypothetical protein	Hypothetical	107.027	164.302	117.156	116.895	708.791	107.044
AMK58_09855	no KO assigned | (GenBank) restriction endonuclease	DNA Repair and Replication	287.034	324.496	264.307	116.208	1220.175	291.342
AMK58_09860	no KO assigned | (GenBank) hypothetical protein	Hypothetical	836.924	752.582	789.553	520.415	3495.350	470.814
AMK58_09865	K00058 D-3-phosphoglycerate dehydrogenase [EC:1.1.1.95] | (GenBank) D-3-phosphoglycerate dehydrog	Metabolism	84.888	89.840	121.022	486.428	208.938	375.312
AMK58_09870	K00537 arsenate reductase [EC:1.20.4.1] | (GenBank) arsenate reductase	enzyme	18.055	21.151	18.513	168.825	301.633	153.304
AMK58_09875	K07320 ribosomal protein L3 glutamine methyltransferase [EC:2.1.1.298] | (GenBank) protein-(glutamin	Ribosome	44.343	51.449	52.793	220.036	285.044	152.553
AMK58_09880	no KO assigned | (GenBank) GCN5 family acetyltransferase	Acetyltransferase	638.357	580.153	549.104	159.442	338.736	410.136
AMK58_09885	no KO assigned | (GenBank) hypothetical protein	Hypothetical	92.503	98.154	82.519	231.557	94.342	308.328
AMK58_09890	K17103 CDP-diacylglycerol---serine O-phosphatidyltransferase [EC:2.7.8.8] | (GenBank) CDP-diacylglyc	Metabolism	170.624	155.971	166.916	169.501	164.839	233.308
AMK58_09895	K01613 phosphatidylserine decarboxylase [EC:4.1.1.65] | (GenBank) phosphatidylserine decarboxylase	Metabolism: Lipid	74.654	99.345	88.933	165.434	188.467	198.680
AMK58_09900	no KO assigned | (GenBank) hypothetical protein	Hypothetical	140.603	183.561	152.749	149.281	520.081	201.603
AMK58_09905	no KO assigned | (GenBank) peptidoglycan-binding protein	Cell Wall	123.098	137.686	121.746	187.636	410.807	172.418
AMK58_09910	no KO assigned | (GenBank) hypothetical protein	Hypothetical	890.034	829.448	761.320	616.306	1180.684	847.761
AMK58_09915	K06966 uncharacterized protein | (GenBank) lysine decarboxylase	No COG	32.908	32.149	41.522	161.909	92.357	220.317
AMK58_09920	K00031 isocitrate dehydrogenase [EC:1.1.1.42] | (GenBank) isocitrate dehydrogenase	Metabolism	85.687	113.433	126.272	121.307	122.685	84.894
AMK58_09925	no KO assigned | (GenBank) oxidoreductase	Oxidoreductase	14.300	13.290	19.338	113.232	75.726	180.633
AMK58_09930	no KO assigned | (GenBank) hypothetical protein	Hypothetical	76.939	95.022	88.466	196.274	81.790	191.172
AMK58_09935	K13583 GcrA cell cycle regulator | (GenBank) global cell cycle regulator GcrA-like protein	Cell Cycle/Shape/Homeostatsis	7631.810	6860.575	6429.273	230.186	3000.784	730.338
AMK58_09940	no KO assigned | (GenBank) transcriptional regulator	Transcription	33.020	40.186	28.780	131.878	56.705	111.844
AMK58_09945	K03088 RNA polymerase sigma-70 factor, ECF subfamily | (GenBank) RNA polymerase subunit sigma-70	Transcription	149.902	144.958	159.887	317.292	459.585	231.222
AMK58_09950	K00031 isocitrate dehydrogenase [EC:1.1.1.42] | (GenBank) isocitrate dehydrogenase	Metabolism	234.363	285.140	279.438	52.043	83.011	84.785
AMK58_09955	no KO assigned | (GenBank) hypothetical protein	Hypothetical	21.440	36.168	28.780	183.135	125.603	251.671
AMK58_09960	no KO assigned | (GenBank) antibiotic biosynthesis monooxygenase	Defense	11.531	25.834	19.872	543.516	78.578	195.804
AMK58_09965	no KO assigned | (GenBank) hypothetical protein	Hypothetical	60.623	70.797	75.603	409.261	212.344	379.328
AMK58_09970	K03406 methyl-accepting chemotaxis protein | (GenBank) chemotaxis protein	Chemotaxis	20.465	28.216	23.949	26.456	48.099	55.405
AMK58_09975	K09989 uncharacterized protein | (GenBank) hypothetical protein	Hypothetical	53.845	56.342	71.636	30.019	95.174	64.973
AMK58_09980	no KO assigned | (GenBank) phosphohydrolase	No COG	76.768	102.213	102.815	111.944	71.745	315.860
AMK58_09985	no KO assigned | (GenBank) cytochrome c oxidase accessory protein CcoG	Energy	41.502	41.488	72.629	226.535	78.332	256.432
AMK58_09990	no KO assigned | (GenBank) permease	enzyme	335.752	305.029	354.603	327.954	333.401	732.909
AMK58_09995	no KO assigned | (GenBank) hypothetical protein	Hypothetical	41.048	63.886	39.685	226.413	124.849	398.861
AMK58_10000	K01754 threonine dehydratase [EC:4.3.1.19] | (GenBank) threonine dehydratase	Metabolism: Amino Acid	120.949	153.507	162.787	305.400	143.701	299.851
AMK58_10005	no KO assigned | (GenBank) AAA family ATPase	enzyme	8.444	11.970	13.199	91.254	43.434	88.648
AMK58_10010	no KO assigned | (GenBank) pseudogene	Pseudogene	19.624	28.757	28.868	219.720	73.665	136.990
AMK58_10015	K01872 alanyl-tRNA synthetase [EC:6.1.1.7] | (GenBank) alanine--tRNA ligase	tRNA synthesis/modification	0.453	1.355	1.293	70.402	12.743	41.922
AMK58_10020	K03553 recombination protein RecA | (GenBank) DNA recombination/repair protein RecA	DNA Repair and Replication	108.855	101.806	92.308	83.496	250.764	206.763
AMK58_10025	no KO assigned | (GenBank) hypothetical protein	Hypothetical	8.648	12.917	11.192	69.508	82.088	71.282
AMK58_10030	K13587 two-component system, cell cycle sensor histidine kinase and response regulator CckA [EC:2.7.	Signal Transduction	185.734	211.337	210.368	155.573	760.096	199.739
AMK58_10035	K02401 flagellar biosynthetic protein FlhB | (GenBank) flagellar biosynthetic protein FlhB	Motility	293.750	381.491	340.654	180.257	262.709	256.542
AMK58_10040	K02421 flagellar biosynthetic protein FliR | (GenBank) flagellar biosynthetic protein FliR	Motility	10.521	7.506	15.379	109.807	71.489	183.184
AMK58_10045	K02420 flagellar biosynthetic protein FliQ | (GenBank) flagellar biosynthesis protein	Motility	508.439	635.471	605.002	1276.266	935.694	1080.727

AMK58_10050	K02408 flagellar hook-basal body complex protein FliE | (GenBank) flagellar hook-basal body protein	Motility	242.553	248.352	227.935	451.958	123.391	897.035
AMK58_10055	K02388 flagellar basal-body rod protein FlgC | (GenBank) flagellar basal-body rod protein FlgC	Motility	141.107	142.559	152.091	119.568	66.557	308.127
AMK58_10060	K02387 flagellar basal-body rod protein FlgB | (GenBank) flagellar biosynthesis protein FlgB	Motility	611.652	764.435	668.827	30.835	68.215	97.300
AMK58_10065	K04061 flagellar biosynthesis protein | (GenBank) flagellar protein FhlB	Motility	393.586	506.570	491.456	67.949	38.705	165.486
AMK58_10070	no KO assigned | (GenBank) hypothetical protein	Hypothetical	888.522	1090.774	966.938	89.674	45.724	209.392
AMK58_10075	K02418 flagellar protein FliO/FliZ | (GenBank) hypothetical protein	Motility	785.545	923.570	852.428	425.738	38.884	340.485
AMK58_10080	K02419 flagellar biosynthetic protein FliP | (GenBank) flagellar biosynthesis protein flip	Motility	47.725	62.193	54.514	37.758	12.781	93.855
AMK58_10085	K03549 KUP system potassium uptake protein | (GenBank) trkD; potassium transport protein Kup	Transport: Inorganic	1.658	2.285	1.455	10.590	0.430	1.655
AMK58_10090	no KO assigned | (GenBank) hypothetical protein	Hypothetical	1.348	1.271	2.327	13.254	0.678	3.486
AMK58_10095	K02557 chemotaxis protein MotB | (GenBank) flagellar motor protein MotB	Chemotaxis	46.757	53.909	52.412	68.566	24.711	181.792
AMK58_10100	no KO assigned | (GenBank) pseudogene	Pseudogene	152.338	134.709	164.662	461.938	343.534	589.811
AMK58_10105	K03414 chemotaxis protein CheZ | (GenBank) chemotaxis protein CheZ	Chemotaxis	117.529	168.480	165.213	962.484	212.586	529.092
AMK58_10110	K06884 uncharacterized protein | (GenBank) glucose starvation-inducible protein B	Starvation	67.145	76.817	79.802	668.837	110.802	292.100
AMK58_10115	no KO assigned | (GenBank) hypothetical protein	Hypothetical	16.900	20.344	20.386	149.515	12.759	70.753
AMK58_10120	no KO assigned | (GenBank) hypothetical protein	Hypothetical	236.530	258.414	243.029	220.810	96.280	203.696
AMK58_10125	K02416 flagellar motor switch protein FliM | (GenBank) flagellar motor switch protein FliM	Motility	9.279	12.023	9.886	52.714	336.652	133.417
AMK58_10130	K02415 flagellar FliL protein | (GenBank) flagellar basal body protein FliL	Motility	136.383	167.674	134.085	236.754	66.482	322.184
AMK58_10135	K02391 flagellar basal-body rod protein FlgF | (GenBank) flagellar biosynthesis protein FlgF	Motility	30.478	49.024	38.851	65.502	12.423	64.442
AMK58_10140	K02392 flagellar basal-body rod protein FlgG | (GenBank) flgG; flagellar basal-body rod protein FlgG	Motility	121.383	152.770	142.068	170.233	33.417	315.955
AMK58_10145	K02386 flagella basal body P-ring formation protein FlgA | (GenBank) flagellin biosynthesis protein	Motility	168.237	180.146	176.427	54.476	17.555	121.733
AMK58_10150	K02393 flagellar L-ring protein precursor FlgH | (GenBank) flagellar biosynthesis protein FlgH	Motility	161.747	206.405	214.411	138.626	33.538	197.746
AMK58_10155	no KO assigned | (GenBank) hypothetical protein	Hypothetical	33.193	41.429	38.021	78.709	9.769	35.537
AMK58_10160	K06204 DnaK suppressor protein | (GenBank) RNA polymerase-binding protein DksA	Transcription	82.158	98.170	114.023	415.119	58.974	171.345
AMK58_10165	no KO assigned | (GenBank) flagellar assembly protein FliX	Motility	231.987	216.833	214.226	295.591	128.913	395.502
AMK58_10170	K02394 flagellar P-ring protein precursor FlgI | (GenBank) flgI; flagellar biosynthesis protein FlgA	Motility	68.652	53.582	72.685	407.467	183.018	264.339
AMK58_10175	no KO assigned | (GenBank) flagellar biosynthesis protein FlgJ	Motility	854.861	1261.610	1060.102	268.672	3085.696	374.232
AMK58_10180	no KO assigned | (GenBank) flagellar basal-body protein	Motility	119.902	173.830	147.914	126.040	652.245	129.458
AMK58_10185	no KO assigned | (GenBank) hypothetical protein	Hypothetical	61.169	70.431	86.239	88.384	51.179	105.689
AMK58_10190	K02389 flagellar basal-body rod modification protein FlgD | (GenBank) flagellar biosynthesis protein	Motility	35.542	40.683	34.741	79.424	42.377	27.105
AMK58_10195	no KO assigned | (GenBank) hypothetical protein	Hypothetical	117.916	157.251	165.171	192.273	71.794	120.731
AMK58_10200	no KO assigned | (GenBank) dehydratase	dehydratase	266.311	333.124	316.072	664.037	617.791	404.516
AMK58_10205	K14229 tRNA Lys | (GenBank) tRNA-Lys	tRNA synthesis/modification	2665.238	3159.922	2896.150	862.146	3905.517	3139.119
AMK58_10210	K14229 tRNA Lys | (GenBank) tRNA-Lys	tRNA synthesis/modification	1108.790	1637.068	1161.792	544.176	96.698	1144.855
AMK58_10215	no KO assigned | (GenBank) hypothetical protein	Hypothetical	4.519	1.895	7.237	65.427	26.381	69.773
AMK58_10220	no KO assigned | (GenBank) hypothetical protein	Hypothetical	96.647	115.120	108.483	21.543	45.705	75.972
AMK58_10225	K06147 ATP-binding cassette, subfamily B, bacterial | (GenBank) ABC transporter	Membrane Transport	48.282	54.161	54.144	6.836	23.928	96.516
AMK58_10230	K02909 large subunit ribosomal protein L31 | (GenBank) 50S ribosomal protein L31	Ribosome	71.569	80.373	95.165	1533.197	399.812	698.567
AMK58_10235	K13592 regulator of CtrA degradation | (GenBank) hypothetical protein	Cell Cycle/Shape/Homeostatsis	11.870	10.638	15.801	469.465	309.879	298.285
AMK58_10240	K04080 molecular chaperone IbpA | (GenBank) heat-shock protein	Chaperone	323.652	248.947	318.695	741.070	916.420	693.545
AMK58_10245	no KO assigned | (GenBank) hypothetical protein	Hypothetical	342.066	265.699	350.076	34.292	152.242	32.518
AMK58_10250	no KO assigned | (GenBank) alpha/beta hydrolase	Hydrolase	15.021	19.942	24.957	117.270	110.341	130.700
AMK58_10255	K01740 O-acetylhomoserine (thiol)-lyase [EC:2.5.1.49] | (GenBank) O-acetylhomoserine aminocarboxypro	Metabolism: Amino Acid	218.969	243.326	264.396	78.557	10549.558	637.736
AMK58_10260	K06048 carboxylate-amine ligase [EC:6.3.-.-] | (GenBank) carboxylate--amine ligase	enzyme	71.724	77.388	81.733	172.511	152.338	335.022
AMK58_10265	no KO assigned | (GenBank) N-formylglutamate amidohydrolase	enzyme	119.411	111.031	120.657	148.252	31.334	98.252
AMK58_10270	no KO assigned | (GenBank) hypothetical protein	Hypothetical	466.457	372.253	407.292	230.285	111.262	306.214
AMK58_10275	no KO assigned | (GenBank) transcriptional regulator	Transcription	87.417	96.820	101.933	183.685	109.996	275.400
AMK58_10280	K00939 adenylate kinase [EC:2.7.4.3] | (GenBank) adenylate kinase	Metabolism	56.614	79.257	86.339	342.643	79.808	147.614
AMK58_10285	K03076 preprotein translocase subunit SecY | (GenBank) preprotein translocase subunit SecY	Secretion system	165.225	178.007	192.380	110.301	149.388	188.288
AMK58_10290	K02876 large subunit ribosomal protein L15 | (GenBank) 50S ribosomal protein L15	Ribosome	453.159	501.586	501.691	395.453	273.306	475.876
AMK58_10295	K02907 large subunit ribosomal protein L30 | (GenBank) 50S ribosomal protein L30	Ribosome	2751.298	3055.424	2391.116	628.470	939.894	709.287
AMK58_10300	K02988 small subunit ribosomal protein S5 | (GenBank) 30S ribosomal protein S5	Ribosome	1691.302	1240.393	1256.023	282.014	335.089	623.463
AMK58_10305	K02881 large subunit ribosomal protein L18 | (GenBank) 50S ribosomal protein L18	Ribosome	2679.989	2542.704	2738.435	165.760	926.783	407.635
AMK58_10310	K02933 large subunit ribosomal protein L6 | (GenBank) 50S ribosomal protein L6	Ribosome	605.147	456.500	413.695	50.721	197.259	55.294
AMK58_10315	K02994 small subunit ribosomal protein S8 | (GenBank) 30S ribosomal protein S8	Ribosome	1806.715	1816.547	1803.242	327.624	650.450	481.156
AMK58_10320	K02954 small subunit ribosomal protein S14 | (GenBank) 30S ribosomal protein S14	Ribosome	1839.466	1609.821	1415.286	99.644	611.975	351.485
AMK58_10325	K02931 large subunit ribosomal protein L5 | (GenBank) 50S ribosomal protein L5	Ribosome	2370.212	2135.431	1752.013	33.751	615.552	182.567
AMK58_10330	K02895 large subunit ribosomal protein L24 | (GenBank) 50S ribosomal protein L24	Ribosome	3890.765	2806.984	2645.926	112.204	737.813	314.920
AMK58_10335	K02874 large subunit ribosomal protein L14 | (GenBank) 50S ribosomal protein L14	Ribosome	3267.379	2816.970	2469.598	32.086	535.890	149.693
AMK58_10340	K02961 small subunit ribosomal protein S17 | (GenBank) 30S ribosomal protein S17	Ribosome	6833.841	5840.742	5353.256	158.370	1258.462	601.853
AMK58_10345	K02904 large subunit ribosomal protein L29 | (GenBank) 50S ribosomal protein L29	Ribosome	4135.844	3931.280	3531.426	47.010	784.344	277.149
AMK58_10350	K02878 large subunit ribosomal protein L16 | (GenBank) 50S ribosomal protein L16	Ribosome	1779.715	1652.421	1554.508	31.001	374.653	124.308
AMK58_10355	K02982 small subunit ribosomal protein S3 | (GenBank) 30S ribosomal protein S3	Ribosome	1004.905	809.004	749.829	11.689	179.881	52.595
AMK58_10360	K02890 large subunit ribosomal protein L22 | (GenBank) 50S ribosomal protein L22	Ribosome	956.063	1004.660	852.345	36.041	200.016	68.609
AMK58_10365	K02965 small subunit ribosomal protein S19 | (GenBank) 30S ribosomal protein S19	Ribosome	2316.043	1778.573	2023.865	242.988	673.637	283.692
AMK58_10370	K02886 large subunit ribosomal protein L2 | (GenBank) rplB; 50S ribosomal protein L2	Ribosome	1371.888	1068.435	1244.063	134.763	484.709	218.980
AMK58_10375	K02892 large subunit ribosomal protein L23 | (GenBank) 50S ribosomal protein L23	Ribosome	3171.550	2274.703	2421.480	121.384	910.284	259.491
AMK58_10380	K02926 large subunit ribosomal protein L4 | (GenBank) 50S ribosomal protein L4	Ribosome	914.592	608.038	754.298	38.131	266.269	150.054

AMK58_10385	K02906 large subunit ribosomal protein L3 | (GenBank) 50S ribosomal protein L3	Ribosome	2376.540	1835.349	2094.161	123.407	780.382	295.912
AMK58_10390	K02946 small subunit ribosomal protein S10 | (GenBank) rpsJ; 30S ribosomal protein S10	Ribosome	2860.735	2069.406	2195.829	37.791	827.130	167.860
AMK58_10395	K02358 elongation factor Tu | (GenBank) tuf; elongation factor Tu	translation	1042.707	725.178	881.612	65.909	347.032	100.769
AMK58_10400	K02355 elongation factor G | (GenBank) fusA; elongation factor G	translation	657.554	514.943	646.416	38.850	231.797	75.656
AMK58_10405	K02992 small subunit ribosomal protein S7 | (GenBank) 30S ribosomal protein S7	Ribosome	2137.703	1862.909	1788.621	40.633	704.737	128.478
AMK58_10410	K02950 small subunit ribosomal protein S12 | (GenBank) 30S ribosomal protein S12	Ribosome	1574.949	1405.877	1487.881	25.723	686.319	67.000
AMK58_10415	K18199 cyclohexyl-isocyanide hydratase [EC:4.2.1.103] | (GenBank) AraC family transcriptional regula	Transcription	2153.402	1946.171	1827.553	223.062	1104.947	803.184
AMK58_10420	K03046 DNA-directed RNA polymerase subunit beta' [EC:2.7.7.6] | (GenBank) DNA-directed RNA poly	Transcription	242.004	196.885	218.229	17.424	71.898	23.127
AMK58_10425	K03043 DNA-directed RNA polymerase subunit beta [EC:2.7.7.6] | (GenBank) DNA-directed RNA polyr	Transcription	329.737	272.854	289.774	4.022	92.254	13.224
AMK58_10430	K02935 large subunit ribosomal protein L7/L12 | (GenBank) 50S ribosomal protein L7	Ribosome	233.432	223.758	245.894	312.694	164.763	316.451
AMK58_10435	K02864 large subunit ribosomal protein L10 | (GenBank) 50S ribosomal protein L10	Ribosome	6419.063	5678.132	6150.767	844.679	5778.847	1858.831
AMK58_10440	K02863 large subunit ribosomal protein L1 | (GenBank) 50S ribosomal protein L1	Ribosome	4420.265	3782.676	4037.198	706.964	4773.537	1610.478
AMK58_10445	K02867 large subunit ribosomal protein L11 | (GenBank) 50S ribosomal protein L11	Ribosome	2807.265	2221.616	2158.943	158.085	1614.973	129.304
AMK58_10450	K02601 transcriptional antiterminator NusG | (GenBank) antitermination protein NusG	Transcription	3478.791	3411.078	2960.356	83.384	2870.003	1476.843
AMK58_10455	K03073 preprotein translocase subunit SecE | (GenBank) preprotein translocase subunit SecE	Secretion System	3179.130	3021.288	3258.796	641.371	5157.107	928.209
AMK58_10460	K14235 tRNA Trp | (GenBank) tRNA-Trp	tRNA synthesis/modification	10018.939	8642.195	8065.899	243.279	9927.640	1223.149
AMK58_10465	K02358 elongation factor Tu | (GenBank) tuf; elongation factor Tu	translation	306.296	290.921	281.950	21.652	509.635	111.044
AMK58_10470	K14225 tRNA Gly | (GenBank) tRNA-Gly	tRNA synthesis/modification	2949.971	2688.146	2557.507	105.202	2986.694	321.637
AMK58_10475	K14236 tRNA Tyr | (GenBank) tRNA-Tyr	tRNA synthesis/modification	2069.534	1741.100	1499.223	60.348	1783.454	375.973
AMK58_10480	K03218 23S rRNA (guanosine2251-2'-O)-methyltransferase [EC:2.1.1.185] | (GenBank) pseudouridine sy	Ribosome	1110.773	939.939	798.562	17.641	931.960	49.372
AMK58_10485	K14234 tRNA Thr | (GenBank) tRNA-Thr	tRNA synthesis/modification	4608.807	3921.349	3289.978	142.980	4247.540	502.259
AMK58_10490	no KO assigned | (GenBank) hypothetical protein	Hypothetical	269.583	200.144	177.318	46.642	275.744	194.699
AMK58_10495	no KO assigned | (GenBank) hypothetical protein	Hypothetical	72.734	100.687	94.878	639.832	111.168	459.360
AMK58_10500	K01991 polysaccharide biosynthesis/export protein | (GenBank) polysaccharide biosynthesis protein	Transport: polysacchharide	3.592	5.050	3.616	32.267	12.351	36.835
AMK58_10505	no KO assigned | (GenBank) hypothetical protein	Hypothetical	5.567	7.760	7.939	48.966	27.427	77.858
AMK58_10510	no KO assigned | (GenBank) MarR family transcriptional regulator	Transcription	3.844	3.281	7.831	108.124	145.351	94.449
AMK58_10515	K01999 branched-chain amino acid transport system substrate-binding protein | (GenBank) ABC transpor	Transport: Amino Acid	44.493	55.281	106.508	73.445	98.806	83.609
AMK58_10520	K01997 branched-chain amino acid transport system permease protein K01998 branched-chain amino acid	Transport: Amino Acid	625.356	540.357	512.723	138.237	73.218	182.728
AMK58_10525	K01995 branched-chain amino acid transport system ATP-binding protein | (GenBank) ABC transporter A'	Transport: Amino Acid	88.496	78.436	86.455	91.623	96.923	121.738
AMK58_10530	K01996 branched-chain amino acid transport system ATP-binding protein | (GenBank) ABC transporter A'	Transport: Amino Acid	137.792	129.135	129.248	370.517	290.218	324.282
AMK58_10535	no KO assigned | (GenBank) hypothetical protein	Hypothetical	99.419	88.214	94.997	1153.102	469.134	587.818
AMK58_10540	K01087 trehalose 6-phosphate phosphatase [EC:3.1.3.12] | (GenBank) trehalose phosphatase	Metabolism: Carbohydrate	7.338	8.768	10.326	209.564	49.323	100.566
AMK58_10545	K01443 N-acetylglucosamine-6-phosphate deacetylase [EC:3.5.1.25] | (GenBank) N-acetylglucosamine-6-p	Metabolism: Carbohydrate	22.176	17.582	22.684	110.658	61.431	55.258
AMK58_10550	no KO assigned | (GenBank) hypothetical protein	Hypothetical	51.365	46.032	56.513	208.384	85.368	67.362
AMK58_10555	K00847 fructokinase [EC:2.7.1.4] | (GenBank) hypothetical protein	Metabolism: Carbohydrates	12.377	8.037	14.070	215.707	135.791	164.912
AMK58_10560	no KO assigned | (GenBank) hypothetical protein	Hypothetical	4.754	5.621	7.063	84.708	7.904	54.367
AMK58_10565	K03406 methyl-accepting chemotaxis protein | (GenBank) chemotaxis protein	Chemotaxis	2.648	1.283	2.926	19.554	11.180	33.641
AMK58_10570	no KO assigned | (GenBank) hypothetical protein	Hypothetical	2.420	5.163	5.752	107.082	6.314	41.670
AMK58_10575	K11963 urea transport system ATP-binding protein | (GenBank) urea ABC transporter ATP-binding subur	Transport: Inorganic	65.756	70.673	142.245	588.856	394.788	317.936
AMK58_10580	K11962 urea transport system ATP-binding protein | (GenBank) urea ABC transporter ATP-binding protei	Transport: Inorganic	12.804	14.525	22.962	346.300	116.781	609.142
AMK58_10585	K11961 urea transport system permease protein | (GenBank) urea ABC transporter permease subunit UrtC	Transport: Inorganic	259.355	290.611	278.507	267.607	251.772	223.358
AMK58_10590	K11960 urea transport system permease protein | (GenBank) urea ABC transporter permease	Transport: Inorganic	1.056	0.901	3.228	105.901	6.359	65.823
AMK58_10595	K11959 urea transport system substrate-binding protein | (GenBank) branched-chain amino acid ABC tra	Transport: Inorganic	0.826	3.836	3.401	102.963	18.146	108.612
AMK58_10600	no KO assigned | (GenBank) endonuclease	DNA Repair and Replication	2.663	8.372	7.328	269.184	33.708	282.233
AMK58_10605	no KO assigned | (GenBank) hypothetical protein	Hypothetical	2.890	4.933	4.809	238.647	22.624	238.800
AMK58_10610	no KO assigned | (GenBank) oxidoreductase	Oxidoreductase	17.762	18.837	15.289	155.850	112.938	242.179
AMK58_10615	no KO assigned | (GenBank) hypothetical protein	Hypothetical	42.722	52.677	41.311	110.316	26.360	161.662
AMK58_10620	K02049 NitT/TauT family transport system ATP-binding protein | (GenBank) ABC transporter ATP-bindi	Transport: NitT/TauT	156.650	138.420	158.040	337.787	85.352	210.956
AMK58_10625	K02051 NitT/TauT family transport system substrate-binding protein | (GenBank) nitrate ABC transport	Transport: NitT/TauT	207.730	199.122	199.586	190.110	79.694	218.358
AMK58_10630	K02050 NitT/TauT family transport system permease protein | (GenBank) ABC transporter permease	Transport: NitT/TauT	75.150	85.916	75.864	433.738	58.748	172.204
AMK58_10635	no KO assigned | (GenBank) TetR family transcriptional regulator	Transcription	436.798	426.758	407.501	193.283	190.089	359.826
AMK58_10640	no KO assigned | (GenBank) hypothetical protein	Hypothetical	295.961	277.878	259.241	46.029	103.799	96.507
AMK58_10645	no KO assigned | (GenBank) hypothetical protein	Hypothetical	158.305	137.932	143.145	38.481	68.294	76.236
AMK58_10650	no KO assigned | (GenBank) hypothetical protein	Hypothetical	187.640	253.644	194.357	611.532	380.000	763.531
AMK58_10655	no KO assigned | (GenBank) hypothetical protein	Hypothetical	16.537	16.467	33.319	609.186	376.414	276.019
AMK58_10660	no KO assigned | (GenBank) cupin	No COG	10.954	18.945	25.765	1134.143	184.374	602.781
AMK58_10665	no KO assigned | (GenBank) acetyl-CoA synthetase	Oxidoreductase	25.735	24.847	22.003	6.799	22.594	9.682
AMK58_10670	K02049 NitT/TauT family transport system ATP-binding protein | (GenBank) nitrate ABC transporter ATF	Transport: NitT/TauT	49.275	56.074	47.743	233.221	85.102	196.544
AMK58_10675	K02050 NitT/TauT family transport system permease protein | (GenBank) ABC transporter permease	Transport: NitT/TauT	59.724	63.592	56.084	113.273	12.463	129.405
AMK58_10680	K02051 NitT/TauT family transport system substrate-binding protein | (GenBank) ABC transporter subst	Transport: NitT/TauT	184.867	192.329	211.445	901.614	135.162	695.348
AMK58_10685	no KO assigned | (GenBank) lytic murein transglycosylase	enzyme	102.267	88.956	92.021	814.079	70.029	307.016
AMK58_10690	K02836 peptide chain release factor 2 | (GenBank) peptide chain release factor 2	Translation	44.142	39.935	40.412	114.375	44.514	129.461
AMK58_10695	no KO assigned | (GenBank) alginate biosynthesis protein	Biosynthesis: secondary metabolites	126.540	100.466	126.391	150.698	86.570	151.486
AMK58_10700	no KO assigned | (GenBank) hypothetical protein	Hypothetical	79.831	77.853	84.084	173.541	98.266	102.650
AMK58_10705	K05366 penicillin-binding protein 1A [EC:2.4.1.- 3.4.-.-] | (GenBank) penicillin-binding protein	Cell Wall	106.079	114.654	108.380	66.915	95.498	85.980
AMK58_10710	K01448 N-acetylmuramoyl-L-alanine amidase [EC:3.5.1.28] | (GenBank) N-acetylmuramoyl-L-alanine ami	Cell Wall	7.177	2.227	10.989	136.591	16.239	217.107
AMK58_10715	K08300 ribonuclease E [EC:3.1.26.12] | (GenBank) ribonuclease E	Genetic Information Processing	3.166	3.419	6.130	50.485	13.678	55.640

AMK58_10720	K04760 transcription elongation factor GreB | (GenBank) transcription elongation factor GreB	Transcription	837.434	759.267	755.213	621.137	956.754	735.327
AMK58_10725	K01560 2-haloacid dehalogenase [EC:3.8.1.2] | (GenBank) 2-haloalkanoic acid dehalogenase	Metabolism	282.112	294.306	322.784	396.719	127.643	268.934
AMK58_10730	no KO assigned | (GenBank) hypothetical protein	Hypothetical	1833.578	1778.727	1967.986	481.194	1220.698	447.445
AMK58_10735	no KO assigned | (GenBank) peptidase	Peptidase	13.238	8.239	15.734	40.393	54.768	91.470
AMK58_10740	K00681 gamma-glutamyltranspeptidase / glutathione hydrolase [EC:2.3.2.2 3.4.19.13] | (GenBank) gamma	Metabolism: amino acids	30.597	25.233	25.433	111.896	68.680	137.422
AMK58_10745	no KO assigned | (GenBank) 1-aminocyclopropane-1-carboxylate deaminase	No COG	68.976	68.891	64.088	104.333	90.375	107.507
AMK58_10750	K01919 glutamate--cysteine ligase [EC:6.3.2.2] | (GenBank) glutamate--cysteine ligase	Metabolism: Amino Acid	59.429	68.891	65.698	227.060	343.374	210.475
AMK58_10755	K09761 16S rRNA (uracil1498-N3)-methyltransferase [EC:2.1.1.193] | (GenBank) 16S rRNA methyltrans	Ribosome	72.645	53.683	72.112	174.222	86.449	52.957
AMK58_10760	K03179 4-hydroxybenzoate polyprenyltransferase [EC:2.5.1.39] | (GenBank) 4-hydroxybenzoate octapreny	Metabolism: Co-Factors and Vitamens	101.222	99.881	116.485	306.176	163.694	279.039
AMK58_10765	no KO assigned | (GenBank) hypothetical protein	Hypothetical	261.041	225.728	219.080	202.253	223.283	266.873
AMK58_10770	no KO assigned | (GenBank) nicotinamide N-methyase	methyltransferase	60.721	59.184	76.746	282.597	105.988	236.787
AMK58_10775	no KO assigned | (GenBank) DNA alkylation repair protein	DNA Repair and Replication	74.639	66.098	78.376	207.481	128.737	170.066
AMK58_10780	K03182 4-hydroxy-3-polyprenylbenzoate decarboxylase [EC:4.1.1.98] | (GenBank) hypothetical protein	Metabolism: Co-Factors and Vitamens	130.284	121.038	124.864	92.785	29.253	113.532
AMK58_10785	no KO assigned | (GenBank) hypothetical protein	Hypothetical	28.175	35.745	31.236	109.727	27.145	117.717
AMK58_10790	K15977 putative oxidoreductase | (GenBank) hypothetical protein	Oxidoreductase	14.798	24.112	21.745	296.620	46.272	91.562
AMK58_10795	K03592 PmbA protein | (GenBank) modulator protein	Peptidase	57.323	53.345	58.069	209.311	77.380	189.434
AMK58_10800	no KO assigned | (GenBank) hypothetical protein	Hypothetical	7.741	9.878	13.005	167.320	38.015	167.464
AMK58_10805	no KO assigned | (GenBank) lipid kinase	No COG	4.188	3.033	5.430	100.304	27.532	112.858
AMK58_10810	no KO assigned | (GenBank) UDP-2,3-diacylglucosamine hydrolase	hydrolase	413.887	434.355	480.615	298.683	848.732	268.598
AMK58_10815	no KO assigned | (GenBank) alpha-mannosyltransferase	transferase	863.764	1237.452	1089.654	316.092	209.657	436.941
AMK58_10820	no KO assigned | (GenBank) chemotaxis protein	Chemotaxis	6.429	4.801	7.980	163.621	27.399	90.660
AMK58_10825	K02527 3-deoxy-D-manno-octulosonic-acid transferase [EC:2.4.99.12 2.4.99.13 2.4.99.14 2.4.99.15] | (	Lipopolysaccharide biosynthesis	1.980	1.690	2.510	39.157	30.844	154.415
AMK58_10830	K00912 tetraacyldisaccharide 4'-kinase [EC:2.7.1.130] | (GenBank) tetraacyldisaccharide 4'-kinase	Lipopolysaccharide biosynthesis	11.976	12.163	18.105	270.951	52.768	195.001
AMK58_10835	K02517 Kdo2-lipid IVA lauroyltransferase [EC:2.3.1.241] | (GenBank) lauroyl acyltransferase	Lipopolysaccharide biosynthesis	61.137	66.049	47.238	300.820	167.234	257.763
AMK58_10840	no KO assigned | (GenBank) transporter	transport	61.078	58.127	62.185	312.304	90.931	154.705
AMK58_10845	no KO assigned | (GenBank) NADH dehydrogenase	Energy	3.389	9.203	9.081	208.819	29.089	48.850
AMK58_10850	K02067 phospholipid/cholesterol/gamma-HCH transport system substrate-binding protein | (GenBank) out	Transport: phospholipid	21.176	29.768	28.188	220.920	69.727	152.003
AMK58_10855	no KO assigned | (GenBank) hypothetical protein	Hypothetical	100.647	98.233	78.167	473.876	48.165	220.843
AMK58_10860	no KO assigned | (GenBank) hypothetical protein	Hypothetical	1297.770	1461.781	1243.524	107.279	894.529	312.250
AMK58_10865	no KO assigned | (GenBank) carnitine dehydratase	Dehydratase	89.963	113.085	82.331	29.435	73.366	78.680
AMK58_10870	no KO assigned | (GenBank) hypothetical protein	Hypothetical	2.771	6.069	4.127	42.680	6.229	59.946
AMK58_10875	no KO assigned | (GenBank) cytidine deaminase	No COG	5.584	12.132	5.792	47.262	17.404	133.453
AMK58_10880	K09919 uncharacterized protein | (GenBank) hypothetical protein	Hypothetical	57.597	69.064	62.091	209.456	80.357	152.984
AMK58_10885	no KO assigned | (GenBank) hypothetical protein	Hypothetical	191.184	242.664	199.244	536.114	359.135	420.531
AMK58_10890	no KO assigned | (GenBank) flagellin	motility	27.900	28.686	30.908	60.928	26.839	58.241
AMK58_10895	no KO assigned | (GenBank) septum formation initiator	Cell Cycle/Shape/Homeostatsis	92.548	90.203	87.443	179.785	263.619	115.366
AMK58_10900	K07216 hemerythrin | (GenBank) hypothetical protein	hemerythrin	315.847	287.545	317.948	267.959	134.966	230.118
AMK58_10905	K00525 ribonucleoside-diphosphate reductase alpha chain [EC:1.17.4.1] | (GenBank) ribonucleotide-dip	Metabolism: Nucleotide	491.492	626.714	550.467	55.414	23.926	91.053
AMK58_10910	no KO assigned | (GenBank) hypothetical protein	Hypothetical	68.372	73.754	81.486	778.810	411.482	620.565
AMK58_10915	K07216 hemerythrin | (GenBank) hypothetical protein	hemerythrin	60.668	66.891	54.712	226.363	22.243	254.236
AMK58_10920	no KO assigned | (GenBank) hypothetical protein	Hypothetical	1489.315	1229.847	1396.867	496.954	781.798	775.037
AMK58_10925	K01048 lysophospholipase [EC:3.1.1.5] | (GenBank) lysophospholipase	Metabolism: Lipid	1.157	1.536	4.522	180.264	658.940	118.586
AMK58_10930	K03642 rare lipoprotein A | (GenBank) hypothetical protein	No COG	13.978	12.938	10.670	70.412	208.012	227.095
AMK58_10935	K07124 uncharacterized protein | (GenBank) hypothetical protein	Hypothetical	67.956	71.804	72.090	237.515	291.867	200.049
AMK58_10940	K17865 3-hydroxybutyryl-CoA dehydratase [EC:4.2.1.55] | (GenBank) dehydratase	dehydratase	49.775	67.513	57.304	556.478	349.003	171.648
AMK58_10945	K11753 riboflavin kinase / FMN adenylyltransferase [EC:2.7.1.26 2.7.7.2] | (GenBank) bifunctional ri	Metabolism: Co-Factors and Vitamens	9.489	11.338	15.999	49.995	466.158	105.817
AMK58_10950	K00762 orotate phosphoribosyltransferase [EC:2.4.2.10] | (GenBank) orotate phosphoribosyltransferase	Metabolism: Nucleotide	49.056	60.280	67.484	316.216	435.264	91.945
AMK58_10955	K01870 isoleucyl-tRNA synthetase [EC:6.1.1.5] | (GenBank) ileS; isoleucine--tRNA ligase	tRNA synthesis/modification	12.930	21.022	16.123	38.729	47.938	41.197
AMK58_10960	K03101 signal peptidase II [EC:3.4.23.36] | (GenBank) signal peptidase II	Peptidase	57.174	61.715	71.036	403.212	128.802	195.358
AMK58_10965	no KO assigned | (GenBank) hypothetical protein	Hypothetical	228.963	183.823	222.259	177.173	73.886	316.430
AMK58_10970	K07263 zinc protease [EC:3.4.24.-] | (GenBank) zinc protease	Peptidases	288.015	250.887	283.584	329.905	322.097	473.735
AMK58_10975	K07263 zinc protease [EC:3.4.24.-] | (GenBank) zinc protease	Peptidase	51.825	35.601	40.433	42.330	38.057	102.977
AMK58_10980	no KO assigned | (GenBank) hypothetical protein	Hypothetical	928.240	948.663	842.318	633.677	1081.309	504.293
AMK58_10985	K01810 glucose-6-phosphate isomerase [EC:5.3.1.9] | (GenBank) pgi; glucose-6-phosphate isomerase	Metabolism: Carbohydrate	74.236	66.000	64.562	131.998	112.004	114.660
AMK58_10990	no KO assigned | (GenBank) hypothetical protein	Hypothetical	370.940	407.774	348.461	471.850	508.682	468.891
AMK58_10995	no KO assigned | (GenBank) hypothetical protein	Hypothetical	27.814	32.584	35.435	37.015	91.137	100.470
AMK58_11000	no KO assigned | (GenBank) hypothetical protein	Hypothetical	387.066	355.242	376.765	213.170	271.379	440.156
AMK58_11005	K03572 DNA mismatch repair protein MutL | (GenBank) DNA mismatch repair protein MutL	DNA Repair and Replication	8.685	9.156	9.775	33.190	17.227	95.986
AMK58_11010	no KO assigned | (GenBank) pseudogene	Pseudogene	30.225	35.489	44.519	116.783	63.432	205.423
AMK58_11015	no KO assigned | (GenBank) hypothetical protein	Hypothetical	305.922	288.974	347.407	384.211	364.647	556.206
AMK58_11020	no KO assigned | (GenBank) hypothetical protein	Hypothetical	427.196	541.234	595.598	1207.192	2689.530	724.577
AMK58_11025	K14393 cation/acetate symporter | (GenBank) sodium:solute symporter	Transport: Ion	3.432	7.791	7.048	77.507	57.168	118.571
AMK58_11030	K07182 CBS domain-containing protein | (GenBank) hypothetical protein	No COG	194.175	204.562	223.182	20.317	79.607	46.175
AMK58_11035	no KO assigned | (GenBank) hypothetical protein	Hypothetical	261.512	154.706	108.465	49.062	98.071	234.706
AMK58_11040	K01485 cytosine deaminase [EC:3.5.4.1] | (GenBank) amidohydrolase	Metabolism: Nucleotide	465.575	260.825	206.624	139.573	288.544	298.756
AMK58_11045	no KO assigned | (GenBank) hypothetical protein	Hypothetical	156.578	99.711	113.676	499.971	315.504	1073.174
AMK58_11050	no KO assigned | (GenBank) GntR family transcriptional regulator	Transcription	40.530	50.276	45.394	49.461	51.542	208.615

AMK58_11055	K02049 NitT/TauT family transport system ATP-binding protein | (GenBank) nitrate ABC transporter ATP	Transport: NitT/TauT	9.623	11.155	11.607	186.042	100.092	337.874
AMK58_11060	K02050 NitT/TauT family transport system permease protein | (GenBank) ABC transporter permease	Transport: NitT/TauT	5.631	2.804	4.462	44.842	34.990	104.872
AMK58_11065	K02051 NitT/TauT family transport system substrate-binding protein | (GenBank) hypothetical protein	Transport: NitT/TauT	6.999	9.756	7.431	62.656	35.531	166.674
AMK58_11070	no KO assigned | (GenBank) gliding motility-associated lipoprotein GldK	Motility	3.190	6.629	5.275	185.408	27.281	201.005
AMK58_11075	K01130 arylsulfatase [EC:3.1.6.1] | (GenBank) arylsulfatase	Metabolism: Lipid	2.403	1.267	3.091	39.576	21.927	128.130
AMK58_11080	K18292 (S)-citramalyl-CoA lyase [EC:4.1.3.25] | (GenBank) hypothetical protein	Metabolism: Carbohydrate	8.317	10.590	8.686	80.728	40.154	266.640
AMK58_11085	no KO assigned | (GenBank) cytosine deaminase	No COG	4.854	7.961	9.322	117.304	17.440	167.875
AMK58_11090	no KO assigned | (GenBank) alpha/beta hydrolase	hydrolase	42.099	52.952	49.659	324.160	103.715	505.287
AMK58_11095	no KO assigned | (GenBank) hydrolase	hydrolase	9.746	7.381	13.313	220.661	25.151	206.846
AMK58_11100	no KO assigned | (GenBank) GntR family transcriptional regulator	Transcription	50.790	35.759	66.828	201.129	71.314	320.793
AMK58_11105	no KO assigned | (GenBank) NADP-dependent oxidoreductase	Energy	8.260	5.640	11.893	79.038	17.509	76.597
AMK58_11110	no KO assigned | (GenBank) hypothetical protein	Hypothetical	59.276	57.266	70.750	100.353	88.461	355.521
AMK58_11115	no KO assigned | (GenBank) hypothetical protein	Hypothetical	225.696	291.197	239.388	281.122	258.926	450.806
AMK58_11120	no KO assigned | (GenBank) hypothetical protein	Hypothetical	46.000	52.718	47.657	85.647	142.191	128.902
AMK58_11125	no KO assigned | (GenBank) hypothetical protein	Hypothetical	40.757	46.222	41.861	30.343	16.227	85.644
AMK58_11130	K09458 3-oxoacyl-[acyl-carrier-protein] synthase II [EC:2.3.1.179] | (GenBank) 3-oxoacyl-ACP synthas	Metabolism: Lipid	23.344	26.003	18.716	57.242	15.233	77.873
AMK58_11135	K00059 3-oxoacyl-[acyl-carrier protein] reductase [EC:1.1.1.100] | (GenBank) fabG; 3-ketoacyl-ACP re	Biosynthesis: Lipid	53.366	45.832	62.951	526.769	99.539	349.180
AMK58_11140	no KO assigned | (GenBank) hypothetical protein	Hypothetical	40.104	36.395	64.920	782.028	126.963	390.708
AMK58_11145	K00647 3-oxoacyl-[acyl-carrier-protein] synthase I [EC:2.3.1.41] | (GenBank) 3-oxoacyl-ACP synthase	Metabolism: Lipid	19.480	17.308	25.265	189.351	45.926	188.691
AMK58_11150	no KO assigned | (GenBank) hypothetical protein	Hypothetical	18.874	13.196	19.473	79.076	38.142	114.504
AMK58_11155	no KO assigned | (GenBank) hypothetical protein	Hypothetical	2.746	4.643	3.694	32.271	6.056	17.294
AMK58_11160	no KO assigned | (GenBank) hypothetical protein	Hypothetical	14.175	17.643	21.713	361.028	59.748	111.722
AMK58_11165	K07107 acyl-CoA thioester hydrolase [EC:3.1.2.-] | (GenBank) thioesterase	Hydrolase	32.939	23.048	38.091	173.714	44.364	93.283
AMK58_11170	no KO assigned | (GenBank) hypothetical protein	Hypothetical	16.673	14.963	16.873	251.331	19.947	141.531
AMK58_11175	no KO assigned | (GenBank) hypothetical protein	Hypothetical	26.656	23.814	23.924	256.961	18.482	190.581
AMK58_11180	K02078 acyl carrier protein | (GenBank) acyl carrier protein	Biosynthesis: Lipid	42.327	23.524	29.482	76.478	30.981	205.828
AMK58_11185	K02078 acyl carrier protein | (GenBank) acyl carrier protein	Biosynthesis: Lipid	44.069	60.972	53.369	1042.712	102.722	567.370
AMK58_11190	no KO assigned | (GenBank) hypothetical protein	Hypothetical	6.494	5.543	8.821	69.804	12.341	43.159
AMK58_11195	no KO assigned | (GenBank) hypothetical protein	Hypothetical	43.419	44.567	35.281	23.060	25.800	37.778
AMK58_11200	no KO assigned | (GenBank) hypothetical protein	Hypothetical	77.749	54.961	66.871	60.422	52.036	158.498
AMK58_11205	no KO assigned | (GenBank) hypothetical protein	Hypothetical	8.554	2.621	9.385	145.438	42.652	115.727
AMK58_11210	no KO assigned | (GenBank) excinuclease ABC subunit A	No COG	39.773	40.186	41.710	305.959	58.185	160.811
AMK58_11215	no KO assigned | (GenBank) TetR family transcriptional regulator	Transcription	48.864	38.925	42.503	187.399	24.615	150.532
AMK58_11220	K09951 CRISPR-associated protein Cas2 | (GenBank) CRISPR-associated protein Cas2	Defense	134.805	131.745	130.547	569.601	132.352	538.200
AMK58_11225	K15342 CRISP-associated protein Cas1 | (GenBank) type I-C CRISPR-associated endonuclease Cas1	DNA Repair and Replication	163.984	128.181	104.974	37.129	45.495	121.841
AMK58_11230	K07464 CRISPR-associated exonuclease Cas4 [EC:3.1.12.1] | (GenBank) CRISPR-associated protein Cas	Defense	54.873	49.704	52.174	121.165	31.437	324.170
AMK58_11235	K19118 CRISPR-associated protein Csd2 | (GenBank) type I-C CRISPR-associated protein Cas7/Csd2	Defense	3.873	5.225	4.396	13.390	6.882	53.296
AMK58_11240	K19117 CRISPR-associated protein Csd1 | (GenBank) hypothetical protein	Defense	4.170	2.009	5.756	55.594	10.736	74.285
AMK58_11245	K19119 CRISPR-associated protein Cas5d | (GenBank) type I-C CRISPR-associated protein Cas5	Defense	6.840	8.174	6.666	162.873	7.304	49.158
AMK58_11250	K07012 CRISPR-associated endonuclease/helicase Cas3 [EC:3.1.-.- 3.6.4.-] | (GenBank) hypothetical pr	DNA Repair and Replication	14.010	13.430	11.234	65.967	6.816	82.700
AMK58_11255	no KO assigned | (GenBank) serine hydrolase	hydrolase	23.134	28.544	25.941	419.359	59.073	358.260
AMK58_11260	K02030 polar amino acid transport system substrate-binding protein | (GenBank) hypothetical protein	Transport: Amino Acid	32.928	27.239	21.515	150.876	44.985	197.764
AMK58_11265	K02858 3,4-dihydroxy 2-butanone 4-phosphate synthase [EC:4.1.99.12] | (GenBank) ribB; 3,4-dihydroxy-	Metabolism: Co-Factors and Vitamens	51.285	60.016	65.184	705.166	128.169	838.346
AMK58_11270	K05343 maltose alpha-D-glucosyltransferase / alpha-amylase [EC:5.4.99.16 3.2.1.1] | (GenBank) trehal	Metabolism: Carbohydrate	15.412	22.270	15.813	80.373	16.035	161.857
AMK58_11275	no KO assigned | (GenBank) hypothetical protein	Hypothetical	14.414	15.993	18.697	80.128	24.187	160.576
AMK58_11280	K08316 16S rRNA (guanine966-N2)-methyltransferase [EC:2.1.1.171] | (GenBank) 16S rRNA (guanine(	Ribosome	70.470	82.455	87.880	119.889	128.572	148.723
AMK58_11285	K06178 23S rRNA pseudouridine2605 synthase [EC:5.4.99.22] | (GenBank) pseudouridine synthase	Ribosome	9.868	17.096	13.604	184.392	84.296	269.649
AMK58_11290	K09165 uncharacterized protein | (GenBank) dodecin flavoprotein	No COG	123.958	96.447	137.595	1058.070	196.363	1244.055
AMK58_11295	no KO assigned | (GenBank) CMP deaminase	enzyme	17.258	30.140	23.731	96.031	36.411	19.450
AMK58_11300	K07075 uncharacterized protein | (GenBank) hypothetical protein	Hypothetical	329.809	422.606	341.643	449.354	335.599	476.844
AMK58_11305	K01945 phosphoribosylamine--glycine ligase [EC:6.3.4.13] | (GenBank) phosphoribosylamine--glycine li	Metabolism: Nucleotide	1.411	1.124	2.505	49.021	16.496	44.053
AMK58_11310	K03601 exodeoxyribonuclease VII large subunit [EC:3.1.11.6] | (GenBank) exodeoxyribonuclease VII lar	DNA Repair and Replication	3.190	6.205	5.149	64.497	7.172	33.433
AMK58_11315	K08714 voltage-gated sodium channel | (GenBank) hypothetical protein	Transport: Ion	11.788	6.629	13.450	55.362	11.380	62.886
AMK58_11320	K01465 dihydroorotase [EC:3.5.2.3] | (GenBank) dihydroorotase	Metabolism: Nucleotide	88.720	98.360	100.661	245.653	52.175	208.107
AMK58_11325	K01892 histidyl-tRNA synthetase [EC:6.1.1.21] | (GenBank) histidine--tRNA ligase	tRNA synthesis/modification	37.040	41.258	45.877	197.506	53.026	141.626
AMK58_11330	no KO assigned | (GenBank) hypothetical protein	Hypothetical	49.788	34.928	61.086	61.135	28.900	94.199
AMK58_11335	no KO assigned | (GenBank) glycosyl transferase family 51	Glycosyl Transferase	24.719	20.312	28.485	65.635	20.232	53.038
AMK58_11340	no KO assigned | (GenBank) hypothetical protein	Hypothetical	473.975	504.667	462.261	723.550	338.123	1125.743
AMK58_11345	K03594 bacterioferritin [EC:1.16.3.1] | (GenBank) bacterioferritin	Metabolism: Co-Factors and Vitamins	35.789	33.362	48.992	567.141	63.624	324.805
AMK58_11350	no KO assigned | (GenBank) hypothetical protein	Hypothetical	214.162	293.220	248.364	1120.903	103.039	945.924
AMK58_11355	K14228 tRNA Leu | (GenBank) tRNA-Leu	tRNA synthesis/modification	585.195	680.299	816.796	463.388	610.799	851.378
AMK58_11360	no KO assigned | (GenBank) hypothetical protein	Hypothetical	580.765	635.141	613.500	117.354	621.824	528.489
AMK58_11365	no KO assigned | (GenBank) molybdopterin-binding protein	No COG	39.267	48.410	34.373	136.512	140.997	336.665
AMK58_11370	K06206 sugar fermentation stimulation protein A | (GenBank) XRE family transcriptional regulator	transcription	13.619	7.938	11.844	6.229	44.244	57.136
AMK58_11375	K01265 methionyl aminopeptidase [EC:3.4.11.18] | (GenBank) methionine aminopeptidase	Peptidase	195.113	176.069	191.865	66.313	203.650	153.193
AMK58_11380	K03630 DNA repair protein RadC | (GenBank) DNA repair protein RadC	DNA Repair and Replication	74.241	85.471	81.757	193.453	115.273	271.039
AMK58_11385	no KO assigned | (GenBank) Tellurite resistance protein TerB	defense	135.285	120.559	140.791	491.888	263.241	334.701

AMK58_11390	no KO assigned | (GenBank) hypothetical protein	Hypothetical	63.356	50.633	61.233	36.708	77.426	63.976
AMK58_11395	K01756 adenylosuccinate lyase [EC:4.3.2.2] | (GenBank) adenylosuccinate lyase	Metabolism	50.728	44.652	50.809	106.247	89.049	132.281
AMK58_11400	no KO assigned | (GenBank) aldolase	enzyme	95.804	83.377	125.878	300.625	202.656	300.085
AMK58_11405	no KO assigned | (GenBank) hypothetical protein	Hypothetical	181.612	170.638	180.648	654.566	312.142	765.881
AMK58_11410	K01923 phosphoribosylaminoimidazole-succinocarboxamide synthase [EC:6.3.2.6] | (GenBank) phosphori	Metabolism: Nucleotide	354.361	303.526	353.182	272.218	536.723	350.604
AMK58_11415	K01952 phosphoribosylformylglycinamidine synthase [EC:6.3.5.3] | (GenBank) phosphoribosylformylglyc	Metabolism: Nucleotide	276.529	360.189	308.405	227.594	818.799	315.094
AMK58_11420	K01247 DNA-3-methyladenine glycosylase II [EC:3.2.2.21] | (GenBank) DNA glycosylase	DNA Repair and Replication	30.361	32.075	31.860	312.964	325.250	104.545
AMK58_11425	no KO assigned | (GenBank) molybdenum cofactor sulfurase	No COG	429.026	486.430	426.441	212.487	673.563	328.999
AMK58_11430	no KO assigned | (GenBank) transporter	Transport	85.452	99.031	92.604	37.374	93.390	62.149
AMK58_11435	K03566 LysR family transcriptional regulator, glycine cleavage system transcriptional activator | (G	Transcription	58.175	74.997	61.474	167.791	115.459	132.170
AMK58_11440	K01952 phosphoribosylformylglycinamidine synthase [EC:6.3.5.3] | (GenBank) phosphoribosylformylglyc	Metabolism: Nucleotide	40.015	42.067	49.150	254.666	68.722	109.562
AMK58_11445	K01952 phosphoribosylformylglycinamidine synthase [EC:6.3.5.3] | (GenBank) phosphoribosylformylglyc	Metabolism: Nucleotide	6.384	8.623	8.181	111.396	18.657	36.085
AMK58_11450	no KO assigned | (GenBank) ATP-binding protein	Metabolism: Nucleotide	40.358	48.224	63.315	1173.145	201.305	704.492
AMK58_11455	K07390 monothiol glutaredoxin | (GenBank) glutaredoxin	Chaperone	801.855	846.030	756.352	339.547	500.998	436.604
AMK58_11460	no KO assigned | (GenBank) hypothetical protein	Hypothetical	1818.348	1669.538	1695.027	1039.854	818.749	776.596
AMK58_11465	K05786 chloramphenicol-sensitive protein RarD | (GenBank) transporter	Transport	148.631	180.657	151.405	56.512	386.077	92.597
AMK58_11470	no KO assigned | (GenBank) transcriptional regulator	Transcription	941.448	1018.178	889.904	36.860	1636.830	158.538
AMK58_11475	K10680 N-ethylmaleimide reductase [EC:1.-.-.-] | (GenBank) alkene reductase	Metabolism	2.413	4.586	4.797	66.990	152.612	68.440
AMK58_11480	K02986 small subunit ribosomal protein S4 | (GenBank) 30S ribosomal protein S4	Ribosome	515.208	542.223	493.608	183.547	777.833	331.150
AMK58_11485	no KO assigned | (GenBank) nitrogen fixation protein CowN	Nitrogen	138.604	168.052	167.833	508.401	532.917	414.680
AMK58_11490	no KO assigned | (GenBank) hypothetical protein	Hypothetical	104.590	91.693	122.145	1209.922	2236.273	1195.435
AMK58_11495	no KO assigned | (GenBank) response regulator	Signal Transduction	1460.189	1182.291	1305.004	110.395	1979.832	285.549
AMK58_11500	K02533 tRNA/rRNA methyltransferase [EC:2.1.1.-] | (GenBank) rRNA methyltransferase	tRNA synthesis/modification	17.745	22.517	13.139	19.774	32.126	131.491
AMK58_11505	K01649 2-isopropylmalate synthase [EC:2.3.3.13] | (GenBank) transferase	Metabolism	8.147	8.563	6.312	19.301	10.430	44.488
AMK58_11510	no KO assigned | (GenBank) hypothetical protein	Hypothetical	45.622	62.900	70.072	452.083	90.400	164.608
AMK58_11515	no KO assigned | (GenBank) hypothetical protein	Hypothetical	35.249	27.435	36.221	98.161	30.098	97.156
AMK58_11520	no KO assigned | (GenBank) preQ0 transporter	transport	585.065	510.238	607.036	553.873	269.845	899.606
AMK58_11525	K11941 glucans biosynthesis protein C [EC:2.1.-.-] | (GenBank) hypothetical protein	Transferase	87.493	102.128	104.251	88.748	109.034	80.568
AMK58_11530	K01883 cysteinyl-tRNA synthetase [EC:6.1.1.16] | (GenBank) cysteine--tRNA ligase	tRNA synthesis/modification	141.254	147.409	144.946	276.212	126.882	191.202
AMK58_11535	K01885 glutamyl-tRNA synthetase [EC:6.1.1.17] | (GenBank) glutamine--tRNA ligase	tRNA synthesis/modification	431.779	364.907	390.154	74.456	91.944	86.959
AMK58_11540	no KO assigned | (GenBank) indolepyruvate/phenylpyruvate decarboxylase	IAA Biosynthesis	8.944	10.599	14.407	211.594	53.174	106.170
AMK58_11545	no KO assigned | (GenBank) hypothetical protein	Hypothetical	300.563	261.423	299.511	349.980	116.037	392.747
AMK58_11550	K01916 NAD+ synthase [EC:6.3.1.5] | (GenBank) NAD synthetase	Energy	63.919	59.519	66.790	133.450	38.253	141.399
AMK58_11555	no KO assigned | (GenBank) hypothetical protein	Hypothetical	614.480	698.921	839.803	222.760	65.661	144.541
AMK58_11560	no KO assigned | (GenBank) hypothetical protein	Hypothetical	83.448	89.028	115.355	81.509	30.150	127.700
AMK58_11565	no KO assigned | (GenBank) F-box protein SKIP8	No COG	110.220	144.671	141.054	871.702	155.803	648.325
AMK58_11570	K18988 D-alanyl-D-alanine-carboxypeptidase / D-alanyl-D-alanine-endopeptidase [EC:3.4.-.-] | (GenBan	Cell Wall	1.746	2.454	5.566	261.781	147.752	223.968
AMK58_11575	no KO assigned | (GenBank) hypothetical protein	Hypothetical	668.774	903.339	842.437	74.287	96.198	116.782
AMK58_11580	K20534 polyisoprenyl-phosphate glycosyltransferase [EC:2.4.-.-] | (GenBank) glycosyl transferase fam	Glycosyl Transferase	181.241	207.577	230.036	86.982	39.649	58.541
AMK58_11585	no KO assigned | (GenBank) chemotaxis protein	Chemotaxis	2.711	1.799	5.670	112.804	33.245	58.201
AMK58_11590	no KO assigned | (GenBank) hypothetical protein	Hypothetical	107.243	92.825	110.075	347.469	118.842	238.419
AMK58_11595	K01999 branched-chain amino acid transport system substrate-binding protein | (GenBank) ABC transpor	Transport: Amino Acid	52.082	46.488	57.905	99.090	73.236	156.427
AMK58_11600	K01997 branched-chain amino acid transport system permease protein | (GenBank) ABC transporter perme	Transport: Amino Acid	112.474	119.373	115.622	402.540	76.450	305.643
AMK58_11605	K06920 7-cyano-7-deazaguanine synthase [EC:6.3.4.20] | (GenBank) 7-cyano-7-deazaguanine synthase	Metabolism: Co-Factors and Vitamens	51.318	31.179	35.892	130.960	19.358	153.403
AMK58_11610	no KO assigned | (GenBank) hypothetical protein	Hypothetical	158.844	118.425	116.138	139.820	28.905	168.901
AMK58_11615	no KO assigned | (GenBank) hypothetical protein	Hypothetical	71.288	43.696	50.941	161.714	18.720	108.920
AMK58_11620	K01998 branched-chain amino acid transport system permease protein | (GenBank) ABC transporter perme	Transport: Amino Acid	15.550	14.183	16.929	144.695	31.488	167.414
AMK58_11625	K01995 branched-chain amino acid transport system ATP-binding protein | (GenBank) ABC transporter A	Transport: Amino Acid	360.034	336.422	319.724	271.592	118.521	455.735
AMK58_11630	K01996 branched-chain amino acid transport system ATP-binding protein | (GenBank) ABC transporter A	Transport: Amino Acid	64.677	76.251	80.190	401.768	112.054	384.947
AMK58_11635	K01432 arylformamidase [EC:3.5.1.9] | (GenBank) hypothetical protein	Metabolism	42.513	38.613	41.377	317.061	62.316	200.434
AMK58_11640	K14233 tRNA Ser | (GenBank) tRNA-Ser	tRNA synthesis/modification	143.944	176.820	122.794	870.398	244.968	793.372
AMK58_11645	K02888 large subunit ribosomal protein L21 | (GenBank) 50S ribosomal protein L21	Ribosome	38.074	27.296	39.821	453.916	42.369	224.186
AMK58_11650	K02899 large subunit ribosomal protein L27 | (GenBank) 50S ribosomal protein L27	Ribosome	103.716	95.929	110.168	696.411	116.094	373.025
AMK58_11655	K03979 GTPase [EC:3.6.5.-] | (GenBank) obgE; GTPase Obg	Hydrolase	2.374	4.137	4.044	10.733	9.562	18.620
AMK58_11660	K00931 glutamate 5-kinase [EC:2.7.2.11] | (GenBank) glutamate 5-kinase	Metabolism	597.238	636.374	532.438	30.544	1012.456	116.141
AMK58_11665	no KO assigned | (GenBank) haloacid dehalogenase	Dehydrogenase	830.000	1014.178	980.531	29.876	1749.912	219.615
AMK58_11670	no KO assigned | (GenBank) hypothetical protein	Hypothetical	167.079	142.422	164.825	344.820	366.676	472.347
AMK58_11675	no KO assigned | (GenBank) hypothetical protein	Hypothetical	38.007	39.211	42.750	138.304	46.135	51.593
AMK58_11680	K07090 uncharacterized protein | (GenBank) hypothetical protein	Hypothetical	32.547	23.334	35.433	216.902	69.144	128.567
AMK58_11685	no KO assigned | (GenBank) cytochrome C	Energy	18.758	21.225	19.592	89.659	44.725	158.522
AMK58_11690	K00147 glutamate-5-semialdehyde dehydrogenase [EC:1.2.1.41] | (GenBank) proA; gamma-glutamyl phos	Metabolism: Amino Acid	3.686	5.647	5.841	104.959	31.022	311.919
AMK58_11695	K00969 nicotinate-nucleotide adenylyltransferase [EC:2.7.7.18] | (GenBank) nicotinate-nucleotide ade	Metabolism: Co-Factors and Vitamins	18.024	19.898	25.334	270.310	39.859	264.324
AMK58_11700	K09710 ribosome-associated protein | (GenBank) ribosome-associated protein IOJAP	Ribosome	51.798	89.233	127.205	445.692	76.798	448.784
AMK58_11705	K00783 23S rRNA (pseudouridine1915-N3)-methyltransferase [EC:2.1.1.177] | (GenBank) 50S rRNA me	Ribosome	195.461	196.992	226.225	322.605	209.032	305.501
AMK58_11710	K15633 2,3-bisphosphoglycerate-independent phosphoglycerate mutase [EC:5.4.2.12] | (GenBank) 2,3-bis	Metabolism	84.118	104.854	95.344	99.942	229.082	86.174
AMK58_11715	no KO assigned | (GenBank) metallopeptidase	Peptidase	403.491	512.446	490.370	31.895	1100.939	236.303
AMK58_11720	K03797 carboxyl-terminal processing protease [EC:3.4.21.102] | (GenBank) peptidase S41	Peptidase	107.793	182.759	161.802	84.020	540.809	147.389

AMK58_11725	K09798 uncharacterized protein | (GenBank) hypothetical protein	Hypothetical	355.621	346.535	352.494	281.932	794.644	290.346
AMK58_11730	K08311 putative (di)nucleoside polyphosphate hydrolase [EC:3.6.1.-] | (GenBank) RNA pyrophosphohydr	Genetic Information Processing	174.805	167.040	158.346	289.850	352.531	202.897
AMK58_11735	no KO assigned | (GenBank) hypothetical protein	Hypothetical	459.020	474.288	472.423	226.347	302.429	303.087
AMK58_11740	K02114 F-type H+-transporting ATPase subunit epsilon | (GenBank) ATP synthase subunit epsilon	Energy	200.914	223.646	236.355	492.042	235.040	235.387
AMK58_11745	K02112 F-type H+-transporting ATPase subunit beta [EC:3.6.3.14] | (GenBank) ATP synthase subunit bet	Energy	36.911	42.698	40.452	41.222	59.446	61.047
AMK58_11750	K02115 F-type H+-transporting ATPase subunit gamma | (GenBank) ATP synthase F0F1 subunit gamma	Energy	123.113	131.519	130.106	176.202	251.566	245.078
AMK58_11755	K02111 F-type H+-transporting ATPase subunit alpha [EC:3.6.3.14] | (GenBank) ATP synthase subunit al	Energy	317.248	278.468	289.453	53.320	77.119	61.125
AMK58_11760	K02113 F-type H+-transporting ATPase subunit delta | (GenBank) ATP synthase subunit delta	Energy	4631.642	3313.476	3812.860	299.902	949.310	454.988
AMK58_11765	no KO assigned | (GenBank) hypothetical protein	Hypothetical	4912.871	3706.081	3752.380	296.340	979.955	783.462
AMK58_11770	no KO assigned | (GenBank) chemotaxis protein	Chemotaxis	5704.409	4234.726	4768.734	446.971	1148.736	676.072
AMK58_11775	K04066 primosomal protein N' (replication factor Y) (superfamily II helicase) [EC:3.6.4.-] | (GenBan	DNA Repair and Replication	549.221	510.782	440.488	24.622	145.570	65.569
AMK58_11780	K00616 transaldolase [EC:2.2.1.2] | (GenBank) fructose-6-phosphate aldolase	Metabolism	104.305	142.029	115.995	92.078	129.050	143.370
AMK58_11785	K09921 uncharacterized protein | (GenBank) hypothetical protein	Hypothetical	283.525	358.638	294.569	162.640	207.761	227.841
AMK58_11790	K03733 integrase/recombinase XerC | (GenBank) recombinase XerC	DNA Repair and Replication	43.763	44.456	50.005	477.266	121.917	194.601
AMK58_11795	K00382 dihydrolipoamide dehydrogenase [EC:1.8.1.4] | (GenBank) dihydrolipoamide dehydrogenase	Metabolism: Carbon	227.631	209.107	205.258	49.316	220.022	102.974
AMK58_11800	no KO assigned | (GenBank) hypothetical protein	Hypothetical	183.020	186.166	209.356	227.060	254.674	205.567
AMK58_11805	K01561 haloacetate dehalogenase [EC:3.8.1.3] | (GenBank) alpha/beta hydrolase	Metabolism	46.124	50.028	50.772	156.118	82.242	167.004
AMK58_11810	no KO assigned | (GenBank) entericidin	Defense	5001.379	4362.880	4630.822	1928.890	1075.907	1894.462
AMK58_11815	K03272 D-beta-D-heptose 7-phosphate kinase / D-beta-D-heptose 1-phosphate adenosyltransferase [EC:2.	Biosynthesis: polysacchharide	10.550	18.509	13.295	85.692	41.600	126.323
AMK58_11820	no KO assigned | (GenBank) hypothetical protein	Hypothetical	199.610	275.874	230.929	535.749	232.952	442.673
AMK58_11825	no KO assigned | (GenBank) histidine kinase	Signal Transduction	6.507	4.921	14.018	30.782	88.507	53.996
AMK58_11830	no KO assigned | (GenBank) hypothetical protein	Hypothetical	708.576	833.581	869.422	1606.412	1379.078	1069.235
AMK58_11835	K00598 trans-aconitate 2-methyltransferase [EC:2.1.1.144] | (GenBank) trans-aconitate methyltransfer	methyltransferase	69.638	75.645	86.377	142.045	56.394	124.451
AMK58_11840	no KO assigned | (GenBank) hypothetical protein	Hypothetical	27.369	27.715	27.346	494.014	97.247	288.417
AMK58_11845	K03496 chromosome partitioning protein | (GenBank) chromosome partitioning protein ParA	Cell Cycle/Shape/Homeostatsis	142.030	168.087	117.333	92.789	76.116	98.956
AMK58_11850	no KO assigned | (GenBank) hypothetical protein	Hypothetical	57.977	80.973	71.663	119.925	61.022	32.396
AMK58_11855	no KO assigned | (GenBank) ubiquinone biosynthesis methyltransferase UbiE	methyltransferase	16.210	14.026	19.027	109.621	79.042	66.968
AMK58_11860	K00927 phosphoglycerate kinase [EC:2.7.2.3] | (GenBank) pgk; phosphoglycerate kinase	Signal Transduction	36.515	52.575	47.894	120.860	55.938	69.594
AMK58_11865	K06187 recombination protein RecR | (GenBank) recombination protein RecR	DNA Repair and Replication	192.415	179.012	218.416	478.913	416.757	320.554
AMK58_11870	K09747 uncharacterized protein | (GenBank) nucleoid-associated protein	No COG	275.781	216.560	205.367	501.575	570.415	398.835
AMK58_11875	K02343 DNA polymerase III subunit gamma/tau [EC:2.7.7.7] | (GenBank) DNA polymerase III subunit ga	DNA Repair and Replication	162.341	160.100	148.556	109.254	144.918	113.593
AMK58_11880	K08738 cytochrome c | (GenBank) cytochrome C	Energy	226.455	259.872	242.389	230.064	607.379	246.993
AMK58_11885	K00979 3-deoxy-manno-octulosonate cytidylyltransferase (CMP-KDO synthetase) [EC:2.7.7.38] | (GenBa	Biosynthesis: polysacchharide	316.268	386.890	295.895	53.445	653.903	104.586
AMK58_11890	K04518 prephenate dehydratase [EC:4.2.1.51] | (GenBank) prephenate dehydratase	Biosynthesis: Amino Acid	156.782	154.479	167.020	334.634	431.192	245.598
AMK58_11895	K03426 NAD+ diphosphatase [EC:3.6.1.22] | (GenBank) NADH pyrophosphatase	Energy	12.437	10.615	14.239	24.991	83.739	52.840
AMK58_11900	no KO assigned | (GenBank) histidinol phosphate phosphatase	phosphatase	657.020	592.172	498.702	149.758	533.597	112.413
AMK58_11905	no KO assigned | (GenBank) hypothetical protein	Hypothetical	62.372	49.320	61.048	318.228	61.603	162.663
AMK58_11910	K06188 aquaporin Z | (GenBank) porin	Transport	135.290	146.428	135.781	290.446	92.749	237.266
AMK58_11915	K13893 microcin C transport system substrate-binding protein | (GenBank) ABC transporter substrate-b	Membrane transport	59.720	81.675	70.154	131.343	39.924	90.192
AMK58_11920	no KO assigned | (GenBank) hypothetical protein	Hypothetical	41.876	34.769	42.463	133.375	47.213	94.907
AMK58_11925	K13893 microcin C transport system substrate-binding protein | (GenBank) hypothetical protein	Transport	19.698	16.483	21.047	49.593	52.577	51.345
AMK58_11930	K13894 microcin C transport system permease protein | (GenBank) microcin ABC transporter permease	Transport	21.706	13.685	43.247	115.868	53.211	142.714
AMK58_11935	K13895 microcin C transport system permease protein | (GenBank) ABC transporter permease	Transport	133.694	146.881	146.665	320.146	355.823	421.137
AMK58_11940	K13896 microcin C transport system ATP-binding protein | (GenBank) microcin ABC transporter ATP-bir	Membrane transport	35.110	30.913	29.518	41.685	46.528	146.746
AMK58_11945	no KO assigned | (GenBank) hypothetical protein	Hypothetical	425.849	387.453	374.203	506.318	825.945	923.192
AMK58_11950	K06181 23S rRNA pseudouridine2457 synthase [EC:5.4.99.20] | (GenBank) pseudouridine synthase	Metabolism	135.096	142.633	127.009	107.109	250.250	299.913
AMK58_11955	K12972 glyoxylate/hydroxypyruvate reductase A [EC:1.1.1.79 1.1.1.81] | (GenBank) glyoxylate/hydroxyp	Metabolism	83.721	88.956	74.014	237.068	175.292	296.842
AMK58_11960	no KO assigned | (GenBank) MarR family transcriptional regulator	Transcription	143.862	149.264	169.154	600.566	695.896	651.215
AMK58_11965	no KO assigned | (GenBank) hypothetical protein	Hypothetical	105.018	75.187	81.697	120.913	822.413	570.201
AMK58_11970	no KO assigned | (GenBank) hypothetical protein	Hypothetical	83.706	122.048	137.385	786.234	558.934	475.414
AMK58_11975	K02410 flagellar motor switch protein FliG | (GenBank) flagellar motor switch protein FliG	Motility	101.902	91.922	98.239	192.467	432.492	193.152
AMK58_11980	K01255 leucyl aminopeptidase [EC:3.4.11.1] | (GenBank) cytochrome C oxidase subunit II	Energy	28.068	32.288	36.633	99.250	187.434	84.546
AMK58_11985	K00799 glutathione S-transferase [EC:2.5.1.18] | (GenBank) glutathione S-transferase	Metabolism: Amino Acid	181.703	195.295	209.923	54.796	350.394	87.039
AMK58_11990	K07080 uncharacterized protein | (GenBank) immunogenic protein	No COG	242.856	236.901	314.479	13.713	156.593	50.189
AMK58_11995	K03192 urease accessory protein | (GenBank) hypothetical protein	Enzyme	292.383	278.312	259.222	154.134	120.891	435.543
AMK58_12000	no KO assigned | (GenBank) hypothetical protein	Hypothetical	241.918	281.305	235.972	661.482	383.772	339.820
AMK58_12005	K14161 protein ImuB | (GenBank) DNA polymerase	DNA Repair and Replication	22.177	19.664	21.310	135.312	85.884	73.175
AMK58_12010	K14162 error-prone DNA polymerase [EC:2.7.7.7] | (GenBank) DNA polymerase	DNA Repair and Replication	16.643	21.016	21.830	87.958	97.829	61.764
AMK58_12015	no KO assigned | (GenBank) protein tyrosine phosphatase	signal transduction	14.847	19.989	26.046	376.753	281.118	145.039
AMK58_12020	no KO assigned | (GenBank) hypothetical protein	Hypothetical	12.900	12.111	19.800	142.931	158.879	55.879
AMK58_12025	K14160 protein ImuA | (GenBank) hypothetical protein	Hypothetical	1.768	1.760	7.142	241.306	49.503	68.564
AMK58_12030	no KO assigned | (GenBank) hypothetical protein	Hypothetical	12.478	12.090	23.622	435.658	411.944	337.434
AMK58_12035	no KO assigned | (GenBank) hypothetical protein	Hypothetical	92.738	75.243	103.961	339.325	1755.055	276.029
AMK58_12040	no KO assigned | (GenBank) coproporphyrinogen III oxidase	enzyme	31.876	35.208	27.252	58.846	33.823	78.986
AMK58_12045	K11209 GSH-dependent disulfide-bond oxidoreductase [EC:1.8.4.-] | (GenBank) glutathione S-transferas	Oxidoreductase	52.836	93.186	55.984	257.251	200.085	102.146
AMK58_12050	no KO assigned | (GenBank) hypothetical protein	Hypothetical	402.020	362.456	393.137	365.877	2832.202	283.451
AMK58_12055	K02034 peptide/nickel transport system permease protein | (GenBank) NAD synthetase	Energy	2.488	4.954	6.702	119.233	86.223	75.231

AMK58_12060	K02033 peptide/nickel transport system permease protein | (GenBank) diguanylate cyclase	Quorum sensing	64.548	79.374	79.249	303.049	142.433	238.137
AMK58_12065	K02035 peptide/nickel transport system substrate-binding protein | (GenBank) peptide ABC transporter	Quorum sensing	149.448	174.261	178.410	66.054	86.752	91.784
AMK58_12070	no KO assigned | (GenBank) enoyl-CoA hydratase	hydratase	71.093	85.319	81.174	80.469	58.450	64.627
AMK58_12075	no KO assigned | (GenBank) MFS transporter	Transport: MFS	19.843	23.610	24.703	81.093	37.520	61.748
AMK58_12080	no KO assigned | (GenBank) hypothetical protein	Hypothetical	160.941	202.892	184.377	894.659	407.173	682.746
AMK58_12085	no KO assigned | (GenBank) LysR family transcriptional regulator	Transcription	216.195	220.822	189.539	208.239	385.063	415.310
AMK58_12090	no KO assigned | (GenBank) hypothetical protein	Hypothetical	62.204	79.635	59.496	386.369	216.417	256.813
AMK58_12095	no KO assigned | (GenBank) universal stress protein UspA	Stress Response	91.527	115.392	109.363	305.257	341.853	267.175
AMK58_12100	no KO assigned | (GenBank) hypothetical protein	Hypothetical	2.561	3.309	4.015	22.626	21.943	23.879
AMK58_12105	K00799 glutathione S-transferase [EC:2.5.1.18] | (GenBank) glutathione S-transferase	Metabolism: amino acids	836.292	933.766	884.570	427.650	341.517	531.692
AMK58_12110	K16871 4-aminobutyrate---pyruvate transaminase [EC:2.6.1.96] | (GenBank) aminotransferase	Metabolism: Amino Acid	21.259	30.463	26.212	63.323	171.258	85.585
AMK58_12115	no KO assigned | (GenBank) hypothetical protein	Hypothetical	1386.939	1700.425	1461.037	422.409	495.623	346.304
AMK58_12120	K00643 5-aminolevulinate synthase [EC:2.3.1.37] | (GenBank) 5-aminolevulinate synthase	Metabolism: Amino Acid	175.882	157.174	162.209	235.336	129.373	267.599
AMK58_12125	no KO assigned | (GenBank) hypothetical protein	Hypothetical	43.684	27.821	48.282	231.991	63.061	266.692
AMK58_12130	no KO assigned | (GenBank) hypothetical protein	Hypothetical	724.267	678.670	757.385	321.241	193.543	407.176
AMK58_12135	no KO assigned | (GenBank) oxidoreductase	Oxidoreductase	19.359	26.464	20.278	114.277	20.654	75.759
AMK58_12140	no KO assigned | (GenBank) signal protein PDZ	No COG	187.625	213.110	176.748	172.971	167.138	175.398
AMK58_12145	no KO assigned | (GenBank) NUDIX hydrolase	hydrolase	1426.199	2098.349	1868.862	115.332	2427.810	314.355
AMK58_12150	K09760 DNA recombination protein RmuC | (GenBank) DNA polymerase V	DNA Repair and Replication	295.304	349.426	312.833	44.832	961.412	148.096
AMK58_12155	K03704 cold shock protein (beta-ribbon, CspA family) | (GenBank) cold-shock protein	Stress Response	773.149	957.585	939.590	560.700	1255.946	1069.770
AMK58_12160	no KO assigned | (GenBank) hypothetical protein	Hypothetical	149.378	200.410	172.928	619.957	925.552	353.820
AMK58_12165	K01462 peptide deformylase [EC:3.5.1.88] | (GenBank) peptide deformylase	Hydrolase	147.105	174.062	171.203	193.535	631.880	293.185
AMK58_12170	K00604 methionyl-tRNA formyltransferase [EC:2.1.2.9] | (GenBank) methionyl-tRNA formyltransferase	tRNA synthesis/modification	33.111	35.832	53.580	455.928	226.962	315.259
AMK58_12175	K06173 tRNA pseudouridine38-40 synthase [EC:5.4.99.12] | (GenBank) pseudouridine synthase	tRNA synthesis/modification	1190.569	1116.398	1098.778	113.838	2943.154	207.653
AMK58_12180	K01997 branched-chain amino acid transport system permease protein | (GenBank) branched-chain amino	Transport: Amino Acid	18.907	20.093	23.162	46.513	28.915	68.104
AMK58_12185	no KO assigned | (GenBank) hypothetical protein	Hypothetical	400.288	382.181	364.935	87.529	565.975	262.217
AMK58_12190	no KO assigned | (GenBank) drug:proton antiporter	Transport	63.100	63.150	62.432	387.444	190.458	100.686
AMK58_12195	no KO assigned | (GenBank) DNA-binding protein	No COG	14.389	14.677	18.019	162.421	50.887	95.836
AMK58_12200	K01703 3-isopropylmalate/(R)-2-methylmalate dehydratase large subunit [EC:4.2.1.33 4.2.1.35] | (GenB	Metabolism	36.219	34.519	42.309	175.355	55.058	120.106
AMK58_12205	no KO assigned | (GenBank) hypothetical protein	Hypothetical	13.453	32.880	28.489	444.782	87.292	293.716
AMK58_12210	no KO assigned | (GenBank) hypothetical protein	Hypothetical	1.302	2.333	1.609	103.590	10.829	45.029
AMK58_12215	K02884 large subunit ribosomal protein L19 | (GenBank) 50S ribosomal protein L19	Ribosome	5.198	1.827	9.593	324.372	38.835	201.008
AMK58_12220	K00554 tRNA (guanine37-N1)-methyltransferase [EC:2.1.1.228] | (GenBank) tRNA (guanine-N1)-methyl	tRNA synthesis/modification	220.232	153.521	195.745	466.917	98.884	292.238
AMK58_12225	K02860 16S rRNA processing protein RimM | (GenBank) rimM; 16S rRNA-processing protein RimM	Ribosome	24.175	17.905	19.756	77.346	20.436	113.567
AMK58_12230	K02959 small subunit ribosomal protein S16 | (GenBank) 30S ribosomal protein S16	Ribosome	148.094	145.080	148.289	626.294	126.482	330.783
AMK58_12235	K03106 signal recognition particle subunit SRP54 [EC:3.6.5.4] | (GenBank) RNA-binding protein	Secretion system	367.915	401.335	411.416	37.345	653.208	103.242
AMK58_12240	no KO assigned | (GenBank) hypothetical protein	Hypothetical	321.979	369.362	328.490	109.431	958.136	262.045
AMK58_12245	no KO assigned | (GenBank) hypothetical protein	Hypothetical	443.941	558.106	481.316	100.667	1120.227	1392.563
AMK58_12250	no KO assigned | (GenBank) hypothetical protein	Hypothetical	372.107	368.023	367.206	125.972	564.342	240.296
AMK58_12255	no KO assigned | (GenBank) hypothetical protein	Hypothetical	96.860	123.539	111.670	190.978	881.089	234.715
AMK58_12260	no KO assigned | (GenBank) diguanylate cyclase	cyclic nucleotide	202.363	210.819	220.594	169.554	106.707	168.769
AMK58_12265	K07025 putative hydrolase of the HAD superfamily | (GenBank) HAD family hydrolase	hydrolase	77.667	111.450	110.344	133.833	121.980	102.623
AMK58_12270	K00674 2,3,4,5-tetrahydropyridine-2-carboxylate N-succinyltransferase [EC:2.3.1.117] | (GenBank) 2,3	Metabolism: Amino Acid	27.719	30.033	30.043	171.998	56.181	65.391
AMK58_12275	K01439 succinyl-diaminopimelate desuccinylase [EC:3.5.1.18] | (GenBank) succinyl-diaminopimelate des	Metabolism: Amino Acid	73.034	92.408	103.919	185.512	43.752	141.239
AMK58_12280	no KO assigned | (GenBank) hypothetical protein	Hypothetical	580.734	684.015	770.287	499.931	178.354	384.474
AMK58_12285	K00768 nicotinate-nucleotide--dimethylbenzimidazole phosphoribosyltransferase [EC:2.4.2.21] | (GenBa	Metabolism: Co-Factors and Vitamens	111.426	130.049	114.671	98.194	164.264	172.387
AMK58_12290	K02233 adenosylcobinamide-GDP ribazoletransferase [EC:2.7.8.26] | (GenBank) cobalamin synthase	Metabolism: Co-Factors and Vitamins	324.418	273.577	285.288	258.458	242.874	247.281
AMK58_12295	K02226 alpha-ribazole phosphatase [EC:3.1.3.73] | (GenBank) phosphoglycerate mutase	Metabolism	190.205	175.359	168.547	535.446	185.799	341.106
AMK58_12300	no KO assigned | (GenBank) pyridoxamine 5'-phosphate oxidase	No COG	64.098	59.570	51.916	127.841	259.911	198.123
AMK58_12305	K02536 UDP-3-O-[3-hydroxymyristoyl] glucosamine N-acyltransferase [EC:2.3.1.191] | (GenBank) UDP	Lipopolysaccharide biosynthesis	39.070	46.813	43.782	138.893	91.332	87.984
AMK58_12310	K01992 ABC-2 type transport system permease protein | (GenBank) ABC transporter	Membrane Transport	2.170	3.457	3.576	100.761	16.585	36.216
AMK58_12315	K01990 ABC-2 type transport system ATP-binding protein | (GenBank) ABC transporter	Membrane Transport	2.721	4.515	3.162	110.149	18.520	33.499
AMK58_12320	K15011 two-component system, sensor histidine kinase RegB [EC:2.7.13.3] | (GenBank) histidine kinase	Signal Transduction	21.997	22.809	30.768	42.008	697.529	135.617
AMK58_12325	K15012 two-component system, response regulator RegA | (GenBank) two-component system response re	Signal Transduction	179.232	200.314	216.069	387.583	512.269	386.707
AMK58_12330	no KO assigned | (GenBank) hypothetical protein	Hypothetical	422.501	478.470	515.637	717.166	1009.358	808.692
AMK58_12335	K01420 CRP/FNR family transcriptional regulator, anaerobic regulatory protein | (GenBank) Crp/Fnr fa	Transcription	118.978	135.694	146.847	274.756	344.455	233.126
AMK58_12340	K16554 polysaccharide biosynthesis transport protein | (GenBank) protein tyrosine kinase	Biosynthesis: polysacchharide	15.813	8.589	14.327	75.316	16.578	77.315
AMK58_12345	no KO assigned | (GenBank) polysaccharide biosynthesis protein	Biosynthesis: polysacchharide	123.252	127.563	147.203	59.334	59.670	57.299
AMK58_12350	K02472 UDP-N-acetyl-D-mannosaminuronic acid dehydrogenase [EC:1.1.1.336] | (GenBank) UDP-gluco	Metabolism: Carbohydrates	30.715	53.878	49.664	40.666	35.629	51.250
AMK58_12355	K01784 UDP-glucose 4-epimerase [EC:5.1.3.2] | (GenBank) NAD-dependent dehydratase	Energy	23.997	33.308	34.570	154.393	58.170	206.221
AMK58_12360	K02377 GDP-L-fucose synthase [EC:1.1.1.271] | (GenBank) GDP-fucose synthetase	Metabolism: Carbohydrate	48.105	44.043	99.437	406.887	172.188	481.983
AMK58_12365	K03208 colanic acid biosynthesis glycosyl transferase WcaI | (GenBank) glycosyl transferase	glycosyl transferase	3.712	2.843	11.584	252.969	34.986	175.289
AMK58_12370	no KO assigned | (GenBank) glycosyl transferase family 1	Glycosyl Transferase	14.885	9.549	31.724	250.639	129.805	191.655
AMK58_12375	no KO assigned | (GenBank) glycosyl transferase	Glycosyl Transferase	7.587	8.184	18.134	178.983	76.634	103.964
AMK58_12380	K06134 ubiquinone biosynthesis monooxygenase Coq7 [EC:1.14.13.-] | (GenBank) hypothetical protein	Metabolism: Co-Factors and Vitamens	9.424	9.792	22.400	250.551	141.620	269.941
AMK58_12385	no KO assigned | (GenBank) hypothetical protein	Hypothetical	1.488	1.111	4.185	125.065	22.856	47.886
AMK58_12390	no KO assigned | (GenBank) hypothetical protein	Hypothetical	4.937	6.726	11.693	150.817	47.895	115.080

AMK58_12395	no KO assigned | (GenBank) hypothetical protein	Hypothetical	3.622	4.946	8.757	204.742	49.203	120.042
AMK58_12400	K16649 rhamnopyranosyl-N-acetylglucosaminyl-diphospho-decaprenol beta-1,3/1,4-galactofuranosyltrans	Biosynthesis: polysaccharide	0.843	3.599	2.635	123.616	21.667	24.018
AMK58_12405	no KO assigned | (GenBank) hypothetical protein	Hypothetical	12.841	20.354	30.898	312.260	91.318	187.845
AMK58_12410	no KO assigned | (GenBank) hypothetical protein	Hypothetical	8.619	6.738	19.225	149.237	88.737	184.427
AMK58_12415	K02914 large subunit ribosomal protein L34 | (GenBank) rpmH; 50S ribosomal protein L34	Ribosome	10.762	10.716	34.962	1768.427	306.462	540.558
AMK58_12420	K03536 ribonuclease P protein component [EC:3.1.26.5] | (GenBank) ribonuclease P protein component	post translation modificaton	4.261	7.488	19.067	417.049	154.408	238.718
AMK58_12425	K03217 YidC/Oxa1 family membrane protein insertase | (GenBank) hypothetical protein	Membrane	1.600	3.741	7.807	120.894	47.085	96.462
AMK58_12430	K03978 GTP-binding protein | (GenBank) GTP-binding protein	Cell Cycle/Shape/Homeostatsis	29.584	42.550	65.113	616.698	542.320	276.017
AMK58_12435	no KO assigned | (GenBank) cation:proton antiporter	Transport: ion	26.428	40.471	36.922	11.758	24.988	12.651
AMK58_12440	no KO assigned | (GenBank) hypothetical protein	Hypothetical	533.517	817.451	699.605	354.699	797.196	523.012
AMK58_12445	K00930 acetylglutamate kinase [EC:2.7.2.8] | (GenBank) acetylglutamate kinase	Biosynthesis: Amino Acid	944.921	726.570	736.633	338.067	1437.598	325.707
AMK58_12450	no KO assigned | (GenBank) hypothetical protein	Hypothetical	141.254	111.460	133.219	376.904	386.025	435.310
AMK58_12455	K03284 magnesium transporter | (GenBank) magnesium transporter	Transport: Inorganic	37.528	30.875	36.384	278.716	235.259	199.210
AMK58_12460	no KO assigned | (GenBank) hypothetical protein	Hypothetical	6.031	16.629	12.350	207.548	463.551	193.138
AMK58_12465	no KO assigned | (GenBank) hypothetical protein	Hypothetical	151.399	142.540	140.136	335.722	124.063	347.147
AMK58_12470	K01962 acetyl-CoA carboxylase carboxyl transferase subunit alpha [EC:6.4.1.2] | (GenBank) acetyl-CoA	Metabolism	8.983	18.141	10.225	32.369	11.661	20.882
AMK58_12475	no KO assigned | (GenBank) hypothetical protein	Hypothetical	31.096	41.109	31.327	254.180	114.080	107.239
AMK58_12480	K01638 malate synthase [EC:2.3.3.9] | (GenBank) malate synthase	Metabolism	12.221	8.112	9.898	7.781	25.438	30.778
AMK58_12485	K04763 integrase/recombinase XerD | (GenBank) recombinase XerD	Genetic Information Processing	14.933	11.252	14.070	78.029	48.086	84.576
AMK58_12490	no KO assigned | (GenBank) hypothetical protein	Hypothetical	134.587	116.329	120.953	86.547	84.959	92.519
AMK58_12495	K00891 shikimate kinase [EC:2.7.1.71] | (GenBank) shikimate kinase	Biosynthesis: Amino Acid	23.987	26.159	32.762	217.013	169.689	249.959
AMK58_12500	K01735 3-dehydroquinate synthase [EC:4.2.3.4] | (GenBank) 3-dehydroquinate synthase	Biosynthesis: Amino Acid	650.579	600.593	623.185	268.616	352.610	346.873
AMK58_12505	no KO assigned | (GenBank) histidine kinase	Signal Transduction	27.653	26.791	32.925	340.056	87.929	274.668
AMK58_12510	no KO assigned | (GenBank) hypothetical protein	Hypothetical	42.721	57.633	53.722	407.970	119.496	100.039
AMK58_12515	no KO assigned | (GenBank) hypothetical protein	Hypothetical	88.099	72.042	72.300	47.139	66.110	101.317
AMK58_12520	K05527 BolA protein | (GenBank) transcriptional regulator	Transcription	136.154	151.030	192.708	825.782	161.019	459.771
AMK58_12525	no KO assigned | (GenBank) molecular chaperone DnaJ	Chaperone	193.513	144.671	214.102	479.349	167.499	231.832
AMK58_12530	K09882 cobaltochelatase CobS [EC:6.6.1.2] | (GenBank) cobalamin biosynthesis protein CobS	Metabolism: Co-Factors and Vitamins	981.368	1209.911	1112.244	128.296	78.000	87.582
AMK58_12535	no KO assigned | (GenBank) GxxExxY protein	DNA Repair and Replication	3956.699	3947.129	3959.064	254.134	1428.086	439.649
AMK58_12540	K09883 cobaltochelatase CobT [EC:6.6.1.2] | (GenBank) cobalt chelatase	Metabolism: Co-Factors and Vitamens	64.950	98.463	101.978	45.110	250.161	60.467
AMK58_12545	no KO assigned | (GenBank) hypothetical protein	Hypothetical	63.252	76.143	67.097	102.117	66.057	140.939
AMK58_12550	K02012 iron(III) transport system substrate-binding protein | (GenBank) iron deficiency-induced prot	Transport: Metal	38.593	41.475	41.841	178.420	163.867	201.191
AMK58_12555	K06911 uncharacterized protein | (GenBank) quercetin 2,3-dioxygenase	No COG	1238.984	1121.244	1089.359	88.066	657.864	259.847
AMK58_12560	K02011 iron(III) transport system permease protein | (GenBank) iron ABC transporter permease	No COG	128.836	97.596	106.123	27.031	106.349	78.753
AMK58_12565	K00761 uracil phosphoribosyltransferase [EC:2.4.2.9] | (GenBank) Uracil phosphoribosyltransferase	Metabolism: Nucleotides	368.804	352.233	319.716	363.486	445.282	452.528
AMK58_12570	K13069 diguanylate cyclase [EC:2.7.7.65] | (GenBank) diguanylate cyclase	cyclic nucleotide	213.568	186.570	177.019	107.947	31.383	163.312
AMK58_12575	K01817 phosphoribosylanthranilate isomerase [EC:5.3.1.24] | (GenBank) N-(5'-phosphoribosyl)anthranil	Biosynthesis: Amino Acid	6422.124	5811.066	5150.039	291.540	312.372	402.428
AMK58_12580	K01591 orotidine-5'-phosphate decarboxylase [EC:4.1.1.23] | (GenBank) orotidine 5'-phosphate decarbo	Metabolism: Nucleotides	100.387	80.035	112.056	185.355	177.264	192.462
AMK58_12585	no KO assigned | (GenBank) hypothetical protein	Hypothetical	1719.699	1918.258	2028.475	448.475	411.632	519.316
AMK58_12590	no KO assigned | (GenBank) hypothetical protein	Hypothetical	252.919	243.068	232.410	118.470	122.765	127.199
AMK58_12595	K01750 ornithine cyclodeaminase [EC:4.3.1.12] | (GenBank) ornithine cyclodeaminase	Metabolism: Amino Acid	103.616	121.693	120.412	274.547	217.152	187.703
AMK58_12600	no KO assigned | (GenBank) hypothetical protein	Hypothetical	138.770	126.124	152.311	476.993	291.379	217.918
AMK58_12605	K05788 integration host factor subunit beta | (GenBank) integration host factor subunit beta	DNA Repair and Replication	217.085	200.509	218.524	751.746	980.826	367.929
AMK58_12610	no KO assigned | (GenBank) hypothetical protein	Hypothetical	7.502	4.482	8.702	100.688	591.930	94.462
AMK58_12615	K02945 small subunit ribosomal protein S1 | (GenBank) 30S ribosomal protein S1	Ribosome	14.444	14.383	18.648	153.270	58.098	57.251
AMK58_12620	K00945 CMP/dCMP kinase [EC:2.7.4.25] | (GenBank) cytidylate kinase	Metabolism: Nucleotide	266.438	259.666	251.421	274.958	238.933	233.345
AMK58_12625	K00800 3-phosphoshikimate 1-carboxyvinyltransferase [EC:2.5.1.19] | (GenBank) 3-phosphoshikimate 1-	Metabolism: Amino Acid	183.874	134.075	146.179	100.972	52.179	39.269
AMK58_12630	K00681 gamma-glutamyltranspeptidase / glutathione hydrolase [EC:2.3.2.2 3.4.19.13] | (GenBank) gamma	Metabolism: amino acids	114.728	100.157	111.970	76.149	28.614	61.924
AMK58_12635	no KO assigned | (GenBank) hypothetical protein	Hypothetical	201.483	224.553	282.671	451.067	445.333	334.238
AMK58_12640	K14218 tRNA Ala | (GenBank) tRNA-Ala	tRNA synthesis/modification	41729.381	35425.387	41059.593	1841.663	92093.907	7762.857
AMK58_12645	no KO assigned | (GenBank) hypothetical protein	Hypothetical	885.278	964.474	864.424	188.926	2446.263	373.427
AMK58_12650	K06911 uncharacterized protein | (GenBank) pirin	No COG	146.952	131.374	145.078	205.651	110.020	144.098
AMK58_12655	K03750 molybdopterin molybdotransferase [EC:2.10.1.1] K07219 putative molybdopterin biosynthesis pro	Metabolism: Co-Factors and Vitamins	36.518	40.429	38.721	210.368	87.028	104.016
AMK58_12660	K03750 molybdopterin molybdotransferase [EC:2.10.1.1] | (GenBank) molybdopterin biosynthesis protein	Metabolism: Co-Factors and Vitamins	208.995	238.171	158.183	22.999	110.250	82.166
AMK58_12665	no KO assigned | (GenBank) diguanylate cyclase	cyclic nucleotide	40.753	34.558	41.123	46.138	26.894	83.379
AMK58_12670	no KO assigned | (GenBank) histidine kinase	Signal Transduction	4.828	4.902	4.920	10.736	5.604	11.031
AMK58_12675	no KO assigned | (GenBank) hypothetical protein	Hypothetical	55.464	55.366	69.653	60.765	123.385	55.770
AMK58_12680	K20534 polyisoprenyl-phosphate glycosyltransferase [EC:2.4.-.-] | (GenBank) glycosyltransferase	Biosynthesis: polysacchharide	102.848	129.634	124.918	368.697	187.799	167.197
AMK58_12685	no KO assigned | (GenBank) hypothetical protein	Hypothetical	38.085	39.054	57.289	461.811	109.897	125.183
AMK58_12690	no KO assigned | (GenBank) heat-shock protein Hsp20	Stress Response	117.790	141.587	118.465	273.139	126.862	356.609
AMK58_12695	no KO assigned | (GenBank) hypothetical protein	Hypothetical	1138.942	1265.089	1229.430	3047.831	1355.630	2669.685
AMK58_12700	no KO assigned | (GenBank) hypothetical protein	Hypothetical	7.057	13.835	15.307	517.134	30.863	332.617
AMK58_12705	no KO assigned | (GenBank) hypothetical protein	Hypothetical	0.000	0.000	0.834	135.938	4.931	152.405
AMK58_12710	no KO assigned | (GenBank) transcriptional regulator	Transcription	6.939	3.384	16.292	261.584	151.215	238.064
AMK58_12715	no KO assigned | (GenBank) hypothetical protein	Hypothetical	0.887	1.136	1.024	59.748	16.664	38.363
AMK58_12720	K01681 aconitate hydratase [EC:4.2.1.3] | (GenBank) aconitate hydratase	Metabolism: Carbohydrate	83.731	80.373	78.585	57.979	714.148	42.263
AMK58_12725	K01999 branched-chain amino acid transport system substrate-binding protein | (GenBank) ABC transpor	Transport: Amino Acid	39.994	38.100	38.373	52.720	428.760	62.175

AMK58_12730	no KO assigned | (GenBank) hypothetical protein	Hypothetical	271.231	260.975	229.448	129.272	101.670	270.108
AMK58_12735	no KO assigned | (GenBank) DNA mismatch repair protein MutT	DNA Repair and Replication	24.584	24.479	25.127	214.601	70.234	162.231
AMK58_12740	K02342 DNA polymerase III subunit epsilon [EC:2.7.7.7] | (GenBank) exonuclease	DNA Repair and Replication	17.742	19.802	20.206	334.035	75.169	122.213
AMK58_12745	K08963 methylthioribose-1-phosphate isomerase [EC:5.3.1.23] | (GenBank) methylthioribose-1-phosphate	Metabolism: Amino Acid	51.465	42.048	64.303	345.289	247.588	451.016
AMK58_12750	no KO assigned | (GenBank) histidine kinase	Signal Transduction	439.457	408.346	455.662	272.829	998.505	334.730
AMK58_12755	K02342 DNA polymerase III subunit epsilon [EC:2.7.7.7] | (GenBank) hypothetical protein	DNA Repair and Replication	104.633	136.932	119.856	683.116	131.333	405.418
AMK58_12760	K07182 CBS domain-containing protein | (GenBank) hypothetical protein	No COG	62.522	56.129	59.572	81.979	94.001	91.411
AMK58_12765	K14393 cation/acetate symporter | (GenBank) actP; cation/acetate symporter ActP	Transport: Ion	12.016	11.824	11.089	19.504	26.853	60.886
AMK58_12770	no KO assigned | (GenBank) hypothetical protein	Hypothetical	46.352	57.295	64.208	468.894	121.720	344.590
AMK58_12775	no KO assigned | (GenBank) hypothetical protein	Hypothetical	84.385	83.569	92.662	344.952	142.279	276.209
AMK58_12780	no KO assigned | (GenBank) SAM-dependent methyltransferase	methyltransferase	51.492	57.013	61.435	659.662	135.199	396.170
AMK58_12785	no KO assigned | (GenBank) hypothetical protein	Hypothetical	51.232	67.463	56.864	147.900	38.313	161.715
AMK58_12790	no KO assigned | (GenBank) amidohydrolase	hydrolase	201.502	229.019	219.923	408.274	105.037	478.320
AMK58_12795	K03676 glutaredoxin 3 | (GenBank) glutaredoxin	Chaperone	52428.134	47015.371	51554.534	1209.636	2540.922	1272.390
AMK58_12800	no KO assigned | (GenBank) competence protein ComF	Secretion System	5095.068	4392.635	4218.088	57.332	213.652	149.693
AMK58_12805	K02420 flagellar biosynthetic protein FliQ | (GenBank) flagellar biosynthesis protein FliQ	Motility	118.807	143.587	104.771	194.988	134.564	336.389
AMK58_12810	no KO assigned | (GenBank) SAM-dependent methyltransferase	methyltransferase	28.750	35.482	44.405	223.299	56.592	107.639
AMK58_12815	K09792 uncharacterized protein | (GenBank) hypothetical protein	Hypothetical	14.661	11.302	19.037	210.757	105.437	136.536
AMK58_12820	no KO assigned | (GenBank) hypothetical protein	Hypothetical	127.668	157.563	149.503	309.920	442.640	239.175
AMK58_12825	no KO assigned | (GenBank) hypothetical protein	Hypothetical	86.298	91.061	72.051	81.381	162.395	105.303
AMK58_12830	K02227 adenosylcobinamide-phosphate synthase [EC:6.3.1.10] | (GenBank) cobalamin biosynthesis protei	Metabolism: Co-Factors and Vitamens	8.528	17.223	16.103	156.072	28.218	42.435
AMK58_12835	no KO assigned | (GenBank) RNA helicase	No COG	24.250	20.179	16.634	31.439	25.895	51.879
AMK58_12840	K03282 large conductance mechanosensitive channel | (GenBank) mechanosensitive ion channel protein M	Transport: Ion	129.201	140.787	169.974	363.558	490.788	210.223
AMK58_12845	K03070 preprotein translocase subunit SecA | (GenBank) preprotein translocase subunit SecA	Secretion system	7.042	8.940	9.415	69.626	71.089	19.507
AMK58_12850	K03769 peptidyl-prolyl cis-trans isomerase C [EC:5.2.1.8] | (GenBank) peptidylprolyl isomerase	Chaperone	2.937	3.815	3.764	79.553	193.804	77.097
AMK58_12855	K00620 glutamate N-acetyltransferase / amino-acid N-acetyltransferase [EC:2.3.1.35 2.3.1.1] | (GenBa	Metabolism: Amino Acid	69.088	74.145	80.648	94.118	100.175	85.811
AMK58_12860	no KO assigned | (GenBank) hypothetical protein	Hypothetical	10.031	12.544	16.192	128.749	29.893	49.321
AMK58_12865	K03574 8-oxo-dGTP diphosphatase [EC:3.6.1.55] | (GenBank) NTP pyrophosphohydrolase	DNA Repair and Replication	575.378	509.933	747.236	839.421	635.102	439.470
AMK58_12870	K03654 ATP-dependent DNA helicase RecQ [EC:3.6.4.12] | (GenBank) ATP-dependent DNA helicase Re	DNA Repair and Replication	9.164	6.375	8.594	23.962	25.963	69.143
AMK58_12875	K14234 tRNA Thr | (GenBank) tRNA-Thr	tRNA synthesis/modification	8836.845	7918.332	7801.994	3827.587	17699.374	6405.359
AMK58_12880	no KO assigned | (GenBank) hypothetical protein	Hypothetical	233.940	175.567	195.513	72.151	144.286	95.621
AMK58_12885	no KO assigned | (GenBank) hypothetical protein	Hypothetical	323.431	264.725	294.908	599.218	283.290	410.870
AMK58_12890	no KO assigned | (GenBank) multidrug DMT transporter permease	Defense	19.474	21.077	20.931	137.990	25.855	27.348
AMK58_12895	no KO assigned | (GenBank) C4-dicarboxylate ABC transporter	Membrane Transport	13.201	11.831	13.986	93.851	14.733	54.736
AMK58_12900	no KO assigned | (GenBank) C4-dicarboxylate ABC transporter permease	Membrane Transport	149.800	185.803	185.545	757.185	509.416	618.364
AMK58_12905	no KO assigned | (GenBank) C4-dicarboxylate ABC transporter	Membrane Transport	2.118	2.812	2.461	32.942	7.010	24.440
AMK58_12910	no KO assigned | (GenBank) hypothetical protein	Hypothetical	73.463	73.336	82.758	315.901	139.438	303.180
AMK58_12915	K11890 type VI secretion system protein ImpM | (GenBank) hypothetical protein	Secretion System	61.194	35.887	57.649	120.194	44.520	80.590
AMK58_12920	K11891 type VI secretion system protein ImpL | (GenBank) hypothetical protein	Secretion System	33.183	25.005	34.450	81.614	20.968	58.325
AMK58_12925	K11892 type VI secretion system protein ImpK | (GenBank) flagellar motor protein MotB	Motility	60.267	52.206	56.189	152.170	26.834	165.147
AMK58_12930	no KO assigned | (GenBank) hypothetical protein	Hypothetical	175.744	111.154	144.783	260.724	27.347	245.242
AMK58_12935	no KO assigned | (GenBank) hypothetical protein	Hypothetical	393.018	313.454	373.798	294.320	39.627	242.480
AMK58_12940	K07305 peptide-methionine (R)-S-oxide reductase [EC:1.8.4.12] | (GenBank) methionine sulfoxide reduc	Oxidoreductase	13.208	29.226	21.861	147.442	21.445	68.496
AMK58_12945	K01061 carboxymethylenebutenolidase [EC:3.1.1.45] | (GenBank) carboxymethylenebutenolidase	Metabolism	10.219	10.348	14.493	141.072	18.886	41.760
AMK58_12950	K01835 phosphoglucomutase [EC:5.4.2.2] | (GenBank) phosphoglucomutase	Metabolism	18.992	21.718	22.643	185.440	20.347	68.655
AMK58_12955	K03704 cold shock protein (beta-ribbon, CspA family) | (GenBank) cold-shock protein	Stress Response	17.568	13.238	17.004	155.190	37.003	35.369
AMK58_12960	no KO assigned | (GenBank) hypothetical protein	Hypothetical	53.811	36.838	42.637	174.200	110.891	99.795
AMK58_12965	no KO assigned | (GenBank) hypothetical protein	Hypothetical	89.581	111.500	99.370	604.369	606.257	281.648
AMK58_12970	no KO assigned | (GenBank) hypothetical protein	Hypothetical	43.163	52.134	54.522	179.093	538.418	218.182
AMK58_12975	no KO assigned | (GenBank) metallophosphoesterase	phosphoesterase	51.606	60.280	59.132	141.359	86.639	114.570
AMK58_12980	no KO assigned | (GenBank) hypothetical protein	Hypothetical	644.803	689.250	720.882	324.905	349.485	178.499
AMK58_12985	no KO assigned | (GenBank) MarR family transcriptional regulator	Transcription	187.574	161.659	168.318	420.209	703.664	215.821
AMK58_12990	no KO assigned | (GenBank) ABC transporter	Membrane Transport	0.779	0.000	3.259	68.882	155.535	62.854
AMK58_12995	no KO assigned | (GenBank) pseudogene	Pseudogene	0.868	0.000	0.825	96.885	76.095	20.657
AMK58_13000	K02034 peptide/nickel transport system permease protein | (GenBank) D-ala-D-ala transporter subunit	Quorum sensing	1.328	2.379	2.398	82.516	173.399	154.125
AMK58_13005	K02033 peptide/nickel transport system permease protein | (GenBank) peptide ABC transporter permease	Transport: Metal	2.018	4.964	2.483	113.848	186.394	117.663
AMK58_13010	K02035 peptide/nickel transport system substrate-binding protein | (GenBank) ABC transporter substra	Quorum sensing	20.104	23.842	27.205	118.210	70.586	74.218
AMK58_13015	no KO assigned | (GenBank) hypothetical protein	Hypothetical	68.173	107.526	86.598	372.589	307.742	423.793
AMK58_13020	no KO assigned | (GenBank) hypothetical protein	Hypothetical	18.289	22.580	24.127	80.912	25.261	58.888
AMK58_13025	no KO assigned | (GenBank) hypothetical protein	Hypothetical	122.559	95.738	139.102	387.577	145.433	370.655
AMK58_13030	K04343 streptomycin 6-kinase [EC:2.7.1.72] | (GenBank) streptomycin kinase	Defense	65.870	65.446	73.731	294.831	50.063	201.684
AMK58_13035	K06915 uncharacterized protein | (GenBank) ATP-binding protein	No COG	61.041	52.759	68.571	109.473	26.905	122.613
AMK58_13040	no KO assigned | (GenBank) hypothetical protein	Hypothetical	586.727	547.476	604.496	122.238	138.318	209.499
AMK58_13045	K01985 5S ribosomal RNA | (GenBank) 5S ribosomal RNA	Ribosome	457.162	523.809	529.944	335.557	936.227	549.734
AMK58_13050	K01980 23S ribosomal RNA | (GenBank) 23S ribosomal RNA	Ribosome	16.932	26.801	24.253	135.675	56.751	70.184
AMK58_13055	K14218 tRNA Ala | (GenBank) tRNA-Ala	tRNA synthesis/modification	27.083	38.071	48.471	1011.528	250.698	261.470
AMK58_13060	K14227 tRNA Ile | (GenBank) tRNA-Ile	tRNA synthesis/modification	257.874	385.163	349.842	2550.742	973.862	2286.219

AMK58_13065	K01977 16S ribosomal RNA | (GenBank) 16S ribosomal RNA	Ribosome	22.463	25.194	32.076	205.312	56.971	125.655
AMK58_13070	K19302 undecaprenyl-diphosphatase [EC:3.6.1.27] | (GenBank) hypothetical protein	Cell Wall	23.528	43.094	36.087	454.351	143.622	498.127
AMK58_13075	no KO assigned | (GenBank) CopG family transcriptional regulator	Transcription	0.000	0.000	0.000	10.679	0.000	41.812
AMK58_13080	no KO assigned | (GenBank) diguanylate kinase	signal transduction	7.090	5.120	5.408	4.905	6.218	26.225
AMK58_13085	no KO assigned | (GenBank) pseudogene	Pseudogene	0.000	0.000	0.000	0.000	0.000	0.000
AMK58_13090	no KO assigned | (GenBank) hypothetical protein	Hypothetical	0.000	0.000	0.000	0.000	0.000	0.000
AMK58_13095	no KO assigned | (GenBank) phage shock protein C	Defense	0.000	0.000	0.000	0.000	0.000	0.000
AMK58_13100	K03970 phage shock protein B | (GenBank) phage shock protein B	Defense	54.696	69.797	74.221	574.764	698.373	728.768
AMK58_13105	K03969 phage shock protein A | (GenBank) phage shock protein A	Defense	22.521	36.598	25.867	212.386	99.842	141.841
AMK58_13110	K03974 psp operon transcriptional activator | (GenBank) pspF; psp operon transcriptional activator P	Transcription	100.654	144.382	168.083	315.146	168.147	227.340
AMK58_13115	no KO assigned | (GenBank) polyhydroxyalkanoic acid synthase	enzyme	8.537	4.637	11.069	396.108	389.087	343.286
AMK58_13120	K05366 penicillin-binding protein 1A [EC:2.4.1.- 3.4.-.-] | (GenBank) carboxypeptidase	Cell Wall	3.735	3.996	5.989	40.994	107.208	45.177
AMK58_13125	K07007 uncharacterized protein | (GenBank) NAD(FAD)-utilizing dehydrogenase	Energy	117.873	139.686	154.322	35.218	558.985	85.552
AMK58_13130	no KO assigned | (GenBank) hypothetical protein	Hypothetical	105.940	82.199	140.846	151.742	1539.297	275.146
AMK58_13135	K03532 trimethylamine-N-oxide reductase (cytochrome c), cytochrome c-type subunit TorC | (GenBank) c	Energy	109.062	99.469	155.896	166.844	1219.627	294.012
AMK58_13140	K00428 cytochrome c peroxidase [EC:1.11.1.5] | (GenBank) cytochrome C biogenesis protein CcsA	Energy	7.349	15.115	12.486	180.099	224.114	138.969
AMK58_13145	K03781 catalase [EC:1.11.1.6] | (GenBank) catalase	enzyme	3.143	3.414	2.853	39.422	46.248	57.691
AMK58_13150	K04077 chaperonin GroEL | (GenBank) groEL; molecular chaperone GroEL	Chaperone	31.521	32.560	36.482	282.543	96.358	169.156
AMK58_13155	K04078 chaperonin GroES | (GenBank) molecular chaperone GroES	Chaperone	89.038	99.430	75.559	888.957	239.449	550.546
AMK58_13160	no KO assigned | (GenBank) Usg family protein	No COG	158.223	158.919	121.224	111.196	119.597	308.742
AMK58_13165	K01185 lysozyme [EC:3.2.1.17] | (GenBank) muraminidase	Cell Wall	82.685	64.690	74.172	490.843	178.968	495.173
AMK58_13170	no KO assigned | (GenBank) pyridine nucleotide-disulfide oxidoreductase	oxidoreductase	93.188	76.578	90.514	44.688	26.985	70.574
AMK58_13175	K02259 cytochrome c oxidase assembly protein subunit 15 | (GenBank) heme A synthase	Energy	14.270	11.989	23.639	157.407	2690.660	397.699
AMK58_13180	K05973 poly(3-hydroxybutyrate) depolymerase [EC:3.1.1.75] | (GenBank) poly(3-hydroxybutyrate) depol	Metabolism: Carbohydrate	2993.849	2487.016	2838.941	128.978	5076.577	293.501
AMK58_13185	no KO assigned | (GenBank) endonuclease	DNA Repair and Replication	859.140	736.702	723.989	21.744	1506.567	111.027
AMK58_13190	no KO assigned | (GenBank) phospholipase	No COG	20.128	18.406	14.939	10.455	13.390	37.471
AMK58_13195	K05846 osmoprotectant transport system permease protein | (GenBank) hypothetical protein	Membrane Transport	82.441	90.677	83.305	140.238	101.779	195.113
AMK58_13200	no KO assigned | (GenBank) pseudogene	Pseudogene	144.783	188.137	191.521	1228.394	584.651	781.189
AMK58_13205	K02031 peptide/nickel transport system ATP-binding protein K02032 peptide/nickel transport system AT	Quorum sensing	77.235	72.590	75.800	104.793	287.042	79.458
AMK58_13210	no KO assigned | (GenBank) mechanosensitive ion channel protein	Transport: Ion	224.794	188.500	198.810	102.503	332.459	103.863
AMK58_13215	no KO assigned | (GenBank) transcriptional regulator	Transcription	15.458	20.017	20.635	79.869	244.711	99.419
AMK58_13220	K00762 orotate phosphoribosyltransferase [EC:2.4.2.10] | (GenBank) orotate phosphoribosyltransferase	Metabolism: Nucleotide	19.243	27.469	25.906	642.128	237.924	350.862
AMK58_13225	no KO assigned | (GenBank) glycoside hydrolase	hydrolase	3.809	3.992	3.685	22.645	3.981	4.027
AMK58_13230	K00528 ferredoxin--NADP+ reductase [EC:1.18.1.2] | (GenBank) ferredoxin-NADP reductase	Energy	3.441	3.738	6.990	75.226	8.088	6.962
AMK58_13235	K07110 uncharacterized protein | (GenBank) DNA-binding protein	No COG	9.985	12.031	11.009	157.240	16.505	52.439
AMK58_13240	K01637 isocitrate lyase [EC:4.1.3.1] | (GenBank) isocitrate lyase	Metabolism: Carbohydrate	67.737	55.469	62.784	329.829	64.319	283.046
AMK58_13245	no KO assigned | (GenBank) peptidase C56	peptidase	135.458	179.556	153.696	517.614	125.717	465.003
AMK58_13250	no KO assigned | (GenBank) hypothetical protein	Hypothetical	58.647	81.854	65.134	111.280	38.535	88.528
AMK58_13255	no KO assigned | (GenBank) molybdopterin-binding protein	No COG	224.329	241.119	240.371	449.814	323.050	425.264
AMK58_13260	K00174 2-oxoglutarate/2-oxoacid ferredoxin oxidoreductase subunit alpha [EC:1.2.7.3 1.2.7.11] | (Gen	Metabolism: Carbohydrate	219.299	165.506	139.854	30.327	208.091	126.091
AMK58_13265	K00175 2-oxoglutarate/2-oxoacid ferredoxin oxidoreductase subunit beta [EC:1.2.7.3 1.2.7.11] | (GenB	Metabolism: Carbohydrate	93.643	82.375	108.154	222.789	126.891	465.060
AMK58_13270	no KO assigned | (GenBank) rubrerythrin	No COG	284.775	293.948	212.129	314.349	168.205	573.963
AMK58_13275	K06938 uncharacterized protein | (GenBank) hypothetical protein	Hypothetical	4169.184	3658.711	4202.119	553.156	13715.170	791.311
AMK58_13280	no KO assigned | (GenBank) hypothetical protein	Hypothetical	425.314	411.138	476.219	890.636	370.476	802.440
AMK58_13285	no KO assigned | (GenBank) hypothetical protein	Hypothetical	41.894	41.934	51.303	374.320	106.754	257.081
AMK58_13290	no KO assigned | (GenBank) hypothetical protein	Hypothetical	78.304	81.246	64.650	557.661	248.765	590.839
AMK58_13295	K14219 tRNA Arg | (GenBank) tRNA-Arg	tRNA synthesis/modification	234.288	234.856	251.169	2262.177	813.024	2356.205
AMK58_13300	K00769 xanthine phosphoribosyltransferase [EC:2.4.2.22] | (GenBank) xanthine phosphoribosyltransfera	Metabolism: Nucleotide	21.267	20.214	26.195	210.097	135.822	256.808
AMK58_13305	K02034 peptide/nickel transport system permease protein | (GenBank) peptide ABC transporter permease	Quorum sensing	6.869	9.405	10.342	36.425	31.530	68.344
AMK58_13310	K02033 peptide/nickel transport system permease protein | (GenBank) peptide ABC transporter	Quorum sensing	7.152	6.867	7.893	144.564	109.391	139.982
AMK58_13315	K02035 peptide/nickel transport system substrate-binding protein | (GenBank) ABC transporter substra	Quorum sensing	2.002	1.914	3.883	44.515	284.338	79.450
AMK58_13320	K01470 creatinine amidohydrolase [EC:3.5.2.10] | (GenBank) amidase	Metabolism: Amino Acid	0.000	0.000	2.620	164.357	17.308	100.246
AMK58_13325	no KO assigned | (GenBank) pseudogene	Pseudogene	1.456	0.621	0.494	9.335	5.378	56.617
AMK58_13330	no KO assigned | (GenBank) hypothetical protein	Hypothetical	1082.491	1115.684	1070.216	142.943	505.928	336.175
AMK58_13335	no KO assigned | (GenBank) hypothetical protein	Hypothetical	140.094	169.060	121.735	457.352	195.015	320.033
AMK58_13340	no KO assigned | (GenBank) hypothetical protein	Hypothetical	44.106	43.057	47.692	396.969	132.205	123.497
AMK58_13345	K07483 transposase | (GenBank) hypothetical protein	Genetic Information Processing	738.890	661.716	660.708	717.845	558.016	558.253
AMK58_13350	no KO assigned | (GenBank) pseudogene	Pseudogene	1049.315	1094.307	1088.464	1418.087	1138.472	819.713
AMK58_13355	no KO assigned | (GenBank) hypothetical protein	Hypothetical	440.578	602.796	578.792	1487.605	426.425	855.121
AMK58_13360	no KO assigned | (GenBank) pseudogene	Pseudogene	2.547	3.803	4.567	39.849	9.043	30.214
AMK58_13365	no KO assigned | (GenBank) hypothetical protein	Hypothetical	7.677	5.897	9.072	50.830	12.451	111.660
AMK58_13370	no KO assigned | (GenBank) hypothetical protein	Hypothetical	10.025	11.359	16.187	94.811	12.337	39.106
AMK58_13375	no KO assigned | (GenBank) hypothetical protein	Hypothetical	2.018	1.855	3.468	74.959	8.462	40.878
AMK58_13380	no KO assigned | (GenBank) transposase	Genetic Information Processing	0.809	0.000	0.979	4.727	3.801	12.134
AMK58_13385	K07484 transposase | (GenBank) transposase	Genetic Information Processing	4.175	4.157	3.970	18.176	20.727	100.978
AMK58_13390	no KO assigned | (GenBank) transposase	Genetic Information Processing	313.049	375.600	350.731	248.526	140.663	479.422
AMK58_13395	no KO assigned | (GenBank) hypothetical protein	Hypothetical	72.895	95.326	82.100	58.253	388.235	41.775

AMK58_13400	no KO assigned | (GenBank) hypothetical protein	Hypothetical	891.758	1048.934	799.545	408.061	217.595	476.353
AMK58_13405	no KO assigned | (GenBank) pseudogene	Pseudogene	2039.847	2358.768	2028.763	1051.270	347.136	1956.177
AMK58_13410	K07483 transposase | (GenBank) transposase	Genetic Information Processing	79.882	104.762	104.533	705.415	324.434	626.904
AMK58_13415	no KO assigned | (GenBank) integrase	Genetic Information Processing	2.640	3.380	3.766	100.166	40.701	72.136
AMK58_13420	no KO assigned | (GenBank) hypothetical protein	Hypothetical	7.338	6.263	6.977	30.190	40.062	112.270
AMK58_13425	no KO assigned | (GenBank) pseudogene	Pseudogene	25.600	32.749	31.787	40.748	38.052	63.955
AMK58_13430	no KO assigned | (GenBank) transposase	Genetic Information Processing	288.074	272.159	262.789	58.785	99.906	232.024
AMK58_13435	no KO assigned | (GenBank) ATP-binding protein	No COG	67.161	69.022	77.478	95.124	36.880	69.133
AMK58_13440	no KO assigned | (GenBank) transposase	Genetic Information Processing	99.787	108.636	98.041	164.859	96.212	254.355
AMK58_13445	no KO assigned | (GenBank) hypothetical protein	Hypothetical	5.144	21.275	17.682	777.538	145.433	1224.693
AMK58_13450	no KO assigned | (GenBank) hypothetical protein	Hypothetical	3.363	2.870	5.482	157.681	34.563	321.662
AMK58_13455	no KO assigned | (GenBank) hypothetical protein	Hypothetical	10.317	23.568	24.524	982.464	99.597	957.247
AMK58_13460	no KO assigned | (GenBank) hypothetical protein	Hypothetical	3.459	8.611	12.882	780.809	20.414	426.357
AMK58_13465	no KO assigned | (GenBank) hypothetical protein	Hypothetical	3.972	2.848	9.367	167.081	13.395	180.633
AMK58_13470	no KO assigned | (GenBank) hypothetical protein	Hypothetical	6.647	8.510	14.898	393.698	103.004	671.857
AMK58_13475	no KO assigned | (GenBank) hypothetical protein	Hypothetical	25.024	28.664	30.054	10.712	32.887	62.634
AMK58_13480	no KO assigned | (GenBank) hypothetical protein	Hypothetical	50.228	44.370	55.190	55.109	20.870	168.812
AMK58_13485	no KO assigned | (GenBank) hypothetical protein	Hypothetical	40.358	75.350	49.765	199.353	192.799	849.624
AMK58_13490	K07484 transposase | (GenBank) hypothetical protein	Hypothetical	11.283	10.371	19.805	144.747	178.531	418.496
AMK58_13495	no KO assigned | (GenBank) hypothetical protein	Hypothetical	35.932	42.001	45.552	153.117	123.508	455.839
AMK58_13500	no KO assigned | (GenBank) hypothetical protein	Hypothetical	18.345	23.290	25.618	16.126	24.744	44.759
AMK58_13505	K06871 uncharacterized protein | (GenBank) FxsB family radical SAM/SPASM domain protein	No COG	46.580	60.706	47.871	60.179	83.157	106.394
AMK58_13510	no KO assigned | (GenBank) hypothetical protein	Hypothetical	69.186	95.682	117.555	700.231	528.531	2174.705
AMK58_13515	no KO assigned | (GenBank) hypothetical protein	Hypothetical	1.953	7.778	11.141	99.404	58.535	288.521
AMK58_13520	no KO assigned | (GenBank) hypothetical protein	Hypothetical	0.651	0.000	4.951	286.005	68.778	310.854
AMK58_13525	no KO assigned | (GenBank) hypothetical protein	Hypothetical	3.232	4.208	4.846	10.102	8.058	32.428
AMK58_13530	no KO assigned | (GenBank) pseudogene	Pseudogene	187.666	226.049	308.423	279.095	294.302	827.521
AMK58_13535	no KO assigned | (GenBank) pseudogene	Pseudogene	753.021	860.853	1052.138	151.761	349.668	443.615
AMK58_13540	no KO assigned | (GenBank) hypothetical protein	Hypothetical	559.931	748.037	770.481	129.834	70.726	239.273
AMK58_13545	no KO assigned | (GenBank) hypothetical protein	Hypothetical	1.510	1.203	1.675	25.818	4.186	25.758
AMK58_13550	no KO assigned | (GenBank) AAA family ATPase	Enzyme	2.907	2.672	5.740	35.792	10.430	59.297
AMK58_13555	K00449 protocatechuate 3,4-dioxygenase, beta subunit [EC:1.13.11.3] | (GenBank) hypothetical protein	Metabolism	673.111	697.911	783.272	141.811	56.307	607.046
AMK58_13560	no KO assigned | (GenBank) 3'-5' exonuclease	DNA Repair and Replication	154.389	174.482	223.959	30.471	39.096	102.880
AMK58_13565	no KO assigned | (GenBank) hypothetical protein	Hypothetical	14.980	15.267	20.359	133.856	66.759	200.762
AMK58_13570	K07286 uncharacterized lipoprotein | (GenBank) hypothetical protein	Hypothetical	104.969	87.378	108.078	45.878	47.653	248.058
AMK58_13575	K07337 penicillin-binding protein activator | (GenBank) penicillin-binding protein activator LpoB	No COG	363.618	371.675	318.215	316.988	388.142	940.692
AMK58_13580	no KO assigned | (GenBank) hypothetical protein	Hypothetical	1021.682	1207.765	947.916	246.445	639.188	1142.251
AMK58_13585	no KO assigned | (GenBank) hypothetical protein	Hypothetical	3.260	4.637	3.985	243.279	19.891	144.943
AMK58_13590	no KO assigned | (GenBank) hypothetical protein	Hypothetical	35.358	34.141	47.542	510.957	78.685	548.764
AMK58_13595	no KO assigned | (GenBank) hypothetical protein	Hypothetical	77.697	97.596	107.157	241.624	51.536	289.712
AMK58_13600	no KO assigned | (GenBank) hypothetical protein	Hypothetical	207.893	253.270	210.593	32.695	8.303	112.766
AMK58_13605	no KO assigned | (GenBank) hypothetical protein	Hypothetical	191.137	252.692	225.940	333.454	23.952	450.875
AMK58_13610	no KO assigned | (GenBank) hypothetical protein	Hypothetical	51.930	53.957	66.012	78.258	11.738	168.535
AMK58_13615	no KO assigned | (GenBank) hypothetical protein	Hypothetical	55.652	60.914	67.557	82.800	50.617	113.353
AMK58_13620	no KO assigned | (GenBank) hypothetical protein	Hypothetical	14.603	13.484	18.050	43.036	13.132	93.804
AMK58_13625	K07497 putative transposase | (GenBank) hypothetical protein	Genetic Information Processing	5.077	6.445	6.818	56.442	9.130	127.157
AMK58_13630	no KO assigned | (GenBank) hypothetical protein	Hypothetical	132.934	125.702	133.669	215.735	35.690	287.857
AMK58_13635	no KO assigned | (GenBank) hypothetical protein	Hypothetical	89.232	141.574	133.777	310.980	21.642	168.920
AMK58_13640	no KO assigned | (GenBank) hypothetical protein	Hypothetical	40.045	41.121	65.145	599.711	106.554	454.010
AMK58_13645	K07391 magnesium chelatase family protein | (GenBank) hypothetical protein	No COG	54.564	38.579	62.011	320.047	76.938	514.345
AMK58_13650	no KO assigned | (GenBank) hypothetical protein	Hypothetical	77.987	76.878	88.795	472.193	107.823	385.983
AMK58_13655	no KO assigned | (GenBank) cell envelope biogenesis protein LolA	Membrane	21.366	29.458	26.219	146.285	53.780	175.601
AMK58_13660	K03466 DNA segregation ATPase FtsK/SpoIIIE, S-DNA-T family | (GenBank) cell division protein FtsK	Cell Cycle/Shape/Homeostatsis	7.525	9.164	7.884	8.347	9.375	55.468
AMK58_13665	no KO assigned | (GenBank) aspartate aminotransferase	tRNA synthesis/modification	11.103	16.151	13.495	36.472	42.109	107.614
AMK58_13670	K04752 nitrogen regulatory protein P-II 2 | (GenBank) transcriptional regulator	Nitrogen	1.429	0.000	2.038	112.908	105.181	80.476
AMK58_13675	K03185 2-octaprenyl-6-methoxyphenol hydroxylase [EC:1.14.13.-] | (GenBank) 2-octaprenyl-6-methoxypl	Metabolism: Co-Factors and Vitamens	4.452	5.204	6.718	105.920	239.985	101.859
AMK58_13680	K01975 2'-5' RNA ligase [EC:6.5.1.-] | (GenBank) 2'-5' RNA ligase	tRNA synthesis/modification	52.082	71.393	48.234	95.439	112.017	229.557
AMK58_13685	no KO assigned | (GenBank) aldo/keto reductase	Oxidoreductases	174.306	190.539	202.892	394.901	128.402	411.763
AMK58_13690	no KO assigned | (GenBank) histidine kinase	Signal Transduction	144.548	153.159	172.874	267.552	118.156	188.262
AMK58_13695	K10804 acyl-CoA thioesterase I [EC:3.1.2.- 3.1.1.5] | (GenBank) acyl-CoA thioesterase	Lipid metabolism	173.088	210.979	156.469	66.412	38.716	189.374
AMK58_13700	K02003 putative ABC transport system ATP-binding protein | (GenBank) ABC transporter	Membrane Transport	231.113	226.356	219.431	301.423	136.464	231.566
AMK58_13705	K02004 putative ABC transport system permease protein | (GenBank) glycosyl transferase family 1	Membrane Transport	13.037	14.032	13.535	55.025	15.786	37.957
AMK58_13710	no KO assigned | (GenBank) diguanylate cyclase	cyclic nucleotide	133.418	145.569	117.466	178.002	122.339	175.272
AMK58_13715	K03686 molecular chaperone DnaJ | (GenBank) molecular chaperone DnaJ	Chaperone	80.822	82.904	82.084	182.193	188.244	144.291
AMK58_13720	K04043 molecular chaperone DnaK | (GenBank) dnaK; Fe-S protein assembly chaperone HscA	Chaperone	20.494	28.256	28.420	75.856	85.129	53.913
AMK58_13725	no KO assigned | (GenBank) hypothetical protein	Hypothetical	151.343	141.834	158.571	779.923	1450.326	484.301
AMK58_13730	no KO assigned | (GenBank) hypothetical protein	Hypothetical	84.236	109.344	100.394	791.128	319.661	470.836

AMK58_13735	no KO assigned | (GenBank) DNA repair photolyase	DNA Repair and Replication	210.957	228.041	222.173	253.267	67.662	296.849
AMK58_13740	no KO assigned | (GenBank) chemotaxis protein	Chemotaxis	184.355	171.669	165.479	156.374	872.414	141.115
AMK58_13745	K03413 two-component system, chemotaxis family, response regulator CheY | (GenBank) Fis family trans	Chemotaxis	3886.633	3587.132	3778.795	558.788	21236.860	1265.028
AMK58_13750	K03687 molecular chaperone GrpE | (GenBank) molecular chaperone GrpE	Chaperone	205.569	273.188	224.945	55.127	41.565	129.230
AMK58_13755	K03705 heat-inducible transcriptional repressor | (GenBank) hrcA; heat-inducible transcription repre	Transcription	58.502	59.064	62.149	75.406	55.169	71.180
AMK58_13760	K00989 ribonuclease PH [EC:2.7.7.56] | (GenBank) ribonuclease PH	post translation modificaton	22.870	29.135	25.582	220.753	115.868	135.347
AMK58_13765	K02428 XTP/dITP diphosphohydrolase [EC:3.6.1.66] | (GenBank) non-canonical purine NTP pyrophosph	Metabolism: Nucleotide	95.173	98.367	151.010	271.117	323.645	535.469
AMK58_13770	no KO assigned | (GenBank) coproporphyrinogen III oxidase	Biosynthesis: Co-Factors and Vitamins	16.704	14.876	14.501	70.739	47.930	47.236
AMK58_13775	K03406 methyl-accepting chemotaxis protein | (GenBank) chemotaxis protein	Chemotaxis	78.713	98.328	72.528	70.357	434.823	76.107
AMK58_13780	K00287 dihydrofolate reductase [EC:1.5.1.3] | (GenBank) dihydrofolate reductase	Metabolism: Co-Factors and Vitamins	113.433	134.113	142.139	413.942	1631.508	574.413
AMK58_13785	no KO assigned | (GenBank) hypothetical protein	Hypothetical	180.367	125.024	134.069	418.480	215.621	496.204
AMK58_13790	no KO assigned | (GenBank) hypothetical protein	Hypothetical	98.217	72.549	103.573	390.394	244.486	314.951
AMK58_13795	K01920 glutathione synthase [EC:6.3.2.3] | (GenBank) glutathione synthetase	Metabolism: Amino Acid	23.026	17.385	20.450	250.312	84.045	148.520
AMK58_13800	no KO assigned | (GenBank) hypothetical protein	Hypothetical	9.291	14.745	15.460	449.414	127.281	355.924
AMK58_13805	K07460 putative endonuclease | (GenBank) hypothetical protein	DNA Repair and Replication	30.515	41.167	45.860	336.239	96.630	130.525
AMK58_13810	K07056 16S rRNA (cytidine1402-2'-O)-methyltransferase [EC:2.1.1.198] | (GenBank) 16S rRNA methylt	Ribosome	10.311	7.040	7.563	29.596	25.993	55.998
AMK58_13815	no KO assigned | (GenBank) ABC transporter substrate-binding protein	Membrane Transport	23.280	27.938	22.908	15.660	8.913	45.907
AMK58_13820	no KO assigned | (GenBank) hypothetical protein	Hypothetical	38.950	47.663	46.405	28.917	31.913	60.704
AMK58_13825	K10764 O-succinylhomoserine sulfhydrylase [EC:2.5.1.-] | (GenBank) O-succinylhomoserine sulfhydrylas	Energy	6.910	6.581	7.237	43.330	11.594	45.131
AMK58_13830	no KO assigned | (GenBank) hypothetical protein	Hypothetical	12.653	3.910	15.142	80.362	22.314	28.320
AMK58_13835	no KO assigned | (GenBank) hypothetical protein	Hypothetical	52.267	71.150	61.648	325.258	53.545	96.932
AMK58_13840	no KO assigned | (GenBank) pseudogene	Pseudogene	0.000	0.000	0.000	0.000	0.000	0.000
AMK58_13845	no KO assigned | (GenBank) pseudogene	Pseudogene	8.810	1.350	13.316	19.063	18.027	51.317
AMK58_13850	no KO assigned | (GenBank) hypothetical protein	Hypothetical	0.000	0.000	0.000	0.000	0.000	0.000
AMK58_13855	no KO assigned | (GenBank) hypothetical protein	Hypothetical	0.000	0.000	0.000	0.000	0.000	0.000
AMK58_13860	K01978 M1 RNA | (GenBank) bacterial RNase P	post translation modificaton	0.000	0.000	0.000	0.000	0.000	0.000
AMK58_13865	no KO assigned | (GenBank) hypothetical protein	Hypothetical	0.000	0.000	0.000	0.000	0.000	0.000
AMK58_13870	K03408 purine-binding chemotaxis protein CheW | (GenBank) hypothetical protein	Chemotaxis	0.000	0.000	0.000	0.000	0.000	0.000
AMK58_13875	K01579 aspartate 1-decarboxylase [EC:4.1.1.11] | (GenBank) aspartate decarboxylase	Metabolism	0.000	0.000	0.000	0.000	0.000	0.000
AMK58_13880	no KO assigned | (GenBank) hypothetical protein	Hypothetical	0.000	0.000	0.000	0.000	0.000	0.000
AMK58_13885	no KO assigned | (GenBank) hypothetical protein	Hypothetical	0.000	0.000	0.000	0.000	0.000	0.000
AMK58_13890	no KO assigned | (GenBank) endonuclease	DNA Repair and Replication	0.000	0.000	0.000	0.000	0.000	0.000
AMK58_13895	no KO assigned | (GenBank) hypothetical protein	Hypothetical	0.000	0.000	0.000	0.000	0.000	0.000
AMK58_13900	K03411 chemotaxis protein CheD [EC:3.5.1.44] | (GenBank) chemotaxis protein	Chemotaxis	0.000	0.000	0.000	0.000	0.000	0.000
AMK58_13905	K07259 D-alanyl-D-alanine carboxypeptidase / D-alanyl-D-alanine-endopeptidase (penicillin-binding pr	Cell Wall	0.000	0.000	0.000	0.000	0.000	0.000
AMK58_13910	no KO assigned | (GenBank) hypothetical protein	Hypothetical	0.000	0.000	0.000	0.000	0.000	0.000
AMK58_13915	K06133 4'-phosphopantetheinyl transferase [EC:2.7.8.-] | (GenBank) 4-phosphopantetheinyl transferase	Metabolism: Co-Factors and Vitamens	5.503	5.978	8.087	17.202	17.996	11.443
AMK58_13920	K01923 phosphoribosylaminoimidazole-succinocarboxamide synthase [EC:6.3.2.6] | (GenBank) phosphori	Metabolism: Nucleotide	0.000	0.000	0.000	0.000	0.000	0.000
AMK58_13925	no KO assigned | (GenBank) hydroxyacid dehydrogenase	dehydrogenase	0.256	0.765	0.122	7.380	11.089	11.643
AMK58_13930	K02806 PTS system, nitrogen regulatory IIA component [EC:2.7.1.-] | (GenBank) transcriptional regula	Nitrogen	58.433	78.401	76.981	215.584	100.191	202.269
AMK58_13935	K05559 multicomponent K+:H+ antiporter subunit A | (GenBank) cation:proton antiporter	Transport: Ion	2.235	2.428	2.744	42.574	10.873	19.861
AMK58_13940	K05560 multicomponent K+:H+ antiporter subunit C | (GenBank) cation:proton antiporter	Transport: Ion	502.899	438.207	423.437	352.578	196.842	406.123
AMK58_13945	K05561 multicomponent K+:H+ antiporter subunit D | (GenBank) cation:proton antiporter	Transport: Ion	51.932	44.766	56.219	94.310	39.027	103.189
AMK58_13950	K05562 multicomponent K+:H+ antiporter subunit E | (GenBank) cation:proton antiporter	Transport: Ion	366.443	387.565	373.371	229.183	953.209	340.942
AMK58_13955	K05563 multicomponent K+:H+ antiporter subunit F | (GenBank) cation:proton antiporter	Transport: Ion	1438.729	1310.761	1585.131	2402.882	2069.389	1574.562
AMK58_13960	K05564 multicomponent K+:H+ antiporter subunit G | (GenBank) cation:proton antiporter	Transport: Ion	70.476	55.781	69.873	68.183	80.573	39.099
AMK58_13965	K06991 uncharacterized protein | (GenBank) dioxygenase	No COG	421.615	446.326	496.944	736.163	355.194	552.324
AMK58_13970	no KO assigned | (GenBank) hypothetical protein	Hypothetical	23.534	20.967	26.498	25.164	18.912	25.555
AMK58_13975	no KO assigned | (GenBank) hypothetical protein	Hypothetical	37.452	41.472	30.698	65.091	22.138	54.788
AMK58_13980	K07117 uncharacterized protein | (GenBank) hypothetical protein	Hypothetical	100.200	91.459	132.850	82.771	43.487	76.653
AMK58_13985	no KO assigned | (GenBank) hypothetical protein	Hypothetical	162.161	179.596	147.459	44.448	55.069	95.680
AMK58_13990	K06160 putative ATP-binding cassette transporter | (GenBank) hypothetical protein	Membrane transport	2.545	1.738	4.702	140.074	44.874	192.173
AMK58_13995	no KO assigned | (GenBank) hypothetical protein	Hypothetical	2.278	3.266	4.764	127.162	17.818	131.024
AMK58_14000	K06159 putative ATP-binding cassette transporter | (GenBank) hypothetical protein	Membrane transport	0.070	0.000	0.601	56.130	2.998	11.325
AMK58_14005	K02030 polar amino acid transport system substrate-binding protein | (GenBank) hypothetical protein	Transport: Amino Acid	7.309	9.493	13.899	333.311	37.685	265.646
AMK58_14010	no KO assigned | (GenBank) hypothetical protein	Hypothetical	19.475	33.644	31.680	713.178	88.883	541.331
AMK58_14015	K11891 type VI secretion system protein ImpL | (GenBank) hypothetical protein	Secretion System	2.118	1.746	2.639	25.245	14.164	49.107
AMK58_14020	no KO assigned | (GenBank) hypothetical protein	Hypothetical	9.633	9.923	12.475	468.314	60.673	485.424
AMK58_14025	K11918 type VI secretion system protein | (GenBank) hypothetical protein	Secretion System	18.487	12.445	15.349	249.383	36.292	413.346
AMK58_14030	no KO assigned | (GenBank) hypothetical protein	Hypothetical	60.537	42.100	58.473	251.002	19.082	281.862
AMK58_14035	no KO assigned | (GenBank) hypothetical protein	Hypothetical	2.471	2.812	5.221	130.820	7.935	41.678
AMK58_14040	K12542 membrane fusion protein, adhesin transport system | (GenBank) hypothetical protein	Secretion system	0.874	1.044	1.329	41.307	2.357	8.100
AMK58_14045	K12541 ATP-binding cassette, subfamily C, bacterial LapB | (GenBank) hypothetical protein	Secretion system	0.144	0.000	0.479	38.258	1.213	4.069
AMK58_14050	K12543 outer membrane protein, adhesin transport system | (GenBank) hypothetical protein	Membrane	0.370	0.000	0.704	67.701	1.457	11.072
AMK58_14055	no KO assigned | (GenBank) hypothetical protein	Hypothetical	2.072	1.188	2.506	69.848	5.850	50.118
AMK58_14060	K10112 multiple sugar transport system ATP-binding protein | (GenBank) sugar ABC transporter	Transport: Sugar	2.547	2.926	5.495	269.522	11.671	160.684
AMK58_14065	K02026 multiple sugar transport system permease protein | (GenBank) sugar ABC transporter	Transport: Sugar	4.713	7.040	6.582	267.350	30.464	113.226

AMK58_14070	K02025 multiple sugar transport system permease protein | (GenBank) sugar ABC transporter	Transport: Sugar	4.527	10.047	9.101	354.002	19.920	195.392
AMK58_14075	K02027 multiple sugar transport system substrate-binding protein | (GenBank) sugar ABC transporter	Transport: Sugar	27.926	35.996	37.282	1019.875	36.872	599.304
AMK58_14080	K13877 2,5-dioxopentanoate dehydrogenase [EC:1.2.1.26] | (GenBank) 2,5-dioxovalerate dehydrogenase	Metabolism: Carbohydrate	17.550	21.248	20.216	106.570	10.689	93.128
AMK58_14085	no KO assigned | (GenBank) fumarylacetoacetate hydrolase	hydrolase	15.224	10.377	17.972	79.930	9.647	158.314
AMK58_14090	no KO assigned | (GenBank) enoyl-CoA hydratase	hydratase	34.896	32.633	39.960	309.535	23.023	214.269
AMK58_14095	K02027 multiple sugar transport system substrate-binding protein | (GenBank) hypothetical protein	Transport: Sugar	82.293	76.605	97.431	154.302	31.897	246.954
AMK58_14100	no KO assigned | (GenBank) racemase	enzyme	4.366	8.115	7.103	251.076	11.014	75.206
AMK58_14105	no KO assigned | (GenBank) oxidoreductase	Oxidoreductase	4.115	4.728	10.684	291.723	19.569	245.085
AMK58_14110	K06145 LacI family transcriptional regulator, gluconate utilization system Gnt-I transcriptional rep	Transcription	1.938	1.362	3.469	145.387	6.279	67.658
AMK58_14115	K02014 iron complex outermembrane recepter protein | (GenBank) TonB-dependent receptor	Transport	0.176	1.050	1.949	95.334	5.201	38.129
AMK58_14120	no KO assigned | (GenBank) cobalt ABC transporter substrate-binding protein	transport	4.412	4.881	8.079	330.500	35.079	269.084
AMK58_14125	no KO assigned | (GenBank) iron uptake protein	Transport: Metal	5.300	4.871	10.465	612.156	77.898	373.476
AMK58_14130	no KO assigned | (GenBank) peptidase	peptidase	14.945	12.237	13.921	83.938	13.473	123.665
AMK58_14135	no KO assigned | (GenBank) hypothetical protein	Hypothetical	17133.343	13459.939	12499.998	1010.283	206.975	1478.217
AMK58_14140	K06200 carbon starvation protein | (GenBank) carbon starvation protein A	Starvation	663.188	474.188	545.186	47.000	8.428	65.070
AMK58_14145	no KO assigned | (GenBank) hypothetical protein	Hypothetical	729.416	758.814	723.444	297.341	27.352	209.680
AMK58_14150	no KO assigned | (GenBank) cyclase dehydrase	hydratase	1683.285	1629.961	1255.345	375.962	60.659	423.203
AMK58_14155	no KO assigned | (GenBank) alcohol dehydrogenase	dehydrogenase	68.950	57.350	52.702	63.326	6.265	28.713
AMK58_14160	no KO assigned | (GenBank) GntR family transcriptional regulator	Transcription	1328.787	1345.356	1085.650	457.135	348.864	647.289
AMK58_14165	K02030 polar amino acid transport system substrate-binding protein | (GenBank) amino acid ABC transp	Transport: Amino Acid	75.213	100.466	63.083	43.618	91.588	69.035
AMK58_14170	K02029 polar amino acid transport system permease protein | (GenBank) polar amino acid ABC transport	Transport: Amino Acid	35.674	46.286	32.206	125.260	170.116	60.144
AMK58_14175	K02029 polar amino acid transport system permease protein | (GenBank) amino acid ABC transporter per	Transport: Amino Acid	6.971	4.384	8.547	180.616	1096.787	342.422
AMK58_14180	K02028 polar amino acid transport system ATP-binding protein [EC:3.6.3.21] | (GenBank) amino acid AB	Transport: Amino Acid	0.824	0.984	4.855	186.017	17.775	115.477
AMK58_14185	no KO assigned | (GenBank) hypothetical protein	Hypothetical	5.445	2.870	10.659	187.072	53.465	203.749
AMK58_14190	no KO assigned | (GenBank) hypothetical protein	Hypothetical	1.118	3.037	3.093	166.816	21.597	105.954
AMK58_14195	no KO assigned | (GenBank) hypothetical protein	Hypothetical	2.917	3.389	3.313	38.430	66.498	74.997
AMK58_14200	no KO assigned | (GenBank) hypothetical protein	Hypothetical	13.693	18.658	15.304	191.148	123.942	263.522
AMK58_14205	K01950 NAD+ synthase (glutamine-hydrolysing) [EC:6.3.5.1] | (GenBank) nadE; NAD synthetase	Energy	15.659	18.357	20.226	47.413	6.509	56.491
AMK58_14210	K03406 methyl-accepting chemotaxis protein | (GenBank) hypothetical protein	Chemotaxis	2.643	2.987	6.588	131.375	613.744	103.725
AMK58_14215	K01750 ornithine cyclodeaminase [EC:4.3.1.12] | (GenBank) hypothetical protein	Metabolism: Amino Acid	3.691	8.086	9.944	308.219	734.267	263.560
AMK58_14220	K01432 arylformamidase [EC:3.5.1.9] | (GenBank) esterase	Metabolism	1.141	1.704	3.254	43.937	53.860	23.406
AMK58_14225	K02049 NitT/TauT family transport system ATP-binding protein | (GenBank) sulfonate ABC transporter A	Transport: NitT/TauT	211.528	189.517	212.470	593.630	380.037	470.921
AMK58_14230	K02051 NitT/TauT family transport system substrate-binding protein | (GenBank) hypothetical protein	Transport: NitT/TauT	11.083	10.796	11.798	314.205	46.989	150.810
AMK58_14235	K02050 NitT/TauT family transport system permease protein | (GenBank) hypothetical protein	Transport: NitT/TauT	8.138	8.930	9.475	250.287	28.749	101.797
AMK58_14240	no KO assigned | (GenBank) hypothetical protein	Hypothetical	7.799	10.483	10.937	241.059	26.517	110.643
AMK58_14245	no KO assigned | (GenBank) 3-demethylubiquinone-9 3-methyltransferase	methyltransferase	18.918	16.577	22.784	369.987	44.797	264.769
AMK58_14250	K08169 MFS transporter, DHA2 family, multidrug resistance protein | (GenBank) multidrug ABC transpo	Transport: MFS	70.897	51.704	53.880	90.958	43.400	176.699
AMK58_14255	no KO assigned | (GenBank) LysR family transcriptional regulator	Transcription	31.967	31.831	29.761	114.190	64.977	238.372
AMK58_14260	no KO assigned | (GenBank) AraC family transcriptional regulator	Transcription	22.026	33.511	31.609	131.581	143.936	137.514
AMK58_14265	K02014 iron complex outermembrane recepter protein | (GenBank) ferrichrome-iron receptor	Transport: Metal	23.889	28.209	23.302	31.925	15.227	49.203
AMK58_14270	K02013 iron complex transport system ATP-binding protein [EC:3.6.3.34] | (GenBank) iron ABC transpor	Transport: Metal	13.453	16.440	19.332	238.364	46.739	90.156
AMK58_14275	K02016 iron complex transport system substrate-binding protein | (GenBank) amino acid ABC transporte	Transport: Metal	8.369	10.483	14.631	178.278	41.571	115.023
AMK58_14280	K02015 iron complex transport system permease protein | (GenBank) iron ABC transporter	Transport: Metal	52.729	61.469	49.669	89.830	12.873	76.891
AMK58_14285	K06147 ATP-binding cassette, subfamily B, bacterial | (GenBank) multidrug ABC transporter ATP-bindin	Membrane Transport	50.033	55.211	48.258	184.623	39.521	287.201
AMK58_14290	no KO assigned | (GenBank) hypothetical protein	Hypothetical	47.085	63.150	60.986	276.102	37.534	219.861
AMK58_14295	no KO assigned | (GenBank) hypothetical protein	Hypothetical	30.541	35.842	44.596	612.215	57.625	207.345
AMK58_14300	no KO assigned | (GenBank) hypothetical protein	Hypothetical	12.622	14.895	14.371	162.116	9.166	49.657
AMK58_14305	K02470 DNA gyrase subunit B [EC:5.99.1.3] | (GenBank) DNA gyrase subunit B	DNA Repair and Replication	56.784	48.923	38.836	95.653	29.202	152.540
AMK58_14310	K03629 DNA replication and repair protein RecF | (GenBank) recombinase RecF	DNA Repair and Replication	8.832	11.044	9.374	56.263	33.937	114.079
AMK58_14315	K02338 DNA polymerase III subunit beta [EC:2.7.7.7] | (GenBank) DNA polymerase III subunit beta	DNA Repair and Replication	1.606	1.279	6.616	10.755	14.680	66.409
AMK58_14320	no KO assigned | (GenBank) NAD(P)-dependent oxidoreductase	Energy	45.712	59.049	48.815	563.421	180.840	386.579
AMK58_14325	no KO assigned | (GenBank) pseudogene	Pseudogene	1333.550	1453.279	1495.053	447.431	2255.334	571.396
AMK58_14330	K04758 ferrous iron transport protein A | (GenBank) hypothetical protein	Transport: Metal	207.441	231.474	260.169	817.957	2786.281	491.741
AMK58_14335	K04759 ferrous iron transport protein B | (GenBank) iron transporter FeoB	Transport: Metal	184.111	198.253	191.195	106.236	290.764	121.299
AMK58_14340	no KO assigned | (GenBank) hypothetical protein	Hypothetical	144.893	146.252	164.815	1234.562	107.833	544.784
AMK58_14345	no KO assigned | (GenBank) hypothetical protein	Hypothetical	32.080	35.329	44.487	114.560	19.442	38.383
AMK58_14350	no KO assigned | (GenBank) chloroperoxidase	Defense	83.358	89.433	64.467	27.326	32.497	40.009
AMK58_14355	no KO assigned | (GenBank) two-component system response regulator	Signal Transduction	279.597	367.474	297.391	481.359	141.982	321.156
AMK58_14360	no KO assigned | (GenBank) histidine kinase	Signal Transduction	5.872	8.437	6.431	12.843	2.590	1.516
AMK58_14365	no KO assigned | (GenBank) chloroperoxidase	Defense	214.223	250.806	263.814	459.044	165.054	499.533
AMK58_14370	no KO assigned | (GenBank) XapX domain-containing protein	No COG	291.387	320.688	377.974	574.679	408.733	477.146
AMK58_14375	no KO assigned | (GenBank) amidohydrolase	Hydrolase	10.258	12.111	16.996	63.196	30.726	44.942
AMK58_14380	no KO assigned | (GenBank) hypothetical protein	Hypothetical	149.266	166.655	162.803	1036.320	337.898	420.461
AMK58_14385	no KO assigned | (GenBank) MFS transporter	Transport: MFS	29.936	20.756	20.803	58.716	14.623	89.103
AMK58_14390	no KO assigned | (GenBank) hypothetical protein	Hypothetical	0.688	1.028	0.655	22.478	1.741	19.390
AMK58_14395	no KO assigned | (GenBank) hypothetical protein	Hypothetical	17.981	13.130	23.936	758.206	76.356	447.041
AMK58_14400	K14221 tRNA Asp | (GenBank) tRNA-Asp	tRNA synthesis/modification	6.290	18.788	22.426	992.072	72.465	332.435

AMK58_14405	K14220 tRNA Asn | (GenBank) tRNA-Asn	tRNA synthesis/modification	36.641	19.036	59.074	3555.285	465.582	1282.238
AMK58_14410	no KO assigned | (GenBank) hypothetical protein	Hypothetical	5.228	1.249	13.520	470.311	127.396	228.999
AMK58_14415	no KO assigned | (GenBank) hypothetical protein	Hypothetical	49.717	55.912	67.292	588.413	525.964	514.163
AMK58_14420	no KO assigned | (GenBank) hypothetical protein	Hypothetical	14.544	17.378	16.594	4.708	64.573	29.669
AMK58_14425	K11208 GST-like protein | (GenBank) hypothetical protein	No COG	0.189	0.000	0.538	16.673	5.724	25.881
AMK58_14430	no KO assigned | (GenBank) LysR family transcriptional regulator	Transcription	19.262	31.314	25.416	140.421	47.279	51.032
AMK58_14435	no KO assigned | (GenBank) mandelate racemase	enzyme	23.268	28.910	34.947	26.617	18.400	27.781
AMK58_14440	no KO assigned | (GenBank) hypothetical protein	Hypothetical	51.120	49.460	55.493	344.333	192.159	301.497
AMK58_14445	no KO assigned | (GenBank) hypothetical protein	Hypothetical	4.178	4.992	4.568	51.096	4.743	34.711
AMK58_14450	K01866 tyrosyl-tRNA synthetase [EC:6.1.1.1] | (GenBank) tyrosine--tRNA ligase	tRNA synthesis/modification	4.505	4.486	6.514	114.284	14.453	45.691
AMK58_14455	no KO assigned | (GenBank) DNA repair photolyase	DNA Repair and Replication	18.886	22.164	17.209	151.645	57.748	253.519
AMK58_14460	no KO assigned | (GenBank) hypothetical protein	Hypothetical	1.832	2.264	2.282	24.620	32.657	42.343
AMK58_14465	no KO assigned | (GenBank) hypothetical protein	Hypothetical	168.014	170.909	170.603	330.039	364.198	185.808
AMK58_14470	no KO assigned | (GenBank) hypothetical protein	Hypothetical	335.179	240.863	193.206	83.899	366.087	71.239
AMK58_14475	no KO assigned | (GenBank) glycosyltransferase	glycosyltransferase	9.769	10.612	12.666	112.618	44.699	152.346
AMK58_14480	no KO assigned | (GenBank) hypothetical protein	Hypothetical	6.897	12.399	8.713	138.106	29.788	52.582
AMK58_14485	no KO assigned | (GenBank) hypothetical protein	Hypothetical	16.868	24.261	20.969	201.184	25.070	116.557
AMK58_14490	no KO assigned | (GenBank) hypothetical protein	Hypothetical	43.487	45.539	58.284	358.120	60.516	209.436
AMK58_14495	no KO assigned | (GenBank) hypothetical protein	Hypothetical	70.829	85.999	83.461	148.941	53.152	145.015
AMK58_14500	no KO assigned | (GenBank) hypothetical protein	Hypothetical	40.168	52.021	62.847	429.601	172.456	323.018
AMK58_14505	no KO assigned | (GenBank) hypothetical protein	Hypothetical	12.531	17.065	14.105	264.879	36.457	142.345
AMK58_14510	K20276 large repetitive protein | (GenBank) hypothetical protein	Quorum sensing	3.982	5.692	6.125	40.480	7.976	25.661
AMK58_14515	no KO assigned | (GenBank) acyl-CoA dehydrogenase	dehydrogenase	26.004	29.208	32.736	316.290	48.170	216.245
AMK58_14520	K01644 citrate lyase subunit beta / citryl-CoA lyase [EC:4.1.3.34] | (GenBank) hypothetical protein	Signal transduction	22.001	20.930	29.179	478.110	60.328	277.166
AMK58_14525	K09709 3-methylfumaryl-CoA hydratase [EC:4.2.1.153] | (GenBank) acyl-CoA dehydrogenase	Metabolism	52.754	61.141	68.249	262.587	87.164	271.318
AMK58_14530	no KO assigned | (GenBank) carnitine dehydratase	dehydratase	531.828	595.453	644.841	907.802	149.219	734.787
AMK58_14535	no KO assigned | (GenBank) hypothetical protein	Hypothetical	96.860	105.331	88.258	207.427	86.431	437.458
AMK58_14540	K19338 LysR family transcriptional regulator, nitrogen assimilation regulatory protein | (GenBank) h	Nitrogen	11.218	15.407	11.769	119.178	11.015	25.706
AMK58_14545	K13483 xanthine dehydrogenase YagT iron-sulfur-binding subunit | (GenBank) ferredoxin	Metabolism: Nucleotide	46.908	35.956	55.372	309.196	39.395	164.465
AMK58_14550	K11178 xanthine dehydrogenase YagS FAD-binding subunit [EC:1.17.1.4] | (GenBank) molybdopterin de	Metabolism: Nucleotide	10.567	9.128	12.226	295.380	37.207	161.852
AMK58_14555	K11177 xanthine dehydrogenase YagR molybdenum-binding subunit [EC:1.17.1.4] | (GenBank) xanthine d	Metabolism: Nucleotide	3.699	5.937	4.619	13.830	15.887	50.017
AMK58_14560	no KO assigned | (GenBank) hypothetical protein	Hypothetical	20.806	24.711	25.860	153.120	113.693	170.032
AMK58_14565	K07323 phospholipid transport system substrate-binding protein | (GenBank) hypothetical protein	Transport: phospholipid	0.952	3.412	3.258	103.789	216.123	135.041
AMK58_14570	no KO assigned | (GenBank) sulfotransferase	No COG	0.553	0.661	2.418	103.236	89.983	68.490
AMK58_14575	no KO assigned | (GenBank) hypothetical protein	Hypothetical	5.438	10.260	13.880	460.102	311.599	316.903
AMK58_14580	K07003 uncharacterized protein | (GenBank) hypothetical protein	Hypothetical	1.088	2.438	2.673	105.147	33.947	45.749
AMK58_14585	K06045 squalene-hopene/tetraprenyl-beta-curcumene cyclase [EC:5.4.99.17 4.2.1.129] | (GenBank) squal	Metabolism	0.501	1.498	2.026	53.055	12.468	34.634
AMK58_14590	no KO assigned | (GenBank) hypothetical protein	Hypothetical	20.949	47.856	32.807	526.176	194.617	477.646
AMK58_14595	no KO assigned | (GenBank) hypothetical protein	Hypothetical	1.552	2.061	8.199	202.617	37.610	145.055
AMK58_14600	no KO assigned | (GenBank) radical SAM protein	No COG	21.252	19.238	32.352	360.892	116.548	279.977
AMK58_14605	K06952 uncharacterized protein | (GenBank) hydrolase	hydrolase	26.455	18.459	51.042	676.421	119.597	346.836
AMK58_14610	K03413 two-component system, chemotaxis family, response regulator CheY | (GenBank) regulator	Chemotaxis	4.175	3.326	12.173	165.541	64.136	108.914
AMK58_14615	no KO assigned | (GenBank) hypothetical protein	Hypothetical	1.527	1.303	3.319	87.960	22.437	32.366
AMK58_14620	no KO assigned | (GenBank) hypothetical protein	Hypothetical	12.990	7.702	22.156	107.593	37.897	183.438
AMK58_14625	no KO assigned | (GenBank) exopolysaccharide biosynthesis protein	Biosynthesis: polysacchharide	40.533	42.796	42.028	120.060	88.961	259.046
AMK58_14630	K03699 putative hemolysin | (GenBank) hypothetical protein	Defense	31.513	33.030	34.781	25.303	21.232	87.235
AMK58_14635	no KO assigned | (GenBank) hypothetical protein	Hypothetical	48.815	56.922	58.425	163.190	222.851	124.380
AMK58_14640	no KO assigned | (GenBank) hypothetical protein	Hypothetical	19.951	23.839	27.316	298.257	183.636	130.347
AMK58_14645	no KO assigned | (GenBank) hypothetical protein	Hypothetical	102.184	121.414	140.157	287.564	121.937	223.743
AMK58_14650	no KO assigned | (GenBank) hypothetical protein	Hypothetical	1227.347	1260.039	1316.441	469.118	399.134	738.807
AMK58_14655	no KO assigned | (GenBank) hypothetical protein	Hypothetical	16.078	18.427	16.971	58.106	64.174	151.701
AMK58_14660	K05837 rod shape determining protein RodA | (GenBank) rod shape-determining protein RodA	Cell Cycle/Shape/Homeostatsis	13.109	14.183	11.875	99.828	50.005	234.589
AMK58_14665	no KO assigned | (GenBank) hypothetical protein	Hypothetical	10.234	9.229	12.485	77.342	26.698	187.832
AMK58_14670	no KO assigned | (GenBank) hypothetical protein	Hypothetical	6.974	8.334	6.897	14.450	10.191	47.900
AMK58_14675	K07216 hemerythrin | (GenBank) hypothetical protein	hemerythrin	45.835	41.335	50.707	630.980	295.517	815.024
AMK58_14680	K01586 diaminopimelate decarboxylase [EC:4.1.1.20] | (GenBank) diaminopimelate decarboxylase	Biosynthesis: Amino Acid	77.465	55.994	72.927	116.067	90.084	199.975
AMK58_14685	no KO assigned | (GenBank) hypothetical protein	Hypothetical	97.641	120.559	147.922	325.244	413.401	1185.471
AMK58_14690	K01755 argininosuccinate lyase [EC:4.3.2.1] | (GenBank) argininosuccinate lyase	Metabolism: Amino Acid	134.528	150.130	155.584	321.538	369.477	167.345
AMK58_14695	no KO assigned | (GenBank) thiol:disulfide interchange protein	Cell Cycle/Shape/Homeostatsis	207.583	179.723	185.647	132.402	43.054	131.674
AMK58_14700	K08995 putative membrane protein | (GenBank) hypothetical protein	Membrane	327.125	316.241	318.168	301.280	70.668	293.932
AMK58_14705	K00074 3-hydroxybutyryl-CoA dehydrogenase [EC:1.1.1.157] | (GenBank) 3-hydroxybutyryl-CoA dehyd	Metabolism	13.223	6.583	10.477	14.761	3.871	11.623
AMK58_14710	K03522 electron transfer flavoprotein alpha subunit | (GenBank) electron transfer flavoprotein subun	Energy	307.651	295.813	296.195	235.593	231.315	222.148
AMK58_14715	K03521 electron transfer flavoprotein beta subunit | (GenBank) electron transfer flavoprotein subuni	Energy	90.213	85.101	98.114	247.413	364.516	159.085
AMK58_14720	K00798 cob(I)alamin adenosyltransferase [EC:2.5.1.17] | (GenBank) cob(I)yrinic acid a c-diamide aden	Metabolism: Co-Factors and Vitamens	21.241	43.148	34.536	254.945	169.520	142.405
AMK58_14725	no KO assigned | (GenBank) hypothetical protein	Hypothetical	6888.845	5746.040	5921.836	235.377	3132.935	645.982
AMK58_14730	K05350 beta-glucosidase [EC:3.2.1.21] | (GenBank) beta-glucosidase	Metabolism: Carbohydrates	263.290	236.126	206.165	133.084	124.447	47.341
AMK58_14735	no KO assigned | (GenBank) hypothetical protein	Hypothetical	309.413	319.884	352.776	142.363	176.619	83.579

AMK58_14740	no KO assigned | (GenBank) hypothetical protein	Hypothetical	128.033	138.574	127.028	597.167	398.372	311.860
AMK58_14745	no KO assigned | (GenBank) hypothetical protein	Hypothetical	27.529	27.146	30.114	16.111	100.643	33.210
AMK58_14750	no KO assigned | (GenBank) hypothetical protein	Hypothetical	596.405	532.842	539.590	1230.744	2222.295	1025.212
AMK58_14755	no KO assigned | (GenBank) hypothetical protein	Hypothetical	46.731	42.830	40.898	229.408	145.345	103.899
AMK58_14760	K10773 endonuclease III [EC:4.2.99.18] | (GenBank) DNA lyase	DNA Repair and Replication	22.712	25.051	19.270	21.531	31.618	57.512
AMK58_14765	no KO assigned | (GenBank) carboxymuconolactone decarboxylase	No COG	174.558	227.396	164.099	493.590	397.215	192.288
AMK58_14770	no KO assigned | (GenBank) hypothetical protein	Hypothetical	8.347	8.226	6.955	47.146	25.826	23.938
AMK58_14775	K01999 branched-chain amino acid transport system substrate-binding protein | (GenBank) branched-cha	Transport: Amino Acid	10.636	7.375	14.537	82.047	51.128	100.030
AMK58_14780	no KO assigned | (GenBank) hypothetical protein	Hypothetical	2.346	2.804	4.717	87.110	38.959	93.372
AMK58_14785	K16291 L,D-transpeptidase ErfK/SrfK | (GenBank) hypothetical protein	Cell Wall	238.256	406.258	475.035	76.313	18.963	97.228
AMK58_14790	K01997 branched-chain amino acid transport system permease protein | (GenBank) branched-chain amino	Transport: Amino Acid	122.327	193.726	225.342	353.640	54.750	208.796
AMK58_14795	K01998 branched-chain amino acid transport system permease protein | (GenBank) branched-chain amino	Transport: Amino Acid	250.588	208.239	197.563	150.227	55.674	201.859
AMK58_14800	K01995 branched-chain amino acid transport system ATP-binding protein | (GenBank) ABC transporter A'	Transport: Amino Acid	318.363	281.969	300.800	492.589	117.535	392.635
AMK58_14805	K03406 methyl-accepting chemotaxis protein | (GenBank) hypothetical protein	Chemotaxis	2.050	4.223	2.487	86.442	11.599	53.218
AMK58_14810	K07091 lipopolysaccharide export system permease protein | (GenBank) LPS export ABC transporter perm	Transport: polysacchharide	33.668	32.880	35.911	118.821	14.313	126.178
AMK58_14815	no KO assigned | (GenBank) pseudogene	Pseudogene	37.183	26.879	35.983	167.326	12.345	147.362
AMK58_14820	K06602 flagellar protein FlaF | (GenBank) hypothetical protein	Motility	44.072	36.981	42.610	174.458	33.675	206.861
AMK58_14825	no KO assigned | (GenBank) hypothetical protein	Hypothetical	314.373	415.746	390.747	154.889	42.533	141.057
AMK58_14830	K02396 flagellar hook-associated protein 1 FlgK | (GenBank) flagellar hook protein	Motility	132.003	118.760	124.364	133.368	44.687	145.384
AMK58_14835	K02390 flagellar hook protein FlgE | (GenBank) hypothetical protein	Motility	6.282	4.613	6.340	29.240	4.063	29.418
AMK58_14840	no KO assigned | (GenBank) hypothetical protein	Hypothetical	1987.950	2743.636	2620.409	472.633	2097.760	604.442
AMK58_14845	no KO assigned | (GenBank) hypothetical protein	Hypothetical	271.024	311.714	278.263	158.173	52.286	291.361
AMK58_14850	no KO assigned | (GenBank) hypothetical protein	Hypothetical	526.112	662.533	562.458	633.645	185.953	898.157
AMK58_14855	no KO assigned | (GenBank) histidine kinase	Signal Transduction	470.879	560.366	502.015	202.207	57.917	343.894
AMK58_14860	no KO assigned | (GenBank) bifunctional metallophosphatase/5'-nucleotidase	Metabolism: Nucleotide	9.234	15.219	11.429	40.520	3.579	32.404
AMK58_14865	K00375 GntR family transcriptional regulator / MocR family aminotransferase | (GenBank) GntR family	Transcription	16.613	34.589	24.492	132.514	41.120	64.187
AMK58_14870	no KO assigned | (GenBank) alkylhydroperoxidase	Defense	39.551	46.616	43.489	132.632	63.813	154.184
AMK58_14875	K19335 TetR/AcrR family transcriptional regulator, repressor for divergent bdcA | (GenBank) TetR fam	Transcription	130.155	127.793	129.125	769.302	296.910	696.633
AMK58_14880	K19336 cyclic-di-GMP-binding biofilm dispersal mediator protein | (GenBank) oxidoreductase	cyclic nucleotide	101.913	111.441	116.570	366.622	174.977	204.255
AMK58_14885	K04719 5,6-dimethylbenzimidazole synthase [EC:1.13.11.79] | (GenBank) 5,6-dimethylbenzimidazole synt	Metabolism: Co-Factors and Vitamins	21.830	26.304	31.745	496.879	51.757	188.137
AMK58_14890	K02224 cobyrinic acid a,c-diamide synthase [EC:6.3.5.9 6.3.5.11] | (GenBank) cobyrinic acid a,c-diam	Metabolism: Co-Factors and Vitamens	10.635	9.775	11.062	52.844	15.121	58.242
AMK58_14895	K02303 uroporphyrin-III C-methyltransferase [EC:2.1.1.107] | (GenBank) uroporphyrin-III methyltransf	Metabolism: Co-Factors and Vitamins	7.051	7.171	9.986	153.142	20.574	119.782
AMK58_14900	K02188 cobalt-precorrin-5B (C1)-methyltransferase [EC:2.1.1.195] | (GenBank) cobalt-precorrin-6A syn	Metabolism: Co-Factors and Vitamins	4.856	6.447	6.874	103.787	9.582	83.252
AMK58_14905	K05936 precorrin-4/cobalt-precorrin-4 C11-methyltransferase [EC:2.1.1.133 2.1.1.271] | (GenBank) pre	Metabolism: Co-Factors and Vitamens	19.067	17.087	17.676	103.867	19.289	132.160
AMK58_14910	K02189 cobalt-precorrin 5A hydrolase [EC:3.7.1.12] | (GenBank) cobalamin biosynthesis protein	Metabolism: Co-Factors and Vitamens	42.600	43.535	57.293	642.361	42.529	238.833
AMK58_14915	K00595 precorrin-6Y C5,15-methyltransferase (decarboxylating) [EC:2.1.1.132] | (GenBank) precorrin-6	Metabolism: Co-Factors and Vitamens	16.281	16.507	19.327	121.474	14.641	51.514
AMK58_14920	K05895 precorrin-6A/cobalt-precorrin-6A reductase [EC:1.3.1.54 1.3.1.106] | (GenBank) cobalt-precorr	Metabolism: Co-Factors and Vitamins	26.192	18.699	27.723	249.568	21.849	86.761
AMK58_14925	K05934 precorrin-3B C17-methyltransferase [EC:2.1.1.131] | (GenBank) precorrin-3B C17-methyltransfer	methyltransferase	11.554	10.401	14.747	248.473	10.140	84.679
AMK58_14930	K03394 precorrin-2/cobalt-factor-2 C20-methyltransferase [EC:2.1.1.130 2.1.1.151] | (GenBank) precor	Metabolism: Co-Factors and Vitamens	1.286	0.961	1.529	45.662	1.807	5.218
AMK58_14935	K06042 precorrin-8X/cobalt-precorrin-8 methylmutase [EC:5.4.99.61 5.4.99.60] | (GenBank) cobH; preco	Metabolism: Co-Factors and Vitamens	15.087	19.154	18.290	336.750	19.926	135.178
AMK58_14940	K02229 precorrin-3B synthase [EC:1.14.13.83] | (GenBank) precorrin-3B synthase	Metabolism: Co-Factors and Vitamins	18.534	24.836	18.692	67.287	13.921	42.964
AMK58_14945	K02230 cobaltochelatase CobN [EC:6.6.1.2] | (GenBank) cobalt chelatase	Metabolism: Co-Factors and Vitamens	3.621	3.817	3.375	31.096	3.032	16.029
AMK58_14950	K02234 cobalamin biosynthesis protein CobW | (GenBank) cobalamin biosynthesis protein CobW	Metabolism: Co-Factors and Vitamens	18.263	16.164	17.579	139.081	14.446	90.004
AMK58_14955	K01995 branched-chain amino acid transport system ATP-binding protein | (GenBank) ABC transporter A'	Transport: Amino Acid	34.502	35.931	38.975	148.829	23.305	53.567
AMK58_14960	K01996 branched-chain amino acid transport system ATP-binding protein | (GenBank) ABC transporter A'	Transport: Amino Acid	36.172	40.020	45.697	622.498	32.376	278.267
AMK58_14965	K01999 branched-chain amino acid transport system substrate-binding protein | (GenBank) ABC transpor	Transport: Amino Acid	17.311	17.437	20.670	488.310	51.700	201.395
AMK58_14970	K01997 branched-chain amino acid transport system permease protein | (GenBank) ABC transporter perme	Transport: Amino Acid	55.902	57.021	52.633	229.946	129.043	190.277
AMK58_14975	K01998 branched-chain amino acid transport system permease protein | (GenBank) ABC transporter perme	Transport: Amino Acid	77.404	92.343	90.626	104.345	55.982	140.366
AMK58_14980	K01907 acetoacetyl-CoA synthetase [EC:6.2.1.16] | (GenBank) acetoacetyl-CoA synthetase	Metabolism	50.754	43.474	48.315	55.946	32.236	71.104
AMK58_14985	K00019 3-hydroxybutyrate dehydrogenase [EC:1.1.1.30] | (GenBank) 3-hydroxybutyrate dehydrogenase	Lipid metabolism	756.178	643.289	812.863	210.883	245.176	365.805
AMK58_14990	K11209 GSH-dependent disulfide-bond oxidoreductase [EC:1.8.4.-] | (GenBank) glutathione S-transferas	transferase	264.398	187.537	271.563	131.689	109.534	284.832
AMK58_14995	no KO assigned | (GenBank) hypothetical protein	Hypothetical	653.299	467.167	658.336	514.433	369.295	1017.444
AMK58_15000	no KO assigned | (GenBank) hypothetical protein	Hypothetical	1326.847	1205.593	1428.211	2037.321	910.395	1771.708
AMK58_15005	no KO assigned | (GenBank) regulator	signal transduction	144.596	158.280	166.204	263.345	295.008	275.279
AMK58_15010	K06995 uncharacterized protein | (GenBank) hypothetical protein	Hypothetical	596.708	609.453	472.011	133.331	842.164	329.921
AMK58_15015	K06044 (1->4)-alpha-D-glucan 1-alpha-D-glucosylmutase [EC:5.4.99.15] | (GenBank) malto-oligosyltreha	Metabolism: Carbohydrate	0.787	0.522	0.790	9.723	108.029	21.367
AMK58_15020	no KO assigned | (GenBank) 4-alpha-glucanotransferase	transferase	0.344	0.385	0.327	4.949	34.766	7.708
AMK58_15025	no KO assigned | (GenBank) hypothetical protein	Hypothetical	81.101	128.597	110.368	448.457	2539.542	352.491
AMK58_15030	K14218 tRNA Ala | (GenBank) tRNA-Ala	tRNA synthesis/modification	60.537	76.143	151.472	1700.818	2114.817	1463.937
AMK58_15035	K14228 tRNA Leu | (GenBank) tRNA-Leu	tRNA synthesis/modification	175.350	99.773	348.003	5861.062	2931.479	5373.459
AMK58_15040	no KO assigned | (GenBank) hypothetical protein	Hypothetical	12.175	15.464	22.878	850.224	496.207	528.725
AMK58_15045	no KO assigned | (GenBank) hypothetical protein	Hypothetical	4.763	2.995	6.197	105.102	96.364	80.658
AMK58_15050	no KO assigned | (GenBank) hypothetical protein	Hypothetical	11.227	10.878	7.213	63.411	1.705	235.232
AMK58_15055	no KO assigned | (GenBank) hypothetical protein	Hypothetical	1.058	1.355	0.503	6.277	1.359	19.062
AMK58_15060	K01286 D-alanyl-D-alanine carboxypeptidase [EC:3.4.16.4] | (GenBank) hypothetical protein	Hydrolases	3.096	1.914	8.426	160.470	51.245	185.235
AMK58_15065	K07401 selenoprotein W-related protein | (GenBank) hypothetical protein	Hypothetical	43.311	94.095	54.752	45.491	94.048	182.096
AMK58_15070	no KO assigned | (GenBank) hypothetical protein	Hypothetical	31.355	33.386	45.162	306.906	115.156	243.827

AMK58_15075	K02012 iron(III) transport system substrate-binding protein | (GenBank) ABC transporter substrate-bi	Transport: Metal	29.298	29.525	30.766	129.560	97.077	140.964
AMK58_15080	K02011 iron(III) transport system permease protein | (GenBank) ABC transporter permease	Transport: Metal	94.441	80.225	86.198	155.130	145.933	113.026
AMK58_15085	K02010 iron(III) transport system ATP-binding protein [EC:3.6.3.30] | (GenBank) spermidine/putrescin	Transport: Metal	54.367	48.489	55.710	158.463	25.869	147.528
AMK58_15090	no KO assigned | (GenBank) hypothetical protein	Hypothetical	12.302	16.762	9.645	40.330	11.644	42.126
AMK58_15095	K03316 monovalent cation:H+ antiporter, CPA1 family | (GenBank) sodium:proton antiporter	Transport: Ion	1.455	5.217	3.690	36.258	7.852	31.036
AMK58_15100	K07112 uncharacterized protein | (GenBank) hypothetical protein	Hypothetical	54.352	47.860	49.750	109.618	62.120	158.438
AMK58_15105	no KO assigned | (GenBank) LysR family transcriptional regulator	Transcription	9.654	9.613	14.533	264.024	16.428	127.065
AMK58_15110	K00121 S-(hydroxymethyl)glutathione dehydrogenase / alcohol dehydrogenase [EC:1.1.1.284 1.1.1.1] | (	Metabolism	7.790	7.757	12.139	148.417	12.405	40.834
AMK58_15115	K01070 S-formylglutathione hydrolase [EC:3.1.2.12] | (GenBank) S-formylglutathione hydrolase	Metabolism	20.740	19.256	25.982	253.040	12.444	89.140
AMK58_15120	K07080 uncharacterized protein | (GenBank) immunogenic protein	No COG	5.520	7.329	10.847	292.822	27.026	113.293
AMK58_15125	no KO assigned | (GenBank) C4-dicarboxylate ABC transporter permease	Membrane Transport	13.589	3.136	6.044	140.155	24.518	92.855
AMK58_15130	no KO assigned | (GenBank) EBNA-1 nuclear protein	No COG	14.328	10.701	12.034	181.220	43.351	148.475
AMK58_15135	no KO assigned | (GenBank) NUDIX hydrolase	hydrolase	1076.599	898.251	1059.581	529.959	181.458	503.632
AMK58_15140	K00041 tagaturonate reductase [EC:1.1.1.58] | (GenBank) altronate oxidoreductase	Oxidoreductase	168.201	132.147	159.220	88.887	36.180	72.704
AMK58_15145	K01685 altronate hydrolase [EC:4.2.1.7] | (GenBank) altronate hydrolase	Metabolism: Carbohydrates	2767.714	2300.348	2492.000	114.446	192.158	146.599
AMK58_15150	no KO assigned | (GenBank) C4-dicarboxylate ABC transporter	Membrane Transport	457.425	388.626	416.685	261.533	89.768	359.703
AMK58_15155	no KO assigned | (GenBank) C4-dicarboxylate ABC transporter permease	Membrane Transport	412.837	344.016	388.862	409.080	63.568	416.184
AMK58_15160	no KO assigned | (GenBank) C4-dicarboxylate ABC transporter	Membrane Transport	355.774	419.917	461.893	69.366	284.888	147.851
AMK58_15165	no KO assigned | (GenBank) sorbitol dehydrogenase	dehydrogenase	31.564	33.829	37.113	258.852	94.114	294.694
AMK58_15170	no KO assigned | (GenBank) hypothetical protein	Hypothetical	36.165	32.358	33.556	406.284	67.556	324.335
AMK58_15175	no KO assigned | (GenBank) RND transporter	Transport	12.846	8.429	9.743	45.806	8.973	102.320
AMK58_15180	no KO assigned | (GenBank) hemolysin secretion protein D	Secretion system	19.016	18.734	14.330	33.602	15.235	81.506
AMK58_15185	no KO assigned | (GenBank) hypothetical protein	Hypothetical	319.744	227.797	268.823	112.903	110.779	309.103
AMK58_15190	no KO assigned | (GenBank) hypothetical protein	Hypothetical	25.411	24.112	23.877	211.643	51.413	239.786
AMK58_15195	no KO assigned | (GenBank) phosphoglycerate mutase	No COG	21.620	18.453	18.795	67.302	105.773	191.317
AMK58_15200	no KO assigned | (GenBank) hypothetical protein	Hypothetical	97.623	110.784	213.126	2510.228	512.495	2846.188
AMK58_15205	K05879 dihydroxyacetone kinase, C-terminal domain [EC:2.7.1.-] | (GenBank) Dak phosphatase	Metabolism: Lipid	20.081	22.348	45.860	549.586	110.202	310.339
AMK58_15210	K05878 dihydroxyacetone kinase, N-terminal domain [EC:2.7.1.-] | (GenBank) dihydroxyacetone kinase	Lipid metabolism	2.303	5.793	6.223	76.628	9.127	123.733
AMK58_15215	no KO assigned | (GenBank) transcriptional regulator	Transcription	6.151	8.964	10.128	157.563	21.923	253.476
AMK58_15220	K01714 4-hydroxy-tetrahydrodipicolinate synthase [EC:4.3.3.7] | (GenBank) dihydrodipicolinate syntha	Metabolism: Amino Acid	96.595	106.725	93.605	59.911	121.517	183.681
AMK58_15225	no KO assigned | (GenBank) hypothetical protein	Hypothetical	36.478	38.641	64.078	22.006	37.222	85.162
AMK58_15230	no KO assigned | (GenBank) hypothetical protein	Hypothetical	14.584	12.770	14.225	76.293	64.302	152.957
AMK58_15235	no KO assigned | (GenBank) hypothetical protein	Hypothetical	37.618	35.721	45.479	342.726	154.015	330.803
AMK58_15240	no KO assigned | (GenBank) C4-dicarboxylate ABC transporter permease	Membrane Transport	59.853	65.453	58.134	54.386	101.776	122.279
AMK58_15245	K13877 2,5-dioxopentanoate dehydrogenase [EC:1.2.1.26] | (GenBank) 2,5-dioxovalerate dehydrogenase	Metabolism: Carbohydrate	9.069	5.418	9.054	98.709	31.008	114.933
AMK58_15250	no KO assigned | (GenBank) pseudogene	Pseudogene	27.229	30.008	34.686	376.781	67.155	478.494
AMK58_15255	no KO assigned | (GenBank) transposase	Genetic Information Processing	62.985	66.941	62.679	179.381	41.207	164.117
AMK58_15260	no KO assigned | (GenBank) hypothetical protein	Hypothetical	45.739	37.890	43.489	90.361	20.306	145.416
AMK58_15265	no KO assigned | (GenBank) hypothetical protein	Hypothetical	296.888	216.175	281.402	871.205	260.715	1352.398
AMK58_15270	no KO assigned | (GenBank) hypothetical protein	Hypothetical	71.671	87.302	91.632	1002.476	139.222	741.690
AMK58_15275	no KO assigned | (GenBank) hypothetical protein	Hypothetical	18.627	28.664	17.711	239.309	46.951	255.158
AMK58_15280	K09780 uncharacterized protein | (GenBank) hypothetical protein	Hypothetical	18.944	19.683	31.716	412.636	230.988	902.358
AMK58_15285	no KO assigned | (GenBank) hypothetical protein	Hypothetical	24.593	18.837	24.583	312.546	200.596	957.216
AMK58_15290	no KO assigned | (GenBank) hypothetical protein	Hypothetical	17.575	19.445	22.281	173.521	66.583	436.403
AMK58_15295	no KO assigned | (GenBank) hypothetical protein	Hypothetical	0.545	5.431	2.247	46.269	11.239	147.164
AMK58_15300	no KO assigned | (GenBank) hypothetical protein	Hypothetical	33.192	24.317	29.052	22.884	23.971	106.103
AMK58_15305	no KO assigned | (GenBank) hypothetical protein	Hypothetical	60.981	63.592	60.300	68.617	30.658	247.980
AMK58_15310	no KO assigned | (GenBank) hypothetical protein	Hypothetical	1.022	1.526	3.400	87.936	8.326	117.007
AMK58_15315	no KO assigned | (GenBank) hypothetical protein	Hypothetical	0.922	4.133	5.482	211.923	37.588	316.066
AMK58_15320	no KO assigned | (GenBank) hypothetical protein	Hypothetical	22.691	18.627	30.962	290.032	133.757	568.737
AMK58_15325	no KO assigned | (GenBank) hypothetical protein	Hypothetical	3.401	4.064	7.761	245.709	29.818	155.790
AMK58_15330	no KO assigned | (GenBank) hypothetical protein	Hypothetical	0.897	8.037	8.527	405.465	25.202	416.229
AMK58_15335	no KO assigned | (GenBank) transposase	Genetic Information Processing	0.791	3.377	2.150	85.560	4.193	95.707
AMK58_15340	no KO assigned | (GenBank) hypothetical protein	Hypothetical	11.087	12.365	10.823	69.508	42.207	275.945
AMK58_15345	no KO assigned | (GenBank) transposase	Genetic Information Processing	0.813	0.000	2.404	57.690	10.656	78.530
AMK58_15350	K03427 type I restriction enzyme M protein [EC:2.1.1.72] | (GenBank) restriction endonuclease subuni	DNA Repair and Replication	0.417	0.415	0.462	23.635	4.607	26.344
AMK58_15355	no KO assigned | (GenBank) endonuclease	DNA Repair and Replication	1.423	0.000	1.722	18.367	6.252	23.869
AMK58_15360	K01154 type I restriction enzyme, S subunit [EC:3.1.21.3] | (GenBank) hypothetical protein	Defense	25.071	16.701	21.014	42.009	16.398	205.099
AMK58_15365	K01153 type I restriction enzyme, R subunit [EC:3.1.21.3] | (GenBank) type I restriction endonucleas	Defense	0.696	0.693	1.066	19.005	5.040	16.776
AMK58_15370	no KO assigned | (GenBank) hypothetical protein	Hypothetical	417.233	340.403	294.569	659.874	377.592	1863.456
AMK58_15375	no KO assigned | (GenBank) hypothetical protein	Hypothetical	30.269	45.729	23.156	98.104	35.588	311.323
AMK58_15380	no KO assigned | (GenBank) hypothetical protein	Hypothetical	78.184	67.135	59.591	146.497	25.973	643.679
AMK58_15385	no KO assigned | (GenBank) hypothetical protein	Hypothetical	322.368	333.398	326.881	1254.771	685.506	3251.200
AMK58_15390	no KO assigned | (GenBank) hypothetical protein	Hypothetical	17.296	12.157	11.931	78.594	37.359	268.567
AMK58_15395	no KO assigned | (GenBank) hypothetical protein	Hypothetical	10.258	10.047	4.797	59.468	22.493	252.919
AMK58_15400	no KO assigned | (GenBank) hypothetical protein	Hypothetical	6.081	6.606	8.805	186.625	27.498	114.705
AMK58_15405	no KO assigned | (GenBank) recombinase	DNA Repair and Replication	1.062	1.058	2.525	32.722	4.775	22.979

AMK58_15410	no KO assigned | (GenBank) hypothetical protein	Hypothetical	4.682	3.996	4.452	42.712	10.777	77.741
AMK58_15415	no KO assigned | (GenBank) hypothetical protein	Hypothetical	33.592	23.101	33.318	168.660	38.573	277.201
AMK58_15420	no KO assigned | (GenBank) hypothetical protein	Hypothetical	59.049	56.661	61.015	94.029	72.365	148.281
AMK58_15425	K14223 tRNA Gln | (GenBank) tRNA-Gln	tRNA synthesis/modification	514.971	704.066	681.504	733.080	1426.257	2529.810
AMK58_15430	no KO assigned | (GenBank) hypothetical protein	Hypothetical	17.239	17.567	18.419	20.621	3.759	128.201
AMK58_15435	no KO assigned | (GenBank) hypothetical protein	Hypothetical	11.638	11.369	14.979	27.624	7.213	76.652
AMK58_15440	no KO assigned | (GenBank) hypothetical protein	Hypothetical	48.295	63.495	55.257	240.936	138.210	504.340
AMK58_15445	K03924 MoxR-like ATPase [EC:3.6.3.-] | (GenBank) AAA family ATPase	Enzyme	5.013	7.488	6.674	27.703	18.456	60.435
AMK58_15450	K09986 uncharacterized protein | (GenBank) proteophosphoglycan precursor	No COG	127.836	181.786	158.515	830.177	270.049	442.613
AMK58_15455	no KO assigned | (GenBank) NUDIX hydrolase	hydrolase	82.126	117.927	103.054	740.342	283.478	246.274
AMK58_15460	no KO assigned | (GenBank) hypothetical protein	Hypothetical	140.067	167.837	155.749	529.489	391.379	216.468
AMK58_15465	K00970 poly(A) polymerase [EC:2.7.7.19] | (GenBank) tRNA nucleotidyltransferase	translation	97.329	103.337	109.916	134.173	212.982	123.143
AMK58_15470	K00228 coproporphyrinogen III oxidase [EC:1.3.3.3] | (GenBank) coproporphyrinogen III oxidase	Metabolism: Co-Factors and Vitamins	65.022	66.140	62.489	97.612	275.337	158.399
AMK58_15475	K03216 tRNA (cytidine/uridine-2'-O-)-methyltransferase [EC:2.1.1.207] | (GenBank) tRNA methyltransfe	tRNA synthesis/modification	390.045	416.616	422.103	334.295	1813.140	313.834
AMK58_15480	K00411 ubiquinol-cytochrome c reductase iron-sulfur subunit [EC:1.10.2.2] | (GenBank) ubiquinol-cyto	Energy	64.786	78.681	99.770	116.375	321.371	64.801
AMK58_15485	K00412 ubiquinol-cytochrome c reductase cytochrome b subunit | (GenBank) cytochrome B	Energy	17.078	26.221	24.493	157.201	311.974	87.232
AMK58_15490	K00413 ubiquinol-cytochrome c reductase cytochrome c1 subunit | (GenBank) cytochrome C	Energy	35.135	37.823	39.878	241.689	394.937	190.492
AMK58_15495	no KO assigned | (GenBank) hypothetical protein	Hypothetical	13.160	17.036	16.475	211.253	202.167	180.608
AMK58_15500	no KO assigned | (GenBank) hypothetical protein	Hypothetical	815.073	697.289	878.430	300.263	309.581	348.693
AMK58_15505	no KO assigned | (GenBank) hypothetical protein	Hypothetical	3359.003	2411.185	2657.968	307.152	636.782	694.686
AMK58_15510	no KO assigned | (GenBank) ArsR family transcriptional regulator	Transcription	1426.328	1167.416	1383.985	364.918	392.402	503.031
AMK58_15515	K04085 tRNA 2-thiouridine synthesizing protein A [EC:2.8.1.-] | (GenBank) SirA family protein	tRNA synthesis/modification	53.103	63.452	63.113	490.114	244.132	606.650
AMK58_15520	no KO assigned | (GenBank) acetoin utilization protein	No COG	3.515	2.333	6.437	98.183	44.779	126.152
AMK58_15525	K03602 exodeoxyribonuclease VII small subunit [EC:3.1.11.6] | (GenBank) exodeoxyribonuclease VII	DNA Repair and Replication	131.046	158.855	138.143	273.490	488.868	426.184
AMK58_15530	K00795 farnesyl diphosphate synthase [EC:2.5.1.1 2.5.1.10] | (GenBank) farnesyl-diphosphate synthase	Metabolism: terpenoids and polyketide	73.817	77.780	68.948	20.404	166.531	113.477
AMK58_15535	K01662 1-deoxy-D-xylulose-5-phosphate synthase [EC:2.2.1.7] | (GenBank) 1-deoxy-D-xylulose-5-phosp	Biosynthesis: Terepenoids	4.674	6.792	4.564	42.979	5.537	38.419
AMK58_15540	no KO assigned | (GenBank) hypothetical protein	Hypothetical	114.948	90.718	102.961	149.696	60.636	215.153
AMK58_15545	K06442 23S rRNA (cytidine1920-2'-O)/16S rRNA (cytidine1409-2'-O)-methyltransferase [EC:2.1.1.226 2	Ribosome	16.883	21.134	19.110	39.416	35.785	80.363
AMK58_15550	no KO assigned | (GenBank) pseudogene	Pseudogene	139.328	151.074	151.740	252.588	210.257	206.527
AMK58_15555	no KO assigned | (GenBank) LysR family transcriptional regulator	Transcription	299.290	334.224	315.849	448.018	488.378	348.048
AMK58_15560	no KO assigned | (GenBank) hypothetical protein	Hypothetical	1203.777	1519.047	1370.264	690.519	1016.163	701.175
AMK58_15565	no KO assigned | (GenBank) short-chain dehydrogenase	dehydrogenase	80.256	97.181	99.361	140.602	201.466	307.923
AMK58_15570	no KO assigned | (GenBank) MarR family transcriptional regulator	Transcription	5.364	9.156	6.072	88.621	153.607	398.628
AMK58_15575	no KO assigned | (GenBank) hydrolase	hydrolase	80.717	76.499	73.356	95.965	157.045	325.387
AMK58_15580	K00108 choline dehydrogenase [EC:1.1.99.1] | (GenBank) oxidoreductase	Metabolism: Amino Acid	3.076	3.137	1.926	12.561	23.947	47.579
AMK58_15585	no KO assigned | (GenBank) LysR family transcriptional regulator	Transcription	23.906	26.884	26.874	89.475	110.013	310.078
AMK58_15590	no KO assigned | (GenBank) ethyl tert-butyl ether degradation protein EthD	Metabolism	87.577	77.158	71.374	110.286	92.543	497.729
AMK58_15595	no KO assigned | (GenBank) dehydrogenase	dehydrogenase	8.805	9.645	9.349	102.619	32.827	205.079
AMK58_15600	no KO assigned | (GenBank) dehalogenase	enzyme	21.106	21.300	21.424	223.889	84.798	375.158
AMK58_15605	no KO assigned | (GenBank) glyoxalase	enzyme	27.362	32.694	30.352	131.948	116.871	477.833
AMK58_15610	no KO assigned | (GenBank) hypothetical protein	Hypothetical	8.171	4.438	12.006	68.324	49.817	215.356
AMK58_15615	K16137 TetR/AcrR family transcriptional regulator, transcriptional repressor for nem operon | (GenBa	Transcription	22.166	26.236	22.666	126.611	114.234	290.854
AMK58_15620	no KO assigned | (GenBank) polyketide cyclase	No COG	60.045	60.770	68.635	121.310	234.198	523.317
AMK58_15625	no KO assigned | (GenBank) arylesterase	esterase	31.374	25.560	33.220	77.941	75.180	202.588
AMK58_15630	no KO assigned | (GenBank) XRE family transcriptional regulator	Transcription	20.608	20.521	23.405	45.243	88.155	134.034
AMK58_15635	K06893 uncharacterized protein | (GenBank) hypothetical protein	Hypothetical	118.752	88.468	103.248	73.509	401.100	320.924
AMK58_15640	no KO assigned | (GenBank) thymidylate synthase	No COG	3.483	6.120	5.649	65.039	12.895	198.324
AMK58_15645	no KO assigned | (GenBank) AAA family ATPase	Transcription	2.565	2.617	3.094	44.507	8.440	108.904
AMK58_15650	no KO assigned | (GenBank) acetoin catabolism protein X	Metabolism	38.624	29.708	41.122	158.468	31.976	211.028
AMK58_15655	K00161 pyruvate dehydrogenase E1 component alpha subunit [EC:1.2.4.1] | (GenBank) ABC transporter s	Transport	15.503	12.497	23.047	36.261	28.353	110.216
AMK58_15660	K00162 pyruvate dehydrogenase E1 component beta subunit [EC:1.2.4.1] | (GenBank) pyruvate dehydroge	dehydrogenase	11.497	15.420	14.167	61.763	27.957	152.195
AMK58_15665	K00627 pyruvate dehydrogenase E2 component (dihydrolipoamide acetyltransferase) [EC:2.3.1.12] | (Gen	Metabolism: Carbohydrate	54.772	80.373	72.990	332.667	100.570	323.444
AMK58_15670	K03366 meso-butanediol dehydrogenase / (S,S)-butanediol dehydrogenase / diacetyl reductase [EC:1.1.1	Metabolism: Carbohydrate	174.218	285.293	129.765	150.012	32.205	118.126
AMK58_15675	K03644 lipoyl synthase [EC:2.8.1.8] | (GenBank) lipoyl synthase	Metabolism: Lipid	1136.758	1322.212	646.698	253.703	23.423	149.571
AMK58_15680	no KO assigned | (GenBank) oxidoreductase	Oxidoreductase	887.101	966.472	498.319	128.584	50.001	143.490
AMK58_15685	K18335 2-keto-3-deoxy-L-fuconate dehydrogenase [EC:1.1.1.-] | (GenBank) NAD(P)-dependent oxidored	Energy	1035.808	1112.256	597.721	302.137	40.791	197.528
AMK58_15690	no KO assigned | (GenBank) C4-dicarboxylate ABC transporter	Membrane Transport	260.486	325.256	148.010	76.725	13.067	91.761
AMK58_15695	no KO assigned | (GenBank) C4-dicarboxylate ABC transporter	Membrane Transport	412.231	588.444	306.527	360.091	56.705	390.048
AMK58_15700	no KO assigned | (GenBank) C4-dicarboxylate ABC transporter	Membrane Transport	81.330	85.747	65.899	281.319	34.471	187.457
AMK58_15705	no KO assigned | (GenBank) GntR family transcriptional regulator	Transcription	61.755	32.218	49.951	193.411	30.895	171.953
AMK58_15710	K18910 D-psicose/D-tagatose/L-ribulose 3-epimerase [EC:5.1.3.30 5.1.3.31] | (GenBank) hypothetical p	Enzyme	267.249	147.398	195.797	107.933	55.463	319.089
AMK58_15715	no KO assigned | (GenBank) hypothetical protein	Hypothetical	187.673	105.649	136.950	35.150	34.785	98.120
AMK58_15720	no KO assigned | (GenBank) hypothetical protein	Hypothetical	462.908	263.153	333.234	54.627	54.840	190.341
AMK58_15725	K07068 uncharacterized protein | (GenBank) hypothetical protein	Hypothetical	214.441	208.729	187.569	348.982	150.312	621.672
AMK58_15730	no KO assigned | (GenBank) hypothetical protein	Hypothetical	121.390	129.978	100.729	412.645	122.626	422.473
AMK58_15735	no KO assigned | (GenBank) acyl dehydratase	dehydratase	49.695	75.574	72.450	512.782	33.177	179.017
AMK58_15740	no KO assigned | (GenBank) hypothetical protein	Hypothetical	32.903	29.050	38.219	258.586	18.765	137.820

AMK58_15745	K01999 branched-chain amino acid transport system substrate-binding protein | (GenBank) hypothetical	Transport: Amino Acid	6.867	8.837	11.150	84.490	7.482	39.774
AMK58_15750	K01997 branched-chain amino acid transport system permease protein | (GenBank) branched-chain amino	Transport: Amino Acid	14.854	20.930	13.857	99.301	8.033	73.157
AMK58_15755	K01995 branched-chain amino acid transport system ATP-binding protein K01998 branched-chain amino a	Transport: Amino Acid	8.740	8.490	8.715	30.172	3.913	19.700
AMK58_15760	K01996 branched-chain amino acid transport system ATP-binding protein | (GenBank) ABC transporter A	Transport: Amino Acid	18.120	20.667	21.614	163.069	6.851	79.582
AMK58_15765	no KO assigned | (GenBank) hypothetical protein	Hypothetical	99.656	105.068	108.795	173.059	36.748	235.306
AMK58_15770	K02182 crotonobetaine/carnitine-CoA ligase [EC:6.2.1.-] | (GenBank) hypothetical protein	Enzyme	28.953	32.761	38.234	110.181	15.943	105.897
AMK58_15775	no KO assigned | (GenBank) hypothetical protein	Hypothetical	238.306	249.730	289.168	1746.716	173.357	1009.536
AMK58_15780	no KO assigned | (GenBank) FAD-dependent oxidoreductase	Oxidoreductase	13.350	19.633	16.599	78.548	21.008	108.937
AMK58_15785	no KO assigned | (GenBank) hypothetical protein	Hypothetical	278.145	295.424	266.882	381.356	121.380	490.548
AMK58_15790	K07638 two-component system, OmpR family, osmolarity sensor histidine kinase EnvZ [EC:2.7.13.3] | (G	Signal Transduction	50.380	58.239	62.020	276.534	131.690	230.791
AMK58_15795	K07659 two-component system, OmpR family, phosphate regulon response regulator OmpR | (GenBank) t	Signal transduction	23.384	25.871	23.386	66.359	216.503	106.166
AMK58_15800	no KO assigned | (GenBank) hypothetical protein	Hypothetical	60.876	71.056	67.260	77.771	50.053	95.858
AMK58_15805	K03704 cold shock protein (beta-ribbon, CspA family) | (GenBank) cold-shock protein	Stress Response	15.207	34.945	18.352	314.186	54.569	118.236
AMK58_15810	no KO assigned | (GenBank) MarR family transcriptional regulator	Transcription	129.099	163.566	164.712	482.132	184.110	363.290
AMK58_15815	K00826 branched-chain amino acid aminotransferase [EC:2.6.1.42] | (GenBank) branched-chain amino aci	Metabolism	52.840	77.887	81.229	219.592	86.364	123.587
AMK58_15820	no KO assigned | (GenBank) hypothetical protein	Hypothetical	614.849	884.800	1084.787	1357.660	471.790	795.976
AMK58_15825	no KO assigned | (GenBank) addiction module toxin RelE	defense	1043.277	1141.041	1123.392	60.873	3517.348	383.367
AMK58_15830	K03702 excinuclease ABC subunit B | (GenBank) excinuclease ABC subunit B	DNA Repair and Replication	31.132	32.465	35.144	22.065	145.128	43.827
AMK58_15835	no KO assigned | (GenBank) hypothetical protein	Hypothetical	365.915	434.013	390.125	356.809	1763.667	668.628
AMK58_15840	no KO assigned | (GenBank) hypothetical protein	Hypothetical	53.594	58.335	55.806	41.418	148.654	124.744
AMK58_15845	no KO assigned | (GenBank) MFS transporter	Transport: MFS	60.859	49.981	62.236	192.727	488.055	388.298
AMK58_15850	no KO assigned | (GenBank) histidine kinase	Signal Transduction	26.097	26.662	29.702	20.923	74.153	61.802
AMK58_15855	K02339 DNA polymerase III subunit chi [EC:2.7.7.7] | (GenBank) DNA polymerase III subunit chi	DNA Repair and Replication	904.025	1043.239	1043.745	388.886	345.072	772.665
AMK58_15860	K01255 leucyl aminopeptidase [EC:3.4.11.1] | (GenBank) aminopeptidase	Peptidase	19.494	21.657	29.795	224.125	109.019	186.892
AMK58_15865	K11720 lipopolysaccharide export system permease protein | (GenBank) LPS export ABC transporter perm	Transport: polysacchharide	130.584	165.810	147.920	189.883	96.943	285.442
AMK58_15870	K03771 peptidyl-prolyl cis-trans isomerase SurA [EC:5.2.1.8] | (GenBank) peptidylprolyl isomerase	Chaperone	34.725	34.661	30.139	69.154	41.301	94.961
AMK58_15875	K00097 4-hydroxythreonine-4-phosphate dehydrogenase [EC:1.1.1.262] | (GenBank) 4-hydroxythreonine-	Metabolism: Co-Factors and Vitamens	331.926	328.990	316.269	318.540	628.753	258.580
AMK58_15880	no KO assigned | (GenBank) histidine kinase	Signal Transduction	31.587	30.569	28.246	68.749	52.759	116.240
AMK58_15885	no KO assigned | (GenBank) hypothetical protein	Hypothetical	285.184	205.151	226.846	432.794	259.655	241.700
AMK58_15890	no KO assigned | (GenBank) murein transglycosylase	transglycosylase	61.135	73.229	80.868	199.829	148.862	73.599
AMK58_15895	no KO assigned | (GenBank) alpha/beta hydrolase	hydrolase	424.048	526.699	532.867	739.805	1003.647	323.538
AMK58_15900	no KO assigned | (GenBank) hypothetical protein	Hypothetical	22.592	19.656	24.400	273.836	29.339	134.032
AMK58_15905	K02528 16S rRNA (adenine1518-N6/adenine1519-N6)-dimethyltransferase [EC:2.1.1.182] | (GenBank) 1	Ribosome	38.932	51.973	59.661	185.492	195.083	130.651
AMK58_15910	K00942 guanylate kinase [EC:2.7.4.8] | (GenBank) guanylate kinase	Metabolism: Nucleotide	119.393	145.118	169.658	267.306	209.180	290.550
AMK58_15915	no KO assigned | (GenBank) hypothetical protein	Hypothetical	57.116	82.598	70.638	134.126	101.717	156.944
AMK58_15920	no KO assigned | (GenBank) short-chain dehydrogenase	dehydrogenase	59.425	72.146	68.739	285.422	139.487	227.191
AMK58_15925	K00570 phosphatidylethanolamine/phosphatidyl-N-methylethanolamine N-methyltransferase [EC:2.1.1.17	Metabolism: Lipid	81.898	104.681	100.706	210.710	115.292	250.279
AMK58_15930	no KO assigned | (GenBank) hypothetical protein	Hypothetical	85.546	87.586	90.193	257.372	136.093	154.811
AMK58_15935	K06158 ATP-binding cassette, subfamily F, member 3 | (GenBank) glycosyl transferase family 1	Glycosyl Transferase	12.325	9.538	11.597	47.646	28.425	42.397
AMK58_15940	K00940 nucleoside-diphosphate kinase [EC:2.7.4.6] | (GenBank) ndk; phosphodiesterase	Metabolism: Nucleotide	398.431	417.255	354.882	126.145	388.654	388.299
AMK58_15945	no KO assigned | (GenBank) hypothetical protein	Hypothetical	60.537	66.308	87.538	358.160	135.810	251.905
AMK58_15950	K11175 phosphoribosylglycinamide formyltransferase 1 [EC:2.1.2.2] | (GenBank) phosphoribosylglycinan	Metabolism	264.953	270.312	270.160	375.767	532.371	416.522
AMK58_15955	K01933 phosphoribosylformylglycinamidine cyclo-ligase [EC:6.3.3.1] | (GenBank) phosphoribosylaminoi	Metabolism: Nucleotide	762.447	620.496	712.292	34.444	198.673	114.965
AMK58_15960	no KO assigned | (GenBank) hypothetical protein	Hypothetical	85.367	142.389	126.410	40.491	166.990	80.237
AMK58_15965	K08744 cardiolipin synthase (CMP-forming) [EC:2.7.8.41] | (GenBank) CDP-alcohol phosphatidyltransfe	Metabolism: Lipid	131.650	143.124	162.316	313.386	96.308	140.287
AMK58_15970	no KO assigned | (GenBank) hypothetical protein	Hypothetical	330.733	262.233	351.102	167.378	140.578	107.195
AMK58_15975	no KO assigned | (GenBank) DNA replication protein	DNA Repair and Replication	227.114	225.044	280.048	273.914	183.770	195.041
AMK58_15980	K00059 3-oxoacyl-[acyl-carrier protein] reductase [EC:1.1.1.100] | (GenBank) 3-oxoacyl-ACP reductase	Metabolism: Lipid	75.284	69.553	79.845	167.592	68.221	138.754
AMK58_15985	no KO assigned | (GenBank) metal-chelation protein CHAD	No COG	55.718	52.182	64.647	102.375	49.596	147.249
AMK58_15990	K08296 phosphohistidine phosphatase [EC:3.1.3.-] | (GenBank) phosphohistidine phosphatase	Hydrolases	46.657	44.600	59.223	312.497	83.124	89.772
AMK58_15995	K00937 polyphosphate kinase [EC:2.7.4.1] | (GenBank) polyphosphate kinase	Signal Transduction	32.862	45.462	40.624	71.730	34.720	49.601
AMK58_16000	K01524 exopolyphosphatase / guanosine-5'-triphosphate,3'-diphosphate pyrophosphatase [EC:3.6.1.11 3.	Metabolism: Nucleotide	20.336	22.048	23.666	150.616	42.893	77.686
AMK58_16005	no KO assigned | (GenBank) hypothetical protein	Hypothetical	9.778	13.173	13.034	118.397	25.107	54.935
AMK58_16010	K03684 ribonuclease D [EC:3.1.13.5] | (GenBank) ribonuclease D	post translation modificaton	311.972	330.996	317.933	279.343	225.423	397.225
AMK58_16015	K01876 aspartyl-tRNA synthetase [EC:6.1.1.12] | (GenBank) aspartate--tRNA(Asp/Asn) ligase	tRNA synthesis/modification	103.476	137.951	131.789	131.078	154.908	123.619
AMK58_16020	no KO assigned | (GenBank) hypothetical protein	Hypothetical	4192.480	4397.240	3796.905	398.351	576.605	1291.594
AMK58_16025	no KO assigned | (GenBank) hypothetical protein	Hypothetical	313.354	318.244	300.911	183.847	98.824	246.464
AMK58_16030	no KO assigned | (GenBank) D-glycerate dehydrogenase	dehydrogenase	1006.503	849.411	848.171	220.356	469.087	403.466
AMK58_16035	no KO assigned | (GenBank) pseudogene	Pseudogene	259.899	308.026	293.925	59.440	179.558	168.894
AMK58_16040	K07716 two-component system, cell cycle sensor histidine kinase PleC [EC:2.7.13.3] | (GenBank) hypot	Signal Transduction	175.751	178.136	158.803	46.580	186.940	110.300
AMK58_16045	K11443 two-component system, cell cycle response regulator DivK | (GenBank) two-component system re	Cell Cycle/Shape/Homeostatsis	57.334	45.927	51.164	425.202	45.004	513.233
AMK58_16050	K02488 two-component system, cell cycle response regulator [EC:2.7.7.65] | (GenBank) pleD; response	Cell Cycle/Shape/Homeostatsis	50.231	70.887	65.382	72.392	9.053	83.841
AMK58_16055	K02913 large subunit ribosomal protein L33 | (GenBank) 50S ribosomal protein L33	Ribosome	188.819	284.175	241.887	1757.979	415.570	2080.999
AMK58_16060	K03797 carboxyl-terminal processing protease [EC:3.4.21.102] | (GenBank) peptidase S41	Peptidase	47.098	46.897	41.313	16.569	23.083	73.523
AMK58_16065	K12573 ribonuclease R [EC:3.1.-.-] | (GenBank) RNAse R	Genetic Information Processing	78.454	86.451	77.246	51.947	77.835	139.251
AMK58_16070	no KO assigned | (GenBank) hypothetical protein	Hypothetical	533.204	578.684	479.437	64.874	594.540	190.638
AMK58_16075	no KO assigned | (GenBank) pseudogene	Pseudogene	608.862	638.021	681.081	401.571	104.357	273.821

AMK58_16080	no KO assigned | (GenBank) pseudogene	Pseudogene	68.246	62.312	79.764	713.436	142.210	734.588
AMK58_16085	K03723 transcription-repair coupling factor (superfamily II helicase) [EC:3.6.4.-] | (GenBank) hypot	Transcription	5.309	5.008	7.553	16.951	10.141	24.579
AMK58_16090	no KO assigned | (GenBank) hypothetical protein	Hypothetical	3.622	7.728	6.395	83.865	35.477	114.069
AMK58_16095	no KO assigned | (GenBank) hypothetical protein	Hypothetical	4.646	6.939	9.386	249.696	84.528	133.539
AMK58_16100	no KO assigned | (GenBank) hypothetical protein	Hypothetical	12.726	19.550	40.793	1698.812	519.851	1352.630
AMK58_16105	no KO assigned | (GenBank) hypothetical protein	Hypothetical	38.604	38.439	47.827	564.125	467.451	1191.577
AMK58_16110	no KO assigned | (GenBank) polyketide cyclase	No COG	4.853	10.682	9.229	83.146	150.736	229.276
AMK58_16115	no KO assigned | (GenBank) hypothetical protein	Hypothetical	7.088	3.457	4.676	59.681	13.658	97.649
AMK58_16120	no KO assigned | (GenBank) hypothetical protein	Hypothetical	3.722	3.511	4.284	10.760	4.514	19.166
AMK58_16125	no KO assigned | (GenBank) hypothetical protein	Hypothetical	11.822	13.395	11.241	21.934	8.554	53.804
AMK58_16130	no KO assigned | (GenBank) hypothetical protein	Hypothetical	64.780	56.879	60.019	228.169	55.600	299.770
AMK58_16135	K01999 branched-chain amino acid transport system substrate-binding protein | (GenBank) ABC transpor	Transport: Amino Acid	24.904	27.640	27.610	187.635	39.168	142.208
AMK58_16140	no KO assigned | (GenBank) N-methylproline demethylase	No COG	7.882	7.081	8.847	130.908	18.319	54.019
AMK58_16145	K00111 glycerol-3-phosphate dehydrogenase [EC:1.1.5.3] | (GenBank) hypothetical protein	Lipid metabolism	6.624	5.062	7.836	61.078	13.073	43.565
AMK58_16150	no KO assigned | (GenBank) transcriptional regulator	Transcription	34.764	40.584	37.866	160.045	57.192	397.452
AMK58_16155	no KO assigned | (GenBank) hypothetical protein	Hypothetical	16.320	11.338	20.329	208.452	130.263	459.754
AMK58_16160	no KO assigned | (GenBank) hypothetical protein	Hypothetical	31.710	32.532	24.668	190.504	75.607	439.723
AMK58_16165	no KO assigned | (GenBank) hypothetical protein	Hypothetical	10.312	13.691	9.321	41.783	44.792	184.873
AMK58_16170	no KO assigned | (GenBank) hypothetical protein	Hypothetical	22.937	19.469	22.166	89.823	45.505	233.256
AMK58_16175	K02034 peptide/nickel transport system permease protein | (GenBank) hypothetical protein	Quorum sensing	31.854	40.473	29.328	98.663	51.683	180.834
AMK58_16180	K02033 peptide/nickel transport system permease protein | (GenBank) hypothetical protein	Quorum sensing	26.315	29.478	24.299	53.892	47.355	185.943
AMK58_16185	K02035 peptide/nickel transport system substrate-binding protein | (GenBank) hypothetical protein	Quorum sensing	32.181	23.639	25.415	33.803	80.351	164.613
AMK58_16190	no KO assigned | (GenBank) hypothetical protein	Hypothetical	44.285	39.438	33.527	28.430	133.088	185.721
AMK58_16195	no KO assigned | (GenBank) integrase	Genetic Information Processing	177.816	145.626	135.635	353.276	362.915	927.429
AMK58_16200	K03088 RNA polymerase sigma-70 factor, ECF subfamily | (GenBank) hypothetical protein	Transcription	190.473	121.894	150.943	175.552	174.260	416.213
AMK58_16205	no KO assigned | (GenBank) hypothetical protein	Hypothetical	85.665	79.449	70.571	128.423	124.274	457.968
AMK58_16210	no KO assigned | (GenBank) hypothetical protein	Hypothetical	56.502	70.917	66.814	155.826	409.881	425.744
AMK58_16215	no KO assigned | (GenBank) hypothetical protein	Hypothetical	36.948	54.337	42.697	399.754	1548.779	741.085
AMK58_16220	K02277 cytochrome c oxidase subunit IV [EC:1.9.3.1] | (GenBank) hypothetical protein	Energy	4.535	5.418	6.467	447.074	202.866	501.219
AMK58_16225	K02276 cytochrome c oxidase subunit III [EC:1.9.3.1] | (GenBank) hypothetical protein	Energy	4.484	3.494	6.117	89.842	32.654	133.603
AMK58_16230	K02274 cytochrome c oxidase subunit I [EC:1.9.3.1] | (GenBank) cytochrome C oxidase subunit I	Energy	0.587	0.438	1.186	26.540	5.609	30.483
AMK58_16235	K02275 cytochrome c oxidase subunit II [EC:1.9.3.1] | (GenBank) cytochrome B	Energy	0.511	0.763	0.243	12.831	4.881	31.976
AMK58_16240	K07152 protein SCO1/2 | (GenBank) hypothetical protein	energy	2.242	2.870	3.350	74.228	22.142	70.540
AMK58_16245	no KO assigned | (GenBank) hypothetical protein	Hypothetical	13.057	31.913	23.137	399.104	175.787	768.277
AMK58_16250	no KO assigned | (GenBank) hypothetical protein	Hypothetical	2.640	6.009	4.304	90.440	39.853	192.246
AMK58_16255	no KO assigned | (GenBank) hypothetical protein	Hypothetical	1.824	0.000	5.203	218.080	20.504	102.967
AMK58_16260	no KO assigned | (GenBank) hypothetical protein	Hypothetical	0.897	0.000	1.919	130.950	12.349	65.491
AMK58_16265	K00185 prokaryotic molybdopterin-containing oxidoreductase family, membrane subunit | (GenBank) hydr	Oxidoreductase	0.450	2.691	1.884	134.431	13.366	79.524
AMK58_16270	K00184 prokaryotic molybdopterin-containing oxidoreductase family, iron-sulfur binding subunit | (Ge	oxidoreductase	0.126	0.251	0.199	16.578	2.688	12.876
AMK58_16275	no KO assigned | (GenBank) cytochrome C	Energy	0.553	3.303	2.103	165.889	16.779	109.877
AMK58_16280	no KO assigned | (GenBank) hypothetical protein	Hypothetical	8.142	6.345	12.118	408.310	109.034	619.453
AMK58_16285	K02051 NitT/TauT family transport system substrate-binding protein | (GenBank) sulfonate ABC transpo	Transport: NitT/TauT	6.154	9.192	11.253	536.498	68.379	271.401
AMK58_16290	K02049 NitT/TauT family transport system ATP-binding protein | (GenBank) ABC transporter	Transport: NitT/TauT	0.285	0.000	3.254	195.043	37.670	187.248
AMK58_16295	K02050 NitT/TauT family transport system permease protein | (GenBank) ABC transporter permease	Transport: NitT/TauT	0.540	0.806	1.797	94.202	4.703	71.217
AMK58_16300	K01733 threonine synthase [EC:4.2.3.1] | (GenBank) threonine synthase	Metabolism	4.484	4.659	3.800	63.464	27.065	161.535
AMK58_16305	K13574 uncharacterized oxidoreductase [EC:1.1.1.-] | (GenBank) malate dehydrogenase	Oxidoreductase	2.035	1.351	3.977	100.704	18.044	135.856
AMK58_16310	K00058 D-3-phosphoglycerate dehydrogenase [EC:1.1.1.95] | (GenBank) 3-phosphoglycerate dehydrogen	Metabolism	2.515	0.715	3.985	114.380	24.903	174.457
AMK58_16315	no KO assigned | (GenBank) hypothetical protein	Hypothetical	6.457	3.553	8.482	145.569	52.145	118.888
AMK58_16320	K01066 acetyl esterase [EC:3.1.1.-] | (GenBank) acetyl esterase	Hydrolase	6.809	8.136	9.417	274.456	44.669	123.520
AMK58_16325	K14228 tRNA Leu | (GenBank) tRNA-Leu	tRNA synthesis/modification	436.983	324.263	411.517	2160.614	376.994	1649.203
AMK58_16330	no KO assigned | (GenBank) hypothetical protein	Hypothetical	8.284	4.013	7.025	121.696	14.345	164.070
AMK58_16335	no KO assigned | (GenBank) hypothetical protein	Hypothetical	30.078	31.846	30.047	708.416	128.818	673.265
AMK58_16340	no KO assigned | (GenBank) hypothetical protein	Hypothetical	141.785	161.803	129.761	588.991	103.264	368.816
AMK58_16345	no KO assigned | (GenBank) hypothetical protein	Hypothetical	38.019	31.450	65.623	2573.036	402.362	755.515
AMK58_16350	no KO assigned | (GenBank) hypothetical protein	Hypothetical	5.954	3.953	18.872	435.154	44.621	280.367
AMK58_16355	no KO assigned | (GenBank) hypothetical protein	Hypothetical	0.777	1.092	0.608	10.408	4.007	16.826
AMK58_16360	no KO assigned | (GenBank) hypothetical protein	Hypothetical	41.931	53.234	37.376	248.545	37.116	389.785
AMK58_16365	no KO assigned | (GenBank) MFS transporter	Transport: MFS	21.230	25.744	20.586	26.468	14.058	46.292
AMK58_16370	K00432 glutathione peroxidase [EC:1.11.1.9] | (GenBank) glutathione peroxidase	Metabolism	78.258	63.645	96.614	578.216	201.492	484.032
AMK58_16375	no KO assigned | (GenBank) hypothetical protein	Hypothetical	44.445	47.308	39.830	65.696	22.969	44.529
AMK58_16380	K05539 tRNA-dihydrouridine synthase A [EC:1.-.-.-] | (GenBank) tRNA-dihydrouridine synthase A	tRNA synthesis/modification	51.365	35.072	50.466	268.999	47.151	226.581
AMK58_16385	K19337 RpiR family transcriptional regulator, carbohydrate utilization regulator | (GenBank) transcr	Transcription	31.876	45.772	38.503	182.161	113.642	119.501
AMK58_16390	no KO assigned | (GenBank) C4-dicarboxylate ABC transporter permease	Membrane Transport	22.870	42.866	40.505	86.499	92.493	71.936
AMK58_16395	no KO assigned | (GenBank) L-dehydroascorbate transporter large permease subunit	Transport	14.650	12.988	15.098	153.323	64.488	90.885
AMK58_16400	no KO assigned | (GenBank) ABC transporter substrate-binding protein	Membrane Transport	5.615	11.182	12.124	205.279	52.728	169.110
AMK58_16405	no KO assigned | (GenBank) hypothetical protein	Hypothetical	13.611	7.961	15.204	126.485	31.883	70.257
AMK58_16410	no KO assigned | (GenBank) 2-hydroxyhepta-2,4-diene-1,7-dioate isomerase	No COG	56.416	55.186	64.045	262.505	161.651	214.788

AMK58_16415	no KO assigned | (GenBank) hypothetical protein	Hypothetical	540.748	553.154	482.045	314.125	368.549	373.009
AMK58_16420	no KO assigned | (GenBank) hypothetical protein	Hypothetical	241.007	297.986	278.023	871.622	542.233	462.142
AMK58_16425	K03168 DNA topoisomerase I [EC:5.99.1.2] | (GenBank) DNA topoisomerase I	DNA Repair and Replication	72.603	69.685	72.969	58.364	107.782	49.571
AMK58_16430	K04096 DNA processing protein | (GenBank) DNA processing protein DprA	DNA Repair and Replication	43.596	47.708	58.791	36.138	99.826	49.331
AMK58_16435	no KO assigned | (GenBank) hypothetical protein	Hypothetical	9.037	8.589	14.549	68.368	116.470	53.040
AMK58_16440	no KO assigned | (GenBank) regulator	Signal transduction	371.519	333.572	357.128	526.524	997.460	512.446
AMK58_16445	no KO assigned | (GenBank) hypothetical protein	Hypothetical	18.706	18.480	18.066	245.055	86.093	197.223
AMK58_16450	no KO assigned | (GenBank) chemotaxis protein	Chemotaxis	26.456	20.124	36.451	135.852	26.503	107.272
AMK58_16455	K01989 putative ABC transport system substrate-binding protein | (GenBank) ABC transporter substrate	Membrane Transport	154.764	169.676	189.897	182.671	15.897	170.910
AMK58_16460	no KO assigned | (GenBank) hypothetical protein	Hypothetical	84.259	111.356	142.507	173.535	33.071	147.408
AMK58_16465	no KO assigned | (GenBank) hypothetical protein	Hypothetical	76.179	82.488	130.909	319.716	30.496	164.286
AMK58_16470	no KO assigned | (GenBank) hypothetical protein	Hypothetical	62.884	67.289	92.065	174.130	24.265	154.770
AMK58_16475	no KO assigned | (GenBank) hypothetical protein	Hypothetical	48.938	42.718	77.955	216.035	19.289	85.897
AMK58_16480	no KO assigned | (GenBank) hypothetical protein	Hypothetical	16.143	17.794	24.009	62.446	3.978	41.746
AMK58_16485	no KO assigned | (GenBank) two-component system response regulator	Signal Transduction	19.372	28.934	29.624	164.997	14.880	114.365
AMK58_16490	no KO assigned | (GenBank) hypothetical protein	Hypothetical	400.326	449.578	549.386	75.095	19.218	210.735
AMK58_16495	no KO assigned | (GenBank) hypothetical protein	Hypothetical	571.586	518.685	586.303	2455.419	346.563	2044.352
AMK58_16500	no KO assigned | (GenBank) hypothetical protein	Hypothetical	10.939	11.816	14.221	105.143	31.748	94.962
AMK58_16505	no KO assigned | (GenBank) hypothetical protein	Hypothetical	372.720	393.591	749.684	449.986	146.434	400.373
AMK58_16510	no KO assigned | (GenBank) GCN5 family acetyltransferase	Acetyltransferase	16.229	29.174	33.047	30.013	22.279	59.797
AMK58_16515	K16841 allantoin racemase [EC:5.1.99.3] | (GenBank) Asp/Glu/hydantoin racemase	Metabolism: Nucleotide	64.995	58.101	85.684	663.942	167.976	548.874
AMK58_16520	K02053 putative spermidine/putrescine transport system permease protein | (GenBank) ABC transporter	Membrane Transport	3.748	3.445	4.523	104.262	14.905	108.527
AMK58_16525	K02054 putative spermidine/putrescine transport system permease protein | (GenBank) ABC transporter	Membrane Transport	15.724	21.920	22.301	198.344	33.876	125.149
AMK58_16530	K02055 putative spermidine/putrescine transport system substrate-binding protein | (GenBank) ABC tra	Quorum sensing	0.092	1.648	1.049	115.882	10.334	43.305
AMK58_16535	K02052 putative spermidine/putrescine transport system ATP-binding protein | (GenBank) Fe3+/spermidi	Quorum sensing	1.068	1.418	4.289	86.340	20.814	137.365
AMK58_16540	no KO assigned | (GenBank) transcriptional regulator	Transcription	3.400	1.766	4.357	282.984	36.724	221.112
AMK58_16545	K01772 protoporphyrin/coproporphyrin ferrochelatase [EC:4.99.1.1 4.99.1.9] | (GenBank) hemH; ferroch	Metabolism: Co-Factors and Vitamens	4.237	4.663	7.738	184.587	47.495	208.826
AMK58_16550	K05845 osmoprotectant transport system substrate-binding protein | (GenBank) ABC transporter	Membrane transport	0.386	0.768	3.911	228.472	19.648	163.756
AMK58_16555	K05846 osmoprotectant transport system permease protein | (GenBank) osmoprotectant uptake system per	Membrane transport	1.548	2.467	3.140	112.548	15.663	86.140
AMK58_16560	K05847 osmoprotectant transport system ATP-binding protein | (GenBank) ABC transporter	Transport	112.694	117.093	120.023	206.403	54.640	212.463
AMK58_16565	K05846 osmoprotectant transport system permease protein | (GenBank) osmoprotectant uptake system per	Membrane Transport	348.031	450.088	383.429	322.676	120.260	271.502
AMK58_16570	no KO assigned | (GenBank) hypothetical protein	Hypothetical	40.907	45.107	53.905	208.157	48.759	54.735
AMK58_16575	no KO assigned | (GenBank) hypothetical protein	Hypothetical	41.355	44.652	46.663	403.462	61.326	196.818
AMK58_16580	no KO assigned | (GenBank) signal protein PDZ	No COG	21.352	31.707	37.904	175.412	26.636	58.595
AMK58_16585	K02614 acyl-CoA thioesterase [EC:3.1.2.-] | (GenBank) aromatic compounds catabolism protein	Metabolism: Amino Acid	328.752	318.142	465.272	167.817	144.284	410.096
AMK58_16590	no KO assigned | (GenBank) enoyl-CoA hydratase	hydratase	2.267	7.225	4.168	141.735	84.952	175.062
AMK58_16595	K00033 6-phosphogluconate dehydrogenase [EC:1.1.1.44 1.1.1.343] | (GenBank) 6-phosphogluconate deh	Metabolism	37.338	40.103	44.197	132.963	232.481	92.508
AMK58_16600	K06145 LacI family transcriptional regulator, gluconate utilization system Gnt-I transcriptional rep	Transcription	22.101	15.788	20.443	67.577	80.333	42.324
AMK58_16605	K00851 gluconokinase [EC:2.7.1.12] | (GenBank) gluconokinase	Metabolism: Carbon	100.896	89.109	90.093	163.361	134.121	85.219
AMK58_16610	K01690 phosphogluconate dehydratase [EC:4.2.1.12] | (GenBank) phosphogluconate dehydratase	Metabolism: Carbon	23.235	27.682	30.711	91.483	91.257	60.151
AMK58_16615	no KO assigned | (GenBank) 2-keto-4-pentenoate hydratase	Metabolism: Carbon	8.863	12.292	12.490	220.064	66.890	104.492
AMK58_16620	K02529 LacI family transcriptional regulator | (GenBank) LacI family transcriptional regulator	Transcription	4.254	4.809	5.029	148.016	64.751	128.476
AMK58_16625	no KO assigned | (GenBank) dihydrofolate reductase	No COG	11.734	8.474	14.466	270.828	110.875	154.954
AMK58_16630	no KO assigned | (GenBank) glnQ; glutamine ABC transporter ATP-binding protein	Membrane Transport	122.763	108.182	124.019	252.632	67.270	172.175
AMK58_16635	K02030 polar amino acid transport system substrate-binding protein | (GenBank) ABC transporter subst	Transport: Amino Acid	23.127	18.964	23.139	246.417	52.670	115.914
AMK58_16640	K02029 polar amino acid transport system permease protein | (GenBank) amino acid ABC transporter per	Transport: Amino Acid	57.283	38.890	58.532	167.418	69.405	77.778
AMK58_16645	K02029 polar amino acid transport system permease protein | (GenBank) ABC transporter permease	Transport: Amino Acid	237.486	176.820	220.005	149.932	133.069	286.080
AMK58_16650	K03750 molybdopterin molybdotransferase [EC:2.10.1.1] | (GenBank) molybdopterin biosynthesis protein	Metabolism: Co-Factors and Vitamens	338.351	263.669	290.144	140.924	91.708	120.077
AMK58_16655	K09948 uncharacterized protein | (GenBank) alkaline phosphatase	Enzyme	158.841	123.877	156.308	321.892	155.654	279.129
AMK58_16660	K09138 uncharacterized protein | (GenBank) hypothetical protein	Hypothetical	93.909	64.804	84.644	41.626	114.674	103.480
AMK58_16665	K04085 tRNA 2-thiouridine synthesizing protein A [EC:2.8.1.-] | (GenBank) regulator of disulfide-bon	tRNA synthesis/modification	658.504	674.521	778.631	1169.518	836.083	604.456
AMK58_16670	no KO assigned | (GenBank) short-chain dehydrogenase	dehydrogenase	125.843	118.822	101.296	49.232	92.167	66.326
AMK58_16675	K07090 uncharacterized protein | (GenBank) hypothetical protein	Hypothetical	96.537	94.518	102.686	323.939	156.961	127.089
AMK58_16680	no KO assigned | (GenBank) ferredoxin	ferredoxin	53.213	42.866	38.657	113.730	74.599	170.206
AMK58_16685	no KO assigned | (GenBank) hypothetical protein	Hypothetical	3.594	4.129	5.125	199.215	232.570	144.054
AMK58_16690	K01494 dCTP deaminase [EC:3.5.4.13] | (GenBank) deoxycytidine triphosphate deaminase	Metabolism: Nucleotide	9.727	3.710	8.068	204.326	140.048	105.553
AMK58_16695	K03710 GntR family transcriptional regulator | (GenBank) GntR family transcriptional regulator	Transcription	4.956	4.230	4.039	89.345	74.971	69.725
AMK58_16700	K01999 branched-chain amino acid transport system substrate-binding protein | (GenBank) ABC transpor	Transport: Amino Acid	15.463	10.458	16.551	122.454	115.980	141.938
AMK58_16705	K01997 branched-chain amino acid transport system permease protein | (GenBank) branched-chain amino	Transport: Amino Acid	216.770	229.518	224.568	106.080	242.099	187.888
AMK58_16710	K01998 branched-chain amino acid transport system permease protein | (GenBank) ABC transporter perme	Transport: Amino Acid	7.393	6.517	8.643	183.616	21.660	90.468
AMK58_16715	K01995 branched-chain amino acid transport system ATP-binding protein | (GenBank) ABC transporter A'	Transport: Amino Acid	57.215	49.386	59.023	211.906	33.158	347.179
AMK58_16720	K01996 branched-chain amino acid transport system ATP-binding protein | (GenBank) livF; ABC transpor	Transport: Amino Acid	18.483	27.354	24.668	139.925	17.917	235.074
AMK58_16725	no KO assigned | (GenBank) carboxyvinyl-carboxyphosphonate phosphorylmutase	No COG	8.453	5.702	9.982	204.559	9.502	188.128
AMK58_16730	K01474 N-methylhydantoinase B [EC:3.5.2.14] | (GenBank) hydantoin utilization protein B	Metabolism: Amino Acid	1.387	0.414	2.176	54.062	2.494	33.565
AMK58_16735	K01473 N-methylhydantoinase A [EC:3.5.2.14] | (GenBank) hydantoin utilization protein A	Metabolism: Amino Acid	2.714	2.760	2.525	77.729	4.608	75.114
AMK58_16740	no KO assigned | (GenBank) cysteine hydrolase	hydrolase	6.890	8.233	13.852	260.816	23.014	209.570
AMK58_16745	K00244 fumarate reductase flavoprotein subunit [EC:1.3.5.4] | (GenBank) 3-ketosteroid dehydrogenase	Metabolism	10.728	9.665	10.767	189.217	21.247	191.932

AMK58_16750	K01703 3-isopropylmalate/(R)-2-methylmalate dehydratase large subunit [EC:4.2.1.33 4.2.1.35] | (GenB	Metabolism	8.003	8.740	11.700	308.810	25.245	122.802
AMK58_16755	K01704 3-isopropylmalate/(R)-2-methylmalate dehydratase small subunit [EC:4.2.1.33 4.2.1.35] | (GenB	Metabolism	9.329	10.905	11.570	162.729	37.158	172.636
AMK58_16760	no KO assigned | (GenBank) transcriptional regulator	Transcription	10.798	8.871	13.219	286.583	60.385	124.285
AMK58_16765	K14233 tRNA Ser | (GenBank) tRNA-Ser	tRNA synthesis/modification	99.550	80.373	129.189	2524.693	756.073	1600.467
AMK58_16770	no KO assigned | (GenBank) hypothetical protein	Hypothetical	9.086	12.624	14.867	201.812	59.853	160.861
AMK58_16775	K01142 exodeoxyribonuclease III [EC:3.1.11.2] | (GenBank) exodeoxyribonuclease III	DNA Repair and Replication	64.696	67.182	78.943	270.722	74.626	194.686
AMK58_16780	K15724 iron-sulfur cluster insertion protein | (GenBank) heme biosynthesis protein HemY	No COG	40.030	52.928	50.228	42.195	42.414	162.167
AMK58_16785	K01129 dGTPase [EC:3.1.5.1] | (GenBank) deoxyguanosinetriphosphate triphosphohydrolase	Metabolism: Nucleotide	38.744	46.415	35.303	49.467	106.833	130.794
AMK58_16790	K01887 arginyl-tRNA synthetase [EC:6.1.1.19] | (GenBank) arginine--tRNA ligase	tRNA synthesis/modification	106.224	100.226	91.258	33.984	365.311	106.404
AMK58_16795	no KO assigned | (GenBank) hypothetical protein	Hypothetical	159.730	185.536	166.698	121.990	622.435	133.533
AMK58_16800	K01207 beta-N-acetylhexosaminidase [EC:3.2.1.52] | (GenBank) beta-hexosaminidase	Defense	81.164	92.173	96.942	244.701	113.223	279.897
AMK58_16805	no KO assigned | (GenBank) peptidase M48	Peptidases	210.453	231.210	200.626	369.053	199.065	309.126
AMK58_16810	K05896 segregation and condensation protein A | (GenBank) chromosome segregation protein ScpA	Cell Cycle/Shape/Homeostatsis	34.593	46.811	37.248	118.421	28.083	36.601
AMK58_16815	K06024 segregation and condensation protein B | (GenBank) segregation and condensation protein B	Cell Cycle/Shape/Homeostatsis	40.358	48.906	48.509	267.505	44.722	88.615
AMK58_16820	K02010 iron(III) transport system ATP-binding protein [EC:3.6.3.30] | (GenBank) Fe3+/spermidine/putr	Transport: Metal	102.628	110.207	102.682	148.259	41.720	102.110
AMK58_16825	K03116 sec-independent protein translocase protein TatA | (GenBank) preprotein translocase	Secretion system	249.150	241.119	277.617	649.847	191.641	444.496
AMK58_16830	K03117 sec-independent protein translocase protein TatB | (GenBank) preprotein translocase subunit T	Secretion system	69.583	58.201	72.115	308.526	54.489	288.632
AMK58_16835	K03118 sec-independent protein translocase protein TatC | (GenBank) preprotein translocase subunit T	Secretion system	125.291	154.748	138.171	270.512	148.450	179.017
AMK58_16840	no KO assigned | (GenBank) hypothetical protein	Hypothetical	217.394	189.539	220.842	67.159	681.518	81.405
AMK58_16845	K01875 seryl-tRNA synthetase [EC:6.1.1.11] | (GenBank) serine--tRNA ligase	tRNA synthesis/modification	235.168	243.404	220.690	58.290	883.746	81.054
AMK58_16850	K03787 5'-nucleotidase [EC:3.1.3.5] | (GenBank) stationary phase survival protein SurE	Metabolism	257.149	191.232	220.534	103.981	661.694	247.624
AMK58_16855	K00573 protein-L-isoaspartate(D-aspartate) O-methyltransferase [EC:2.1.1.77] | (GenBank) protein-L-i	Transferases	24.807	23.227	21.827	170.618	186.036	386.077
AMK58_16860	no KO assigned | (GenBank) gamma-D-glutamyl-meso-diaminopimelate peptidase	peptidase	157.674	155.427	146.532	191.072	128.532	245.177
AMK58_16865	K06923 uncharacterized protein | (GenBank) AAA family ATPase	enzyme	150.426	141.636	139.538	104.910	141.962	169.320
AMK58_16870	no KO assigned | (GenBank) hypothetical protein	Hypothetical	121.402	142.523	127.392	150.148	375.588	215.297
AMK58_16875	K03210 preprotein translocase subunit YajC | (GenBank) preprotein translocase subunit YajC	Secretion system	279.904	283.898	287.797	337.451	443.400	277.354
AMK58_16880	K03072 preprotein translocase subunit SecD | (GenBank) preprotein translocase subunit SecD	Secretion system	146.744	130.738	128.798	139.466	92.261	73.161
AMK58_16885	K03074 preprotein translocase subunit SecF | (GenBank) preprotein translocase subunit SecF	Secretion system	21.702	23.505	24.375	221.008	83.168	110.034
AMK58_16890	no KO assigned | (GenBank) hypothetical protein	Hypothetical	1235.411	920.460	978.816	78.519	726.190	169.890
AMK58_16895	K04564 superoxide dismutase, Fe-Mn family [EC:1.15.1.1] | (GenBank) superoxide dismutase	Defense	733.841	583.698	686.724	369.067	595.801	400.169
AMK58_16900	no KO assigned | (GenBank) phytoene dehydrogenase	dehydrogenase	268.008	190.908	212.588	98.103	184.883	151.748
AMK58_16905	K02291 15-cis-phytoene/all-trans-phytoene synthase [EC:2.5.1.32 2.5.1.99] | (GenBank) squalene synth	Metabolism	53.294	43.278	64.570	107.777	149.470	122.465
AMK58_16910	no KO assigned | (GenBank) squalene synthase	Enzyme	2805.246	2851.993	2897.358	70.039	827.939	189.302
AMK58_16915	K02291 15-cis-phytoene/all-trans-phytoene synthase [EC:2.5.1.32 2.5.1.99] | (GenBank) phytoene synth	Biosynthesis: Terpenoid	8.496	16.921	10.233	228.388	55.233	129.471
AMK58_16920	K03406 methyl-accepting chemotaxis protein | (GenBank) chemotaxis protein	Chemotaxis	15.666	20.528	21.208	80.352	44.382	48.224
AMK58_16925	K04094 methylenetetrahydrofolate--tRNA-(uracil-5-)-methyltransferase [EC:2.1.1.74] | (GenBank) tRNA	tRNA synthesis/modification	58.985	54.583	55.915	72.241	138.685	87.924
AMK58_16930	no KO assigned | (GenBank) hypothetical protein	Hypothetical	29.951	48.527	43.924	315.191	73.040	230.423
AMK58_16935	no KO assigned | (GenBank) hypothetical protein	Hypothetical	1101.720	1313.556	1093.916	2127.782	474.633	2159.934
AMK58_16940	K03701 excinuclease ABC subunit A | (GenBank) ABC-ATPase UvrA	DNA Repair and Replication	32.507	36.510	37.889	77.597	60.438	79.136
AMK58_16945	K03111 single-strand DNA-binding protein | (GenBank) single-stranded DNA-binding protein	DNA Repair and Replication	343.649	362.400	400.504	162.833	54.600	256.360
AMK58_16950	no KO assigned | (GenBank) hypothetical protein	Hypothetical	28.093	44.914	43.640	101.764	266.849	456.417
AMK58_16955	no KO assigned | (GenBank) hypothetical protein	Hypothetical	1354.885	1452.452	1293.718	1413.334	3328.343	2166.537
AMK58_16960	no KO assigned | (GenBank) hypothetical protein	Hypothetical	426.034	370.460	357.047	178.655	317.851	253.536
AMK58_16965	no KO assigned | (GenBank) hypothetical protein	Hypothetical	346.811	401.480	474.613	106.055	1603.816	189.981
AMK58_16970	no KO assigned | (GenBank) entericidin EcnAB	Defense	61.724	52.006	79.756	724.006	2035.171	529.795
AMK58_16975	K03327 multidrug resistance protein, MATE family | (GenBank) MATE family efflux transporter	Defense	1.141	0.000	4.421	123.990	60.552	61.911
AMK58_16980	K02469 DNA gyrase subunit A [EC:5.99.1.3] | (GenBank) DNA gyrase subunit A	DNA Repair and Replication	1.035	1.546	2.542	39.218	28.837	66.130
AMK58_16985	K00954 pantetheine-phosphate adenylyltransferase [EC:2.7.7.3] | (GenBank) phosphopantetheine adenyly	Metabolism: Co-Factors and Vitamens	4.411	1.318	7.129	71.492	659.891	141.717
AMK58_16990	K14229 tRNA Lys | (GenBank) tRNA-Lys	tRNA synthesis/modification	1701.419	1846.460	2134.245	3401.635	1081.583	3069.689
AMK58_16995	no KO assigned | (GenBank) hypothetical protein	Hypothetical	680.807	768.949	747.924	341.868	687.724	704.373
AMK58_17000	K07058 membrane protein | (GenBank) hypothetical protein	Membrane	104.175	93.433	98.810	92.581	65.920	114.258
AMK58_17005	no KO assigned | (GenBank) hypothetical protein	Hypothetical	7.544	6.760	10.400	14.315	21.057	40.921
AMK58_17010	no KO assigned | (GenBank) hypothetical protein	Hypothetical	8.657	3.043	16.342	101.643	325.994	191.366
AMK58_17015	no KO assigned | (GenBank) hypothetical protein	Hypothetical	6.777	8.390	10.856	93.484	196.831	145.560
AMK58_17020	K01687 dihydroxy-acid dehydratase [EC:4.2.1.9] | (GenBank) dihydroxy-acid dehydratase	Metabolism	8.759	9.691	9.747	81.614	75.121	121.956
AMK58_17025	no KO assigned | (GenBank) hypothetical protein	Hypothetical	243.002	200.366	196.459	462.572	303.175	481.495
AMK58_17030	K03665 GTPase | (GenBank) GTPase HflX	Ribosome	43.061	63.482	68.238	270.809	79.728	288.064
AMK58_17035	no KO assigned | (GenBank) hypothetical protein	Hypothetical	69.929	68.955	86.501	200.383	224.702	256.087
AMK58_17040	no KO assigned | (GenBank) hypothetical protein	Hypothetical	5.107	7.873	10.650	193.520	363.841	149.235
AMK58_17045	no KO assigned | (GenBank) phasin	No COG	22.014	28.664	39.446	661.219	1186.136	576.265
AMK58_17050	K06878 tRNA-binding protein | (GenBank) tRNA-binding protein	tRNA synthesis/modification	159.992	146.393	133.963	1332.724	99.640	632.185
AMK58_17055	K14441 ribosomal protein S12 methylthiotransferase [EC:2.8.4.4] | (GenBank) ribosomal protein S12 me	Ribosome	26.344	28.616	29.939	85.786	149.653	49.761
AMK58_17060	no KO assigned | (GenBank) transcriptional regulator	Transcription	761.555	886.854	723.042	52.951	1466.264	249.915
AMK58_17065	K00126 formate dehydrogenase subunit delta [EC:1.2.1.2] | (GenBank) formate dehydrogenase	Metabolism	404.107	485.368	320.439	123.570	113.116	411.655
AMK58_17070	K02379 FdhD protein | (GenBank) formate dehydrogenase family accessory protein FdhD	Biosynthesis: Co-Factors and Vitamins	247.387	259.525	288.530	123.806	415.032	343.094
AMK58_17075	K00123 formate dehydrogenase major subunit [EC:1.2.1.2] | (GenBank) formate dehydrogenase	Metabolism: Carbon	45.280	53.951	59.126	22.588	49.838	42.781
AMK58_17080	K00124 formate dehydrogenase iron-sulfur subunit | (GenBank) formate dehydrogenase	Metabolism: Carbon	34.250	40.186	43.151	35.731	24.855	41.151

AMK58_17085	K00127 formate dehydrogenase subunit gamma | (GenBank) ATP synthase subunit E	Metabolism: Carbon	234.969	289.934	274.967	231.173	87.111	140.968
AMK58_17090	no KO assigned | (GenBank) LysR family transcriptional regulator	Transcription	1315.167	1380.116	1566.958	345.138	188.658	658.649
AMK58_17095	K07552 MFS transporter, DHA1 family, bicyclomycin/chloramphenicol resistance protein | (GenBank) MI	Transport: MFS	614.937	705.643	643.975	224.831	55.792	203.433
AMK58_17100	no KO assigned | (GenBank) histidine kinase	Signal Transduction	216.042	246.711	210.062	48.349	26.424	70.120
AMK58_17105	no KO assigned | (GenBank) multidrug transporter	Transport	32.312	33.802	32.037	132.039	93.273	188.632
AMK58_17110	no KO assigned | (GenBank) hypothetical protein	Hypothetical	505.036	519.537	567.326	403.523	462.165	444.323
AMK58_17115	no KO assigned | (GenBank) hypothetical protein	Hypothetical	122.065	128.695	151.844	316.080	152.235	515.889
AMK58_17120	K07028 uncharacterized protein | (GenBank) hypothetical protein	Hypothetical	187.976	197.995	198.433	52.295	64.034	132.349
AMK58_17125	no KO assigned | (GenBank) universal stress protein UspA	stress Response	776.163	872.699	741.966	120.470	137.192	178.958
AMK58_17130	no KO assigned | (GenBank) hypothetical protein	Hypothetical	481.516	656.840	512.081	327.479	779.016	567.801
AMK58_17135	no KO assigned | (GenBank) hypothetical protein	Hypothetical	323.539	359.668	365.183	670.368	317.551	821.128
AMK58_17140	K01633 dihydroneopterin aldolase / 7,8-dihydroneopterin epimerase [EC:4.1.2.25 5.1.99.8] | (GenBank)	Metabolism: Co-Factors and Vitamins	2494.489	3506.637	3110.106	1347.878	5194.224	1393.900
AMK58_17145	K00950 2-amino-4-hydroxy-6-hydroxymethyldihydropteridine diphosphokinase [EC:2.7.6.3] | (GenBank)	Metabolism: Co-Factors and Vitamens	571.702	650.592	547.666	2890.875	8097.948	1181.378
AMK58_17150	no KO assigned | (GenBank) hypothetical protein	Hypothetical	1378.995	2331.334	1904.944	94.433	1944.188	229.156
AMK58_17155	K09386 uncharacterized protein | (GenBank) carbon monoxide dehydrogenase	dehydrogenase	114.171	113.156	118.148	134.206	428.972	198.770
AMK58_17160	K08738 cytochrome c | (GenBank) cytochrome C	Energy	274.563	292.380	287.344	264.350	362.201	401.637
AMK58_17165	K01256 aminopeptidase N [EC:3.4.11.2] | (GenBank) pepN; aminopeptidase	Metabolism: amino acids	1.002	2.177	3.075	56.076	43.419	35.734
AMK58_17170	no KO assigned | (GenBank) hypothetical protein	Hypothetical	417.661	462.611	437.943	279.111	344.453	246.297
AMK58_17175	K13628 iron-sulfur cluster assembly protein | (GenBank) hypothetical protein	tRNA synthesis/modification	768.842	1027.286	890.640	252.592	461.268	315.110
AMK58_17180	no KO assigned | (GenBank) hypothetical protein	Hypothetical	843.369	749.950	751.941	854.258	1494.023	1311.739
AMK58_17185	K04488 nitrogen fixation protein NifU and related proteins | (GenBank) nitrogen fixation protein Nif	Nitrogen	299.065	375.588	288.781	195.801	617.654	403.499
AMK58_17190	K11717 cysteine desulfurase / selenocysteine lyase [EC:2.8.1.7 4.4.1.16] | (GenBank) cysteine desulf	Metabolism: Amino Acid	314.814	371.128	238.077	13.740	473.471	64.357
AMK58_17195	K09015 Fe-S cluster assembly protein SufD | (GenBank) Fe-S cluster assembly protein SufD	No COG	152.058	194.709	119.194	36.958	65.738	69.134
AMK58_17200	K09013 Fe-S cluster assembly ATP-binding protein | (GenBank) sufC; Fe-S cluster assembly ATPase Suf	Transport	184.265	317.008	196.604	42.431	92.355	127.925
AMK58_17205	K09014 Fe-S cluster assembly protein SufB | (GenBank) Fe-S cluster assembly protein SufB	Biosynthesis: Co-Factors and Vitamins	355.576	428.277	319.517	131.667	208.438	179.043
AMK58_17210	no KO assigned | (GenBank) BadM/Rrf2 family transcriptional regulator	Transcription	1675.495	1833.100	1607.137	302.210	993.227	590.171
AMK58_17215	no KO assigned | (GenBank) hypothetical protein	Hypothetical	1005.603	1094.692	862.083	56.308	749.657	161.242
AMK58_17220	no KO assigned | (GenBank) hypothetical protein	Hypothetical	4400.793	5094.713	3687.119	251.641	7377.100	711.580
AMK58_17225	no KO assigned | (GenBank) hypothetical protein	Hypothetical	188.777	317.303	233.363	125.205	922.360	134.455
AMK58_17230	no KO assigned | (GenBank) hypothetical protein	Hypothetical	30.955	33.806	43.358	95.862	414.546	106.020
AMK58_17235	no KO assigned | (GenBank) hypothetical protein	Hypothetical	11.755	9.364	17.696	71.120	306.980	124.623
AMK58_17240	K14981 two-component system, OmpR family, response regulator ChvI | (GenBank) response regulator	Signal transduction	18.894	21.674	30.900	202.074	646.825	137.218
AMK58_17245	no KO assigned | (GenBank) histidine kinase	Signal Transduction	178.340	210.862	174.827	139.747	74.002	191.812
AMK58_17250	no KO assigned | (GenBank) pseudogene	Pseudogene	64.169	89.952	87.082	48.456	40.552	71.680
AMK58_17255	no KO assigned | (GenBank) peptide ABC transporter ATP-binding protein	Membrane Transport	363.486	401.864	457.620	69.983	197.019	141.457
AMK58_17260	no KO assigned | (GenBank) hypothetical protein	Hypothetical	269.460	324.513	416.621	634.517	292.993	815.995
AMK58_17265	no KO assigned | (GenBank) hypothetical protein	Hypothetical	55.686	44.814	59.071	367.840	102.643	393.662
AMK58_17270	no KO assigned | (GenBank) hypothetical protein	Hypothetical	202.360	193.574	223.752	1027.939	535.428	815.932
AMK58_17275	K03088 RNA polymerase sigma-70 factor, ECF subfamily | (GenBank) RNA polymerase subunit sigma-7(	Transcription	258.753	286.901	271.525	145.078	227.109	113.928
AMK58_17280	no KO assigned | (GenBank) carboxylate--amine ligase	Enzyme	20.996	26.357	28.819	172.801	286.420	191.964
AMK58_17285	no KO assigned | (GenBank) ribosomal-protein-alanine acetyltransferase	acetyltransferase	110.718	168.400	151.563	49.342	2457.598	268.794
AMK58_17290	K00688 glycogen phosphorylase [EC:2.4.1.1] | (GenBank) maltodextrin phosphorylase	Metabolism: Carbohydrates	2.810	3.473	3.593	20.638	430.771	62.941
AMK58_17295	K00133 aspartate-semialdehyde dehydrogenase [EC:1.2.1.11] | (GenBank) aspartate-semialdehyde dehydro	Metabolism: Amino Acid	55.284	63.490	63.009	291.583	271.918	377.000
AMK58_17300	no KO assigned | (GenBank) hypothetical protein	Hypothetical	144.493	159.257	152.308	354.740	648.823	319.945
AMK58_17305	K02837 peptide chain release factor 3 | (GenBank) prfC; peptide chain release factor 3	Translation	578.268	556.012	565.500	215.138	377.726	367.180
AMK58_17310	no KO assigned | (GenBank) PAS domain-containing sensor histidine kinase	Signal Transduction	96.599	80.220	79.153	147.283	269.944	181.567
AMK58_17315	no KO assigned | (GenBank) regulator	Signal transduction	86.058	83.328	71.667	62.012	68.714	318.923
AMK58_17320	K07160 UPF0271 protein | (GenBank) hypothetical protein	No COG	285.457	263.376	268.758	332.481	242.002	485.638
AMK58_17325	no KO assigned | (GenBank) allophanate hydrolase	hydrolase	376.567	389.347	354.583	223.486	322.509	332.003
AMK58_17330	no KO assigned | (GenBank) allophanate hydrolase	hydrolase	276.814	294.145	259.217	37.462	83.388	72.517
AMK58_17335	no KO assigned | (GenBank) C4-dicarboxylate ABC transporter permease	Membrane Transport	335.327	343.899	328.911	92.678	62.298	107.958
AMK58_17340	no KO assigned | (GenBank) hypothetical protein	Hypothetical	531.067	563.083	476.276	381.932	122.217	114.911
AMK58_17345	no KO assigned | (GenBank) C4-dicarboxylate ABC transporter substrate-binding protein	Membrane Transport	319.922	290.222	270.944	138.359	48.398	51.187
AMK58_17350	no KO assigned | (GenBank) hypothetical protein	Hypothetical	2397.533	1839.366	1766.889	150.923	149.009	253.776
AMK58_17355	no KO assigned | (GenBank) LysR family transcriptional regulator	Transcription	956.529	672.277	643.853	53.220	45.647	56.893
AMK58_17360	no KO assigned | (GenBank) pseudogene	Pseudogene	4934.221	3809.943	3780.545	162.186	199.883	195.188
AMK58_17365	no KO assigned | (GenBank) acriflavine resistance protein B	Defense	64.279	60.222	64.729	41.442	18.303	19.520
AMK58_17370	no KO assigned | (GenBank) LysR family transcriptional regulator	Transcription	29.723	37.471	31.632	134.424	52.721	235.412
AMK58_17375	K07090 uncharacterized protein | (GenBank) hypothetical protein	Hypothetical	129.146	154.316	145.817	296.692	198.515	142.508
AMK58_17380	no KO assigned | (GenBank) nodulation protein NolG	No COG	116.150	107.113	117.606	143.942	73.863	177.234
AMK58_17385	no KO assigned | (GenBank) RND transporter	transport	16.998	13.707	20.823	179.368	40.441	142.344
AMK58_17390	no KO assigned | (GenBank) hypothetical protein	Hypothetical	4.036	14.248	8.896	213.299	35.879	116.692
AMK58_17395	no KO assigned | (GenBank) transglycosylase	transglycosylase	83.855	77.694	96.039	552.934	167.218	532.137
AMK58_17400	K03406 methyl-accepting chemotaxis protein | (GenBank) hypothetical protein	Chemotaxis	9.051	13.704	12.260	117.093	32.194	56.394
AMK58_17405	no KO assigned | (GenBank) methyltransferase type 11	methyltransferase	22.014	18.267	28.954	136.629	75.882	152.868
AMK58_17410	K03168 DNA topoisomerase I [EC:5.99.1.2] | (GenBank) DNA topoisomerase I	DNA Repair and Replication	65.039	79.740	77.149	160.199	54.533	160.792
AMK58_17415	no KO assigned | (GenBank) hypothetical protein	Hypothetical	296.478	187.794	262.707	1152.975	490.721	882.324

AMK58_17420	K01187 alpha-glucosidase [EC:3.2.1.20] | (GenBank) alpha-glucosidase	Metabolism: Carbohydrate	9.308	8.452	8.142	150.316	41.096	47.228
AMK58_17425	no KO assigned | (GenBank) ABC transporter substrate-binding protein	Membrane Transport	1.750	1.960	4.264	214.197	103.146	138.943
AMK58_17430	K00799 glutathione S-transferase [EC:2.5.1.18] | (GenBank) glutathione S-transferase	Metabolism: Amino Acid	50.969	54.337	52.245	110.154	87.534	97.863
AMK58_17435	K13893 microcin C transport system substrate-binding protein | (GenBank) hypothetical protein	Transport	25.911	28.903	30.560	203.287	75.309	156.944
AMK58_17440	K11443 two-component system, cell cycle response regulator DivK | (GenBank) cell division protein	Cell Cycle/Shape/Homeostatsis	6644.764	6099.406	5915.976	180.607	1341.778	495.373
AMK58_17445	no KO assigned | (GenBank) hypothetical protein	Hypothetical	498.197	411.977	436.080	224.840	115.664	223.300
AMK58_17450	K00641 homoserine O-acetyltransferase [EC:2.3.1.31] | (GenBank) homoserine acetyltransferase	Metabolism: Amino Acid	150.812	158.328	140.124	175.058	370.646	331.557
AMK58_17455	no KO assigned | (GenBank) hypothetical protein	Hypothetical	88.260	90.138	78.539	79.830	109.807	316.524
AMK58_17460	no KO assigned | (GenBank) hypothetical protein	Hypothetical	37.312	47.769	41.812	82.878	79.602	217.831
AMK58_17465	no KO assigned | (GenBank) hypothetical protein	Hypothetical	50.482	55.580	71.673	139.462	289.871	290.633
AMK58_17470	no KO assigned | (GenBank) hypothetical protein	Hypothetical	13.353	13.445	15.406	63.508	76.226	58.987
AMK58_17475	no KO assigned | (GenBank) hypothetical protein	Hypothetical	26.442	38.801	41.460	648.122	133.486	447.788
AMK58_17480	no KO assigned | (GenBank) hypothetical protein	Hypothetical	9.807	18.028	13.269	408.244	3126.328	541.413
AMK58_17485	no KO assigned | (GenBank) threonine transporter	Transport	8.186	9.099	13.213	190.237	1056.434	329.042
AMK58_17490	no KO assigned | (GenBank) hypothetical protein	Hypothetical	1.527	0.652	1.971	68.819	167.726	40.458
AMK58_17495	K06959 protein Tex | (GenBank) RNA-binding transcriptional accessory protein	Transcription	2.299	4.578	3.546	28.263	106.463	36.334
AMK58_17500	no KO assigned | (GenBank) oxidoreductase	Oxidoreductase	14.584	18.137	17.206	75.272	158.307	91.681
AMK58_17505	no KO assigned | (GenBank) formate hydrogenlyase	Enzyme	129.516	137.895	149.730	145.049	274.558	244.352
AMK58_17510	K12140 hydrogenase-4 component E [EC:1.-.-.-] | (GenBank) hydrogenase-4 component E	hydrogenase	256.942	209.479	253.505	500.501	338.693	594.538
AMK58_17515	K12141 hydrogenase-4 component F [EC:1.-.-.-] | (GenBank) hydrogenase 4 subunit F	hydrogenase	210.012	198.948	234.896	259.965	48.538	168.790
AMK58_17520	no KO assigned | (GenBank) hydrogenase expression protein HypE	enzyme	31.407	28.146	40.389	136.102	22.241	95.742
AMK58_17525	no KO assigned | (GenBank) hydrogenase	hydrogenase	73.526	90.073	82.259	149.447	45.364	225.400
AMK58_17530	K04765 nucleoside triphosphate diphosphatase [EC:3.6.1.9] | (GenBank) nucleoside triphosphate hydrol	Metabolism	122.271	125.917	155.482	300.344	72.415	293.703
AMK58_17535	no KO assigned | (GenBank) hypothetical protein	Hypothetical	90.806	94.725	166.648	667.665	237.191	391.073
AMK58_17540	no KO assigned | (GenBank) hypothetical protein	Hypothetical	54.072	86.615	93.138	317.024	111.869	182.393
AMK58_17545	no KO assigned | (GenBank) inositol-1-monophosphatase	No COG	69.100	48.046	61.029	138.048	123.204	145.688
AMK58_17550	no KO assigned | (GenBank) hypothetical protein	Hypothetical	9.983	6.345	10.300	75.402	240.670	242.266
AMK58_17555	no KO assigned | (GenBank) hypothetical protein	Hypothetical	39.933	45.686	55.742	233.889	164.267	205.237
AMK58_17560	K03665 GTPase | (GenBank) GTPase HflX	Ribosome	80.717	73.099	79.785	119.254	121.827	116.926
AMK58_17565	K03666 host factor-I protein | (GenBank) RNA-binding protein hfq	Quorum sensing	113.295	159.777	137.773	546.482	265.081	315.167
AMK58_17570	K01091 phosphoglycolate phosphatase [EC:3.1.3.18] | (GenBank) HAD family hydrolase	Metabolism	262.415	454.296	311.101	102.323	440.794	186.956
AMK58_17575	K00824 D-alanine transaminase [EC:2.6.1.21] | (GenBank) D-amino acid aminotransferase	Metabolism: Amino Acid	420.819	418.609	398.519	174.575	660.147	379.560
AMK58_17580	K03499 trk system potassium uptake protein | (GenBank) potassium transporter TrkA	Transport: Inorganic	450.711	379.276	342.431	17.785	711.302	120.586
AMK58_17585	no KO assigned | (GenBank) hypothetical protein	Hypothetical	463.532	404.985	420.240	273.459	191.147	448.715
AMK58_17590	K13599 two-component system, NtrC family, nitrogen regulation response regulator NtrX | (GenBank) AA	Nitrogen	146.223	168.835	165.817	100.252	95.586	121.165
AMK58_17595	K13598 two-component system, NtrC family, nitrogen regulation sensor histidine kinase NtrY [EC:2.7.1	Nitrogen	89.320	88.472	99.552	50.779	79.651	123.197
AMK58_17600	K07712 two-component system, NtrC family, nitrogen regulation response regulator GlnG | (GenBank) ni	Nitrogen	20.430	16.508	21.575	8.749	10.447	66.539
AMK58_17605	K07708 two-component system, NtrC family, nitrogen regulation sensor histidine kinase GlnL [EC:2.7.1	Nitrogen	181.260	199.028	200.286	160.568	82.470	153.239
AMK58_17610	K05540 tRNA-dihydrouridine synthase B [EC:1.-.-.-] | (GenBank) tRNA-dihydrouridine synthase	tRNA synthesis/modification	468.843	447.059	512.262	441.533	241.300	472.852
AMK58_17615	no KO assigned | (GenBank) hypothetical protein	Hypothetical	139.473	120.559	135.011	126.211	78.053	351.255
AMK58_17620	K12506 2-C-methyl-D-erythritol 4-phosphate cytidylyltransferase / 2-C-methyl-D-erythritol 2,4-cyclod	Metabolism: terpenoids and polyketide	144.241	133.398	128.674	100.962	66.338	233.968
AMK58_17625	K03743 nicotinamide-nucleotide amidase [EC:3.5.1.42] | (GenBank) damage-inducible protein CinA	Metabolism: Co-Factors and Vitamins	209.959	200.932	226.812	225.580	292.168	393.167
AMK58_17630	K18588 coenzyme Q-binding protein COQ10 | (GenBank) cyclase	No COG	138.667	146.835	150.048	309.124	249.213	290.750
AMK58_17635	K03644 lipoyl synthase [EC:2.8.1.8] | (GenBank) lipoyl synthase	Metabolism: Co-Factors and Vitamins	44.533	55.933	56.176	248.278	193.403	170.223
AMK58_17640	K00382 dihydrolipoamide dehydrogenase [EC:1.8.1.4] | (GenBank) dihydrolipoamide dehydrogenase	Metabolism	35.508	37.254	37.550	50.930	74.764	51.075
AMK58_17645	K00627 pyruvate dehydrogenase E2 component (dihydrolipoamide acetyltransferase) [EC:2.3.1.12] | (Gen	Metabolism: Carbohydrate	64.916	77.676	71.681	44.145	225.401	68.651
AMK58_17650	K00162 pyruvate dehydrogenase E1 component beta subunit [EC:1.2.4.1] | (GenBank) pyruvate dehydroge	Metabolism: Carbohydrate	251.936	233.357	253.130	89.446	350.649	170.332
AMK58_17655	K00161 pyruvate dehydrogenase E1 component alpha subunit [EC:1.2.4.1] | (GenBank) pyruvate dehydrog	Metabolism: Carbon	1587.544	1529.218	1764.591	129.653	287.709	304.905
AMK58_17660	no KO assigned | (GenBank) septation ring formation regulator EzrA	Cell Cycle/Shape/Homeostatsis	4828.990	3865.277	4696.774	295.134	724.442	457.480
AMK58_17665	K10806 acyl-CoA thioesterase YciA [EC:3.1.2.-] | (GenBank) acyl-CoA thioesterase	Lipid metabolism	4852.863	4024.424	5011.953	400.603	865.187	585.848
AMK58_17670	K02622 topoisomerase IV subunit B [EC:5.99.1.-] | (GenBank) DNA topoisomerase IV subunit B	DNA Repair and Replication	1286.716	1150.677	1233.897	48.843	243.654	113.342
AMK58_17675	no KO assigned | (GenBank) hypothetical protein	Hypothetical	134.745	168.524	145.652	48.540	71.217	126.354
AMK58_17680	no KO assigned | (GenBank) alpha/beta hydrolase	hydrolase	20.265	25.651	25.636	89.720	76.058	112.270
AMK58_17685	no KO assigned | (GenBank) isochorismatase	Enzyme	342.042	400.976	336.029	632.316	475.449	831.788
AMK58_17690	no KO assigned | (GenBank) DNA helicase	DNA Repair and Replication	31.218	30.545	36.458	78.966	41.794	81.167
AMK58_17695	no KO assigned | (GenBank) AsnC family transcriptional regulator	Transcription	284.999	389.957	367.149	559.308	272.746	461.091
AMK58_17700	K04744 LPS-assembly protein | (GenBank) organic solvent tolerance protein	Transport	28.849	34.832	30.918	49.215	21.637	67.669
AMK58_17705	no KO assigned | (GenBank) hypothetical protein	Hypothetical	1375.350	1304.232	1272.492	173.802	1242.460	363.253
AMK58_17710	no KO assigned | (GenBank) hypothetical protein	Hypothetical	54.854	46.355	50.897	8.460	77.545	24.930
AMK58_17715	K09978 uncharacterized protein | (GenBank) hypothetical protein	Hypothetical	1097.240	964.474	948.361	861.612	335.373	1473.874
AMK58_17720	no KO assigned | (GenBank) hypothetical protein	Hypothetical	289.630	232.608	241.975	331.368	193.199	620.345
AMK58_17725	no KO assigned | (GenBank) hypothetical protein	Hypothetical	14.660	20.608	22.302	340.198	35.962	170.564
AMK58_17730	no KO assigned | (GenBank) peptidylprolyl isomerase	No COG	39.441	52.060	41.861	122.254	16.238	40.825
AMK58_17735	no KO assigned | (GenBank) hypothetical protein	Hypothetical	13.925	15.820	13.283	6.054	2.976	11.134
AMK58_17740	K01785 aldose 1-epimerase [EC:5.1.3.3] | (GenBank) hypothetical protein	Metabolism: Carbohydrate	28.808	30.850	33.334	261.573	42.157	152.591
AMK58_17745	no KO assigned | (GenBank) hypothetical protein	Hypothetical	1257.683	925.181	920.809	83.496	166.504	180.879
AMK58_17750	no KO assigned | (GenBank) hypothetical protein	Hypothetical	69.223	72.570	87.302	518.575	93.078	480.689

AMK58_17755	no KO assigned | (GenBank) IMP dehydrogenase	dehydrogenase	15.193	17.981	15.377	66.788	24.700	54.908
AMK58_17760	no KO assigned | (GenBank) hypothetical protein	Hypothetical	11.249	15.590	15.051	93.055	16.816	53.178
AMK58_17765	no KO assigned | (GenBank) hypothetical protein	Hypothetical	19.774	23.059	21.701	44.041	17.353	67.203
AMK58_17770	no KO assigned | (GenBank) transglutaminase	Enzyme	381.802	378.901	399.810	590.734	123.996	634.175
AMK58_17775	K18138 multidrug efflux pump | (GenBank) multidrug transporter AcrB	Defense	14.616	18.993	19.316	67.928	24.098	157.781
AMK58_17780	K03585 membrane fusion protein, multidrug efflux system | (GenBank) hemolysin D	Defense	172.750	195.829	194.707	388.016	68.287	296.020
AMK58_17785	no KO assigned | (GenBank) hypothetical protein	Hypothetical	37.242	53.188	40.207	242.683	21.014	174.183
AMK58_17790	no KO assigned | (GenBank) glycosyl transferase	Glycosyl Transferase	429.598	427.628	408.599	536.852	275.348	726.558
AMK58_17795	K01358 ATP-dependent Clp protease, protease subunit [EC:3.4.21.92] | (GenBank) ATP-dependent Clp pr	Peptidase	205.192	216.736	234.981	567.347	117.998	377.596
AMK58_17800	no KO assigned | (GenBank) hypothetical protein	Hypothetical	76.169	88.297	75.125	845.955	229.378	708.933
AMK58_17805	no KO assigned | (GenBank) hypothetical protein	Hypothetical	31.493	40.419	32.162	225.935	60.049	153.587
AMK58_17810	no KO assigned | (GenBank) hypothetical protein	Hypothetical	377.834	350.104	313.430	24.004	672.119	121.551
AMK58_17815	K08990 putative membrane protein | (GenBank) hypothetical protein	Membrane	110.491	125.771	123.634	13.554	93.474	54.463
AMK58_17820	K06918 uncharacterized protein | (GenBank) hypothetical protein	Hypothetical	622.304	943.529	761.687	72.961	211.252	105.610
AMK58_17825	K03595 GTPase | (GenBank) GTPase Era	Ribosome	70.159	85.429	83.734	301.458	199.333	173.733
AMK58_17830	K03685 ribonuclease III [EC:3.1.26.3] | (GenBank) ribonuclease	Genetic Information Processing	15.498	19.289	24.405	180.134	91.636	86.073
AMK58_17835	K03100 signal peptidase I [EC:3.4.21.89] | (GenBank) S26 family signal peptidase	Quorum sensing	82.287	74.118	85.107	356.346	536.430	283.951
AMK58_17840	K00997 holo-[acyl-carrier protein] synthase [EC:2.7.8.7] | (GenBank) 4'-phosphopantetheinyl transfer	Metabolism: Co-Factors and Vitamins	246.132	250.636	245.385	232.253	327.400	490.934
AMK58_17845	K03474 pyridoxine 5-phosphate synthase [EC:2.6.99.2] | (GenBank) pyridoxine 5'-phosphate synthase	Metabolism: Co-Factors and Vitamens	55.955	65.323	59.318	178.340	82.596	188.160
AMK58_17850	K02503 histidine triad (HIT) family protein | (GenBank) histidine triad (HIT) protein	No COG	329.163	241.119	259.086	126.911	327.525	338.667
AMK58_17855	K00951 GTP pyrophosphokinase [EC:2.7.6.5] | (GenBank) GTP pyrophosphokinase	Metabolism: Nucleotide	11.985	7.327	10.865	47.790	31.392	25.121
AMK58_17860	K03060 DNA-directed RNA polymerase subunit omega [EC:2.7.7.6] | (GenBank) DNA-directed RNA po	Transcription	113.220	105.186	102.500	245.274	275.535	149.442
AMK58_17865	no KO assigned | (GenBank) hypothetical protein	Hypothetical	37.030	55.930	47.274	63.537	120.192	166.668
AMK58_17870	no KO assigned | (GenBank) hypothetical protein	Hypothetical	228.657	244.654	229.563	151.564	731.684	416.045
AMK58_17875	no KO assigned | (GenBank) histidine kinase	Signal Transduction	412.143	668.034	425.425	79.104	1015.298	211.903
AMK58_17880	no KO assigned | (GenBank) nitrogenase accessory factor	Nitrogen	1619.712	1575.308	1669.651	211.442	1072.280	307.148
AMK58_17885	no KO assigned | (GenBank) transcriptional regulator	Transcription	95.681	98.886	123.742	363.248	234.723	323.774
AMK58_17890	K09945 uncharacterized protein | (GenBank) hypothetical protein	Hypothetical	131.818	158.722	185.515	83.232	246.241	184.155
AMK58_17895	K01338 ATP-dependent Lon protease [EC:3.4.21.53] | (GenBank) DNA-binding protein	Peptidase	18.321	18.500	22.621	37.539	32.602	46.468
AMK58_17900	no KO assigned | (GenBank) heat-shock protein Hsp20	Stress Response	104.160	92.192	116.248	217.043	158.775	327.728
AMK58_17905	no KO assigned | (GenBank) hypothetical protein	Hypothetical	353.135	293.863	290.196	534.707	1573.578	476.561
AMK58_17910	no KO assigned | (GenBank) hypothetical protein	Hypothetical	56.213	60.280	106.896	1829.740	1123.849	1411.390
AMK58_17915	no KO assigned | (GenBank) universal stress protein UspA	stress Response	1.901	6.989	5.005	164.928	72.978	56.542
AMK58_17920	K12686 outer membrane lipase/esterase | (GenBank) hypothetical protein	Secretion system	1.004	1.599	2.864	30.393	20.011	28.300
AMK58_17925	K12262 cytochrome b561 | (GenBank) hypothetical protein	Energy	124.851	114.214	140.252	153.334	37.140	94.762
AMK58_17930	K07315 phosphoserine phosphatase RsbU/P [EC:3.1.3.3] | (GenBank) protein phosphatase	Transcription	203.018	186.519	257.414	95.142	27.026	79.908
AMK58_17935	K03799 heat shock protein HtpX [EC:3.4.24.-] | (GenBank) peptidase M48	Stress response	4.867	7.092	10.835	327.552	119.148	263.944
AMK58_17940	no KO assigned | (GenBank) molecular chaperone DnaJ	Chaperone	9.723	9.549	13.044	95.813	87.285	124.250
AMK58_17945	K18997 chaperone modulatory protein CbpM | (GenBank) hypothetical protein	Chaperone	215.900	180.349	239.285	739.286	642.478	526.055
AMK58_17950	no KO assigned | (GenBank) hypothetical protein	Hypothetical	99.004	90.419	149.894	1005.215	1663.598	848.455
AMK58_17955	K00384 thioredoxin reductase (NADPH) [EC:1.8.1.9] | (GenBank) pyridine nucleotide-disulfide oxidored	Energy	25.517	31.890	37.878	224.968	1129.574	249.891
AMK58_17960	no KO assigned | (GenBank) hypothetical protein	Hypothetical	41.290	42.659	53.132	168.424	845.174	202.949
AMK58_17965	K03413 two-component system, chemotaxis family, response regulator CheY | (GenBank) hypothetical pro	Chemotaxis	74.951	88.027	78.573	214.532	374.436	163.058
AMK58_17970	no KO assigned | (GenBank) hypothetical protein	Hypothetical	12.076	15.189	18.431	484.855	548.659	528.345
AMK58_17975	K07393 putative glutathione S-transferase | (GenBank) glutathionyl-hydroquinone reductase YqjG	No COG	0.639	0.000	0.850	35.243	22.826	40.502
AMK58_17980	K03809 NAD(P)H dehydrogenase (quinone) [EC:1.6.5.2] | (GenBank) NAD(P)H:quinone oxidoreductase	Energy	94.972	113.961	88.964	78.807	39.045	178.552
AMK58_17985	no KO assigned | (GenBank) LysR family transcriptional regulator	Transcription	136.548	157.151	122.230	32.817	48.843	87.759
AMK58_17990	no KO assigned | (GenBank) methyltransferase	Methyltransferase	163.451	131.567	147.819	101.307	131.853	164.337
AMK58_17995	no KO assigned | (GenBank) lytic transglycosylase	transglycosylase	383.222	368.194	420.893	188.243	229.478	230.945
AMK58_18000	K07115 23S rRNA (adenine2030-N6)-methyltransferase [EC:2.1.1.266] | (GenBank) lactate dehydrogenas	Ribosome	85.905	76.641	83.735	114.496	111.953	252.874
AMK58_18005	no KO assigned | (GenBank) hypothetical protein	Hypothetical	7.658	10.168	11.443	158.929	43.861	74.846
AMK58_18010	no KO assigned | (GenBank) hypothetical protein	Hypothetical	1176.431	1290.778	1317.664	86.111	80.135	196.304
AMK58_18015	no KO assigned | (GenBank) protein tyrosine phosphatase	Phosphatase	73.803	82.605	86.872	230.764	144.914	343.046
AMK58_18020	no KO assigned | (GenBank) hypothetical protein	Hypothetical	96.642	91.893	84.317	59.988	67.932	90.245
AMK58_18025	K20975 two-component system, sensor histidine kinase [EC:2.7.13.3] | (GenBank) histidine kinase	Signal Transduction	52.869	46.784	46.171	60.703	26.875	74.889
AMK58_18030	K11085 ATP-binding cassette, subfamily B, bacterial MsbA [EC:3.6.3.-] | (GenBank) ABC transporter pe	Membrane Transport	28.683	32.660	27.528	35.167	71.460	82.606
AMK58_18035	K07001 NTE family protein | (GenBank) lysophospholipase	No COG	51.192	66.859	68.030	230.920	146.932	320.692
AMK58_18040	no KO assigned | (GenBank) hypothetical protein	Hypothetical	2112.082	2328.516	2164.236	981.868	1038.629	1504.414
AMK58_18045	no KO assigned | (GenBank) GCN5 family acetyltransferase	Acetyltransferase	225.733	185.266	198.369	487.779	205.293	587.777
AMK58_18050	no KO assigned | (GenBank) hypothetical protein	Hypothetical	415.690	478.218	553.210	980.324	285.796	971.133
AMK58_18055	no KO assigned | (GenBank) hypothetical protein	Hypothetical	33.020	43.840	43.170	67.168	21.651	91.999
AMK58_18060	K11105 cell volume regulation protein A | (GenBank) potassium transporter	Transport	12.492	13.013	14.131	41.619	24.914	75.381
AMK58_18065	no KO assigned | (GenBank) diguanylate cyclase	cyclic nucleotide	2.504	3.479	2.602	20.283	40.520	12.636
AMK58_18070	K03089 RNA polymerase sigma-32 factor | (GenBank) RNA polymerase subunit sigma-70	Transcription	2.211	3.303	6.176	199.029	47.850	141.362
AMK58_18075	no KO assigned | (GenBank) regulator	Signal transduction	224.392	231.474	252.341	945.003	685.910	986.476
AMK58_18080	no KO assigned | (GenBank) hypothetical protein	Hypothetical	481.185	622.472	642.776	238.226	699.850	516.929
AMK58_18085	no KO assigned | (GenBank) hypothetical protein	Hypothetical	1353.739	1767.670	1649.276	162.544	4504.294	567.049

AMK58_18090	K09767 cyclic-di-GMP-binding protein | (GenBank) nucleotide-binding protein	cyclic nucleotide	25.502	38.461	23.071	89.219	116.055	190.790
AMK58_18095	no KO assigned | (GenBank) hypothetical protein	Hypothetical	79.364	137.397	96.468	364.239	180.697	339.736
AMK58_18100	no KO assigned | (GenBank) hypothetical protein	Hypothetical	68.152	121.697	115.481	143.315	78.960	167.698
AMK58_18105	no KO assigned | (GenBank) hypothetical protein	Hypothetical	162.174	163.695	121.808	88.781	135.053	191.579
AMK58_18110	K07216 hemerythrin | (GenBank) hemerythrin	hemerythrin	5.303	10.560	10.644	331.869	165.563	292.010
AMK58_18115	no KO assigned | (GenBank) hypothetical protein	Hypothetical	13.873	25.117	17.987	166.691	393.158	549.654
AMK58_18120	K12372 dipeptide transport system ATP-binding protein | (GenBank) peptide ABC transporter ATP-bindin	Membrane transport	4.937	4.424	8.332	96.882	171.469	167.661
AMK58_18125	K12371 dipeptide transport system ATP-binding protein | (GenBank) dppD; peptide ABC transporter ATP	Transport: Amino Acid	82.062	111.061	91.863	68.806	102.689	138.806
AMK58_18130	K12370 dipeptide transport system permease protein | (GenBank) peptide transporter	Transport: Amino Acid	8.359	7.023	8.445	29.218	62.688	80.781
AMK58_18135	K12369 dipeptide transport system permease protein | (GenBank) peptide ABC transporter permease	chemotaxis	36.167	36.490	40.081	157.855	193.977	164.018
AMK58_18140	K12368 dipeptide transport system substrate-binding protein | (GenBank) peptide ABC transporter subs	Hypothetical	24.563	22.125	26.875	76.132	118.848	103.299
AMK58_18145	no KO assigned | (GenBank) hypothetical protein	Hypothetical	107.876	71.274	134.426	361.433	457.924	368.348
AMK58_18150	no KO assigned | (GenBank) SAM-dependent methyltransferase	methyltransferase	166.181	125.760	153.040	108.760	726.898	297.184
AMK58_18155	no KO assigned | (GenBank) DNA-binding protein	No COG	3374.467	3083.779	2693.610	114.424	443.070	392.100
AMK58_18160	no KO assigned | (GenBank) hypothetical protein	Hypothetical	47.443	52.154	58.702	104.565	56.030	137.140
AMK58_18165	K01712 urocanate hydratase [EC:4.2.1.49] | (GenBank) urocanate hydratase	Metabolism: Amino Acid	28.244	45.397	33.193	53.198	43.269	38.487
AMK58_18170	no KO assigned | (GenBank) hypothetical protein	Hypothetical	12.418	20.234	17.174	224.565	221.429	127.448
AMK58_18175	no KO assigned | (GenBank) hypothetical protein	Hypothetical	157.346	158.711	192.351	734.969	480.633	537.663
AMK58_18180	K01468 imidazolonepropionase [EC:3.5.2.7] | (GenBank) imidazolonepropionase	Metabolism: Amino Acid	296.658	331.017	313.332	342.659	300.525	232.671
AMK58_18185	K03478 chitin disaccharide deacetylase [EC:3.5.1.105] | (GenBank) hypothetical protein	Hydrolases	77.261	75.969	65.444	41.102	81.097	97.147
AMK58_18190	no KO assigned | (GenBank) hypothetical protein	Hypothetical	70.469	76.291	65.354	55.751	60.781	53.504
AMK58_18195	no KO assigned | (GenBank) hypothetical protein	Hypothetical	141.413	212.640	147.147	990.994	183.958	320.307
AMK58_18200	K08295 2-aminobenzoate-CoA ligase [EC:6.2.1.32] | (GenBank) 2-aminobenzoate-CoA ligase	Metabolism	14.118	14.573	18.484	137.927	38.302	93.010
AMK58_18205	no KO assigned | (GenBank) hypothetical protein	Hypothetical	773.113	881.590	895.529	318.290	464.134	258.454
AMK58_18210	no KO assigned | (GenBank) hypothetical protein	Hypothetical	103.464	141.748	128.375	135.319	101.451	71.671
AMK58_18215	no KO assigned | (GenBank) regulator	Signal transduction	245.043	305.720	239.650	333.756	275.781	344.576
AMK58_18220	K01433 formyltetrahydrofolate deformylase [EC:3.5.1.10] | (GenBank) formyltetrahydrofolate deformyla	Metabolism	54.463	60.071	50.721	105.505	55.097	104.241
AMK58_18225	no KO assigned | (GenBank) hypothetical protein	Hypothetical	99.634	97.954	95.932	123.892	349.684	398.401
AMK58_18230	no KO assigned | (GenBank) hypothetical protein	Hypothetical	230.552	248.047	301.029	299.826	381.426	425.850
AMK58_18235	no KO assigned | (GenBank) hypothetical protein	Hypothetical	226.718	216.297	189.231	195.842	216.148	357.722
AMK58_18240	no KO assigned | (GenBank) hypothetical protein	Hypothetical	25.224	32.084	23.364	63.436	31.462	143.959
AMK58_18245	K01448 N-acetylmuramoyl-L-alanine amidase [EC:3.5.1.28] | (GenBank) N-acetylmuramoyl-L-alanine ami	Defense	17.145	14.503	14.570	80.890	17.566	125.213
AMK58_18250	no KO assigned | (GenBank) hypothetical protein	Hypothetical	11.383	11.129	11.610	131.967	27.451	201.702
AMK58_18255	no KO assigned | (GenBank) hypothetical protein	Hypothetical	10.505	9.938	9.989	92.175	15.942	113.163
AMK58_18260	no KO assigned | (GenBank) hypothetical protein	Hypothetical	32.287	38.691	35.874	185.697	115.943	137.160
AMK58_18265	no KO assigned | (GenBank) hypothetical protein	Hypothetical	15.210	8.425	13.057	19.700	21.248	29.142
AMK58_18270	no KO assigned | (GenBank) hypothetical protein	Hypothetical	377.118	438.757	366.745	1670.603	570.402	1031.899
AMK58_18275	no KO assigned | (GenBank) hypothetical protein	Hypothetical	5.451	8.006	7.990	56.374	9.292	23.292
AMK58_18280	K06601 flagellar protein FlbT | (GenBank) flagellar protein	Motility	100.611	132.445	129.441	1795.466	212.126	1182.521
AMK58_18285	no KO assigned | (GenBank) flagellin	motility	161.238	346.947	223.452	15.732	69.724	47.772
AMK58_18290	K00648 3-oxoacyl-[acyl-carrier-protein] synthase III [EC:2.3.1.180] | (GenBank) 3-oxoacyl-ACP syntha	Metabolism: Lipid	114.874	144.931	154.733	1085.611	210.559	871.426
AMK58_18295	K00059 3-oxoacyl-[acyl-carrier protein] reductase [EC:1.1.1.100] | (GenBank) short-chain dehydrogena	Metabolism: Lipid	214.858	223.331	223.790	56.056	108.949	185.276
AMK58_18300	no KO assigned | (GenBank) AMP-binding protein	Metabolism	4066.575	4879.175	4072.729	138.204	57.985	285.598
AMK58_18305	no KO assigned | (GenBank) hypothetical protein	Hypothetical	132.194	159.926	164.847	647.640	178.680	616.338
AMK58_18310	K00627 pyruvate dehydrogenase E2 component (dihydrolipoamide acetyltransferase) [EC:2.3.1.12] | (Gen	Metabolism: Carbon	26.019	33.562	33.032	256.052	26.089	105.279
AMK58_18315	K02078 acyl carrier protein | (GenBank) hypothetical protein	Lipid metabolism	180.060	225.662	219.415	1429.176	184.366	653.467
AMK58_18320	K07257 spore coat polysaccharide biosynthesis protein SpsF | (GenBank) spore coat protein	Biosynthesis: polysachharide	29.990	34.309	29.879	22.864	17.034	54.434
AMK58_18325	no KO assigned | (GenBank) pseudaminic acid synthase	No COG	67.724	76.251	83.305	145.397	24.427	92.225
AMK58_18330	no KO assigned | (GenBank) hypothetical protein	Hypothetical	74.618	86.821	74.169	5.648	6.686	13.754
AMK58_18335	no KO assigned | (GenBank) hypothetical protein	Hypothetical	6.963	10.307	7.850	79.071	5.264	28.624
AMK58_18340	K00966 mannose-1-phosphate guanylyltransferase [EC:2.7.7.13] | (GenBank) nucleotidyl transferase	Hypothetical	76.714	78.712	78.173	347.382	44.427	222.529
AMK58_18345	no KO assigned | (GenBank) acetyltransferase	Acetyltransferase	78.962	79.936	86.548	322.021	119.821	377.893
AMK58_18350	no KO assigned | (GenBank) short-chain dehydrogenase	dehydrogenase	16.489	22.517	22.845	623.501	64.605	166.730
AMK58_18355	no KO assigned | (GenBank) (2Fe-2S)-binding protein	No COG	34.133	44.248	41.026	152.409	29.559	101.222
AMK58_18360	K00648 3-oxoacyl-[acyl-carrier-protein] synthase III [EC:2.3.1.180] | (GenBank) 3-oxoacyl-ACP syntha	Lipid metabolism	24.819	20.038	16.689	89.255	24.253	140.052
AMK58_18365	no KO assigned | (GenBank) short-chain dehydrogenase	dehydrogenase	24.498	20.640	30.609	171.442	29.125	114.745
AMK58_18370	no KO assigned | (GenBank) hypothetical protein	Hypothetical	84.660	90.073	93.286	174.769	95.031	225.400
AMK58_18375	no KO assigned | (GenBank) hypothetical protein	Hypothetical	107.981	113.594	130.468	255.893	114.722	270.778
AMK58_18380	K00426 cytochrome d ubiquinol oxidase subunit II [EC:1.10.3.14] | (GenBank) ubiquinol oxidase subuni	Energy	6.846	6.459	11.992	125.984	37.129	55.801
AMK58_18385	K00425 cytochrome d ubiquinol oxidase subunit I [EC:1.10.3.14] | (GenBank) cytochrome D ubiquinol ox	Energy	7.299	7.695	11.267	144.242	43.048	45.624
AMK58_18390	K03089 RNA polymerase sigma-32 factor | (GenBank) RNA polymerase subunit sigma-70	Transcription	20.179	24.916	24.559	105.241	97.836	55.386
AMK58_18395	no KO assigned | (GenBank) hypothetical protein	Hypothetical	9.375	7.594	14.201	108.762	204.140	297.176
AMK58_18400	K18480 cholesterol transport system auxiliary component | (GenBank) hypothetical protein	Transport: Lipid	15.271	15.206	21.434	163.891	229.478	545.165
AMK58_18405	K02067 phospholipid/cholesterol/gamma-HCH transport system substrate-binding protein | (GenBank) AB	Transport: phospholipid	439.905	496.553	539.740	135.831	4581.804	422.532
AMK58_18410	K02065 phospholipid/cholesterol/gamma-HCH transport system ATP-binding protein | (GenBank) ABC tr	Transport: Lipid	140.416	128.293	137.129	182.587	682.520	220.162
AMK58_18415	K02066 phospholipid/cholesterol/gamma-HCH transport system permease protein | (GenBank) ABC transp	Transport: phospholipid	27.178	28.080	26.424	140.698	95.897	108.480
AMK58_18420	K00759 adenine phosphoribosyltransferase [EC:2.4.2.7] | (GenBank) adenine phosphoribosyltransferase	Metabolism: Nucleotide	89.633	113.550	82.993	197.703	283.527	380.977

AMK58_18425 K06978 uncharacterized protein | (GenBank) X-Pro dipeptidyl-peptidase	peptidase	10.704	10.803	14.098	96.498	35.040	87.254
AMK58_18430 no KO assigned | (GenBank) cell filamentation protein Fic	Cell Cycle/Shape/Homeostatsis	77.716	62.745	81.025	207.606	140.309	285.752
AMK58_18435 K07216 hemerythrin | (GenBank) hemerythrin	hemerythrin	130.170	103.579	124.847	214.344	97.438	231.655
AMK58_18440 no KO assigned | (GenBank) hypothetical protein	Hypothetical	15.567	17.223	21.562	352.690	100.882	184.265
AMK58_18445 no KO assigned | (GenBank) hypothetical protein	Hypothetical	60.918	94.021	71.919	287.650	120.401	236.072
AMK58_18450 no KO assigned | (GenBank) hypothetical protein	Hypothetical	600.265	814.920	569.766	123.179	378.994	541.926
AMK58_18455 no KO assigned | (GenBank) hypothetical protein	Hypothetical	11.437	17.845	18.078	68.895	160.577	101.056
AMK58_18460 no KO assigned | (GenBank) hypothetical protein	Hypothetical	58.256	68.142	60.062	70.986	68.441	189.231
AMK58_18465 K07313 serine/threonine protein phosphatase 1 [EC:3.1.3.16] | (GenBank) metallophosphoesterase	Hydrolase	2.895	5.087	2.752	80.751	3.445	43.266
AMK58_18470 no KO assigned | (GenBank) transcriptional regulator	Transcription	37.017	35.130	42.947	235.760	96.888	309.051
AMK58_18475 no KO assigned | (GenBank) aldo/keto reductase	enzyme	4.833	8.663	9.191	185.170	25.127	123.026
AMK58_18480 K00873 pyruvate kinase [EC:2.7.1.40] | (GenBank) pyruvate kinase	Metabolism	32.252	42.310	49.893	101.038	168.030	92.806
AMK58_18485 K07483 transposase | (GenBank) hypothetical protein	Hypothetical	31.621	17.400	55.779	812.044	478.898	518.138
AMK58_18490 no KO assigned | (GenBank) LysR family transcriptional regulator	Transcription	24.566	25.805	25.411	151.518	321.799	242.187
AMK58_18495 K20276 large repetitive protein | (GenBank) hypothetical protein	Quorum sensing	24.759	24.805	24.339	11.748	86.043	18.983
AMK58_18500 no KO assigned | (GenBank) hypothetical protein	Hypothetical	706.455	741.052	672.055	382.898	481.945	1172.480
AMK58_18505 K01951 GMP synthase (glutamine-hydrolysing) [EC:6.3.5.2] | (GenBank) guaA; GMP synthetase	Metabolism: Nucleotide	59.405	66.692	76.301	1288.912	309.215	1564.464
AMK58_18510 no KO assigned | (GenBank) GNAT family acetyltransferase	Acetyltransferase	12.802	5.185	7.840	22.671	63.169	107.642
AMK58_18515 no KO assigned | (GenBank) purine nucleoside permease	Metabolism: Nucleotide	335.686	269.485	318.044	213.227	278.724	254.918
AMK58_18520 no KO assigned | (GenBank) hypothetical protein	Hypothetical	168.409	206.886	194.470	298.676	417.801	256.649
AMK58_18525 no KO assigned | (GenBank) hypothetical protein	Hypothetical	264.228	176.584	202.361	142.151	58.440	211.111
AMK58_18530 K03500 16S rRNA (cytosine967-C5)-methyltransferase [EC:2.1.1.176] | (GenBank) rRNA cytosine-C5-m	Ribosome	5.753	6.345	6.059	40.309	7.561	95.692
AMK58_18535 K08304 membrane-bound lytic murein transglycosylase A | (GenBank) murein transglycosylase	Cell Wall	16.496	26.033	22.590	83.664	16.093	202.797
AMK58_18540 no KO assigned | (GenBank) GCN5 family acetyltransferase	Acetyltransferase	149.769	165.856	163.030	594.994	87.058	284.460
AMK58_18545 K09984 uncharacterized protein | (GenBank) hypothetical protein	Hypothetical	216.379	180.113	213.594	767.940	148.664	513.555
AMK58_18550 K03437 RNA methyltransferase, TrmH family | (GenBank) RNA methyltransferase	tRNA synthesis/modification	13.665	15.189	15.712	77.049	52.866	126.709
AMK58_18555 K00088 IMP dehydrogenase [EC:1.1.1.205] | (GenBank) inosine-5'-monophosphate dehydrogenase	Metabolism: Nucleotide	6.713	8.804	8.173	32.898	9.570	41.143
AMK58_18560 K02427 23S rRNA (uridine2552-2'-O)-methyltransferase [EC:2.1.1.166] | (GenBank) 23S rRNA methyltr	Ribosome	28.098	25.434	26.553	211.458	31.583	161.220
AMK58_18565 K01524 exopolyphosphatase / guanosine-5'-triphosphate,3'-diphosphate pyrophosphatase [EC:3.6.1.11 3.	Metabolism: Nucleotide	323.807	302.980	288.748	172.746	357.757	224.539
AMK58_18570 K14223 tRNA Gln | (GenBank) tRNA-Gln	tRNA synthesis/modification	1420.175	1632.438	1855.904	2857.978	1537.485	1964.719
AMK58_18575 no KO assigned | (GenBank) hypothetical protein	Hypothetical	602.069	791.950	791.939	242.580	279.812	381.168
AMK58_18580 no KO assigned | (GenBank) transposase	Genetic Information Processing	0.198	0.592	0.660	6.774	2.787	18.022
AMK58_18585 K01689 enolase [EC:4.2.1.11] | (GenBank) phosphopyruvate hydratase	Metabolism: Carbon	5.860	2.259	6.740	62.292	69.056	88.343
AMK58_18590 no KO assigned | (GenBank) hypothetical protein	Hypothetical	7.693	10.606	9.340	89.575	69.044	267.669
AMK58_18595 no KO assigned | (GenBank) GntR family transcriptional regulator	Transcription	16.030	23.661	22.952	254.647	73.134	286.095
AMK58_18600 no KO assigned | (GenBank) phytanoyl-CoA dioxygenase	No COG	8.391	12.732	10.005	109.016	82.195	79.293
AMK58_18605 no KO assigned | (GenBank) C4-dicarboxylate ABC transporter permease	Membrane Transport	29.076	30.140	32.007	64.383	130.629	90.921
AMK58_18610 no KO assigned | (GenBank) hypothetical protein	Hypothetical	75.292	63.520	80.666	286.005	94.875	351.899
AMK58_18615 no KO assigned | (GenBank) C4-dicarboxylate ABC transporter substrate-binding protein	Membrane Transport	21.046	22.108	24.629	126.248	53.033	207.275
AMK58_18620 no KO assigned | (GenBank) alcohol dehydrogenase	dehydrogenase	18.034	12.231	15.479	34.474	30.900	95.004
AMK58_18625 no KO assigned | (GenBank) hypothetical protein	Hypothetical	69.554	67.547	83.414	232.543	110.837	285.186
AMK58_18630 no KO assigned | (GenBank) hypothetical protein	Hypothetical	86.957	82.030	96.921	763.556	153.397	655.099
AMK58_18635 no KO assigned | (GenBank) hypothetical protein	Hypothetical	9.025	16.278	10.686	160.361	19.715	52.582
AMK58_18640 no KO assigned | (GenBank) fatty acid hydroxylase	hydroxylase	1.943	4.465	1.563	24.628	3.864	14.969
AMK58_18645 no KO assigned | (GenBank) hypothetical protein	Hypothetical	14.785	14.324	21.276	348.459	30.542	227.504
AMK58_18650 no KO assigned | (GenBank) hypothetical protein	Hypothetical	130.761	115.737	110.514	247.820	260.210	809.539
AMK58_18655 no KO assigned | (GenBank) hypothetical protein	Hypothetical	46.217	42.779	55.703	172.649	51.950	472.016
AMK58_18660 K01207 beta-N-acetylhexosaminidase [EC:3.2.1.52] | (GenBank) hypothetical protein	Defense	1.787	2.248	4.293	78.888	4.336	52.776
AMK58_18665 K06143 inner membrane protein | (GenBank) hypothetical protein	Membrane	1.762	1.053	1.927	11.214	3.863	13.237
AMK58_18670 no KO assigned | (GenBank) histidine kinase	Signal Transduction	0.494	2.460	2.349	162.407	11.665	52.011
AMK58_18675 no KO assigned | (GenBank) hypothetical protein	Hypothetical	11.119	14.762	17.229	766.246	100.913	717.533
AMK58_18680 K14230 tRNA Met | (GenBank) tRNA-Met	tRNA synthesis/modification	20.441	37.577	56.812	2582.336	316.372	1344.320
AMK58_18685 K01985 5S ribosomal RNA | (GenBank) 5S ribosomal RNA	Ribosome	1.044	6.236	6.947	483.761	76.259	473.275
AMK58_18690 K01980 23S ribosomal RNA | (GenBank) 23S ribosomal RNA	Ribosome	8.576	8.934	7.067	4.065	45.974	8.442
AMK58_18695 K14218 tRNA Ala | (GenBank) tRNA-Ala	tRNA synthesis/modification	0.000	0.000	0.000	0.000	0.000	0.000
AMK58_18700 K14227 tRNA Ile | (GenBank) tRNA-Ile	tRNA synthesis/modification	0.000	0.000	0.000	0.000	0.000	0.000
AMK58_18705 K01977 16S ribosomal RNA | (GenBank) 16S ribosomal RNA	Ribosome	0.000	0.000	0.000	0.000	0.000	0.000
AMK58_18710 no KO assigned | (GenBank) hypothetical protein	Hypothetical	0.000	0.000	0.000	0.000	0.000	0.000
AMK58_18715 no KO assigned | (GenBank) acyl carrier protein	No COG	0.000	0.000	0.000	0.000	0.000	0.000
AMK58_18720 no KO assigned | (GenBank) hypothetical protein	Hypothetical	34.843	27.327	41.315	33.338	67.744	110.274
AMK58_18725 K02017 molybdate transport system ATP-binding protein [EC:3.6.3.29] | (GenBank) molybdenum ABC tr	Transport: organic	110.985	145.731	110.691	44.111	24.053	144.964
AMK58_18730 K02018 molybdate transport system permease protein | (GenBank) molybdenum ABC transporter permease	Membrane Transport	138.630	160.025	178.787	281.704	44.958	242.664
AMK58_18735 K02020 molybdate transport system substrate-binding protein | (GenBank) molybdate ABC transporter su	Membrane Transport	583.979	791.140	935.438	385.599	58.482	164.121
AMK58_18740 K02019 molybdate transport system regulatory protein | (GenBank) molybdenum-dependent transcriptiona	Transcription	748.004	914.241	1027.495	116.520	33.851	111.703
AMK58_18745 K09797 uncharacterized protein | (GenBank) hypothetical protein	Hypothetical	2203.660	2620.682	3071.254	150.886	63.956	236.349
AMK58_18750 no KO assigned | (GenBank) hypothetical protein	Hypothetical	252.303	356.303	395.706	163.563	24.705	147.548
AMK58_18755 no KO assigned | (GenBank) hypothetical protein	Hypothetical	46.196	51.560	70.712	427.735	47.647	312.096

AMK58_18760	no KO assigned | (GenBank) hypothetical protein	Hypothetical	538.544	466.681	530.620	235.431	107.314	492.940
AMK58_18765	K11902 type VI secretion system protein ImpA | (GenBank) hypothetical protein	Secretion System	89.637	76.690	102.811	40.364	21.276	102.384
AMK58_18770	K11891 type VI secretion system protein ImpL | (GenBank) hypothetical protein	Secretion System	18.994	19.933	21.955	13.290	3.520	31.616
AMK58_18775	K11892 type VI secretion system protein ImpK | (GenBank) hypothetical protein	Secretion System	67.173	72.744	106.013	278.557	25.681	147.536
AMK58_18780	K11893 type VI secretion system protein ImpJ | (GenBank) hypothetical protein	Secretion System	102.517	117.330	159.488	458.803	34.935	179.682
AMK58_18785	K11918 type VI secretion system protein | (GenBank) hypothetical protein	Secretion System	62.421	75.550	99.514	199.308	12.097	109.276
AMK58_18790	no KO assigned | (GenBank) hypothetical protein	Hypothetical	153.554	185.402	221.065	259.325	14.459	149.693
AMK58_18795	no KO assigned | (GenBank) hypothetical protein	Hypothetical	32.000	35.668	61.760	159.627	2.147	114.484
AMK58_18800	no KO assigned | (GenBank) hypothetical protein	Hypothetical	22.399	40.990	43.361	188.406	4.763	64.555
AMK58_18805	no KO assigned | (GenBank) hypothetical protein	Hypothetical	2.870	2.500	3.695	37.803	1.479	12.696
AMK58_18810	no KO assigned | (GenBank) hypothetical protein	Hypothetical	4.173	8.930	7.698	41.631	1.470	19.751
AMK58_18815	no KO assigned | (GenBank) hypothetical protein	Hypothetical	42.288	51.874	76.287	296.156	10.663	143.729
AMK58_18820	no KO assigned | (GenBank) hypothetical protein	Hypothetical	122.612	112.522	190.038	1161.045	34.995	301.881
AMK58_18825	no KO assigned | (GenBank) hypothetical protein	Hypothetical	20.077	28.151	33.309	155.874	8.750	71.997
AMK58_18830	K11901 type VI secretion system protein ImpB | (GenBank) type VI secretion protein	Secretion System	3.382	4.041	3.644	124.433	7.350	49.549
AMK58_18835	K11900 type VI secretion system protein ImpC | (GenBank) EvpB family type VI secretion protein	Secretion System	20.426	24.112	27.174	72.708	8.610	59.495
AMK58_18840	no KO assigned | (GenBank) hypothetical protein	Hypothetical	2126.552	2149.039	3027.450	164.700	29.803	215.619
AMK58_18845	K11897 type VI secretion system protein ImpF | (GenBank) hypothetical protein	Secretion System	5979.881	5228.962	8402.679	442.036	162.482	664.813
AMK58_18850	K11896 type VI secretion system protein ImpG | (GenBank) hypothetical protein	Secretion System	1049.921	861.023	1301.670	18.773	29.157	71.535
AMK58_18855	K11895 type VI secretion system protein ImpH | (GenBank) hypothetical protein	Secretion System	149.150	135.629	218.142	82.709	11.218	104.744
AMK58_18860	K11907 type VI secretion system protein VasG | (GenBank) ClpV1 family T6SS ATPase	Secretion System	18.374	23.227	36.965	146.562	6.971	91.841
AMK58_18865	K11904 type VI secretion system secreted protein VgrG | (GenBank) hypothetical protein	Secretion System	11.548	11.498	20.074	129.139	6.162	51.322
AMK58_18870	no KO assigned | (GenBank) hypothetical protein	Hypothetical	91.261	94.492	159.542	836.256	36.169	281.180
AMK58_18875	no KO assigned | (GenBank) hypothetical protein	Hypothetical	272.033	265.046	349.458	622.744	253.140	1190.116
AMK58_18880	K02346 DNA polymerase IV [EC:2.7.7.7] | (GenBank) DNA polymerase IV	DNA Repair and Replication	19.675	21.701	33.001	80.687	26.652	118.257
AMK58_18885	K07167 putative transcriptional regulator | (GenBank) hypothetical protein	Transcription	54.428	71.893	106.846	118.044	85.735	181.623
AMK58_18890	K03088 RNA polymerase sigma-70 factor, ECF subfamily | (GenBank) RNA polymerase subunit sigma	Transcription	24.722	35.347	34.154	535.242	142.743	280.184
AMK58_18895	no KO assigned | (GenBank) Fis family transcriptional regulator	Transcription	16.261	26.176	25.555	106.882	21.782	57.580
AMK58_18900	K00656 formate C-acetyltransferase [EC:2.3.1.54] | (GenBank) formate acetyltransferase	Metabolism: Carbohydrate	22.486	21.229	26.574	25.006	19.851	58.225
AMK58_18905	K09857 uncharacterized protein | (GenBank) hypothetical protein	Hypothetical	191.306	245.846	208.042	338.682	749.180	493.106
AMK58_18910	K06192 paraquat-inducible protein B | (GenBank) hypothetical protein	Membrane	350.206	361.246	311.454	133.314	125.443	201.200
AMK58_18915	K03808 paraquat-inducible protein A | (GenBank) paraquat-inducible protein A	No COG	13.832	23.772	21.979	233.761	30.882	121.933
AMK58_18920	no KO assigned | (GenBank) hypothetical protein	Hypothetical	31.562	43.278	46.736	465.765	71.342	382.708
AMK58_18925	no KO assigned | (GenBank) hypothetical protein	Hypothetical	10.143	10.242	11.953	60.856	19.430	44.577
AMK58_18930	K00029 malate dehydrogenase (oxaloacetate-decarboxylating)(NADP+) [EC:1.1.1.40] | (GenBank) malic e	Metabolism	569.245	734.897	569.389	36.577	191.610	95.906
AMK58_18935	K11103 aerobic C4-dicarboxylate transport protein | (GenBank) glutamate:protein symporter	Transport	43.055	56.113	56.587	327.361	30.835	121.927
AMK58_18940	K10125 two-component system, NtrC family, C4-dicarboxylate transport sensor histidine kinase DctB [E	Signal Transduction	624.622	525.515	618.887	198.042	190.996	221.149
AMK58_18945	K10126 two-component system, NtrC family, C4-dicarboxylate transport response regulator DctD | (GenB	Signal Transduction	56.063	53.990	87.340	105.421	50.591	222.343
AMK58_18950	no KO assigned | (GenBank) hypothetical protein	Hypothetical	10.467	9.029	12.560	111.273	25.671	85.850
AMK58_18955	no KO assigned | (GenBank) hypothetical protein	Hypothetical	13.627	15.657	17.941	316.649	48.703	202.183
AMK58_18960	K01847 methylmalonyl-CoA mutase [EC:5.4.99.2] | (GenBank) methylmalonyl-CoA mutase	Metabolism	11.523	16.455	14.858	229.123	27.989	91.838
AMK58_18965	K03523 biotin transport system substrate-specific component | (GenBank) biotin transporter BioY	Membrane transport	92.709	77.158	86.175	270.619	66.880	151.190
AMK58_18970	K01965 propionyl-CoA carboxylase alpha chain [EC:6.4.1.3] | (GenBank) acetyl/propionyl-CoA carboxyla	Metabolism	507.231	424.398	412.207	139.896	114.533	231.777
AMK58_18975	K01966 propionyl-CoA carboxylase beta chain [EC:6.4.1.3] | (GenBank) methylmalonyl-CoA carboxyltrar	Metabolism	313.547	297.269	345.432	104.633	88.687	68.329
AMK58_18980	K07110 uncharacterized protein | (GenBank) Cro/Cl family transcriptional regulator	Transcription	630.775	504.701	494.082	194.645	222.877	325.264
AMK58_18985	K01779 aspartate racemase [EC:5.1.1.13] | (GenBank) aspartate racemase	enzyme	1062.768	852.349	851.943	233.156	556.507	461.245
AMK58_18990	K14233 tRNA Ser | (GenBank) tRNA-Ser	tRNA synthesis/modification	233.133	215.468	258.405	2489.726	870.128	3249.849
AMK58_18995	no KO assigned | (GenBank) hypothetical protein	Hypothetical	238.010	213.297	261.231	518.440	391.413	549.834
AMK58_19000	no KO assigned | (GenBank) PAS domain-containing sensor histidine kinase	Signal Transduction	1.480	1.360	1.840	8.006	28.985	23.172
AMK58_19005	K02434 aspartyl-tRNA(Asn)/glutamyl-tRNA(Gln) amidotransferase subunit B [EC:6.3.5.6 6.3.5.7] | (Genl	tRNA synthesis/modification	14.759	14.447	18.394	25.467	2.718	38.583
AMK58_19010	K02433 aspartyl-tRNA(Asn)/glutamyl-tRNA(Gln) amidotransferase subunit A [EC:6.3.5.6 6.3.5.7] | (Gen	tRNA synthesis/modification	91.174	93.226	85.990	173.130	79.617	289.007
AMK58_19015	K02435 aspartyl-tRNA(Asn)/glutamyl-tRNA(Gln) amidotransferase subunit C [EC:6.3.5.6 6.3.5.7] | (Genl	tRNA synthesis/modification	2106.197	2147.462	1941.033	369.987	1067.008	1057.986
AMK58_19020	no KO assigned | (GenBank) acetyltransferase	Acetyltransferase	794.609	704.602	720.133	378.133	532.276	391.904
AMK58_19025	K07447 putative holliday junction resolvase [EC:3.1.-.-] | (GenBank) Holliday junction resolvase	DNA Repair and Replication	200.496	170.201	172.165	36.716	156.956	141.288
AMK58_19030	K07088 uncharacterized protein | (GenBank) hypothetical protein	Hypothetical	10.675	5.445	13.121	87.894	56.779	168.646
AMK58_19035	no KO assigned | (GenBank) hypothetical protein	Hypothetical	65.662	74.632	79.487	110.055	60.306	192.611
AMK58_19040	no KO assigned | (GenBank) aldehyde-activating protein	Metabolism	39.773	45.428	58.950	460.310	298.813	347.114
AMK58_19045	no KO assigned | (GenBank) aldehyde-activating protein	Metabolism	302.821	328.943	402.789	127.457	656.732	363.823
AMK58_19050	no KO assigned | (GenBank) hypothetical protein	Hypothetical	1352.294	1582.996	1994.839	110.474	1507.544	265.759
AMK58_19055	K03566 LysR family transcriptional regulator, glycine cleavage system transcriptional activator | (G	Transcription	468.816	553.511	593.333	59.502	547.226	86.026
AMK58_19060	no KO assigned | (GenBank) hypothetical protein	Hypothetical	560.946	613.940	786.807	186.718	599.649	104.093
AMK58_19065	no KO assigned | (GenBank) hypothetical protein	Hypothetical	25.552	38.522	29.745	202.492	80.797	321.751
AMK58_19070	K00609 aspartate carbamoyltransferase catalytic subunit [EC:2.1.3.2] | (GenBank) pyrB; aspartate car	Metabolism	28.917	35.304	31.200	81.346	144.996	59.457
AMK58_19075	K01465 dihydroorotase [EC:3.5.2.3] | (GenBank) dihydroorotase	Metabolism: Nucleotide	6.031	5.543	7.410	143.917	85.097	51.188
AMK58_19080	K08591 glycerol-3-phosphate acyltransferase PlsY [EC:2.3.1.15] | (GenBank) glycerol-3-phosphate acyl	Lipid metabolism	471.458	450.575	413.769	181.572	266.917	305.434
AMK58_19085	K03413 two-component system, chemotaxis family, response regulator CheY | (GenBank) hypothetical pro	Chemotaxis	680.810	626.457	688.921	862.465	391.321	794.282
AMK58_19090	no KO assigned | (GenBank) restriction endonuclease subunit R	DNA Repair and Replication	27.965	26.748	25.047	37.461	28.590	35.438

AMK58_19095	no KO assigned | (GenBank) cupin	No COG	146.904	188.072	184.190	107.583	203.233	210.319
AMK58_19100	no KO assigned | (GenBank) hypothetical protein	Hypothetical	10.378	24.112	30.424	1112.904	225.202	716.655
AMK58_19105	K07038 inner membrane protein | (GenBank) metal-dependent hydrolase	Membrane	68.498	59.727	57.031	32.735	23.723	166.345
AMK58_19110	no KO assigned | (GenBank) hypothetical protein	Hypothetical	11.027	12.549	9.843	26.428	9.275	28.102
AMK58_19115	K13483 xanthine dehydrogenase YagT iron-sulfur-binding subunit | (GenBank) oxidoreductase	Metabolism: Nucleotide	74.854	78.128	91.752	151.313	210.602	309.212
AMK58_19120	K11178 xanthine dehydrogenase YagS FAD-binding subunit [EC:1.17.1.4] | (GenBank) FAD-binding mo	Metabolism: Nucleotide	181.067	245.463	237.037	191.896	102.717	258.703
AMK58_19125	K11177 xanthine dehydrogenase YagR molybdenum-binding subunit [EC:1.17.1.4] | (GenBank) aldehyde	Metabolism: Nucleotide	0.593	0.644	1.178	39.626	17.201	45.867
AMK58_19130	K03387 alkyl hydroperoxide reductase subunit F [EC:1.6.4.-] | (GenBank) alkyl hydroperoxide reductas	Oxidoreductase	0.842	1.373	2.913	117.972	17.388	74.491
AMK58_19135	K03386 peroxiredoxin (alkyl hydroperoxide reductase subunit C) [EC:1.11.1.15] | (GenBank) alkyl hydr	Oxidoreductase	30.913	38.476	42.659	623.725	316.344	899.551
AMK58_19140	K04761 LysR family transcriptional regulator, hydrogen peroxide-inducible genes activator | (GenBank	Transcription	255.687	228.269	257.063	287.427	351.681	321.324
AMK58_19145	no KO assigned | (GenBank) transcriptional regulator	Transcription	3692.450	3155.622	3271.559	114.032	734.755	371.574
AMK58_19150	K02048 sulfate transport system substrate-binding protein | (GenBank) ABC transporter permease	Energy	66.192	79.662	82.745	174.944	59.148	155.433
AMK58_19155	K00220 cyclohexadieny/prephenate dehydrogenase [EC:1.3.1.43 1.3.1.12] | (GenBank) cyclohexadienyl de	Metabolism	8.369	8.871	11.294	93.298	30.496	83.608
AMK58_19160	K00817 histidinol-phosphate aminotransferase [EC:2.6.1.9] | (GenBank) histidinol-phosphate aminotran	Metabolism: Amino Acid	5.639	5.285	7.359	149.892	52.200	237.150
AMK58_19165	no KO assigned | (GenBank) chorismate mutase	Metabolism	44.196	32.413	46.299	136.307	31.235	208.098
AMK58_19170	K00641 homoserine O-acetyltransferase [EC:2.3.1.31] | (GenBank) metX; homoserine O-acetyltransferase	Metabolism: Amino Acid	186.907	185.017	189.110	146.931	103.305	174.981
AMK58_19175	no KO assigned | (GenBank) methionine biosynthesis protein MetW	Biosynthesis: Amino Acid	190.074	170.005	204.774	293.977	127.104	218.848
AMK58_19180	no KO assigned | (GenBank) hypothetical protein	Hypothetical	115.764	98.985	109.262	376.726	212.496	302.340
AMK58_19185	K07002 uncharacterized protein | (GenBank) alpha/beta hydrolase	hydrolase	38.856	50.019	36.536	221.424	146.952	214.984
AMK58_19190	no KO assigned | (GenBank) methyltransferase type 11	methyltransferase	4.519	3.724	6.963	94.974	116.651	52.595
AMK58_19195	K01069 hydroxyacylglutathione hydrolase [EC:3.1.2.6] | (GenBank) hydroxyacylglutathione hydrolase	Metabolism: Carbohydrate	60.855	67.399	75.537	129.834	202.890	47.295
AMK58_19200	no KO assigned | (GenBank) glutathione S-transferase	Transferase	299.690	352.129	298.815	354.614	578.059	136.539
AMK58_19205	K09705 uncharacterized protein | (GenBank) cupin	No COG	186.833	185.476	167.982	463.874	389.246	392.028
AMK58_19210	no KO assigned | (GenBank) hypothetical protein	Hypothetical	95.158	113.241	97.220	118.315	54.785	67.075
AMK58_19215	no KO assigned | (GenBank) hypothetical protein	Hypothetical	77.864	65.605	83.254	143.274	80.309	112.402
AMK58_19220	no KO assigned | (GenBank) hypothetical protein	Hypothetical	67.808	70.031	84.646	394.334	91.896	237.060
AMK58_19225	no KO assigned | (GenBank) hypothetical protein	Hypothetical	32.422	48.425	44.474	232.082	62.068	229.080
AMK58_19230	K01486 adenine deaminase [EC:3.5.4.2] | (GenBank) adenosine deaminase	Metabolism: Nucleotide	35.756	44.840	44.432	76.256	37.565	67.165
AMK58_19235	no KO assigned | (GenBank) hypothetical protein	Hypothetical	327.030	419.087	393.780	278.891	207.380	441.059
AMK58_19240	K03406 methyl-accepting chemotaxis protein | (GenBank) hypothetical protein	Chemotaxis	119.894	131.359	114.558	166.633	303.120	203.194
AMK58_19245	K06282 hydrogenase small subunit [EC:1.12.99.6] | (GenBank) hydrogenase 2 small subunit	Metabolism	15.564	26.933	14.084	49.461	10.979	127.498
AMK58_19250	no KO assigned | (GenBank) hydrogenase 2 protein HybA	hydrogenase	230.226	264.408	247.135	295.191	32.090	303.638
AMK58_19255	no KO assigned | (GenBank) hydrogenase	hydrogenase	54.929	63.881	61.895	110.371	15.318	150.471
AMK58_19260	K06281 hydrogenase large subunit [EC:1.12.99.6] | (GenBank) hydrogenase 2 large subunit	Metabolism	10.658	11.037	14.457	79.094	12.140	77.614
AMK58_19265	K03605 hydrogenase maturation protease [EC:3.4.23.-] | (GenBank) hybD; hydrogenase 2 maturation endo	Peptidase	93.833	102.475	107.205	221.992	56.138	475.977
AMK58_19270	no KO assigned | (GenBank) MBL fold metallo-hydrolase	hydrolase	70.958	75.574	87.628	174.935	32.364	314.355
AMK58_19275	no KO assigned | (GenBank) MBL fold metallo-hydrolase	hydrolase	36.250	38.261	43.888	75.590	9.100	100.169
AMK58_19280	K03192 urease accessory protein | (GenBank) urease accessory protein	Enzyme	410.610	386.269	415.230	475.799	37.691	260.659
AMK58_19285	K04653 hydrogenase expression/formation protein HypC | (GenBank) hydrogenase	hydrogenase	90.030	105.986	117.649	909.548	74.776	778.074
AMK58_19290	no KO assigned | (GenBank) hypothetical protein	Hypothetical	141.771	129.833	136.765	224.565	29.661	89.080
AMK58_19295	no KO assigned | (GenBank) hypothetical protein	Hypothetical	5.899	4.946	4.624	33.685	2.559	35.408
AMK58_19300	K04651 hydrogenase nickel incorporation protein HypA/HybF | (GenBank) hydrogenase nickel incorporati	Chaperone	14.161	12.690	20.196	390.764	23.080	220.272
AMK58_19305	K04652 hydrogenase nickel incorporation protein HypB | (GenBank) hydrogenase nickel incorporation pr	Chaperone	2.956	4.607	3.544	194.210	6.501	34.563
AMK58_19310	no KO assigned | (GenBank) hypothetical protein	Hypothetical	8.705	8.274	8.653	98.849	13.787	69.160
AMK58_19315	K04656 hydrogenase maturation protein HypF | (GenBank) carbamoyltransferase	enzyme	17.670	15.628	15.430	40.189	9.481	82.222
AMK58_19320	K04653 hydrogenase expression/formation protein HypC | (GenBank) hydrogenase assembly protein HupI	No COG	48.430	87.405	72.909	379.109	27.219	401.832
AMK58_19325	K04655 hydrogenase expression/formation protein HypE | (GenBank) hydrogenase expression/formation p	No COG	8.095	11.054	13.414	340.017	17.548	233.225
AMK58_19330	no KO assigned | (GenBank) hypothetical protein	Hypothetical	3.014	7.003	2.229	41.724	6.400	34.473
AMK58_19335	K03797 carboxyl-terminal processing protease [EC:3.4.21.102] | (GenBank) carboxyl-terminal protease	Peptidase	4.846	5.491	6.197	126.592	12.022	98.633
AMK58_19340	K03704 cold shock protein (beta-ribbon, CspA family) | (GenBank) cold-shock protein	Stress Response	6.482	13.743	18.888	210.926	68.164	279.802
AMK58_19345	K09858 SEC-C motif domain protein | (GenBank) hypothetical protein	Secretion system	735.687	864.250	896.448	513.914	867.662	546.920
AMK58_19350	no KO assigned | (GenBank) cAMP-binding protein	cyclic nucleotide	1037.161	882.550	833.686	63.246	1092.949	108.946
AMK58_19355	K03470 ribonuclease HII [EC:3.1.26.4] | (GenBank) ribonuclease HII	DNA Repair and Replication	110.851	81.444	92.607	100.195	36.090	194.268
AMK58_19360	K13581 modification methylase [EC:2.1.1.72] | (GenBank) modification methylase	Defense	24.973	32.638	32.225	187.425	21.554	126.846
AMK58_19365	K01633 dihydroneopterin aldolase / 7,8-dihydroneopterin epimerase [EC:4.1.2.25 5.1.99.8] | (GenBank)	Metabolism: Co-Factors and Vitamens	23.021	22.074	29.726	165.993	27.155	194.232
AMK58_19370	no KO assigned | (GenBank) hypothetical protein	Hypothetical	106.821	76.734	75.604	62.524	13.322	62.937
AMK58_19375	no KO assigned | (GenBank) hypothetical protein	Hypothetical	69.976	86.530	84.947	106.380	56.157	89.031
AMK58_19380	K03575 A/G-specific adenine glycosylase [EC:3.2.2.-] | (GenBank) A/G-specific adenine glycosylase	DNA Repair and Replication	34.433	44.083	55.380	429.800	113.223	395.587
AMK58_19385	no KO assigned | (GenBank) hypothetical protein	Hypothetical	23.963	24.112	38.613	224.695	130.706	410.954
AMK58_19390	no KO assigned | (GenBank) disulfide bond formation protein DsbA	Cell Cycle/Shape/Homeostatsis	15.202	18.381	26.844	454.557	117.581	240.818
AMK58_19395	K03529 chromosome segregation protein | (GenBank) chromosome partitioning protein ParA	Cell Cycle/Shape/Homeostatsis	9.373	10.447	11.538	36.354	26.417	41.023
AMK58_19400	K19746 D-arginine dehydrogenase [EC:1.4.99.6] | (GenBank) FAD-dependent oxidoreductase	Metabolism: Amino Acid	229.012	219.769	203.357	73.350	44.065	143.553
AMK58_19405	no KO assigned | (GenBank) hypothetical protein	Hypothetical	186.465	235.014	224.652	1049.418	162.795	701.093
AMK58_19410	K07275 outer membrane protein | (GenBank) hypothetical protein	Membrane	117.264	129.355	126.647	250.124	100.269	122.549
AMK58_19415	no KO assigned | (GenBank) inosine-5-monophosphate dehydrogenase	dehydrogenase	79.047	91.459	86.273	129.749	118.573	253.962
AMK58_19420	no KO assigned | (GenBank) two-component system response regulator	Signal Transduction	2363.305	2637.724	2353.602	107.118	143.689	245.949
AMK58_19425	no KO assigned | (GenBank) acyl-CoA dehydrogenase	dehydrogenase	162.785	234.656	189.230	60.582	62.917	62.874

AMK58_19430	K07516 3-hydroxyacyl-CoA dehydrogenase [EC:1.1.1.35] | (GenBank) 3-hydroxyacyl-CoA dehydrogenas	Metabolism: Carbon	554.226	634.718	564.744	18.831	151.487	68.256
AMK58_19435	no KO assigned | (GenBank) acetyl-CoA acetyltransferase	Acetyltransferase	687.235	522.009	652.637	204.126	182.159	412.716
AMK58_19440	no KO assigned | (GenBank) transcriptional regulator	Transcription	2040.334	1832.203	2060.534	857.983	805.358	888.224
AMK58_19445	no KO assigned | (GenBank) hypothetical protein	Hypothetical	2235.583	2039.626	2303.637	988.182	1633.118	679.753
AMK58_19450	K06894 uncharacterized protein | (GenBank) alpha-2-macroglobulin	No COG	25.554	12.933	11.711	20.265	56.719	14.789
AMK58_19455	no KO assigned | (GenBank) histidine kinase	Signal Transduction	18.730	22.646	17.455	12.047	26.483	37.768
AMK58_19460	no KO assigned | (GenBank) aminotransferase	transferase	301.487	276.309	312.792	879.457	185.504	379.087
AMK58_19465	no KO assigned | (GenBank) pseudogene	Pseudogene	139.012	149.025	153.492	502.326	154.995	769.255
AMK58_19470	K02409 flagellar M-ring protein FliF | (GenBank) flagellar M-ring protein FliF	Motility	53.229	35.625	42.383	67.797	53.702	155.762
AMK58_19475	K02411 flagellar assembly protein FliH | (GenBank) flagellar assembly protein FliH	Motility	107.256	85.377	128.856	193.950	273.422	172.263
AMK58_19480	K02417 flagellar motor switch protein FliN/FliY | (GenBank) flagellar motor switch protein FliN	Motility	988.411	1095.993	987.581	570.108	91.141	1086.975
AMK58_19485	K02556 chemotaxis protein MotA | (GenBank) flagellar motor protein MotA	Chemotaxis	71.360	111.799	88.165	251.416	12.244	114.860
AMK58_19490	no KO assigned | (GenBank) AAA family ATPase	Enzyme	41.381	38.071	41.515	18.126	6.190	30.201
AMK58_19495	K02400 flagellar biosynthesis protein FlhA | (GenBank) flhA; flagellar biosynthesis protein FlhA	Motility	25.917	23.410	28.101	41.315	10.067	26.316
AMK58_19500	K02404 flagellar biosynthesis protein FlhF | (GenBank) GTPase	Motility	179.446	197.526	197.610	189.217	38.701	216.505
AMK58_19505	K04562 flagellar biosynthesis protein FlhG | (GenBank) cobyrinic acid a,c-diamide synthase	Motility	192.501	224.741	197.657	217.268	96.207	456.705
AMK58_19510	no KO assigned | (GenBank) hypothetical protein	Hypothetical	21.606	30.618	25.803	91.976	25.333	87.645
AMK58_19515	no KO assigned | (GenBank) hypothetical protein	Hypothetical	158.431	135.505	130.024	348.945	94.103	276.190
AMK58_19520	no KO assigned | (GenBank) hypothetical protein	Hypothetical	99.166	175.672	166.648	1922.289	320.791	903.504
AMK58_19525	no KO assigned | (GenBank) hypothetical protein	Hypothetical	5.272	8.689	4.753	46.026	14.304	56.641
AMK58_19530	no KO assigned | (GenBank) hypothetical protein	Hypothetical	51.692	59.454	39.424	69.614	21.439	152.769
AMK58_19535	K02412 flagellum-specific ATP synthase [EC:3.6.3.14] | (GenBank) fliI; flagellar protein export ATPa	Motility	82.424	73.034	67.430	102.465	52.942	188.950
AMK58_19540	K13584 two-component system, cell cycle response regulator CtrA | (GenBank) two-component system res	Cell Cycle/Shape/Homeostatsis	32.254	30.634	29.881	32.349	12.828	67.331
AMK58_19545	no KO assigned | (GenBank) hypothetical protein	Hypothetical	352.639	458.520	519.608	1383.454	334.655	1217.175
AMK58_19550	K03566 LysR family transcriptional regulator, glycine cleavage system transcriptional activator | (G	Transcription	1931.049	2072.661	1871.763	88.074	1258.937	214.626
AMK58_19555	K00575 chemotaxis protein methyltransferase CheR [EC:2.1.1.80] | (GenBank) chemotaxis protein CheR	Chemotaxis	68.266	95.763	71.303	67.482	111.963	80.818
AMK58_19560	K03412 two-component system, chemotaxis family, response regulator CheB [EC:3.1.1.61] | (GenBank) tw	Chemotaxis	49.046	54.531	60.958	114.311	220.642	179.060
AMK58_19565	K03413 two-component system, chemotaxis family, response regulator CheY | (GenBank) two-component	Chemotaxis	484.961	658.135	566.159	312.850	460.337	545.397
AMK58_19570	K03408 purine-binding chemotaxis protein CheW | (GenBank) chemotaxis protein CheW	Chemotaxis	367.333	440.366	449.446	247.973	240.404	322.915
AMK58_19575	K03407 two-component system, chemotaxis family, sensor kinase CheA [EC:2.7.13.3] | (GenBank) ATPa	Chemotaxis	78.353	74.271	72.168	8.854	17.467	23.258
AMK58_19580	K13588 histidine phosphotransferase ChpT | (GenBank) hypothetical protein	Signal Transduction	421.423	387.031	431.490	112.996	77.489	103.596
AMK58_19585	no KO assigned | (GenBank) hypothetical protein	Hypothetical	7030.939	8008.297	7954.530	2080.942	1482.152	2884.656
AMK58_19590	K08738 cytochrome c | (GenBank) hypothetical protein	Energy	427.360	469.153	484.273	311.380	265.505	342.321
AMK58_19595	K01434 penicillin amidase [EC:3.5.1.11] | (GenBank) acyl-homoserine-lactone acylase	Defense	7.001	7.171	8.179	6.029	8.601	10.480
AMK58_19600	K01754 threonine dehydratase [EC:4.3.1.19] | (GenBank) threonine dehydratase	Metabolism	10.381	15.070	13.744	93.439	17.012	58.024
AMK58_19605	no KO assigned | (GenBank) flavin reductase	No COG	102.730	121.290	133.491	943.955	366.214	593.101
AMK58_19610	K02557 chemotaxis protein MotB | (GenBank) chemotaxis protein MotB	Chemotaxis	27.248	34.056	40.541	277.029	67.790	107.830
AMK58_19615	no KO assigned | (GenBank) hypothetical protein	Hypothetical	45.201	38.579	46.508	32.870	33.660	75.296
AMK58_19620	no KO assigned | (GenBank) hypothetical protein	Hypothetical	87.154	82.099	98.051	246.098	48.008	200.088
AMK58_19625	K00685 arginine-tRNA-protein transferase [EC:2.3.2.8] | (GenBank) arginyl-tRNA-protein transferase	tRNA synthesis/modification	885.267	893.212	824.475	168.314	167.132	495.290
AMK58_19630	no KO assigned | (GenBank) sugar transporter	Transport: Sugar	710.530	647.001	646.585	211.442	144.662	454.900
AMK58_19635	no KO assigned | (GenBank) C4-dicarboxylate ABC transporter	Membrane Transport	88.724	100.466	84.131	68.865	42.394	121.848
AMK58_19640	no KO assigned | (GenBank) ABC transporter substrate-binding protein	Membrane Transport	821.169	664.723	645.316	26.883	97.546	92.229
AMK58_19645	K02621 topoisomerase IV subunit A [EC:5.99.1.-] | (GenBank) DNA topoisomerase IV subunit A	DNA Repair and Replication	654.316	470.770	555.622	78.772	39.611	156.663
AMK58_19650	K03584 DNA repair protein RecO (recombination protein O) | (GenBank) DNA repair protein RecO	DNA Repair and Replication	258.393	241.119	251.096	126.070	45.553	444.420
AMK58_19655	no KO assigned | (GenBank) diguanylate cyclase	cyclic nucleotide	173.170	159.788	176.314	105.998	119.656	199.828
AMK58_19660	no KO assigned | (GenBank) hypothetical protein	Hypothetical	39.412	41.442	46.467	348.446	214.418	225.416
AMK58_19665	no KO assigned | (GenBank) hypothetical protein	Hypothetical	459.481	598.639	532.590	747.546	531.337	1083.983
AMK58_19670	K03446 MFS transporter, DHA2 family, multidrug resistance protein | (GenBank) MFS transporter	Transport: MFS	12.211	14.211	12.062	15.613	54.188	51.687
AMK58_19675	K03543 membrane fusion protein, multidrug efflux system | (GenBank) hemolysin D	Transport	38.165	30.140	37.747	45.982	97.016	86.338
AMK58_19680	no KO assigned | (GenBank) transcriptional regulator	Transcription	110.547	99.010	122.719	337.169	726.926	487.587
AMK58_19685	no KO assigned | (GenBank) hypothetical protein	Hypothetical	148.100	170.075	181.586	338.853	1557.376	500.369
AMK58_19690	K09812 cell division transport system ATP-binding protein | (GenBank) cell division ATP-binding prot	Cell Cycle/Shape/Homeostatsis	228.815	212.439	207.248	134.798	898.095	213.494
AMK58_19695	K09811 cell division transport system permease protein | (GenBank) hypothetical protein	Cell Cycle/Shape/Homeostatsis	56.314	76.101	63.233	153.565	340.610	89.766
AMK58_19700	no KO assigned | (GenBank) hypothetical protein	Hypothetical	108.251	127.320	111.353	225.342	408.704	329.289
AMK58_19705	no KO assigned | (GenBank) hypothetical protein	Hypothetical	17.628	17.091	23.230	69.597	80.039	24.662
AMK58_19710	K00655 1-acyl-sn-glycerol-3-phosphate acyltransferase [EC:2.3.1.51] | (GenBank) acyltransferase	Lipid metabolism	29.380	30.486	26.464	52.405	129.662	84.453
AMK58_19715	no KO assigned | (GenBank) hypothetical protein	Hypothetical	109.263	111.003	84.818	178.668	193.214	599.456
AMK58_19720	K00525 ribonucleoside-diphosphate reductase alpha chain [EC:1.17.4.1] | (GenBank) ribonucleotide red	Metabolism: Nucleotide	37.201	26.092	33.420	38.132	40.337	46.255
AMK58_19725	no KO assigned | (GenBank) hypothetical protein	Hypothetical	20.761	19.707	26.197	212.869	97.272	110.470
AMK58_19730	K01951 GMP synthase (glutamine-hydrolysing) [EC:6.3.5.2] | (GenBank) glutamine amidotransferase	Metabolism: Nucleotide	245.082	250.392	265.823	387.213	248.341	653.787
AMK58_19735	K00375 GntR family transcriptional regulator / MocR family aminotransferase | (GenBank) GntR family	Transcription	22.899	24.743	27.023	103.120	42.352	101.276
AMK58_19740	K05782 benzoate membrane transport protein | (GenBank) benzoate transporter	Membrane Transport	5.375	7.006	5.761	147.787	31.964	86.082
AMK58_19745	no KO assigned | (GenBank) LysR family transcriptional regulator	Transcription	2.562	3.827	8.771	305.836	58.902	249.251
AMK58_19750	K00355 NAD(P)H dehydrogenase (quinone) [EC:1.6.5.2] | (GenBank) NAD(P)H dehydrogenase	Energy	3.711	6.467	5.734	261.196	87.948	321.754
AMK58_19755	K09936 bacterial/archaeal transporter family-2 protein | (GenBank) hypothetical protein	Transport	11.493	11.178	18.805	276.038	85.622	200.995
AMK58_19760	no KO assigned | (GenBank) hypothetical protein	Hypothetical	2.214	2.035	5.991	280.575	28.903	237.803

AMK58_19765	no KO assigned | (GenBank) glycosyl transferase family 1	Glycosyl Transferase	3.737	3.126	5.472	58.968	11.257	51.006
AMK58_19770	no KO assigned | (GenBank) hypothetical protein	Hypothetical	109.706	108.673	92.419	766.005	194.875	627.761
AMK58_19775	no KO assigned | (GenBank) ABC transporter substrate-binding protein	Membrane Transport	82.051	75.059	81.080	209.248	98.852	286.911
AMK58_19780	K04654 hydrogenase expression/formation protein HypD | (GenBank) hydrogenase formation protein Hup	enzyme	136.899	138.960	166.317	27.600	16.236	51.112
AMK58_19785	no KO assigned | (GenBank) hypothetical protein	Hypothetical	9721.942	9314.488	8533.896	181.658	267.456	421.410
AMK58_19790	no KO assigned | (GenBank) peptidase M1	Peptidase	4.547	8.186	6.218	105.621	55.095	118.738
AMK58_19795	K07232 cation transport protein ChaC | (GenBank) gamma-glutamyl cyclotransferase	Transport: Ion	8.337	13.584	14.412	311.681	160.160	206.794
AMK58_19800	no KO assigned | (GenBank) hypothetical protein	Hypothetical	80.239	84.891	103.539	310.936	264.805	183.352
AMK58_19805	no KO assigned | (GenBank) hypothetical protein	Hypothetical	112.427	128.022	121.606	146.868	218.397	90.529
AMK58_19810	no KO assigned | (GenBank) hypothetical protein	Hypothetical	81.188	90.068	84.062	272.203	142.977	184.058
AMK58_19815	K08989 putative membrane protein | (GenBank) hypothetical protein	Membrane	360.666	334.509	352.113	429.069	1086.509	664.658
AMK58_19820	no KO assigned | (GenBank) hypothetical protein	Hypothetical	68.293	81.351	74.744	45.224	106.113	60.723
AMK58_19825	K01682 aconitate hydratase 2 / 2-methylisocitrate dehydratase [EC:4.2.1.3 4.2.1.99] | (GenBank) acon	Metabolism: Carbon	9.926	10.909	9.215	31.358	19.840	21.186
AMK58_19830	no KO assigned | (GenBank) AraC family transcriptional regulator	Transcription	65.299	76.761	79.620	389.570	68.471	132.873
AMK58_19835	K08223 MFS transporter, FSR family, fosmidomycin resistance protein | (GenBank) Fosmidomycin resista	Transport: MFS	1031.746	845.114	899.379	299.358	472.828	418.079
AMK58_19840	no KO assigned | (GenBank) hypothetical protein	Hypothetical	15.394	39.772	30.065	628.122	79.505	560.577
AMK58_19845	K02553 regulator of ribonuclease activity A | (GenBank) ribonuclease activity regulator protein RraA	post translation modificaton	25.285	30.503	37.448	438.683	86.903	469.143
AMK58_19850	no KO assigned | (GenBank) hypothetical protein	Hypothetical	40.127	41.453	43.431	44.303	64.082	110.232
AMK58_19855	K07220 uncharacterized protein | (GenBank) hypothetical protein	Hypothetical	59.797	95.120	74.458	138.131	53.272	205.656
AMK58_19860	K03306 inorganic phosphate transporter, PiT family | (GenBank) inorganic phosphate transporter	Transport: Inorganic	18.858	21.528	23.412	161.060	47.930	103.694
AMK58_19865	K14225 tRNA Gly | (GenBank) tRNA-Gly	tRNA synthesis/modification	807.165	511.171	646.201	1816.482	361.101	2184.019
AMK58_19870	no KO assigned | (GenBank) hypothetical protein	Hypothetical	25.552	15.219	20.057	61.846	9.842	43.587
AMK58_19875	no KO assigned | (GenBank) hypothetical protein	Hypothetical	7.053	16.387	10.059	49.338	11.451	88.653
AMK58_19880	no KO assigned | (GenBank) hypothetical protein	Hypothetical	1328.097	1654.444	1766.338	3351.010	1754.555	2609.838
AMK58_19885	K09136 ribosomal protein S12 methylthiotransferase accessory factor | (GenBank) hypothetical protein	Ribosome	144.369	158.073	134.807	52.007	90.190	73.691
AMK58_19890	no KO assigned | (GenBank) tfuA protein	No COG	336.424	409.713	361.396	100.099	49.086	101.744
AMK58_19895	no KO assigned | (GenBank) Crp/Fnr family transcriptional regulator	Transcription	273.483	327.596	272.335	362.922	86.709	322.282
AMK58_19900	no KO assigned | (GenBank) hypothetical protein	Hypothetical	113.253	164.484	128.802	385.977	72.442	295.614
AMK58_19905	no KO assigned | (GenBank) hypothetical protein	Hypothetical	33.484	31.512	26.653	24.054	14.122	95.949
AMK58_19910	no KO assigned | (GenBank) regulator	Signal transduction	598.009	688.156	776.983	106.940	191.723	312.771
AMK58_19915	K01784 UDP-glucose 4-epimerase [EC:5.1.3.2] | (GenBank) epimerase	Metabolism: Carbohydrates	95.404	73.251	95.082	305.039	246.489	501.542
AMK58_19920	K03324 phosphate:Na+ symporter | (GenBank) sodium:phosphate symporter	Transport: Ion	61.114	68.460	71.264	19.501	118.190	54.932
AMK58_19925	no KO assigned | (GenBank) multidrug DMT transporter permease	Defense	33.497	35.364	55.385	152.635	235.290	153.560
AMK58_19930	no KO assigned | (GenBank) LysR family transcriptional regulator	Transcription	213.816	272.497	375.451	324.927	410.144	198.779
AMK58_19935	no KO assigned | (GenBank) hypothetical protein	Hypothetical	2.465	4.601	6.298	222.644	132.803	233.967
AMK58_19940	no KO assigned | (GenBank) hypothetical protein	Hypothetical	4.309	3.003	6.623	102.160	55.939	62.261
AMK58_19945	no KO assigned | (GenBank) hypothetical protein	Hypothetical	28.761	27.715	44.989	763.081	443.737	709.751
AMK58_19950	K03745 SlyX protein | (GenBank) SlyX protein	No COG	330.153	304.747	378.933	1306.497	1308.007	1062.924
AMK58_19955	no KO assigned | (GenBank) hypothetical protein	Hypothetical	6.246	17.223	6.395	189.217	169.576	111.379
AMK58_19960	K03711 Fur family transcriptional regulator, ferric uptake regulator | (GenBank) Fur family transcri	Transcription	39.512	40.467	38.910	65.782	228.408	108.606
AMK58_19965	no KO assigned | (GenBank) hypothetical protein	Hypothetical	19.262	25.573	26.842	27.167	59.913	24.949
AMK58_19970	no KO assigned | (GenBank) hypothetical protein	Hypothetical	150.352	191.923	177.366	66.318	317.322	91.249
AMK58_19975	no KO assigned | (GenBank) hypothetical protein	Hypothetical	4.713	7.920	10.784	409.608	91.722	278.079
AMK58_19980	K03406 methyl-accepting chemotaxis protein | (GenBank) chemotaxis protein	Chemotaxis	3.766	5.519	7.499	197.593	71.321	115.630
AMK58_19985	no KO assigned | (GenBank) hypothetical protein	Hypothetical	8.617	9.314	10.540	64.271	40.740	51.758
AMK58_19990	no KO assigned | (GenBank) hypothetical protein	Hypothetical	1416.626	1288.000	1308.081	710.675	1457.403	645.432
AMK58_19995	no KO assigned | (GenBank) hypothetical protein	Hypothetical	22.005	24.484	28.716	340.722	117.567	398.107
AMK58_20000	K14234 tRNA Thr | (GenBank) tRNA-Thr	tRNA synthesis/modification	831.380	1138.079	1026.861	1394.799	2386.167	1588.242
AMK58_20005	K14218 tRNA Ala | (GenBank) tRNA-Ala	tRNA synthesis/modification	211.881	333.124	375.651	2641.923	1925.002	1978.014
AMK58_20010	K14221 tRNA Asp | (GenBank) tRNA-Asp	tRNA synthesis/modification	9.434	9.394	8.970	141.123	40.651	202.669
AMK58_20015	K14237 tRNA Val | (GenBank) tRNA-Val	tRNA synthesis/modification	1444.826	1745.698	839.601	60.549	381.061	366.748
AMK58_20020	K14223 tRNA Gln | (GenBank) tRNA-Gln	tRNA synthesis/modification	948.965	928.632	724.938	333.139	191.266	723.684
AMK58_20025	no KO assigned | (GenBank) hypothetical protein	Hypothetical	84.965	82.488	90.378	157.206	37.008	32.992
AMK58_20030	no KO assigned | (GenBank) hypothetical protein	Hypothetical	59.790	53.582	52.822	15.351	75.887	49.436
AMK58_20035	no KO assigned | (GenBank) hypothetical protein	Hypothetical	16.993	25.381	25.919	138.474	100.677	248.503
AMK58_20040	K01420 CRP/FNR family transcriptional regulator, anaerobic regulatory protein | (GenBank) hypothetic	Transcription	28.488	48.224	36.718	182.327	78.098	115.058
AMK58_20045	no KO assigned | (GenBank) hypothetical protein	Hypothetical	125.908	163.152	133.501	130.461	786.407	71.709
AMK58_20050	no KO assigned | (GenBank) hypothetical protein	Hypothetical	88.519	90.018	104.886	418.079	587.015	268.948
AMK58_20055	K17675 ATP-dependent RNA helicase SUPV3L1/SUV3 [EC:3.6.4.13] | (GenBank) RNA helicase	Genetic Information Processing	5.385	5.199	6.051	31.111	17.913	35.406
AMK58_20060	K00311 electron-transferring-flavoprotein dehydrogenase [EC:1.5.5.1] | (GenBank) electron transfer f	Energy	1.027	0.877	1.605	58.584	25.899	29.530
AMK58_20065	K02334 DNA polymerase bacteriophage-type [EC:2.7.7.7] | (GenBank) uracil-DNA glycosylase	DNA Repair and Replication	21.785	25.864	28.547	537.627	141.430	143.477
AMK58_20070	no KO assigned | (GenBank) murein transglycosylase	transglycosylase	80.655	58.720	74.293	104.750	55.860	130.953
AMK58_20075	K03638 molybdenum cofactor biosynthesis protein B | (GenBank) molybdenum cofactor biosynthesis prot	Biosynthesis: Co-Factors and Vitamins	81.167	101.027	111.046	176.985	63.865	240.010
AMK58_20080	no KO assigned | (GenBank) aminoglycoside phosphotransferase	transferase	33.791	49.936	55.629	251.756	97.574	178.148
AMK58_20085	no KO assigned | (GenBank) hypothetical protein	Hypothetical	11.768	9.512	13.559	30.045	54.780	28.437
AMK58_20090	K00919 4-diphosphocytidyl-2-C-methyl-D-erythritol kinase [EC:2.7.1.148] | (GenBank) 4-diphosphocytid	Metabolism: terpenoids and polyketide	8.589	11.248	11.076	111.434	78.164	66.554
AMK58_20095	no KO assigned | (GenBank) hypothetical protein	Hypothetical	395.813	424.214	487.973	571.843	600.896	499.110

AMK58_20100	no KO assigned | (GenBank) pseudogene	Pseudogene	102.497	107.164	113.596	335.956	126.012	211.768
AMK58_20105	K17686 Cu+-exporting ATPase [EC:3.6.3.54] | (GenBank) copper-transporting ATPase	Hydrolase	6.617	8.148	10.470	54.536	24.868	36.580
AMK58_20110	no KO assigned | (GenBank) hypothetical protein	Hypothetical	26.321	34.945	37.817	336.908	65.746	398.096
AMK58_20115	no KO assigned | (GenBank) hypothetical protein	Hypothetical	21.765	29.279	34.399	576.146	110.333	336.809
AMK58_20120	no KO assigned | (GenBank) hypothetical protein	Hypothetical	5.805	0.000	8.673	125.898	13.361	160.202
AMK58_20125	no KO assigned | (GenBank) hypothetical protein	Hypothetical	2.590	1.289	7.798	131.541	69.866	287.178
AMK58_20130	K06911 uncharacterized protein | (GenBank) quercetin 2,3-dioxygenase	No COG	1.559	0.000	4.776	169.843	12.850	146.802
AMK58_20135	no KO assigned | (GenBank) hypothetical protein	Hypothetical	4.973	1.143	6.729	302.593	37.410	172.927
AMK58_20140	K06975 uncharacterized protein | (GenBank) acetyltransferase	Acetyltransferase	32.113	51.853	37.548	344.718	174.141	575.432
AMK58_20145	K01607 4-carboxymuconolactone decarboxylase [EC:4.1.1.44] | (GenBank) 4-carboxymuconolactone deca	Metabolism	23.142	25.292	37.568	303.957	77.722	217.735
AMK58_20150	no KO assigned | (GenBank) MerR family transcriptional regulator	Transcription	12.575	12.231	14.459	166.103	187.046	88.134
AMK58_20155	K01996 branched-chain amino acid transport system ATP-binding protein | (GenBank) livF; ABC transpor	Transport: Amino Acid	9.274	9.234	9.797	51.761	97.485	78.031
AMK58_20160	K07090 uncharacterized protein | (GenBank) hypothetical protein	Hypothetical	9.276	8.750	10.831	74.553	62.742	115.439
AMK58_20165	K01895 acetyl-CoA synthetase [EC:6.2.1.1] | (GenBank) AMP-dependent synthetase	Metabolism	38.112	36.235	39.298	84.353	32.151	97.967
AMK58_20170	no KO assigned | (GenBank) hypothetical protein	Hypothetical	266.637	346.777	304.828	400.605	94.297	254.394
AMK58_20175	no KO assigned | (GenBank) hypothetical protein	Hypothetical	239.071	289.944	260.468	149.514	79.278	155.091
AMK58_20180	no KO assigned | (GenBank) hypothetical protein	Hypothetical	97.450	82.333	134.305	355.358	408.833	305.319
AMK58_20185	no KO assigned | (GenBank) hypothetical protein	Hypothetical	8.607	12.531	9.903	224.968	54.470	79.608
AMK58_20190	no KO assigned | (GenBank) glycosyl transferase	Glycosyl Transferase	35.719	48.039	42.637	61.933	10.602	66.674
AMK58_20195	no KO assigned | (GenBank) hypothetical protein	Hypothetical	136.080	148.381	183.502	946.777	175.642	496.577
AMK58_20200	no KO assigned | (GenBank) hypothetical protein	Hypothetical	282.508	304.019	369.827	463.836	577.903	602.568
AMK58_20205	no KO assigned | (GenBank) fusaric acid resistance protein	Defense	2.030	2.079	2.812	17.011	5.671	8.926
AMK58_20210	no KO assigned | (GenBank) secretion protein HlyD	Secretion System	4.677	3.194	5.718	55.315	30.944	50.806
AMK58_20215	K06075 MarR family transcriptional regulator, transcriptional regulator for hemolysin | (GenBank) Ma	Transcription	33.505	21.231	44.406	798.009	219.119	410.479
AMK58_20220	no KO assigned | (GenBank) LysR family transcriptional regulator	Transcription	8.072	12.056	13.047	254.271	79.085	210.319
AMK58_20225	no KO assigned | (GenBank) MFS sugar transporter	Transport: Sugar	4.056	9.693	9.834	105.951	27.925	90.455
AMK58_20230	no KO assigned | (GenBank) 2,5-didehydrogluconate reductase	enzyme	4.696	6.138	9.628	248.882	45.364	152.415
AMK58_20235	no KO assigned | (GenBank) permease	No COG	0.883	2.436	2.455	139.888	26.883	99.984
AMK58_20240	K07290 AsmA family protein | (GenBank) membrane biogenesis protein	Membrane	2.878	2.508	5.246	78.643	38.624	97.979
AMK58_20245	no KO assigned | (GenBank) hypothetical protein	Hypothetical	60.537	71.418	77.267	287.498	130.546	249.251
AMK58_20250	no KO assigned | (GenBank) hypothetical protein	Hypothetical	9.420	8.828	19.318	255.341	104.224	293.134
AMK58_20255	no KO assigned | (GenBank) hypothetical protein	Hypothetical	30.825	34.174	39.582	495.071	45.364	480.019
AMK58_20260	no KO assigned | (GenBank) hypothetical protein	Hypothetical	11.624	16.387	16.392	217.123	29.949	117.841
AMK58_20265	no KO assigned | (GenBank) CoA-transferase	No COG	1.812	4.810	3.349	69.566	6.788	35.277
AMK58_20270	K00252 glutaryl-CoA dehydrogenase [EC:1.3.8.6] | (GenBank) acyl-CoA dehydrogenase	Metabolism	13.386	15.032	17.416	141.828	19.911	102.377
AMK58_20275	no KO assigned | (GenBank) LysR family transcriptional regulator	Transcription	107.358	167.049	133.177	302.111	56.631	198.612
AMK58_20280	K02052 putative spermidine/putrescine transport system ATP-binding protein | (GenBank) spermidine/pu	Quorum sensing	370.423	609.965	384.248	148.305	65.227	224.539
AMK58_20285	K02055 putative spermidine/putrescine transport system substrate-binding protein | (GenBank) spermid	Quorum sensing	4.626	4.145	8.576	155.835	18.718	150.336
AMK58_20290	K02054 putative spermidine/putrescine transport system permease protein | (GenBank) polyamine ABC tr	Quorum sensing	120.404	150.055	119.221	203.792	51.937	187.561
AMK58_20295	K02053 putative spermidine/putrescine transport system permease protein | (GenBank) polyamine ABC tr	Quorum sensing	994.387	934.878	924.830	134.081	113.002	323.569
AMK58_20300	no KO assigned | (GenBank) XRE family transcriptional regulator	Transcription	204.689	175.584	185.174	318.550	57.229	410.696
AMK58_20305	K00135 succinate-semialdehyde dehydrogenase / glutarate-semialdehyde dehydrogenase [EC:1.2.1.16 1.2.	Metabolism	56.801	49.613	56.059	53.283	11.761	117.814
AMK58_20310	K07250 4-aminobutyrate aminotransferase / (S)-3-amino-2-methylpropionate transaminase [EC:2.6.1.19 2	Metabolism	35.937	25.854	31.664	36.923	48.220	103.546
AMK58_20315	no KO assigned | (GenBank) hypothetical protein	Hypothetical	131.791	103.531	103.412	441.353	133.018	423.918
AMK58_20320	no KO assigned | (GenBank) RNA signal recognition particle 4.5S RNA	No COG	472.390	373.536	523.068	582.717	199.306	753.372
AMK58_20325	K04750 PhnB protein | (GenBank) bleomycin resistance protein	Defense	31.452	34.921	31.228	203.571	37.230	200.279
AMK58_20330	no KO assigned | (GenBank) polyketide cyclase	No COG	186.690	197.562	184.933	97.312	64.096	145.347
AMK58_20335	K16137 TetR/AcrR family transcriptional regulator, transcriptional repressor for nem operon | (GenBa	Transcription	162.363	164.449	142.351	68.013	57.071	135.034
AMK58_20340	no KO assigned | (GenBank) hypothetical protein	Hypothetical	31.917	35.459	37.119	96.817	41.065	68.854
AMK58_20345	K06987 uncharacterized protein | (GenBank) deacylase	No COG	20.722	20.741	22.591	54.353	49.998	81.385
AMK58_20350	K00819 ornithine--oxo-acid transaminase [EC:2.6.1.13] | (GenBank) rocD; ornithine--oxo-acid aminotra	Metabolism: Amino Acid	59.046	65.922	50.471	72.038	19.442	272.378
AMK58_20355	K01476 arginase [EC:3.5.3.1] | (GenBank) arginase	Biosynthesis: Amino Acid	244.155	186.455	182.331	255.703	54.099	141.209
AMK58_20360	no KO assigned | (GenBank) AsnC family transcriptional regulator	Transcription	2902.690	1812.604	1512.647	669.537	161.789	425.002
AMK58_20365	no KO assigned | (GenBank) chromosome partitioning protein	Cell Cycle/Shape/Homeostatsis	446.728	309.058	286.737	159.199	40.226	97.590
AMK58_20370	no KO assigned | (GenBank) galactosyltransferase	enzyme	6.624	12.884	8.202	42.094	9.523	62.420
AMK58_20375	no KO assigned | (GenBank) hypothetical protein	Hypothetical	29.343	27.956	31.190	81.746	17.368	48.858
AMK58_20380	no KO assigned | (GenBank) hypothetical protein	Hypothetical	40.693	42.021	30.889	286.461	29.553	197.831
AMK58_20385	no KO assigned | (GenBank) alpha-1,3-fucosyltransferase	transferase	18.446	19.230	23.071	310.276	30.336	125.586
AMK58_20390	K14231 tRNA Phe | (GenBank) tRNA-Phe	tRNA synthesis/modification	353.666	295.053	301.430	1348.704	284.721	1793.360
AMK58_20395	K09862 uncharacterized protein | (GenBank) hypothetical protein	Hypothetical	283.810	268.342	230.857	917.309	297.064	617.483
AMK58_20400	K08300 ribonuclease E [EC:3.1.26.12] | (GenBank) ribonuclease E	Genetic Information Processing	1.755	2.330	2.410	18.412	9.314	34.621
AMK58_20405	K06287 septum formation protein | (GenBank) septum formation inhibitor Maf	Cell Cycle/Shape/Homeostatsis	7.392	2.387	3.229	49.245	33.462	62.804
AMK58_20410	K02518 translation initiation factor IF-1 | (GenBank) translation initiation factor IF-1	Translation	90.115	59.454	97.772	1041.248	513.301	702.839
AMK58_20415	no KO assigned | (GenBank) hypothetical protein	Hypothetical	92.247	87.439	93.508	85.994	113.536	44.723
AMK58_20420	no KO assigned | (GenBank) histidine kinase	Signal Transduction	103.838	100.466	85.840	27.594	65.802	34.061
AMK58_20425	no KO assigned | (GenBank) ArsC family transcriptional regulator	Transcription	105.306	140.519	164.419	534.533	55.879	305.830
AMK58_20430	no KO assigned | (GenBank) hypothetical protein	Hypothetical	166.196	200.682	181.140	1000.314	158.635	901.179

AMK58_20435	K00013 histidinol dehydrogenase [EC:1.1.1.23] | (GenBank) histidinol dehydrogenase	Metabolism: Amino Acid	36.933	44.242	33.532	38.067	30.382	87.893
AMK58_20440	K00765 ATP phosphoribosyltransferase [EC:2.4.2.17] | (GenBank) hisG; ATP phosphoribosyltransferase	Biosynthesis: Amino Acid	115.826	104.881	100.665	42.183	78.523	102.536
AMK58_20445	no KO assigned | (GenBank) hypothetical protein	Hypothetical	789.094	899.696	892.888	1285.384	577.550	692.609
AMK58_20450	K02073 D-methionine transport system substrate-binding protein | (GenBank) methionine ABC transporte	Membrane Transport	244.323	249.465	218.283	115.401	104.862	234.039
AMK58_20455	K00666 fatty-acyl-CoA synthase [EC:6.2.1.-] | (GenBank) acyl-CoA synthetase	enzyme	0.217	1.732	0.689	88.809	3.991	41.253
AMK58_20460	K02071 D-methionine transport system ATP-binding protein | (GenBank) phosphate ABC transporter ATF	Transport	9.891	10.188	11.458	112.083	43.575	187.854
AMK58_20465	K02072 D-methionine transport system permease protein | (GenBank) DL-methionine transporter permease	Transport: Amino Acid	14.544	15.206	17.631	379.408	100.333	390.415
AMK58_20470	K02073 D-methionine transport system substrate-binding protein | (GenBank) metal ABC transporter sub	Transport: Amino Acid	97.797	80.373	90.112	178.384	206.433	187.677
AMK58_20475	no KO assigned | (GenBank) hypothetical protein	Hypothetical	26.500	20.699	24.751	65.978	47.364	53.921
AMK58_20480	no KO assigned | (GenBank) histidine kinase	Signal Transduction	163.681	131.641	140.291	57.601	60.718	81.257
AMK58_20485	K01214 isoamylase [EC:3.2.1.68] | (GenBank) glycogen debranching protein	Metabolism: Carbohydrate	66.257	80.689	73.825	109.188	29.052	108.979
AMK58_20490	no KO assigned | (GenBank) hypothetical protein	Hypothetical	6465.883	8325.896	7418.195	2187.052	1875.749	1727.138
AMK58_20495	no KO assigned | (GenBank) hypothetical protein	Hypothetical	92.045	126.904	150.126	979.754	870.280	1579.326
AMK58_20500	no KO assigned | (GenBank) hypothetical protein	Hypothetical	48.919	58.453	38.373	102.663	20.620	43.093
AMK58_20505	K01834 2,3-bisphosphoglycerate-dependent phosphoglycerate mutase [EC:5.4.2.11] | (GenBank) gpmA; pl	Metabolism	107.569	146.190	161.046	29.585	52.151	73.078
AMK58_20510	K17675 ATP-dependent RNA helicase SUPV3L1/SUV3 [EC:3.6.4.13] | (GenBank) helicase	Translation	27.401	37.754	48.219	13.231	9.968	30.136
AMK58_20515	no KO assigned | (GenBank) glycosyltransferase	Glycosyl Transferase	24.030	23.928	37.641	145.678	474.075	177.546
AMK58_20520	K16554 polysaccharide biosynthesis transport protein | (GenBank) exopolysaccharide biosynthesis prot	Transport: polysaccharide	22.175	27.722	30.524	239.665	167.023	142.288
AMK58_20525	K01181 endo-1,4-beta-xylanase [EC:3.2.1.8] | (GenBank) glycosyl hydrolase	Hydrolase	20.467	17.223	42.850	127.046	47.201	183.552
AMK58_20530	no KO assigned | (GenBank) methyltransferase	methyltransferase	34.350	25.751	74.759	411.501	186.890	386.222
AMK58_20535	K01179 endoglucanase [EC:3.2.1.4] | (GenBank) endoglucanase	Metabolism: Carbohydrate	1.558	1.454	4.073	95.146	17.620	99.585
AMK58_20540	no KO assigned | (GenBank) hypothetical protein	Hypothetical	49.182	45.881	80.008	66.647	97.157	133.784
AMK58_20545	no KO assigned | (GenBank) UDP-phosphate galactose phosphotransferase	No COG	37.009	45.022	51.748	256.065	53.082	380.444
AMK58_20550	no KO assigned | (GenBank) polymerase	DNA Repair and Replication	13.573	14.056	26.070	164.004	92.865	177.970
AMK58_20555	no KO assigned | (GenBank) glycosyl transferase family 2	Glycosyl Transferase	17.602	10.516	38.373	215.069	52.996	313.492
AMK58_20560	no KO assigned | (GenBank) glycosyl transferase family 1	Glycosyl Transferase	14.020	14.958	37.706	187.272	93.093	224.632
AMK58_20565	no KO assigned | (GenBank) glycosyl transferase family 1	Glycosyl Transferase	8.587	7.054	24.902	175.270	64.910	137.948
AMK58_20570	no KO assigned | (GenBank) polysaccharide deacetylase	Metabolism: Carbohydrates	15.665	15.863	43.675	328.995	132.363	245.221
AMK58_20575	no KO assigned | (GenBank) acyl carrier protein	No COG	63.595	36.533	105.429	751.953	351.460	725.406
AMK58_20580	no KO assigned | (GenBank) long-chain fatty acid--CoA ligase	No COG	5.433	10.201	15.497	90.970	29.312	117.595
AMK58_20585	K01953 asparagine synthase (glutamine-hydrolysing) [EC:6.3.5.4] | (GenBank) asparagine synthase	Metabolism: Amino Acid	25.486	25.079	47.667	10.122	32.220	34.476
AMK58_20590	K01916 NAD+ synthase [EC:6.3.1.5] | (GenBank) nadE; NAD(+) synthetase	Energy	33.031	33.010	73.777	353.012	105.715	295.710
AMK58_20595	no KO assigned | (GenBank) hypothetical protein	Hypothetical	12.031	16.336	30.782	434.986	71.919	334.882
AMK58_20600	K01980 23S ribosomal RNA | (GenBank) 23S ribosomal RNA	Ribosome	8.177	9.193	17.347	54.945	16.950	62.984
AMK58_20605	K01977 16S ribosomal RNA | (GenBank) 16S ribosomal RNA	Ribosome	8.839	10.174	20.741	143.827	29.807	111.743
AMK58_20610	no KO assigned | (GenBank) transcriptional regulator	Transcription	85.884	51.458	70.662	32.635	185.883	247.130
AMK58_20615	no KO assigned | (GenBank) hypothetical protein	Hypothetical	8.760	9.346	13.088	13.411	46.068	40.614
AMK58_20620	K03455 monovalent cation:H+ antiporter-2, CPA2 family | (GenBank) potassium transporter KefB	Transport: Ion	9.040	9.356	11.776	43.383	15.522	48.512
AMK58_20625	no KO assigned | (GenBank) hypothetical protein	Hypothetical	4.245	5.404	3.705	43.436	11.028	42.069
AMK58_20630	K02045 sulfate transport system ATP-binding protein [EC:3.6.3.25] | (GenBank) sulfate ABC transporte	Energy	5.117	6.254	7.077	248.031	58.830	215.480
AMK58_20635	K02047 sulfate transport system permease protein | (GenBank) cysW; sulfate/thiosulfate transporter p	Energy	4.922	4.201	8.557	371.841	57.851	302.385
AMK58_20640	K02046 sulfate transport system permease protein | (GenBank) sulfate transporter	Energy	35.155	41.167	31.955	164.823	49.948	125.570
AMK58_20645	K02048 sulfate transport system substrate-binding protein | (GenBank) ABC transporter permease	Energy	76.182	68.407	69.201	131.814	57.215	166.407
AMK58_20650	no KO assigned | (GenBank) hypothetical protein	Hypothetical	100.841	86.724	99.121	208.882	103.213	102.175
AMK58_20655	no KO assigned | (GenBank) Rrf2 family transcriptional regulator	Transcription	255.513	134.132	137.482	253.125	307.336	542.017
AMK58_20660	no KO assigned | (GenBank) hypothetical protein	Hypothetical	13.772	13.713	20.550	338.208	132.008	154.482
AMK58_20665	no KO assigned | (GenBank) osmotically inducible protein OsmC	Stress response	91.101	90.243	88.191	70.818	197.110	246.862
AMK58_20670	no KO assigned | (GenBank) ABC transporter substrate-binding protein	Membrane Transport	249.558	237.155	224.245	73.613	127.642	152.769
AMK58_20675	K14393 cation/acetate symporter | (GenBank) actP; cation/acetate symporter ActP	Transport: Ion	176.866	199.128	168.993	59.109	40.071	41.656
AMK58_20680	no KO assigned | (GenBank) hypothetical protein	Hypothetical	1337.255	1340.063	1151.927	492.796	383.416	842.382
AMK58_20685	no KO assigned | (GenBank) hypothetical protein	Hypothetical	15.463	24.019	18.525	307.594	80.126	366.633
AMK58_20690	no KO assigned | (GenBank) hypothetical protein	Hypothetical	1.416	2.820	3.590	132.151	16.978	130.434
AMK58_20695	no KO assigned | (GenBank) hypothetical protein	Hypothetical	3.232	2.758	3.951	43.342	12.541	69.317
AMK58_20700	no KO assigned | (GenBank) short-chain dehydrogenase	dehydrogenase	8.601	10.870	9.751	64.697	42.018	92.945
AMK58_20705	no KO assigned | (GenBank) hypothetical protein	Hypothetical	29.175	17.430	38.142	787.806	289.676	1246.689
AMK58_20710	no KO assigned | (GenBank) 2-methylisocitrate lyase	enzyme	2.883	5.167	6.578	146.933	85.220	232.826
AMK58_20715	no KO assigned | (GenBank) esterase	enzyme	15.061	14.702	13.945	30.591	200.267	136.823
AMK58_20720	no KO assigned | (GenBank) CopG family transcriptional regulator	Transcription	88.500	124.004	116.489	388.860	194.095	351.778
AMK58_20725	no KO assigned | (GenBank) hypothetical protein	Hypothetical	18.923	16.781	19.678	238.031	115.987	158.603
AMK58_20730	no KO assigned | (GenBank) hypothetical protein	Hypothetical	9.532	16.963	16.969	130.401	102.811	244.097
AMK58_20735	no KO assigned | (GenBank) hypothetical protein	Hypothetical	134.735	144.671	155.706	196.286	42.224	194.745
AMK58_20740	K05794 tellurite resistance protein TerC | (GenBank) tellurium resistance protein TerC	Defense	63.371	52.507	74.107	39.036	50.694	79.672
AMK58_20745	no KO assigned | (GenBank) hypothetical protein	Hypothetical	111.850	106.781	122.794	548.343	425.129	685.380
AMK58_20750	no KO assigned | (GenBank) hypothetical protein	Hypothetical	97.071	81.949	100.322	296.811	1189.259	525.882
AMK58_20755	K16092 vitamin B12 transporter | (GenBank) outer membrane cobalamin receptor protein BtuB	Transport: Inorganic	21.842	25.124	29.839	20.431	465.708	52.497
AMK58_20760	K02016 iron complex transport system substrate-binding protein | (GenBank) hypothetical protein	Transport: Metal	20.390	18.483	28.345	61.220	22.129	152.301
AMK58_20765	K02015 iron complex transport system permease protein | (GenBank) ABC transporter permease	Transport: Metal	42.970	73.045	72.119	303.860	138.762	357.942

AMK58_20770	K02013 iron complex transport system ATP-binding protein [EC:3.6.3.34] | (GenBank) ABC transporter	Transport: Metal	0.453	0.903	2.012	167.045	19.879	24.388
AMK58_20775	no KO assigned | (GenBank) hypothetical protein	Hypothetical	0.170	1.013	2.741	247.368	33.547	42.612
AMK58_20780	no KO assigned | (GenBank) ATPase	No COG	0.340	0.338	0.646	27.448	8.398	10.707
AMK58_20785	no KO assigned | (GenBank) hypothetical protein	Hypothetical	13.453	16.487	23.942	231.958	79.872	138.498
AMK58_20790	no KO assigned | (GenBank) hypothetical protein	Hypothetical	123.900	156.727	146.201	1249.372	288.064	1064.691
AMK58_20795	no KO assigned | (GenBank) hypothetical protein	Hypothetical	74.218	93.995	78.697	226.327	46.518	130.664
AMK58_20800	no KO assigned | (GenBank) hypothetical protein	Hypothetical	6.018	14.806	9.088	44.577	5.571	17.070
AMK58_20805	no KO assigned | (GenBank) hypothetical protein	Hypothetical	19.675	27.126	19.426	108.462	14.176	18.478
AMK58_20810	no KO assigned | (GenBank) hypothetical protein	Hypothetical	5.204	6.453	5.509	61.428	4.194	8.195
AMK58_20815	no KO assigned | (GenBank) hypothetical protein	Hypothetical	9.283	9.283	10.190	38.318	8.822	29.591
AMK58_20820	no KO assigned | (GenBank) hypothetical protein	Hypothetical	17.059	25.480	23.730	158.695	22.815	104.067
AMK58_20825	K09956 uncharacterized protein | (GenBank) hypothetical protein	Hypothetical	40.729	46.454	61.256	1584.660	267.608	693.188
AMK58_20830	no KO assigned | (GenBank) PadR family transcriptional regulator	Transcription	98.969	72.699	77.903	448.524	201.290	317.063
AMK58_20835	K02016 iron complex transport system substrate-binding protein | (GenBank) iron ABC transporter subs	Transport: Metal	44.004	46.668	36.764	73.245	37.755	41.286
AMK58_20840	K02015 iron complex transport system permease protein | (GenBank) iron ABC transporter permease	Transport: Metal	30.647	35.414	32.377	63.861	34.732	54.498
AMK58_20845	K02015 iron complex transport system permease protein | (GenBank) enterobactin ABC transporter perme	Transport: Metal	319.544	326.392	308.622	128.232	32.640	200.693
AMK58_20850	K02013 iron complex transport system ATP-binding protein [EC:3.6.3.34] | (GenBank) iron ABC transpor	Transport: Metal	182.968	185.842	200.662	230.778	20.082	209.895
AMK58_20855	K16422 4-hydroxymandelate oxidase [EC:1.1.3.46] | (GenBank) alpha-hydroxy-acid oxidizing enzyme	Biosynthesis: secondary metabolites	138.085	89.672	104.336	103.507	7.623	151.858
AMK58_20860	K07126 uncharacterized protein | (GenBank) hypothetical protein	Hypothetical	123.237	130.247	108.781	155.428	17.822	194.133
AMK58_20865	K07336 PKHD-type hydroxylase [EC:1.14.11.-] | (GenBank) PKHD-type hydroxylase	Oxidoreductase	143.104	162.150	132.043	231.829	16.244	109.818
AMK58_20870	K16090 catecholate siderophore receptor | (GenBank) hydrogenase	No COG	35.313	39.559	31.628	54.625	2.304	17.883
AMK58_20875	no KO assigned | (GenBank) anthranilate phosphoribosyltransferase	No COG	417.375	388.599	302.290	72.223	8.632	101.224
AMK58_20880	no KO assigned | (GenBank) hypothetical protein	Hypothetical	16943.310	14506.001	12768.918	1154.484	233.407	1412.727
AMK58_20885	no KO assigned | (GenBank) hypothetical protein	Hypothetical	236.413	310.884	279.714	706.922	158.660	444.140
AMK58_20890	K19302 undecaprenyl-diphosphatase [EC:3.6.1.27] | (GenBank) phosphoesterase	Cell Wall	65.222	82.884	71.607	104.262	139.334	106.088
AMK58_20895	K01251 adenosylhomocysteinase [EC:3.3.1.1] | (GenBank) adenosylhomocysteinase	Metabolism: Amino Acid	51.955	64.853	56.721	45.487	121.180	49.295
AMK58_20900	no KO assigned | (GenBank) hypothetical protein	Hypothetical	19.355	8.611	23.494	183.700	71.981	332.608
AMK58_20905	K04083 molecular chaperone Hsp33 | (GenBank) molecular chaperone Hsp33	Chaperone	576.893	353.284	471.162	166.805	180.453	232.000
AMK58_20910	K00611 ornithine carbamoyltransferase [EC:2.1.3.3] | (GenBank) ornithine carbamoyltransferase	Biosynthesis: Amino Acid	35.065	25.298	30.698	87.917	62.172	87.853
AMK58_20915	K00821 acetylornithine/N-succinyldiaminopimelate aminotransferase [EC:2.6.1.11 2.6.1.17] | (GenBank)	Biosynthesis: Amino Acid	120.236	125.256	122.794	144.914	666.444	252.242
AMK58_20920	K01992 ABC-2 type transport system permease protein | (GenBank) multidrug ABC transporter permease	Membrane Transport	338.829	343.684	340.775	165.010	294.361	222.584
AMK58_20925	K03565 regulatory protein | (GenBank) regulator	signal transduction	781.914	704.015	761.509	380.747	638.983	311.994
AMK58_20930	no KO assigned | (GenBank) hypothetical protein	Hypothetical	172.062	196.944	181.613	85.839	228.380	254.821
AMK58_20935	K02902 large subunit ribosomal protein L28 | (GenBank) 50S ribosomal protein L28	Ribosome	9.590	16.711	11.778	407.338	81.746	91.150
AMK58_20940	no KO assigned | (GenBank) hypothetical protein	Hypothetical	4.405	2.547	8.377	199.877	84.180	154.173
AMK58_20945	K07588 LAO/AO transport system kinase [EC:2.7.-.-] | (GenBank) protein kinase	Enzyme	831.983	816.204	756.578	53.739	415.457	135.617
AMK58_20950	no KO assigned | (GenBank) heme oxygenase	Enzyme	72.905	71.298	74.477	479.873	82.436	258.945
AMK58_20955	K06147 ATP-binding cassette, subfamily B, bacterial | (GenBank) metal ABC transporter permease	Membrane Transport	13.019	15.556	16.773	94.434	21.072	56.497
AMK58_20960	no KO assigned | (GenBank) pseudogene	Pseudogene	5.973	5.607	7.058	52.690	18.222	31.568
AMK58_20965	no KO assigned | (GenBank) hypothetical protein	Hypothetical	242.412	300.615	290.538	753.357	762.652	852.229
AMK58_20970	no KO assigned | (GenBank) branched-chain amino acid transporter	Transport: Amino Acid	396.440	405.421	400.030	535.835	127.663	344.095
AMK58_20975	no KO assigned | (GenBank) branched-chain amino acid ABC transporter permease	transport	43.680	53.582	57.796	174.867	39.577	114.118
AMK58_20980	K11068 hemolysin III | (GenBank) DNA-binding protein	Defense	61.822	49.320	57.211	112.301	49.282	103.594
AMK58_20985	K03695 ATP-dependent Clp protease ATP-binding subunit ClpB | (GenBank) ATP-dependent chaperone (	Chaperone	52.645	57.187	52.418	74.836	45.933	65.629
AMK58_20990	no KO assigned | (GenBank) hypothetical protein	Hypothetical	44.128	45.044	73.162	291.994	342.227	268.954
AMK58_20995	K07140 uncharacterized protein | (GenBank) hypothetical protein	Hypothetical	382.718	375.781	513.908	638.747	3115.935	835.597
AMK58_21000	no KO assigned | (GenBank) hypothetical protein	Hypothetical	184.376	260.936	309.349	342.146	258.826	309.895
AMK58_21005	K02493 release factor glutamine methyltransferase [EC:2.1.1.297] | (GenBank) protein-(glutamine-N5)	Translation	114.844	125.212	131.680	168.825	423.879	237.276
AMK58_21010	no KO assigned | (GenBank) hypothetical protein	Hypothetical	384.749	472.190	382.451	381.587	1538.231	361.758
AMK58_21015	K02835 peptide chain release factor 1 | (GenBank) peptide chain release factor 1	Enzyme	13.528	15.491	16.507	233.916	70.454	87.600
AMK58_21020	K02492 glutamyl-tRNA reductase [EC:1.2.1.70] | (GenBank) glutamyl-tRNA reductase	tRNA synthesis/modification	13.302	18.922	11.357	56.486	43.737	69.896
AMK58_21025	K03526 (E)-4-hydroxy-3-methylbut-2-enyl-diphosphate synthase [EC:1.17.7.1 1.17.7.3] | (GenBank) 4-hy	Metabolism: terpenoids and polyketide	35.673	47.715	46.169	189.573	127.954	201.236
AMK58_21030	no KO assigned | (GenBank) hypothetical protein	Hypothetical	32.675	39.265	48.758	177.437	154.718	85.232
AMK58_21035	K08484 phosphotransferase system, enzyme I, PtsP [EC:2.7.3.9] | (GenBank) peptidase	Membrane transport	39.727	45.269	41.575	99.172	181.044	124.028
AMK58_21040	K02371 enoyl-[acyl-carrier protein] reductase II [EC:1.3.1.9] | (GenBank) 2-nitropropane dioxygenase	Metabolism: Lipid	49.423	50.931	49.351	125.759	45.122	119.774
AMK58_21045	K00928 aspartate kinase [EC:2.7.2.4] | (GenBank) aspartate kinase	Signal Transduction	74.349	94.223	86.246	335.394	70.909	208.825
AMK58_21050	K00568 2-polyprenyl-6-hydroxyphenyl methylase / 3-demethylubiquinone-9 3-methyltransferase [EC:2.1.1	Metabolism: Co-Factors and Vitamins	161.751	174.669	182.498	262.647	140.380	233.527
AMK58_21055	K02806 PTS system, nitrogen regulatory IIA component [EC:2.7.1.-] | (GenBank) transcriptional regula	Nitrogen	1680.803	1668.918	1475.729	319.778	574.319	475.495
AMK58_21060	no KO assigned | (GenBank) ribose ABC transporter permease	Membrane Transport	59.422	54.524	50.521	238.796	101.215	131.075
AMK58_21065	K03092 RNA polymerase sigma-54 factor | (GenBank) RNA polymerase sigma-54 factor	Transcription	135.039	151.272	128.688	25.900	168.693	62.538
AMK58_21070	K06861 lipopolysaccharide export system ATP-binding protein [EC:3.6.3.-] | (GenBank) LPS export ABC	Membrane Transport	923.609	974.356	849.395	118.981	451.227	245.091
AMK58_21075	K09774 lipopolysaccharide export system protein LptA | (GenBank) hypothetical protein	Transport: polysacchharide	245.929	270.956	275.669	273.022	321.040	275.480
AMK58_21080	K11719 lipopolysaccharide export system protein LptC | (GenBank) LPS export ABC transporter periplas	Transport: polysacchharide	135.604	134.062	152.417	89.310	201.055	140.262
AMK58_21085	K06041 arabinose-5-phosphate isomerase [EC:5.3.1.13] | (GenBank) D-arabinose 5-phosphate	Biosynthesis: polysacchharide	237.651	277.484	241.184	196.041	355.330	174.846
AMK58_21090	K03684 ribonuclease D [EC:3.1.13.5] | (GenBank) 3'-5' exonuclease	trna synthesis/modification	279.225	346.103	320.203	120.281	591.474	141.277
AMK58_21095	K14228 tRNA Leu | (GenBank) tRNA-Leu	tRNA synthesis/modification	1553.097	1912.319	1959.668	2179.256	3111.372	1695.659
AMK58_21100	K00329 NADH dehydrogenase [EC:1.6.5.3] K00356 NADH dehydrogenase [EC:1.6.99.3] | (GenBank) 3	Energy	128.780	132.981	146.748	94.527	208.126	155.703

AMK58_21105	no KO assigned | (GenBank) MFS transporter	Transport: MFS	7.516	6.151	7.440	26.755	10.531	62.722
AMK58_21110	K06153 undecaprenyl-diphosphatase [EC:3.6.1.27] | (GenBank) UDP-diphosphatase	Cell Wall	373.352	357.614	364.471	277.397	224.104	256.076
AMK58_21115	K00266 glutamate synthase (NADPH/NADH) small chain [EC:1.4.1.13 1.4.1.14] | (GenBank) dihydropyr	Energy	31.668	32.948	39.326	155.134	263.639	94.527
AMK58_21120	K00265 glutamate synthase (NADPH/NADH) large chain [EC:1.4.1.13 1.4.1.14] | (GenBank) glutamate sy	Energy	20.153	21.313	24.553	31.239	28.128	33.548
AMK58_21125	no KO assigned | (GenBank) acetyltransferase	Acetyltransferase	307.669	361.678	392.257	441.172	385.037	584.911
AMK58_21130	K00799 glutathione S-transferase [EC:2.5.1.18] | (GenBank) glutathione S-transferase	Metabolism: Amino Acid	653.514	749.306	930.932	946.448	891.624	1414.195
AMK58_21135	no KO assigned | (GenBank) hypothetical protein	Hypothetical	30.672	31.345	27.820	201.381	79.841	207.886
AMK58_21140	no KO assigned | (GenBank) hypothetical protein	Hypothetical	171.854	238.854	216.906	179.445	187.208	282.870
AMK58_21145	K07240 chromate transporter | (GenBank) chromate transporter	transport	22.093	32.218	30.599	49.401	64.820	237.767
AMK58_21150	no KO assigned | (GenBank) histidine kinase	Signal Transduction	25.201	25.183	29.675	35.561	109.479	131.480
AMK58_21155	no KO assigned | (GenBank) two-component system response regulator	Signal Transduction	24.765	31.784	41.338	596.156	137.949	237.978
AMK58_21160	no KO assigned | (GenBank) peptidase	Peptidase	13.227	14.183	17.090	468.386	122.751	135.856
AMK58_21165	no KO assigned | (GenBank) hypothetical protein	Hypothetical	10.314	12.056	15.136	272.412	71.323	176.721
AMK58_21170	no KO assigned | (GenBank) mono-heme class I cytochrome C	Energy	4.866	0.000	5.715	93.554	37.965	61.310
AMK58_21175	no KO assigned | (GenBank) cytochrome C	Energy	3.104	0.000	5.635	194.321	103.418	126.925
AMK58_21180	no KO assigned | (GenBank) hypothetical protein	Hypothetical	2.925	3.494	11.679	442.681	128.204	172.472
AMK58_21185	K00059 3-oxoacyl-[acyl-carrier protein] reductase [EC:1.1.1.100] | (GenBank) 3-oxoacyl-ACP reductase	Metabolism	7.882	3.767	5.246	150.782	33.669	74.700
AMK58_21190	K02051 NitT/TauT family transport system substrate-binding protein | (GenBank) nitrate ABC transport	Transport: NitT/TauT	19.684	17.011	20.834	30.348	46.895	73.928
AMK58_21195	K02050 NitT/TauT family transport system permease protein | (GenBank) ABC transporter permease	Transport: NitT/TauT	78.123	97.803	83.219	186.720	109.676	137.970
AMK58_21200	K02049 NitT/TauT family transport system ATP-binding protein | (GenBank) nitrate ABC transporter ATP	Transport: NitT/TauT	421.642	393.108	356.555	208.675	108.380	260.506
AMK58_21205	no KO assigned | (GenBank) acyl-CoA reductase	enzyme	36.968	38.539	36.643	103.139	44.156	70.015
AMK58_21210	no KO assigned | (GenBank) long-chain fatty acid--CoA ligase	No COG	27.386	31.796	32.048	129.660	53.216	52.845
AMK58_21215	no KO assigned | (GenBank) hypothetical protein	Hypothetical	21.407	24.741	31.633	174.879	67.928	70.681
AMK58_21220	K01439 succinyl-diaminopimelate desuccinylase [EC:3.5.1.18] | (GenBank) succinyl-diaminopimelate des	Metabolism	169.920	202.810	193.210	153.723	392.063	211.249
AMK58_21225	K00127 formate dehydrogenase subunit gamma | (GenBank) formate dehydrogenase	Metabolism	89.631	90.689	94.501	324.372	176.041	400.038
AMK58_21230	K00124 formate dehydrogenase iron-sulfur subunit | (GenBank) formate dehydrogenase	Metabolism	118.790	154.680	128.151	405.465	371.902	330.278
AMK58_21235	K00123 formate dehydrogenase major subunit [EC:1.2.1.2] | (GenBank) formate dehydrogenase	Metabolism	5.060	4.461	8.243	53.895	107.233	74.462
AMK58_21240	no KO assigned | (GenBank) hypothetical protein	Hypothetical	15.605	16.075	28.652	437.541	894.586	437.103
AMK58_21245	no KO assigned | (GenBank) molecular chaperone TorD	Chaperone	45.692	47.799	80.634	535.856	1945.473	895.190
AMK58_21250	no KO assigned | (GenBank) hypothetical protein	Hypothetical	0.215	1.283	0.612	20.129	36.919	11.147
AMK58_21255	no KO assigned | (GenBank) molybdopterin-guanine dinucleotide biosynthesis protein A	No COG	4.612	8.267	11.402	253.937	593.885	133.227
AMK58_21260	no KO assigned | (GenBank) hypothetical protein	Hypothetical	18.381	28.648	29.635	122.844	150.691	179.891
AMK58_21265	no KO assigned | (GenBank) hypothetical protein	Hypothetical	2.668	5.978	5.708	259.140	93.728	98.352
AMK58_21270	K03517 quinolinate synthase [EC:2.5.1.72] | (GenBank) quinolinate synthetase	Metabolism: Co-Factors and Vitamens	2.874	4.293	5.238	82.296	62.056	42.754
AMK58_21275	K00278 L-aspartate oxidase [EC:1.4.3.16] | (GenBank) L-aspartate oxidase	Metabolism: Amino Acid	1.119	1.337	1.490	69.851	64.651	70.765
AMK58_21280	K00767 nicotinate-nucleotide pyrophosphorylase (carboxylating) [EC:2.4.2.19] | (GenBank) nicotinate-	Metabolism: Co-Factors and Vitamins	7.985	5.204	10.490	194.856	59.398	287.809
AMK58_21285	K01854 UDP-galactopyranose mutase [EC:5.4.99.9] | (GenBank) UDP-galactopyranose mutase	Metabolism: Carbohydrate	2.124	2.307	2.662	114.073	47.318	32.410
AMK58_21290	K00364 GMP reductase [EC:1.7.1.7] | (GenBank) GMP reductase	Metabolism: Nucleotide	3.827	2.771	7.278	124.902	82.256	81.299
AMK58_21295	no KO assigned | (GenBank) peptidase M20	Peptidases	12.864	15.253	26.049	214.932	253.202	258.867
AMK58_21300	K02083 allantoate deiminase [EC:3.5.3.9] | (GenBank) Zn-dependent hydrolase	Metabolism: Nucleotide	93.194	94.933	108.538	107.602	79.662	186.755
AMK58_21305	no KO assigned | (GenBank) OHCU decarboxylase	No COG	158.926	155.754	192.342	525.845	174.977	252.432
AMK58_21310	K07127 5-hydroxyisourate hydrolase [EC:3.5.2.17] | (GenBank) 5-hydroxyisourate hydrolase	Metabolism: Nucleotide	140.754	129.526	143.027	473.601	220.448	217.735
AMK58_21315	no KO assigned | (GenBank) cysteine desulfurase	No COG	24.093	27.938	24.068	75.305	47.307	53.920
AMK58_21320	no KO assigned | (GenBank) LysR family transcriptional regulator	Transcription	29.860	26.881	30.724	83.650	51.035	64.858
AMK58_21325	K06145 LacI family transcriptional regulator, gluconate utilization system Gnt-I transcriptional rep	Transcription	46.923	52.725	39.806	147.914	62.179	169.951
AMK58_21330	K03703 excinuclease ABC subunit C | (GenBank) excinuclease ABC subunit C	DNA Repair and Replication	11.943	17.251	15.911	112.168	24.489	97.726
AMK58_21335	no KO assigned | (GenBank) hypothetical protein	Hypothetical	570.205	640.686	760.881	1427.236	810.079	1294.309
AMK58_21340	no KO assigned | (GenBank) hypothetical protein	Hypothetical	131.837	212.184	186.237	1298.208	1868.408	1210.517
AMK58_21345	no KO assigned | (GenBank) hypothetical protein	Hypothetical	237.182	471.108	344.766	124.758	725.830	288.447
AMK58_21350	no KO assigned | (GenBank) hypothetical protein	Hypothetical	172.400	228.014	185.191	95.490	706.353	102.914
AMK58_21355	no KO assigned | (GenBank) hypothetical protein	Hypothetical	112.615	165.717	124.547	42.636	433.144	17.297
AMK58_21360	no KO assigned | (GenBank) hypothetical protein	Hypothetical	4.557	3.889	19.186	665.311	251.699	408.034
AMK58_21365	no KO assigned | (GenBank) YgiQ family radical SAM protein	No COG	9.188	13.550	23.057	177.921	146.421	183.395
AMK58_21370	no KO assigned | (GenBank) hypothetical protein	Hypothetical	1.614	2.411	2.302	52.981	260.392	247.742
AMK58_21375	no KO assigned | (GenBank) peptidase	peptidase	73.711	61.872	87.317	471.257	1050.571	519.265
AMK58_21380	K02303 uroporphyrin-III C-methyltransferase [EC:2.1.1.107] | (GenBank) uroporphyrin-III methyltransf	Metabolism: Co-Factors and Vitamins	17.448	19.942	19.042	103.246	87.318	56.838
AMK58_21385	K00372 assimilatory nitrate reductase catalytic subunit [EC:1.7.99.4] | (GenBank) nitrate reductase	Nitrogen	11.206	13.336	14.263	49.457	18.166	43.509
AMK58_21390	K00363 nitrite reductase (NADH) small subunit [EC:1.7.1.15] | (GenBank) nitrite reductase	Energy	2.713	6.079	7.094	276.216	46.508	161.329
AMK58_21395	K00362 nitrite reductase (NADH) large subunit [EC:1.7.1.15] | (GenBank) nitrite reductase	Energy	1.620	5.573	4.435	262.746	30.408	128.159
AMK58_21400	K15576 nitrate/nitrite transport system substrate-binding protein | (GenBank) nitrate ABC transporte	Nitrogen	0.951	0.000	1.150	48.390	6.411	22.518
AMK58_21405	K15577 nitrate/nitrite transport system permease protein | (GenBank) nitrate ABC transporter permeas	Nitrogen	1.663	5.299	7.169	360.611	32.777	498.839
AMK58_21410	K15578 nitrate/nitrite transport system ATP-binding protein [EC:3.6.3.-] | (GenBank) nitrate/sulfona	Nitrogen	2.182	2.607	4.840	175.531	22.887	240.183
AMK58_21415	K07183 response regulator NasT | (GenBank) two-component system response regulator	Signal Transduction	7.228	13.196	15.082	363.641	76.059	376.466
AMK58_21420	no KO assigned | (GenBank) antibiotic biosynthesis monooxygenase	Defense	37.836	36.598	45.911	757.350	152.295	504.211
AMK58_21425	no KO assigned | (GenBank) pseudogene	Pseudogene	12.205	15.743	17.934	53.505	31.490	55.299
AMK58_21430	no KO assigned | (GenBank) enamine deaminase RidA	Enzyme	73.434	96.085	65.205	110.156	95.845	404.340
AMK58_21435	no KO assigned | (GenBank) LysR family transcriptional regulator	Transcription	25.440	32.627	27.355	136.493	68.571	270.979

AMK58_21440	no KO assigned | (GenBank) hypothetical protein	Hypothetical	3.884	6.053	6.262	55.872	57.133	149.693
AMK58_21445	K03649 double-stranded uracil-DNA glycosylase [EC:3.2.2.28] | (GenBank) DNA glycosylase	DNA Repair and Replication	12.175	17.511	19.937	351.554	121.394	455.351
AMK58_21450	K03497 chromosome partitioning protein, ParB family | (GenBank) hypothetical protein	Cell Cycle/Shape/Homeostatsis	18.257	23.784	19.578	95.804	38.575	175.405
AMK58_21455	K03496 chromosome partitioning protein | (GenBank) chromosome partitioning protein	Cell Cycle/Shape/Homeostatsis	13.057	13.474	13.882	110.032	48.567	91.137
AMK58_21460	no KO assigned | (GenBank) plasmid replication protein	DNA Repair and Replication	55.181	48.373	53.059	143.331	49.705	137.565
AMK58_21465	no KO assigned | (GenBank) hypothetical protein	Hypothetical	63.616	54.879	59.000	146.871	80.184	169.628
AMK58_21470	K03546 exonuclease SbcC | (GenBank) hypothetical protein	DNA Repair and Replication	63.764	61.191	58.552	36.342	66.982	74.668
AMK58_21475	K03547 exonuclease SbcD | (GenBank) metallophosphatase	DNA Repair and Replication	143.421	151.346	125.285	95.608	90.079	227.219
AMK58_21480	K01977 16S ribosomal RNA | (GenBank) 16S ribosomal RNA	Ribosome	17.517	21.318	20.356	345.446	43.572	156.862
AMK58_21485	K14227 tRNA Ile | (GenBank) tRNA-Ile	tRNA synthesis/modification	199.695	131.519	203.327	1760.875	231.535	1131.445
AMK58_21490	K14218 tRNA Ala | (GenBank) tRNA-Ala	tRNA synthesis/modification	33.455	190.357	15.147	72.557	96.698	101.929
AMK58_21495	K01980 23S ribosomal RNA | (GenBank) 23S ribosomal RNA	Ribosome	0.000	0.000	0.000	0.000	0.000	0.000
AMK58_21500	K01985 5S ribosomal RNA | (GenBank) 5S ribosomal RNA	Ribosome	0.000	0.000	0.000	0.000	0.000	0.000
AMK58_21505	no KO assigned | (GenBank) hypothetical protein	Hypothetical	0.000	0.000	0.000	0.000	0.000	0.000
AMK58_21510	no KO assigned | (GenBank) hypothetical protein	Hypothetical	0.000	0.000	0.000	8.029	0.000	7.411
AMK58_21515	K01821 4-oxalocrotonate tautomerase [EC:5.3.2.6] | (GenBank) 4-oxalocrotonate tautomerase	Metabolism	93.529	110.991	81.010	384.441	379.477	833.409
AMK58_21520	no KO assigned | (GenBank) aldolase	No COG	27.014	32.084	35.588	36.841	44.633	147.278
AMK58_21525	no KO assigned | (GenBank) two-component system response regulator	Signal Transduction	59.818	69.252	63.070	13.714	18.165	43.857
AMK58_21530	no KO assigned | (GenBank) histidine kinase	Signal Transduction	31.173	27.096	29.367	50.063	27.877	123.875
AMK58_21535	K02380 FdhE protein | (GenBank) formate dehydrogenase accessory protein FdhE	dehydrogenase	44.125	48.224	56.408	319.686	105.094	252.357
AMK58_21540	K00123 formate dehydrogenase major subunit [EC:1.2.1.2] | (GenBank) formate dehydrogenase	Metabolism	20.777	19.944	21.176	200.229	52.925	174.041
AMK58_21545	K00124 formate dehydrogenase iron-sulfur subunit | (GenBank) formate dehydrogenase	Metabolism	19.789	18.667	15.349	79.872	24.292	82.814
AMK58_21550	K00127 formate dehydrogenase subunit gamma | (GenBank) formate dehydrogenase	Metabolism	1078.579	867.341	735.452	623.378	163.183	829.873
AMK58_21555	K02379 FdhD protein | (GenBank) hypothetical protein	No COG	215.870	193.456	179.669	149.613	62.947	251.084
AMK58_21560	no KO assigned | (GenBank) histidine kinase	Signal Transduction	97.178	70.251	59.218	39.429	17.395	101.788
AMK58_21565	no KO assigned | (GenBank) regulator	signal transduction	110.343	109.271	115.550	206.934	47.913	195.946
AMK58_21570	K03412 two-component system, chemotaxis family, response regulator CheB [EC:3.1.1.61] | (GenBank) gl	Chemotaxis	185.312	236.430	260.190	292.535	80.144	240.756
AMK58_21575	K03407 two-component system, chemotaxis family, sensor kinase CheA [EC:2.7.13.3] | (GenBank) histidi	Chemotaxis	37.196	43.228	42.297	44.370	18.676	44.182
AMK58_21580	no KO assigned | (GenBank) chemotaxis protein	Chemotaxis	46.423	59.093	62.811	128.724	59.242	76.788
AMK58_21585	K03408 purine-binding chemotaxis protein CheW | (GenBank) hypothetical protein	Chemotaxis	257.791	351.872	339.849	533.118	231.814	297.953
AMK58_21590	K00575 chemotaxis protein methyltransferase CheR [EC:2.1.1.80] | (GenBank) chemotaxis protein	Chemotaxis	451.127	454.281	488.044	206.174	70.629	267.851
AMK58_21595	K03408 purine-binding chemotaxis protein CheW | (GenBank) hypothetical protein	Chemotaxis	61.114	84.047	75.650	57.151	26.182	32.291
AMK58_21600	K01579 aspartate 1-decarboxylase [EC:4.1.1.11] | (GenBank) aspartate decarboxylase	Metabolism: Amino Acid	329.824	330.914	410.988	649.489	135.780	448.563
AMK58_21605	no KO assigned | (GenBank) hypothetical protein	Hypothetical	0.000	0.000	0.000	0.000	0.000	0.000
AMK58_21610	no KO assigned | (GenBank) hypothetical protein	Hypothetical	0.000	0.000	0.000	0.000	0.000	0.000
AMK58_21615	no KO assigned | (GenBank) endonuclease	DNA Repair and Replication	0.000	0.000	0.000	0.000	0.000	0.000
AMK58_21620	no KO assigned | (GenBank) hypothetical protein	Hypothetical	0.000	0.000	0.000	0.000	0.000	0.000
AMK58_21625	K03411 chemotaxis protein CheD [EC:3.5.1.44] | (GenBank) chemotaxis protein	Chemotaxis	0.000	0.000	0.000	0.000	0.000	0.000
AMK58_21630	K07259 D-alanyl-D-alanine carboxypeptidase / D-alanyl-D-alanine-endopeptidase (penicillin-binding pr	Cell Wall	0.000	0.000	0.000	0.000	0.000	0.000
AMK58_21635	no KO assigned | (GenBank) hypothetical protein	Hypothetical	0.000	0.000	0.000	0.000	0.000	0.000
AMK58_21640	no KO assigned | (GenBank) hypothetical protein	Hypothetical	0.000	0.000	0.000	0.000	0.000	0.000
AMK58_21645	no KO assigned | (GenBank) hypothetical protein	Hypothetical	0.000	0.000	0.000	0.000	0.000	0.000
AMK58_21650	no KO assigned | (GenBank) hypothetical protein	Hypothetical	0.000	0.000	0.000	0.000	0.000	0.000
AMK58_21655	no KO assigned | (GenBank) hypothetical protein	Hypothetical	0.000	0.000	0.000	0.000	0.000	0.000
AMK58_21660	no KO assigned | (GenBank) hypothetical protein	Hypothetical	0.000	0.000	0.000	0.000	0.000	0.000
AMK58_21665	no KO assigned | (GenBank) hypothetical protein	Hypothetical	0.000	0.000	0.000	0.000	0.000	0.000
AMK58_21670	no KO assigned | (GenBank) hypothetical protein	Hypothetical	0.000	0.000	0.000	0.000	0.000	0.000
AMK58_21675	no KO assigned | (GenBank) hypothetical protein	Hypothetical	0.000	0.000	0.000	0.000	0.000	0.000
AMK58_21680	no KO assigned | (GenBank) hypothetical protein	Hypothetical	0.000	0.000	0.000	0.000	0.000	0.000
AMK58_21685	no KO assigned | (GenBank) SapC family protein	No COG	0.000	0.000	0.000	0.000	0.000	0.000
AMK58_21690	no KO assigned | (GenBank) hypothetical protein	Hypothetical	1480.731	1544.406	1598.102	2087.910	432.331	1611.134
AMK58_21695	no KO assigned | (GenBank) hypothetical protein	Hypothetical	901.226	785.580	837.088	531.464	319.380	379.966
AMK58_21700	K03498 trk system potassium uptake protein | (GenBank) potassium transporter TrkH	Transport: Inorganic	133.723	133.237	136.798	126.182	135.158	112.578
AMK58_21705	K14205 phosphatidylglycerol lysyltransferase [EC:2.3.2.3] | (GenBank) hypothetical protein	Defense	3.548	4.416	3.725	21.189	124.627	97.533
AMK58_21710	no KO assigned | (GenBank) hypothetical protein	Hypothetical	361.711	295.370	349.674	701.454	318.685	1255.549
AMK58_21715	K02168 choline/glycine/proline betaine transport protein | (GenBank) beta-aspartyl peptidase	Transport	2.435	4.000	5.556	166.100	58.844	118.592
AMK58_21720	no KO assigned | (GenBank) hypothetical protein	Hypothetical	15.587	14.135	17.863	41.479	7.665	49.747
AMK58_21725	no KO assigned | (GenBank) hypothetical protein	Hypothetical	27.009	31.531	32.174	642.506	271.140	941.050
AMK58_21730	no KO assigned | (GenBank) phosphoglycerate mutase	Enzyme	73.278	97.393	98.415	453.272	97.667	342.533
AMK58_21735	no KO assigned | (GenBank) carbohydrate kinase	No COG	13.408	21.037	27.943	183.956	41.254	99.712
AMK58_21740	no KO assigned | (GenBank) hypothetical protein	Hypothetical	124.438	128.597	141.980	773.987	727.343	609.375
AMK58_21745	no KO assigned | (GenBank) arabinose ABC transporter permease	Membrane Transport	15.921	18.250	19.275	166.526	49.630	71.156
AMK58_21750	K02297 cytochrome o ubiquinol oxidase subunit II [EC:1.10.3.10] | (GenBank) cytochrome O ubiquinol o	Energy	562.774	568.699	606.022	255.156	873.837	242.873
AMK58_21755	K02298 cytochrome o ubiquinol oxidase subunit I [EC:1.10.3.10] | (GenBank) cytochrome o ubiquinol ox	Energy	638.191	752.189	736.658	94.305	361.966	139.624
AMK58_21760	K02299 cytochrome o ubiquinol oxidase subunit III | (GenBank) cytochrome o ubiquinol oxidase subunit	Energy	3954.348	4466.379	4576.521	274.390	1308.506	695.154
AMK58_21765	K02300 cytochrome o ubiquinol oxidase subunit IV | (GenBank) hypothetical protein	Energy	1528.081	1715.053	1771.965	106.816	607.663	291.841
AMK58_21770	K14998 surfeit locus 1 family protein | (GenBank) Surfeit locus 1 family protein	No COG	717.442	630.220	723.322	32.020	345.003	70.189

AMK58_21775	K15011 two-component system, sensor histidine kinase RegB [EC:2.7.13.3] | (GenBank) histidine kinase	Signal Transduction	223.530	309.070	319.281	149.653	190.324	67.702
AMK58_21780	K15012 two-component system, response regulator RegA | (GenBank) two-component system response re	Signal Transduction	139.213	131.396	151.767	421.866	1086.452	385.375
AMK58_21785	no KO assigned | (GenBank) hypothetical protein	Hypothetical	86.070	88.574	100.631	179.287	598.069	309.696
AMK58_21790	K03530 DNA-binding protein HU-beta | (GenBank) DNA-binding protein HU	DNA Repair and Replication	314.794	317.261	278.305	272.586	991.331	343.112
AMK58_21795	K02569 cytochrome c-type protein NapC | (GenBank) cytochrome C	Energy	218.710	311.664	193.541	85.460	402.139	85.142
AMK58_21800	K02568 cytochrome c-type protein NapB | (GenBank) cytochrome C	Energy	135.864	131.791	148.248	231.694	613.406	300.083
AMK58_21805	K02567 periplasmic nitrate reductase NapA [EC:1.7.99.4] | (GenBank) nitrate reductase	Nitrogen	11.704	9.285	11.637	30.837	49.404	39.270
AMK58_21810	K02570 periplasmic nitrate reductase NapD | (GenBank) nitrate reductase	Nitrogen	694.162	649.872	707.525	928.836	1251.625	1521.878
AMK58_21815	K02572 ferredoxin-type protein NapF | (GenBank) ferredoxin-type protein NapF	ferredoxin	47.203	57.812	52.286	77.773	152.276	78.348
AMK58_21820	K02571 periplasmic nitrate reductase NapE | (GenBank) nitrate reductase	Nitrogen	121.836	250.217	191.141	596.722	869.627	303.622
AMK58_21825	no KO assigned | (GenBank) hypothetical protein	Hypothetical	123.917	152.822	177.272	153.049	732.859	134.934
AMK58_21830	no KO assigned | (GenBank) oxalate/formate MFS antiporter	Transport	1683.933	2438.944	2157.636	60.774	1710.228	194.550
AMK58_21835	no KO assigned | (GenBank) hypothetical protein	Hypothetical	970.616	861.999	910.639	352.754	91.863	489.309
AMK58_21840	K06890 uncharacterized protein | (GenBank) hypothetical protein	Hypothetical	18.756	24.012	23.406	115.527	23.906	148.295
AMK58_21845	no KO assigned | (GenBank) hypothetical protein	Hypothetical	3828.717	3208.668	3454.994	47.122	2116.035	97.730
AMK58_21850	K03088 RNA polymerase sigma-70 factor, ECF subfamily | (GenBank) hypothetical protein	Transcription	1663.498	1637.648	1927.804	175.358	1680.325	112.840
AMK58_21855	K03530 DNA-binding protein HU-beta | (GenBank) DNA-binding protein HU	No COG	2817.856	3428.959	3977.460	402.904	3788.166	613.741
AMK58_21860	no KO assigned | (GenBank) hypothetical protein	Hypothetical	960.911	1377.820	1065.916	339.818	229.702	298.198
AMK58_21865	no KO assigned | (GenBank) hypothetical protein	Hypothetical	39.390	40.508	30.084	95.582	33.388	99.995
AMK58_21870	K01420 CRP/FNR family transcriptional regulator, anaerobic regulatory protein | (GenBank) Crp/Fnr fa	Transcription	48.959	57.315	50.168	76.218	54.474	102.147
AMK58_21875	no KO assigned | (GenBank) hypothetical protein	Hypothetical	169.023	71.340	59.936	161.070	6.971	107.034
AMK58_21880	K01601 ribulose-bisphosphate carboxylase large chain [EC:4.1.1.39] | (GenBank) ribulose 1,5-bisphosp	Metabolism	2.475	2.843	4.706	196.102	14.123	77.318
AMK58_21885	no KO assigned | (GenBank) glyoxalase	No COG	12.556	13.395	22.171	555.336	30.999	227.242
AMK58_21890	no KO assigned | (GenBank) ABC transporter permease	Membrane Transport	5.625	3.908	4.540	13.932	4.706	33.177
AMK58_21895	no KO assigned | (GenBank) ABC transporter substrate-binding protein	Membrane Transport	25.683	29.455	27.036	360.925	25.260	260.580
AMK58_21900	K02529 LacI family transcriptional regulator | (GenBank) LacI family transcriptional regulator	Transcription	24.416	17.984	25.441	123.656	20.301	175.744
AMK58_21905	no KO assigned | (GenBank) hypothetical protein	Hypothetical	13.158	16.629	20.690	266.581	35.979	178.787
AMK58_21910	no KO assigned | (GenBank) 3-keto-5-aminohexanoate cleavage protein	No COG	25.496	19.902	29.967	179.863	32.691	106.923
AMK58_21915	K01652 acetolactate synthase I/II/III large subunit [EC:2.2.1.6] | (GenBank) decarboxylase	Metabolism	64.839	59.345	63.881	157.890	26.550	128.225
AMK58_21920	no KO assigned | (GenBank) acetylpolyamine aminohydrolase	No COG	43.664	52.447	52.573	223.884	21.785	115.441
AMK58_21925	K06016 beta-ureidopropionase / N-carbamoyl-L-amino-acid hydrolase [EC:3.5.1.6 3.5.1.87] | (GenBank)	Metabolism: Nucleotide	28.152	27.640	30.886	99.421	9.626	63.619
AMK58_21930	K00077 2-dehydropantoate 2-reductase [EC:1.1.1.169] | (GenBank) hypothetical protein	Metabolism: Co-Factors and Vitamins	40.995	44.877	48.057	306.294	13.595	178.026
AMK58_21935	K02031 peptide/nickel transport system ATP-binding protein K02032 peptide/nickel transport system AT	Quorum sensing	7.708	8.255	12.100	90.785	3.024	36.344
AMK58_21940	K02034 peptide/nickel transport system permease protein | (GenBank) ABC transporter permease	Quorum sensing	121.768	148.317	150.459	298.177	21.981	294.370
AMK58_21945	K02033 peptide/nickel transport system permease protein | (GenBank) ABC transporter permease	Quorum sensing	74.422	70.787	78.976	219.058	14.705	157.361
AMK58_21950	K02035 peptide/nickel transport system substrate-binding protein | (GenBank) peptide ABC transporter	Quorum sensing	127.237	131.111	117.691	117.688	15.713	148.160
AMK58_21955	K01438 acetylornithine deacetylase [EC:3.5.1.16] | (GenBank) acetylornithine deacetylase	Metabolism: Amino Acid	901.335	779.506	815.294	89.733	113.307	233.682
AMK58_21960	no KO assigned | (GenBank) LysR family transcriptional regulator	Transcription	183.854	257.048	202.466	231.671	51.631	214.313
AMK58_21965	no KO assigned | (GenBank) hypothetical protein	Hypothetical	20.566	25.449	29.987	70.891	22.682	78.198
AMK58_21970	no KO assigned | (GenBank) pseudogene	Pseudogene	19.743	16.292	20.742	379.018	83.372	558.820
AMK58_21975	no KO assigned | (GenBank) hypothetical protein	Hypothetical	19.991	22.534	48.146	109.387	44.940	158.524
AMK58_21980	no KO assigned | (GenBank) hypothetical protein	Hypothetical	7.255	9.753	13.538	302.140	22.835	144.479
AMK58_21985	no KO assigned | (GenBank) hypothetical protein	Hypothetical	2.368	1.769	10.414	164.301	15.417	193.796
AMK58_21990	no KO assigned | (GenBank) hypothetical protein	Hypothetical	1.523	1.706	6.697	212.550	11.448	55.958
AMK58_21995	no KO assigned | (GenBank) oxidoreductase	Oxidoreductase	2.323	1.614	8.168	104.795	8.138	86.820
AMK58_22000	K16554 polysaccharide biosynthesis transport protein | (GenBank) hypothetical protein	Transport: polysaccharide	2.577	3.387	10.537	175.235	10.255	138.366
AMK58_22005	K08678 UDP-glucuronate decarboxylase [EC:4.1.1.35] | (GenBank) NAD-dependent dehydratase	Metabolism: Carbohydrate	11.367	10.682	47.845	383.566	29.573	418.761
AMK58_22010	K20997 polysaccharide biosynthesis protein PslA | (GenBank) hypothetical protein	Biosynthesis: polysaccharides	24.486	8.577	71.437	50.322	18.648	120.923
AMK58_22015	K12454 CDP-paratose 2-epimerase [EC:5.1.3.10] | (GenBank) NAD-dependent epimerase	Metabolism: Carbohydrate	9.529	4.688	34.429	296.440	37.552	204.684
AMK58_22020	no KO assigned | (GenBank) UDP-N-acetyl-D-mannosaminuronic acid transferase	No COG	12.903	6.274	45.363	143.093	19.394	253.615
AMK58_22025	no KO assigned | (GenBank) pseudogene	Pseudogene	3.704	6.407	14.645	98.200	6.027	106.304
AMK58_22030	K12454 CDP-paratose 2-epimerase [EC:5.1.3.10] | (GenBank) CDP-paratose 2-epimerase	Metabolism	7.735	9.377	34.003	209.490	50.531	183.166
AMK58_22035	K01784 UDP-glucose 4-epimerase [EC:5.1.3.2] | (GenBank) hypothetical protein	Metabolism: Carbohydrate	8.888	8.611	37.459	144.970	37.399	199.925
AMK58_22040	no KO assigned | (GenBank) pseudogene	Pseudogene	8.741	6.111	29.885	117.965	24.877	168.663
AMK58_22045	no KO assigned | (GenBank) hypothetical protein	Hypothetical	27.134	17.709	123.357	523.210	68.175	453.096
AMK58_22050	no KO assigned | (GenBank) hypothetical protein	Hypothetical	2.489	3.187	4.226	121.223	12.590	42.699
AMK58_22055	no KO assigned | (GenBank) hypothetical protein	Hypothetical	3.476	5.768	10.649	239.528	24.093	243.340
AMK58_22060	no KO assigned | (GenBank) hypothetical protein	Hypothetical	53.395	49.715	138.195	158.656	58.927	150.079
AMK58_22065	no KO assigned | (GenBank) hypothetical protein	Hypothetical	7.959	12.488	20.486	42.862	21.779	101.833
AMK58_22070	no KO assigned | (GenBank) glucoamylase	Metabolism: Carbohydrates	4.110	4.428	9.609	103.340	21.357	148.693
AMK58_22075	no KO assigned | (GenBank) hypothetical protein	Hypothetical	4.998	7.324	10.848	249.973	181.140	242.551
AMK58_22080	K12574 ribonuclease J [EC:3.1.-.-] | (GenBank) MBL fold metallo-hydrolase	Genetic Information Processing	2.963	6.698	9.060	152.746	551.033	144.124
AMK58_22085	no KO assigned | (GenBank) hypothetical protein	Hypothetical	1391.259	1523.431	1793.414	1080.256	9282.793	1785.768
AMK58_22090	no KO assigned | (GenBank) hypothetical protein	Hypothetical	106.766	116.175	117.910	113.039	580.252	224.539
AMK58_22095	no KO assigned | (GenBank) methyltransferase	methyltransferase	58.273	75.276	63.830	154.274	175.483	115.738
AMK58_22100	no KO assigned | (GenBank) hypothetical protein	Hypothetical	1.755	2.995	2.264	107.117	63.397	84.841
AMK58_22105	no KO assigned | (GenBank) hypothetical protein	Hypothetical	4.484	15.309	18.273	949.088	399.639	1058.543

AMK58_22110	no KO assigned | (GenBank) hypothetical protein	Hypothetical	22.363	31.072	32.736	166.087	65.708	253.282
AMK58_22115	K02433 aspartyl-tRNA(Asn)/glutamyl-tRNA(Gln) amidotransferase subunit A [EC:6.3.5.6 6.3.5.7] | (Gen	tRNA synthesis/modification	8.124	11.359	8.135	124.447	10.005	64.589
AMK58_22120	no KO assigned | (GenBank) hypothetical protein	Hypothetical	8.761	9.998	8.704	111.289	8.741	40.983
AMK58_22125	K07127 5-hydroxyisourate hydrolase [EC:3.5.2.17] | (GenBank) 5-hydroxyisourate hydrolase	Metabolism: Nucleotide	33.236	30.393	31.601	149.465	16.392	166.046
AMK58_22130	K02053 putative spermidine/putrescine transport system permease protein | (GenBank) ABC transporter	Membrane Transport	9.348	11.524	12.697	119.453	16.511	128.505
AMK58_22135	K02054 putative spermidine/putrescine transport system permease protein | (GenBank) ABC transporter	Membrane Transport	8.833	16.237	12.403	232.994	27.952	197.700
AMK58_22140	K02055 putative spermidine/putrescine transport system substrate-binding protein | (GenBank) ABC tra	Quorum sensing	7.401	4.836	7.367	219.346	41.465	257.673
AMK58_22145	K02052 putative spermidine/putrescine transport system ATP-binding protein | (GenBank) Fe3+/spermidi	Quorum sensing	1.237	1.343	3.314	155.409	22.366	89.336
AMK58_22150	K07395 putative proteasome-type protease | (GenBank) peptidase	peptidase	236.055	245.055	202.985	127.321	92.951	262.115
AMK58_22155	no KO assigned | (GenBank) GntR family transcriptional regulator	Transcription	21.783	21.186	27.455	220.093	64.345	210.447
AMK58_22160	no KO assigned | (GenBank) ABC transporter substrate-binding protein	Membrane Transport	6.806	7.847	8.174	104.445	63.215	274.917
AMK58_22165	K02050 NitT/TauT family transport system permease protein | (GenBank) ABC transporter permease	Transport: NitT/TauT	1.650	2.689	3.994	164.598	25.802	169.865
AMK58_22170	K02049 NitT/TauT family transport system ATP-binding protein | (GenBank) ABC transporter ATP-bindi	Transport: NitT/TauT	1.392	4.619	2.793	142.715	18.076	155.428
AMK58_22175	K12960 5-methylthioadenosine/S-adenosylhomocysteine deaminase [EC:3.5.4.31 3.5.4.28] | (GenBank) hy	Metabolism: Amino Acid	0.727	0.543	3.111	166.569	10.217	90.102
AMK58_22180	K01799 maleate isomerase [EC:5.2.1.1] | (GenBank) Asp/Glu/hydantoin racemase	Metabolism: Carbohydrates	2.646	0.988	6.605	186.115	35.139	215.030
AMK58_22185	K01464 dihydropyrimidinase [EC:3.5.2.2] | (GenBank) dihydropyrimidinase	Metabolism	6.151	9.061	9.934	182.501	25.287	154.302
AMK58_22190	K01426 amidase [EC:3.5.1.4] | (GenBank) glutamyl-tRNA amidotransferase	tRNA synthesis/modification	14.053	19.453	16.376	145.772	32.362	92.168
AMK58_22195	no KO assigned | (GenBank) hypothetical protein	Hypothetical	66.352	104.212	86.502	248.777	49.978	231.517
AMK58_22200	no KO assigned | (GenBank) hypothetical protein	Hypothetical	7.532	5.519	8.242	182.174	11.820	161.421
AMK58_22205	K02182 crotonobetaine/carnitine-CoA ligase [EC:6.2.1.-] | (GenBank) ATP-dependent acyl-CoA ligase	Enzyme	7.676	9.928	9.556	210.418	27.841	130.468
AMK58_22210	no KO assigned | (GenBank) ABC transporter permease	Membrane Transport	12.586	13.486	15.024	226.694	21.452	149.566
AMK58_22215	K02052 putative spermidine/putrescine transport system ATP-binding protein | (GenBank) ABC transport	Membrane Transport	26.540	20.967	27.007	162.333	34.763	212.133
AMK58_22220	K02055 putative spermidine/putrescine transport system substrate-binding protein | (GenBank) ABC tra	Quorum sensing	34.400	32.910	42.114	150.892	34.624	179.506
AMK58_22225	no KO assigned | (GenBank) enoyl-CoA hydratase	hydratase	29.052	33.775	37.178	237.073	43.599	221.045
AMK58_22230	no KO assigned | (GenBank) IclR family transcriptional regulator	Transcription	26.541	22.068	19.772	73.121	27.526	220.099
AMK58_22235	K01977 16S ribosomal RNA | (GenBank) 16S ribosomal RNA	Ribosome	0.000	0.000	0.000	0.000	0.000	0.000
AMK58_22240	K14227 tRNA Ile | (GenBank) tRNA-Ile	tRNA synthesis/modification	0.000	0.000	0.000	0.000	0.000	0.000
AMK58_22245	K14218 tRNA Ala | (GenBank) tRNA-Ala	tRNA synthesis/modification	0.000	0.000	0.000	0.000	0.000	0.000
AMK58_22250	K01980 23S ribosomal RNA | (GenBank) 23S ribosomal RNA	Ribosome	3.167	27.852	3.596	4.124	6.229	0.652
AMK58_22255	K01985 5S ribosomal RNA | (GenBank) 5S ribosomal RNA	Ribosome	0.000	0.000	0.000	11.185	0.000	120.980
AMK58_22260	K14230 tRNA Met | (GenBank) tRNA-Met	tRNA synthesis/modification	113.213	169.096	103.159	267.501	625.675	304.732
AMK58_22265	no KO assigned | (GenBank) hypothetical protein	Hypothetical	6.342	3.445	8.223	128.204	25.923	375.836
AMK58_22270	K20276 large repetitive protein | (GenBank) hypothetical protein	Quorum sensing	10.032	14.288	17.436	142.729	27.956	134.559
AMK58_22275	no KO assigned | (GenBank) riboflavin biosynthesis protein RibD	Biosynthesis: Co-Factors and Vitamins	29.761	15.612	57.007	190.578	116.511	243.116
AMK58_22280	no KO assigned | (GenBank) pyrroloquinoline quinone biosynthesis protein PqqA	Biosynthesis: quinone	735.417	446.516	1219.408	242.278	2021.236	1054.780
AMK58_22285	no KO assigned | (GenBank) hypothetical protein	Hypothetical	9.913	6.345	15.484	69.711	68.842	265.902
AMK58_22290	K17226 sulfur-oxidizing protein SoxY | (GenBank) quinoprotein dehydrogenase-associated SoxYZ-like ca	Energy	8.742	13.927	15.238	144.230	62.724	186.846
AMK58_22295	no KO assigned | (GenBank) MBL fold metallo-hydrolase	No COG	4.235	5.209	12.317	280.989	77.988	150.963
AMK58_22300	no KO assigned | (GenBank) pseudogene	Pseudogene	0.592	0.707	2.926	181.845	26.474	77.261
AMK58_22305	no KO assigned | (GenBank) hypothetical protein	Hypothetical	2.303	1.448	4.148	134.749	32.014	119.125
AMK58_22310	K01814 phosphoribosylformimino-5-aminoimidazole carboxamide ribotide isomerase [EC:5.3.1.16] | (Gen	Metabolism: Amino Acid	3.762	2.043	9.431	233.658	37.676	95.461
AMK58_22315	K10713 5,6,7,8-tetrahydromethanopterin hydro-lyase [EC:4.2.1.147] | (GenBank) aldehyde-activating pr	Metabolism	36.647	30.486	60.647	96.317	190.583	155.070
AMK58_22320	K05966 triphosphoribosyl-dephospho-CoA synthase [EC:2.4.2.52] | (GenBank) hypothetical protein	Signal transduction	4.408	4.115	8.644	203.701	36.075	175.876
AMK58_22325	no KO assigned | (GenBank) alpha-L-glutamate ligase	Enzyme	0.682	3.258	4.278	118.534	7.663	40.458
AMK58_22330	K01499 methenyltetrahydromethanopterin cyclohydrolase [EC:3.5.4.27] | (GenBank) methenyltetrahydrom	Metabolism: Carbon	4.280	8.037	9.419	200.521	27.081	101.270
AMK58_22335	no KO assigned | (GenBank) pseudogene	Pseudogene	3.793	3.148	8.216	164.162	18.596	84.129
AMK58_22340	K10714 methylene-tetrahydromethanopterin dehydrogenase [EC:1.5.1.-] | (GenBank) methylenetetrahydror	Energy	6.749	8.064	12.064	162.186	21.241	60.203
AMK58_22345	no KO assigned | (GenBank) GHMP kinase	No COG	14.344	13.073	29.473	181.401	12.571	75.635
AMK58_22350	K01724 4a-hydroxytetrahydrobiopterin dehydratase [EC:4.2.1.96] | (GenBank) pterin dehydratase	dehydratase	99.294	133.955	86.491	136.442	151.215	250.676
AMK58_22355	K00201 formylmethanofuran dehydrogenase subunit B [EC:1.2.99.5] | (GenBank) formylmethanofuran del	Metabolism: Carbon	16.088	21.112	21.397	112.733	93.342	100.629
AMK58_22360	K00200 formylmethanofuran dehydrogenase subunit A [EC:1.2.99.5] | (GenBank) formylmethanofuran de	Metabolism	10.346	15.782	12.489	148.916	61.531	168.064
AMK58_22365	K00672 formylmethanofuran--tetrahydromethanopterin N-formyltransferase [EC:2.3.1.101] | (GenBank) fo	Metabolism: Carbon	6.995	8.841	9.593	218.230	38.409	106.282
AMK58_22370	K00202 formylmethanofuran dehydrogenase subunit C [EC:1.2.99.5] | (GenBank) hypothetical protein	Metabolism: Carbon	7.778	8.768	10.884	178.699	27.219	61.238
AMK58_22375	no KO assigned | (GenBank) branched-chain amino acid ABC transporter substrate-binding protein	Membrane Transport	6.404	6.074	10.536	212.435	26.739	93.982
AMK58_22380	no KO assigned | (GenBank) hypothetical protein	Hypothetical	60.229	58.252	79.562	95.724	66.174	190.893
AMK58_22385	K01990 ABC-2 type transport system ATP-binding protein | (GenBank) ABC transporter ATP-binding pro	Membrane Transport	12.341	13.949	16.491	228.981	31.305	94.486
AMK58_22390	K01992 ABC-2 type transport system permease protein | (GenBank) multidrug ABC transporter permease	Membrane Transport	10.386	15.070	15.660	255.005	43.697	180.099
AMK58_22395	no KO assigned | (GenBank) hypothetical protein	Hypothetical	15.217	11.858	12.581	380.206	1445.711	278.527
AMK58_22400	no KO assigned | (GenBank) hypothetical protein	Hypothetical	5.381	3.572	9.096	339.189	682.482	149.693
AMK58_22405	no KO assigned | (GenBank) hypothetical protein	Hypothetical	1.546	3.078	6.368	168.627	221.031	111.792
AMK58_22410	K09154 uncharacterized protein | (GenBank) tetrahydromethanopterin synthesis protein	Biosynthesis	3.776	5.640	6.732	223.835	188.620	137.000
AMK58_22415	no KO assigned | (GenBank) dihydropteroate synthase	No COG	4.868	2.644	8.668	227.511	107.641	148.298
AMK58_22420	no KO assigned | (GenBank) hypothetical protein	Hypothetical	2.829	2.253	6.635	166.481	46.212	75.021
AMK58_22425	K01633 dihydroneopterin aldolase / 7,8-dihydroneopterin epimerase [EC:4.1.2.25 5.1.99.8] | (GenBank)	Metabolism: Co-Factors and Vitamins	21.988	24.943	19.848	121.173	119.825	191.246
AMK58_22430	no KO assigned | (GenBank) hypothetical protein	Hypothetical	4.871	2.771	7.498	265.339	48.493	176.362
AMK58_22435	no KO assigned | (GenBank) hypothetical protein	Hypothetical	18.065	25.260	34.536	287.816	76.687	191.928
AMK58_22440	no KO assigned | (GenBank) hypothetical protein	Hypothetical	12.170	13.672	19.681	161.071	56.472	163.582

AMK58_22445	no KO assigned | (GenBank) hypothetical protein	Hypothetical	62.555	55.113	66.330	162.958	103.042	314.890
AMK58_22450	no KO assigned | (GenBank) methanol dehydrogenase	dehydrogenase	237.908	212.706	267.083	103.992	317.045	185.626
AMK58_22455	no KO assigned | (GenBank) hypothetical protein	Hypothetical	2.484	7.419	4.132	93.985	28.614	269.447
AMK58_22460	no KO assigned | (GenBank) hypothetical protein	Hypothetical	58.050	36.333	56.771	132.563	101.293	226.077
AMK58_22465	K02334 DNA polymerase bacteriophage-type [EC:2.7.7.7] | (GenBank) DNA polymerase	DNA Repair and Replication	2.707	1.011	4.103	210.921	25.012	97.755
AMK58_22470	no KO assigned | (GenBank) hypothetical protein	Hypothetical	5.481	4.763	6.727	206.236	67.543	175.196
AMK58_22475	no KO assigned | (GenBank) 6-aminohexanoate hydrolase	hydrolase	20.582	25.417	25.582	255.832	380.751	162.807
AMK58_22480	K02004 putative ABC transport system permease protein | (GenBank) hypothetical protein	Membrane Transport	9.053	10.818	13.773	199.014	100.155	67.070
AMK58_22485	K07107 acyl-CoA thioester hydrolase [EC:3.1.2.-] | (GenBank) thioesterase	hydrolase	11.985	13.152	12.326	252.617	65.435	119.754
AMK58_22490	no KO assigned | (GenBank) LysR family transcriptional regulator	Transcription	26.246	39.398	44.016	320.132	97.548	166.448
AMK58_22495	no KO assigned | (GenBank) hypothetical protein	Hypothetical	4.227	3.608	7.464	299.026	36.201	178.530
AMK58_22500	no KO assigned | (GenBank) hypothetical protein	Hypothetical	14.042	16.157	25.219	273.654	48.638	161.942
AMK58_22505	K07068 uncharacterized protein | (GenBank) hypothetical protein	Hypothetical	8.734	7.198	16.609	269.099	33.515	189.909
AMK58_22510	no KO assigned | (GenBank) hypothetical protein	Hypothetical	13.184	12.860	22.001	139.119	35.082	159.922
AMK58_22515	K00059 3-oxoacyl-[acyl-carrier protein] reductase [EC:1.1.1.100] | (GenBank) 3-oxoacyl-ACP reductase	Metabolism: Lipid	7.852	15.011	10.900	143.464	17.652	95.815
AMK58_22520	K02012 iron(III) transport system substrate-binding protein | (GenBank) ABC transporter substrate-bi	Transport: Metal	68.449	66.445	81.979	173.858	59.154	175.847
AMK58_22525	K02011 iron(III) transport system permease protein | (GenBank) ABC transporter substrate-binding pro	Transport: Metal	16.130	15.322	21.561	195.816	22.303	138.741
AMK58_22530	K02010 iron(III) transport system ATP-binding protein [EC:3.6.3.30] | (GenBank) sulfate ABC transpor	Transport: Metal	11.323	12.056	17.481	168.793	19.028	119.442
AMK58_22535	no KO assigned | (GenBank) enoyl-CoA hydratase	hydratase	5.045	4.432	8.182	197.366	16.178	104.290
AMK58_22540	no KO assigned | (GenBank) hypothetical protein	Hypothetical	9.879	13.268	10.704	156.749	18.852	83.439
AMK58_22545	no KO assigned | (GenBank) hypothetical protein	Hypothetical	8.957	10.823	9.378	139.132	22.513	81.473
AMK58_22550	no KO assigned | (GenBank) hypothetical protein	Hypothetical	24.872	25.233	25.210	131.069	35.078	159.701
AMK58_22555	no KO assigned | (GenBank) hypothetical protein	Hypothetical	45.674	38.792	35.195	132.597	32.342	115.664
AMK58_22560	K04063 osmotically inducible protein OsmC | (GenBank) peroxiredoxin	Stress Response	65.937	49.243	91.068	266.883	436.393	319.679
AMK58_22565	no KO assigned | (GenBank) acyl carrier protein	No COG	16.849	7.023	42.099	370.561	128.606	196.199
AMK58_22570	no KO assigned | (GenBank) acyl-CoA dehydrogenase	dehydrogenase	7.870	8.439	19.666	182.189	107.854	124.245
AMK58_22575	no KO assigned | (GenBank) hypothetical protein	Hypothetical	8.264	7.406	16.895	212.373	156.066	239.866
AMK58_22580	no KO assigned | (GenBank) serine--tRNA ligase	tRNA synthesis/modification	4.156	3.204	11.219	213.913	74.603	165.607
AMK58_22585	K01192 beta-mannosidase [EC:3.2.1.25] | (GenBank) beta-mannosidase	Metabolism	1.339	2.074	4.007	177.860	36.280	63.215
AMK58_22590	no KO assigned | (GenBank) hypothetical protein	Hypothetical	5.078	7.186	7.814	189.754	23.508	57.312
AMK58_22595	no KO assigned | (GenBank) hypothetical protein	Hypothetical	217.653	163.202	151.649	150.146	104.001	195.777
AMK58_22600	no KO assigned | (GenBank) hypothetical protein	Hypothetical	408.109	274.920	246.376	81.851	249.716	306.731
AMK58_22605	no KO assigned | (GenBank) hypothetical protein	Hypothetical	306.035	256.256	227.141	211.399	135.890	217.827
AMK58_22610	no KO assigned | (GenBank) acetyltransferase	Acetyltransferase	133.820	157.362	114.109	85.076	100.757	223.751
AMK58_22615	no KO assigned | (GenBank) hypothetical protein	Hypothetical	137.457	141.304	170.476	105.155	19.574	147.168
AMK58_22620	no KO assigned | (GenBank) hypothetical protein	Hypothetical	155.939	171.558	193.668	172.437	19.778	195.729
AMK58_22625	no KO assigned | (GenBank) hypothetical protein	Hypothetical	54.887	62.155	65.490	137.918	22.783	148.861
AMK58_22630	no KO assigned | (GenBank) hypothetical protein	Hypothetical	45.426	54.603	62.033	178.307	23.394	117.220
AMK58_22635	K16922 putative peptide zinc metalloprotease protein | (GenBank) peptidase	peptidase	46.022	49.603	55.416	174.118	35.105	176.562
AMK58_22640	no KO assigned | (GenBank) hypothetical protein	Hypothetical	50.690	55.939	69.992	115.909	75.849	245.796
AMK58_22645	no KO assigned | (GenBank) hypothetical protein	Hypothetical	15.212	19.289	24.972	156.114	63.231	183.777
AMK58_22650	no KO assigned | (GenBank) hypothetical protein	Hypothetical	29.102	39.366	41.244	176.529	99.061	110.742
AMK58_22655	no KO assigned | (GenBank) hypothetical protein	Hypothetical	31.950	42.926	37.937	217.477	249.332	109.435
AMK58_22660	K06966 uncharacterized protein | (GenBank) 3-isopropylmalate dehydrogenase	No COG	138.451	157.397	122.447	118.824	237.533	131.501
AMK58_22665	no KO assigned | (GenBank) histidine kinase	Signal Transduction	16.934	19.992	20.536	237.981	86.284	126.374
AMK58_22670	no KO assigned | (GenBank) hypothetical protein	Hypothetical	65.690	78.235	84.910	221.136	149.123	127.796
AMK58_22675	K06886 hemoglobin | (GenBank) preprotein translocase	No COG	122.700	114.895	138.555	96.876	237.478	193.144
AMK58_22680	K00782 L-lactate dehydrogenase complex protein LldG | (GenBank) lactate utilization protein B/C	No COG	26.049	24.112	38.199	154.568	53.819	249.035
AMK58_22685	K18929 L-lactate dehydrogenase complex protein LldF | (GenBank) (Fe-S)-binding protein	No COG	32.457	27.919	33.445	142.837	29.511	164.426
AMK58_22690	K18928 L-lactate dehydrogenase complex protein LldE | (GenBank) oxidoreductase	oxidoreductase	58.849	62.441	64.821	101.662	47.714	166.094
AMK58_22695	K18930 D-lactate dehydrogenase | (GenBank) 4Fe-4S ferredoxin	ferredoxin	7.162	5.789	8.211	195.763	26.423	180.671
AMK58_22700	no KO assigned | (GenBank) L-lactate permease	Membrane transport	3.082	2.998	5.316	143.557	29.330	259.570
AMK58_22705	no KO assigned | (GenBank) GntR family transcriptional regulator	Transcription	29.480	46.152	30.728	271.788	86.121	247.198
AMK58_22710	K02351 putative membrane protein | (GenBank) hypothetical protein	Membrane	17.770	16.195	18.471	207.574	101.224	221.188
AMK58_22715	no KO assigned | (GenBank) SAM-dependent methyltransferase	methyltransferase	35.670	13.395	33.722	157.817	112.265	251.189
AMK58_22720	K03695 ATP-dependent Clp protease ATP-binding subunit ClpB | (GenBank) ATP-dependent chaperone (	Chaperone	88.335	88.862	109.945	228.397	1485.659	215.667
AMK58_22725	no KO assigned | (GenBank) transcriptional regulator	Transcription	54.340	66.308	64.549	241.927	113.735	171.479
AMK58_22730	K00883 2-dehydro-3-deoxygalactonokinase [EC:2.7.1.58] | (GenBank) 2-keto-3-deoxy-galactonokinase	Metabolism: Carbohydrate	23.654	16.026	32.461	173.619	67.567	85.022
AMK58_22735	K01631 2-dehydro-3-deoxyphosphogalactonate aldolase [EC:4.1.2.21] | (GenBank) 2-dehydro-3-deoxy-6-[illegible]	Metabolism: Carbohydrate	24.736	27.779	35.897	204.539	74.841	82.607
AMK58_22740	no KO assigned | (GenBank) ABC transporter substrate-binding protein	Membrane Transport	676.189	615.526	750.180	141.703	151.027	222.796
AMK58_22745	no KO assigned | (GenBank) hypothetical protein	Hypothetical	165.445	133.955	201.963	156.495	96.830	213.816
AMK58_22750	K07793 putative tricarboxylic transport membrane protein | (GenBank) C4-dicarboxylate ABC transporte	Membrane	107.047	91.297	121.676	156.412	79.366	195.327
AMK58_22755	no KO assigned | (GenBank) gluconolactonase	enzyme	86.152	91.739	106.204	188.034	118.375	224.161
AMK58_22760	K00059 3-oxoacyl-[acyl-carrier protein] reductase [EC:1.1.1.100] | (GenBank) 3-ketoacyl-ACP synthase	Metabolism: Lipid	106.427	126.610	127.564	200.384	190.566	163.709
AMK58_22765	no KO assigned | (GenBank) hypothetical protein	Hypothetical	25.708	39.636	50.463	228.097	72.397	150.462
AMK58_22770	no KO assigned | (GenBank) hypothetical protein	Hypothetical	16.537	27.052	20.590	96.652	65.170	293.909
AMK58_22775	K07814 putative two-component system response regulator | (GenBank) hypothetical protein	Hypothetical	18.102	14.183	22.854	92.223	77.053	105.390

AMK58_22780	no KO assigned | (GenBank) hypothetical protein	Hypothetical	15.060	21.898	19.546	167.469	58.620	144.890
AMK58_22785	no KO assigned | (GenBank) hypothetical protein	Hypothetical	21.375	25.898	30.841	258.696	49.733	177.691
AMK58_22790	K02030 polar amino acid transport system substrate-binding protein | (GenBank) hypothetical protein	Transport: Amino Acid	66.936	89.194	71.286	101.531	58.273	220.128
AMK58_22795	no KO assigned | (GenBank) MarR family transcriptional regulator	Transcription	20.839	17.335	24.077	190.100	67.602	232.122
AMK58_22800	no KO assigned | (GenBank) hypothetical protein	Hypothetical	46.382	34.188	55.269	194.058	127.630	168.405
AMK58_22805	no KO assigned | (GenBank) hypothetical protein	Hypothetical	89.635	70.604	103.141	267.622	86.092	105.653
AMK58_22810	no KO assigned | (GenBank) hypothetical protein	Hypothetical	56.585	68.329	82.439	200.286	42.739	147.861
AMK58_22815	no KO assigned | (GenBank) hypothetical protein	Hypothetical	966.013	1057.832	1004.645	146.223	82.262	131.033
AMK58_22820	K06140 regulator of nucleoside diphosphate kinase | (GenBank) hypothetical protein	Transcription	1455.670	2114.849	2062.181	153.932	67.873	266.819
AMK58_22825	K00262 glutamate dehydrogenase (NADP+) [EC:1.4.1.4] | (GenBank) glutamate dehydrogenase	Metabolism: Amino Acid	330.169	250.304	251.891	178.791	71.827	184.087
AMK58_22830	no KO assigned | (GenBank) hypothetical protein	Hypothetical	49.142	49.762	51.188	215.842	42.556	207.197
AMK58_22835	no KO assigned | (GenBank) LuxR family transcriptional regulator	Transcription	196.964	252.655	219.038	144.596	50.140	130.713
AMK58_22840	K03832 periplasmic protein TonB | (GenBank) hypothetical protein	Transport	289.234	289.855	284.804	168.321	34.747	91.037
AMK58_22845	K03559 biopolymer transport protein ExbD | (GenBank) biopolymer transporter	Transport: Biopolymer	442.510	497.628	416.388	213.947	44.721	78.562
AMK58_22850	K03561 biopolymer transport protein ExbB | (GenBank) hypothetical protein	Transport: Biopolymer	716.716	755.143	743.523	142.890	88.978	114.315
AMK58_22855	no KO assigned | (GenBank) hypothetical protein	Hypothetical	85.819	182.917	132.762	287.088	110.543	144.961
AMK58_22860	no KO assigned | (GenBank) hypothetical protein	Hypothetical	108.555	117.919	121.141	162.975	151.822	157.341
AMK58_22865	no KO assigned | (GenBank) hypothetical protein	Hypothetical	6.105	12.157	9.351	149.465	71.287	172.021
AMK58_22870	no KO assigned | (GenBank) hypothetical protein	Hypothetical	4.817	9.156	7.911	156.809	54.306	148.407
AMK58_22875	K03642 rare lipoprotein A | (GenBank) hypothetical protein	No COG	18.978	31.575	26.496	184.068	92.619	261.294
AMK58_22880	no KO assigned | (GenBank) hypothetical protein	Hypothetical	19.887	43.681	32.812	222.516	60.157	181.421
AMK58_22885	K02478 two-component system, LytT family, sensor kinase [EC:2.7.13.3] | (GenBank) histidine kinase	Signal Transduction	10.550	12.690	8.011	142.837	42.738	156.324
AMK58_22890	K02477 two-component system, LytT family, response regulator | (GenBank) two-component system respo	Signal Transduction	5.381	8.037	9.273	151.373	19.091	168.716
AMK58_22895	K06200 carbon starvation protein | (GenBank) carbon starvation protein CstA	Starvation	3.942	3.194	6.353	133.365	64.366	214.812
AMK58_22900	no KO assigned | (GenBank) MFS transporter	Transport: MFS	8.303	12.114	10.649	142.915	85.628	127.400
AMK58_22905	no KO assigned | (GenBank) peptidase M42	peptidase	9.490	8.723	18.443	155.900	289.535	178.316
AMK58_22910	no KO assigned | (GenBank) GNAT family acetyltransferase	Acetyltransferase	5.702	7.079	14.011	211.784	158.817	171.597
AMK58_22915	K01953 asparagine synthase (glutamine-hydrolysing) [EC:6.3.5.4] | (GenBank) asparagine synthase	Metabolism: Amino Acid	9.885	12.626	22.298	225.106	233.412	211.517
AMK58_22920	K00375 GntR family transcriptional regulator / MocR family aminotransferase | (GenBank) transcriptio	Transcription	3.692	6.517	5.425	227.712	205.790	103.556
AMK58_22925	no KO assigned | (GenBank) flavin-nucleotide-binding protein	No COG	13.508	9.964	13.002	218.705	744.389	164.693
AMK58_22930	no KO assigned | (GenBank) hypothetical protein	Hypothetical	88.219	119.014	93.473	240.853	2868.135	251.887
AMK58_22935	no KO assigned | (GenBank) hypothetical protein	Hypothetical	257.473	312.036	311.293	338.827	1392.469	246.353
AMK58_22940	no KO assigned | (GenBank) LysR family transcriptional regulator	Transcription	5.896	10.960	5.980	155.867	26.217	111.055
AMK58_22945	K00822 beta-alanine--pyruvate transaminase [EC:2.6.1.18] | (GenBank) omega amino acid--pyruvate amin	Metabolism	107.296	115.248	156.028	276.026	64.349	203.602
AMK58_22950	K00140 malonate-semialdehyde dehydrogenase (acetylating) / methylmalonate-semialdehyde dehydrogenas	Metabolism	113.245	140.331	164.543	132.127	56.070	158.151
AMK58_22955	no KO assigned | (GenBank) GntR family transcriptional regulator	Transcription	59.022	60.994	84.481	180.776	42.316	272.908
AMK58_22960	no KO assigned | (GenBank) hypothetical protein	Hypothetical	30.665	43.170	43.400	219.789	45.761	175.023
AMK58_22965	no KO assigned | (GenBank) hypothetical protein	Hypothetical	12.812	22.964	24.364	127.003	20.882	163.355
AMK58_22970	no KO assigned | (GenBank) hypothetical protein	Hypothetical	47.228	56.432	71.357	93.401	27.026	178.039
AMK58_22975	no KO assigned | (GenBank) tricarboxylate transporter	transport	77.941	71.530	108.334	151.140	73.884	191.822
AMK58_22980	no KO assigned | (GenBank) hypothetical protein	Hypothetical	299.702	275.360	356.029	142.033	58.786	267.720
AMK58_22985	no KO assigned | (GenBank) tricarboxylate transporter	transport	530.370	493.366	629.789	144.553	75.096	280.041
AMK58_22990	no KO assigned | (GenBank) hypothetical protein	Hypothetical	11.673	11.482	17.055	145.405	40.564	218.203
AMK58_22995	no KO assigned | (GenBank) pseudogene	Pseudogene	6.684	10.751	13.198	50.963	13.291	173.053
AMK58_23000	no KO assigned | (GenBank) hypothetical protein	Hypothetical	1.727	7.506	5.226	198.998	6.531	114.454
AMK58_23005	no KO assigned | (GenBank) hypothetical protein	Hypothetical	13.758	11.569	12.306	139.114	16.840	290.124
AMK58_23010	no KO assigned | (GenBank) hypothetical protein	Hypothetical	1.268	3.156	6.429	278.801	13.063	179.181
AMK58_23015	no KO assigned | (GenBank) glycosyl transferase	Glycosyl Transferase	3.225	4.954	4.205	177.861	15.225	194.977
AMK58_23020	no KO assigned | (GenBank) hypothetical protein	Hypothetical	2.407	2.675	3.938	186.105	8.746	99.501
AMK58_23025	no KO assigned | (GenBank) glycosyl transferase	Glycosyl Transferase	1.395	1.667	3.802	192.082	10.975	207.380
AMK58_23030	no KO assigned | (GenBank) methyltransferase	methyltransferase	1.170	0.874	3.059	190.588	10.848	120.948
AMK58_23035	no KO assigned | (GenBank) glycosyl transferase family 2	Glycosyl Transferase	0.669	1.230	2.447	195.147	8.506	82.532
AMK58_23040	no KO assigned | (GenBank) hemolysin expression modulating protein	No COG	8.985	3.520	6.302	113.254	24.338	219.213
AMK58_23045	no KO assigned | (GenBank) hypothetical protein	Hypothetical	5.605	3.349	9.060	93.858	97.659	278.075
AMK58_23050	no KO assigned | (GenBank) type I secretion membrane fusion protein	Secretion System	4.053	4.212	5.781	133.030	24.465	158.518
AMK58_23055	no KO assigned | (GenBank) ABC transporter ATP-binding protein	Membrane Transport	4.148	7.141	9.359	196.375	57.456	143.499
AMK58_23060	K01784 UDP-glucose 4-epimerase [EC:5.1.3.2] | (GenBank) UDP-glucose 4-epimerase	Metabolism: Carbohydrate	17.805	15.602	18.735	231.194	49.501	143.529
AMK58_23065	no KO assigned | (GenBank) transcriptional regulator	Transcription	7.891	6.735	9.432	191.784	42.830	148.438
AMK58_23070	no KO assigned | (GenBank) hypothetical protein	Hypothetical	11.091	13.498	18.357	168.101	60.370	110.841
AMK58_23075	no KO assigned | (GenBank) TetR family transcriptional regulator	Transcription	222.931	241.119	222.320	173.628	324.031	240.380
AMK58_23080	K18661 malonyl-CoA/methylmalonyl-CoA synthetase [EC:6.2.1.-] | (GenBank) malonyl-CoA synthase	Metabolism: Amino Acid	335.651	298.637	312.183	174.360	72.116	207.470
AMK58_23085	no KO assigned | (GenBank) AsnC family transcriptional regulator	Transcription	111.333	134.070	122.066	233.492	69.611	260.833
AMK58_23090	no KO assigned | (GenBank) acyl-CoA dehydrogenase	dehydrogenase	495.345	563.456	513.592	260.351	103.622	184.063
AMK58_23095	K00020 3-hydroxyisobutyrate dehydrogenase [EC:1.1.1.31] | (GenBank) 3-hydroxyisobutyrate dehydroger	Metabolism: Amino Acid	299.086	408.359	352.851	88.465	73.622	97.023
AMK58_23100	K07214 enterochelin esterase and related enzymes | (GenBank) hypothetical protein	Enzyme	315.358	331.700	310.828	191.296	102.171	210.280
AMK58_23105	no KO assigned | (GenBank) hypothetical protein	Hypothetical	139.941	165.505	123.776	203.690	29.247	97.085
AMK58_23110	no KO assigned | (GenBank) protein mbtH	Biosynthesis: secondary metabolites	119.320	146.768	105.109	364.331	40.762	45.559

AMK58_23115	K00216 2,3-dihydro-2,3-dihydroxybenzoate dehydrogenase [EC:1.3.1.28] | (GenBank) 2,3-dihydro-2,3-dil	Metabolism: terpenoids and polyketide	83.239	123.385	99.830	180.136	28.176	91.804
AMK58_23120	K01252 bifunctional isochorismate lyase / aryl carrier protein [EC:3.3.2.1 6.3.2.14] | (GenBank) iso	Metabolism: terpenoids and polyketide	376.769	414.526	347.591	152.373	53.132	130.212
AMK58_23125	K02363 2,3-dihydroxybenzoate-AMP ligase [EC:6.3.2.14 2.7.7.58] | (GenBank) entE; 2,3-dihydroxybenzo	Biosynthesis: secondary metabolites	171.855	186.801	133.884	198.722	49.103	88.692
AMK58_23130	K02361 isochorismate synthase [EC:5.4.4.2] | (GenBank) hypothetical protein	Metabolism: Co-Factors and Vitamens	289.535	360.478	272.715	201.186	48.186	124.279
AMK58_23135	no KO assigned | (GenBank) hypothetical protein	Hypothetical	107.930	98.285	82.666	180.052	12.184	161.098
AMK58_23140	no KO assigned | (GenBank) hypothetical protein	Hypothetical	102.628	90.837	85.356	168.775	8.671	48.723
AMK58_23145	K08225 MFS transporter, ENTS family, enterobactin (siderophore) exporter | (GenBank) POT family tran	Transport: MFS	245.409	222.791	198.063	213.936	7.651	89.780
AMK58_23150	K16089 outer membrane receptor for ferrienterochelin and colicins | (GenBank) hypothetical protein	Transport	1792.385	1371.855	1184.893	138.992	45.647	188.692
AMK58_23155	no KO assigned | (GenBank) peptidase	peptidase	253.989	245.567	198.662	223.529	20.673	126.217
AMK58_23160	no KO assigned | (GenBank) hypothetical protein	Hypothetical	310.682	263.866	254.492	176.466	20.542	154.989
AMK58_23165	no KO assigned | (GenBank) hypothetical protein	Hypothetical	216.926	193.397	193.064	92.919	13.231	204.658
AMK58_23170	K03413 two-component system, chemotaxis family, response regulator CheY | (GenBank) hypothetical pro	Chemotaxis	75.528	74.058	70.716	96.539	100.774	271.586
AMK58_23175	no KO assigned | (GenBank) hypothetical protein	Hypothetical	33.610	40.716	38.310	165.125	23.803	143.867
AMK58_23180	K12368 dipeptide transport system substrate-binding protein | (GenBank) peptide ABC transporter subs	chemotaxis	184.826	173.695	150.507	76.888	28.731	225.510
AMK58_23185	no KO assigned | (GenBank) acetyl-CoA synthetase	enzyme	27.952	25.725	38.165	186.724	26.989	129.865
AMK58_23190	K01995 branched-chain amino acid transport system ATP-binding protein | (GenBank) ABC transporter A'	Transport: Amino Acid	869.878	771.759	900.125	117.369	119.735	226.487
AMK58_23195	K01897 long-chain acyl-CoA synthetase [EC:6.2.1.3] | (GenBank) AMP-dependent synthetase	Metabolism: Lipid	525.785	453.782	565.942	139.016	80.726	259.696
AMK58_23200	K01997 branched-chain amino acid transport system permease protein | (GenBank) ABC transporter perme	Transport: Amino Acid	604.283	500.158	679.176	62.829	106.055	210.506
AMK58_23205	K01998 branched-chain amino acid transport system permease protein | (GenBank) ABC transporter perme	Transport: Amino Acid	567.947	447.214	598.104	71.428	69.060	192.761
AMK58_23210	K01999 branched-chain amino acid transport system substrate-binding protein | (GenBank) branched-cha	Transport: Amino Acid	1080.904	919.405	1307.819	194.017	77.162	176.274
AMK58_23215	K01996 branched-chain amino acid transport system ATP-binding protein | (GenBank) ABC transporter A'	Transport: Amino Acid	284.898	289.006	353.780	161.621	56.745	178.510
AMK58_23220	K01912 phenylacetate-CoA ligase [EC:6.2.1.30] | (GenBank) AMP-dependent synthetase	Metabolism: Amino Acid	125.898	127.616	167.624	222.577	56.982	222.805
AMK58_23225	no KO assigned | (GenBank) aldehyde dehydrogenase	dehydrogenase	289.720	294.862	319.929	123.431	697.227	192.827
AMK58_23230	K00253 isovaleryl-CoA dehydrogenase [EC:1.3.8.4] | (GenBank) isovaleryl-CoA dehydrogenase	Metabolism: Amino Acid	413.904	407.620	366.948	137.989	535.323	201.473
AMK58_23235	K00626 acetyl-CoA C-acetyltransferase [EC:2.3.1.9] | (GenBank) acetyl-CoA acetyltransferase	Metabolism	478.153	395.948	469.339	212.269	391.815	174.388
AMK58_23240	no KO assigned | (GenBank) acyl-CoA dehydrogenase	dehydrogenase	243.110	209.862	245.364	250.859	192.739	130.981
AMK58_23245	K01969 3-methylcrotonyl-CoA carboxylase beta subunit [EC:6.4.1.4] | (GenBank) methylcrotonoyl-CoA c	Metabolism: Amino Acid	423.611	321.191	377.358	130.919	253.567	212.111
AMK58_23250	K13766 methylglutaconyl-CoA hydratase [EC:4.2.1.18] | (GenBank) enoyl-CoA hydratase	Metabolism: Amino Acid	209.987	204.165	219.505	115.995	132.443	125.604
AMK58_23255	K01968 3-methylcrotonyl-CoA carboxylase alpha subunit [EC:6.4.1.4] | (GenBank) 3-methylcrotonyl-CoA	Metabolism: Amino Acid	98.539	89.517	111.442	129.571	50.118	83.698
AMK58_23260	K01640 hydroxymethylglutaryl-CoA lyase [EC:4.1.3.4] | (GenBank) hydroxymethylglutaryl-CoA lyase	Metabolism	205.154	163.157	242.389	213.184	111.748	167.407
AMK58_23265	no KO assigned | (GenBank) hypothetical protein	Hypothetical	172.839	168.608	187.277	207.042	85.305	133.964
AMK58_23270	K00311 electron-transferring-flavoprotein dehydrogenase [EC:1.5.5.1] | (GenBank) electron transfer f	Energy	213.972	170.098	207.144	114.022	88.749	178.883
AMK58_23275	no KO assigned | (GenBank) 3-hydroxy-2-methylbutyryl-CoA dehydrogenase	dehydrogenase	355.280	323.706	381.313	215.609	178.064	190.800
AMK58_23280	no KO assigned | (GenBank) hypothetical protein	Hypothetical	122.476	112.187	148.162	247.784	402.924	324.594
AMK58_23285	K07017 uncharacterized protein | (GenBank) hypothetical protein	Hypothetical	69.528	59.683	62.689	96.348	55.096	235.408
AMK58_23290	no KO assigned | (GenBank) methyltransferase	methyltransferase	79.986	106.921	88.379	42.320	332.741	220.137
AMK58_23295	no KO assigned | (GenBank) hypothetical protein	Hypothetical	196.936	181.292	187.729	114.899	130.408	295.822
AMK58_23300	no KO assigned | (GenBank) transcriptional regulator	Transcription	1828.022	1910.401	1519.668	72.429	232.929	289.070
AMK58_23305	K05350 beta-glucosidase [EC:3.2.1.21] | (GenBank) beta-glucosidase	Metabolism: Carbohydrates	59.716	67.099	59.469	137.112	92.987	295.830
AMK58_23310	K05350 beta-glucosidase [EC:3.2.1.21] | (GenBank) beta-glucosidase	Metabolism	181.476	176.098	176.602	218.192	131.608	189.387
AMK58_23315	no KO assigned | (GenBank) hypothetical protein	Hypothetical	22.249	25.074	29.627	168.501	68.240	150.173
AMK58_23320	K02471 putative ATP-binding cassette transporter | (GenBank) ABC transporter	Membrane Transport	31.019	35.470	31.903	237.873	26.694	207.838
AMK58_23325	no KO assigned | (GenBank) hypothetical protein	Hypothetical	4.702	7.491	8.643	187.590	25.985	121.644
AMK58_23330	no KO assigned | (GenBank) hypothetical protein	Hypothetical	6.565	9.208	9.661	182.220	28.489	188.916
AMK58_23335	no KO assigned | (GenBank) hypothetical protein	Hypothetical	2.484	11.129	4.723	86.499	46.062	301.689
AMK58_23340	K03446 MFS transporter, DHA2 family, multidrug resistance protein | (GenBank) MFS transporter	Transport: MFS	2.741	2.730	4.634	171.570	49.473	215.572
AMK58_23345	K03543 membrane fusion protein, multidrug efflux system | (GenBank) multidrug transporter	Transport	2.176	5.199	5.896	152.714	26.901	126.795
AMK58_23350	no KO assigned | (GenBank) alkylhydroperoxidase	Defense	7.042	4.855	8.241	150.212	31.664	230.316
AMK58_23355	no KO assigned | (GenBank) NmrA family transcriptional regulator	Transcription	6.592	15.370	10.243	165.847	32.352	223.645
AMK58_23360	no KO assigned | (GenBank) CadC-family transcriptional regulator	Transcription	32.317	45.350	39.445	148.897	54.150	255.133
AMK58_23365	no KO assigned | (GenBank) transposase	Genetic Information Processing	16.525	9.493	13.597	186.024	35.720	218.057
AMK58_23370	K14230 tRNA Met | (GenBank) tRNA-Met	tRNA synthesis/modification	69.186	122.125	56.812	98.997	259.814	298.900
AMK58_23375	K01985 5S ribosomal RNA | (GenBank) 5S ribosomal RNA	Ribosome	0.000	0.000	0.000	0.000	0.000	0.000
AMK58_23380	K01980 23S ribosomal RNA | (GenBank) 23S ribosomal RNA	Ribosome	0.220	0.263	0.084	0.412	0.099	1.590
AMK58_23385	K14218 tRNA Ala | (GenBank) tRNA-Ala	tRNA synthesis/modification	36.641	19.036	43.927	21.340	23.279	193.517
AMK58_23390	K14227 tRNA Ile | (GenBank) tRNA-Ile	tRNA synthesis/modification	17.296	9.394	10.465	10.532	15.907	94.773
AMK58_23395	K01977 16S ribosomal RNA | (GenBank) 16S ribosomal RNA	Ribosome	0.000	0.000	0.000	0.000	0.000	0.000
AMK58_23400	no KO assigned | (GenBank) peptidase M23	peptidase	367.999	439.180	368.226	157.962	64.705	191.460
AMK58_23405	no KO assigned | (GenBank) hypothetical protein	Hypothetical	44.748	42.250	51.009	240.584	28.507	173.775
AMK58_23410	K02556 chemotaxis protein MotA | (GenBank) flagellar motor stator protein MotA	Chemotaxis	194.620	160.466	151.353	139.582	29.874	307.601
AMK58_23415	K01596 phosphoenolpyruvate carboxykinase (GTP) [EC:4.1.1.32] | (GenBank) phosphoenolpyruvate carb	Metabolism: Carbohydrate	671.288	566.708	613.463	171.078	130.721	186.378
AMK58_23420	K07226 heme iron utilization protein | (GenBank) heme iron utilization protein	No COG	115.419	179.012	157.998	213.995	121.831	87.746
AMK58_23425	K14981 two-component system, OmpR family, response regulator ChvI | (GenBank) two-component syste	Signal transduction	118.291	138.227	124.381	185.022	78.801	118.722
AMK58_23430	K14980 two-component system, OmpR family, sensor histidine kinase ChvG [EC:2.7.13.3] | (GenBank) h	Signal Transduction	23.485	31.271	27.699	228.149	56.357	144.456
AMK58_23435	no KO assigned | (GenBank) hypothetical protein	Hypothetical	122.804	170.505	120.053	156.394	95.913	286.020
AMK58_23440	no KO assigned | (GenBank) aldolase	enzyme	175.984	232.917	210.921	125.409	342.547	249.234
AMK58_23445	K06958 RNase adapter protein RapZ | (GenBank) nucleotide-binding protein	Genetic Information Processing	210.440	227.027	247.431	158.851	287.357	219.679

AMK58_23450	K02793 PTS system, mannose-specific IIA component [EC:2.7.1.191] | (GenBank) PTS fructose transport	Metabolism	514.116	642.383	551.540	93.600	268.462	220.909
AMK58_23455	K11189 phosphocarrier protein | (GenBank) serine kinase	Signal Transduction	268.749	309.618	255.093	47.304	169.085	124.602
AMK58_23460	K08483 phosphotransferase system, enzyme I, PtsI [EC:2.7.3.9] | (GenBank) phosphoenolpyruvate-protei	Membrane transport	72.659	82.005	76.551	200.240	96.332	140.701
AMK58_23465	no KO assigned | (GenBank) hypothetical protein	Hypothetical	23.601	21.151	23.001	260.193	72.424	245.877
AMK58_23470	no KO assigned | (GenBank) hypothetical protein	Hypothetical	3.908	4.061	7.432	198.037	29.320	133.857
AMK58_23475	K02035 peptide/nickel transport system substrate-binding protein | (GenBank) hypothetical protein	Quorum sensing	58.322	59.678	63.342	144.381	63.293	268.387
AMK58_23480	K02033 peptide/nickel transport system permease protein | (GenBank) ABC transporter	Quorum sensing	21.128	24.187	28.268	152.695	50.484	165.061
AMK58_23485	K02034 peptide/nickel transport system permease protein | (GenBank) peptide ABC transporter permease	Quorum sensing	6.869	9.234	12.083	153.674	24.516	178.676
AMK58_23490	K02031 peptide/nickel transport system ATP-binding protein K02032 peptide/nickel transport system AT	Quorum sensing	9.235	13.963	14.959	173.945	21.401	104.447
AMK58_23495	no KO assigned | (GenBank) enamine deaminase RidA	enzyme	16.887	25.651	16.329	165.253	24.452	303.367
AMK58_23500	K01480 agmatinase [EC:3.5.3.11] | (GenBank) arginase	Metabolism: Amino Acid	20.557	16.577	18.827	133.803	29.912	190.625
AMK58_23505	K01438 acetylornithine deacetylase [EC:3.5.1.16] | (GenBank) acetylornithine deacetylase	Metabolism: Amino Acid	10.537	10.702	15.830	169.667	36.363	137.870
AMK58_23510	K15634 probable phosphoglycerate mutase [EC:5.4.2.12] | (GenBank) hypothetical protein	Metabolism	6.101	5.607	10.486	139.555	15.825	69.189
AMK58_23515	K01091 phosphoglycolate phosphatase [EC:3.1.3.18] | (GenBank) phosphoglycolate phosphatase	Metabolism: Carbohydrate	27.020	30.651	33.170	210.750	39.982	65.173
AMK58_23520	K02010 iron(III) transport system ATP-binding protein [EC:3.6.3.30] | (GenBank) ABC transporter ATP-	Transport: Metal	16.409	16.560	21.295	188.920	23.056	109.802
AMK58_23525	K02011 iron(III) transport system permease protein | (GenBank) ABC transporter permease	Transport: Metal	28.343	19.847	27.889	194.472	20.895	218.182
AMK58_23530	no KO assigned | (GenBank) ABC transporter substrate-binding protein	Membrane Transport	99.952	91.292	136.485	109.328	34.857	185.032
AMK58_23535	no KO assigned | (GenBank) aspartate-alanine antiporter	Transport: Amino Acid	2.796	4.283	5.248	216.536	62.044	185.521
AMK58_23540	K09758 aspartate 4-decarboxylase [EC:4.1.1.12] | (GenBank) aspartate 4-decarboxylase	Metabolism: Amino Acid	3.932	5.319	7.830	161.291	46.615	238.160
AMK58_23545	K10126 two-component system, NtrC family, C4-dicarboxylate transport response regulator DctD | (GenB	Signal transduction	10.810	17.223	17.559	135.879	28.049	72.675
AMK58_23550	K08475 two-component system, NtrC family, phosphoglycerate transport system sensor histidine kinase	Signal Transduction	14.288	16.174	19.115	182.468	30.600	70.662
AMK58_23555	no KO assigned | (GenBank) hypothetical protein	Hypothetical	49.404	67.555	61.198	157.059	101.679	162.597
AMK58_23560	no KO assigned | (GenBank) hypothetical protein	Hypothetical	136.046	180.839	164.014	62.782	92.558	264.377
AMK58_23565	K05350 beta-glucosidase [EC:3.2.1.21] | (GenBank) beta-glucosidase	Metabolism: Carbohydrate	200.522	197.771	178.327	164.737	136.501	211.588
AMK58_23570	K07406 alpha-galactosidase [EC:3.2.1.22] | (GenBank) alpha-glucosidase/alpha-galactosidase	Metabolism	439.737	467.167	438.980	136.406	262.420	267.680
AMK58_23575	no KO assigned | (GenBank) hypothetical protein	Hypothetical	808.345	1108.299	902.326	298.131	535.884	215.129
AMK58_23580	no KO assigned | (GenBank) hypothetical protein	Hypothetical	128.729	151.738	110.819	172.439	130.618	135.175
AMK58_23585	K01438 acetylornithine deacetylase [EC:3.5.1.16] | (GenBank) acetylornithine deacetylase	Biosynthesis: Amino Acid	137.454	184.197	185.351	203.055	142.110	277.547
AMK58_23590	K03410 chemotaxis protein CheC | (GenBank) chemotaxis protein CheX	Chemotaxis	361.904	392.099	371.716	77.556	175.734	200.931
AMK58_23595	no KO assigned | (GenBank) histidine kinase	Signal Transduction	196.632	209.516	207.512	118.971	134.038	177.124
AMK58_23600	no KO assigned | (GenBank) regulator	Signal transduction	142.013	146.847	175.131	112.189	99.597	192.462
AMK58_23605	no KO assigned | (GenBank) hypothetical protein	Hypothetical	69.618	105.489	82.502	124.343	72.583	150.628
AMK58_23610	K03407 two-component system, chemotaxis family, sensor kinase CheA [EC:2.7.13.3] | (GenBank) histidi	Chemotaxis	23.533	30.056	27.584	221.864	68.361	100.711
AMK58_23615	K03413 two-component system, chemotaxis family, response regulator CheY | (GenBank) two-component	Chemotaxis	41.572	38.071	36.353	73.167	97.551	157.290
AMK58_23620	K00575 chemotaxis protein methyltransferase CheR [EC:2.1.1.80] | (GenBank) chemotaxis protein	Chemotaxis	38.530	35.286	31.420	97.575	144.313	200.156
AMK58_23625	no KO assigned | (GenBank) hypothetical protein	Hypothetical	37.224	30.432	41.167	150.901	133.891	124.986
AMK58_23630	K03412 two-component system, chemotaxis family, response regulator CheB [EC:3.1.1.61] | (GenBank) tw	Chemotaxis	44.595	54.618	49.503	219.533	232.211	145.454
AMK58_23635	K03408 purine-binding chemotaxis protein CheW | (GenBank) hypothetical protein	Chemotaxis	73.822	92.560	99.284	280.168	213.065	153.500
AMK58_23640	K03406 methyl-accepting chemotaxis protein | (GenBank) chemotaxis protein	Chemotaxis	110.202	120.376	123.529	200.778	192.436	126.966
AMK58_23645	K03410 chemotaxis protein CheC | (GenBank) hypothetical protein	Chemotaxis	205.581	242.251	232.220	355.083	312.652	200.820
AMK58_23650	no KO assigned | (GenBank) regulator	Signal transduction	461.558	493.719	432.762	192.220	331.592	253.349
AMK58_23655	K05595 multiple antibiotic resistance protein | (GenBank) antibiotic resistance protein	Transport	25.784	32.372	33.932	207.988	123.702	182.092
AMK58_23660	no KO assigned | (GenBank) aldose epimerase	Enzyme	302.547	360.003	414.908	247.033	227.295	158.529
AMK58_23665	K01647 citrate synthase [EC:2.3.3.1] | (GenBank) gltA; type II citrate synthase	Metabolism: Carbon	2645.768	2601.059	2598.117	115.260	716.900	243.879
AMK58_23670	no KO assigned | (GenBank) flavodoxin	No COG	46.309	55.643	66.907	495.568	204.721	273.477
AMK58_23675	no KO assigned | (GenBank) ATPase	No COG	17.475	22.372	21.956	269.752	120.192	198.112
AMK58_23680	K00059 3-oxoacyl-[acyl-carrier protein] reductase [EC:1.1.1.100] | (GenBank) oxidoreductase	Metabolism	45.520	49.532	42.835	192.150	95.652	66.724
AMK58_23685	K00864 glycerol kinase [EC:2.7.1.30] | (GenBank) glycerol kinase	Lipid metabolism	155.725	149.859	138.663	202.140	178.837	165.721
AMK58_23690	K17321 glycerol transport system substrate-binding protein | (GenBank) ABC transporter substrate-bin	Membrane transport	970.185	919.548	776.249	159.136	522.427	280.386
AMK58_23695	no KO assigned | (GenBank) hypothetical protein	Hypothetical	100.484	103.337	72.047	265.897	254.596	237.905
AMK58_23700	K17323 glycerol transport system permease protein | (GenBank) sugar ABC transporter permease	Transport	145.350	102.430	103.722	96.336	300.155	177.127
AMK58_23705	K17322 glycerol transport system permease protein | (GenBank) ABC transporter permease	Transport: phospholipid	223.407	187.173	165.329	99.694	321.241	200.690
AMK58_23710	K17325 glycerol transport system ATP-binding protein | (GenBank) ABC transporter ATP-binding protein	Transport: Lipid	124.966	128.862	109.833	182.143	374.038	103.713
AMK58_23715	K17324 glycerol transport system ATP-binding protein | (GenBank) ABC transporter ATP-binding protein	Transport	183.806	188.046	189.988	294.990	840.474	215.895
AMK58_23720	K00111 glycerol-3-phosphate dehydrogenase [EC:1.1.5.3] | (GenBank) glycerol-3-phosphate dehydrogena	Metabolism: Lipid	189.071	174.037	170.289	201.628	1138.788	174.156
AMK58_23725	no KO assigned | (GenBank) alkaline phosphatase	enzyme	3.396	2.387	6.079	165.130	421.080	148.211
AMK58_23730	no KO assigned | (GenBank) hypothetical protein	Hypothetical	7.122	17.729	14.108	112.894	332.228	323.050
AMK58_23735	no KO assigned | (GenBank) hypothetical protein	Hypothetical	1.971	3.619	4.763	200.417	27.541	88.596
AMK58_23740	no KO assigned | (GenBank) hypothetical protein	Hypothetical	7.143	9.958	13.131	209.550	32.652	60.937
AMK58_23745	K06160 putative ATP-binding cassette transporter | (GenBank) peptide ABC transporter	Membrane Transport	3.816	4.384	5.233	163.660	19.548	166.227
AMK58_23750	K06160 putative ATP-binding cassette transporter | (GenBank) cyclic peptide transporter	Membrane transport	3.296	4.376	4.875	176.805	10.456	148.063
AMK58_23755	no KO assigned | (GenBank) hypothetical protein	Hypothetical	4.575	3.905	5.904	187.356	4.959	114.997
AMK58_23760	no KO assigned | (GenBank) hypothetical protein	Hypothetical	29.943	35.557	33.422	248.386	17.560	147.623
AMK58_23765	no KO assigned | (GenBank) hypothetical protein	Hypothetical	10.395	10.960	5.233	175.292	62.548	233.612
AMK58_23770	no KO assigned | (GenBank) hypothetical protein	Hypothetical	8.354	7.778	7.943	154.774	33.048	195.466
AMK58_23775	no KO assigned | (GenBank) hypothetical protein	Hypothetical	13.170	18.129	20.831	165.682	29.129	189.480
AMK58_23780	K03406 methyl-accepting chemotaxis protein | (GenBank) hypothetical protein	Chemotaxis	21.059	29.007	30.583	213.321	27.355	206.419

AMK58_23785	K08738 cytochrome c | (GenBank) cytochrome C	Energy	50.448	66.113	51.680	297.341	106.826	317.494
AMK58_23790	no KO assigned | (GenBank) hypothetical protein	Hypothetical	86.749	124.909	103.251	199.388	24.553	249.810
AMK58_23795	no KO assigned | (GenBank) hypothetical protein	Hypothetical	374.914	487.226	513.139	341.895	148.295	288.804
AMK58_23800	no KO assigned | (GenBank) hypothetical protein	Hypothetical	28.059	36.226	38.490	124.139	62.417	172.252
AMK58_23805	no KO assigned | (GenBank) histidine kinase	Signal Transduction	18.807	26.520	31.820	203.808	87.381	210.187
AMK58_23810	no KO assigned | (GenBank) hypothetical protein	Hypothetical	34.356	38.332	51.164	267.815	121.204	130.501
AMK58_23815	no KO assigned | (GenBank) hypothetical protein	Hypothetical	43.556	51.863	54.012	176.058	132.156	71.881
AMK58_23820	K06975 uncharacterized protein | (GenBank) acetyltransferase	Acetyltransferase	116.183	129.084	94.963	343.485	298.764	162.545
AMK58_23825	K01187 alpha-glucosidase [EC:3.2.1.20] | (GenBank) alpha-amylase	Metabolism: Carbohydrate	2.750	3.996	6.714	160.892	166.336	123.435
AMK58_23830	no KO assigned | (GenBank) 3-ketoacyl-ACP reductase	No COG	17.252	22.087	20.944	191.280	278.766	200.257
AMK58_23835	no KO assigned | (GenBank) multidrug DMT transporter permease	No COG	68.021	67.066	71.155	278.544	113.111	117.474
AMK58_23840	no KO assigned | (GenBank) hypothetical protein	Hypothetical	22.495	11.858	22.646	117.873	653.694	446.011
AMK58_23845	K06938 uncharacterized protein | (GenBank) hypothetical protein	Hypothetical	14.268	19.484	22.094	198.227	237.819	223.027
AMK58_23850	no KO assigned | (GenBank) hypothetical protein	Hypothetical	16.845	16.773	25.360	312.619	50.493	86.887
AMK58_23855	K03406 methyl-accepting chemotaxis protein | (GenBank) hypothetical protein	Chemotaxis	29.964	29.979	28.013	161.706	52.939	159.531
AMK58_23860	no KO assigned | (GenBank) hypothetical protein	Hypothetical	130.735	97.473	147.573	201.295	285.217	150.887
AMK58_23865	no KO assigned | (GenBank) hypothetical protein	Hypothetical	17.053	16.980	22.429	295.056	139.607	85.388
AMK58_23870	no KO assigned | (GenBank) hypothetical protein	Hypothetical	15.049	8.174	22.764	351.861	232.589	194.728
AMK58_23875	no KO assigned | (GenBank) hypothetical protein	Hypothetical	51.939	69.191	61.397	242.339	304.533	141.069
AMK58_23880	no KO assigned | (GenBank) hypothetical protein	Hypothetical	337.147	600.942	442.175	270.310	1229.724	256.781
AMK58_23885	no KO assigned | (GenBank) hypothetical protein	Hypothetical	3.830	5.280	8.123	198.688	27.484	57.091
AMK58_23890	no KO assigned | (GenBank) methyltransferase	methyltransferase	24.460	24.355	27.714	169.558	0.000	0.000
AMK58_23895	K05939 acyl-[acyl-carrier-protein]-phospholipid O-acyltransferase / long-chain-fatty-acid--[acyl-car	Metabolism: Lipid	12.814	12.088	22.848	148.017	0.000	0.000
AMK58_23900	no KO assigned | (GenBank) hypothetical protein	Hypothetical	16.851	2.486	20.175	74.405	0.000	0.000
AMK58_23905	K01451 hippurate hydrolase [EC:3.5.1.32] | (GenBank) peptidase M20	Metabolism: Amino Acid	245.453	249.135	221.503	154.858	0.000	0.096
AMK58_23910	no KO assigned | (GenBank) oxidoreductase	Oxidoreductase	68.429	77.926	79.712	232.834	0.000	0.088
AMK58_23915	no KO assigned | (GenBank) hypothetical protein	Hypothetical	108.802	111.394	130.827	232.688	0.531	0.000
AMK58_23920	K01999 branched-chain amino acid transport system substrate-binding protein | (GenBank) amino acid A	Transport: Amino Acid	417.942	355.504	330.463	154.317	0.122	0.101
AMK58_23925	K01997 branched-chain amino acid transport system permease protein | (GenBank) branched-chain amino	Transport: Amino Acid	310.295	243.490	243.322	90.399	0.000	0.000
AMK58_23930	K01998 branched-chain amino acid transport system permease protein | (GenBank) branched-chain amino	Transport: Amino Acid	138.388	100.585	111.827	143.152	0.000	0.000
AMK58_23935	K01995 branched-chain amino acid transport system ATP-binding protein | (GenBank) ABC transporter A'	Transport: Amino Acid	89.545	64.047	65.504	139.801	0.000	0.000
AMK58_23940	K01996 branched-chain amino acid transport system ATP-binding protein | (GenBank) livF; ABC transpor	Transport: Amino Acid	65.647	67.265	65.712	207.895	0.000	0.000
AMK58_23945	no KO assigned | (GenBank) 2-amino-thiazoline-4-carboxylic acid hydrolase	Hydrolase	74.951	61.715	55.275	254.220	0.000	0.223
AMK58_23950	no KO assigned | (GenBank) hypothetical protein	Hypothetical	286.617	289.025	350.776	211.454	0.000	0.246
AMK58_23955	K19784 chromate reductase | (GenBank) NADPH-dependent FMN reductase	Energy	74.161	67.317	73.045	312.023	0.000	0.000
AMK58_23960	K03303 lactate permease | (GenBank) lactate permease	Transport: Carbohydrate	11.058	10.609	15.119	170.944	0.000	0.075
AMK58_23965	K02444 DeoR family transcriptional regulator, glycerol-3-phosphate regulon repressor | (GenBank) Deo	Transcription	29.434	38.978	39.527	220.109	0.000	0.000
AMK58_23970	K10111 multiple sugar transport system ATP-binding protein [EC:3.6.3.-] | (GenBank) hypothetical pro	Transport: Sugar	2.136	2.015	5.131	215.947	0.000	0.000
AMK58_23975	K10238 trehalose/maltose transport system permease protein | (GenBank) sugar ABC transporter permeas	Transport: Carbohydrate	3.082	5.261	4.605	192.264	0.000	0.000
AMK58_23980	K10237 trehalose/maltose transport system permease protein | (GenBank) ABC transporter permease	Transport: Carbohydrate	2.626	2.477	5.388	148.485	0.000	0.000
AMK58_23985	K10236 trehalose/maltose transport system substrate-binding protein | (GenBank) ABC transporter subs	Transport: Carbohydrate	6.282	6.824	9.412	185.392	0.000	0.000
AMK58_23990	K00570 phosphatidylethanolamine/phosphatidyl-N-methylethanolamine N-methyltransferase [EC:2.1.1.17	Metabolism: Lipid	83.762	78.477	77.832	184.882	0.000	0.177
AMK58_23995	K03496 chromosome partitioning protein | (GenBank) hypothetical protein	Cell Cycle/Shape/Homeostatsis	30.800	22.561	28.948	247.705	0.000	0.000
AMK58_24000	K03497 chromosome partitioning protein, ParB family | (GenBank) hypothetical protein	Cell Cycle/Shape/Homeostatsis	20.744	32.037	26.566	185.247	0.000	0.131
AMK58_24005	no KO assigned | (GenBank) hypothetical protein	Hypothetical	87.657	93.683	76.746	248.960	0.000	0.000
AMK58_24010	no KO assigned | (GenBank) hypothetical protein	Hypothetical	32.258	31.413	28.645	183.887	0.000	0.000
AMK58_24015	no KO assigned | (GenBank) hypothetical protein	Hypothetical	4.532	6.447	9.850	183.001	0.000	0.000
AMK58_24020	K00023 acetoacetyl-CoA reductase [EC:1.1.1.36] | (GenBank) 3-ketoacyl-ACP reductase	Metabolism: Carbon	70.096	57.595	56.239	154.306	0.000	0.000
AMK58_24025	no KO assigned | (GenBank) acetyltransferase	Acetyltransferase	11.634	15.377	41.310	171.840	0.000	0.000
AMK58_24030	no KO assigned | (GenBank) hypothetical protein	Hypothetical	6.861	3.617	22.064	136.236	0.000	0.094
AMK58_24035	K00820 glucosamine--fructose-6-phosphate aminotransferase (isomerizing) [EC:2.6.1.16] | (GenBank) ir	Metabolism: Carbohydrates	32.101	28.490	37.931	153.617	0.000	0.108
AMK58_24040	no KO assigned | (GenBank) hypothetical protein	Hypothetical	2.387	0.648	5.983	95.626	0.000	0.101
AMK58_24045	no KO assigned | (GenBank) glucosidase	Metabolism: Carbohydrates	2.892	3.403	4.916	120.627	0.000	0.041
AMK58_24050	K21134 multidrug efflux pump | (GenBank) ACR family transporter	Transport	3.525	3.471	5.377	136.089	0.000	0.108
AMK58_24055	K21137 membrane fusion protein, multidrug efflux system | (GenBank) hypothetical protein	Membrane Transport	0.667	3.321	4.123	229.205	0.000	0.000
AMK58_24060	K21136 membrane fusion protein, multidrug efflux system | (GenBank) hypothetical protein	Membrane Transport	5.016	8.174	8.889	265.270	0.000	0.000
AMK58_24065	K00925 acetate kinase [EC:2.7.2.1] | (GenBank) acetate kinase	Metabolism: Carbon	2.344	4.871	5.911	199.865	0.000	0.095
AMK58_24070	K00625 phosphate acetyltransferase [EC:2.3.1.8] | (GenBank) phosphate acetyltransferase	Metabolism: Carbon	1.892	5.274	5.516	134.648	0.000	0.117
AMK58_24075	no KO assigned | (GenBank) hypothetical protein	Hypothetical	4.315	6.066	5.068	74.803	0.000	0.000
AMK58_24080	no KO assigned | (GenBank) hypothetical protein	Hypothetical	2.480	3.152	5.367	177.804	0.000	0.049
AMK58_24085	no KO assigned | (GenBank) hypothetical protein	Hypothetical	7.228	3.599	13.746	208.179	0.000	0.000
AMK58_24090	no KO assigned | (GenBank) hypothetical protein	Hypothetical	5.300	17.049	10.853	194.951	0.000	0.000
AMK58_24095	no KO assigned | (GenBank) hypothetical protein	Hypothetical	38.869	43.597	47.294	195.900	0.000	0.138
AMK58_24100	no KO assigned | (GenBank) hypothetical protein	Hypothetical	8.870	13.248	16.867	218.327	0.000	0.000
AMK58_24105	no KO assigned | (GenBank) hypothetical protein	Hypothetical	7.031	6.721	14.440	154.839	0.000	0.000
AMK58_24110	no KO assigned | (GenBank) hypothetical protein	Hypothetical	23.090	20.866	26.566	194.675	0.000	0.000
AMK58_24115	no KO assigned | (GenBank) hypothetical protein	Hypothetical	5.820	7.559	8.060	138.036	0.000	0.117

AMK58_24120	no KO assigned | (GenBank) hypothetical protein	Hypothetical	2.275	1.510	4.967	173.811	0.000	0.000
AMK58_24125	no KO assigned | (GenBank) polyphosphate kinase	Signal Transduction	6.411	11.316	11.360	90.949	0.000	0.135
AMK58_24130	no KO assigned | (GenBank) hypothetical protein	Hypothetical	3.363	8.611	3.655	108.768	0.000	0.000
AMK58_24135	no KO assigned | (GenBank) hypothetical protein	Hypothetical	12.604	11.790	18.884	159.457	0.000	0.000
AMK58_24140	no KO assigned | (GenBank) hypothetical protein	Hypothetical	6.529	10.638	9.875	79.105	0.000	0.275
AMK58_24145	K07444 putative N6-adenine-specific DNA methylase [EC:2.1.1.-] | (GenBank) RNA methyltransferase	DNA Repair and Replication	21.024	18.936	24.310	194.736	0.000	0.098
AMK58_24150	no KO assigned | (GenBank) dehydrogenase	Dehydrogenase	26.809	28.245	31.356	232.930	0.000	0.053
AMK58_24155	no KO assigned | (GenBank) hypothetical protein	Hypothetical	6.400	8.498	9.128	123.366	0.000	0.000
AMK58_24160	no KO assigned | (GenBank) hypothetical protein	Hypothetical	35.237	41.614	35.743	127.745	0.000	0.000
AMK58_24165	no KO assigned | (GenBank) hypothetical protein	Hypothetical	54.547	56.512	57.859	247.925	0.177	0.146
AMK58_24170	no KO assigned | (GenBank) esterase	esterase	54.009	62.053	56.995	148.869	0.000	0.138
AMK58_24175	K18118 succinyl-CoA:acetate CoA-transferase [EC:2.8.3.18] | (GenBank) acetyl-CoA hydrolase	Metabolism: Carbon	113.538	120.075	97.474	183.246	0.000	0.000
AMK58_24180	K12252 arginine:pyruvate transaminase [EC:2.6.1.84] | (GenBank) aspartate aminotransferase	Metabolism: Amino Acid	27.112	35.240	27.156	135.432	0.000	0.000
AMK58_24185	K07799 membrane fusion protein, multidrug efflux system | (GenBank) multidrug transporter	Membrane Transport	20.940	18.712	22.462	180.167	0.000	0.000
AMK58_24190	K07788 multidrug efflux pump | (GenBank) multidrug transporter	Membrane Transport	18.930	13.525	16.477	179.963	0.000	0.145
AMK58_24195	K07789 multidrug efflux pump | (GenBank) nodulation protein	Membrane Transport	11.128	9.751	11.357	178.385	0.000	0.069
AMK58_24200	no KO assigned | (GenBank) hypothetical protein	Hypothetical	5.126	8.264	10.676	258.756	0.000	0.000
AMK58_24205	no KO assigned | (GenBank) FAD-dependent oxidoreductase	Oxidoreductase	29.509	47.100	51.920	212.179	0.000	0.067
AMK58_24210	K14986 two-component system, LuxR family, sensor kinase FixL [EC:2.7.13.3] | (GenBank) PAS domain	Signal Transduction	135.188	152.535	146.632	194.899	0.000	0.074
AMK58_24215	K14987 two-component system, LuxR family, response regulator FixJ | (GenBank) fixJ; two-component sy	Signal Transduction	72.309	85.954	90.070	110.376	0.000	0.000
AMK58_24220	no KO assigned | (GenBank) transcriptional regulator	Transcription	153.361	148.529	113.584	195.488	0.363	0.000
AMK58_24225	no KO assigned | (GenBank) nitroreductase	Nitrogen	50.834	47.198	45.394	132.509	0.000	0.000
AMK58_24230	no KO assigned | (GenBank) hypothetical protein	Hypothetical	6.089	1.692	7.271	133.922	0.000	0.000
AMK58_24235	no KO assigned | (GenBank) hypothetical protein	Hypothetical	52.696	36.230	37.578	141.457	0.000	0.194
AMK58_24240	no KO assigned | (GenBank) Crp/Fnr family transcriptional regulator	Transcription	31.178	42.429	37.385	180.284	0.000	0.000
AMK58_24245	K01768 adenylate cyclase [EC:4.6.1.1] | (GenBank) hypothetical protein	cyclic nucleotide	25.055	25.090	23.619	231.599	0.000	0.264
AMK58_24250	no KO assigned | (GenBank) hypothetical protein	Hypothetical	49.430	34.287	35.557	216.000	0.000	0.343
AMK58_24255	K07667 two-component system, OmpR family, KDP operon response regulator KdpE | (GenBank) two-co	Signal Transduction	5.543	3.105	8.235	205.575	0.000	0.161
AMK58_24260	K07646 two-component system, OmpR family, sensor histidine kinase KdpD [EC:2.7.13.3] | (GenBank) h	Signal Transduction	5.012	5.034	6.536	259.260	0.050	0.000
AMK58_24265	K01548 K+-transporting ATPase ATPase C chain [EC:3.6.3.12] | (GenBank) potassium-transporting ATP	Transport: Ion	0.596	1.188	5.860	250.336	0.000	0.000
AMK58_24270	K01547 K+-transporting ATPase ATPase B chain [EC:3.6.3.12] | (GenBank) potassium-transporting ATP	Transport: Ion	1.819	3.396	3.621	184.496	0.000	0.000
AMK58_24275	K01546 K+-transporting ATPase ATPase A chain [EC:3.6.3.12] | (GenBank) potassium-transporting ATP	Transport: Ion	1.558	1.692	5.116	265.188	0.000	0.066
AMK58_24280	no KO assigned | (GenBank) potassium transporter TrkH	Transport: Ion	0.000	8.037	3.837	191.019	0.000	0.000
AMK58_24285	K11475 GntR family transcriptional regulator, vanillate catabolism transcriptional regulator | (GenB	Transcription	7.663	5.087	8.905	114.055	0.000	0.158
AMK58_24290	K07120 uncharacterized protein | (GenBank) hypothetical protein	Hypothetical	1.258	0.000	5.000	210.581	0.000	0.000
AMK58_24295	K13995 maleamate amidohydrolase [EC:3.5.1.107] | (GenBank) isochorismatase	Metabolism: Co-Factors and Vitamens	6.109	11.060	12.498	193.185	0.000	0.000
AMK58_24300	no KO assigned | (GenBank) dipeptide/oligopeptide/nickel ABC transporter ATP-binding protein	Membrane Transport	2.456	1.914	5.177	161.328	0.000	0.099
AMK58_24305	no KO assigned | (GenBank) methionine ABC transporter ATP-binding protein	Membrane Transport	0.915	2.049	1.957	88.214	0.000	0.000
AMK58_24310	K02034 peptide/nickel transport system permease protein | (GenBank) peptide ABC transporter permease	Transport: Metal	1.909	0.000	3.760	210.038	0.000	0.000
AMK58_24315	K02033 peptide/nickel transport system permease protein | (GenBank) peptide ABC transporter permease	Transport: Metal	4.771	4.622	9.685	173.067	196.096	186.040
AMK58_24320	K02035 peptide/nickel transport system substrate-binding protein | (GenBank) ABC transporter substra	Transport: Metal	4.280	8.220	6.832	104.438	118.824	190.589
AMK58_24325	K13995 maleamate amidohydrolase [EC:3.5.1.107] | (GenBank) N-carbamoylsarcosine amidase	Metabolism: Co-Factors and Vitamens	1.153	3.445	5.299	224.743	170.008	265.883
AMK58_24330	K02433 aspartyl-tRNA(Asn)/glutamyl-tRNA(Gln) amidotransferase subunit A [EC:6.3.5.6 6.3.5.7] | (Gen	tRNA synthesis/modification	0.519	1.549	2.383	242.989	71.301	120.363
AMK58_24335	no KO assigned | (GenBank) amidase	Enzyme	0.660	1.690	2.600	237.847	44.092	100.640
AMK58_24340	no KO assigned | (GenBank) alanine acetyltransferase	Acetyltransferase	58.703	39.456	55.583	290.296	283.459	255.839
AMK58_24345	K01992 ABC-2 type transport system permease protein | (GenBank) sugar ABC transporter permease	Membrane Transport	279.330	238.271	218.605	56.190	238.431	161.038
AMK58_24350	K01990 ABC-2 type transport system ATP-binding protein | (GenBank) multidrug ABC transporter ATP-l	Membrane Transport	158.037	181.034	147.779	170.759	156.940	235.682
AMK58_24355	K06950 uncharacterized protein | (GenBank) hydrolase	hydrolase	311.360	377.541	332.398	239.248	748.513	209.931
AMK58_24360	no KO assigned | (GenBank) hypothetical protein	Hypothetical	20.022	25.579	26.161	202.163	237.650	177.717
AMK58_24365	no KO assigned | (GenBank) hypothetical protein	Hypothetical	33.528	40.805	74.385	252.012	170.291	371.929
AMK58_24370	no KO assigned | (GenBank) hypothetical protein	Hypothetical	50.100	83.144	59.544	137.685	231.515	246.846
AMK58_24375	K09781 uncharacterized protein | (GenBank) ABC transporter	Membrane Transport	22.098	18.761	25.766	103.917	135.962	110.544
AMK58_24380	no KO assigned | (GenBank) C4-dicarboxylate ABC transporter	Membrane Transport	61.295	55.469	69.630	135.282	103.082	148.814
AMK58_24385	no KO assigned | (GenBank) C4-dicarboxylate ABC transporter permease	Membrane Transport	55.755	37.631	55.896	109.061	72.111	225.621
AMK58_24390	no KO assigned | (GenBank) C4-dicarboxylate ABC transporter	Membrane Transport	140.639	152.904	140.389	283.496	112.858	243.365
AMK58_24395	no KO assigned | (GenBank) GntR family transcriptional regulator	Transcription	18.161	24.112	24.463	346.222	85.058	258.376
AMK58_24400	no KO assigned | (GenBank) LysR family transcriptional regulator	Transcription	97.159	103.916	105.170	102.481	136.246	261.081
AMK58_24405	no KO assigned | (GenBank) hypothetical protein	Hypothetical	413.761	496.107	514.639	180.366	97.754	362.641
AMK58_24410	no KO assigned | (GenBank) ArsR family transcriptional regulator	Transcription	11.531	10.483	15.016	181.997	38.038	348.199
AMK58_24415	K00163 pyruvate dehydrogenase E1 component [EC:1.2.4.1] | (GenBank) transketolase	Metabolism: Carbon	20.908	21.319	23.033	137.747	175.865	169.500
AMK58_24420	no KO assigned | (GenBank) hypothetical protein	Hypothetical	43.721	47.915	44.031	140.353	120.972	171.523
AMK58_24425	no KO assigned | (GenBank) hypothetical protein	Hypothetical	9.313	9.274	11.217	91.905	219.145	313.779
AMK58_24430	no KO assigned | (GenBank) protease	protease	63.213	68.606	72.082	275.387	356.524	228.778
AMK58_24435	K09004 uncharacterized protein | (GenBank) hypothetical protein	Hypothetical	28.303	23.486	30.399	211.684	332.279	120.775
AMK58_24440	K00826 branched-chain amino acid aminotransferase [EC:2.6.1.42] | (GenBank) 2-keto-4-methylthiobutyr	Metabolism	15.041	12.456	18.125	186.125	111.319	60.485
AMK58_24445	K01665 para-aminobenzoate synthetase component I [EC:2.6.1.85] | (GenBank) aminobenzoate synthetase	Metabolism: Co-Factors and Vitamens	16.446	25.493	21.722	136.584	80.637	57.536
AMK58_24450	K00046 gluconate 5-dehydrogenase [EC:1.1.1.69] | (GenBank) gluconate 5-dehydrogenase	Oxidoreductases	58.997	53.377	60.928	173.328	125.185	97.272

AMK58_24455	no KO assigned | (GenBank) hypothetical protein	Hypothetical	15.160	25.703	25.322	156.972	55.497	75.986
AMK58_24460	K03672 thioredoxin 2 [EC:1.8.1.8] | (GenBank) thioredoxin	Chaperone	24.215	46.616	50.652	331.580	105.850	126.241
AMK58_24465	no KO assigned | (GenBank) two-component system response regulator	Signal Transduction	22.949	41.368	44.016	294.426	113.633	113.921
AMK58_24470	K13040 two-component system, LuxR family, sensor histidine kinase TtrS [EC:2.7.13.3] | (GenBank) hyp	Signal Transduction	192.398	241.950	365.337	193.411	58.739	86.202
AMK58_24475	K17218 sulfide:quinone oxidoreductase [EC:1.8.5.4] | (GenBank) pyridine nucleotide-disulfide oxidore	Metabolism	1653.101	2186.253	2778.221	174.575	74.557	177.674
AMK58_24480	no KO assigned | (GenBank) sulfurtransferase	Transferase	3705.245	4474.876	6006.500	275.080	75.385	337.359
AMK58_24485	no KO assigned | (GenBank) RND transporter	Transport	215.320	308.248	422.866	138.841	21.136	81.863
AMK58_24490	no KO assigned | (GenBank) multidrug transporter AcrB	Transport	410.910	572.240	815.346	218.091	45.615	143.036
AMK58_24495	no KO assigned | (GenBank) sulfurtransferase	Transferase	137.917	241.119	308.797	277.121	33.220	109.028
AMK58_24500	no KO assigned | (GenBank) ArsR family transcriptional regulator	Transcription	100.033	208.145	276.154	305.889	46.915	182.638
AMK58_24505	no KO assigned | (GenBank) hypothetical protein	Hypothetical	9.113	11.667	26.923	271.618	118.533	192.549
AMK58_24510	K03088 RNA polymerase sigma-70 factor, ECF subfamily | (GenBank) RNA polymerase	Transcription	542.854	641.124	704.909	266.247	7182.784	226.054
AMK58_24515	no KO assigned | (GenBank) hypothetical protein	Hypothetical	433.740	517.566	568.426	183.771	5038.772	157.918
AMK58_24520	no KO assigned | (GenBank) hypothetical protein	Hypothetical	287.192	333.691	335.421	201.767	4888.824	286.354
AMK58_24525	no KO assigned | (GenBank) hypothetical protein	Hypothetical	114.068	147.350	151.893	158.807	1961.695	123.705
AMK58_24530	no KO assigned | (GenBank) hypothetical protein	Hypothetical	50.646	70.917	60.193	237.979	719.159	212.799
AMK58_24535	no KO assigned | (GenBank) extensin	enzyme	16.874	19.747	25.472	218.578	536.747	129.530
AMK58_24540	no KO assigned | (GenBank) MBL fold metallo-hydrolase	No COG	195.860	221.662	202.149	224.349	310.360	256.678
AMK58_24545	no KO assigned | (GenBank) hypothetical protein	Hypothetical	36.515	38.273	35.328	156.179	255.625	187.116
AMK58_24550	K13821 RHH-type transcriptional regulator, proline utilization regulon repressor / proline dehydroge	Transcription	32.461	30.937	32.926	143.414	89.965	95.974
AMK58_24555	K00286 pyrroline-5-carboxylate reductase [EC:1.5.1.2] | (GenBank) pyrroline-5-carboxylate reductase	Metabolism: Amino Acid	35.402	27.073	34.603	131.266	57.780	53.443
AMK58_24560	K03719 Lrp/AsnC family transcriptional regulator, leucine-responsive regulatory protein | (GenBank)	Transcription	39.310	54.800	62.792	113.390	100.450	267.309
AMK58_24565	no KO assigned | (GenBank) hypothetical protein	Hypothetical	8.888	10.353	13.659	211.231	54.035	309.493
AMK58_24570	no KO assigned | (GenBank) hypothetical protein	Hypothetical	108.446	100.876	205.831	141.039	109.245	221.484
AMK58_24575	no KO assigned | (GenBank) hypothetical protein	Hypothetical	42.917	43.082	80.727	170.755	78.048	176.439
AMK58_24580	no KO assigned | (GenBank) hypothetical protein	Hypothetical	5.935	0.000	31.601	217.838	17.345	109.518
AMK58_24585	no KO assigned | (GenBank) hypothetical protein	Hypothetical	4.264	5.924	15.368	136.815	19.394	224.999
AMK58_24590	no KO assigned | (GenBank) hypothetical protein	Hypothetical	10.988	6.749	28.999	128.758	23.663	192.068
AMK58_24595	K13015 UDP-N-acetyl-D-glucosamine dehydrogenase [EC:1.1.1.136] | (GenBank) UDP-N-acetyl-D-gluc	Metabolism: Carbohydrate	34.010	23.299	94.165	180.773	47.913	226.894
AMK58_24600	K16554 polysaccharide biosynthesis transport protein | (GenBank) hypothetical protein	Transport: polysacchharide	17.788	14.769	55.643	152.612	38.478	187.814
AMK58_24605	no KO assigned | (GenBank) hypothetical protein	Hypothetical	8.698	8.834	21.419	132.475	19.260	157.436
AMK58_24610	no KO assigned | (GenBank) hypothetical protein	Hypothetical	4.644	3.531	16.296	172.365	17.368	116.341
AMK58_24615	no KO assigned | (GenBank) hypothetical protein	Hypothetical	6.159	10.177	15.573	203.083	18.264	137.178
AMK58_24620	no KO assigned | (GenBank) hypothetical protein	Hypothetical	12.955	19.349	20.963	192.888	31.923	122.087
AMK58_24625	K07088 uncharacterized protein | (GenBank) permease	Enzyme	97.974	75.502	85.015	271.402	49.947	201.607
AMK58_24630	K01816 hydroxypyruvate isomerase [EC:5.3.1.22] | (GenBank) hydroxypyruvate isomerase	Metabolism: Carbohydrate	174.783	142.104	127.667	219.948	97.973	190.673
AMK58_24635	K07120 uncharacterized protein | (GenBank) hypothetical protein	Hypothetical	31.416	45.693	47.547	238.472	100.384	71.682
AMK58_24640	no KO assigned | (GenBank) hypothetical protein	Hypothetical	201.283	214.778	207.666	202.392	263.990	222.023
AMK58_24645	no KO assigned | (GenBank) flavodoxin	Enzyme	1.193	2.376	2.268	104.129	82.237	279.107
AMK58_24650	no KO assigned | (GenBank) peptidase M48 Ste24p	peptidase	60.225	50.467	71.094	284.559	56.793	185.521
AMK58_24655	K03496 chromosome partitioning protein | (GenBank) hypothetical protein	Cell Cycle/Shape/Homeostatsis	40.826	38.439	38.484	151.060	61.669	184.838
AMK58_24660	K03497 chromosome partitioning protein, ParB family | (GenBank) hypothetical protein	Cell Cycle/Shape/Homeostatsis	20.652	20.967	21.946	205.580	43.999	160.332
AMK58_24665	K07168 CBS domain-containing membrane protein | (GenBank) hypothetical protein	Membrane	22.004	12.461	19.831	259.693	52.632	180.250
AMK58_24670	K02471 putative ATP-binding cassette transporter | (GenBank) hypothetical protein	Membrane transport	16.143	22.208	22.519	194.196	37.724	195.093
AMK58_24675	no KO assigned | (GenBank) chemotaxis protein	Chemotaxis	5.612	6.420	7.152	228.404	58.048	182.023
AMK58_24680	K00064 D-threo-aldose 1-dehydrogenase [EC:1.1.1.122] | (GenBank) D-threo-aldose 1-dehydrogenase	Metabolism: Carbohydrate	48.945	77.686	65.432	219.534	186.697	176.993
AMK58_24685	K17850 LysR family transcriptional regulator, regulator of gene expression of beta-lactamase | (GenB	Transcription	18.797	28.075	24.706	223.098	102.381	102.914
AMK58_24690	K17836 beta-lactamase class A [EC:3.5.2.6] | (GenBank) class A beta-lactamase	Defense	7.202	9.742	10.078	271.038	66.137	162.923
AMK58_24695	K08358 tetrathionate reductase subunit B | (GenBank) tetrathionate reductase subunit B	Energy	1.922	3.827	3.959	122.927	39.784	185.037
AMK58_24700	K08359 tetrathionate reductase subunit C | (GenBank) tetrathionate reductase	Energy	1.922	4.465	3.655	161.042	34.563	45.541
AMK58_24705	K08357 tetrathionate reductase subunit A | (GenBank) tetrathionate reductase subunit A	Energy	2.220	5.353	7.260	255.229	54.472	127.188
AMK58_24710	no KO assigned | (GenBank) exodeoxyribonuclease V	No COG	29.535	33.841	32.891	233.320	66.512	174.829
AMK58_24715	no KO assigned | (GenBank) hypothetical protein	Hypothetical	22.328	29.435	32.891	330.831	19.560	72.222
AMK58_24720	K02028 polar amino acid transport system ATP-binding protein [EC:3.6.3.21] | (GenBank) polar amino a	Transport: Amino Acid	142.450	196.467	188.312	182.409	20.162	182.404
AMK58_24725	K02029 polar amino acid transport system permease protein | (GenBank) ABC transporter permease	Transport: Amino Acid	265.524	378.589	354.559	118.398	75.539	189.148
AMK58_24730	K02030 polar amino acid transport system substrate-binding protein | (GenBank) ABC transporter perme	Transport: Amino Acid	685.180	938.187	785.781	163.405	48.093	167.138
AMK58_24735	no KO assigned | (GenBank) N-formylglutamate deformylase	Cell Wall	111.401	166.164	168.439	265.653	41.287	153.057
AMK58_24740	K05603 formimidoylglutamate deiminase [EC:3.5.3.13] | (GenBank) N-formimino-L-glutamate deiminase	Metabolism: Amino Acid	50.469	69.622	71.206	161.612	24.753	65.550
AMK58_24745	no KO assigned | (GenBank) hypothetical protein	Hypothetical	56.778	80.678	82.582	150.880	60.543	210.879
AMK58_24750	no KO assigned | (GenBank) hypothetical protein	Hypothetical	28.686	24.821	38.561	229.498	78.276	219.036
AMK58_24755	K09975 uncharacterized protein | (GenBank) hypothetical protein	Hypothetical	33.667	27.485	38.771	223.251	68.399	111.300
AMK58_24760	K01284 peptidyl-dipeptidase Dcp [EC:3.4.15.5] | (GenBank) peptidase M3	peptidase	169.445	162.400	148.470	154.951	166.314	219.146
AMK58_24765	K02000 glycine betaine/proline transport system ATP-binding protein [EC:3.6.3.32] | (GenBank) prolin	Transport: organic	5.159	3.022	7.213	136.849	1107.392	199.872
AMK58_24770	K02001 glycine betaine/proline transport system permease protein | (GenBank) glycine/betaine ABC tra	Transport: organic	2.321	0.770	7.478	165.813	572.055	168.823
AMK58_24775	K02002 glycine betaine/proline transport system substrate-binding protein | (GenBank) glycine/betain	Transport: organic	21.742	17.861	24.032	145.803	661.984	170.862
AMK58_24780	no KO assigned | (GenBank) hypothetical protein	Hypothetical	11.119	15.247	16.847	210.925	322.181	192.322
AMK58_24785	K07814 putative two-component system response regulator | (GenBank) transcriptional regulator	Signal Transduction	19.386	31.833	19.402	168.412	162.726	190.795

AMK58_24790	no KO assigned | (GenBank) hypothetical protein	Hypothetical	51.559	71.167	71.168	175.046	86.132	140.955
AMK58_24795	no KO assigned | (GenBank) organic hydroperoxide resistance protein	Defense	23.350	29.279	23.846	216.634	141.926	285.218
AMK58_24800	no KO assigned | (GenBank) MarR family transcriptional regulator	Transcription	34.782	50.762	52.510	137.289	64.167	122.118
AMK58_24805	no KO assigned | (GenBank) plasmid replication protein	DNA Repair and Replication	143.341	125.904	124.287	102.798	84.025	184.259
AMK58_24810	no KO assigned | (GenBank) hypothetical protein	Hypothetical	34.037	24.693	43.343	210.874	36.756	168.517
AMK58_24815	no KO assigned | (GenBank) hypothetical protein	Hypothetical	30.489	28.429	24.465	243.869	15.254	59.812
AMK58_24820	no KO assigned | (GenBank) C4-dicarboxylate ABC transporter substrate-binding protein	Membrane Transport	16.167	11.813	11.943	202.226	21.310	139.447
AMK58_24825	K01768 adenylate cyclase [EC:4.6.1.1] | (GenBank) hypothetical protein	Metabolism: Nucleotide	3.043	3.430	4.913	209.096	15.681	104.018
AMK58_24830	no KO assigned | (GenBank) hypothetical protein	Hypothetical	1.376	4.110	5.669	259.252	11.599	79.524
AMK58_24835	no KO assigned | (GenBank) hypothetical protein	Hypothetical	0.290	1.156	2.024	180.336	11.368	66.814
AMK58_24840	no KO assigned | (GenBank) hypothetical protein	Hypothetical	0.102	3.060	3.311	175.907	15.544	150.168
AMK58_24845	K01775 alanine racemase [EC:5.1.1.1] | (GenBank) hypothetical protein	Metabolism: Amino Acid	0.307	1.224	1.753	186.610	13.471	68.483
AMK58_24850	K07001 NTE family protein | (GenBank) alpha/beta hydrolase	hydrolase	2.274	2.717	2.919	126.094	23.513	177.734
AMK58_24855	K03088 RNA polymerase sigma-70 factor, ECF subfamily | (GenBank) hypothetical protein	Transcription	2.458	3.672	7.012	393.253	12.435	354.286
AMK58_24860	K14235 tRNA Trp | (GenBank) tRNA-Trp	tRNA synthesis/modification	20.710	38.071	30.294	471.619	3.581	827.250
AMK58_24865	no KO assigned | (GenBank) molybdate ABC transporter substrate-binding protein	Membrane Transport	13.559	18.108	15.319	169.237	17.931	209.895
AMK58_24870	no KO assigned | (GenBank) hemolysin activation/secretion protein	Secretion system	57.905	70.326	60.827	94.902	27.860	62.575
AMK58_24875	no KO assigned | (GenBank) hypothetical protein	Hypothetical	8.157	7.658	7.165	14.153	1.965	11.436
AMK58_24880	no KO assigned | (GenBank) hypothetical protein	Hypothetical	0.000	0.000	0.000	0.000	0.000	0.000
AMK58_24885	no KO assigned | (GenBank) hypothetical protein	Hypothetical	0.320	0.000	0.000	0.000	0.000	0.000
AMK58_24890	no KO assigned | (GenBank) hypothetical protein	Hypothetical	0.472	0.000	0.000	0.000	0.000	0.000
AMK58_24895	no KO assigned | (GenBank) pseudogene	Pseudogene	0.000	0.000	0.000	0.000	0.000	0.000
AMK58_24900	no KO assigned | (GenBank) pseudogene	Pseudogene	0.000	0.000	0.000	0.000	0.000	0.000
AMK58_24905	no KO assigned | (GenBank) dehydratase	dehydratase	0.000	0.000	0.000	0.000	0.000	0.000
AMK58_24910	K18292 (S)-citramalyl-CoA lyase [EC:4.1.3.25] | (GenBank) hypothetical protein	Metabolism: Carbohydrate	0.000	0.000	0.000	0.000	0.000	0.000
AMK58_24915	no KO assigned | (GenBank) hypothetical protein	Hypothetical	0.000	0.000	0.000	0.000	0.000	0.000
AMK58_24920	K11688 C4-dicarboxylate-binding protein DctP | (GenBank) hypothetical protein	Transport	0.000	0.000	0.000	0.000	0.000	0.000
AMK58_24925	no KO assigned | (GenBank) 3-oxoacyl-ACP reductase	No COG	0.000	0.000	0.000	0.000	0.000	0.000
AMK58_24930	no KO assigned | (GenBank) glycosyl transferase	Glycosyl Transferase	0.000	0.000	0.000	0.000	0.000	0.000
AMK58_24935	no KO assigned | (GenBank) hypothetical protein	Hypothetical	11.099	10.686	13.823	120.656	17.527	49.883
AMK58_24940	K03088 RNA polymerase sigma-70 factor, ECF subfamily | (GenBank) RNA polymerase	Transcription	43.385	47.018	55.833	286.258	353.842	130.607
AMK58_24945	no KO assigned | (GenBank) hypothetical protein	Hypothetical	47.085	45.210	63.156	158.807	387.803	91.219
AMK58_24950	no KO assigned | (GenBank) hypothetical protein	Hypothetical	35.443	26.276	59.035	165.998	354.482	130.262
AMK58_24955	K06867 uncharacterized protein | (GenBank) hypothetical protein	Hypothetical	10.257	10.657	12.296	137.694	392.239	94.488
AMK58_24960	K03566 LysR family transcriptional regulator, glycine cleavage system transcriptional activator | (G	Transcription	10.511	14.731	18.508	240.584	101.669	100.597
AMK58_24965	no KO assigned | (GenBank) hypothetical protein	Hypothetical	45.707	66.816	58.253	244.256	95.648	102.350
AMK58_24970	no KO assigned | (GenBank) hypothetical protein	Hypothetical	28.128	42.013	39.827	163.824	64.610	59.820
AMK58_24975	K01759 lactoylglutathione lyase [EC:4.4.1.5] | (GenBank) lactoylglutathione lyase	Metabolism: Carbohydrate	1102.820	1158.100	1030.256	103.619	63.579	214.050
AMK58_24980	no KO assigned | (GenBank) Twin-arginine translocation pathway signal	No COG	752.287	884.101	795.681	242.564	44.164	154.643
AMK58_24985	no KO assigned | (GenBank) 2-keto-gluconate dehydrogenase	dehydrogenase	1025.670	1152.675	1052.181	129.068	78.522	227.110
AMK58_24990	no KO assigned | (GenBank) pseudogene	Pseudogene	320.944	430.569	345.357	430.565	115.031	479.819
AMK58_24995	K01854 UDP-galactopyranose mutase [EC:5.4.99.9] | (GenBank) UDP-galactopyranose mutase	Metabolism: Carbohydrate	3.258	5.651	5.296	181.051	34.378	197.398
AMK58_25000	no KO assigned | (GenBank) beta-glucosidase	No COG	1.054	4.407	3.206	297.411	15.161	86.865
AMK58_25005	K06147 ATP-binding cassette, subfamily B, bacterial | (GenBank) ABC transporter	Transport	1.028	4.503	5.277	207.712	30.269	149.502
AMK58_25010	no KO assigned | (GenBank) hypothetical protein	Hypothetical	43.441	64.460	63.132	222.931	64.924	173.087
AMK58_25015	K02824 uracil permease | (GenBank) uracil permease	Transport	153.952	90.488	103.967	227.258	115.435	198.677
AMK58_25020	K13069 diguanylate cyclase [EC:2.7.7.65] | (GenBank) diguanylate cyclase	cyclic nucleotide	43.454	56.901	56.711	177.814	82.700	227.078
AMK58_25025	no KO assigned | (GenBank) hypothetical protein	Hypothetical	68.609	69.322	82.742	225.371	506.380	295.643
AMK58_25030	K13609 delta1-piperideine-2-carboxylate reductase [EC:1.5.1.21] | (GenBank) lactate dehydrogenase	Metabolism: Amino Acid	71.990	60.099	77.092	222.904	37.872	124.856
AMK58_25035	K02433 aspartyl-tRNA(Asn)/glutamyl-tRNA(Gln) amidotransferase subunit A [EC:6.3.5.6 6.3.5.7] | (Gen	tRNA synthesis/modification	29.072	27.510	27.814	159.646	23.748	49.395
AMK58_25040	K00681 gamma-glutamyltranspeptidase / glutathione hydrolase [EC:2.3.2.2 3.4.19.13] | (GenBank) gamma	Metabolism: Amino Acid	18.005	20.967	21.979	125.497	22.039	41.880
AMK58_25045	no KO assigned | (GenBank) hypothetical protein	Hypothetical	280.631	243.922	261.026	94.294	128.181	187.551
AMK58_25050	K09947 uncharacterized protein | (GenBank) hypothetical protein	Hypothetical	118.599	118.309	115.631	147.049	13.912	73.649
AMK58_25055	K07338 uncharacterized protein | (GenBank) hypothetical protein	Hypothetical	63.956	63.244	58.084	124.963	7.313	28.016
AMK58_25060	no KO assigned | (GenBank) hypothetical protein	Hypothetical	216.721	189.327	170.526	194.829	10.285	73.239
AMK58_25065	K07231 putative iron-regulated protein | (GenBank) peptidase	peptidase	2361.289	2133.362	1922.603	200.422	59.813	218.404
AMK58_25070	no KO assigned | (GenBank) methylamine utilization protein MauG	No COG	146.842	127.978	122.400	167.869	76.538	192.490
AMK58_25075	K02651 pilus assembly protein Flp/PilA | (GenBank) pilus assembly protein	Secretion System	113.117	163.010	319.955	395.185	26.196	430.631
AMK58_25080	K02278 prepilin peptidase CpaA [EC:3.4.23.43] | (GenBank) hypothetical protein	Secretion system	23.666	30.298	72.125	225.305	21.138	140.876
AMK58_25085	K02279 pilus assembly protein CpaB | (GenBank) hypothetical protein	Secretion System	4.189	6.675	17.128	218.306	10.517	61.250
AMK58_25090	K02280 pilus assembly protein CpaC | (GenBank) hypothetical protein	Secretion System	7.624	10.397	20.881	167.403	18.723	168.981
AMK58_25095	K02281 pilus assembly protein CpaD | (GenBank) hypothetical protein	Secretion System	4.338	5.634	9.145	198.311	16.323	58.408
AMK58_25100	K02282 pilus assembly protein CpaE | (GenBank) hypothetical protein	Secretion System	1.498	4.775	5.984	77.614	5.165	28.067
AMK58_25105	K02283 pilus assembly protein CpaF | (GenBank) hypothetical protein	Secretion System	4.687	9.334	18.898	151.025	17.658	138.265
AMK58_25110	no KO assigned | (GenBank) hypothetical protein	Hypothetical	2.256	2.995	8.938	121.052	7.467	59.157
AMK58_25115	K12511 tight adherence protein C | (GenBank) hypothetical protein	Secretion system	0.828	1.414	2.813	101.148	5.321	42.911
AMK58_25120	no KO assigned | (GenBank) hypothetical protein	Hypothetical	2.914	5.223	7.342	158.673	11.955	73.090

AMK58_25125	no KO assigned | (GenBank) hypothetical protein	Hypothetical	8.364	4.997	20.678	298.041	20.214	130.690
AMK58_25130	no KO assigned | (GenBank) hypothetical protein	Hypothetical	7.901	14.033	13.197	276.317	26.643	111.874
AMK58_25135	no KO assigned | (GenBank) hypothetical protein	Hypothetical	7.112	5.631	14.502	229.907	33.518	215.640
AMK58_25140	no KO assigned | (GenBank) hypothetical protein	Hypothetical	424.246	522.745	386.186	56.657	63.510	212.265
AMK58_25145	K14220 tRNA Asn | (GenBank) tRNA-Asn	tRNA synthesis/modification	87.620	104.696	106.031	29.876	19.698	161.018
AMK58_25150	K14221 tRNA Asp | (GenBank) tRNA-Asp	tRNA synthesis/modification	0.000	9.394	0.000	0.000	0.000	4.374
AMK58_25155	K14225 tRNA Gly | (GenBank) tRNA-Gly	tRNA synthesis/modification	172.733	221.829	190.330	190.298	16.331	399.680
AMK58_25160	no KO assigned | (GenBank) AraC family transcriptional regulator	Transcription	10.247	17.896	15.439	275.800	62.199	193.841
AMK58_25165	K07006 uncharacterized protein | (GenBank) pyridoxamine 5'-phosphate oxidase	enzyme	5.600	8.076	6.977	171.757	37.116	287.210
AMK58_25170	K01985 5S ribosomal RNA | (GenBank) 5S ribosomal RNA	Ribosome	0.000	6.236	2.977	27.963	0.000	20.325
AMK58_25175	K01980 23S ribosomal RNA | (GenBank) 23S ribosomal RNA	Ribosome	0.000	0.000	0.000	0.000	0.000	0.000
AMK58_25180	K14218 tRNA Ala | (GenBank) tRNA-Ala	tRNA synthesis/modification	0.000	0.000	0.000	0.000	0.000	0.000
AMK58_25185	K14227 tRNA Ile | (GenBank) tRNA-Ile	tRNA synthesis/modification	0.000	0.000	0.000	0.000	0.000	0.000
AMK58_25190	K01977 16S ribosomal RNA | (GenBank) 16S ribosomal RNA	Ribosome	0.000	0.000	0.000	0.000	0.000	0.000
AMK58_25195	no KO assigned | (GenBank) pseudogene	Pseudogene	5.717	14.065	9.913	113.981	26.463	199.591
AMK58_25200	no KO assigned | (GenBank) glycosyl transferase	Glycosyl Transferase	3.048	5.023	7.395	171.900	16.894	67.050
AMK58_25205	no KO assigned | (GenBank) acyltransferase	Acyltransferase	3.548	6.183	5.482	152.284	15.121	22.893
AMK58_25210	no KO assigned | (GenBank) phytoene desaturase	No COG	19.630	19.233	23.517	190.269	63.192	120.788
AMK58_25215	K10027 phytoene desaturase [EC:1.3.99.26 1.3.99.28 1.3.99.29 1.3.99.31] | (GenBank) phytoene desatur	Metabolism	20.098	21.218	26.708	208.355	190.893	162.866
AMK58_25220	no KO assigned | (GenBank) aldehyde dehydrogenase	dehydrogenase	4.805	5.741	8.040	153.562	68.587	27.978
AMK58_25225	no KO assigned | (GenBank) hypothetical protein	Hypothetical	6.147	4.790	12.706	232.001	79.913	113.013
AMK58_25230	K14218 tRNA Ala | (GenBank) tRNA-Ala	tRNA synthesis/modification	79.654	76.143	72.707	207.000	82.372	515.554
AMK58_25235	K14228 tRNA Leu | (GenBank) tRNA-Leu	tRNA synthesis/modification	50.100	133.031	23.818	65.247	70.393	238.734
AMK58_25240	no KO assigned | (GenBank) hypothetical protein	Hypothetical	0.844	1.576	3.110	214.976	103.122	101.459
AMK58_25245	K00134 glyceraldehyde 3-phosphate dehydrogenase [EC:1.2.1.12] | (GenBank) gapA; glyceraldehyde-3-ph	Metabolism	3.906	4.243	6.639	63.257	46.163	74.627
AMK58_25250	no KO assigned | (GenBank) hypothetical protein	Hypothetical	5.281	2.253	8.248	188.459	37.733	114.543
AMK58_25255	K02410 flagellar motor switch protein FliG | (GenBank) flagellar motor switch protein FliG	Motility	6.983	6.364	5.401	154.417	34.589	190.738
AMK58_25260	no KO assigned | (GenBank) hypothetical protein	Hypothetical	0.747	4.465	2.487	174.200	16.802	99.795
AMK58_25265	K02419 flagellar biosynthetic protein FliP | (GenBank) flagellar biosynthetic protein flip	Motility	9.469	7.419	7.822	225.397	40.653	176.609
AMK58_25270	K02417 flagellar motor switch protein FliN/FliY | (GenBank) flagellar motor switch protein FliN	Motility	7.788	6.345	8.752	109.547	29.049	135.249
AMK58_25275	K02411 flagellar assembly protein FliH | (GenBank) hypothetical protein	Motility	3.143	4.941	2.202	129.173	7.437	32.822
AMK58_25280	K02409 flagellar M-ring protein FliF | (GenBank) flagellar M-ring protein FliF	Motility	3.986	1.276	5.346	163.807	14.561	116.032
AMK58_25285	K02415 flagellar FliL protein | (GenBank) flagellar basal body protein FliL	Motility	7.975	7.219	5.974	160.244	14.397	121.233
AMK58_25290	K02416 flagellar motor switch protein FliM | (GenBank) fliM; flagellar motor switch protein FliM	Motility	10.062	4.574	6.447	102.996	25.694	209.631
AMK58_25295	no KO assigned | (GenBank) hypothetical protein	Hypothetical	4.791	3.303	3.855	90.597	10.150	35.885
AMK58_25300	no KO assigned | (GenBank) flagellar motor switch protein	Motility	4.354	2.001	3.821	72.232	10.729	45.498
AMK58_25305	no KO assigned | (GenBank) hypothetical protein	Hypothetical	1.581	2.778	2.122	91.557	5.331	27.852
AMK58_25310	K02389 flagellar basal-body rod modification protein FlgD | (GenBank) flagellar biosynthesis protein	Motility	13.019	12.223	9.903	72.996	16.306	186.426
AMK58_25315	no KO assigned | (GenBank) hypothetical protein	Hypothetical	18.516	20.545	18.600	162.099	15.334	141.127
AMK58_25320	K02569 cytochrome c-type protein NapC | (GenBank) cytochrome C	Energy	173.911	139.779	189.084	88.536	33.749	258.347
AMK58_25325	K02568 cytochrome c-type protein NapB | (GenBank) nitrate reductase	Energy	99.266	71.886	99.150	202.816	30.994	144.812
AMK58_25330	K02567 periplasmic nitrate reductase NapA [EC:1.7.99.4] | (GenBank) nitrate reductase	Nitrogen	104.633	72.883	97.835	158.310	50.730	195.656
AMK58_25335	K02570 periplasmic nitrate reductase NapD | (GenBank) nitrate reductase	Nitrogen	55.596	41.827	48.162	193.630	13.887	218.429
AMK58_25340	K02571 periplasmic nitrate reductase NapE | (GenBank) nitrate reductase	Nitrogen	35.570	36.781	33.820	84.300	22.298	258.791
AMK58_25345	no KO assigned | (GenBank) hypothetical protein	Hypothetical	2.446	4.384	4.419	243.115	15.121	143.342
AMK58_25350	K02393 flagellar L-ring protein precursor FlgH | (GenBank) flagellar biosynthesis protein FlgH	Motility	6.509	9.723	4.797	231.725	16.829	219.409
AMK58_25355	K02386 flagella basal body P-ring formation protein FlgA | (GenBank) flagellin biosynthesis protein	Motility	6.536	0.976	6.370	230.475	17.081	102.876
AMK58_25360	K02392 flagellar basal-body rod protein FlgG | (GenBank) flgG; flagellar basal-body rod protein FlgG	Motility	15.250	7.362	8.934	156.408	20.431	168.833
AMK58_25365	K02391 flagellar basal-body rod protein FlgF | (GenBank) flagellar biosynthesis protein FlgG	Motility	7.806	3.969	11.212	191.108	21.469	214.067
AMK58_25370	no KO assigned | (GenBank) hypothetical protein	Hypothetical	0.757	0.603	2.302	126.843	4.480	56.649
AMK58_25375	K02556 chemotaxis protein MotA | (GenBank) flagellar motor stator protein MotA	Chemotaxis	5.863	6.517	6.482	197.253	16.092	168.910
AMK58_25380	K02557 chemotaxis protein MotB | (GenBank) chemotaxis protein	Chemotaxis	2.674	1.521	4.237	176.170	17.173	116.992
AMK58_25385	no KO assigned | (GenBank) hypothetical protein	Hypothetical	15.122	14.958	26.090	240.931	89.040	122.392
AMK58_25390	K09965 uncharacterized protein | (GenBank) hypothetical protein	Hypothetical	31.560	30.854	34.918	193.188	93.094	266.219
AMK58_25395	no KO assigned | (GenBank) hypothetical protein	Hypothetical	11.429	14.937	11.546	71.285	47.372	319.257
AMK58_25400	no KO assigned | (GenBank) hypothetical protein	Hypothetical	27.802	30.810	28.140	100.015	26.715	267.160
AMK58_25405	no KO assigned | (GenBank) glycerol-3-phosphate dehydrogenase	dehydrongenase	12.898	9.943	13.055	166.087	27.593	297.071
AMK58_25410	no KO assigned | (GenBank) glycerol-3-phosphate dehydrogenase	dehydrogenase	23.767	18.754	25.582	124.943	47.885	295.228
AMK58_25415	no KO assigned | (GenBank) glycerol-3-phosphate dehydrogenase	dehydrogenase	15.418	16.255	20.264	269.095	27.015	279.623
AMK58_25420	no KO assigned | (GenBank) glycerol-3-phosphate dehydrogenase	dehydrogenase	17.296	10.599	14.337	200.802	38.385	189.172
AMK58_25425	no KO assigned | (GenBank) hypothetical protein	Hypothetical	4.657	0.000	3.936	92.876	29.661	171.283
AMK58_25430	no KO assigned | (GenBank) pseudogene	Pseudogene	1.899	0.000	4.966	250.593	6.938	150.133
AMK58_25435	no KO assigned | (GenBank) hypothetical protein	Hypothetical	6.899	6.183	10.495	107.200	13.958	221.341
AMK58_25440	no KO assigned | (GenBank) hypothetical protein	Hypothetical	4.848	2.694	6.324	141.497	13.939	129.936
AMK58_25445	no KO assigned | (GenBank) hypothetical protein	Hypothetical	3.669	9.590	7.195	209.797	10.310	53.158
AMK58_25450	no KO assigned | (GenBank) hypothetical protein	Hypothetical	1.155	1.841	4.247	204.796	8.398	103.414
AMK58_25455	K04072 acetaldehyde dehydrogenase / alcohol dehydrogenase [EC:1.2.1.10 1.1.1.1] | (GenBank) acetalde	Metabolism	32.059	30.787	33.127	196.517	117.691	209.908

AMK58_25460	K14231 tRNA Phe | (GenBank) tRNA-Phe	tRNA synthesis/modification	25.489	38.071	24.236	234.743	89.535	426.920
AMK58_25465	no KO assigned | (GenBank) hypothetical protein	Hypothetical	7.160	15.556	12.997	126.435	105.362	475.637
AMK58_25470	no KO assigned | (GenBank) hypothetical protein	Hypothetical	14.304	9.156	17.972	112.230	32.157	412.129
AMK58_25475	no KO assigned | (GenBank) hypothetical protein	Hypothetical	60.764	46.056	40.960	140.318	136.093	975.106
AMK58_25480	no KO assigned | (GenBank) hypothetical protein	Hypothetical	18.048	14.976	17.637	166.551	53.817	408.866
AMK58_25485	no KO assigned | (GenBank) hypothetical protein	Hypothetical	13.305	9.274	12.650	64.161	80.759	276.356
AMK58_25490	no KO assigned | (GenBank) hypothetical protein	Hypothetical	18.022	15.165	16.411	36.041	76.463	195.589
AMK58_25495	no KO assigned | (GenBank) hypothetical protein	Hypothetical	14.927	26.424	15.507	124.417	153.493	414.219
AMK58_25500	K05554 aromatase [EC:4.2.1.-] | (GenBank) hypothetical protein	Metabolism	9.589	12.825	13.607	64.606	63.221	178.888
AMK58_25505	K12420 ketoreductase [EC:1.1.1.-] | (GenBank) ketoacyl reductase	Biosyntehsis	14.180	13.964	17.038	199.340	121.556	377.845
AMK58_25510	K05551 minimal PKS ketosynthase (KS/KS alpha) [EC:2.3.1.- 2.3.1.235] | (GenBank) hypothetical protei	Metabolism	4.165	6.083	5.369	124.739	47.757	116.905
AMK58_25515	no KO assigned | (GenBank) hypothetical protein	Hypothetical	3.698	3.156	6.730	20.804	13.301	50.943
AMK58_25520	K02078 acyl carrier protein | (GenBank) hypothetical protein	Lipid metabolism	205.777	279.817	217.921	266.305	208.900	443.535
AMK58_25525	no KO assigned | (GenBank) hypothetical protein	Hypothetical	13.753	5.388	9.432	107.822	64.879	268.862
AMK58_25530	no KO assigned | (GenBank) hypothetical protein	Hypothetical	11.677	12.240	13.051	162.460	66.320	254.174
AMK58_25535	no KO assigned | (GenBank) hypothetical protein	Hypothetical	13.144	15.485	9.857	89.525	59.099	162.911
AMK58_25540	no KO assigned | (GenBank) hypothetical protein	Hypothetical	8.690	6.922	6.977	131.146	27.566	220.600
AMK58_25545	no KO assigned | (GenBank) 3-ketoacyl-ACP reductase	No COG	5.526	10.005	7.643	124.051	33.506	231.061
AMK58_25550	K06133 4'-phosphopantetheinyl transferase [EC:2.7.8.-] | (GenBank) hypothetical protein	Metabolism: Co-Factors and Vitamins	0.761	3.412	3.258	346.048	36.591	215.007
AMK58_25555	no KO assigned | (GenBank) hypothetical protein	Hypothetical	59.337	61.475	69.245	375.064	206.132	650.189
AMK58_25560	no KO assigned | (GenBank) multidrug transporter AcrB	Defense	13.661	19.355	18.110	246.259	84.718	179.479
AMK58_25565	no KO assigned | (GenBank) helicase SNF2	DNA Repair and Replication	2.932	2.885	4.231	71.805	9.616	31.890
AMK58_25570	no KO assigned | (GenBank) LysR family transcriptional regulator	Transcription	853.478	883.047	872.136	472.023	168.964	171.411
AMK58_25575	K01652 acetolactate synthase I/II/III large subunit [EC:2.2.1.6] | (GenBank) thiamine pyrophosphate-	Biosynthesis: Amino Acid	515.600	529.422	542.733	180.239	73.137	119.123
AMK58_25580	K00128 aldehyde dehydrogenase (NAD+) [EC:1.2.1.3] | (GenBank) aldehyde dehydrogenase	Energy	16.433	17.807	14.629	36.149	63.564	64.538
AMK58_25585	no KO assigned | (GenBank) hypothetical protein	Hypothetical	195.676	142.479	202.912	582.395	211.701	1076.201
AMK58_25590	no KO assigned | (GenBank) hypothetical protein	Hypothetical	350.171	309.555	330.160	200.725	19.912	120.915
AMK58_25595	K09796 periplasmic copper chaperone A | (GenBank) hypothetical protein	Chaperone	306.150	322.443	280.191	254.635	25.769	83.261
AMK58_25600	no KO assigned | (GenBank) hypothetical protein	Hypothetical	4.355	12.143	12.975	497.059	162.529	664.195
AMK58_25605	no KO assigned | (GenBank) hypothetical protein	Hypothetical	2.613	2.168	4.762	96.553	78.572	200.039
AMK58_25610	K08482 circadian clock protein KaiC | (GenBank) circadian clock protein KaiC	No COG	2.281	3.760	4.039	125.723	5.217	78.786
AMK58_25615	no KO assigned | (GenBank) hypothetical protein	Hypothetical	231.550	293.483	291.286	979.122	105.621	604.253
AMK58_25620	no KO assigned | (GenBank) hypothetical protein	Hypothetical	8.795	7.663	8.319	67.808	20.339	87.972
AMK58_25625	no KO assigned | (GenBank) hypothetical protein	Hypothetical	15.786	18.863	16.054	180.574	22.836	158.730
AMK58_25630	no KO assigned | (GenBank) hypothetical protein	Hypothetical	43.174	37.383	32.721	113.362	54.156	304.027
AMK58_25635	no KO assigned | (GenBank) plasmid replication initiator protein	DNA Repair and Replication	7.166	9.014	8.966	196.543	30.808	106.674
AMK58_25640	no KO assigned | (GenBank) LysR family transcriptional regulator	Transcription	6.589	6.561	7.962	248.428	31.169	150.457
AMK58_25645	no KO assigned | (GenBank) hypothetical protein	Hypothetical	0.116	0.693	0.441	42.566	5.214	31.939
AMK58_25650	K10778 AraC family transcriptional regulator, regulatory protein of adaptative response / methylated	Transcription	3.145	0.000	2.990	86.359	19.442	151.151
AMK58_25655	no KO assigned | (GenBank) hypothetical protein	Hypothetical	154.821	147.123	146.121	189.980	151.471	249.065
AMK58_25660	no KO assigned | (GenBank) hypothetical protein	Hypothetical	3834.035	3916.517	4144.810	53.566	2562.464	122.398
AMK58_25665	no KO assigned | (GenBank) hypothetical protein	Hypothetical	244.596	204.585	354.078	1508.001	836.492	1166.357
AMK58_25670	no KO assigned | (GenBank) hypothetical protein	Hypothetical	2.089	3.285	3.450	71.444	4.944	66.179
AMK58_25675	no KO assigned | (GenBank) hypothetical protein	Hypothetical	276.051	218.614	259.913	543.503	179.643	803.851
AMK58_25680	no KO assigned | (GenBank) chromosome partitioning protein	Cell Cycle/Shape/Homeostatsis	54.206	49.496	54.127	122.392	29.001	110.953
AMK58_25685	K03497 chromosome partitioning protein, ParB family | (GenBank) chromosome partitioning protein	Cell Cycle/Shape/Homeostatsis	36.266	46.369	44.142	383.537	63.447	350.679
AMK58_25690	K03852 sulfoacetaldehyde acetyltransferase [EC:2.3.3.15] | (GenBank) sulfoacetaldehyde acetyltransfe	Metabolism: amino acids	2.045	2.444	3.047	67.943	10.192	41.343
AMK58_25695	no KO assigned | (GenBank) hypothetical protein	Hypothetical	9.843	21.563	11.855	450.956	71.550	162.167
AMK58_25700	no KO assigned | (GenBank) DMSO reductase	Energy	2.819	5.358	4.020	126.145	15.265	115.715
AMK58_25705	no KO assigned | (GenBank) ferredoxin	Energy	33.792	26.791	40.962	738.418	79.028	498.679
AMK58_25710	no KO assigned | (GenBank) formate dehydrogenase	Energy	15.034	14.422	17.475	45.836	6.093	38.405
AMK58_25715	K01902 succinyl-CoA synthetase alpha subunit [EC:6.2.1.5] | (GenBank) succinyl-CoA synthetase subuni	Metabolism: Carbon	42.889	50.134	54.710	175.746	17.218	129.561
AMK58_25720	K01903 succinyl-CoA synthetase beta subunit [EC:6.2.1.5] | (GenBank) succinate--CoA ligase	Metabolism: Carbon	63.712	55.297	68.764	257.047	18.872	218.651
AMK58_25725	K15515 sulfoacetaldehyde dehydrogenase [EC:1.2.1.81] | (GenBank) sulfoacetaldehyde dehydrogenase	dehydrogenase	65.625	55.214	64.573	194.896	20.204	251.506
AMK58_25730	no KO assigned | (GenBank) hypothetical protein	Hypothetical	20.454	25.851	24.909	76.298	5.306	82.287
AMK58_25735	K07794 putative tricarboxylic transport membrane protein | (GenBank) hypothetical protein	Signal transduction	154.218	145.479	172.142	449.409	25.850	426.499
AMK58_25740	no KO assigned | (GenBank) hypothetical protein	Hypothetical	13.086	16.075	15.000	160.548	20.895	55.795
AMK58_25745	K00625 phosphate acetyltransferase [EC:2.3.1.8] | (GenBank) phosphate acetyltransferase	Metabolism	23.270	34.756	33.994	189.947	18.800	85.298
AMK58_25750	no KO assigned | (GenBank) hypothetical protein	Hypothetical	11.927	12.429	14.110	250.246	25.099	167.054
AMK58_25755	no KO assigned | (GenBank) hypothetical protein	Hypothetical	168.484	247.393	183.591	70.743	5.228	205.758
AMK58_25760	K05800 Lrp/AsnC family transcriptional regulator | (GenBank) ArsR family transcriptional regulator	Transcription	33.462	70.199	38.859	224.460	10.336	94.979
AMK58_25765	no KO assigned | (GenBank) reactive intermediate/imine deaminase	No COG	107.622	181.796	151.665	662.044	54.005	616.592
AMK58_25770	K01738 cysteine synthase A [EC:2.5.1.47] | (GenBank) cysteine synthase	Metabolism	43.827	64.047	44.369	81.656	6.261	112.952
AMK58_25775	no KO assigned | (GenBank) hypothetical protein	Hypothetical	146.116	267.264	183.312	390.484	39.352	396.776
AMK58_25780	K01999 branched-chain amino acid transport system substrate-binding protein | (GenBank) amino acid A	Transport: Amino Acid	24.933	36.610	36.263	145.769	9.382	133.528
AMK58_25785	K01997 branched-chain amino acid transport system permease protein | (GenBank) branched-chain amino	Transport: Amino Acid	27.697	56.734	45.897	255.293	17.345	212.799
AMK58_25790	K01998 branched-chain amino acid transport system permease protein | (GenBank) branched-chain amino	Transport: Amino Acid	11.563	16.932	19.402	145.329	8.410	91.351

AMK58_25795	K01995 branched-chain amino acid transport system ATP-binding protein | (GenBank) ABC transporter A'	Transport: Amino Acid	11.351	27.314	22.334	203.577	7.974	134.636
AMK58_25800	K01996 branched-chain amino acid transport system ATP-binding protein | (GenBank) livF; ABC transpor	Transport: Amino Acid	27.015	19.683	23.024	86.499	9.628	71.639
AMK58_25805	no KO assigned | (GenBank) 2-amino-thiazoline-4-carboxylic acid hydrolase	hydrolase	77.595	101.243	89.890	310.931	31.329	221.438
AMK58_25810	K01451 hippurate hydrolase [EC:3.5.1.32] | (GenBank) peptidase M20	Metabolism: Amino Acid	11.300	16.276	16.596	231.791	15.764	55.386
AMK58_25815	no KO assigned | (GenBank) FAD-dependent oxidoreductase	Oxidoreductase	1.484	2.771	5.293	166.287	12.201	52.909
AMK58_25820	no KO assigned | (GenBank) MFS transporter	Transport: MFS	0.613	0.610	3.400	195.992	9.762	105.732
AMK58_25825	no KO assigned | (GenBank) serine hydrolase	hydrolase	5.881	9.087	9.063	181.067	26.444	128.537
AMK58_25830	no KO assigned | (GenBank) LysR family transcriptional regulator	Transcription	25.840	27.056	32.927	414.475	77.254	148.211
AMK58_25835	no KO assigned | (GenBank) hypothetical protein	Hypothetical	36.933	39.902	39.458	98.642	28.040	98.507
AMK58_25840	no KO assigned | (GenBank) hypothetical protein	Hypothetical	93.226	94.238	101.880	54.791	29.713	77.267
AMK58_25845	no KO assigned | (GenBank) hypothetical protein	Hypothetical	40.358	44.652	49.743	478.548	39.204	338.195
AMK58_25850	no KO assigned | (GenBank) hypothetical protein	Hypothetical	20.179	21.469	26.546	90.720	18.954	147.899
AMK58_25855	no KO assigned | (GenBank) hypothetical protein	Hypothetical	3805.121	4682.018	5060.079	703.612	5757.893	1126.048
AMK58_25860	K07684 two-component system, NarL family, nitrate/nitrite response regulator NarL | (GenBank) two-co	Nitrogen	181.701	248.554	273.851	383.197	695.254	397.313
AMK58_25865	no KO assigned | (GenBank) hypothetical protein	Hypothetical	1.207	3.155	2.080	50.929	36.122	89.256
AMK58_25870	no KO assigned | (GenBank) hypothetical protein	Hypothetical	45.244	41.878	55.338	611.754	92.161	333.855
AMK58_25875	K02769 PTS system, fructose-specific IIB component [EC:2.7.1.202] K02770 PTS system, fructose-speci	Metabolism: Carbohydrate	2.374	2.086	2.257	55.371	7.692	9.841
AMK58_25880	K00882 1-phosphofructokinase [EC:2.7.1.56] | (GenBank) 1-phosphofructokinase	Metabolism: Carbohydrate	23.259	24.037	31.320	431.148	69.672	214.630
AMK58_25885	K02768 PTS system, fructose-specific IIA component [EC:2.7.1.202] K08483 phosphotransferase system,	Metabolism	10.578	14.518	14.769	77.614	18.692	56.996
AMK58_25890	K03435 LacI family transcriptional regulator, fructose operon transcriptional repressor | (GenBank)	Transcription	21.179	26.010	26.179	87.004	61.500	77.029
AMK58_25895	no KO assigned | (GenBank) TetR family transcriptional regulator	Transcription	13.136	11.347	17.907	164.518	42.874	154.976
AMK58_25900	K13995 maleamate amidohydrolase [EC:3.5.1.107] | (GenBank) N-carbamoylsarcosine amidase	Metabolism: Co-Factors and Vitamens	8.859	11.762	11.793	309.076	104.449	331.332
AMK58_25905	K18028 2,5-dihydroxypyridine 5,6-dioxygenase [EC:1.13.11.9] | (GenBank) 2,5-dihydroxypyridine 5,6-di	Metabolism: Co-Factors and Vitamins	0.922	3.445	3.179	127.895	33.570	108.634
AMK58_25910	K15357 N-formylmaleamate deformylase [EC:3.5.1.106] | (GenBank) alpha/beta hydrolase	Metabolism: Co-Factors and Vitamins	1.794	3.572	4.406	166.591	36.460	139.436
AMK58_25915	K01799 maleate isomerase [EC:5.2.1.1] | (GenBank) Asp/Glu racemase	Metabolism: Carbohydrate	2.883	4.784	8.984	335.956	51.125	247.558
AMK58_25920	K00480 salicylate hydroxylase [EC:1.14.13.1] | (GenBank) salicylate 1-monooxygenase	Metabolism	0.899	0.000	1.900	116.688	6.400	90.409
AMK58_25925	K01996 branched-chain amino acid transport system ATP-binding protein | (GenBank) ABC transporter A'	Transport: Amino Acid	1.741	4.728	6.922	218.580	25.795	114.178
AMK58_25930	K01995 branched-chain amino acid transport system ATP-binding protein | (GenBank) ABC transporter A'	Transport: Amino Acid	4.914	2.669	7.929	153.807	29.964	214.320
AMK58_25935	K01998 branched-chain amino acid transport system permease protein | (GenBank) branched-chain amino	Transport: Amino Acid	4.858	5.209	8.527	124.810	25.062	149.577
AMK58_25940	K01997 branched-chain amino acid transport system permease protein | (GenBank) branched-chain amino	Transport: Amino Acid	13.314	13.257	18.461	268.080	56.900	242.158
AMK58_25945	K01999 branched-chain amino acid transport system substrate-binding protein | (GenBank) branched-cha	Transport: Amino Acid	0.958	0.000	1.417	45.646	2.155	22.118
AMK58_25950	no KO assigned | (GenBank) hypothetical protein	Hypothetical	0.310	0.000	2.066	193.375	6.630	115.148
AMK58_25955	no KO assigned | (GenBank) hypothetical protein	Hypothetical	4.340	20.741	19.805	944.631	66.339	1190.300
AMK58_25960	K01465 dihydroorotase [EC:3.5.2.3] | (GenBank) dihydroorotase	Metabolism: Nucleotides	0.666	1.137	1.629	126.740	2.140	52.693
AMK58_25965	no KO assigned | (GenBank) hypothetical protein	Hypothetical	1.316	1.747	2.920	282.454	7.561	119.998
AMK58_25970	K00681 gamma-glutamyltranspeptidase / glutathione hydrolase [EC:2.3.2.2 3.4.19.13] | (GenBank) gamma	Metabolism: amino acids	3.350	3.185	5.068	100.270	10.186	161.344
AMK58_25975	K01999 branched-chain amino acid transport system substrate-binding protein | (GenBank) ABC transpor	Transport: Amino Acid	1.902	2.525	5.826	160.771	8.788	162.233
AMK58_25980	K01997 branched-chain amino acid transport system permease protein | (GenBank) ABC transporter perme	Transport: Amino Acid	3.745	1.657	5.407	172.775	16.369	192.003
AMK58_25985	K01998 branched-chain amino acid transport system permease protein | (GenBank) ABC transporter perme	Transport: Amino Acid	2.216	2.091	4.880	143.905	14.422	87.393
AMK58_25990	K01995 branched-chain amino acid transport system ATP-binding protein | (GenBank) ABC transporter A'	Transport: Amino Acid	3.104	4.416	4.217	166.741	18.113	141.879
AMK58_25995	K01996 branched-chain amino acid transport system ATP-binding protein | (GenBank) ABC transporter A'	Transport: Amino Acid	8.415	15.391	16.492	626.429	63.896	499.879
AMK58_26000	K03520 carbon-monoxide dehydrogenase large subunit [EC:1.2.7.4] | (GenBank) carbon monoxide dehydr	Metabolism	0.766	1.831	2.137	56.320	4.307	26.149
AMK58_26005	K03519 carbon-monoxide dehydrogenase medium subunit [EC:1.2.7.4] | (GenBank) carbon monoxide deh	Metabolism	0.149	0.000	1.982	109.520	4.185	54.547
AMK58_26010	K03518 carbon-monoxide dehydrogenase small subunit [EC:1.2.7.4] | (GenBank) (2Fe-2S)-binding protei	Metabolism	5.447	5.917	8.946	394.354	35.345	255.534
AMK58_26015	no KO assigned | (GenBank) hypothetical protein	Hypothetical	5.605	4.465	8.527	273.814	22.262	86.801
AMK58_26020	K01426 amidase [EC:3.5.1.4] | (GenBank) amidase	Metabolism: Amino Acid	2.151	2.448	2.824	95.500	6.102	56.325
AMK58_26025	no KO assigned | (GenBank) hypothetical protein	Hypothetical	37.498	36.073	35.351	372.474	56.438	285.536
AMK58_26030	no KO assigned | (GenBank) MarR family transcriptional regulator	Transcription	12.955	22.326	19.186	350.068	39.204	115.504
AMK58_26035	no KO assigned | (GenBank) hypothetical protein	Hypothetical	7.164	11.453	6.811	166.376	9.753	46.592
AMK58_26040	K03522 electron transfer flavoprotein alpha subunit | (GenBank) electron transfer flavoprotein subun	energy	3.844	6.889	4.873	72.769	4.176	25.186
AMK58_26045	K03521 electron transfer flavoprotein beta subunit | (GenBank) electron transfer flavoprotein subuni	energy	28.646	28.367	24.830	300.203	43.408	286.324
AMK58_26050	no KO assigned | (GenBank) hydroxyacid dehydrogenase	dehydrogenase	14.913	15.101	14.740	157.333	25.950	145.474
AMK58_26055	K00128 aldehyde dehydrogenase (NAD+) [EC:1.2.1.3] | (GenBank) aldehyde dehydrogenase	Energy	8.488	10.937	9.732	144.462	9.260	70.217
AMK58_26060	K00077 2-dehydropantoate 2-reductase [EC:1.1.1.169] | (GenBank) hypothetical protein	Metabolism: Co-Factors and Vitamens	46.264	31.610	43.404	304.099	39.279	258.882
AMK58_26065	no KO assigned | (GenBank) C4-dicarboxylate ABC transporter	Membrane Transport	17.509	10.501	15.426	109.301	8.815	78.420
AMK58_26070	no KO assigned | (GenBank) C4-dicarboxylate ABC transporter	Membrane Transport	264.181	258.591	243.585	316.276	100.591	485.598
AMK58_26075	no KO assigned | (GenBank) ABC transporter substrate-binding protein	Membrane Transport	8.250	5.995	10.388	420.100	34.963	192.802
AMK58_26080	K00164 2-oxoglutarate dehydrogenase E1 component [EC:1.2.4.2] | (GenBank) hypothetical protein	Metabolism	6.017	7.466	6.865	97.832	18.520	71.756
AMK58_26085	K14448 (2S)-methylsuccinyl-CoA dehydrogenase [EC:1.3.8.12] | (GenBank) acyl-CoA dehydrogenase	Metabolism: Carbon	26.380	31.692	31.830	100.538	32.472	117.321
AMK58_26090	K19338 LysR family transcriptional regulator, nitrogen assimilation regulatory protein | (GenBank) h	Nitrogen	124.228	110.010	127.950	132.283	72.583	162.206
AMK58_26095	K00059 3-oxoacyl-[acyl-carrier protein] reductase [EC:1.1.1.100] | (GenBank) fabG; 3-ketoacyl-ACP re	Metabolism: Lipid	115.447	112.394	133.770	312.095	113.863	242.878
AMK58_26100	K14470 2-methylfumaryl-CoA isomerase [EC:5.4.1.3] | (GenBank) mesaconyl-CoA isomerase	Metabolism: Carbon	10.742	13.196	12.314	94.272	81.363	111.897
AMK58_26105	K02164 nitric oxide reductase NorE protein | (GenBank) copper oxidase	Nitrogen	15.059	16.794	16.800	51.103	48.524	78.756
AMK58_26110	no KO assigned | (GenBank) hypothetical protein	Hypothetical	721.623	712.872	665.271	722.197	366.367	743.990
AMK58_26115	K08289 phosphoribosylglycinamide formyltransferase 2 [EC:2.1.2.2] | (GenBank) purT; phosphoribosylgl	Metabolism	0.517	1.237	1.476	146.522	35.128	70.624
AMK58_26120	no KO assigned | (GenBank) hypothetical protein	Hypothetical	89.955	113.297	95.701	467.343	347.065	310.432
AMK58_26125	K02016 iron complex transport system substrate-binding protein | (GenBank) ABC transporter substrate	Transport: Metal	16.073	28.738	33.019	213.262	142.423	51.022

AMK58_26130	K02015 iron complex transport system permease protein | (GenBank) iron ABC transporter permease	Transport: Metal	5.835	5.810	7.397	173.910	116.690	26.827
AMK58_26135	K02013 iron complex transport system ATP-binding protein [EC:3.6.3.34] | (GenBank) ABC transporter	Transport: Metal	25.641	25.381	34.766	256.083	317.380	94.121
AMK58_26140	K17247 methionine sulfoxide reductase heme-binding subunit | (GenBank) sulfite oxidase	No COG	151.484	135.699	161.523	158.163	1348.905	254.130
AMK58_26145	K07147 methionine sulfoxide reductase catalytic subunit [EC:1.8.-.-] | (GenBank) sulfoxide reductase	Oxidoreductase	19.413	20.602	29.387	173.991	370.237	77.926
AMK58_26150	no KO assigned | (GenBank) oxidoreductase	Oxidoreductase	28.121	30.228	39.380	90.629	424.151	37.423
AMK58_26155	K01625 2-dehydro-3-deoxyphosphogluconate aldolase / (4S)-4-hydroxy-2-oxoglutarate aldolase [EC:4.1.2	Metabolism: Carbon	26.271	35.258	36.020	358.033	1325.197	206.534
AMK58_26160	no KO assigned | (GenBank) GntR family transcriptional regulator	Transcription	69.276	75.943	85.508	177.298	2625.241	181.665
AMK58_26165	K00874 2-dehydro-3-deoxygluconokinase [EC:2.7.1.45] | (GenBank) ketodeoxygluconokinase	Metabolism: Carbohydrate	123.021	119.396	120.301	182.698	85.332	261.000
AMK58_26170	no KO assigned | (GenBank) multidrug DMT transporter permease	defense	10.121	14.361	12.991	162.016	22.184	82.706
AMK58_26175	no KO assigned | (GenBank) hypothetical protein	Hypothetical	41.380	30.521	41.287	270.652	37.325	131.218
AMK58_26180	K13584 two-component system, cell cycle response regulator CtrA | (GenBank) two-component system res	Cell Cycle/Shape/Homeostatsis	373.428	146.491	180.570	113.632	169.988	353.755
AMK58_26185	K02406 flagellin | (GenBank) flagellin	Motility	5.154	2.905	2.681	47.809	2.405	34.988
AMK58_26190	K06602 flagellar protein FlaF | (GenBank) flagellar basal body protein FliL	Motility	13.247	0.000	8.202	60.252	15.929	82.845
AMK58_26195	K06601 flagellar protein FlbT | (GenBank) flagellar protein	Motility	4.423	8.257	4.731	119.973	8.079	83.561
AMK58_26200	no KO assigned | (GenBank) hypothetical protein	Hypothetical	26.495	11.044	15.232	263.294	55.753	385.374
AMK58_26205	no KO assigned | (GenBank) flagellar biosynthesis protein FlgG	Motility	8.072	14.558	15.639	238.077	39.202	247.699
AMK58_26210	no KO assigned | (GenBank) hypothetical protein	Hypothetical	13.921	13.978	17.240	191.802	21.959	112.812
AMK58_26215	no KO assigned | (GenBank) MFS transporter	Transport: MFS	3.081	2.454	5.370	114.177	24.241	34.090
AMK58_26220	no KO assigned | (GenBank) LysR family transcriptional regulator	Transcription	0.555	1.657	3.297	146.209	17.148	28.807
AMK58_26225	no KO assigned | (GenBank) hypothetical protein	Hypothetical	2.198	1.194	6.079	405.197	41.771	226.392
AMK58_26230	K02412 flagellum-specific ATP synthase [EC:3.6.3.14] | (GenBank) flagellar protein export ATPase Fli	Motility	1.174	2.697	1.459	52.732	4.059	73.507
AMK58_26235	K02387 flagellar basal-body rod protein FlgB | (GenBank) flagellar biosynthesis protein FlgG	Motility	7.086	5.522	3.515	72.220	17.315	149.693
AMK58_26240	K02388 flagellar basal-body rod protein FlgC | (GenBank) flagellar basal-body rod protein FlgC	Motility	2.306	3.445	3.015	108.124	10.693	124.833
AMK58_26245	K02408 flagellar hook-basal body complex protein FliE | (GenBank) flagellar basal body protein FliE	Motility	3.308	5.929	7.234	85.524	28.632	119.018
AMK58_26250	K02420 flagellar biosynthetic protein FliQ | (GenBank) flagellar biosynthetic protein FliQ	Motility	9.069	13.546	20.264	635.987	68.301	309.898
AMK58_26255	no KO assigned | (GenBank) transglycosylase	transglycosylase	17.745	9.382	16.872	447.431	39.010	429.275
AMK58_26260	K02400 flagellar biosynthesis protein FlhA | (GenBank) flhA; flagellar biosynthesis protein FlhA	Motility	1.156	0.345	1.979	84.811	3.900	49.004
AMK58_26265	K02421 flagellar biosynthetic protein FliR | (GenBank) flagellar biosynthesis protein	Motility	2.393	7.624	5.309	238.471	15.420	192.737
AMK58_26270	K02401 flagellar biosynthetic protein FlhB | (GenBank) flagellar biosynthetic protein FlhB	Motility	8.601	8.564	11.428	172.821	8.428	111.452
AMK58_26275	no KO assigned | (GenBank) hypothetical protein	Hypothetical	2.018	1.340	4.050	130.049	11.341	108.527
AMK58_26280	no KO assigned | (GenBank) AsnC family transcriptional regulator	Transcription	4252.293	4019.766	3646.238	359.531	653.184	473.417
AMK58_26285	no KO assigned | (GenBank) saccharopine dehydrogenase	Oxidoreductase	327.052	326.408	323.313	355.429	305.486	291.523
AMK58_26290	K00156 pyruvate dehydrogenase (quinone) [EC:1.2.5.1] | (GenBank) pyruvate oxidase	Metabolism: Carbohydrates	6.071	4.534	7.543	78.644	26.366	120.842
AMK58_26295	no KO assigned | (GenBank) ferredoxin	Ferredoxin	27.036	28.872	26.079	44.264	34.794	118.810
AMK58_26300	no KO assigned | (GenBank) PAS sensor protein	Signal transduction	22.169	23.772	25.402	63.961	454.602	174.993
AMK58_26305	no KO assigned | (GenBank) hypothetical protein	Hypothetical	259.830	360.530	358.148	629.693	897.351	816.539
AMK58_26310	K03797 carboxyl-terminal processing protease [EC:3.4.21.102] | (GenBank) hypothetical protein	Peptidases	2.982	4.157	5.482	209.324	77.879	178.636
AMK58_26315	no KO assigned | (GenBank) LysR family transcriptional regulator	Transcription	25.271	24.788	21.398	174.480	85.500	155.172
AMK58_26320	no KO assigned | (GenBank) oxidoreductase	Oxidoreductase	108.888	86.992	89.838	102.400	134.670	154.829
AMK58_26325	K07220 uncharacterized protein | (GenBank) nuclease PIN	No COG	233.742	208.746	356.904	634.978	411.010	547.315
AMK58_26330	K07303 isoquinoline 1-oxidoreductase subunit beta [EC:1.3.99.16] | (GenBank) twin-arginine transloca	Oxidoreductase	27.162	35.806	35.341	23.569	45.673	46.295
AMK58_26335	K07302 isoquinoline 1-oxidoreductase subunit alpha [EC:1.3.99.16] | (GenBank) isoquinoline 1-oxidore	Oxidoreductase	32.181	34.671	46.900	328.259	156.255	345.859
AMK58_26340	no KO assigned | (GenBank) LysR family transcriptional regulator	Transcription	416.042	449.063	404.590	366.279	351.802	365.693
AMK58_26345	no KO assigned | (GenBank) hypothetical protein	Hypothetical	2.894	1.820	3.089	15.981	15.578	65.008
AMK58_26350	no KO assigned | (GenBank) acetyltransferase	Acetyltransferase	36.110	28.201	31.192	293.705	46.956	236.576
AMK58_26355	no KO assigned | (GenBank) hypothetical protein	Hypothetical	2.537	3.610	3.332	131.594	20.781	96.023
AMK58_26360	no KO assigned | (GenBank) hypothetical protein	Hypothetical	3.210	2.740	2.834	78.943	13.403	89.305
AMK58_26365	no KO assigned | (GenBank) flagellar biosynthesis protein FlgJ	Motility	15.827	9.456	23.701	919.583	101.403	477.696
AMK58_26370	K02394 flagellar P-ring protein precursor FlgI | (GenBank) flgI; flagellar biosynthesis protein FlgA	Motility	5.537	6.998	6.480	231.939	21.665	109.801
AMK58_26375	K02397 flagellar hook-associated protein 3 FlgL | (GenBank) flagellar hook protein	Motility	7.390	4.754	10.593	241.680	18.018	214.630
AMK58_26380	K02396 flagellar hook-associated protein 1 FlgK | (GenBank) flagellar hook protein	Motility	9.613	8.796	8.152	158.125	23.072	206.711
AMK58_26385	K02390 flagellar hook protein FlgE | (GenBank) flagellar hook protein	Motility	91.518	100.319	74.505	90.366	34.768	94.878
AMK58_26390	K03704 cold shock protein (beta-ribbon, CspA family) | (GenBank) DNA-binding protein	Stress Response	190.910	208.024	238.138	124.290	90.951	111.352
AMK58_26395	no KO assigned | (GenBank) hypothetical protein	Hypothetical	334.824	290.235	362.411	620.711	302.429	598.772
AMK58_26400	no KO assigned | (GenBank) hypothetical protein	Hypothetical	31.776	24.250	31.095	26.254	19.554	46.026
AMK58_26405	no KO assigned | (GenBank) hypothetical protein	Hypothetical	3617.999	2797.684	3358.200	1446.952	923.299	1497.479
AMK58_26410	no KO assigned | (GenBank) hypothetical protein	Hypothetical	12.706	10.367	11.868	62.385	29.068	55.953
AMK58_26415	no KO assigned | (GenBank) hypothetical protein	Hypothetical	14.822	17.070	13.923	278.204	117.626	226.195
AMK58_26420	no KO assigned | (GenBank) hypothetical protein	Hypothetical	2.263	2.817	3.138	97.135	48.756	145.933
AMK58_26425	no KO assigned | (GenBank) C4-dicarboxylate ABC transporter permease	Membrane Transport	14.574	9.377	13.004	163.387	262.609	263.002
AMK58_26430	no KO assigned | (GenBank) C4-dicarboxylate ABC transporter	Membrane Transport	3.153	0.753	5.276	211.855	21.123	98.938
AMK58_26435	no KO assigned | (GenBank) GntR family transcriptional regulator	Transcription	2.562	5.741	9.441	434.212	49.145	225.876
AMK58_26440	no KO assigned | (GenBank) multidrug transporter	Transport	6.769	7.630	12.751	244.819	35.028	209.499
AMK58_26445	no KO assigned | (GenBank) formate transporter FocA	Transport	8.969	12.608	11.537	202.997	26.092	178.433
AMK58_26450	K04069 pyruvate formate lyase activating enzyme [EC:1.97.1.4] | (GenBank) pyruvate formate lyase-act	Oxidoreductase	23.439	26.894	29.222	481.151	173.606	338.104
AMK58_26455	K00873 pyruvate kinase [EC:2.7.1.40] | (GenBank) pyruvate kinase	Metabolism	123.140	143.817	147.524	301.598	318.712	404.289
AMK58_26460	no KO assigned | (GenBank) two-component system sensor histidine kinase/response regulator	Signal Transduction	8.043	13.364	11.811	65.920	50.393	124.273

AMK58_26465	no KO assigned | (GenBank) hypothetical protein	Hypothetical	25.251	31.590	28.728	131.252	166.538	115.572
AMK58_26470	no KO assigned | (GenBank) TetR family transcriptional regulator	Transcription	121.611	113.091	112.912	193.283	285.234	214.935
AMK58_26475	no KO assigned | (GenBank) TetR family transcriptional regulator	Transcription	68.796	41.712	63.307	341.936	311.426	250.278
AMK58_26480	K03585 membrane fusion protein, multidrug efflux system | (GenBank) hemolysin D	defense	88.073	79.966	97.147	495.864	387.033	509.714
AMK58_26485	K18138 multidrug efflux pump | (GenBank) multidrug transporter	Defense	3.302	4.359	4.710	50.616	8.719	60.354
AMK58_26490	K19337 RpiR family transcriptional regulator, carbohydrate utilization regulator | (GenBank) transcr	Transcription	11.428	10.371	12.378	284.649	45.852	546.191
AMK58_26495	K00656 formate C-acetyltransferase [EC:2.3.1.54] | (GenBank) formate acetyltransferase	Metabolism: Carbohydrate	2.227	2.852	3.328	60.882	12.638	86.452
AMK58_26500	K01689 enolase [EC:4.2.1.11] | (GenBank) eno; enolase	Metabolism	12.695	11.320	9.638	228.558	103.082	141.611
AMK58_26505	no KO assigned | (GenBank) enoyl-CoA hydratase	hydratase	4.963	2.917	6.344	148.343	58.260	58.097
AMK58_26510	K00925 acetate kinase [EC:2.7.2.1] | (GenBank) acetate kinase	Metabolism	8.151	7.127	10.680	131.426	23.017	96.784
AMK58_26515	no KO assigned | (GenBank) alpha/beta hydrolase	hydrolase	56.104	50.233	60.176	266.214	77.841	160.892
AMK58_26520	K00065 2-deoxy-D-gluconate 3-dehydrogenase [EC:1.1.1.125] | (GenBank) 2-deoxy-D-gluconate 3-dehyd	Metabolism: Carbohydrate	90.054	62.407	81.561	198.863	81.834	191.372
AMK58_26525	K01815 4-deoxy-L-threo-5-hexosulose-uronate ketol-isomerase [EC:5.3.1.17] | (GenBank) 5-keto-4-deoxy	Metabolism: Carbohydrate	10.927	11.316	13.022	221.908	69.439	117.404
AMK58_26530	no KO assigned | (GenBank) GntR family transcriptional regulator	Transcription	4.020	2.882	4.739	242.956	74.101	285.371
AMK58_26535	K02526 2-keto-3-deoxygluconate permease | (GenBank) 2-keto-3-deoxygluconate permease	Transport	8.072	6.290	7.230	111.415	23.405	84.067
AMK58_26540	K13584 two-component system, cell cycle response regulator CtrA | (GenBank) two-component system res	Signal Transduction	81.493	137.252	103.902	226.021	50.599	123.353
AMK58_26545	no KO assigned | (GenBank) hypothetical protein	Hypothetical	43.167	41.511	49.337	31.289	44.201	30.980
AMK58_26550	no KO assigned | (GenBank) hypothetical protein	Hypothetical	526.602	572.293	585.766	767.289	573.340	295.328
AMK58_26555	no KO assigned | (GenBank) hypothetical protein	Hypothetical	220.023	251.694	247.573	300.186	383.608	163.973
AMK58_26560	no KO assigned | (GenBank) hypothetical protein	Hypothetical	411.074	423.762	440.140	116.703	274.088	80.001
AMK58_26565	K05595 multiple antibiotic resistance protein | (GenBank) hypothetical protein	Defense	2614.561	2537.175	2757.323	337.035	1125.323	179.025
AMK58_26570	K00263 leucine dehydrogenase [EC:1.4.1.9] | (GenBank) amino acid dehydrogenase	Metabolism: Amino Acid	67.912	46.448	65.984	113.484	566.348	64.033
AMK58_26575	K09945 uncharacterized protein | (GenBank) hypothetical protein	Hypothetical	4.748	3.546	11.098	365.978	43.585	342.129
AMK58_26580	K11744 AI-2 transport protein TqsA | (GenBank) hypothetical protein	No COG	9.439	13.612	12.791	106.671	124.508	44.666
AMK58_26585	no KO assigned | (GenBank) HAD family hydrolase	hydrolase	6.250	10.669	12.055	303.321	140.308	218.744
AMK58_26590	no KO assigned | (GenBank) lipid kinase	No COG	55.270	50.676	55.153	36.469	36.138	29.431
AMK58_26595	no KO assigned | (GenBank) GntR family transcriptional regulator	Transcription	191.965	171.928	238.914	290.994	47.534	183.374
AMK58_26600	K01577 oxalyl-CoA decarboxylase [EC:4.1.1.8] | (GenBank) oxalyl-CoA decarboxylase	Metabolism: Carbohydrate	87.810	98.752	102.612	170.028	88.871	183.221
AMK58_26605	K00122 formate dehydrogenase [EC:1.2.1.2] | (GenBank) NADH-quinone oxidoreductase subunit F	Energy	101.071	99.456	138.861	376.560	133.499	361.520
AMK58_26610	K00123 formate dehydrogenase major subunit [EC:1.2.1.2] | (GenBank) formate dehydrogenase	Metabolism	24.444	25.740	30.264	70.551	21.644	63.473
AMK58_26615	K13641 IclR family transcriptional regulator, acetate operon repressor | (GenBank) transcriptional r	Transcription	8.730	9.405	9.933	103.139	39.251	141.067
AMK58_26620	K13639 MerR family transcriptional regulator, redox-sensitive transcriptional activator SoxR | (GenB	Transcription	60.275	67.325	75.251	128.836	94.558	207.043
AMK58_26625	no KO assigned | (GenBank) hypothetical protein	Hypothetical	80.253	108.088	91.742	415.096	393.680	1048.280
AMK58_26630	no KO assigned | (GenBank) MarR family transcriptional regulator	Transcription	692.528	526.615	639.275	119.401	184.241	279.297
AMK58_26635	no KO assigned | (GenBank) formyl-coenzyme A transferase	transferase	51.198	51.462	51.992	54.192	41.122	106.975
AMK58_26640	no KO assigned | (GenBank) histidine kinase	Signal Transduction	699.920	807.663	880.163	669.537	282.972	300.171
AMK58_26645	K03406 methyl-accepting chemotaxis protein | (GenBank) chemotaxis protein	Chemotaxis	40.124	43.107	45.233	121.875	33.100	88.336
AMK58_26650	K01485 cytosine deaminase [EC:3.5.4.1] | (GenBank) cytosine deaminase	Metabolism: Nucleotide	20.461	20.187	20.079	136.035	24.581	98.867
AMK58_26655	K02057 simple sugar transport system permease protein | (GenBank) ABC transporter permease	Transport: Sugar	14.283	10.177	15.200	172.015	19.000	85.296
AMK58_26660	K02057 simple sugar transport system permease protein | (GenBank) ABC transporter permease	Transport: Sugar	45.662	47.535	42.320	290.853	42.124	187.437
AMK58_26665	K02056 simple sugar transport system ATP-binding protein [EC:3.6.3.17] | (GenBank) sugar ABC transpc	Transport: Sugar	253.770	245.810	230.089	109.491	79.785	134.112
AMK58_26670	no KO assigned | (GenBank) ABC transporter substrate-binding protein	Membrane Transport	72.051	103.539	86.452	98.266	34.157	98.621
AMK58_26675	K00761 uracil phosphoribosyltransferase [EC:2.4.2.9] | (GenBank) uracil phosphoribosyltransferase	Metabolism: Nucleotide	258.521	345.755	390.608	387.869	161.129	244.310
AMK58_26680	no KO assigned | (GenBank) uracil phosphoribosyltransferase	Transferase	394.591	442.559	375.667	175.736	134.026	185.695
AMK58_26685	no KO assigned | (GenBank) hypothetical protein	Hypothetical	47.740	60.136	64.168	52.007	33.727	58.668
AMK58_26690	no KO assigned | (GenBank) hypothetical protein	Hypothetical	131.164	183.460	167.256	133.980	234.218	285.149
AMK58_26695	no KO assigned | (GenBank) hypothetical protein	Hypothetical	97.884	120.559	130.010	697.157	435.363	929.436
AMK58_26700	K01179 endoglucanase [EC:3.2.1.4] | (GenBank) endoglucanase	Metabolism: Carbohydrate	14.506	12.580	13.292	59.311	53.517	23.213
AMK58_26705	no KO assigned | (GenBank) hypothetical protein	Hypothetical	76.598	83.653	99.718	153.727	119.120	220.211
AMK58_26710	no KO assigned | (GenBank) hypothetical protein	Hypothetical	468.383	546.762	473.987	70.052	125.551	173.675
AMK58_26715	no KO assigned | (GenBank) hypothetical protein	Hypothetical	201.791	199.018	238.156	363.846	160.936	459.177
AMK58_26720	no KO assigned | (GenBank) transglycosylase	transglycosylase	0.000	0.000	0.000	0.000	0.000	0.000
AMK58_26725	K01977 16S ribosomal RNA | (GenBank) 16S ribosomal RNA	Ribosome	0.000	0.000	0.000	0.000	0.000	0.000
AMK58_26730	K14227 tRNA Ile | (GenBank) tRNA-Ile	tRNA synthesis/modification	0.000	0.000	0.000	0.000	0.000	0.000
AMK58_26735	K14218 tRNA Ala | (GenBank) tRNA-Ala	tRNA synthesis/modification	0.000	0.000	0.000	0.000	0.000	0.000
AMK58_26740	K01980 23S ribosomal RNA | (GenBank) 23S ribosomal RNA	Ribosome	0.000	0.000	0.000	0.000	0.000	0.000
AMK58_26745	K01985 5S ribosomal RNA | (GenBank) 5S ribosomal RNA	Ribosome	46.969	37.415	32.749	51.732	127.881	191.633
AMK58_26750	K14230 tRNA Met | (GenBank) tRNA-Met	tRNA synthesis/modification	732.738	901.846	1079.427	271.714	364.093	886.493
AMK58_26755	no KO assigned | (GenBank) hypothetical protein	Hypothetical	223.923	320.843	434.172	362.302	398.036	380.872
AMK58_26760	K04564 superoxide dismutase, Fe-Mn family [EC:1.15.1.1] | (GenBank) superoxide dismutase	Defense	1123.117	1217.120	1461.384	228.309	262.427	149.842
AMK58_26765	no KO assigned | (GenBank) cobalt transporter	Transport: Metal	79.116	93.473	78.267	460.122	105.917	372.913
AMK58_26770	no KO assigned | (GenBank) hypothetical protein	Hypothetical	8.724	8.625	10.324	98.455	42.754	69.434
AMK58_26775	no KO assigned | (GenBank) glutathionylspermidine synthase	No COG	5.914	6.198	7.102	55.035	44.198	26.937
AMK58_26780	no KO assigned | (GenBank) hypothetical protein	Hypothetical	1852.713	1858.220	1456.127	437.541	4632.007	680.105
AMK58_26785	no KO assigned | (GenBank) polyhydroxyalkanoate synthesis repressor	Transcription	59.628	68.426	65.856	406.926	117.498	196.219
AMK58_26790	K03821 polyhydroxyalkanoate synthase [EC:2.3.1.-] | (GenBank) poly-beta-hydroxybutyrate polymerase	Metabolism: Carbohydrate (PHB)	1312.711	1460.042	1563.422	184.279	210.137	173.172
AMK58_26795	K00626 acetyl-CoA C-acetyltransferase [EC:2.3.1.9] | (GenBank) acetyl-CoA acetyltransferase	Metabolism	1836.713	2293.701	2231.800	74.887	74.990	160.290

AMK58_26800	K00023 acetoacetyl-CoA reductase [EC:1.1.1.36] | (GenBank) acetoacetyl-CoA reductase	Metabolism: Carbon	25.287	26.013	33.596	134.145	61.176	75.157
AMK58_26805	no KO assigned | (GenBank) hypothetical protein	Hypothetical	568.630	505.629	541.804	470.420	866.325	674.177
AMK58_26810	no KO assigned | (GenBank) MFS transporter	Transport: MFS	18.024	27.506	31.946	163.104	142.590	84.747
AMK58_26815	no KO assigned | (GenBank) transcriptional regulator	Transcription	15.781	16.229	15.743	129.783	137.547	58.894
AMK58_26820	no KO assigned | (GenBank) phosphohydrolase	hydrolase	51.365	56.365	62.045	309.277	975.924	383.710
AMK58_26825	no KO assigned | (GenBank) hypothetical protein	Hypothetical	315.923	255.810	256.920	314.102	205.684	431.105
AMK58_26830	K06942 ribosome-binding ATPase | (GenBank) GTP-binding protein	Ribosome	33.870	27.594	30.008	205.200	14.957	73.317
AMK58_26835	K03832 periplasmic protein TonB | (GenBank) hypothetical protein	Transport	603.123	417.700	380.877	348.489	95.451	478.572
AMK58_26840	K02014 iron complex outermembrane recepter protein | (GenBank) TonB-dependent receptor	Transport: Ion	63.946	62.704	69.082	52.540	7.297	35.316
AMK58_26845	no KO assigned | (GenBank) hypothetical protein	Hypothetical	822.958	822.948	867.285	1068.311	341.219	1394.422
AMK58_26850	K02335 DNA polymerase I [EC:2.7.7.7] | (GenBank) DNA polymerase I	DNA Repair and Replication	2.416	1.493	3.445	21.703	61.282	32.403
AMK58_26855	no KO assigned | (GenBank) hypothetical protein	Hypothetical	7.487	4.364	13.196	223.342	392.474	176.448
AMK58_26860	no KO assigned | (GenBank) hypothetical protein	Hypothetical	6.348	10.837	10.132	157.326	60.146	92.717
AMK58_26865	K14219 tRNA Arg | (GenBank) tRNA-Arg	tRNA synthesis/modification	718.587	798.509	885.070	2281.134	2193.398	2259.974
AMK58_26870	no KO assigned | (GenBank) alpha/beta hydrolase	Hydrolase	218.225	215.852	187.040	168.175	310.488	172.102
AMK58_26875	K01990 ABC-2 type transport system ATP-binding protein | (GenBank) multidrug ABC transporter ATP-l	Membrane Transport	224.717	309.602	228.421	34.708	243.196	95.662
AMK58_26880	no KO assigned | (GenBank) hypothetical protein	Hypothetical	889.867	1055.388	998.327	2041.947	359.197	2501.835
AMK58_26885	no KO assigned | (GenBank) diguanylate cyclase	cyclic nucleotide	10.442	7.009	9.816	53.276	8.901	79.198
AMK58_26890	no KO assigned | (GenBank) hypothetical protein	Hypothetical	3.245	12.117	11.570	540.620	51.747	274.374
AMK58_26895	no KO assigned | (GenBank) cytochrome C	Energy	2.669	2.552	5.583	183.782	39.844	133.258
AMK58_26900	K02275 cytochrome c oxidase subunit II [EC:1.9.3.1] | (GenBank) cytochrome C oxidase subunit II	Energy	6.136	6.345	12.903	261.299	102.667	461.881
AMK58_26905	K15408 cytochrome c oxidase subunit I+III [EC:1.9.3.1] | (GenBank) cytochrome B	Energy	0.430	0.571	0.364	9.352	2.096	7.449
AMK58_26910	no KO assigned | (GenBank) hypothetical protein	Hypothetical	32.959	38.177	44.449	500.073	156.507	250.424
AMK58_26915	no KO assigned | (GenBank) cytochrome C oxidase subunit I	Energy	3.915	3.599	4.009	142.417	27.083	60.184
AMK58_26920	K01512 acylphosphatase [EC:3.6.1.7] | (GenBank) acylphosphatase	Metabolism	36.240	36.906	47.770	911.330	276.815	793.907
AMK58_26925	no KO assigned | (GenBank) purine permease	Transport	44.832	45.278	45.231	139.925	30.996	81.874
AMK58_26930	K01754 threonine dehydratase [EC:4.3.1.19] | (GenBank) threonine dehydratase	Metabolism	10.017	14.525	12.668	114.768	10.057	45.088
AMK58_26935	no KO assigned | (GenBank) carbon-nitrogen hydrolase	hydrolase	123.175	108.459	105.989	205.395	33.560	140.650
AMK58_26940	K01480 agmatinase [EC:3.5.3.11] | (GenBank) agmatinase	Metabolism: Amino Acid	58.932	52.060	53.526	138.227	18.300	146.078
AMK58_26945	K02053 putative spermidine/putrescine transport system permease protein | (GenBank) spermidine/putre	Quorum sensing	94.018	88.500	82.351	152.467	22.088	194.264
AMK58_26950	K02054 putative spermidine/putrescine transport system permease protein | (GenBank) ABC transporter	Membrane Transport	131.695	128.597	106.367	167.308	28.970	119.360
AMK58_26955	K02052 putative spermidine/putrescine transport system ATP-binding protein | (GenBank) ABC transport	Membrane Transport	717.556	751.963	582.315	175.778	63.561	244.414
AMK58_26960	K02055 putative spermidine/putrescine transport system substrate-binding protein | (GenBank) ABC tra	Quorum sensing	13.381	14.183	14.775	86.008	33.235	128.480
AMK58_26965	no KO assigned | (GenBank) transcriptional regulator	Transcription	8.354	5.375	8.677	41.666	34.673	81.997
AMK58_26970	no KO assigned | (GenBank) hypothetical protein	Hypothetical	18.114	7.594	22.359	37.886	46.436	79.856
AMK58_26975	K07729 putative transcriptional regulator | (GenBank) hypothetical protein	Transcription	559.250	478.410	635.286	648.744	919.529	944.491
AMK58_26980	K00640 serine O-acetyltransferase [EC:2.3.1.30] | (GenBank) serine acetyltransferase	Metabolism	29.140	27.790	30.178	73.488	27.372	240.650
AMK58_26985	no KO assigned | (GenBank) glycosyl transferase	Glycosyl Transferase	45.583	52.745	60.814	598.784	48.605	456.597
AMK58_26990	no KO assigned | (GenBank) hypothetical protein	Hypothetical	22.324	30.846	36.091	277.810	33.022	176.058
AMK58_26995	no KO assigned | (GenBank) hypothetical protein	Hypothetical	4.611	6.190	6.946	162.880	44.549	90.459
AMK58_27000	K02769 PTS system, fructose-specific IIB component [EC:2.7.1.202] K02770 PTS system, fructose-speci	Metabolism: Carbohydrate	1.709	2.356	3.187	68.326	19.357	35.290
AMK58_27005	K00882 1-phosphofructokinase [EC:2.7.1.56] | (GenBank) hypothetical protein	Metabolism: Carbohydrate	23.293	31.256	34.583	600.188	316.431	250.874
AMK58_27010	K02768 PTS system, fructose-specific IIA component [EC:2.7.1.202] K08483 phosphotransferase system,	Metabolism: Carbohydrate	18.489	26.246	30.294	254.790	52.039	145.172
AMK58_27015	no KO assigned | (GenBank) hypothetical protein	Hypothetical	6.909	9.061	8.011	31.658	15.153	35.431
AMK58_27020	no KO assigned | (GenBank) two-component system response regulator	Signal Transduction	225.605	269.574	301.025	760.225	617.914	787.050
AMK58_27025	no KO assigned | (GenBank) ATPase	Enzyme	20.740	32.894	30.851	98.887	49.030	74.549
AMK58_27030	no KO assigned | (GenBank) Crp/Fnr family transcriptional regulator	Transcription	22.744	20.434	53.657	344.530	154.931	436.869
AMK58_27035	no KO assigned | (GenBank) cytochrome c oxidase accessory protein CcoG	Energy	21.366	23.965	20.549	69.634	100.115	145.138
AMK58_27040	no KO assigned | (GenBank) hypothetical protein	Hypothetical	38.517	43.148	55.876	161.237	249.106	248.963
AMK58_27045	no KO assigned | (GenBank) hypothetical protein	Hypothetical	642.429	501.115	603.726	290.318	717.597	294.192
AMK58_27050	K01624 fructose-bisphosphate aldolase, class II [EC:4.1.2.13] | (GenBank) fructose-bisphosphate aldo	Metabolism: Carbohydrate	4.821	1.340	6.715	128.097	89.847	80.876
AMK58_27055	no KO assigned | (GenBank) gluconolaconase	No COG	158.937	139.202	149.404	561.055	753.735	604.559
AMK58_27060	K01595 phosphoenolpyruvate carboxylase [EC:4.1.1.31] | (GenBank) phosphoenolpyruvate carboxylase	Metabolism	32.295	35.292	44.686	92.240	56.512	36.624
AMK58_27065	no KO assigned | (GenBank) GntR family transcriptional regulator	Transcription	26.339	22.843	30.214	222.849	151.469	195.940
AMK58_27070	no KO assigned | (GenBank) cytochrome c oxidase accessory protein CcoG	Energy	43.270	44.455	48.524	108.882	128.002	116.019
AMK58_27075	no KO assigned | (GenBank) hypothetical protein	Hypothetical	188.298	217.666	214.725	143.125	135.817	123.986
AMK58_27080	no KO assigned | (GenBank) 2-hydroxychromene-2-carboxylate isomerase	No COG	539.114	515.826	446.539	224.317	129.774	248.557
AMK58_27085	no KO assigned | (GenBank) two-component system response regulator	Signal Transduction	21.197	35.459	21.605	27.485	15.058	102.206
AMK58_27090	no KO assigned | (GenBank) hypothetical protein	Hypothetical	305.291	272.231	259.327	512.717	122.923	635.591
AMK58_27095	no KO assigned | (GenBank) hypothetical protein	Hypothetical	121.660	118.812	152.658	878.703	404.335	571.924
AMK58_27100	no KO assigned | (GenBank) hypothetical protein	Hypothetical	38.643	45.423	49.026	73.359	51.854	53.113
AMK58_27105	no KO assigned | (GenBank) NnrU protein	No COG	206.641	221.456	203.064	130.630	227.795	123.031
AMK58_27110	no KO assigned | (GenBank) hypothetical protein	Hypothetical	364.246	445.611	342.685	127.969	377.845	191.380
AMK58_27115	no KO assigned | (GenBank) thioesterase	enzyme	362.679	464.316	406.287	267.022	336.555	183.829
AMK58_27120	K03149 thiazole synthase [EC:2.8.1.10] | (GenBank) thiG; thiazole synthase	Metabolism: Co-Factors and Vitamins	24.341	26.270	38.672	22.508	28.242	8.446
AMK58_27125	K03154 sulfur carrier protein | (GenBank) thiamine biosynthesis protein ThiS	Genetic Information Processing	1379.676	1953.060	1805.723	851.090	1576.089	381.717
AMK58_27130	K03153 glycine oxidase [EC:1.4.3.19] | (GenBank) glycine oxidase	Metabolism: Co-Factors and Vitamins	49.672	39.568	53.722	176.325	187.274	305.911

AMK58_27135	K06213 magnesium transporter | (GenBank) magnesium transporter	Transport: Inorganic	31.446	27.126	32.457	14.304	20.225	42.335
AMK58_27140	K20978 HptB-dependent secretion and biofilm anti anti-sigma factor | (GenBank) hypothetical protein	Signal transduction	28.371	57.295	23.556	61.556	12.576	198.973
AMK58_27145	K14228 tRNA Leu | (GenBank) tRNA-Leu	tRNA synthesis/modification	460.641	424.036	506.788	1806.415	580.351	1699.531
AMK58_27150	K03801 lipoyl(octanoyl) transferase [EC:2.3.1.181] | (GenBank) lipoate--protein ligase	Metabolism: Co-Factors and Vitamens	66.385	59.049	64.373	96.208	65.732	105.549
AMK58_27155	no KO assigned | (GenBank) hypothetical protein	Hypothetical	433.967	383.850	396.962	529.124	243.508	464.564
AMK58_27160	K00128 aldehyde dehydrogenase (NAD+) [EC:1.2.1.3] | (GenBank) aldehyde dehydrogenase	Energy	877.708	920.771	860.434	198.002	197.430	267.810
AMK58_27165	K02035 peptide/nickel transport system substrate-binding protein | (GenBank) peptide ABC transporter	Quorum sensing	76.761	87.520	108.433	80.946	38.531	114.036
AMK58_27170	K02033 peptide/nickel transport system permease protein | (GenBank) ABC transporter permease	Quorum sensing	50.881	67.306	78.158	130.843	55.940	115.714
AMK58_27175	K02034 peptide/nickel transport system permease protein | (GenBank) peptide ABC transporter permease	Quorum sensing	46.794	71.294	63.360	181.943	53.804	138.255
AMK58_27180	no KO assigned | (GenBank) methionine ABC transporter ATP-binding protein	Membrane Transport	49.408	65.029	63.374	202.650	51.413	88.001
AMK58_27185	no KO assigned | (GenBank) peptide ABC transporter ATP-binding protein	Membrane Transport	141.734	177.968	174.848	145.613	147.434	179.542
AMK58_27190	K01438 acetylornithine deacetylase [EC:3.5.1.16] | (GenBank) acetylornithine deacetylase	Metabolism: Amino Acid	137.562	201.839	164.263	47.423	724.807	49.147
AMK58_27195	no KO assigned | (GenBank) hypothetical protein	Hypothetical	358.532	423.359	452.444	672.631	398.785	683.191
AMK58_27200	K13775 citronellol/citronellal dehydrogenase | (GenBank) short-chain dehydrogenase	Metabolism	13.891	23.588	26.555	188.825	75.772	126.642
AMK58_27205	no KO assigned | (GenBank) hypothetical protein	Hypothetical	28.364	38.734	38.065	296.798	135.182	152.248
AMK58_27210	K07335 basic membrane protein A and related proteins | (GenBank) ABC transporter substrate-binding p	Membrane	322.988	469.125	441.347	243.360	222.299	166.765
AMK58_27215	no KO assigned | (GenBank) hypothetical protein	Hypothetical	39.745	31.742	42.162	121.126	77.407	123.449
AMK58_27220	no KO assigned | (GenBank) isochorismatase	No COG	110.302	108.551	87.295	74.308	140.250	156.402
AMK58_27225	no KO assigned | (GenBank) hypothetical protein	Hypothetical	122.154	152.150	152.671	164.788	259.572	175.309
AMK58_27230	no KO assigned | (GenBank) hypothetical protein	Hypothetical	395.829	397.905	430.042	160.854	689.852	296.436
AMK58_27235	K00963 UTP--glucose-1-phosphate uridylyltransferase [EC:2.7.7.9] | (GenBank) UTP--glucose-1-phospha	Metabolism: Carbohydrate	41.611	56.538	42.740	138.510	154.083	164.017
AMK58_27240	no KO assigned | (GenBank) hypothetical protein	Hypothetical	36.178	50.913	38.526	219.909	369.241	159.086
AMK58_27245	no KO assigned | (GenBank) hypothetical protein	Hypothetical	35.658	34.861	37.911	285.942	57.024	134.663
AMK58_27250	K06177 tRNA pseudouridine32 synthase / 23S rRNA pseudouridine746 synthase [EC:5.4.99.28 5.4.99.29	tRNA synthesis/modification	61.985	73.251	74.317	190.243	93.791	91.111
AMK58_27255	K02058 simple sugar transport system substrate-binding protein | (GenBank) hypothetical protein	Transport: Sugar	4.925	2.870	5.253	92.839	33.483	43.660
AMK58_27260	no KO assigned | (GenBank) endonuclease III	DNA Repair and Replication	21.861	26.791	26.115	539.118	49.775	135.139
AMK58_27265	K00652 8-amino-7-oxononanoate synthase [EC:2.3.1.47] | (GenBank) 8-amino-7-oxononanoate synthase	Metabolism: Co-Factors and Vitamens	7.375	9.792	9.739	99.068	18.998	65.443
AMK58_27270	K02170 pimeloyl-[acyl-carrier protein] methyl ester esterase [EC:3.1.1.85] | (GenBank) hypothetical	Metabolism: Co-Factors and Vitamens	4.297	8.251	9.046	363.839	19.664	96.333
AMK58_27275	K01935 dethiobiotin synthetase [EC:6.3.3.3] K02169 malonyl-CoA O-methyltransferase [EC:2.1.1.197] |	Metabolism: Co-Factors and Vitamens	15.800	10.773	13.880	222.229	16.119	115.136
AMK58_27280	K00833 adenosylmethionine---8-amino-7-oxononanoate aminotransferase [EC:2.6.1.62] | (GenBank) adeno	Metabolism: Co-Factors and Vitamens	66.985	58.755	56.457	235.884	21.066	164.060
AMK58_27285	K01012 biotin synthase [EC:2.8.1.6] | (GenBank) biotin synthase	Metabolism: Co-Factors and Vitamens	46.333	53.177	48.227	78.071	119.510	90.675
AMK58_27290	no KO assigned | (GenBank) hypothetical protein	Hypothetical	3291.250	2517.441	2388.718	588.726	767.350	785.412
AMK58_27295	K01006 pyruvate, orthophosphate dikinase [EC:2.7.9.1] | (GenBank) pyruvate phosphate dikinase	Metabolism	4.135	5.907	5.769	91.267	6.820	27.922
AMK58_27300	no KO assigned | (GenBank) serine hydrolase	No COG	21.353	27.385	26.149	119.655	27.772	142.125
AMK58_27305	K03411 chemotaxis protein CheD [EC:3.5.1.44] | (GenBank) chemotaxis protein	Chemotaxis	34.332	42.550	32.643	107.145	29.969	261.455
AMK58_27310	K03704 cold shock protein (beta-ribbon, CspA family) | (GenBank) hypothetical protein	Stress Response	1139.781	967.183	1116.050	511.463	1002.349	774.324
AMK58_27315	K01897 long-chain acyl-CoA synthetase [EC:6.2.1.3] | (GenBank) dicarboxylate--CoA ligase PimA	Metabolism: Lipid	50.732	59.006	55.735	72.812	88.413	96.523
AMK58_27320	K03274 ADP-L-glycero-D-manno-heptose 6-epimerase [EC:5.1.3.20] | (GenBank) ADP-L-glycero-D-man	Biosynthesis: polysacchharide	64.401	50.569	63.799	139.345	84.800	153.447
AMK58_27325	K13292 phosphatidylglycerol:prolipoprotein diacylglycerol transferase [EC:2.-.-.-] | (GenBank) proli	Biosynthesis: polysacchharide	48.460	41.232	41.939	298.245	104.726	130.216
AMK58_27330	no KO assigned | (GenBank) ATP synthase subunit beta	No COG	54.596	55.003	57.000	108.984	86.156	106.711
AMK58_27335	K05810 laccase domain-containing protein | (GenBank) polyphenol oxidase	No COG	270.684	392.759	318.526	102.844	137.156	146.477
AMK58_27340	no KO assigned | (GenBank) hypothetical protein	Hypothetical	1338.168	1298.097	1319.886	341.372	1079.045	567.233
AMK58_27345	K00948 ribose-phosphate pyrophosphokinase [EC:2.7.6.1] | (GenBank) phosphoribosylpyrophosphate syn	Metabolism: Carbon	562.687	396.454	438.706	51.116	173.315	129.302
AMK58_27350	K02897 large subunit ribosomal protein L25 | (GenBank) 50S ribosomal protein L25	Ribosome	32.841	26.003	30.473	119.519	44.920	181.980
AMK58_27355	K01056 peptidyl-tRNA hydrolase, PTH1 family [EC:3.1.1.29] | (GenBank) peptidyl-tRNA hydrolase	tRNA synthesis/modification	73.780	90.419	89.937	264.115	32.133	295.877
AMK58_27360	K02057 simple sugar transport system permease protein | (GenBank) ABC transporter permease	Transport: Sugar	44.444	45.857	59.914	171.804	19.342	74.846
AMK58_27365	K02057 simple sugar transport system permease protein | (GenBank) sugar ABC transporter permease	Transport: Sugar	102.658	99.164	134.359	274.726	32.075	147.585
AMK58_27370	K02056 simple sugar transport system ATP-binding protein [EC:3.6.3.17] | (GenBank) sugar ABC transpo	Transport: Sugar	221.619	232.257	223.855	112.202	37.029	139.125
AMK58_27375	K02058 simple sugar transport system substrate-binding protein | (GenBank) sugar ABC transporter sub	Transport: Sugar	10.027	15.725	14.658	302.545	24.936	120.173
AMK58_27380	K13877 2,5-dioxopentanoate dehydrogenase [EC:1.2.1.26] | (GenBank) 2,5-dioxovalerate dehydrogenase	Metabolism: Carbohydrate	7.811	8.236	9.247	143.413	15.839	57.591
AMK58_27385	K01714 4-hydroxy-tetrahydrodipicolinate synthase [EC:4.3.3.7] | (GenBank) cytochrome C biogenesis pr	Biosynthesis: Amino Acid	8.490	13.265	14.405	215.898	21.875	115.419
AMK58_27390	no KO assigned | (GenBank) gluconolaconase	No COG	46.104	49.041	47.739	312.093	58.897	193.586
AMK58_27395	no KO assigned | (GenBank) transcriptional regulator	Transcription	112.838	108.960	118.587	111.749	109.642	235.995
AMK58_27400	K10546 putative multiple sugar transport system substrate-binding protein | (GenBank) sugar ABC tran	Transport: Sugar	40.920	40.298	45.321	215.796	81.883	277.703
AMK58_27405	K10548 putative multiple sugar transport system ATP-binding protein [EC:3.6.3.17] | (GenBank) ABC tr	Transport: Sugar	18.352	20.906	23.881	170.311	35.487	133.469
AMK58_27410	K10547 putative multiple sugar transport system permease protein | (GenBank) ABC transporter permeas	Transport: Sugar	6.993	8.356	9.213	217.051	28.858	91.428
AMK58_27415	K00035 D-galactose 1-dehydrogenase [EC:1.1.1.48] | (GenBank) galactose 1-dehydrogenase	Metabolism: Carbohydrate	33.673	39.200	45.435	391.866	157.523	374.577
AMK58_27420	K01687 dihydroxy-acid dehydratase [EC:4.2.1.9] | (GenBank) dihydroxy-acid dehydratase	Metabolism	7.754	7.523	12.350	101.644	49.089	131.135
AMK58_27425	no KO assigned | (GenBank) lysophospholipase	enzyme	5.094	8.779	8.476	211.918	30.830	179.759
AMK58_27430	K01785 aldose 1-epimerase [EC:5.1.3.3] | (GenBank) aldose epimerase	Metabolism: Carbohydrate	4.397	6.735	10.290	253.095	62.851	214.713
AMK58_27435	K00041 tagaturonate reductase [EC:1.1.1.58] | (GenBank) tagaturonate reductase	Metabolism: Carbohydrate	80.154	114.315	121.081	82.062	45.816	71.492
AMK58_27440	K02020 molybdate transport system substrate-binding protein | (GenBank) hypothetical protein	Transport	24.725	42.887	46.715	179.067	80.329	65.528
AMK58_27445	K02018 molybdate transport system permease protein | (GenBank) hypothetical protein	Transport	8.552	14.342	17.831	336.430	138.286	243.738
AMK58_27450	no KO assigned | (GenBank) hypothetical protein	Hypothetical	6.630	7.463	11.055	144.423	46.120	93.023
AMK58_27455	K19431 pyruvyl transferase EpsO [EC:2.-.-.-] | (GenBank) hypothetical protein	transferase	20.295	11.779	20.289	292.680	46.277	194.966
AMK58_27460	no KO assigned | (GenBank) copper oxidase	No COG	9.139	9.181	12.688	89.598	10.818	29.099
AMK58_27465	no KO assigned | (GenBank) hypothetical protein	Hypothetical	344.166	351.631	342.692	291.709	218.316	317.838

AMK58_27470	no KO assigned | (GenBank) transcriptional regulator	Transcription	572.615	504.432	507.519	305.145	206.702	312.382
AMK58_27475	K18306 membrane fusion protein, multidrug efflux system | (GenBank) RND transporter	Transport	1299.516	964.474	1032.243	383.692	292.776	468.677
AMK58_27480	K18307 multidrug efflux pump | (GenBank) acriflavine resistance protein B	Defense	1.141	0.235	1.197	60.596	3.183	56.791
AMK58_27485	K19267 NAD(P)H dehydrogenase (quinone) [EC:1.6.5.2] | (GenBank) NAD(P)-dependent oxidoreductase	Energy	14.311	13.680	19.731	103.139	21.717	130.450
AMK58_27490	no KO assigned | (GenBank) transcriptional regulator	Transcription	31.877	24.461	39.485	454.434	251.477	695.041
AMK58_27495	K03781 catalase [EC:1.11.1.6] | (GenBank) catalase	Metabolism	6.754	6.889	10.729	126.328	34.810	85.768
AMK58_27500	K04761 LysR family transcriptional regulator, hydrogen peroxide-inducible genes activator | (GenBank	Transcription	10.348	8.501	10.823	179.513	126.351	128.942
AMK58_27505	no KO assigned | (GenBank) RNA methyltransferase	methyltransferase	10.090	6.028	11.512	58.696	72.907	70.169
AMK58_27510	no KO assigned | (GenBank) hypothetical protein	Hypothetical	473.537	565.825	553.083	707.851	3145.870	1257.919
AMK58_27515	no KO assigned | (GenBank) hypothetical protein	Hypothetical	0.000	0.000	0.099	1.397	0.117	13.732
AMK58_27520	K01985 5S ribosomal RNA | (GenBank) 5S ribosomal RNA	Ribosome	0.000	0.000	0.000	0.000	0.000	0.000
AMK58_27525	K01980 23S ribosomal RNA | (GenBank) 23S ribosomal RNA	Ribosome	0.000	0.000	0.000	0.000	0.000	0.000
AMK58_27530	K14218 tRNA Ala | (GenBank) tRNA-Ala	tRNA synthesis/modification	0.000	0.000	0.000	0.000	0.000	0.000
AMK58_27535	K14227 tRNA Ile | (GenBank) tRNA-Ile	tRNA synthesis/modification	0.000	0.000	0.000	0.000	0.000	0.000
AMK58_27540	K01977 16S ribosomal RNA | (GenBank) 16S ribosomal RNA	Ribosome	42.207	61.000	46.246	13.569	34.068	47.487
AMK58_27545	no KO assigned | (GenBank) hypothetical protein	Hypothetical	1416.885	1339.135	1433.969	643.753	760.726	1056.486
AMK58_27550	K01686 mannonate dehydratase [EC:4.2.1.8] | (GenBank) mannonate dehydratase	Metabolism: Carbohydrate	55.328	55.596	55.780	216.654	119.607	108.893
AMK58_27555	K00040 fructuronate reductase [EC:1.1.1.57] | (GenBank) mannitol dehydrogenase	Metabolism: Carbohydrate	12.556	13.152	15.737	97.967	38.399	90.194
AMK58_27560	no KO assigned | (GenBank) transcriptional regulator	Transcription	102.355	111.838	86.707	152.984	42.662	567.559
AMK58_27565	no KO assigned | (GenBank) hypothetical protein	Hypothetical	59.576	70.422	72.360	207.323	53.429	188.661
AMK58_27570	no KO assigned | (GenBank) GntR family transcriptional regulator	Transcription	23.628	26.304	22.745	126.210	23.589	108.868
AMK58_27575	K00065 2-deoxy-D-gluconate 3-dehydrogenase [EC:1.1.1.125] | (GenBank) 2-deoxy-D-gluconate 3-dehyd	Metabolism: Carbohydrate	26.640	34.309	36.705	111.970	10.400	120.553
AMK58_27580	no KO assigned | (GenBank) cupin	No COG	2123.854	1916.031	1947.771	413.671	164.851	622.495
AMK58_27585	no KO assigned | (GenBank) C4-dicarboxylate ABC transporter	Membrane Transport	89.033	61.376	82.095	99.605	27.081	147.878
AMK58_27590	no KO assigned | (GenBank) C4-dicarboxylate ABC transporter permease	Membrane Transport	339.950	298.809	285.561	312.100	60.393	292.498
AMK58_27595	no KO assigned | (GenBank) hypothetical protein	Hypothetical	210.655	216.331	181.196	190.101	37.097	198.483
AMK58_27600	no KO assigned | (GenBank) hypothetical protein	Hypothetical	21.792	21.151	21.767	32.247	5.129	15.611
AMK58_27605	no KO assigned | (GenBank) hypothetical protein	Hypothetical	131.040	175.630	151.597	170.862	34.163	29.107
AMK58_27610	no KO assigned | (GenBank) hypothetical protein	Hypothetical	12976.911	12782.726	10090.997	1611.046	559.278	2078.058
AMK58_27615	K02014 iron complex outermembrane recepter protein | (GenBank) membrane receptor protein	Transport: Metal	40.574	46.035	43.240	95.854	95.271	90.935
AMK58_27620	K19265 L-glyceraldehyde 3-phosphate reductase [EC:1.1.1.-] | (GenBank) aldo/keto reductase	Oxidoreductases	143.638	191.088	165.325	88.805	194.792	146.997
AMK58_27625	no KO assigned | (GenBank) GntR family transcriptional regulator	Transcription	17.427	21.007	17.442	201.708	70.280	181.304
AMK58_27630	K01734 methylglyoxal synthase [EC:4.2.3.3] | (GenBank) methylglyoxal synthase	Metabolism: Carbohydrate	168.948	126.379	161.167	612.951	710.813	609.611
AMK58_27635	K01812 glucuronate isomerase [EC:5.3.1.12] | (GenBank) glucuronate isomerase	Metabolism: Carbohydrate	16.902	23.184	23.532	231.958	114.090	174.562
AMK58_27640	K01685 altronate hydrolase [EC:4.2.1.7] | (GenBank) galactonate dehydratase	Metabolism: Carbohydrate	0.715	0.949	1.964	137.709	11.966	37.350
AMK58_27645	K17850 LysR family transcriptional regulator, regulator of gene expression of beta-lactamase | (GenB	Transcription	3.440	2.372	4.781	111.137	27.070	206.748
AMK58_27650	no KO assigned | (GenBank) permease	Membrane transport	530.607	424.599	382.048	233.910	67.414	301.026
AMK58_27655	K07795 putative tricarboxylic transport membrane protein | (GenBank) C4-dicarboxylate ABC transporte	Membrane Transport	381.784	346.050	300.115	202.565	39.624	153.158
AMK58_27660	K07795 putative tricarboxylic transport membrane protein | (GenBank) C4-dicarboxylate ABC transporte	Membrane Transport	52.767	42.682	52.197	135.323	21.978	91.117
AMK58_27665	K07794 putative tricarboxylic transport membrane protein | (GenBank) C4-dicarboxylate ABC transporte	Membrane Transport	202.782	167.156	156.082	416.907	115.220	434.385
AMK58_27670	K07793 putative tricarboxylic transport membrane protein | (GenBank) tripartite tricarboxylate trans	Signal transduction	18.718	15.571	14.568	87.388	17.933	45.845
AMK58_27675	no KO assigned | (GenBank) hypothetical protein	Hypothetical	69.186	81.808	44.711	182.459	77.565	163.392
AMK58_27680	no KO assigned | (GenBank) universal stress protein UspA	Stress response	31.818	40.324	34.837	97.976	91.348	163.870
AMK58_27685	K07774 two-component system, OmpR family, response regulator TctD | (GenBank) two-component syste	Signal transduction	160.357	203.611	187.090	808.527	550.018	429.452
AMK58_27690	K07649 two-component system, OmpR family, sensor histidine kinase TctE [EC:2.7.13.3] | (GenBank) his	Signal Transduction	54.354	68.155	50.177	48.540	48.435	25.023
AMK58_27695	no KO assigned | (GenBank) hypothetical protein	Hypothetical	4591.220	5686.245	5837.013	118.149	501.412	266.671
AMK58_27700	no KO assigned | (GenBank) hypothetical protein	Hypothetical	1114.303	1340.335	1386.506	433.291	692.474	624.913
AMK58_27705	no KO assigned | (GenBank) hypothetical protein	Hypothetical	115.113	118.504	131.580	309.167	239.194	258.667
AMK58_27710	no KO assigned | (GenBank) hypothetical protein	Hypothetical	11.056	11.086	14.996	97.664	33.372	39.789
AMK58_27715	K02033 peptide/nickel transport system permease protein | (GenBank) hypothetical protein	Quorum sensing	19.200	21.069	22.974	196.128	51.677	38.392
AMK58_27720	K02034 peptide/nickel transport system permease protein | (GenBank) hypothetical protein	Quorum sensing	59.514	57.659	72.575	144.165	132.642	144.812
AMK58_27725	no KO assigned | (GenBank) hypothetical protein	Hypothetical	25.440	17.826	29.116	232.866	86.492	350.060
AMK58_27730	no KO assigned | (GenBank) hypothetical protein	Hypothetical	8.471	4.549	10.046	62.222	23.217	65.138
AMK58_27735	no KO assigned | (GenBank) hypothetical protein	Hypothetical	9.833	7.344	18.310	378.434	36.153	126.897
AMK58_27740	K02433 aspartyl-tRNA(Asn)/glutamyl-tRNA(Gln) amidotransferase subunit A [EC:6.3.5.6 6.3.5.7] | (Gen	tRNA synthesis/modification	2.441	1.042	2.403	42.269	5.291	20.611
AMK58_27745	no KO assigned | (GenBank) hypothetical protein	Hypothetical	13.282	21.365	28.658	786.294	103.936	387.496
AMK58_27750	no KO assigned | (GenBank) hypothetical protein	Hypothetical	15.985	24.585	22.271	302.535	55.149	272.676
AMK58_27755	no KO assigned | (GenBank) CoA-transferase	transferase	4.371	6.074	5.509	87.834	17.483	79.371
AMK58_27760	no KO assigned | (GenBank) acyl-CoA dehydrogenase	dehydrogenase	0.206	0.000	0.294	17.791	1.852	2.005
AMK58_27765	no KO assigned | (GenBank) 2-methylcitrate dehydratase	dehydratase	0.000	0.000	0.000	0.000	0.000	0.000
AMK58_27770	no KO assigned | (GenBank) dehydratase	dehydratase	0.000	0.000	0.000	0.000	0.000	0.000
AMK58_27775	K18292 (S)-citramalyl-CoA lyase [EC:4.1.3.25] | (GenBank) hypothetical protein	Metabolism: Carbohydrate	0.000	0.000	0.000	0.000	0.000	0.000
AMK58_27780	no KO assigned | (GenBank) hypothetical protein	Hypothetical	0.000	0.000	0.000	0.000	0.000	0.000
AMK58_27785	K11688 C4-dicarboxylate-binding protein DctP | (GenBank) hypothetical protein	Signal transduction	0.000	0.000	0.000	0.000	0.000	0.000
AMK58_27790	no KO assigned | (GenBank) 3-oxoacyl-ACP reductase	enzyme	0.000	0.000	0.000	0.000	0.000	0.000
AMK58_27795	no KO assigned | (GenBank) glycosyl transferase	Glycosyl Transferase	0.000	0.000	0.000	0.000	0.000	0.000
AMK58_27800	no KO assigned | (GenBank) hypothetical protein	Hypothetical	0.000	0.000	0.000	0.000	0.000	0.000

AMK58_27805	no KO assigned | (GenBank) hypothetical protein	Hypothetical	0.000	0.000	0.000	0.000	0.000	0.000
AMK58_27810	no KO assigned | (GenBank) hypothetical protein	Hypothetical	0.000	0.000	0.000	0.000	0.000	0.000
AMK58_27815	no KO assigned | (GenBank) hypothetical protein	Hypothetical	0.000	0.000	0.000	0.000	0.000	0.000
AMK58_27820	no KO assigned | (GenBank) hypothetical protein	Hypothetical	0.000	0.000	0.000	0.000	0.000	0.000
AMK58_27825	K04748 nitric oxide reductase NorQ protein | (GenBank) AAA family ATPase	Nitrogen	0.000	0.000	0.000	0.000	0.000	0.000
AMK58_27830	K04561 nitric oxide reductase subunit B [EC:1.7.2.5] | (GenBank) nitric oxide reductase	Nitrogen	0.000	0.000	0.000	0.000	0.000	0.000
AMK58_27835	K02305 nitric oxide reductase subunit C | (GenBank) cytochrome C	Energy	7.216	7.984	8.894	106.692	0.000	0.000
AMK58_27840	K00368 nitrite reductase (NO-forming) [EC:1.7.2.1] | (GenBank) copper oxidase	Nitrogen	0.000	0.000	0.000	0.000	0.000	0.000
AMK58_27845	no KO assigned | (GenBank) hypothetical protein	Hypothetical	4.634	4.615	3.488	122.092	0.000	0.179
AMK58_27850	no KO assigned | (GenBank) hypothetical protein	Hypothetical	16.188	9.274	18.343	232.288	0.000	0.411
AMK58_27855	no KO assigned | (GenBank) hypothetical protein	Hypothetical	66.028	59.049	163.673	224.339	0.000	0.255
AMK58_27860	K00404 cytochrome c oxidase cbb3-type subunit I [EC:1.9.3.1] | (GenBank) hypothetical protein	Energy	24.311	21.571	46.612	76.418	0.000	0.149
AMK58_27865	K01739 cystathionine gamma-synthase [EC:2.5.1.48] | (GenBank) O-succinylhomoserine (thiol)-lyase	Biosynthesis: Amino Acid	4.802	5.494	9.715	56.115	0.000	0.000
AMK58_27870	no KO assigned | (GenBank) hypothetical protein	Hypothetical	8.698	14.550	50.613	2576.332	0.000	0.323
AMK58_27875	K02230 cobaltochelatase CobN [EC:6.6.1.2] | (GenBank) cobalamin biosynthesis protein CobN	Metabolism: Co-Factors and Vitamens	0.309	0.184	0.440	30.526	0.000	0.000
AMK58_27880	no KO assigned | (GenBank) biopolymer transporter ExbD	Transport: Biopolymer	0.990	2.959	2.119	79.932	0.000	0.000
AMK58_27885	no KO assigned | (GenBank) hypothetical protein	Hypothetical	4.443	6.636	8.449	300.565	0.000	0.000
AMK58_27890	no KO assigned | (GenBank) hypothetical protein	Hypothetical	9.526	8.986	19.306	578.228	0.000	0.000
AMK58_27895	K02016 iron complex transport system substrate-binding protein | (GenBank) iron complex transporter	Transport: Metal	0.993	0.742	3.424	237.041	0.000	0.115
AMK58_27900	K02015 iron complex transport system permease protein | (GenBank) ABC transporter permease	Transport: Metal	1.294	0.703	3.132	179.366	0.000	0.000
AMK58_27905	no KO assigned | (GenBank) hypothetical protein	Hypothetical	20.179	31.290	26.217	512.144	0.000	0.286
AMK58_27910	K02014 iron complex outermembrane recepter protein | (GenBank) TonB-dependent receptor	Transport: Metal	13.490	13.769	14.858	74.467	0.000	0.000
AMK58_27915	K07722 CopG family transcriptional regulator, nickel-responsive regulator | (GenBank) nickel respons	Transcription	86.231	113.820	87.471	170.245	0.000	0.232
AMK58_27920	K15584 nickel transport system substrate-binding protein | (GenBank) hypothetical protein	Transport: Metal	78.427	85.503	99.606	372.299	0.000	0.265
AMK58_27925	no KO assigned | (GenBank) hypothetical protein	Hypothetical	5.185	7.228	7.477	109.166	0.000	0.080
AMK58_27930	no KO assigned | (GenBank) hypothetical protein	Hypothetical	1.218	2.079	2.316	65.014	0.000	0.161
AMK58_27935	no KO assigned | (GenBank) hypothetical protein	Hypothetical	6.402	6.652	11.115	241.228	0.000	0.258
AMK58_27940	no KO assigned | (GenBank) hypothetical protein	Hypothetical	38.677	43.535	41.837	230.890	2.520	28.847
AMK58_27945	K07497 putative transposase | (GenBank) integrase	Genetic Information Processing	2.758	4.337	3.037	55.618	0.000	0.000
AMK58_27950	K07483 transposase | (GenBank) transposase	Genetic Information Processing	12.320	25.381	11.310	76.256	0.000	0.000
AMK58_27955	no KO assigned | (GenBank) hypothetical protein	Hypothetical	269.361	326.971	234.889	81.912	0.000	0.567
AMK58_27960	no KO assigned | (GenBank) hypothetical protein	Hypothetical	199.255	190.139	172.788	43.558	0.000	0.000
AMK58_27965	no KO assigned | (GenBank) hypothetical protein	Hypothetical	94.676	104.870	92.647	104.051	0.000	0.256
AMK58_27970	no KO assigned | (GenBank) hypothetical protein	Hypothetical	663.738	701.098	668.280	285.281	0.000	1.727
AMK58_27975	no KO assigned | (GenBank) hypothetical protein	Hypothetical	16.894	11.215	23.648	127.611	0.000	0.000
AMK58_27980	no KO assigned | (GenBank) hypothetical protein	Hypothetical	2.187	4.574	4.160	89.371	0.000	0.000
AMK58_27985	no KO assigned | (GenBank) hypothetical protein	Hypothetical	26.849	21.455	26.287	112.935	0.000	0.000
AMK58_27990	K07497 putative transposase | (GenBank) hypothetical protein	Genetic Information Processing	23.010	22.117	24.363	78.176	0.000	0.269
AMK58_27995	no KO assigned | (GenBank) transposase	Genetic Information Processing	44.251	50.395	45.900	86.047	0.000	0.000
AMK58_28000	no KO assigned | (GenBank) hypothetical protein	Hypothetical	10.612	18.471	15.969	110.139	0.000	0.155
AMK58_28005	no KO assigned | (GenBank) hypothetical protein	Hypothetical	11.860	24.604	26.470	135.707	0.000	0.153
AMK58_28010	no KO assigned | (GenBank) enoyl-CoA hydratase	hydratase	6.777	8.097	7.016	89.767	0.000	0.279
AMK58_28015	K00059 3-oxoacyl-[acyl-carrier protein] reductase [EC:1.1.1.100] | (GenBank) 3-oxoacyl-ACP reductase	Metabolism: Lipid	6.914	10.567	8.867	122.339	0.000	0.149
AMK58_28020	no KO assigned | (GenBank) propanoyl-CoA acyltransferase	Transferase	4.729	2.512	6.296	136.281	0.000	0.097
AMK58_28025	no KO assigned | (GenBank) hypothetical protein	Hypothetical	2.182	4.344	1.383	106.663	0.000	0.000
AMK58_28030	no KO assigned | (GenBank) short-chain dehydrogenase	Dehydrogenase	11.138	12.099	12.929	179.431	0.000	0.268
AMK58_28035	K03566 LysR family transcriptional regulator, glycine cleavage system transcriptional activator | (G	Transcription	53.081	52.310	55.023	99.511	0.000	0.000
AMK58_28040	K03761 MFS transporter, MHS family, alpha-ketoglutarate permease | (GenBank) transporter	Transport: MFS	10.940	11.534	11.713	122.779	0.000	0.085
AMK58_28045	no KO assigned | (GenBank) hydroxyacid dehydrogenase	Dehydrogenase	2.854	3.247	2.196	135.974	0.000	0.000
AMK58_28050	no KO assigned | (GenBank) carnitine dehydratase	Dehydrogenase	8.371	10.121	11.370	148.303	0.000	0.370
AMK58_28055	K00128 aldehyde dehydrogenase (NAD+) [EC:1.2.1.3] | (GenBank) aldehyde dehydrogenase	Energy	2.949	5.034	4.646	223.697	0.000	0.000
AMK58_28060	no KO assigned | (GenBank) hypothetical protein	Hypothetical	4.901	1.722	5.482	234.011	0.000	0.000
AMK58_28065	no KO assigned | (GenBank) transcriptional regulator	Transcription	32.834	56.397	38.243	96.945	0.154	0.127
AMK58_28070	no KO assigned | (GenBank) hypothetical protein	Hypothetical	2.274	5.094	4.594	103.175	0.000	0.527
AMK58_28075	K06893 uncharacterized protein | (GenBank) ketosteroid isomerase	Enzyme	13.369	16.577	10.553	81.093	0.000	0.000
AMK58_28080	no KO assigned | (GenBank) pseudogene	Pseudogene	15.785	17.146	13.985	133.353	0.000	0.000
AMK58_28085	K01118 FMN-dependent NADH-azoreductase [EC:1.7.-.-] | (GenBank) FMN-dependent NADH-azoreduc	Energy	13.986	13.130	11.398	95.278	0.000	0.185
AMK58_28090	no KO assigned | (GenBank) Isoquinoline 1-oxidoreductase subunit	Oxidoreductase	7.884	12.336	12.850	251.954	0.000	0.174
AMK58_28095	K07303 isoquinoline 1-oxidoreductase subunit beta [EC:1.3.99.16] | (GenBank) aldehyde dehydrogenase	Oxidoreductase	7.248	8.857	10.389	200.066	0.000	0.051
AMK58_28100	K07302 isoquinoline 1-oxidoreductase subunit alpha [EC:1.3.99.16] | (GenBank) hypothetical protein	Oxidoreductase	11.930	10.615	13.998	124.104	0.000	0.235
AMK58_28105	no KO assigned | (GenBank) hypothetical protein	Hypothetical	30.114	40.555	37.943	187.316	0.000	0.114
AMK58_28110	no KO assigned | (GenBank) hypothetical protein	Hypothetical	3.765	6.298	6.443	135.155	0.000	0.000
AMK58_28115	K07497 putative transposase | (GenBank) hypothetical protein	Genetic Information Processing	5.228	9.316	9.157	114.636	0.000	0.170
AMK58_28120	no KO assigned | (GenBank) hypothetical protein	Hypothetical	4.426	2.333	5.075	50.574	0.000	0.000
AMK58_28125	no KO assigned | (GenBank) hypothetical protein	Hypothetical	4.283	5.204	8.075	129.710	0.000	0.000
AMK58_28130	no KO assigned | (GenBank) hypothetical protein	Hypothetical	4.805	5.741	6.091	90.532	0.000	0.000
AMK58_28135	no KO assigned | (GenBank) hypothetical protein	Hypothetical	10.158	12.550	12.638	183.312	0.000	0.032

AMK58_28140	no KO assigned | (GenBank) hypothetical protein	Hypothetical	8.584	9.185	15.836	181.408	0.000	0.000
AMK58_28145	no KO assigned | (GenBank) hypothetical protein	Hypothetical	12.055	6.263	16.944	103.209	0.000	0.000
AMK58_28150	no KO assigned | (GenBank) hypothetical protein	Hypothetical	93.401	86.114	129.920	139.789	0.000	0.535
AMK58_28155	no KO assigned | (GenBank) hypothetical protein	Hypothetical	20.737	18.650	20.141	199.820	130.830	194.146
AMK58_28160	K13953 alcohol dehydrogenase, propanol-preferring [EC:1.1.1.1] | (GenBank) alcohol dehydrogenase	Metabolism	41.778	45.254	44.787	266.663	270.191	193.042
AMK58_28165	no KO assigned | (GenBank) hypothetical protein	Hypothetical	3.759	7.683	6.678	115.677	1.001	8.255
AMK58_28170	K03315 Na+:H+ antiporter, NhaC family | (GenBank) sodium:proton antiporter	Transport: Ion	4.502	4.803	5.504	169.186	0.000	0.149
AMK58_28175	K00926 carbamate kinase [EC:2.7.2.2] | (GenBank) carbamate kinase	Metabolism	9.265	9.857	12.550	231.378	0.000	0.000
AMK58_28180	K00611 ornithine carbamoyltransferase [EC:2.1.3.3] | (GenBank) ornithine carbamoyltransferase	Biosynthesis: Amino Acid	9.999	15.115	14.433	110.545	0.000	0.223
AMK58_28185	K01478 arginine deiminase [EC:3.5.3.6] | (GenBank) arginine deiminase	Metabolism: Amino Acid	8.367	8.233	13.384	122.101	0.000	0.183
AMK58_28190	K03758 arginine:ornithine antiporter / lysine permease | (GenBank) arginine-ornithine antiporter	Transport: Amino Acid	11.075	7.018	14.520	116.554	0.000	0.078
AMK58_28195	no KO assigned | (GenBank) hypothetical protein	Hypothetical	39.097	35.163	44.768	116.571	0.000	0.000
AMK58_28200	no KO assigned | (GenBank) hypothetical protein	Hypothetical	5.222	4.237	8.888	174.622	0.000	0.060
AMK58_28205	no KO assigned | (GenBank) hypothetical protein	Hypothetical	13.986	7.162	12.918	123.111	0.000	0.000
AMK58_28210	no KO assigned | (GenBank) hypothetical protein	Hypothetical	6.998	9.164	12.487	83.982	0.000	0.000
AMK58_28215	K02778 PTS system, glucose-specific IIB component [EC:2.7.1.199] K02779 PTS system, glucose-specifi	Metabolism: Carbohydrates	6.501	6.473	14.293	188.128	0.000	0.063
AMK58_28220	no KO assigned | (GenBank) PTS sugar transporter	Transport: Sugar	9.547	8.642	13.295	215.214	0.000	0.089
AMK58_28225	K16044 scyllo-inositol 2-dehydrogenase (NADP+) [EC:1.1.1.371] | (GenBank) oxidoreductase	Metabolism: Carbohydrates	14.430	23.131	20.637	163.129	0.000	0.000
AMK58_28230	K16370 6-phosphofructokinase 2 [EC:2.7.1.11] | (GenBank) 1-phosphofructokinase	Metabolism: Carbohydrates	23.304	30.334	31.936	133.411	0.000	0.121
AMK58_28235	no KO assigned | (GenBank) hypothetical protein	Hypothetical	37.369	17.861	42.163	90.771	0.000	0.462
AMK58_28240	no KO assigned | (GenBank) pseudogene	Pseudogene	1.020	2.611	5.126	197.316	0.000	0.135
AMK58_28245	K03797 carboxyl-terminal processing protease [EC:3.4.21.102] | (GenBank) carboxyl-terminal protease	Hydrolase	5.449	8.139	7.772	110.975	0.000	0.158
AMK58_28250	no KO assigned | (GenBank) pseudogene	Pseudogene	9.539	10.522	13.256	251.634	0.000	0.136
AMK58_28255	no KO assigned | (GenBank) hypothetical protein	Hypothetical	14.294	10.047	15.722	79.216	0.000	0.260
AMK58_28260	no KO assigned | (GenBank) hypothetical protein	Hypothetical	26.822	28.455	27.648	59.435	0.000	0.465
AMK58_28265	no KO assigned | (GenBank) hypothetical protein	Hypothetical	64.979	98.570	75.298	69.363	0.000	0.471
AMK58_28270	no KO assigned | (GenBank) hypothetical protein	Hypothetical	14.314	11.878	16.824	137.419	0.000	0.000
AMK58_28275	K01034 acetate CoA/acetoacetate CoA-transferase alpha subunit [EC:2.8.3.8 2.8.3.9] | (GenBank) acety	Metabolism: Carbohydrates	7.853	7.637	8.508	51.371	0.000	0.169
AMK58_28280	K01035 acetate CoA/acetoacetate CoA-transferase beta subunit [EC:2.8.3.8 2.8.3.9] | (GenBank) acetat	Metabolism	10.180	13.033	11.408	128.093	0.000	0.169
AMK58_28285	K02557 chemotaxis protein MotB | (GenBank) hypothetical protein	Chemotaxis	9.330	15.556	11.553	144.165	0.000	0.201
AMK58_28290	no KO assigned | (GenBank) inosine-5-monophosphate dehydrogenase	Metabolism	6.054	13.778	7.401	65.261	0.000	0.000
AMK58_28295	no KO assigned | (GenBank) hypothetical protein	Hypothetical	14.072	15.070	19.565	259.284	0.000	0.000
AMK58_28300	no KO assigned | (GenBank) hypothetical protein	Hypothetical	10.598	23.418	19.048	148.962	0.000	0.000
AMK58_28305	no KO assigned | (GenBank) hypothetical protein	Hypothetical	19.555	34.801	23.868	124.658	0.000	0.000
AMK58_28310	K07497 putative transposase | (GenBank) hypothetical protein	Genetic Information Processing	35.682	59.313	43.845	142.045	0.000	0.000
AMK58_28315	no KO assigned | (GenBank) hypothetical protein	Hypothetical	0.835	3.326	1.852	85.753	0.000	0.000
AMK58_28320	no KO assigned | (GenBank) Sulphatase-modifying factor protein	No COG	14.268	16.237	16.925	135.974	0.000	0.000
AMK58_28325	no KO assigned | (GenBank) pseudogene	Pseudogene	11.007	16.202	12.285	200.008	0.000	0.296
AMK58_28330	no KO assigned | (GenBank) hypothetical protein	Hypothetical	0.364	2.172	3.803	217.222	0.000	0.000
AMK58_28335	no KO assigned | (GenBank) hypothetical protein	Hypothetical	0.000	0.000	6.140	303.828	0.000	0.000
AMK58_28340	no KO assigned | (GenBank) AAA family ATPase	Energy	1.597	4.337	4.693	168.409	0.000	0.000
AMK58_28345	no KO assigned | (GenBank) hypothetical protein	Hypothetical	0.700	0.000	4.658	179.061	0.000	0.000
AMK58_28350	no KO assigned | (GenBank) integrase	Genetic Information Processing	1.603	1.690	3.048	155.365	0.000	0.000
AMK58_28355	no KO assigned | (GenBank) AAA family ATPase	Energy	8.321	10.772	12.923	186.337	0.000	0.129
AMK58_28360	no KO assigned | (GenBank) hypothetical protein	Hypothetical	2.446	3.653	8.721	379.253	0.687	0.567
AMK58_28365	K09803 uncharacterized protein | (GenBank) hypothetical protein	Hypothetical	358.690	384.706	342.339	103.872	0.000	0.420
AMK58_28370	K07726 putative transcriptional regulator | (GenBank) hypothetical protein	Transcription	237.986	294.700	266.479	93.965	0.000	0.000
AMK58_28375	no KO assigned | (GenBank) hypothetical protein	Hypothetical	132.258	174.303	141.009	311.996	0.000	0.000
AMK58_28380	no KO assigned | (GenBank) pseudogene	Pseudogene	17.624	16.847	15.249	131.496	23.772	51.151
AMK58_28385	no KO assigned | (GenBank) hypothetical protein	Hypothetical	143.712	170.019	162.347	123.892	0.000	0.240
AMK58_28390	K03496 chromosome partitioning protein | (GenBank) hypothetical protein	Cell Cycle/Shape/Homeostatsis	211.320	221.025	230.060	108.875	0.000	0.000
AMK58_28395	no KO assigned | (GenBank) hypothetical protein	Hypothetical	119.327	118.661	149.715	207.734	0.000	0.000
AMK58_28400	no KO assigned | (GenBank) hypothetical protein	Hypothetical	84.918	93.557	93.088	59.860	0.000	0.000
AMK58_28405	K07483 transposase | (GenBank) hypothetical protein	Genetic Information Processing	2.736	4.087	9.431	298.715	0.000	0.000
AMK58_28410	no KO assigned | (GenBank) hypothetical protein	Hypothetical	0.917	2.740	3.052	107.510	0.000	0.851
AMK58_28415	no KO assigned | (GenBank) hypothetical protein	Hypothetical	0.216	2.579	4.720	187.916	0.000	0.000
AMK58_28420	no KO assigned | (GenBank) hypothetical protein	Hypothetical	0.000	0.000	3.101	185.667	0.000	0.000
AMK58_28425	K18144 two-component system, OmpR family, response regulator AdeR | (GenBank) two-component syst	Signal Transduction	7.194	6.290	8.842	147.378	0.000	0.163
AMK58_28430	K18143 two-component system, OmpR family, sensor histidine kinase AdeS [EC:2.7.13.3] | (GenBank) hi	Signal Transduction	6.243	4.663	8.268	176.224	0.000	0.207
AMK58_28435	K06147 ATP-binding cassette, subfamily B, bacterial | (GenBank) multidrug ABC transporter ATP-bindin	Membrane Transport	2.160	2.016	3.786	73.409	0.000	0.063
AMK58_28440	K18145 membrane fusion protein, multidrug efflux system | (GenBank) RND transporter	Membrane Transport	14.676	18.162	23.522	197.853	0.000	0.097
AMK58_28445	no KO assigned | (GenBank) pseudogene	Pseudogene	1.379	2.197	2.797	104.676	0.000	0.170
AMK58_28450	no KO assigned | (GenBank) TetR family transcriptional regulator	Transcription	11.974	25.172	22.560	167.830	0.000	0.000
AMK58_28455	no KO assigned | (GenBank) pseudogene	Pseudogene	19.757	16.646	21.996	195.664	0.000	0.000
AMK58_28460	no KO assigned | (GenBank) pseudogene	Pseudogene	12.370	11.944	14.731	180.430	0.000	0.116
AMK58_28465	no KO assigned | (GenBank) hypothetical protein	Hypothetical	10.051	17.553	15.437	54.269	0.000	0.143
AMK58_28470	no KO assigned | (GenBank) pseudogene	Pseudogene	13.469	16.989	15.368	154.167	0.000	0.000

AMK58_28475	K15864 nitrite reductase (NO-forming) / hydroxylamine reductase [EC:1.7.2.1 1.7.99.1] | (GenBank) ni	Nitrogen	6.862	11.020	12.838	55.643	0.000	0.068
AMK58_28480	no KO assigned | (GenBank) hypothetical protein	Hypothetical	3.208	4.011	5.603	139.502	0.000	0.000
AMK58_28485	no KO assigned | (GenBank) hypothetical protein	Hypothetical	6.294	13.273	11.618	203.352	0.000	0.000
AMK58_28490	no KO assigned | (GenBank) cytochrome C oxidase Cbb3	Energy	3.836	5.042	5.253	103.088	0.000	0.071
AMK58_28495	no KO assigned | (GenBank) hypothetical protein	Hypothetical	10.232	27.168	16.214	160.663	0.000	0.000
AMK58_28500	K13796 tricarballylate dehydrogenase | (GenBank) hypothetical protein	Dehydrogenase	4.314	4.296	7.093	176.273	0.000	0.083
AMK58_28505	K13795 citrate/tricarballylate utilization protein | (GenBank) hypothetical protein	Enzyme	8.735	9.909	12.300	149.448	0.000	0.000
AMK58_28510	no KO assigned | (GenBank) ferredoxin	Ferredoxin	1.511	6.373	4.649	98.598	0.000	0.165
AMK58_28515	K07243 high-affinity iron transporter | (GenBank) hypothetical protein	Transport: Metal	0.669	1.332	2.014	157.258	0.000	0.000
AMK58_28520	no KO assigned | (GenBank) transposase	Genetic Information Processing	0.904	0.000	1.182	10.600	0.000	0.000
AMK58_28525	no KO assigned | (GenBank) hypothetical protein	Hypothetical	8.670	16.075	11.370	153.776	0.000	0.000
AMK58_28530	K07230 periplasmic iron binding protein | (GenBank) hypothetical protein	Transport: Metal	33.632	24.943	31.536	143.855	0.000	0.430
AMK58_28535	no KO assigned | (GenBank) hypothetical protein	Hypothetical	1.596	0.000	2.602	118.814	0.000	0.423
AMK58_28540	K01719 uroporphyrinogen-III synthase [EC:4.2.1.75] | (GenBank) hypothetical protein	Metabolism: Co-Factors and Vitamens	2.894	6.724	5.198	200.525	0.000	0.149
AMK58_28545	K01749 hydroxymethylbilane synthase [EC:2.5.1.61] | (GenBank) porphobilinogen deaminase	Metabolism: Co-Factors and Vitamens	5.896	8.611	9.095	157.622	0.000	0.000
AMK58_28550	no KO assigned | (GenBank) hypothetical protein	Hypothetical	1.755	4.765	4.702	247.873	0.000	0.000
AMK58_28555	no KO assigned | (GenBank) heme d1 biosynthesis radical SAM protein NirJ	Enzyme	2.259	3.681	3.320	177.868	0.000	0.286
AMK58_28560	no KO assigned | (GenBank) protein nirH	No COG	2.431	4.358	4.854	295.061	0.000	0.000
AMK58_28565	no KO assigned | (GenBank) protein nirG	No COG	1.091	4.888	4.148	191.774	0.000	0.000
AMK58_28570	no KO assigned | (GenBank) hypothetical protein	Hypothetical	3.068	8.460	6.283	234.585	0.000	0.000
AMK58_28575	no KO assigned | (GenBank) AsnC family transcriptional regulator	Transcription	2.655	6.345	8.331	229.408	0.000	0.000
AMK58_28580	K19345 protein NirF | (GenBank) protein nirF	No COG	5.368	7.127	8.034	195.608	0.000	0.092
AMK58_28585	K19344 cytochrome c55X | (GenBank) hypothetical protein	Energy	3.844	4.593	7.309	277.003	0.000	0.000
AMK58_28590	K02302 uroporphyrin-III C-methyltransferase / precorrin-2 dehydrogenase / sirohydrochlorin ferrochel	Metabolism: Co-Factors and Vitamens	7.131	6.345	11.108	175.193	0.000	0.000
AMK58_28595	no KO assigned | (GenBank) hypothetical protein	Hypothetical	6.079	9.080	7.386	106.088	0.000	0.000
AMK58_28600	no KO assigned | (GenBank) hypothetical protein	Hypothetical	6.840	4.087	8.618	153.710	0.000	0.476
AMK58_28605	no KO assigned | (GenBank) hypothetical protein	Hypothetical	3.924	3.958	6.250	149.012	0.000	0.000
AMK58_28610	no KO assigned | (GenBank) transposase	Genetic Information Processing	2.175	2.888	4.136	89.024	0.000	0.000
AMK58_28615	K00643 5-aminolevulinate synthase [EC:2.3.1.37] | (GenBank) 5-aminolevulinate synthase	Metabolism: Co-Factors and Vitamens	12.556	14.289	10.801	146.301	0.336	0.000
AMK58_28620	no KO assigned | (GenBank) hypothetical protein	Hypothetical	23.194	18.015	13.453	111.542	0.000	0.430
AMK58_28625	no KO assigned | (GenBank) hypothetical protein	Hypothetical	4.371	5.000	7.250	145.369	0.000	0.086
AMK58_28630	no KO assigned | (GenBank) hypothetical protein	Hypothetical	2.070	3.091	6.395	288.330	0.000	0.480
AMK58_28635	K04561 nitric oxide reductase subunit B [EC:1.7.2.5] | (GenBank) nitric oxide reductase	Nitrogen	13.870	11.227	14.720	148.281	83.084	212.673
AMK58_28640	K02305 nitric oxide reductase subunit C | (GenBank) cytochrome C	Nitrogen	7.751	15.968	10.419	318.285	29.442	80.051
AMK58_28645	K07498 putative transposase | (GenBank) hypothetical protein	Genetic Information Processing	52.398	64.839	59.011	188.535	50.130	97.804
AMK58_28650	no KO assigned | (GenBank) CoA-binding protein	No COG	9.991	11.067	14.090	86.319	0.000	0.104
AMK58_28655	no KO assigned | (GenBank) formyl-coenzyme A transferase	Transferase	2.937	4.528	4.324	93.530	0.000	0.000
AMK58_28660	no KO assigned | (GenBank) lipase	Enzyme	94.613	101.224	97.923	124.955	0.000	0.353
AMK58_28665	no KO assigned | (GenBank) hypothetical protein	Hypothetical	49.592	45.248	59.384	56.147	0.000	0.000
AMK58_28670	no KO assigned | (GenBank) hypothetical protein	Hypothetical	116.090	111.682	111.746	152.940	0.970	7.822
AMK58_28675	K05916 nitric oxide dioxygenase [EC:1.14.12.17] | (GenBank) dihydropteridine reductase	Nitrogen	9.670	4.716	12.103	142.755	0.000	0.091
AMK58_28680	no KO assigned | (GenBank) hypothetical protein	Hypothetical	63.858	51.886	65.088	108.808	0.000	0.000
AMK58_28685	no KO assigned | (GenBank) hypothetical protein	Hypothetical	71.603	70.002	81.697	91.266	0.000	0.000
AMK58_28690	no KO assigned | (GenBank) hypothetical protein	Hypothetical	17.040	25.719	32.915	276.797	0.000	0.166
AMK58_28695	K02448 nitric oxide reductase NorD protein | (GenBank) protein norD	Nitrogen	3.226	2.646	7.458	104.396	0.000	0.000
AMK58_28700	K04748 nitric oxide reductase NorQ protein | (GenBank) AAA family ATPase	Nitrogen	0.000	0.000	0.000	0.000	0.000	0.000
AMK58_28705	K04561 nitric oxide reductase subunit B [EC:1.7.2.5] | (GenBank) nitric oxide reductase	Nitrogen	0.000	0.000	0.000	0.000	0.000	0.000
AMK58_28710	K02305 nitric oxide reductase subunit C | (GenBank) cytochrome C	Nitrogen	0.000	0.000	0.000	0.000	0.000	0.000
AMK58_28715	no KO assigned | (GenBank) pseudogene	Pseudogene	0.000	0.000	0.000	0.000	0.000	0.000
AMK58_28720	no KO assigned | (GenBank) hypothetical protein	Hypothetical	0.000	0.000	0.000	0.000	0.000	0.000
AMK58_28725	no KO assigned | (GenBank) hypothetical protein	Hypothetical	0.000	0.000	0.000	0.000	0.000	0.000
AMK58_28730	no KO assigned | (GenBank) hypothetical protein	Hypothetical	0.000	0.000	0.000	0.000	0.000	0.000
AMK58_28735	no KO assigned | (GenBank) hypothetical protein	Hypothetical	0.000	0.000	0.000	0.000	0.000	0.000
AMK58_28740	no KO assigned | (GenBank) hypothetical protein	Hypothetical	0.000	0.000	0.000	0.000	0.000	0.000
AMK58_28745	no KO assigned | (GenBank) hypothetical protein	Hypothetical	0.000	0.000	0.000	0.000	0.000	0.000
AMK58_28750	no KO assigned | (GenBank) hypothetical protein	Hypothetical	0.000	0.000	0.000	0.000	0.000	0.000
AMK58_28755	no KO assigned | (GenBank) hypothetical protein	Hypothetical	0.000	0.000	0.000	0.000	0.000	0.000
AMK58_28760	no KO assigned | (GenBank) hypothetical protein	Hypothetical	88.892	99.539	101.836	289.162	154.239	111.598
AMK58_28765	no KO assigned | (GenBank) hypothetical protein	Hypothetical	157.573	178.218	158.275	449.263	108.875	503.424
AMK58_28770	no KO assigned | (GenBank) pseudogene	Pseudogene	50.540	76.317	63.632	557.979	110.654	426.290
AMK58_28775	no KO assigned | (GenBank) hypothetical protein	Hypothetical	6.616	11.858	5.976	215.362	98.537	409.815
AMK58_28780	no KO assigned | (GenBank) hypothetical protein	Hypothetical	10.208	5.673	10.383	192.397	78.987	592.167
AMK58_28785	K02474 UDP-N-acetyl-D-galactosamine dehydrogenase [EC:1.1.1.-] | (GenBank) GDP-mannose dehydrog	Metabolism	73.413	72.727	72.472	224.652	98.728	390.903
AMK58_28790	no KO assigned | (GenBank) pseudogene	Pseudogene	24.888	40.186	28.780	237.873	86.948	724.139
AMK58_28795	no KO assigned | (GenBank) pseudogene	Pseudogene	50.707	61.825	55.100	275.855	157.031	861.694
AMK58_28800	no KO assigned | (GenBank) pseudogene	Pseudogene	26.675	24.836	29.315	120.653	165.687	396.718
AMK58_28805	no KO assigned | (GenBank) glycosyl transferase	Glycosyl Transferase	485.359	389.686	450.708	276.317	162.945	366.143

AMK58_28810	K07027 glycosyltransferase 2 family protein | (GenBank) hypothetical protein	glycosyltransferase	337.096	316.381	332.159	348.122	311.651	269.966
AMK58_28815	no KO assigned | (GenBank) hypothetical protein	Hypothetical	56.144	59.794	63.826	474.786	160.422	373.855
AMK58_28820	no KO assigned | (GenBank) hypothetical protein	Hypothetical	23.506	21.606	29.983	299.569	101.135	168.539
AMK58_28825	K01784 UDP-glucose 4-epimerase [EC:5.1.3.2] | (GenBank) UDP-glucose 4-epimerase	Metabolism	98.178	96.033	105.113	357.367	187.177	293.810
AMK58_28830	no KO assigned | (GenBank) hypothetical protein	Hypothetical	113.065	111.656	114.912	416.402	91.322	233.492
AMK58_28835	no KO assigned | (GenBank) hypothetical protein	Hypothetical	104.017	102.575	105.897	337.663	48.497	335.672
AMK58_28840	no KO assigned | (GenBank) hypothetical protein	Hypothetical	147.019	187.885	146.017	438.815	65.101	571.312
AMK58_28845	no KO assigned | (GenBank) hypothetical protein	Hypothetical	3350.532	3997.491	3923.890	145.469	197.872	531.804
AMK58_28850	no KO assigned | (GenBank) hypothetical protein	Hypothetical	703.497	654.537	722.840	217.391	242.743	542.835
AMK58_28855	K09691 lipopolysaccharide transport system ATP-binding protein | (GenBank) hypothetical protein	Transport: polysacchharide	338.022	335.807	353.097	187.013	144.757	397.682
AMK58_28860	no KO assigned | (GenBank) hypothetical protein	Hypothetical	252.265	238.035	245.548	135.777	106.276	415.962
AMK58_28865	K09690 lipopolysaccharide transport system permease protein | (GenBank) sugar ABC transporter permea	Transport: polysacchharide	115.952	95.520	86.634	168.216	128.067	461.169
AMK58_28870	no KO assigned | (GenBank) hypothetical protein	Hypothetical	180.163	182.504	170.346	172.341	167.422	458.900
AMK58_28875	no KO assigned | (GenBank) hypothetical protein	Hypothetical	7503.460	6627.596	6622.080	275.353	89.671	305.387
AMK58_28880	no KO assigned | (GenBank) hypothetical protein	Hypothetical	65.983	53.850	57.353	386.019	23.803	289.474
AMK58_28885	no KO assigned | (GenBank) SAM-dependent methyltransferase	methyltransferase	172.422	177.855	174.578	342.036	119.475	371.639
AMK58_28890	no KO assigned | (GenBank) glycosyl transferase family 1	Glycosyl Transferase	70.142	57.961	57.467	194.805	94.327	435.225
AMK58_28895	no KO assigned | (GenBank) hypothetical protein	Hypothetical	43.007	42.359	48.003	418.197	51.933	440.987
AMK58_28900	K03271 D-sedoheptulose 7-phosphate isomerase [EC:5.3.1.28] | (GenBank) phosphoheptose isomerase	Biosynthesis: polysacchharide	108.883	95.443	117.317	388.570	85.531	384.368
AMK58_28905	no KO assigned | (GenBank) FAD-dependent oxidoreductase	Biosynthesis: polysacchharide	63.883	85.745	82.697	447.963	57.979	347.516
AMK58_28910	no KO assigned | (GenBank) hypothetical protein	Hypothetical	26.328	33.632	49.984	292.584	84.304	455.744
AMK58_28915	no KO assigned | (GenBank) hypothetical protein	Hypothetical	142.009	149.571	148.567	298.642	98.492	241.150
AMK58_28920	K01790 dTDP-4-dehydrorhamnose 3,5-epimerase [EC:5.1.3.13] | (GenBank) dTDP-4-dehydrorhamnose 3	Metabolism	253.527	234.706	245.138	251.906	120.167	529.499
AMK58_28925	K01710 dTDP-glucose 4,6-dehydratase [EC:4.2.1.46] | (GenBank) dTDP-glucose 4,6-dehydratase	Metabolism	263.794	202.055	221.234	285.713	124.055	406.324
AMK58_28930	K00067 dTDP-4-dehydrorhamnose reductase [EC:1.1.1.133] | (GenBank) dTDP-4-dehydrorhamnose redu	Metabolism	68.218	60.669	64.368	323.500	58.681	157.660
AMK58_28935	K00973 glucose-1-phosphate thymidylyltransferase [EC:2.7.7.24] | (GenBank) glucose-1-phosphate thymi	Biosynthesis: secondary metabolites	399.393	325.385	329.291	171.726	127.146	447.136
AMK58_28940	K07126 uncharacterized protein | (GenBank) hypothetical protein	Hypothetical	119.231	135.603	141.825	352.020	131.947	222.040
AMK58_28945	no KO assigned | (GenBank) secretion protein	secretion system	536.774	540.255	519.179	158.486	200.585	344.161
AMK58_28950	no KO assigned | (GenBank) peptidase C39	peptidase	712.058	606.352	675.745	171.897	217.457	424.448
AMK58_28955	no KO assigned | (GenBank) hypothetical protein	Hypothetical	107.332	96.655	104.864	128.164	124.752	348.584
AMK58_28960	K02377 GDP-L-fucose synthase [EC:1.1.1.271] | (GenBank) GDP-fucose synthetase	Metabolism: Carbohydrate	134.363	98.506	122.723	170.097	124.061	278.620
AMK58_28965	K01990 ABC-2 type transport system ATP-binding protein | (GenBank) sugar ABC transporter ATP-bind	Membrane Transport	47.626	36.029	40.284	162.600	104.807	398.608
AMK58_28970	K09690 lipopolysaccharide transport system permease protein | (GenBank) ABC transporter permease	Transport: lipopolysaccharide	89.466	82.621	87.480	187.138	79.150	439.134
AMK58_28975	K01711 GDPmannose 4,6-dehydratase [EC:4.2.1.47] | (GenBank) GDP-mannose 4,6 dehydratase	Metabolism: Carbohydrate	77.468	62.706	74.964	186.957	88.060	442.822
AMK58_28980	K01711 GDPmannose 4,6-dehydratase [EC:4.2.1.47] | (GenBank) GDP-mannose 4,6 dehydratase	Metabolism: Carbohydrate	872.263	739.021	722.366	152.107	237.586	604.797
AMK58_28985	no KO assigned | (GenBank) hypothetical protein	Hypothetical	628.558	548.929	577.636	179.440	23.647	807.386
AMK58_28990	no KO assigned | (GenBank) hypothetical protein	Hypothetical	200.759	185.995	206.680	140.017	47.504	392.989
AMK58_28995	no KO assigned | (GenBank) hypothetical protein	Hypothetical	10.697	14.525	17.106	442.917	25.142	578.933
AMK58_29000	no KO assigned | (GenBank) hypothetical protein	Hypothetical	5.045	9.795	9.833	405.803	13.326	426.274
AMK58_29005	no KO assigned | (GenBank) hypothetical protein	Hypothetical	7.264	7.234	14.965	277.338	27.672	395.938
AMK58_29010	no KO assigned | (GenBank) hypothetical protein	Hypothetical	130.756	126.134	127.707	283.435	52.838	312.365
AMK58_29015	no KO assigned | (GenBank) hypothetical protein	Hypothetical	61.097	60.207	59.203	270.114	91.495	442.984
AMK58_29020	no KO assigned | (GenBank) hypothetical protein	Hypothetical	71.214	74.807	65.691	210.707	108.950	518.327
AMK58_29025	K16011 mannose-1-phosphate guanylyltransferase / mannose-6-phosphate isomerase [EC:2.7.7.13 5.3.1.8]	Metabolism: Carbohydrate	181.151	157.905	169.846	339.657	93.747	375.866
AMK58_29030	no KO assigned | (GenBank) hypothetical protein	Hypothetical	73.400	61.337	74.390	278.530	79.852	261.853
AMK58_29035	no KO assigned | (GenBank) hypothetical protein	Hypothetical	57.472	57.482	70.836	517.581	55.366	274.871
AMK58_29040	no KO assigned | (GenBank) hypothetical protein	Hypothetical	15.731	15.556	26.820	419.561	32.804	203.413
AMK58_29045	no KO assigned | (GenBank) hypothetical protein	Hypothetical	58.005	61.426	53.985	363.839	82.104	210.737
AMK58_29050	no KO assigned | (GenBank) hypothetical protein	Hypothetical	94.709	83.597	77.955	299.122	63.432	410.369
AMK58_29055	no KO assigned | (GenBank) hypothetical protein	Hypothetical	185.450	173.131	160.789	269.158	128.582	400.674
AMK58_29060	no KO assigned | (GenBank) hypothetical protein	Hypothetical	90.089	83.667	76.851	433.087	81.681	418.404
AMK58_29065	no KO assigned | (GenBank) hypothetical protein	Hypothetical	39.688	32.016	41.478	504.167	29.082	225.005
AMK58_29070	no KO assigned | (GenBank) hypothetical protein	Hypothetical	10.213	13.232	18.017	469.416	32.502	242.909
AMK58_29075	K00957 sulfate adenylyltransferase subunit 2 [EC:2.7.7.4] | (GenBank) sulfate adenylyltransferase	Metabolism	107.043	124.551	125.792	309.514	62.338	446.972
AMK58_29080	K00955 bifunctional enzyme CysN/CysC [EC:2.7.7.4 2.7.1.25] | (GenBank) adenylyltransferase	Metabolism	49.067	48.923	57.652	268.046	45.364	370.255
AMK58_29085	K08678 UDP-glucuronate decarboxylase [EC:4.1.1.35] | (GenBank) NAD-dependent dehydratase	Metabolism: Carbohydrate	174.802	148.439	138.502	171.816	85.058	502.056
AMK58_29090	no KO assigned | (GenBank) pirin	No COG	393.912	335.101	321.696	121.424	157.254	533.251
AMK58_29095	K03273 D-glycero-D-manno-heptose 1,7-bisphosphate phosphatase [EC:3.1.3.82 3.1.3.83] | (GenBank) D	Biosynthesis: polysacchharide	85.845	82.593	65.510	89.307	93.486	463.552
AMK58_29100	no KO assigned | (GenBank) hypothetical protein	Hypothetical	11.924	7.916	17.442	398.775	74.576	342.196
AMK58_29105	no KO assigned | (GenBank) hypothetical protein	Hypothetical	22.072	19.863	29.504	311.753	374.438	297.331
AMK58_29110	no KO assigned | (GenBank) hypothetical protein	Hypothetical	14.214	10.868	27.955	286.687	51.974	363.484
AMK58_29115	no KO assigned | (GenBank) hypothetical protein	Hypothetical	12.492	11.482	22.384	149.958	15.121	670.499
AMK58_29120	no KO assigned | (GenBank) hypothetical protein	Hypothetical	21.994	21.024	55.677	215.659	40.420	356.438
AMK58_29125	K03496 chromosome partitioning protein | (GenBank) hypothetical protein	Cell Cycle/Shape/Homeostatsis	108.028	104.169	121.495	153.039	54.958	368.127
AMK58_29130	K03497 chromosome partitioning protein, ParB family | (GenBank) hypothetical protein	Cell Cycle/Shape/Homeostatsis	76.020	90.310	111.630	459.428	48.169	502.288
AMK58_29135	K01840 phosphomannomutase [EC:5.4.2.8] | (GenBank) phosphomannomutase	Metabolism: Carbohydrate	304.757	248.847	272.875	252.174	140.940	421.571
AMK58_29140	K00012 UDPglucose 6-dehydrogenase [EC:1.1.1.22] | (GenBank) UDP-glucose 6-dehydrogenase	Metabolism: Carbohydrate	195.582	132.561	160.611	325.595	140.609	451.280

AMK58_29145	no KO assigned | (GenBank) hypothetical protein	Hypothetical	730.118	589.401	519.004	239.183	149.382	529.217
AMK58_29150	no KO assigned | (GenBank) UDP-4-amino-4,6-dideoxy-N-acetyl-beta-L-altrosamine transaminase	No COG	19.888	21.446	31.547	361.279	31.951	347.064
AMK58_29155	K15894 UDP-N-acetylglucosamine 4,6-dehydratase [EC:4.2.1.115] | (GenBank) UDP-N-acetylglucosamir	Metabolism: Carbohydrate	92.300	111.454	103.758	138.503	53.450	358.178
AMK58_29160	K14225 tRNA Gly | (GenBank) tRNA-Gly	tRNA synthesis/modification	48.430	57.868	53.722	661.718	59.881	1278.377
AMK58_29165	K10218 4-hydroxy-4-methyl-2-oxoglutarate aldolase [EC:4.1.3.17] | (GenBank) 4-hydroxy-4-methyl-2-oxo	Metabolism	66.238	74.606	89.135	420.617	65.097	481.132
AMK58_29170	no KO assigned | (GenBank) hypothetical protein	Hypothetical	13.667	15.370	18.269	308.864	25.032	350.079
AMK58_29175	no KO assigned | (GenBank) hypothetical protein	Hypothetical	15.737	19.714	24.858	638.543	17.689	359.640
AMK58_29180	no KO assigned | (GenBank) NUDIX hydrolase	hydrolase	49.977	33.732	45.729	138.376	23.035	495.615
AMK58_29185	no KO assigned | (GenBank) hypothetical protein	Hypothetical	47.347	57.409	51.828	425.007	53.612	381.036
AMK58_29190	no KO assigned | (GenBank) glycosyl transferase family 1	Glycosyl Transferase	21.832	25.129	23.805	516.875	30.393	487.431
AMK58_29195	no KO assigned | (GenBank) hypothetical protein	Hypothetical	111.505	193.889	122.635	406.858	43.961	536.657
AMK58_29200	K00947 molybdenum storage protein | (GenBank) molybdenum storage protein subunit alpha	No COG	26.240	26.412	34.983	550.744	39.273	320.279
AMK58_29205	K00947 molybdenum storage protein | (GenBank) uridine kinase	No COG	36.188	40.928	36.815	258.340	58.421	470.207
AMK58_29210	no KO assigned | (GenBank) hypothetical protein	Hypothetical	14.334	19.679	20.271	312.299	56.187	372.688
AMK58_29215	no KO assigned | (GenBank) hypothetical protein	Hypothetical	20.078	19.942	27.938	347.135	30.300	293.711
AMK58_29220	no KO assigned | (GenBank) radical SAM protein	No COG	133.713	117.639	122.289	294.909	58.314	261.509
AMK58_29225	K12454 CDP-paratose 2-epimerase [EC:5.1.3.10] | (GenBank) NAD-dependent epimerase	Metabolism: Carbohydrate	175.112	149.264	164.563	198.076	76.116	358.194
AMK58_29230	K12454 CDP-paratose 2-epimerase [EC:5.1.3.10] | (GenBank) 3-beta hydroxysteroid dehydrogenase	Metabolism: Carbohydrate	33.632	32.819	42.637	335.484	20.162	317.162
AMK58_29235	no KO assigned | (GenBank) hypothetical protein	Hypothetical	33.803	36.719	46.846	283.345	30.799	332.392
AMK58_29240	no KO assigned | (GenBank) hypothetical protein	Hypothetical	49.583	47.650	54.910	295.153	59.946	477.413
AMK58_29245	no KO assigned | (GenBank) hypothetical protein	Hypothetical	39.358	47.825	44.081	220.269	123.721	594.132
AMK58_29250	no KO assigned | (GenBank) hypothetical protein	Hypothetical	169.841	193.899	180.513	376.632	92.241	272.410
AMK58_29255	K01953 asparagine synthase (glutamine-hydrolysing) [EC:6.3.5.4] | (GenBank) hypothetical protein	Metabolism: Amino Acid	32.327	44.320	46.785	393.932	33.507	140.858
AMK58_29260	no KO assigned | (GenBank) hypothetical protein	Hypothetical	0.000	0.000	0.000	0.000	0.000	0.000
AMK58_29265	no KO assigned | (GenBank) hypothetical protein	Hypothetical	0.000	0.000	0.000	0.000	0.000	0.000
AMK58_29270	no KO assigned | (GenBank) hypothetical protein	Hypothetical	0.000	0.000	0.000	0.000	0.000	0.000
AMK58_29275	no KO assigned | (GenBank) hypothetical protein	Hypothetical	0.000	0.000	0.000	0.000	0.000	0.000
AMK58_29280	no KO assigned | (GenBank) hypothetical protein	Hypothetical	0.000	0.000	0.000	0.000	0.000	0.000
AMK58_29285	no KO assigned | (GenBank) hypothetical protein	Hypothetical	0.000	0.000	0.000	0.000	0.000	0.000
AMK58_29290	no KO assigned | (GenBank) hypothetical protein	Hypothetical	0.000	0.000	0.000	0.000	0.000	0.000
AMK58_29295	no KO assigned | (GenBank) hypothetical protein	Hypothetical	0.000	0.000	0.000	0.000	0.000	0.000
